# Supplementary material for: Asymmetric synthesis of stereogenic-at-sulfur compounds via biocatalytic oxidation with Unspecific Peroxygenases
Source: Nat Commun. 2025 Dec 12;17:646. doi: 10.1038/s41467-025-67405-0 (PMC12815920; doi:10.1038/s41467-025-67405-0)
Supplement: Supplementary file 1 — Supplementary Information [file 41467_2025_67405_MOESM1_ESM.pdf]

Supplementary Information

Asymmetric synthesis of stereogenic-at-sulfur compounds *via* biocatalytic oxidation with Unspecific Peroxygenases

Jiacheng Li,[†1] Benjamin Melling,[†1] Katy A. S. Cornish,[1,2] Nicholas Mulholland,[3] Jared Cartwright,[2] William P. Unsworth,*[1] Gideon Grogan.*[1]

[†]These authors contributed equally to this manuscript

* william.unsworth@york.ac.uk; gideon.grogan@york.ac.uk

[1] University of York, Department of Chemistry, Heslington, York, YO10 5DD (UK).

[2] University of York, Department of Biology, Heslington, York, YO10 5DD (UK).

[3] Syngenta, Jealott's Hill International Research Centre, Bracknell, Berkshire, RG42 6EY (UK).

Table of Contents

1) General Information	S3
2) Experimental Procedures	S4–S14
3) Compound data	S15–S73
4) Enzyme production	S74–S79
5) HPLC data	S80–S111
6) Molecular Docking	S112
7) ^{1}H, ^{13}C and ^{19}F NMR spectra	S113–S212
8) References	S213–S217

1. General Information

All reactions were performed in oven-dried glassware under a nitrogen atmosphere. Unless specified, all reagents and starting materials were purchased from commercial companies and used as received. Anhydrous solvents were freshly obtained from pure solvent system following standard procedures. Analytical thin layer chromatography (TLC) was performed using pre-coated silica gel plates. A Razel A 99 syringe pump was used for the slow addition of solutions. Visualisation was achieved by UV light (254 nm) or $KMnO_4$ and vanillin as stain. Flash chromatography was performed using silica gel and gradient solvent system (eluent: hexane: ethyl aetate/hexane: DCM). ^{1}H and ^{13}C NMR spectra were recorded on 400MHz Jeol ECS and Bruker AV and AM spectrometers. Chemical shifts (ppm) were recorded with tetramethylsilane (TMS) as the internal reference standard. Multiplicities are given as: s (singlet), br s (broad singlet), d (doublet), t (triplet), dd (doublet of doublets), dt (double of triples), ddd (doublet of doublet of doublets), ddt (doublet of doublet of triplets), dtt (doublet of triplet of triplets), ddq (doublet of doublet of quintets), dddd (doublet of doublet of doublet of doublets), dtd (doublet of triplet of doublets), dt (doublet of triplets), dp (doublet of pentents), dq (doublet of quintets), td (triplet of doublets), tdd (triplet of doublet of doublets), tt (triplet of triplets), qd (quintet of doublets), qt (quintet of triplets) or m (multiplet). The number of protons (*n*) for a given resonance is indicated by *n*H and coupling constants are reported as a *J* value in Hz. Enantiomeric excess values were measured by an Agilent 1200 series HPLC. Unless stated otherwise, all chiral HPLC runs were carried out at 20 °C. High resolution mass spectra (HRMS) were obtained on a LC/HRMS TOF spectrometer using simultaneous electrospray (ESI).

2. Experimental Procedures

General Procedure 2.1

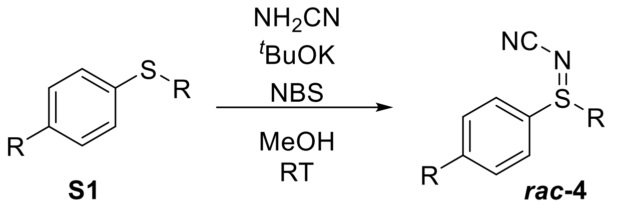

Adapted from Mancheno *et al.* [1] To a stirring solution of sulfide (4.08 mmol) in MeOH (10.0 mL) was added NH_2CN (2.00 eq.). Following this was added *t*BuOK (1.70 eq.) portion-wise followed by addition of NBS (1.30 eq.). The reaction was allowed to stir overnight. The solvent was removed *in vacuo* and the crude dissolved in saturated $Na_2S_2O_3$ (10 mL) which was extracted with EtOAc (3 x 30 mL), and the combined organic phase washed with saturated brine (30 mL), dried over $MgSO_4$, filtered, and the solvent removed *in vacuo* to afford the crude. Purification was *via* done flash column chromatography on silica gel.

General Procedure 2.2

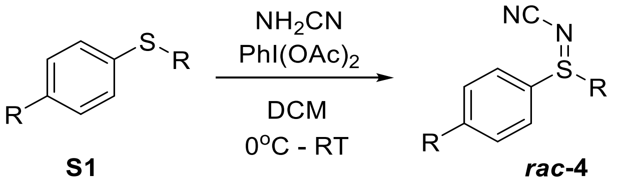

To a stirring solution of sulfide (4.08 mmol) and NH_2CN (2.00 eq.) in DCM (20.0 mL) at 0 °C were added $PhI(OAc)_2$ (1.10 eq.), after which the temperature was raised to RT. After stirring overnight, the solvent was removed *in vacuo* and the crude material dissolved in saturated $Na_2S_2O_3$ (10 mL). Following extraction with EtOAc (3 x 30 mL), the combined organic phase washed with saturated brine (30 mL), dried over $MgSO_4$, filtered, and the solvent removed *in vacuo*. Purification was done *via* flash column chromatography on silica gel.

2.3 Synthesis of Racemic Sulfoximine Standards

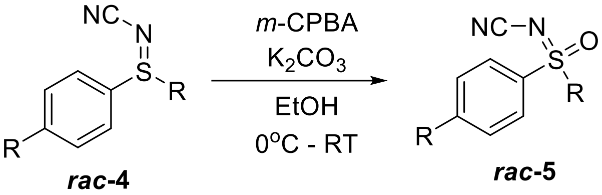

To a stirring solution of sulfilimine (0.600 mmol) in EtOH (5.00 mL) at 0 °C were added K_2CO_3 (3.00 eq.) followed by *m*-CPBA (2.00 eq.) before raising the reaction to RT. After overnight stirring, the solvent was removed *in vacuo* to afford a white solid, which was dissolved in saturated aq. $Na_2S_2O_3$ (10 mL) and extracted with EtOAc (3 x 30 mL). The combined organic phase was washed with saturated brine (50 mL), dried over $MgSO_4$, filtered and the solvent removed *in vacuo* to afford crude material which was purified, where required, *via* flash column chromatography on silica gel.

2.4 General Procedure for 0.3 mmol artUPO Kinetic Resolution of Sulfilimines 4

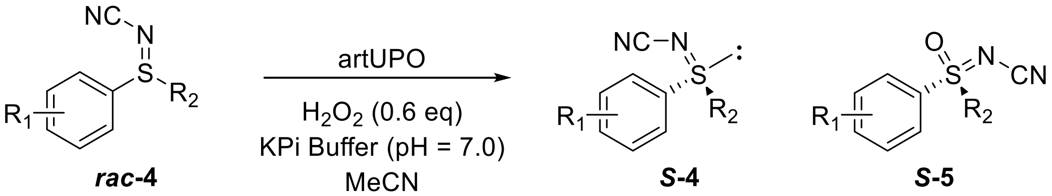

Liquid artUPO secretate (1.00 mL, 0.8 U/mL) was added to KPi Buffer (24.0 mL, pH 7.00) at RT and stirred for five min, after which, a solution of sulfilimine (0.3 mmol) in MeCN (6.00 mL) was added. The reaction was initiated by the slow continuous addition of a H_2O_2 solution (0.180 mmol in 2 mL H_2O) over 4 h followed by stirring overnight. The reaction was extracted with Et_2O (3 x 30 mL), and the combined organic phase washed with saturated brine (40 mL), dried over $MgSO_4$, filtered and the solvent removed *in vacuo* to afford the crude. For preparative reactions, the purified product were isolated following flash column chromatography on silica gel.

2.5 General Procedure for ≈1.0 mmol artUPO Kinetic Resolution of Sulfilimines 4

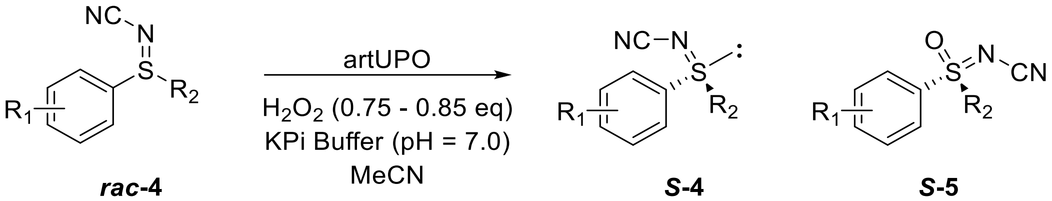

Liquid artUPO secretate (2.50 mL, 0.8 U/mL) was added to KPi Buffer (40.0 mL, pH 7.0) at RT and stirred for five min, after which, a solution of sulfilimine (1.00 mmol) in MeCN (10.0 mL) was added. The reaction was initiated by the slow continuous addition of a H_2O_2 solution (0.600–0.800 mmol in 2.00 mL H_2O, see below for specific amounts for individual substrates) over 4 h. After H_2O_2 addition was complete, a sample of the reaction mixture (1.00 mL) was subjected to standard aqueous work-up (extract with EtOAc, wash with brine) before analysis of the resulting sample using ^{1}H NMR to measure conversion. If 50% conversion was not observed, a further 0.05–0.15 equivalents of H_2O_2 was added over 1 h, before re-analysing as described above. When 50% conversion was reached, the reaction was extracted with Et_2O (3 x 30 mL), and the combined organic phase washed with saturated brine (40 mL), dried over $MgSO_4$, filtered and the solvent removed *in vacuo* to afford the crude. Purified products for preparative reactions were isolated following flash column chromatography on silica gel.

H_2O_2 quantities used for scale ups:

rac-4b: Initial H_2O_2 infusion of 0.60 equiv., followed by an additional 0.10 equiv. H_2O_2 infusion. 1.07 mmol scale.

rac-4e: Initial H_2O_2 infusion of 0.65 equiv., followed by an additional 0.15 mmol H_2O_2 infusion. 1.20 mmol scale.

rac-4j: Initial H_2O_2 infusion of 0.70 equiv. No additional H_2O_2 infusion. 1.00 mmol scale.

rac-4t: Initial H_2O_2 infusion of 0.75 equiv., followed by an additional 0.05 equiv. H_2O_2 infusion. 1.07 mmol scale.

rac-4u: Initial H_2O_2 infusion of 0.80 equiv. No additional H_2O_2 infusion. 1.00 mmol scale.

2.5.1 Time course for artUPO-catalyzed oxidation of 4a to form 5a.

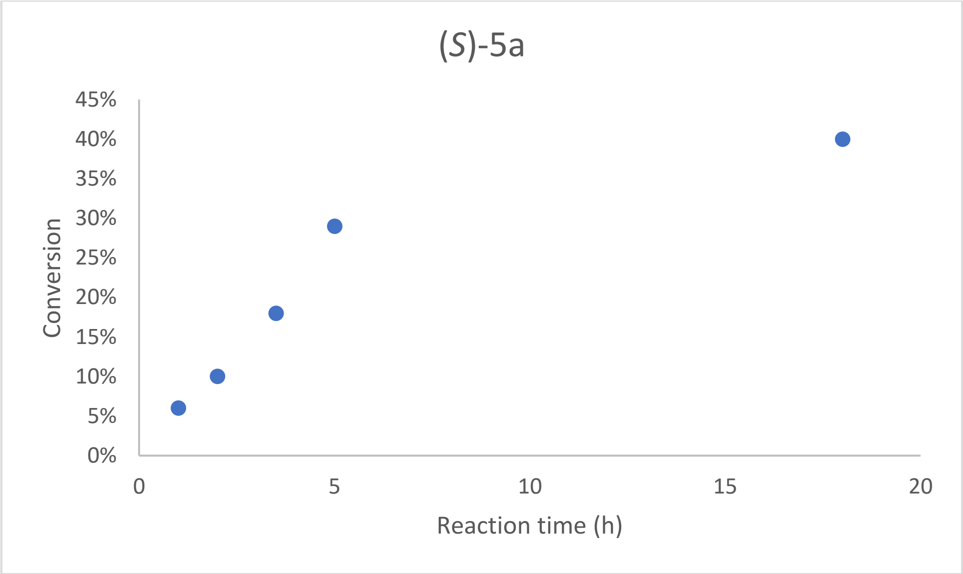

Figure S1. Time course experiment for conversion of substrate **4a** (0.1 mmol, 10 mM) into **(*S*)-5a** by artUPO; H_2O_2 addition rate: 15 µmol/h; conversion was measured by ^1H NMR.

2.6. Screening for UPOs

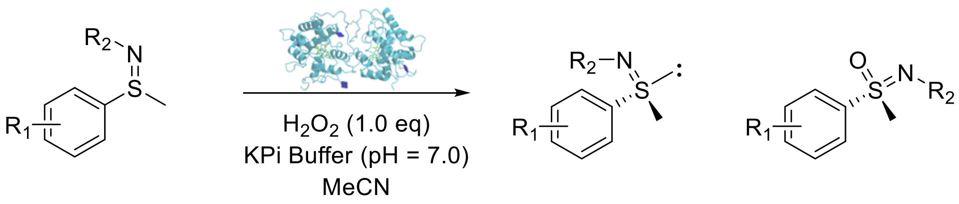

In order to investigate the feasibility of successfully sulfilimine oxidation by UPOs, we first synthesised three substrates to test for activity. Unless otherwise specified, the reactions were carried out following general procedure **2.4** with 0.2 mmol substrate. Unless otherwise specified, conversion was determined by comparing the intensity of the methyl peaks of the sulfilimine and sulfoximine in the ^1H NMR spectra of the crude material.

Entry	R_1	R_2	Conversion (%)				
			without UPO	r*Aae*UPO-PaDa-I-H	art-UPO	*Cma*UPO	*Dca*UPO
1	H	CN	0	11	39	12	36
2	H	$C(O)CF_3$	-	0	14	-	-
3	4-MeO	CN	-	11	65[a,b]	-	-

[a] Reaction carried out on a 60 mL scale with 0.6 mmol substrate
[b] Isolated yield following purification via flash chromatography on silica gel

Sulfilimines **4l** and **4m** were also tested using r*Aae*UPO-PaDa-I under the conditions used above, with 0% conversion observed, further validating our decision to prioritise artUPO for this substrate class.

2.7 General procedure for the synthesis of *i*-Pr *N*-sulfenylimines (6) [2]

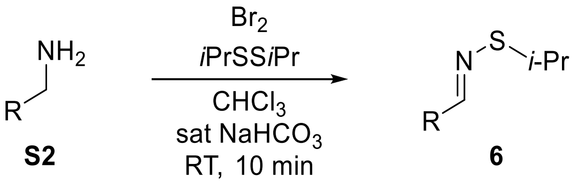

To a round bottom flask containing a magnetic stirring bar was added the appropriate amine **S2** (6 mmol, 3.0 equiv.) and diisopropyl disulfide (2 mmol, 0.34 mL, 1.0 equiv.) in $CHCl_3$ (24 mL) and sat. aq. $NaHCO_3$ (40 mL). The resulting reaction mixture was stirred vigorously by adding Br_2 (10 mmol, 0.52 mL, 5.0 equiv.) solution in DCM (4 mL) slowly. After the addition, the reaction mixture was stirred at room temperature for a further 10 min, open to air. Upon completion (a colour change to light yellow was noted), the reaction mixture was quenched by the addition of saturated aq. $Na_2S_2O_3$ (30 mL). The reaction mixture was then extracted with DCM (3 × 20 mL). The combined organic phases were washed with brine (20 mL), dried over anhydrous $MgSO_4$ and concentrated *in vacuo* to give a residue that was purified by flash column chromatography on silica gel (eluent specified later for each compound) to give the corresponding *N*-sulfenylimines (**6**).

2.8 General procedure for the synthesis of *t*-Bu *N*-sulfenylimines (6) [3]

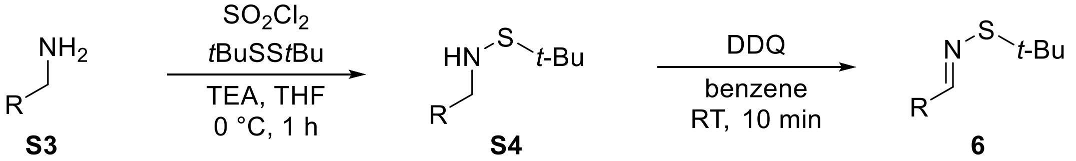

To a flame dried round bottom flask containing a magnetic stirring bar was added the di-*t*-butyl disulfide (5 mmol, 1.0 mL, 1.0 equiv.) anhydrous THF (40 mL) at 0 °C under an argon atmosphere. The sulfuryl dichloride (5.5 mmol, 0.45 mL, 1.1 equiv.) was added slowly and the resulting reaction mixture was stirred at the same temperature for 30 min. Upon completion, the solution was slowly transferred to another flame dried round bottom flask containing the appropriate amine **S3** (10 mmol, 2.0 equiv.) and TEA (15 mmol, 2.0 mL, 3.0 equiv.) in anhydrous THF (20 mL) at 0 °C. The resulting mixture was stirred at the same temperature for another 30 min. On completion, the reaction mixture was filtrated through a pad of Celite and rinsed with EtOAc (10 mL). The filtrate was concentrated under reduced pressure to give a residue that was purified by flash column chromatography on silica gel to give the corresponding compound **S4**.

To a flame dried round bottom flask containing a magnetic stirring bar was added **S4** (1.0 equiv.) in anhydrous benzene (10 mL) under an argon atmosphere. DDQ (1.1 equiv.) was added to the solution in one portion and the resulting mixture was stirred at room temperature for 10 min. Upon completion, the reaction was quenched by the addition of saturated aq. $NaHCO_3$ (50 mL). The reaction mixture was then extracted with DCM (3 × 20 mL). The combined organic phases were washed with water (50 mL) and brine (20 mL), dried over anhydrous $MgSO_4$ and concentrated *in vacuo* to give a residue that was purified by flash column chromatography on silica gel (eluent specified later for each compound) to give the corresponding *N*-sulfenylimines (**6**).

2.9 General procedure for the r*Aae*UPO-PaDa-I-H biotransformations with *i*-Pr *N*-sulfenylimines

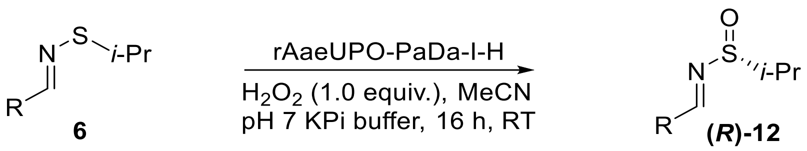

To a round bottom flask containing a magnetic stirring bar was added r*Aae*UPO-PaDa-I-H (1.3 mL, 57 U/mL) and KPi buffer (10 mL, 0.1 mmol/mL, pH = 7, 10 mL). The solution was diluted by the addition of deionised water (2.7 mL), followed by addition of the appropriate *i*-Pr *N*-sulfenylimines **6** (0.2 mmol, 1.0 equiv.,) in MeCN (4 mL). Next, H_2O_2 solution (2 mL, 0.1 mmol/ml, 1.0 equiv.) was added over a 10 h period, using a syringe pump. After the H_2O_2 addition was complete, the reaction was then stirred at room temperature for a further 6 h. The reaction mixture was then extracted with ethyl acetate (3 × 20 mL). The combined organic phases were then washed with brine (20 mL), dried over anhydrous $MgSO_4$ and concentrated *in vacuo* to give the crude product mixture, which was purified by flash column chromatography on silica gel (eluent: specified later for each compound) to provide the corresponding *N*-sulfinyl imine product **(*R*)-12**.

2.9.1. Time course for r*Aae*UPO-PaDa-I-H catalyzed oxidation of 6a-*i*Pr to (*R*)-13a.

Time	Conversion
	0
2	4%
4	24%
6	50%
10	83%
16	89%

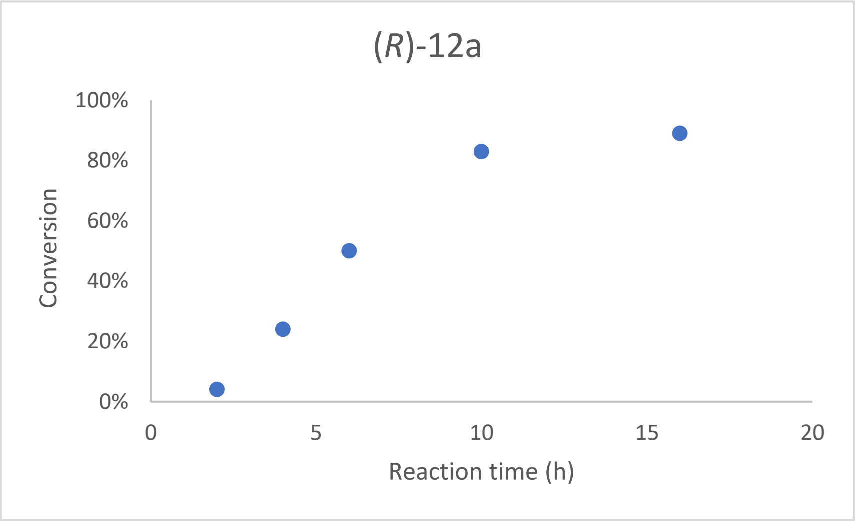

Figure S2. Time course experiment for the conversion of substrate **6a-iPr** (0.1 mmol, 10 mM) into **(*R*)-12a** by r*Aae*UPO-PaDa-I-H; H_2O_2 addition rate: 10 µmol/h; conversion was measured by ^{1}H NMR. This conversion corresponds to a TTN (total turnover number expressed as µmol product/µmol enzyme) of approximately 1.57×10^4.

2.9.2 Screening of alternative UPOs for the oxidation of 6a (*i*-Pr and *t*-Bu)

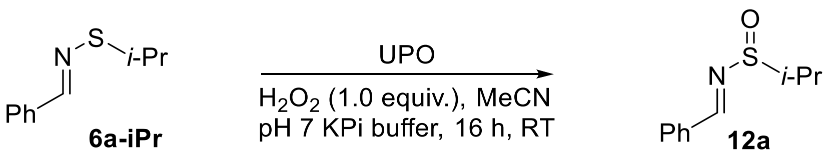

Enzyme	Conversion	*er*
*Dca*UPO (Class I)	88%	83:17 (*S*)
*Cma*UPO (Class II)	100%	16:84 (*R*)
No UPO	13%	50:50 (racemic)

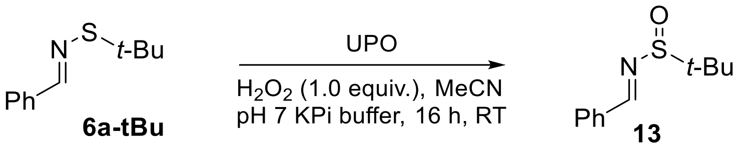

Enzyme	Conversion	*er*
DcaUPO (Class I)	73%	90:10 (*S*)
CmaUPO (Class II)	60%	8:92 (*R*)
No UPO	7%	50:50 (racemic)

2.10 General procedure for the artUPO biotransformations with *t*-Bu *N*-sulfenylimines

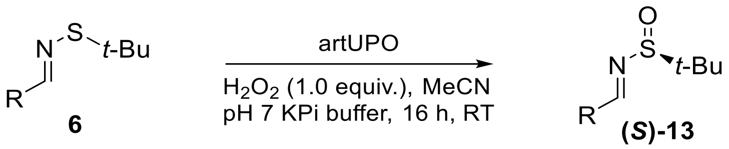

To a round bottom flask containing a magnetic stirring bar was added liquid artUPO secretate (1.0 mL, 0.8 U/mL) and KPi buffer (100 mM, pH = 7, 10 mL). The solution was diluted by the addition of deionised water (3 mL), followed by addition of the appropriate *t*-Bu *N*-sulfenylimine **6** (0.2 mmol, 1.0 equiv., final concentration 10 mM) in MeCN (4 mL). Next, 2 mL of a 100 mM H_2O_2 solution (prepared from 22 μL 30% H_2O_2 in 2 mL deionised water) was added over a 10 h period, using a syringe pump. After the H_2O_2 addition was complete, the reaction was then stirred at room temperature for a further 6 h. The reaction mixture was then extracted with ethyl acetate (3 × 20 mL). The combined organic phases were then washed with brine (20 mL), dried over anhydrous $MgSO_4$ and concentrated *in vacuo* to give the crude product mixture, which was purified by flash column chromatography on silica gel (eluent: specified later for each compound) to provide the corresponding *N*-sulfinyl imine product **(*S*)-13**.

2.10.1 Time course for artUPO-catalyzed oxidation of 6a-tBu to from (*S*)-13a.

Time	Conversion
0	0
2	15%
4	46%
6	60%
10	76%
16	100%

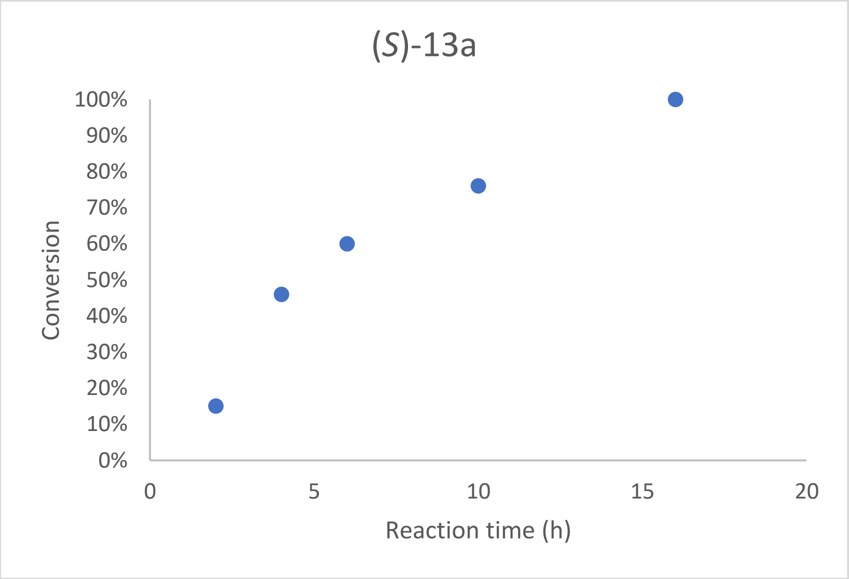

Figure S3. Time course for the conversion of substrate **6a-tBu** (0.1 mmol, 10 mM) into **(*S*)-13a** by artUPO; H_2O_2 addition rate: 10 µmol/h; conversion was measured by ^{1}H NMR. This conversion corresponds to a TTN (total turnover number expressed as µmol product/µmol enzyme) of approximately 5.88 x 10^3.

2.11 Racemic *N*-sulfinyl imine synthesis [4]

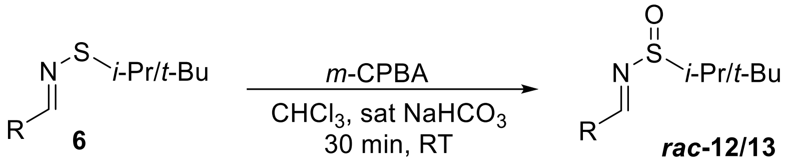

To a round bottom flask containing a magnetic stirring bar was added the corresponding *N*-sulfenylimine (0.1 mmol, 1.0 equiv.) in $CHCl_3$ (3 mL), which was followed by the addition of sat $NaHCO_3$ (0.4 mL) and *m*-CPBA (25 mg, 0.11 mmol, 1.1 equiv.) in one portion and the resulting reaction mixture was stirred at room temperature for 30 min. Upon completion, the reaction mixture was quenched by adding excess neat KOH and dried over anhydrous $MgSO_4$ which was then filtered and concentrated *in vacuo* to give the crude product mixture. The products afforded were of sufficient purity to be used as standards for HPLC analysis without additional purification.

3. Compound data

N-(cyano) methyl phenyl sulfilimine (4a) [1]

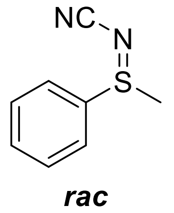

Synthesised from phenyl methyl sulfane using general procedure **2.1**. Purification via flash chromatography on silica gel (eluent: hexane: EtOAc = 2:3). Data for **4a:** as a yellow oil (0.560 g, 84% yield); ^{1}H NMR (400 MHz, *chloroform-d*) δ 7.82 – 7.78 (m, 2H), 7.66 – 7.58 (m, 3H), 3.02 (s, 3H); ^{13}C NMR (101 MHz, *chloroform-d*) δ 136.3, 133.3, 130.5, 126.1, 120.4, 36.6.

N-(cyano) methyl 4-methoxyphenyl sulfilimine (4b) [1]

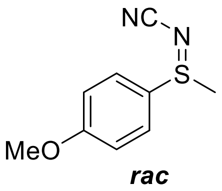

Synthesised from (4-methoxyphenyl)(methyl)sulfane using general procedure **2.1.** Purification via flash chromatography on silica gel (eluent: EtOAc: MeOH = 19:1). Data for **4b:** as a white solid (0.630 g, 80% yield); ^{1}H NMR (400 MHz, *chloroform-d)* δ 7.78 – 7.69 (m, 2H), 7.11 – 7.02 (m, 2H), 3.86 (s, 3H), 3.00 (s, 3H); ^{13}C NMR (101 MHz, *chloroform-d*) δ 163.7, 128.5, 126.7, 120.6, 115.8, 55.9, 36.2; HRMS (ESI, m/z) m/z calculated for $C_9H_{10}N_2NaOS$ $(MNa)^+$ 217.0407, found 217.0412.

N-(cyano) methyl 3-methoxyphenyl sulfilimine (4c)

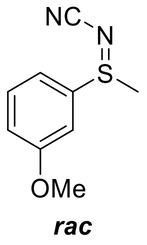

Synthesised from (3-methoxyphenyl) (methyl)sulfane using general procedure **2.1**. Purification via flash column chromatography on silica gel (eluent: hexane: EtOAc = 4:1); as a light yellow oil (0.500 g, 63 % Yield); ^{1}H NMR (400 MHz, *chloroform-d*) δ 7.47 (t, *J* = 8.0 Hz, 1H), 7.37 – 7.24 (m, 2H), 7.15 – 7.08 (m, 1H), 3.87 (s, 2H), 2.99 (s, 3H); ^{13}C NMR (101 MHz, *chloroform-d*) δ 160.9, 137.4, 131.4, 120.5, 119.4, 117.9, 110.5, 55.9, 36.9; HRMS (ESI, m/z) m/z calculated for $C_9H_{10}N_2NaOS$ $(MNa)^+$ 217.0412, found 217.0399; IR film: vmax/cm^{-1} 3007, 2141 (C≡N stretch), 1593, 1481, 1241, 1148, 1029, 760, 680.

N-(cyano) methyl 4-chlorophenyl sulfilimine (4d) [5]

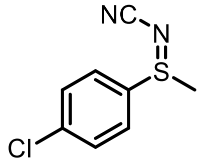

Synthesised from (4-chlorophenyl) (methyl) sulfane using general procedure **2.1.** Purification via flash column chromatography on silica gel (eluent: hexane: EtOAc = 3:7); as a white solid (0.320 g, 40% Yield); ^{1}H NMR (400 MHz, *chloroform-d*) δ 7.78 – 7.72 (m, 2H), 7.62 – 7.57 (m, 2H), 3.02 (s, 3H); ^{13}C NMR (101 MHz, *chloroform-d*) δ 140.0, 134.8, 130.8, 127.5, 36.8; HRMS (ESI, m/z) m/z calculated for $C_8H_7{}^{35}ClN_2NaS$ $(MNa)^+$ 220.9916, found 220.9914.

N-(cyano) methyl 4-trifluorophenyl sulfilimine (4e) [6]

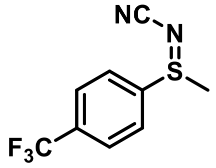

Synthesised from (4-(trifluoromethyl) phenyl (methyl) sulfane using general procedure **2.1.** Purification via flash chromatography on silica gel (eluent: hexane: EtOAc = 2: 3); as a yellow oil (0.700 g, 74 % Yield); ^{1}H NMR (400 MHz, *chloroform-d*) δ 7.95 (d, J = 8.7 Hz, 2H), 7.89 (d, J = 8.7 Hz, 2H), 3.07 (s, 3H); ^{13}C NMR (151 MHz, *Chloroform-d*) δ 140.5, 135.1 (q, J = 33.5 Hz), 127.4 (q, J = 3.8 Hz), 126.4, 123.0 (q, J = 273.4 Hz), 119.6, 36.8; ^{19}F NMR (376 MHz, *chloroform-d*) δ –63.09; HRMS (ESI, m/z) m/z calculated for $C_9H_7F_3N_2NaS$ $(MNa)^+$ 255.0180, found 255.0181.

N-(cyano) methyl 4-cyanophenyl sulfilimine (4f) [7]

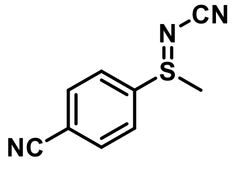

Synthesised from (4-cyanophenyl) (methyl) sulfane using general procedure **2.1.** Purification via flash chromatography on silica gel (eluent: hexane: EtOAc = 3:7); as a white solid (0.640 g, 83% yield); ^{1}H NMR (400 MHz, *chloroform-d*) δ 7.96 – 7.88 (m, 4H), 3.06 (s, 3H); ^{13}C NMR (101 MHz, *chloroform-d*) δ ^{13}C NMR (101 MHz, CHLOROFORM-*D*) δ 141.5, 134.0, 126.7, 119.6, 117.0, 117.0, 37.0.

N-(cyano) methyl 4-methylphenyl sulfilimine (4g)

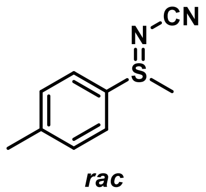

Synthesised from p-tolyl sulfane using general procedure **2.1.** Purification via flash chromatography on silica gel (eluent: hexane: EtOAc = 7:11); as a white solid (0.580 g, 80% Yield); Mp = 86 – 89 °C; ^{1}H NMR (400 MHz, *chloroform-d*) δ 7.71 – 7.64 (m, 2H), 7.43 – 7.35 (m, 2H), 3.00 (s, 3H), 2.44 (s, 3H); ^{13}C NMR (101 MHz, *chloroform-d*) δ 144.4, 133.0, 131.1, 126.2, 120.6, 36.4, 21.7; HRMS (ESI, m/z) m/z calculated for $C_9H_{10}N_2NaS$ $(MNa)^+$ 201.0462, found 201.0455; IR film: νmax/cm^{-1} 2996, 2136, 1592, 1416,1147, 750, 559, 501.

N-(cyano) methyl 3-nitrophenyl sulfilimine (4h)

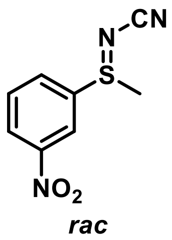

Synthesised from (3-nitrophenyl) (methyl) sulfane using general procedure **2.2.** Purification via flash chromatography on silica gel (eluent: hexane: EtOAc = 1:4); as a red oil (0.290 g, 32% Yield); ^{1}H NMR (400 MHz, *chloroform-d*) δ 8.61 (t, *J* = 2.1 Hz, 1H), 8.50 (ddd, *J* = 8.2, 2.1, 1.0 Hz, 1H), 8.26 – 8.18 (m, 1H), 7.89 (t, *J* = 8.2 Hz, 1H), 3.12 (s, 3H); ^{13}C NMR (101 MHz, *chloroform-d*) δ 149.0, 139.0, 132.0, 131.5, 127.7, 121.3, 119.5, 37.2; HRMS (ESI, m/z) m/z calculated for $C_8H_7N_3NaO_2S$ $(MNa)^+$ 232.0157, found 232.0157; IR film: νmax/cm^{-1} 3069, 2922, 2153, 1525, 1347, 1170, 734.

N-(cyano) methyl 3-chlorophenyl sulfilimine (4i)

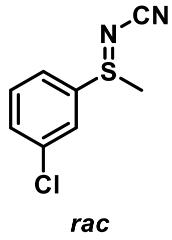

Synthesised from (3-chlorophenyl) (methyl) sulfane using general procedure **2.1.** Purification via flash chromatography on silica gel (gradient eluent: hexane: EtOAc = 1:1 to EtOAc); as a white solid (0.670 g, 84% Yield); M.p. = 79 – 81 °C; ^{1}H NMR (400 MHz, *chloroform-d*) δ 7.78 – 7.76 (m, 1H), 7.70 – 7.66 (m, 1H), 7.61 – 7.53 (m, 2H), 3.02 (s, 3H); ^{13}C NMR (101 MHz, *chloroform-d*) δ 138.2,

136.7, 133.4, 131.7, 125.9, 124.0, 37.1; HRMS (ESI, m/z) m/z calculated for $C_8H_7{}^{35}ClN_2NaS$ $(MNa)^+$ 220.9916, found 220.9911.

***N*-(cyano) methyl 3-chloro-4-(trifluoromethyl)phenyl sulfilimine (4j)**

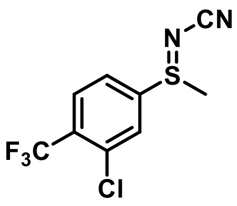

rac

Synthesised from (3-chlorophenyl)-(methyl) sulfane using general procedure **2.1.** Purififcation via flash chromatography on silica gel (eluent: EtOAc); as a yellow oil (0.540 g, 82% Yield); ^{1}H NMR (400 MHz, *chloroform-d*) δ 7.96 – 7.92 (m, 2H), 7.83 – 7.79 (m, 1H), 3.07 (s, 3H); ^{13}C NMR (101 MHz, *chloroform-d*) δ 141.8, 135.1, 132.6 (q, *J* = 32.4 Hz), 129.6 (q, *J* = 4.9 Hz) 128.5, 124.1, 122.0 (q, *J* = 273.8 Hz), 37.3; ^{19}F NMR (376 MHz, *chloroform-d*) δ -63.1; HRMS (ESI, m/z) m/z calculated for $C_9H_6{}^{35}ClF_3N_2NaS$ $(MNa)^+$ 288.9790, found 288.9792; IR film: νmax/cm^{-1} 3043, 2149, 1606, 1403, 1321, 1168, 1126, 840, 700, 595.

***N*-(cyano) methyl 2-pyridyl sulfilimine (4k) [1]**

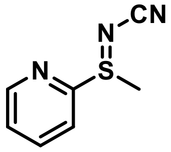

rac

Synthesised from 2-pyridyl methyl sulfane using general procedure **2.1**. Purification via flash chromatography on silica gel (eluent: EtOAc); as a white solid (0.450 g, 66% Yield); ^{1}H NMR (400 MHz, *chloroform-d*) δ 8.67 – 8.64 (m, 1H), 8.07 (dt, *J* = 7.8, 1.1 Hz, 1H), 8.00 (td, *J* = 7.8, 1.1 Hz, 1H), 7.50 (ddd, *J* = 7.5, 4.7, 1.1 Hz, 1H), 3.15 (s, 3H); ^{13}C NMR (101 MHz, *chloroform-d*) δ 156.6, 150.6, 139.1, 126.2, 121.8, 120.1, 119.3, 33.9; HRMS (ESI, m/z) m/z calculated for $C_7H_7N_3NaS$ $(MNa)^+$ 188.0258, found 188.0251.

N-(cyano) methyl (4-trifluoromethyl-2-pyridyl) sulfilimine (4l)

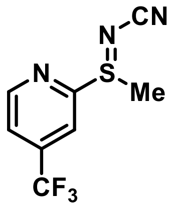

rac

Synthesised from 2-(methylthio)-4-(trifluoromethyl) pyridine. Purification via flash chromatography (eluent: hexane: EtOAc = 2:3); as a yellow oil (0.650 g, 71% Yield); ^{1}H NMR (400 MHz, chloroform-*d*) δ 8.89 (d, J = 5.1 Hz, 1H), 8.31 (s, 1H), 7.75 (d, J = 5.0 Hz, 1H), 3.22 (s, 3H); ^{13}C NMR (101 MHz, chloroform-*d*) δ 158.8, 151.8, 141.7 (q, J = 34.7 Hz), 122.1 (q, J = 3.4 Hz), 121.9 (q, J = 274 Hz), 119.6, 118.1 (q, J = 3.4 Hz), 34.5; HRMS (ESI, m/z) m/z calculated for $C_8H_6F_3N_3NaS$ $(MNa)^+$ 256.0132, found 256.0129; IR film: νmax/cm^{-1} 3075, 2150, 1322, 1172, 1129, 858, 660, 460.

N-(cyano) methyl (5-trifluoromethyl-2-pyridyl) sulfilimine (4m)

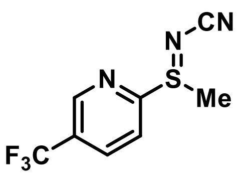

rac

Synthesised from 2-(methylthio)-5-(trifluoromethyl) pyridine. Purifciation via flash chromatography (eluent: hexane: EtOAc = 2:3); as a red oil (0.450 g, 49% Yield); ^{1}H NMR (400 MHz, chloroform-*d*) δ 8.97 – 8.92 (s, 1H), 8.27 (d, J = 1.5 Hz, 2H), 3.23 (s, 3H); ^{13}C NMR (101 MHz, *chloroform-d*) δ 160.7, 147.7 (q, J = 3.5 z), 136.5 (q, J = 3.5 Hz), 129.2 (q, J = 33.8 Hz), 122.6 (J = 273.2 Hz), 122.1, 119.5, 34.1; ^{19}F NMR (376 MHz, *chloroform-d*) δ -62.41; HRMS (ESI, m/z) m/z calculated for $C_8H_6F_3N_3NaS$ $(MNa)^+$ 256.0127, found 256.0129; IR film: νmax/cm^{-1} 3014, 2152, 1713, 1592, 1324, 1101, 1071, 773, 549.

N-(cyano) ethyl phenyl sulfilimine (4n)

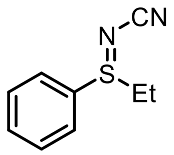

rac

Synthesised from ethyl phenyl sulfane using general procedure **2.1.** Purification via flash chromatography on silica gel (gradient eluent: hexane: EtOAc = 1:1 to EtOAc); as a colourless oil (1.26 g, 98% Yield); ^{1}H NMR (400 MHz, *chloroform-d*) δ 7.78 – 7.74 (m, 2H), 7.65 – 7.56 (m, 3H), 3.26 (dq, J = 13.1, 7.4 Hz, 1H), 3.13 (dq, J = 13.1, 7.4 Hz, 1H), 1.37 (t, J = 7.4 Hz, 3H); ^{13}C NMR (101 MHz, *chloroform-d*) δ

134.6, 133.2, 130.3, 126.5, 121.3, 46.7, 8.1; HRMS (ESI, m/z) m/z calculated for $C_9H_{10}N_2NaS$ $(MNa)^+$ 201.0462, found 201.0463; IR film: νmax/cm^{-1} 3059, 2141, 1711, 1444, 1170, 686, 583.

N-(cyano) propyl phenyl sulfilimine (4o)

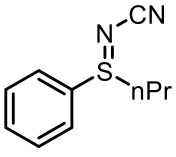

rac

Synthesised from propyl phenyl sulfide using general procedure **2.1**. Purification via flash chromatography on silica gel (eluent: hexane: EtOAc = 2:8); as a colourless oil (0.590 g, 76% Yield); ^{1}H NMR (400 MHz, *chloroform-d*) δ 7.83 – 7.69 (m, 2H), 7.67 – 7.51 (m, 3H), 3.28 (ddd, J = 12.9, 7.9, 6.2 Hz, 1H), 2.99 (ddd, J = 12.9, 8.4, 7.3 Hz, 1H), 1.91 – 1.73 (m, 2H), 1.09 (t, J = 7.4 Hz, 3H); ^{13}C NMR (101 MHz, *chloroform-d*) δ 135.2, 133.2, 130.4, 126.4, 121.2, 54.4, 17.3, 13.0; HRMS (ESI, m/z) m/z calculated for $C_{10}H_{12}N_2NaS$ $(MNa)^+$ 217.0412, found 217.0407; IR film: νmax/cm^{-1} 2967, 2934, 2141, 1444, 1149, 747, 668, 486.

N-(cyano) iso-propyl phenyl sulfilimine (4p) [6]

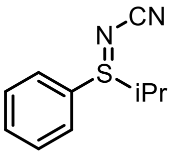

rac

Synthesised from isopropyl phenyl sulfane using general procedure **2.1.** Purification via flash chromatography on silica gel (eluent: hexane: EtOAc = 1:4 to EtOAc); as a colourless oil (0.630 g, 81% Yield); ^{1}H NMR (400 MHz, *chloroform-d*) δ 7.76 – 7.72 (m, 2H), 7.65 – 7.55 (m, 3H), 3.34 (hept, J = 6.8 Hz, 1H), 1.35 (d, J = 6.8 Hz, 3H), 1.26 (d, J = 6.8 Hz, 3H); ^{13}C NMR (101 MHz, *chloroform-d*) δ 133.1, 132.9, 130.1, 127.2, 122.2, 54.3, 16.9, 15.6; HRMS (ESI, m/z) m/z calculated for $C_{10}H_{12}N_2NaS$ $(MNa)^+$ 215.0613, found 215.0613; IR film: νmax/cm^{-1} 3059, 2145, 1633, 1443, 1155, 1081, 747, 483.

***N*-(cyano) cyclopropyl phenyl sulfilimine (4q)**

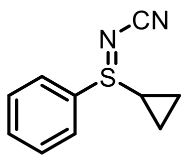

rac

Synthesised from cyclopropyl phenyl sulfane using general procedure **2.1.** Purification via flash chromatography on silica gel (eluent hexane: EtOAc = 3:7); as a golden oil (0.750 g, 70% yield); ^{1}H NMR (400 MHz, *chloroform-d*) δ 7.83 – 7.73 (m, 1H), 7.66 – 7.54 (m, 2H), 2.76 – 2.64 (m, 1H), 1.49 – 1.34 (m, 1H), 1.31 – 1.14 (m, 3H); ^{13}C NMR (101 MHz, *chloroform-d*) δ 136.0, 132.9, 130.2, 128.6, 128.5, 127.3, 126.4, 121.3, 29.7, 28.5, 5.6, 4.6; HRMS (ESI, m/z) m/z calculated for $C_{10}H_{10}N_2NaS$ $(MNa)^+$ 213.0462, found 213.0461; IR film: vmax/cm^{-1} 3025, 2143, 1703, 1444, 1156, 747, 685, 467.

***N*-(cyano) benzyl phenyl sulfilimine (4r) [8]**

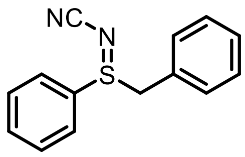

rac

Synthesised from benzyl phenyl sulfane using general procedure **2.2.** Purification via flash chromatography on silica gel (eluent: hexane: EtOAc = 1:4); as a white, gum-like solid (1.08 g, 90% Yield); ^{1}H NMR (400 MHz, *chloroform-d*) δ 7.67 – 7.56 (m, 3H), 7.56 – 7.47 (m, 2H), 7.41 – 7.21 (m, 3H), 7.18 – 7.11 (m, 2H), 4.56 (d, *J* = 12.6 Hz, 1H), 4.27 (d, *J* = 12.6 Hz, 1H); ^{13}C NMR (101 MHz, *chloroform-d*) δ 134.3, 133.3, 130.8, 130.1, 129.8, 129.3, 127.4, 126.9, 120.9, 58.8.

***N*-(cyano) phenethyl methyl sulfilimine (4s)**

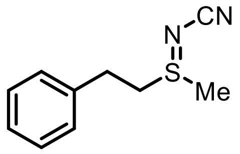

rac

Synthesised from phenethyl methyl sulfane using general procedure **2.1.** Purification via flash chromatography on silica gel (eluent: hexane: EtOAc = 3:7); as a brown solid (0.400 g, 51% Yield); M.p. (°C) 68 – 70; ^{1}H NMR (400 MHz, *chloroform-d*) δ 7.37 – 7.32 (m, 2H), 7.30 – 7.26 (m, 2H), 7.26 – 7.23 (m, 1H), 3.49 – 3.41 (m, 1H), 3.22 – 3.06 (m, 3H), 2.75 (s, 3H); ^{13}C NMR (101 MHz, *chloroform-d*) δ 136.9, 129.3, 128.7, 127.6, 119.9, 51.4, 32.8, 29.6; HRMS (ESI, m/z) m/z calculated for $C_{10}H_{12}N_2NaS$

$(MNa)^+$ 215.0619, found 215.0618; IR film: νmax/cm^{-1} 3028, 2130, 1602, 1454, 1154, 972, 770, 695, 556, 494

***N*-(cyano) methyl 4-nitrophenyl sulfilimine (4t)**

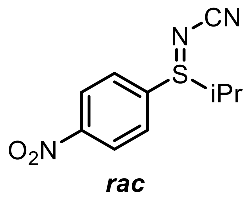

Synthesised from isopropyl 4-nitrophenyl sulfane following general procedure **2.2.** Purification via flash chromatography on silica gel (gradient eluent: hexane: EtOAc = 1:4 to EtOAc); as a red oil (0.860 g, 86% yield); ^{1}H NMR (400 MHz, *chloroform-d*) δ 8.44 – 8.33 (m, 2H), 7.98 – 7.91 (m, 2H), 3.40 (hept, *J* = 6.7 Hz, 1H), 1.35 (d, *J* = 6.7 Hz, 3H), 1.24 (d, *J* = 6.7 Hz, 3H); ^{13}C NMR (101 MHz, *chloroform-d*) δ 150.3, 139.6, 128.4, 128.1, 124.9, 121.5, 55.2, 16.9, 14.6; HRMS (ESI, m/z) m/z calculated for $C_{10}H_{12}N_3NaO_2S$ $(MNa)^+$ 260.0470, found 260.0464; IR film: νmax/cm^{-1} 3027, 2152, 1525, 1345, 1157, 852, 742, 492.

***N*-(cyano) methyl 4-trifluorophenyl sulfilimine (4u)**

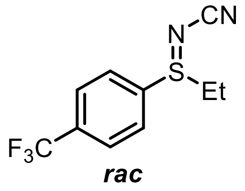

Synthesised from 4-trifluorophenyl methyl sulfane following general procedure **2.1.** Purification via flash chromatography on silica gel (eluent: EtOAc); as a yellow oil (0.660 g, 62% Yield); ^{1}H NMR (400 MHz, *chloroform-d*) δ 7.92 (d, *J* = 8.5 Hz, 2H), 7.87 (d, *J* = 8.6 Hz, 2H), 3.33 – 3.11 (m, 2H), 1.41 (t, *J* = 7.4 Hz, 3H); ^{13}C NMR (101 MHz, *chloroform-d*) δ 138.9, 135.02 (q, *J* = 32.7), 127.4 (q, *J* = 3.7 Hz), 126.9, 123.0 (q, *J* = 273 Hz) 120.7, 47.2, 7.9; ^{19}F NMR (376 MHz, *chloroform-d*) δ -63.06; HRMS (ESI, m/z) m/z calculated for $C_{10}H_{10}F_3N_2NaS$ $(MNa)^+$ 269.0336, found 269.0328; IR film: νmax/cm^{-1} 3043, 2149, 1606, 1403, 1321, 1168, 1012, 840, 761, 595.

***(S)*-*N*-(cyano) methyl phenyl sulfilimine (*(S)*-4a)** [1]

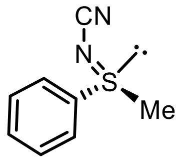

Synthesised from *N*-(cyano) methyl phenyl sulfilimine (49.0 mg, 0.300 mmol) using general procedure **2.4**. Purification via flash chromatography on silica gel (eluent: hexane: EtOAc = 2:3); as a colourless oil (20.0 mg, 35% yield); $[\alpha]_D^{20}$ = –1.59, (c =1.00 in chloroform); ^{1}H NMR (400 MHz, *chloroform-d*) δ 7.82 – 7.78 (m, 2H), 7.66 – 7.58 (m, 3H), 3.02 (s, 3H); ^{13}C NMR (101 MHz, *chloroform-d*) δ 136.3, 133.3, 130.5, 126.1, 120.4, 36.6; HPLC Data: 32% *ee*, determined by HPLC (AS-H, flow rate: 0.8 mL/min, hexane/isopropanol: 65:35): tr (minor) = 27.01, tr (major) = 25.0. See section 4.0 for images depicting the HPLC trace.

***(S)*-*N*-(cyano) methyl 4-methoxyphenyl sulfilimine (*(S)*-4b)** [1]

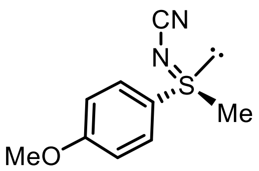

Synthesised from *N*-(cyano) methyl 4-methoxyphenyl sulfilimine (208 mg, 1.07 mmol) using general procedure **2.5.** Purification via flash chromatography on silica gel (gradient eluent: hexane: EtOAc = 1:4 to EtOAc: MeOH = 19:1); as a white solid (53.0 mg, 26% Yield); $[\alpha]_D^{20}$ = –75.1, (c =1.00 in chloroform); ^{1}H NMR (400 MHz, *chloroform-d)* δ 7.78 – 7.69 (m, 2H), 7.11 – 7.02 (m, 2H), 3.86 (s, 3H), 3.00 (s, 3H); ^{13}C NMR (101 MHz, *chloroform-d*) δ 163.7, 128.5, 126.7, 120.6, 115.8, 55.9, 36.2; HRMS (ESI, m/z) m/z calculated for $C_9H_{10}N_2NaOS$ $(MNa)^+$ 217.0407, found 217.0412; HPLC Data: 73% *ee*, determined by HPLC (AD-H, flow rate: 0.8 mL/min, hexane/isopropanol: 80:20) tr (minor) = 19.47 min, tr (major) = 17.80 min. See section 4.0 for images depicting the HPLC trace.

***(S)-N*-(cyano) methyl 3-methoxyphenyl sulfilimine (*(S)*-4c)**

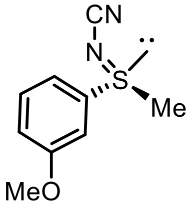

Synthesised from *N*-(cyano) methyl 3-methoxyphenyl sulfilimine (58.0 mg, 0.300 mmol) using general procedure **2.4.** Purification via flash chromatography on silica gel (gradient eluent: hexane: EtOAc = 7:3 to hexane: EtOAc = 1:1); as a yellow oil (21.0 mg, 36% Yield); $[\alpha]_D^{20}$ = –23.34, (c =1.00 in chloroform); ^{1}H NMR (400 MHz, *chloroform-d*) δ 7.47 (t, *J* = 8.0 Hz, 1H), 7.37 – 7.24 (m, 2H), 7.15 – 7.08 (m, 1H), 3.87 (s, 2H), 2.99 (s, 3H); ^{13}C NMR (101 MHz, *chloroform-d*) δ 160.9, 137.4, 131.4, 120.5, 119.4, 117.9, 110.5, 55.9, 36.9; HRMS (ESI, m/z) m/z calculated for $C_9H_{10}N_2NaOS$ $(MNa)^+$ 217.0412, found 217.0399; IR film: νmax/cm^{-1} 3007, 2141 (C≡N stretch), 1593, 1481, 1241, 1148, 1029, 760, 680; ; HPLC Data: 38% *ee*, determined by HPLC (AD-H, flow rate: 1.0 mL/min, hexane/isopropanol: 85:15) tr (minor) = 18.80 min, tr (major) = 17.89 min. See section 4.0 for images depicting the HPLC trace.

***(S)-N*-(cyano) methyl 4-chlorophenyl sulfilimine (*(S)*-4d)** [2]

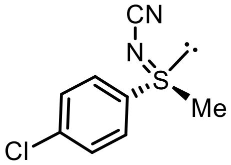

Synthesised from *N*-(cyano) methyl 4-chlorophenyl sulfilimine (99.0 mg, 0.500 mmol) using general procedure **2.4**. Purification via flash column chromatography (gradient eluent: hexane: EtOAc = 2: 3 to EtOAc); as a white solid (38.0 mg, 38% yield); $[\alpha]_D^{20}$ = –71.75 (c =1.5 in chloroform,); ^{1}H NMR (400 MHz, *chloroform-d*) δ 7.78 – 7.72 (m, 2H), 7.62 – 7.57 (m, 2H), 3.02 (s, 3H); ^{13}C NMR (101 MHz, *chloroform-d*) δ 140.0, 134.8, 130.8, 127.5, 36.8; HRMS (ESI, m/z) m/z calculated for $C_8H_7ClN_2NaS$ $(MNa)^+$ 220.9916, found 220.9914; [Note: The CN peak is not present in the ^{13}C NMR spectra recorded. In the cited literature,[2] the CN is very weak and barely visible]; HPLC Data: 59% *ee* determined by HPLC (AD-H Chiralcel Column; flow rate: 1.0 mL/ min, hexane/isopropanol = 90:10, column temperature: 30 °C) tr (minor) = 29.96 min, tr (major) = 25.76 min. See section 4.0 for images depicting the HPLC trace.

(*S*)-*N*-(cyano) methyl 4-trifluorophenyl sulfilimine (*(S)*-4e) [6]

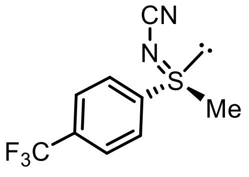

Synthesised from *N*-(cyano) methyl 4-trifluorophenyl sulfilimine (278 mg, 1.20 mmol) following general procedure **2.5.** Purification via flash chromatography on silica gel (gradient eluent: hexane: EtOAc = 7:3 to hexane: EtOAc = 2:3); as a colourless oil (119 mg, 43% Yield); $[\alpha]_D{}^{20}$ = –120.24, (c =1.00 in chloroform); ^{1}H NMR (400 MHz, *chloroform-d*) δ 7.95 (d, *J* = 8.7 Hz, 2H), 7.89 (d, *J* = 8.7 Hz, 2H), 3.07 (s, 3H); ^{13}C NMR (151 MHz, Chloroform-d) δ 140.5, 135.1 (q, *J* = 33.5 Hz), 127.4 (q, *J* = 3.8 Hz), 126.4, 123.0 (q, *J* = 273.4 Hz), 119.6, 36.8; ^{19}F NMR (376 MHz, *chloroform-d*) δ –63.09; HRMS (ESI, m/z) m/z calculated for $C_9H_7F_3N_2NaS$ $(MNa)^+$ 255.0180, found 255.0181; HPLC Data: > 99% *ee* determined by HPLC (AD-H; flow rate: 0.8 mL/ min, hexane/ isopropanol = 80:20) tr (minor) = 12.06, tr (major) = 11.34 min. See section 4.0 for images depicting the HPLC trace.

***(S)*-*N*-(cyano) methyl 4-nitrilephenyl sulfilimine (*(S)*-4f)** [7]

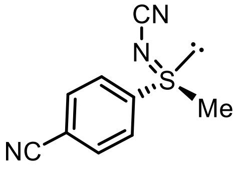

Synthesised from *N*-(cyano) methyl 4-nitrilephenyl sulfilimine (57.0 mg, 0.300 mmol) using general procedure **2.4.** Purification via flash chromatography on silica gel (eluent: hexane: EtOAc = 3:7 to EtOAc: MeOH = 9:1); as a white solid (32.0 mg, 55% yield); $[\alpha]_D{}^{20}$ = – 46.05 (c =1.0 in chloroform); ^{1}H NMR (400 MHz, *chloroform-d*) δ 7.96 – 7.88 (m, 4H), 3.06 (s, 3H); ^{13}C NMR (101 MHz, *chloroform-d*) δ 141.5, 134.0, 126.7, 119.6, 117.0, 117.0, 37.0.; HPLC Data: 34% *ee* determined by HPLC (AS-H, flow rate: 1.0 mL/min, hexane/ isopropanol = 70:30, column temperature = 30 °C) tr (minor) = 51.28 min, tr (major) = 39.54 min. See Section 4.0 for images depicting the HPLC trace.

(*S*)-*N*-(cyano) methyl 4-methylphenyl sulfilimine (*(S)*-4g)

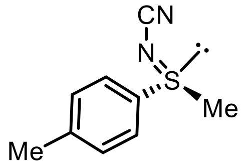

Synthesised from *N*-(cyano) methyl 4-methylphenyl sulfilimine (55.0 mg, 0.300 mmol) using general procedure **2.4.** Purification via flash chromatography on silica gel (gradient eluent: hexane: EtOAc = 3:7 to EtOAc); as a white solid (30.0 mg, 54% Yield); M.p. = 86 – 89 °C; $[\alpha]_D^{20}$ = – 42.94 (c = 1.00 in chloroform); ^{1}H NMR (400 MHz, *chloroform-d*) δ 7.71 – 7.64 (m, 2H), 7.43 – 7.35 (m, 2H), 3.00 (s, 3H), 2.44 (s, 3H); ^{13}C NMR (101 MHz, *chloroform-d*) δ 144.4, 133.0, 131.1, 126.2, 120.6, 36.4, 21.7; HRMS (ESI, m/z) m/z calculated for $C_9H_{10}N_2NaS$ $(MNa)^+$ 201.0462, found 201.0455 IR film: νmax/cm^{-1} 2996, 2136, 1592, 1416,1147, 750, 559, 501; HPLC Data: 46% *ee* determined by HPLC (AD-H, flow rate: 1.00 mL/ min, hexane/ isopropanol = 85:15) tr (minor) = 18.95 min, tr (major) = 16.02 min. See Section 4.0 for images depicting the HPLC trace.

(*S*)-*N*-(cyano) methyl 3-nitrophenyl sulfilimine (*(S)*-4h)

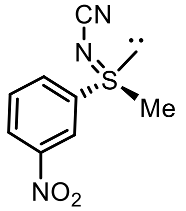

Synthesised from *N*-(cyano) methyl 3-nitrophenyl sulfilimine (63.0 mg, 0.300 mmol) using general procedure **2.4.** Purification via flash chromatography on silica gel (gradient eluent: hexane: EtOAc = 1:1 to EtOAc); as a red oil (25.0 mg, 37% Yield); $[\alpha]_D^{20}$ = – 45.24 (c = 0.6 in chloroform); ^{1}H NMR (400 MHz, *chloroform-d*) δ 8.61 (t, *J* = 2.0 Hz, 1H), 8.50 (ddd, *J* = 8.2, 2.2, 1.0 Hz, 1H), 8.26 – 8.18 (m, 1H), 7.89 (t, *J* = 8.2 Hz, 1H), 3.12 (s, 3H); ^{13}C NMR (101 MHz, *chloroform-d*) δ 149.0, 139.0, 132.0, 131.5, 127.7, 121.3, 119.5, 37.2; HRMS (ESI, m/z) m/z calculated for $C_8H_7N_3NaO_2S$ $(MNa)^+$ 232.0157, found 232.0157; IR film: νmax/cm^{-1} 3069, 2922, 2153, 1525, 1347, 1170, 734; HPLC Data: 44% *ee* determined by HPLC (AS-H, flow rate: 0.8 mL/min, hexane/ isopropanol: 60:40; column temperature: 30 °C) tr (minor) = 43.19 min, tr (major) = 47.35 min. See Section 4.0 for images depicting the HPLC trace.

(*S*)-*N*-(cyano) methyl 3-chlorophenyl sulfilimine (*(S)*-4i)

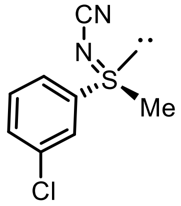

Synthesised from *N*-(cyano) methyl 3-nitrophenyl sulfilimine (59.0 mg, 0.300 mmol) using general procedure **2.4.** Purification via flash chromatography on silica gel (gradient eluent: hexane: EtOAc = 3:7 to EtOAc); as a white solid (26.0 mg, 44% Yield); $[\alpha]_D^{20}$ = –66.55 (c = 1.0 in chloroform); ^{1}H NMR (400 MHz, *chloroform-d*) δ 7.78 – 7.76 (m, 1H), 7.70 – 7.66 (m, 1H), 7.61 – 7.53 (m, 2H), 3.02 (s, 3H); ^{13}C NMR (101 MHz, *chloroform-d*) δ 138.2, 136.7, 133.4, 131.7, 125.9, 124.0, 37.1; HRMS (ESI, m/z) m/z calculated for $C_8H_7{}^{35}ClN_2NaS$ $(MNa)^+$ 220.9916, found 220.9911; HPLC Data: 44% *ee* as determined by HPLC (AS-H, flow rate: 0.8 mL/min, hexane/ isopropanol: 65:35; column temperature: 30 °C) tr (minor) = 26.18 min, tr (major) = 24.02 min. See Section 4.0 for images depicting the HPLC trace.

(*S*)-*N*-cyano methyl 3-chloro-4-(trifluoromethyl)phenyl sulfilimine (*(S)*-4j)

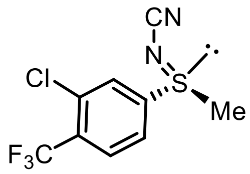

Synthesised from *N*-cyano methyl 3-chloro-4-(trifluoromethyl) phenyl sulfilimine (270 mg, 1.00 mmol) using general procedure **2.5.** Purififcation via flash chromatography on silica gel (gradient eluent: hexane: EtOAc = 7:3 to hexane: EtOAc = 1:4); as a colourless oil (122 mg, 45% Yield); $[\alpha]_D^{20}$ = – 122.51 (c = 1.0 in chloroform); ^{1}H NMR (400 MHz, *chloroform-d*) δ 7.96 – 7.92 (m, 2H), 7.83 – 7.79 (m, 1H), 3.07 (s, 3H); ^{13}C NMR (101 MHz, *chloroform-d*) δ 141.8, 135.1, 132.6 (q, *J* = 32.4 Hz), 129.6 (q, *J* = 4.9 Hz) 128.5, 124.1, 122.0 (q, *J* = 273.8 Hz), 119.5, 37.3; ^{19}F NMR (376 MHz, *chloroform-d*) δ –63.1; HRMS (ESI, m/z) m/z calculated for $C_9H_6{}^{35}ClF_3N_2NaS$ $(MNa)^+$ 288.9790, found 288.9792; IR film: νmax/cm^{-1} IR film: νmax/cm^{-1} 3043, 2149, 1606, 1403, 1321, 1168, 1126, 840, 700, 595; HPLC Data: > 99% *ee* determined by HPLC (AS-H, flow rate: 0.80 mL/min, hexane/isopropanol: 70/30) tr (major) = 15.49. See Section 4.0 for images depicting the HPLC trace.

(*S*)-*N*-(cyano) methyl 2-pridyl sulfilimine (*(S)*-4k) [1]

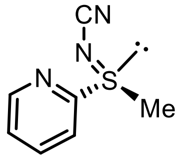

Synthesised from 2-pridyl methyl sulfane (49.5 mg, 0.300 mmol) using general procedure **2.4**. Purification via flash chromatography on silica gel (eluent: EtOAc); as a white solid (20.0 mg, 40% Yield); ^{1}H NMR (400 MHz, *chloroform-d*) δ 8.67 – 8.64 (m, 1H), 8.07 (dt, *J* = 8.0, 1.1 Hz, 1H), 8.00 (td, *J* = 7.5, 1.7 Hz, 1H), 7.50 (ddd, *J* = 7.5, 4.7, 1.1 Hz, 1H), 3.15 (s, 3H); ^{13}C NMR (101 MHz, *chloroform-d*) δ 156.6, 150.6, 139.1, 126.2, 121.8, 120.1, 119.3, 33.9; HRMS (ESI, m/z) m/z calculated for $C_7H_7N_3NaS$ $(MNa)^+$ 188.0258, found 188.0251.

(*S*)-*N*-(cyano) methyl (4-trifluoromethyl-2-pyridyl) sulfilimine (*(S)*-4l)

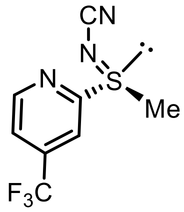

Synthesised from *N*-(cyano) methyl (4-trifluoromethyl-2-pyridyl) sulfilimine (71.0 mg, 0.300 mmol). Purification via flash chromatography (eluent: hexane: EtOAc = 2:3); as a yellow oil (35.0 mg, 50% Yield); $[\alpha]_D^{20}$ = – 27.35 (c = 1.0 in chloroform, measured indirectly after oxidation with *m*-CPBA via general procedure **2.3**), ^{1}H NMR (400 MHz, chloroform-*d*) δ 8.89 (d, *J* = 5.1 Hz, 1H), 8.31 (s, 1H), 7.75 (d, *J* = 5.1 Hz, 1H), 3.22 (s, 3H); ^{13}C NMR (101 MHz, chloroform-*d*) δ 158.8, 151.8, 141.7 (q, *J* = 34.7 Hz), 122.1 (q, *J* = 3.4 Hz), 121.9 (q, *J* = 274 Hz), 119.6, 118.1 (q, *J* = 3.4 Hz), 34.5; HRMS (ESI, m/z) m/z calculated for $C_8H_6F_3N_3NaS$ $(MNa)^+$ 256.0132, found 256.0129; IR film: νmax/cm^{-1} 3075, 2150, 1322, 1172, 1129, 858, 660, 460; HPLC Data: 35% *ee* determined by HPLC (AD-H, flow rate: 1.00 mL/ min, hexane: isopropanol = 85:15) tr (minor) = 15.60 min, tr (major) = 14.08.

(*S*)-*N*-(cyano) (5-trifluoromethyl-2-pyridyl) sulfilimine (*(S)*-4m)

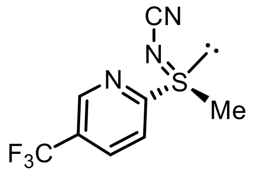

Synthesised *N*-(cyano) methyl (5-trifluoromethyl-2-pyridyl) sulfilimine (60.0 mg, 0.250 mmol) using general procedure **2.4.** Purifciation via flash chromatography (eluent: hexane: EtOAc = 2:3); as a red oil (30.0 mg, 53% Yield); $[\alpha]_D^{21.5}$ = – 32.96 (c = 0.50 in chloroform, measured indirectly after oxidation with *m*-CPBA via general procedure **2.3**); ^{1}H NMR (400 MHz, chloroform-*d*) δ 8.97 – 8.92 (s, 1H), 8.27 (d, *J* = 1.5 Hz, 2H), 3.23 (s, 3H); ^{13}C NMR (101 MHz, *chloroform-d*) δ 160.7, 147.7 (q, *J* = 3.5 Hz), 136.5 (q, *J* = 3.5 Hz), 129.2 (q, *J* = 33.8 Hz), 122.6 (*J* = 273.2 Hz), 122.1, 119.5, 34.1; ^{19}F NMR (376 MHz, *chloroform-d*) δ –62.41; HRMS (ESI, m/z) m/z calculated for $C_8H_6F_3N_3NaS$ $(MNa)^+$ 256.0127, found 256.0129; IR film: νmax/cm^{-1} 3014, 2152, 1713, 1592, 1324, 1101, 1071, 773, 549; HPLC Data: 40% *ee* determined by HPLC (AD-H, flowrate: 0.8 mL/ min, hexane: isopropanol = 85:15) tr (minor) = 20.17 min, tr (major) = 22.56 min. See Section 4.0 for images depicting the HPLC trace.

(*S*)-N-(cyano) ethyl phenyl sulfilimine (*(S)*-4n)

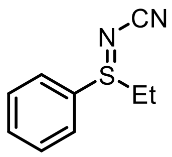

rac

Synthesised from N-(cyano) ethyl phenyl sulfilimine (58.0 mg, 0.300 mmol) using general procedure **2.4.** Purification via flash chromatography on silica gel (gradient eluent: hexane: EtOAc = 2:3 to EtOAc); as a colourless oil (22.0 mg, 48% Yield); $[\alpha]_D^{21.5}$ = – 51.45 (c = 1.00 in chlorform); ^{1}H NMR (400 MHz, *chloroform-d*) δ 7.78 – 7.74 (m, 2H), 7.65 – 7.56 (m, 3H), 3.26 (dq, *J* = 13.1, 7.4 Hz, 1H), 3.13 (dq, *J* = 13.1, 7.4 Hz, 1H), 1.37 (t, *J* = 7.4 Hz, 3H); ^{13}C NMR (101 MHz, *chloroform-d*) δ 134.6, 133.2, 130.3, 126.5, 121.3, 46.7, 8.1; HRMS (ESI, m/z) m/z calculated for $C_9H_{10}N_2NaS$ $(MNa)^+$ 201.0462, found 201.0463.; IR film: νmax/cm^{-1} 3059, 2141, 1711, 1444, 1170, 686, 583; HPLC Data: 28% *ee* determined by HPLC (AD-H, flow rate: 0.8 mL/ min, hexane: isopropanol = 80:20) tr (minor) = 14.90 min, tr (major) = 13.90 min. See Section 4.0 for images of the HPLC trace.

(*S*)-*N*-(cyano) propyl phenyl sulfilimine (*(S)*-4o)

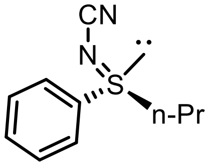

Synthesised from *N*-(cyano) propyl phenyl sulfilimine (58.0 mg, 0.300 mmol) using general procedure **2.1**. Purification via flash chromatography on silica gel (gradient eluent: hexane: EtOAc = 7:8 to EtOAc); as a golden oil (0.590 g, 59% Yield); $[\alpha]_D^{21.5}$ = – 33.38 (c = 1.00 in chloroform); ^{1}H NMR (400 MHz, *chloroform-d*) δ 7.83 – 7.69 (m, 2H), 7.67 – 7.51 (m, 3H), 3.28 (ddd, *J* = 12.9, 7.9, 6.2 Hz, 1H), 2.99 (ddd, *J* = 12.9, 8.4, 7.3 Hz, 1H), 1.91 – 1.73 (m, 2H), 1.09 (t, *J* = 7.4 Hz, 3H); ^{13}C NMR (101 MHz, *chloroform-d*) δ 135.2, 133.2, 130.4, 126.4, 121.2, 54.4, 17.3, 13.0; IR film: νmax/cm^{-1} 2967, 2934, 2141, 1444, 1149, 747, 668, 486; HRMS (ESI, m/z) m/z calculated for $C_{10}H_{12}N_2NaS$ (MNa)$^+$ 217.0412, found 217.0407; HPLC Data: 12% *ee* determined by HPLC (AD-H, flowrate: 1.00 mL/min, hexane/isopropanol = 95:5, column temperature: 30 °C) tr (minor) = 62.51 min, tr (major) = 59.80 min. See Section 4.0 for images depicting the HPLC trace.

(*S*)-*N*-(cyano) iso-propyl phenyl sulfilimine (*(S)*-4p) [6]

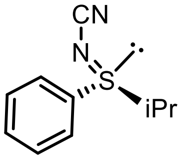

Synthesised from *N*-(cyano) iso-propyl phenyl sulfilimine (58.0 mg, 0.300 mmol) using general procedure **2.4.** Purification via flash chromatography on silica gel (eluent: hexane: EtOAc = 1:1 to hexane: EtOAc = 1:4); as a colourless oil (37.0 mg, 63% Yield); $[\alpha]_D^{20}$ = – 56.93 (c = 1.00 in chloroform); ^{1}H NMR (400 MHz, *chloroform-d*) δ 7.76 – 7.72 (m, 2H), 7.65 – 7.55 (m, 3H), 3.34 (hept, *J* = 6.8 Hz, 1H), 1.35 (d, *J* = 6.8 Hz, 3H), 1.26 (d, *J* = 6.8 Hz, 3H); ^{13}C NMR (101 MHz, *chloroform-d*) δ 133.1, 132.9, 130.1, 127.2, 122.2, 54.3, 16.9, 15.6; HRMS (ESI, m/z) m/z calculated for $C_{10}H_{12}N_2NaS$ (MNa)$^+$ 215.0613, found 215.0613; IR film: νmax/cm^{-1} 3059, 2145, 1633, 1443, 1155, 1081, 747, 483; HPLC Data: 26% *ee* determined by HPLC (AS-H, flowrate: 1.00 mL/ min, hexane:

isopropanol = 70:30) tr (minor) = 39.49 min, tr (major) = 28.21 min. See Section 4.0 for images depicting the HPLC trace.

(*S*)-*N*-(cyano) cyclopropyl phenyl sulfilimine (*(S)*-4q)

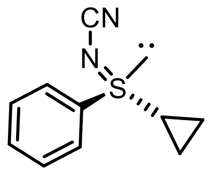

Synthesised from *N*-(cyano) cyclopropyl phenyl sulfilimine (57.0 mg, 0.300 mmol) using general procedure **2.1.** Purification via flash chromatography on silica gel (eluent hexane: EtOAc = 3:7); as a golden oil (26.0 mg, 46% yield); $[\alpha]_D^{20}$ = – 30.82 (c = 1.00 in chloroform); ^{1}H NMR (400 MHz, *chloroform-d*) δ 7.83 – 7.73 (m, 1H), 7.66 – 7.54 (m, 2H), 2.76 – 2.64 (m, 1H), 1.49 – 1.34 (m, 1H), 1.31 – 1.14 (m, 3H); ^{13}C NMR (101 MHz, *chloroform-d*) δ 136.0, 132.9, 130.2, 128.6, 128.5, 127.3, 126.4, 121.3, 29.7, 28.5, 5.6, 4.6; IR film: νmax/cm^{-1} 3025, 2143, 1703, 1444, 1156, 747, 685, 467; HRMS (ESI, m/z) m/z calculated for $C_{10}H_{10}N_2NaS$ $(MNa)^+$ 213.0462, found 213.0461**;** HPLC Data: 34% *ee*, determined by HPLC (AD-H, flowrate: 1.00 mL/ min, hexane: isopropanol = 85:15, column temperature: 30 °C) tr (minor) = 17.71 min, tr (major) = 18.58 min. The *ee* was measured by first oxidising the recovered sulfilimine to the corresponding sulfoximine using general procedure **2.3**. See Section 4.0 for images depicting the HPLC trace.

(*S*)-*N*-(cyano) benzyl phenyl sulfilimine (*(S)*-4r) [8]

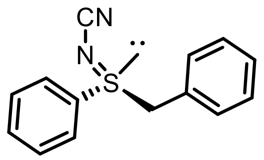

Synthesised from *N*-(cyano) benzyl phenyl sulfilimine (73.0 mg, 0.300 mmol) using general procedure **2.4.** Purification via flash chromatography on silica gel (eluent: hexane: EtOAc = 3: 7); as a white, gum-like solid (40.0 mg, 55% Yield); ^{1}H NMR (400 MHz, *chloroform-d*) δ 7.67 – 7.56 (m, 3H), 7.56 – 7.47 (m, 2H), 7.41 – 7.21 (m, 3H), 7.18 – 7.11 (m, 2H), 4.56 (d, *J* = 12.6 Hz, 1H), 4.27 (d, *J* = 12.6 Hz, 1H); ^{13}C NMR (101 MHz, *chloroform-d*) δ 134.3, 133.3, 130.8, 130.1, 129.8, 129.3, 127.4, 126.9, 120.9, 58.8; HPLC Data: 16% *ee* determined by HPLC (AS-H,

flowrate: 0.8 mL/ min, hexane: isopropanol = 65:35) tr (minor) = 34.12 min, tr (major) = 41.41 min. See Section 4.0 for images depicting the HPLC trace.

(*S*)-*N*-(cyano) phenethyl methyl sulfilimine ((*S*)-4s)

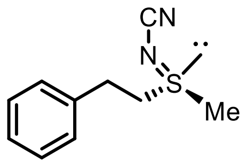

Synthesised from *N*-(cyano) phenethyl methyl sulfilimine (58.0 mg, 0.300 mmol) using general procedure **2.4.** Purification via flash chromatography on silica gel (eluent: hexane: EtOAc = 3:7); as a golden oil (19.0 mg, 31% Yield); $[\alpha]_D^{20}$ = – 29.16 (c = 1.00 in chloroform); M.p. (°C) 68 – 70; ^{1}H NMR (400 MHz, *chloroform-d*) δ 7.37 – 7.32 (m, 2H), 7.30 – 7.26 (m, 2H), 7.26 – 7.23 (m, 1H), 3.49 – 3.41 (m, 1H), 3.22 – 3.06 (m, 3H), 2.75 (s, 3H); ^{13}C NMR (101 MHz, *chloroform-d*) δ 136.9, 129.3, 128.7, 127.6, 119.9, 51.4, 32.8, 29.6; HRMS (ESI, m/z) m/z calculated for $C_{10}H_{12}N_2NaS$ $(MNa)^+$ 215.0619, found 215.0618; IR film: νmax/cm^{-1} 3028, 2130, 1602, 1454, 1154, 972, 770, 695, 556, 494.

(*S*)-*N*-(cyano) isopropyl 4-nitrophenyl sulfilimine ((*S*)-4t)

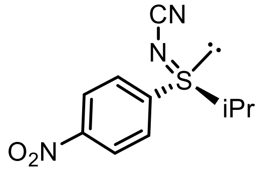

Synthesised from *N*-(cyano) isopropyl 4-nitrophenyl sulfilimine (238 mg, 1.00 mmol) following general procedure **2.5.** Purification via flash chromatography on silica gel (gradient eluent: hexane: EtOAc = 2:3 to EtOAc); as a red oil (111 mg, 47% yield); $[\alpha]_D^{20}$ = – 186.09 (c = 1.00 in chloroform); ^{1}H NMR (400 MHz, *chloroform-d*) δ 8.44 – 8.33 (m, 2H), 7.98 – 7.91 (m, 2H), 3.40 (hept, *J* = 6.7 Hz, 1H), 1.35 (d, *J* = 6.7 Hz, 3H), 1.24 (d, *J* = 6.7 Hz, 3H); ^{13}C NMR (101 MHz, *chloroform-d*) δ 150.3, 139.6, 128.4, 128.1, 124.9, 121.5, 55.2, 16.9, 14.6; HRMS (ESI, m/z) m/z calculated for $C_{10}H_{12}N_3NaO_2S$ $(MNa)^+$ 260.0470, found 260.0464; IR film: νmax/cm^{-1} 3027, 2152, 1525, 1345, 1157, 852, 742, 492; HPLC Data: 84% *ee* determined by HPLC (AD-H, flowrate: 1.00 mL/ min, hexane: isopropanol = 80:20) tr (minor) = 19.61 min, tr (major) = 17.97 min. See Section 4.0 for images depicting the HPLC trace.

(*S*)-*N*-(cyano) ethyl 4-trifluorophenyl sulfilimine (*(S)*-4u)

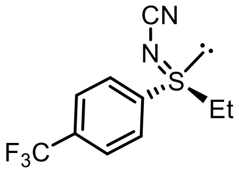

Synthesised from *N*-(cyano) ethyl 4-trifluorophenyl ethyl sulfilimine (246 mg, 1.00 mmol) using general procedure **2.5.** Purification via flash chromatography on silica gel (gradient eluent: hexane: EtOAc = 2:3 to EtOAc); as a colourless oil (109 mg, 47% Yield); $[\alpha]_D^{20}$ = – 194.67 (c = 1.00 in chloroform); ^{1}H NMR (400 MHz, *chloroform-d*) δ 7.92 (d, *J* = 8.6 Hz, 2H), 7.87 (d, *J* = 8.6 Hz, 2H), 3.33 – 3.11 (m, 2H), 1.41 (t, *J* = 7.4 Hz, 3H); ^{13}C NMR (101 MHz, *chloroform-d*) δ 138.9, 135.02 (*J* = 32.7), 127.4 (q, *J* = 3.7 Hz), 126.9, 123.0 (q, *J* = 273 Hz) 120.7, 47.2, 7.9; ^{19}F NMR (376 MHz, *chloroform-d*) δ –63.06; HRMS (ESI, m/z) m/z calculated for $C_{10}H_{10}F_3N_2NaS$ $(MNa)^+$ 269.0336, found 269.0328; IR film: vmax/cm^{-1} 3043, 2149, 1606, 1403, 1321, 1168, 1012, 840, 761, 595**;** HPLC Data: > 99% *ee* determined by HPLC (AS-H, flowrate: 0.80 mL/ min, hexane: isopropanol = 70:30) tr (major) = 14.70 min, tr (minor) = 17.29 min.

***(S)*-*N*-(cyano) methyl phenyl sulfoximine ((*S*)-5a)** [1]

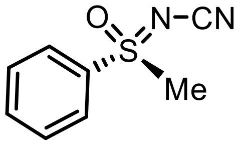

Synthesised from *N*-(cyano) methyl phenyl sulfilimine (49.0 mg, 0.300 mmol) using general procedure **2.4**. Purification via flash chromatography on silica gel (eluent: hexane: EtOAc = 2:3); as a colourless oil (16.0 mg, 22% yield); $[\alpha]_D^{20}$ = + 46.54 (c = 1.00 in chloroform), Lit $[\alpha]_D$ = +196 (c = 0.41 in chloroform); ^{1}H NMR (400 MHz, *chloroform-d*) δ 7.98 – 7.94 (m, 2H), 7.76 (tt, *J* = 7.8, 1.3 Hz, 1H), 7.68 – 7.63 (dd, *J* = 8.5, 7.2 Hz, 2H), 3.32 (s, 3H); ^{13}C NMR (101 MHz, *chloroform-d*) δ 135.9, 135.5, 130.3, 127.8, 127.5, 112.0, 44.7; HPLC Data: 74% *ee*, determined by HPLC (AS-H, flow rate: 0.8 mL/min, hexane/isopropanol: 65:35): tr (minor) = 23.51 min, tr (major) = 26.63 min. See section 4.0 for images depicting the HPLC trace.

To validate the absolute configuration of the major enantiomer of our UPO-promoted synthesis of **(S)-5a**, the same compound was also produced via a published chemical synthesis[8b] and resolution method, summarised below:

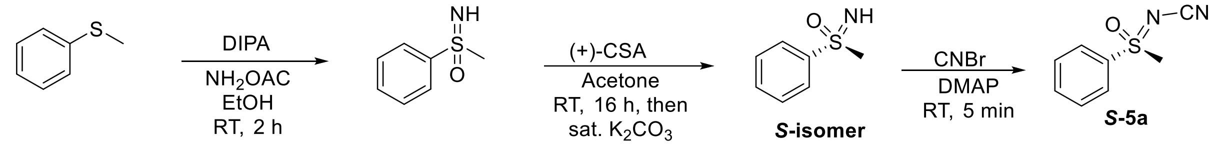

To a round bottom flask containing a magnetic stirring bar was added methyl(phenyl)sulfane (620 mg, 5 mmol, 1.0 equiv.) and NH_2OAc (1.50 g, 20 mmol, 4.0 equiv.) in EtOH (20 mL). To the solution, diisopropylamine (DIPA) (3.20 g, 10 mmol, 2.0 equiv.) was added in one portion. The resulting reaction mixture was stirred vigorously at room temperature 2 h open to air. Upon completion, the reaction mixture was concentrated *in vacuo* to give a residue, which was then purified by column chromayography on silica gel (eluent: ethyl acetate:hexane 1:1), to afford *S*-methyl-*S*-phenylsulfoximine (490 mg, 63% yield) as colourless oil.

S-methyl-*S*-phenylsulfoximine (490 mg, 3.14 mmol, 1.0 equiv.) was dissolved in dry acetone (2 mL). To the solution, (+)-CSA (360 mg, 1.57 mmol, 0.5 equiv.) in 1 mL dry acetone was added. The reaction was stirred at room temperature for further 16 hours. After this time, the precipitate was filtrated and washed by dry acetone (10 mL). The solid was then dissolved in DCM (20 mL) and washed with *sat* K_2CO_3 (50 mL). The organic phase was concentrated *in vacuo* to give (*S*)-*S*-methyl-*S*-phenylsulfoximine (125 mg, 26% yield, $[\alpha]_D^{20} = + 33.13$, (c =0.6 in acetone) as colourless oil.

(*S*)-*S*-methyl-*S*-phenylsulfoximine (60.0 mg, 0.4 mmol, 1.0 equiv.) and DMAP (54 mg, 0.44 mmol, 1.1 equiv.) were added into 2 mL DCM. To the solution, CNBr (85 mg, 0.8 mmol, 2.0 equiv.) was added in one portion. After stirred at room temperature for further 5 min. The reaction was quenched by adding H_2O (10 mL) and the organic phases was dried over anhydrous $MgSO_4$ and concentrated *in vacuo* to give a crude ***S*-5a (**84% ee**)**, which is already

pure enough to do chiral HPLC analysis. HPLC condition: ASH column; 0.8 mL/min flow rate; Hex:IPA 65:35; tr(minor): 22.02, tr (major): 24.85 min.

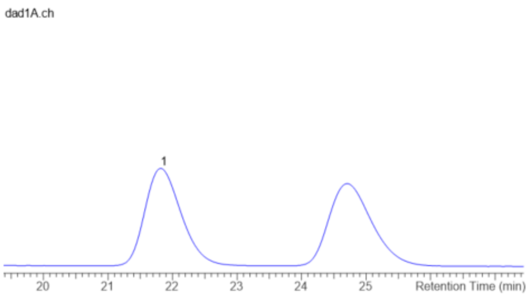

No.	Name	tR	Peak Area (Y units*ms)	Area (%)	Width
1	R	21.82	1981538.375	50.045	1.031
2	S	24.71	1977995.500	49.955	1.223

racemic

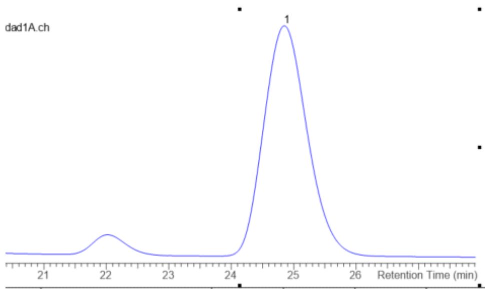

No.	Name	tR	Peak Area (Y units*ms)	Area (%)	Width
1	R	22.02	678982.000	7.836	1.042
2	S	24.85	7986437.000	92.164	1.285

84% *ee*

(S)-N-(cyano) methyl 4-methoxyphenyl sulfoximine ((*S*)-5b) [1]

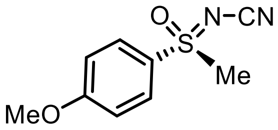

Synthesised from *N*-(cyano) methyl 4-methoxyphenyl sulfilimine (208 mg, 1.07 mmol) using general procedure **2.5.** Purification via flash chromatography on silica gel (gradient eluent: hexane: EtOAc = 1:4 to EtOAc: MeOH = 19:1); as a white solid (93.0 mg, 41% yield); $[\alpha]_D^{20}$ = +174.72 (c = 1.00 in chloroform); ^{1}H NMR (400 MHz, *chloroform-d*) δ 7.94 – 7.85 (m, 2H), 7.12 – 7.05 (m, 2H), 3.90 (s, 3H), 3.30 (s, 3H); ^{13}C NMR (101 MHz, *chloroform-d*) δ 165.2, 130.4, 126.7, 115.6, 112.3, 56.1, 45.3; HPLC Data: 90% *ee* determined by HPLC (AD-H, flow rate: 0.80 mL/min, hexane/isopropanol: 80:20) tr (minor) = 21.95 min, tr (major) = 23.20 min. See section 4.0 for images depicting the HPLC trace.

***(S)-N*-(cyano) methyl 3-methoxyphenyl sulfilimine ((*S*)-5c)** [9]

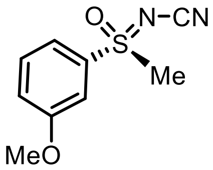

Synthesised from *N*-(cyano) methyl 3-methoxyphenyl sulfilimine (58.0 mg, 0.300 mmol) using general procedure **2.4.** Purification via flash chromatography on silica gel (gradient eluent: hexane: EtOAc = 7:3 to hexane: EtOAc = 1:1); as a colourless oil (18.0 mg, 29% yield); $[\alpha]_D^{20}$ = + 75.14 (c = 1.00 in chloroform); ^{1}H NMR (400 MHz, *chloroform-d*) δ 7.61 – 7.54 (m, 2H), 7.47 – 7.44 (m, 1H), 7.30 – 7.27 (m, 1H), 3.91 (s, 3H), 3.33 (s, 3H); ^{13}C NMR (101 MHz, chloroform-*d*) δ 160.8, 137.3, 131.5, 122.0, 120.0, 112.5, 111.9, 56.1, 44.9; HPLC Data: 90% *ee* determined by HPLC (AD-H, flow-rate: 0.8 mL/min, hexane/isopropanol: 80/20) tr (minor) = 17.20 min, tr (major) 19.24 min. See Section 4.0 for images depicting the HPLC trace.

(*S*)-*N*-(cyano) methyl 4-chlorophenyl sulfoximine ((*S*)-5d) [9]

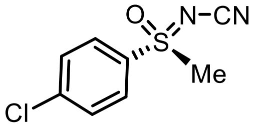

Synthesised from *N*-(cyano) methyl 4-chlorophenyl sulfilimine (99.0 mg, 0.500 mmol) using general procedure **2.4**. Purification via flash column chromatography (gradient eluent: hexane: EtOAc = 2: 3 to EtOAc); as a colourless oil (49.0 mg, 46% yield); $[\alpha]_D^{20}$ = +124.58 (c =1.0 in chloroform); ^{1}H NMR (400 MHz, *chloroform-d*) δ 7.97 – 7.89 (m, 2H), 7.69 – 7.61 (m, 2H), 3.35 (s, 3H); ^{13}C NMR (101 MHz, *chloroform-d*) δ 142.7, 134.5, 130.7, 129.5, 111.6, 44.9; HRMS (ESI, m/z) m/z calculated for $C_8H_7ClN_2NaOS$ $(MNa)^+$ 236.9865, found 236.9871; HPLC Data: 90% *ee* determined by HPLC (AD-H Chiralcel Column; flow rate: 1.0 mL/ min, hexane/isopropanol = 90:10, column temperature: 30 °C) tr (minor) = 32.58 min, tr (major) = 40.14 min. See Section 4.0 for images depicting the HPLC trace.

(*S*)-*N*-(cyano) methyl 4-trifluorophenyl sulfoxime ((*S*)-5e) [9]

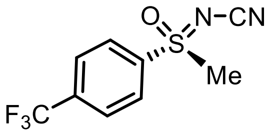

Synthesised from *N*-(cyano) methyl 4-trifluorophenyl sulfilimine (278 mg, 1.20 mmol) following general procedure **2.5**. Purification via flash chromatography on silica gel (gradient eluent: hexane: EtOAc = 7:3 to hexane: EtOAc = 2:3); as a colourless oil (147 mg, 44% yield); $[\alpha]_D^{20}$ = + 123.75, (c =1.00 in chloroform); ^{1}H NMR (400 MHz*, chloroform-d*) δ 8.16 (d, *J* = 8.2 Hz, 2H), 7.96 (d, *J* = 8.2 Hz, 2H), 3.39 (s, 3H; C***H₃***); ^{13}C NMR (101 MHz, *chloroform-d)* δ 139.9, 137.2 (q, *J* = 37.2 Hz), 128.9, 127.59 (q, *J* = 3.6 Hz), 122.8 (q, *J* = 273 Hz) , 111.3, 44.7; ^{19}F NMR (376 MHz, *chloroform-d*) δ −63.30; HRMS (ESI, m/z) m/z calculated for $C_9H_7F_3N_2NaOS$ $(MNa)^+$ 271.0123, found 271.0123; IR film: νmax/cm^{-1} 2924 (C-H stretch), 2197, 1403, 1320, 824; HPLC Data: 94% *ee* as determined by HPLC (AD-H; flow rate: 0.8 mL/ min, hexane/ isopropanol = 80:20) tr (minor) = 11.40 min, tr (major) = 13.12 min. See Section 4.0 for images depicting the HPLC trace.

(*R*)-*N*-(cyano) methyl 4-trifluorophenyl sulfoximine ((*R*)-5e)

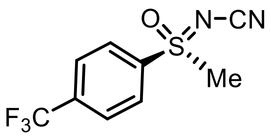

Synthesised from **4e** (119 mg, 0.513 mmol) following general procedure **2.3**. Purification via flash chromatography on silica gel (gradient eluent: hexane: EtOAc = 7:3 to hexane: EtOAc = 2:3); as a colourless oil (108 mg, 87% yield); $[\alpha]_D^{20}$ = –124.85, (c =1.00 in chloroform); All other spectroscopic data were identical to those above.

(*S*)-*N*-(cyano) methyl 4-cyanophenyl sulfoximine ((*S*)-5f) [8]

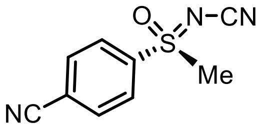

Synthesised from *N*-(cyano) methyl 4-cyanophenyl sulfilimine (57.0 mg, 0.300 mmol) using general procedure **2.4.** Purification via flash chromatography on silica gel (eluent: hexane: EtOAc = 3:7 to EtOAc: MeOH = 9:1); as a colourless oil, (20.0 mg, 33% yield); $[\alpha]_D^{20}$ = +119.1, (c =1.36 in chloroform); ^{1}H NMR (400

MHz, *chloroform-d*) δ 8.19 – 8.10 (m, 2H), 8.05 – 7.96 (m, 2H), 3.40 (s, 3H); ^{13}C NMR (101 MHz, *chloroform-d*) δ 140.5, 134.1, 128.9, 119.4, 116.6, 111.0, 44.6; HRMS (ESI, m/z) m/z calculated for $C_9H_7N_3NaOS$ $(MNa)^+$ 228.0208, found 228.0208; IR film: νmax/cm^{-1} 3093, 2923, 2197, 1399, 1251, 826; HPLC Data: 80% *ee* determined by HPLC (AS-H, flow rate: 1.0 mL/min, hexane/ isopropanol = 70:30, column temperature = 30 °C) tr (minor) = 70.64 min, tr (major) = 66.07 min. See Section 4.0 for pictures depicting the HPLC trace.

(*S*)-*N*-(cyano) methyl 4-methylphenyl sulfilimine ((*S*)-5g) [10]

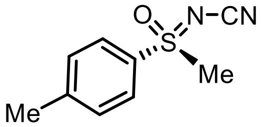

Synthesised from *N*-(cyano) methyl 4-methylphenyl sulfilimine (55.0 mg, 0.300 mmol) using general procedure **2.4.** Purification via flash chromatography on silica gel (gradient eluent: hexane: EtOAc = 3:7 to EtOAc); as a white solid (21.0 mg, 38% Yield); $[\alpha]_D^{20}$ = +115.79 (c = 1.0 in chloroform); ^{1}H NMR (400 MHz, *chloroform-d*) δ 7.90 – 7.83 (m, 2H), 7.51 – 7.43 (m, 2H), 3.31 (s, 3H), 2.49 (s, 3H); ^{13}C NMR (101 MHz, *chloroform-d*) δ 147.1, 133.0, 1301.0, 128.1, 112.1, 45.1, 21.9; HPLC Data: 88% *ee* determined by HPLC (AD-H, flow rate: 1.00 mL/ min, hexane/ isopropanol = 85:15) tr (minor) = 18.91 min, tr (major) = 22.71 min. See Section 4.0 for images depicting the HPLC trace.

(*S*)-*N*-(cyano) methyl 3-nitrophenyl sulfoximine ((*S*)-5h) [11]

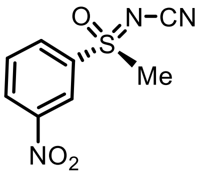

Synthesised from *N*-(cyano) methyl 3-nitrophenyl sulfilimine (63.0 mg, 0.300 mmol) using general procedure **2.4.** Purification via flash chromatography on silica gel (gradient eluent: hexane: EtOAc = 1:1 to EtOAc); as a yellow oil (25.0 mg, 38% yield), $[\alpha]_D^{20}$ = +107.77 (c = 0.8 in chloroform); ^{1}H NMR (400 MHz, *chloroform-d*) δ 8.85 (t, *J* = 1.9 Hz, 1H), 8.65 (ddd, *J* = 8.2, 1.9, 1.0 Hz, 1H), 8.35 (ddd, *J* = 7.9, 1.9, 1.0 Hz, 1H), 7.96 (app. t, *J* = 8.1 Hz, 1H), 3.45 (s, 3H); ^{13}C NMR (101 MHz, *chloroform-d*) δ 149.1, 138.6, 133.5, 132.0, 130.0, 123.7, 110.8, 44.7; HRMS (ESI, m/z)

m/z calculated for $C_8H_7N_3NaO_3S$ $(MNa)^+$ 248.0106, found 248.0099; HPLC Data: 82% *ee* determined by HPLC (AS-H, flow rate: 0.8 mL/min, hexane/ isopropanol: 60:40; column temperature: 30 °C) tr (minor) = 34.45 min, tr (major) = 41.58 min. See Section 4.0 for images depicting the HPLC trace.

(*S*)-*N*-(cyano) methyl 3-chlorophenyl sulfoximine ((*S*)-5i) [12]

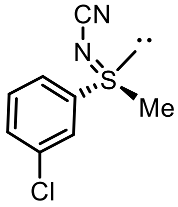

Synthesised from *N*-(cyano) methyl 3-chlorophenyl sulfilimine (59.0 mg, 0.300 mmol) using general procedure **2.4.** Purification via flash chromatography on silica gel (gradient eluent: hexane: EtOAc = 1:1 to EtOAc); as a red solid (23.0 mg, 36% yield), $[\alpha]_D^{20}$ = +103.25 (c = 1.0 in chloroform); ^{1}H NMR (400 MHz, *chloroform-d*) δ 7.98 (t, *J* = 2.0 Hz, 1H), 7.91 – 7.88 (m, 1H), 7.78 – 7.74 (m, 1H), 7.64 (t, *J* = 8.0 Hz, 1H), 3.36 (s, 3H); ^{13}C NMR (101 MHz, chloroform-*d*) δ 138.0, 136.8, 135.8, 131.7, 128.1, 126.2, 111.4, 44.9; HPLC Data: 80% *ee* as determined by HPLC (AS-H, flow rate: 0.8 mL/min, hexane/ isopropanol: 60:40; column temperature: 30 °C) tr (minor) = 16.57 min, tr (major) = 22.03 min. See Section 4.0 for images depicting the HPLC trace.

(*S*)-*N*-cyano methyl 3-chloro-4-(trifluoromethyl)phenyl sulfoximine ((*S*)-5j)

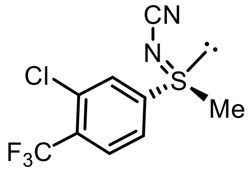

Synthesised from *N*-cyano methyl 3-chloro-4-(trifluoromethyl) phenyl sulfilimine (270 mg, 1.00 mmol) using general procedure **2.5.** Purififcation via flash chromatography on silica gel (gradient eluent: hexane: EtOAc = 7:3 to hexane: EtOAc = 1:4); as a white solid (132 mg, 47% yield); Mp (°C) = 97 – 98; $[\alpha]_D^{20}$ = 106.16 (c = 1.0 in chloroform); ^{1}H NMR (400 MHz, chloroform-*d*) δ 8.16 – 8.14 (m, 1H), 8.05 – 7.99 (m, 2H), 3.41 (s, 3H); ^{13}C NMR (101 MHz, *chloroform-d*) δ 141.04, 135.2 (q, *J* =1.4 Hz), 135.0 (q, *J* =32.4 Hz), 131.0, 129.8 (q, *J* = 5.4 Hz), 126.4, 121.7 (q, *J* = 273.8 Hz), 110.8, 44.7; ^{19}F NMR (376 MHz, *chloroform-d*) δ –63.40; HRMS (ESI, m/z) m/z calculated for $C_9H_6ClF_3N_2NaSO$ $(MNa)^+$ 304.9739, found 304.9737; IR film: νmax/cm^{-1} 3035,

2189, 1588, 1391, 1310, 1249, 1105, 1027, 969, 827, 554, 454; HPLC Data: 98% *ee* determined by HPLC (AS-H, flow rate: 0.80 mL/min, hexane/isopropanol: 70/30) tr (minor) = 14.77 min, tr (major) = 16.49 min. See Section 4.0 for images depicting the HPLC trace.

(*S*)-*N*-(cyano) methyl 2-pyridyl sulfoximine ((*S*)-5k) [1]

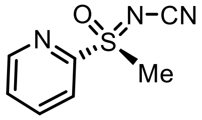

Synthesised from 2-pridyl methyl sulfane (49.5 mg, 0.300 mmol) using general procedure **2.4**. Purification *via* flash chromatography on silica gel (eluent: EtOAc); as a white solid (23.0 mg, 40% Yield); $[\alpha]_D^{20}$ = +17.87 (c = 1.0 in chloroform); ^{1}H NMR (400 MHz, *chloroform-d*) δ 8.79 (ddd, *J* = 4.7, 1.8, 1.0 Hz, 1H), 8.20 (dt, *J* = 7.8, 1.0 Hz, 1H), 8.09 (td, *J* = 7.8, 1.8 Hz, 1H), 7.69 (ddd, *J* = 7.8, 4.7, 1.0 Hz, 1H), 3.51 (s, 3H); ^{13}C NMR (101 MHz, *chloroform-d*) δ 154.7, 150.9, 139.2, 128.9, 122.6, 111.6, 39.8; HRMS (ESI, m/z) m/z calculated for $C_7H_7N_3NaOS$ $(MNa)^+$ 204.0208, found 204.0203; HPLC Data: 64% *ee* determined by HPLC (AD-H, flow rate: 0.8 mL/ min, hexane: isopropanol = 80:20), tr (minor) = 23.24 min, tr (major) = 21.65 min. See Section 4.0 for images depicting the HPLC trace.

(*S*)-*N*-(cyano) methyl (4-trifluoromethyl-2-pyridyl) sulfoximine ((*S*)-5l)

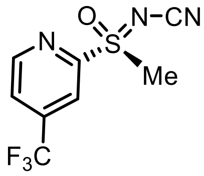

Synthesised from *N*-(cyano) (4-trifluoromethyl-2-pyridyl) sulfilimine (71.0 mg, 0.300 mmol). Purification *via* flash chromatography (eluent: hexane: EtOAc = 2:3); as a white crystaline solid (20 mg; 27% yield); Mp (°C) = 106; $[\alpha]_D^{20}$ = +34.63 (c = 0.79 in chloroform); ^{1}H NMR (400 MHz, chloroform-*d*) δ 9.04 – 9.00 (m, 1H), 8.43 – 8.40 (m, 1H), 7.94 – 7.90 (m, 1H), 3.57 (s, 3H); ^{13}C NMR (101 MHz, chloroform-*d*) δ 156.3, 152.2, 141.8 (q, *J* = 36.4 Hz), 124.8 (q, *J* = 3.4 Hz), 121.7 (q, *J* = 274 Hz) , 118.9 (q, *J* = 3.4 Hz), 110.9, 40.0; ^{19}F NMR (376 MHz, chloroform-*d*) δ –64.49; HRMS (ESI, m/z) m/z calculated for $C_8H_6F_3N_3NaOS$ $(MNa)^+$ 272.0081, found 272.0079: IR film: νmax/cm^{-1} 2924, 2201, 1326 (S=O), 1257, 1144, 989, 751, 662, 489; HPLC Data: 90% *ee* determined by HPLC (AD-H, flowrate: 1.00 mL/ min, hexane/ isopropanol: 85:15, column

temperature: 30 °C) tr (minor) = 14.09 min, tr (major) = 15.67 min. See Section 4.0 depicting the HPLC trace.

(*S*)-*N*-(cyano) methyl (5-trifluoromethyl-2-pyridyl) sulfoximine ((*S*)-5m)

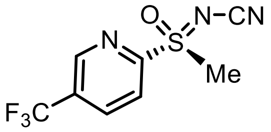

Synthesised *N*-(cyano) (5-trifluoromethyl-2-pyridyl) sulfilimine (60.0 mg, 0.250 mmol) using general procedure **2.4.** Purifciation via flash chromatography (eluent: hexane: EtOAc = 2:3); as a yellow oil (20.0 mg, 30% yield); $[\alpha]_D^{20}$ = + 81.37, (c =1.00 in chloroform); ^{1}H NMR (400 MHz, *chloroform-d*) δ 9.10 – 9.03 (m, 1H), 8.39 – 8.33 (m, 2H), 3.57 (s, 3H); ^{13}C NMR (101 MHz, *chloroform-d*) δ 157.9, 148.0 (q, *J* = 4.0 Hz), 136.6 (q, *J* = 3.6 Hz), 131.5 (q, *J* = 34.5 Hz), 122.2, 122.2 (q, *J* = 273 Hz), 111.0, 39.6; HRMS (ESI, m/z) m/z calculated for $[C_8H_6F_3N_3NaOS]^+$ = 272.0081, found 272.0076; IR film: νmax/cm^{-1} 3015, 2923, 2199, 1594, 1327, 1140, 827; HPLC Data: 90% *ee* determined by HPLC (AD-H, flowrate: 0.8 mL/ min, hexane: isopropanol = 85:15) tr (minor) = 22.34 min, tr (major) = 19.94 min. See Section 4.0 for images depicting the HPLC trace.

(*S*)-*N-(*cyano) ethyl phenyl sulfoximine ((*S*)-5n) [12]

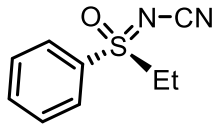

Synthesised from N-(cyano) ethyl phenyl sulfilimine (58.0 mg, 0.300 mmol) using general procedure **2.4.** Purification *via* flash chromatography on silica gel (gradient eluent: hexane: EtOAc = 2:3 to EtOAc); as a colourless oil (22.0 mg, 48% yield); $[\alpha]_D^{21.5}$ = + 58.52 (c = 1.00 in chloroform); ^{1}H NMR (400 MHz, *chloroform-d*) δ 8.00 – 7.91 (m, 2H), 7.79 (tt, 1H), 7.74 – 7.64 (m, 2H), 3.45 (dq, *J* = 14.7, 7.4 Hz, 1H), 3.38 (dq, *J* = 14.7, 7.4 Hz, 1H), 1.34 (t, *J* = 7.4 Hz, 3H); ^{13}C NMR (101 MHz, *chloroform-d*) δ 135.5, 134.1, 130.3, 128.8, 112.2, 51.6, 7.2; HRMS (ESI, m/z) m/z calculated for $C_9H_{10}N_2NaOS$ $(MNa)^+$ 217.0412, found 217.0407; HPLC Data: 50% *ee* determined by HPLC (AD-H, flow rate: 0.8 mL/ min, hexane: isopropanol = 80:20) tr (minor) = 17.28 min, tr (major) = 18.70 min. See Section 4.0 for images depicting the HPLC trace.

(*S*)-*N*-(cyano) n-propyl phenyl sulfoximine ((*S*)-5o) [12]

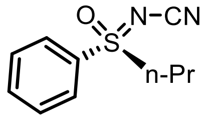

Synthesised from *N*-(cyano) propyl phenyl sulfilimine (58.0 mg, 0.300 mmol) using general procedure **2.1**. Purification *via* flash chromatography on silica gel (gradient eluent: hexane: EtOAc = 7:8 to EtOAc); as a colourless oil (17.0 mg, 27% yield); $[\alpha]_D^{21.5}$ = + 55.75 (c = 1.00 in chloroform); ^{1}H NMR (400 MHz, *chloroform-d*) δ 7.98 – 7.91 (m, 2H), 7.78 (tt, *J* = 7.2, 1.4 Hz, 1H), 7.72 – 7.63 (m, 2H), 3.44 – 3.36 (m, 1H), 3.34 – 3.26 (m, 1H), 1.92 – 1.66 (m, 2H), 1.02 (t, *J* = 7.4 Hz, 3H); ^{13}C NMR (101 MHz, *chloroform-d*) δ 135.5, 134.8, 130.3, 128.6, 112.2, 58.4, 16.3, 12.6; HRMS (ESI, m/z) m/z calculated for $C_{10}H_{12}N_2NaOS$ $(MNa)^+$ 231.0568, found 231.0568; IR film: νmax/cm^{-1} 2971, 2191, 1447, 1240, 1183, 1091, 824, 750, 727, 541, 473; HPLC Data: 42% *ee* determined by HPLC (AD-H, flowrate: 1.00 mL/ min, hexane: isopropanol = 90:10) tr (minor) = 34.11 min, tr (major) = 35.85 min. See Section 4.0 for images depicting the HPLC trace.

(*S*)-*N*-(cyano) iso-propyl phenyl sulfoximine ((*S*)-5p)

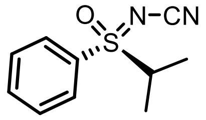

Synthesised from *N*-(cyano) iso-propyl phenyl sulfilimine (58.0 mg, 0.300 mmol) using general procedure **2.4.** Purification *via* flash chromatography on silica gel (eluent: hexane: EtOAc = 1:1 to hexane: EtOAc = 1:4); as a golden oil (22 mg, 35% Yield); $[\alpha]_D^{20}$ = +71.05 (c = 1.00 in chloroform); ^{1}H NMR (400 MHz, *chloroform-d*) δ 7.94 – 7.87 (m, 2H), 7.82 – 7.74 (m, 1H), 7.71 – 7.63 (m, 2H), 3.51 (hept, *J* = 6.8 Hz, 1H), 1.43 (d, *J* = 6.9 Hz, 3H), 1.32 (d, *J* = 6.9 Hz, 3H); ^{13}C NMR (101 MHz, *chloroform-d*) δ 135.4, 132.7, 130.1, 129.6, 112.6, 57.8, 15.8, 15.3; HRMS (ESI, m/z) m/z calculated for $C_{10}H_{12}N_2NaOS$ $(MNa)^+$ 231.0568, found, 231.0558; IR film: νmax/cm^{-1} 2985, 2191, 1448, 1446, 1234, 1189, 1087, 826, 721, 687, 554; HPLC Data: 76% *ee* (AS-H, flowrate: 1.00 mL/ min, hexane: isopropanol = 70:30) tr (minor) = 19.65 min, tr (major) = 21.38 min. See Section 4.0 for images depicting the HPLC trace.

(*S*)-*N*-(cyano) cyclopropyl phenyl sulfoximine ((*S*)-5q) [12]

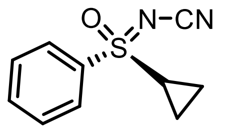

Synthesised from *N*-(cyano) cyclopropyl phenyl sulfilimine (57.0 mg, 0.300 mmol) using general procedure **2.1.** Purification *via* flash chromatography on silica gel (eluent hexane: EtOAc = 3:7); as a golden oil (13 mg, 21% yield); $[\alpha]_D^{21.1}$ = +38.23 (c = 1.00 in chloroform); ^{1}H NMR (400 MHz, *chloroform-d*) δ 7.98 – 7.93 (m, 2H), 7.77 (tt, *J* = 7.8, 1.4 Hz, 1H), 7.69 – 7.63 (m, 2H), 2.73 – 2.65 (m, 1H), 1.74 – 1.67 (m, 1H), 1.43 – 1.28 (m, 3H), 1.16 – 1.08 (m, 1H); ^{13}C NMR (101 MHz, *chloroform-d*) δ 136.5, 135.2, 130.2, 128.1, 112.2, 33.7, 7.2, 6.1; HPLC Data: 74% *ee* determined by HPLC (AD-H, flowrate: 1.00 mL/ min, hexane: isopropanol = 85:15, column temperature: 30 °C) tr (minor) = 17.3 min, tr (major) = 16.59 min. See Section 4.0 for images depicting the HPLC trace.

(*S*)-*N*-(cyano) benzyl phenyl sulfoximine ((*S*)-5r) [8]

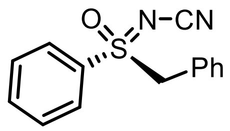

Synthesised from *N*-(cyano) benzyl phenyl sulfilimine (73.0 mg, 0.300 mmol) using general procedure **2.4.** Purification via flash chromatography on silica gel (eluent: hexane: EtOAc = 3: 7); as a white solid (21 mg, 27% yield); ^{1}H NMR (400 MHz, *chloroform-d*) δ 7.75 – 7.69 (m, 1H), 7.65 – 7.61 (m, 2H), 7.56 – 7.51 (m, 2H), 7.40 – 7.35 (m, 1H), 7.31 – 7.27 (m, 2H), 7.06 – 7.03 (m, 2H), 4.62 (s, 2H); ^{13}C NMR (101 MHz, *chloroform-d*) δ 135.5, 133.2, 131.4, 130.1, 129.8, 129.3, 129.1, 125.6, 112.3, 63.5; HPLC Data: 56% *ee* determined by HPLC (AS-H, flowrate: 0.8 mL/ min, hexane: isopropanol = 65:35) tr (minor) = 29.58 min, tr (major) = 25.28 min. See Section 4.0 for images depicting the HPLC trace.

(*S*)-*N*-(cyano) phenethyl methyl sulfoximine ((*S*)-5s)

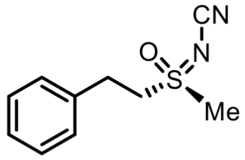

Synthesised from *N*-(cyano) phenethyl methyl sulfilimine (58.0 mg, 0.300 mmol) using general procedure **2.4**. Purification via flash chromatography on silica gel (eluent: hexane: EtOAc = 3:7); as a white

solid (21 mg, 36% yield); $[\alpha]_D^{20}$ = +9.70 (c = 1.00 in chloroform); ^{1}H NMR (400 MHz, *chloroform-d*) δ 7.39 – 7.34 (m, 2H), 7.33 – 7.26 (m, 2H), 7.26 – 7.24 (m, 1H), 3.76 – 3.67 (m, 1H), 3.65 – 3.56 (m, 1H), 3.28 – 3.22 (m, 2H), 3.04 (s, 3H); ^{13}C NMR (101 MHz, *chloroform-d*) δ 135.8, 129.4, 128.7, 127.9, 112.1, 56.5, 41.0, 28.8; $C_{10}H_{12}N_2NaOS$ $(MNa)^+$ 231.0568, found, 231.0564; IR film: v_{max}/cm^{-1} 2923, 2192, 1455, 1245, 1131, 820, 701; HPLC Data: 60% *ee* determined by HPLC (AS-H, flowrate: 1.0 mL/min, hexane: isopropanol = 70:30) tr (minor) = 29.83 min, tr (major) = 25.02 min. See Section 4.0 for images depicting the HPLC trace.

(*S*)-*N*-(cyano) isopropyl 4-nitropheneyl sulfoximine ((*S*)-5t)

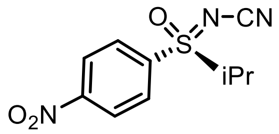

Synthesised from *N*-(cyano) isopropyl 4-nitropheneyl sulfilimine (238 mg, 1.00 mmol) following general procedure **2.5**. Purification via flash chromatography on silica gel (gradient eluent: hexane: EtOAc = 2:3 to EtOAc); as a white solid (111 mg, 44% yield); M.p. 111 – 112 °C; $[\alpha]_D^{20}$ = +178.19 (c = 1.00 in chloroform); ^{1}H NMR (400 MHz, *chloroform-d*) δ 8.54 – 8.50 (m, 2H), 8.17 – 8.13 (m, 2H), 3.59 (hept, *J* = 6.9 Hz, 1H), 1.49 (d, *J* = 6.9 Hz, 3H), 1.38 (d, *J* = 6.9 Hz, 3H); ^{13}C NMR (101 MHz, *chloroform-d*) δ 163.1, 151.9, 139.0, 131.2, 125.2, 111.5, 58.1, 15.8, 15.2; HRMS (ESI, m/z) m/z calculated for $C_{10}H_{12}N_3NaO_3S$ $(MNa)^+$ 276.0413, found 276.0419; IR film: vmax/cm^{-1} 3104, 2987, 2198, 1534, 1353, 1242, 827, 734, 493; HPLC Data: 90% *ee* determined by HPLC (AD-H, flowrate: 1.00 mL/ min, hexane: isopropanol = 80:20) tr (minor) = 24.12 min, tr (major) = 26.89 min. See Section 4.0 for images depicting the HPLC trace.

(*S*)-*N*-(cyano) ethyl 4-trifluorophenyl sulfoximine ((*S*)-5u)

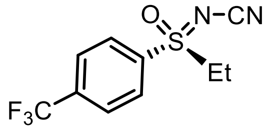

Synthesised from *N*-(cyano) ethyl 4-trifluorophenyl sulfilimine (246 mg, 1.0 mmol) using general procedure **2.5.** Purification *via* flash chromatography on silica gel (gradient eluent: hexane: EtOAc = 2:3 to EtOAc); as a colourless oil (120 mg, 46% yield); $[\alpha]_D^{20}$ = +122.51 (c = 1.00 in chloroform); ^{1}H NMR (400 MHz, *chloroform-d*) δ 8.11 (d, *J* = 8.4 Hz, 2H), 7.95 (d, *J* = 8.4 Hz, 2H), 3.58 – 3.36 (m, 2H), 1.39

(t, J = 7.4 Hz, 3H); ^{13}C NMR (101 MHz, *chloroform-d*) δ 138.0, 137.1 (q, J = 33.8), 129.6, 127.5 (q, J = 3.4 Hz), 122.8 (q, J = 273.1 Hz), 111.5, 51.6, 7.1; ^{19}F NMR (376 MHz, *chloroform-d*) δ –63.28; HRMS (ESI, m/z) m/z calculated for $C_{10}H_{10}F_3N_2NaSO$ $(MNa)^+$ 285.0285, found 285.0281; IR film: νmax/cm^{-1} 3051, 2196, 1404, 1321, 1174, 1091, 831, 719, 540**;** HPLC Data: 96% *ee* determined by HPLC (AS-H, flowrate: 0.80 mL/ min, hexane: isopropanol = 70:30) tr (minor) = 13.79 tr (major) = 15.69 min. See Section 4.0 for images depicting the HPLC trace

Synthesis of *S*-methyl-S-[4-(trifluoromethyl)phenyl]-sulfoxime ((*S*)-9e) [13]

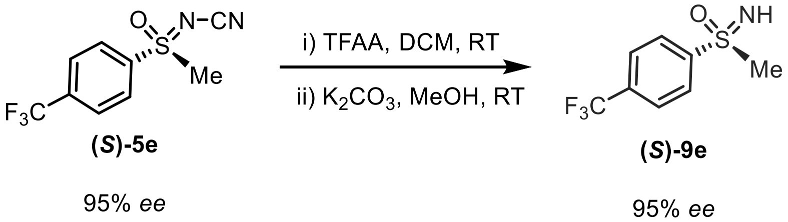

To a stirring solution of **(*S*)-5e** (144 mg, 0.580 mmol) in DCM (8.00 mL) was added TFAA (0.250 mL, 1.74 mmol) at 0 °C before the reaction temperature was raised to RT and stirred for 3 h. A further aliquot of TFAA (0.250 mL, 1.74 mmol) was added and the reaction stirred for 16 h. The solvent was removed *in vacuo* and the crude material dissolved in dry MeOH (10.0 mL) and stirred with solid K_2CO_3 (350 mg, 2.5 mmol) for 4 h, upon which, the solvent was removed *in vacuo* and the resultant crude dissolved in H_2O (10 mL). The reaction was extracted with EtOAc (3 x 30 mL), and the combined organic phase washed with saturated brine (40 mL), dried over $MgSO_4$, filtered, and the solvent removed *in vacuo* to afford a white solid. Purification via flash column chromatography on silica gel (eluent: hexane: EtOAc = 1:4) afforded (*S*)-**9e** as white crystals (100 mg, 83% yield). $[\alpha]_D^{20.9}$ = +16.62 (c = 1.00 in chloroform), Lit $[\alpha]_D^{25}$ = +16.3 (c = 1.1 in chloroform); ^{1}H NMR (400 MHz, *chloroform-d*) δ 8.14 (d, J = 7.6 Hz, 2H), 7.81 (d, J = 7.6 Hz, 2H), 3.11 (d, J = 1.5 Hz, 3H), 2.71 (br s, 1H, N-

H); ^{13}C NMR (101 MHz, *chloroform-d)* δ 147.3, 134.8 (q, *J* = 33.0 Hz), 128.5, 126.5 (q, *J* = 3.7 Hz), 123.3 (q, *J* = 273 Hz), 46.1; ^{19}F NMR (376 MHz, *chloroform-d*) δ –62.99.

(*E*)-*N*-benzylidene-*S*-isopropylthiohydroxylamine (6a-*i*Pr)

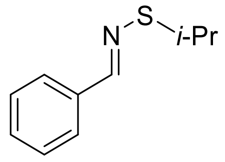

Synthesised from benzylamine (0.660 mL, 6.00 mmol) using general procedure **2.1**. Purified by flash column chromatography on silica gel (eluent: hexane : DCM = 5:1); yellow oil (270 mg, 24% yield); ^{1}H NMR (400 MHz, *Chloroform-d*) δ 8.47 (s, 1H), 7.68 – 7.57 (m, 2H), 7.42 – 7.32 (m, 3H), 3.56 (hept, *J* = 6.8 Hz, 1H), 1.43 (d, *J* = 6.8 Hz, 6H); ^{13}C NMR (101 MHz, *Chloroform-d*) δ 156.1, 136.8, 129.8, 128.7, 127.0, 40.6, 21.7; HRMS (ESI) calcd. for $C_{10}H_{14}NS$ (MH^+): 180.0847, found 180.0841; IR (film): νmax/cm^{-1} 2960, 1447, 1239, 749, 689.

(*E*)-*N*-(4-fluorobenzylidene)-*S*-isopropylthiohydroxylamine (6b-*i*Pr)

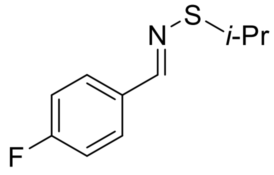

Synthesised from (4-fluorophenyl)methanamine (752 mg, 6.00 mmol) using general procedure **2.1**. Purified by flash column chromatography on silica gel (eluent: hexane : DCM = 5:1); yellow oil (70.0 mg, 7% yield); ^{1}H NMR (400 MHz, *Chloroform-d*) δ 8.42 (s, 1H), 7.69 – 7.54 (m, 2H), 7.06 (t, *J* = 8.7 Hz, 2H), 3.54 (pd, *J* = 6.8, 0.9 Hz, 1H), 1.42 (d, *J* = 6.8 Hz, 6H); ^{13}C NMR (101 MHz, *Chloroform-d*) δ 164.9, 154.5, 128.7 (d, *J* = 9.1 Hz), 115.6 (d, *J* = 22.2 Hz), 40.6, 21.6; HRMS (ESI) calcd. for $C_{10}H_{13}FNS$ (MH^+): 198.0753, found 198.0747; IR (film): νmax/cm^{-1} 2961, 1506, 1227, 830.

(*E*)-*N*-(4-chlorobenzylidene)-*S*-isopropylthiohydroxylamine (6c-*i*Pr)

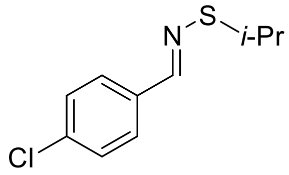

Synthesised from (4-chlorophenyl)methanamine (0.730 mL, 6.00 mmol) using general procedure **2.1**. Purified by flash column chromatography on silica gel (eluent: hexane : DCM = 5:1); yellow oil (150 mg, 15% yield); ^{1}H NMR (400 MHz, *Chloroform-d*) δ 8.41 (s, 1H), 7.54 (d, *J* = 8.4 Hz,

2H), 7.34 (d, J = 8.4 Hz, 2H), 3.54 (dt, J = 13.5, 6.9 Hz, 1H), 1.42 (d, J = 6.9 Hz, 6H). ^{13}C NMR (101 MHz, *Chloroform-d*) δ 154.4, 135.6, 135.2, 128.8, 128.0, 40.7, 21.5; HRMS (ESI) calcd. for $C_{10}H_{13}{}^{35}ClNS$ (MH^+): 214.0457, found 214.0452; IR (film): νmax/cm^{-1} 2961, 1488, 1086, 818.

(*E*)-*N*-(4-bromobenzylidene)-*S*-isopropylthiohydroxylamine (6d-*i*Pr)

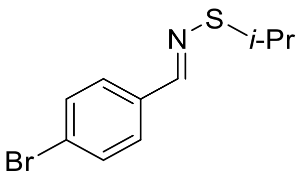

Synthesised from (4-bromophenyl)methanamine (1.10 g, 6.00 mmol) using general procedure **2.1**. Purified by flash column chromatography on silica gel (eluent: hexane : DCM = 5:1); yellow oil (73.0 mg, 6% yield); ^{1}H NMR (400 MHz, *Chloroform-d*) δ 8.39 (s, 1H), 7.58 – 7.39 (m, 4H), 3.54 (hept, J = 6.8 Hz, 1H), 1.42 (d, J = 6.8 Hz, 6H); ^{13}C NMR (101 MHz, *Chloroform-d*) δ 154.6, 135.7, 131.9, 128.3, 124.1, 40.8, 21.6; HRMS (ESI) calcd. for $C_{10}H_{13}{}^{79}BrNS$ (MH^+): 259.9926, found 259.9932; IR (film): νmax/cm^{-1} 2961, 1588, 1485, 1239, 1068, 1008, 816.

(*E*)-*S*-isopropyl-*N*-(4-(trifluoromethyl)benzylidene)thiohydroxylamine (6e-*i*Pr)

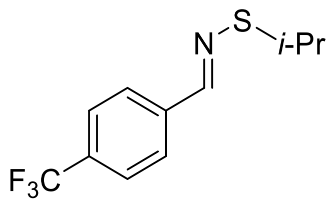

Synthesised from (4-(trifluoromethyl)phenyl)methanamine (0.860 mL, 6.00 mmol) using general procedure **2.1**. Purified by flash column chromatography on silica gel (eluent: hexane : DCM = 5:1); yellow oil (150 mg, 12% yield); ^{1}H NMR (400 MHz, *Chloroform-d*) δ 8.47 (s, 1H), 7.79 – 7.54 (m, 4H), 3.61 – 3.47 (m, 1H), 1.42 (d, J = 7.0 Hz, 6H); ^{13}C NMR (101 MHz, *Chloroform-d*) δ 154.1, 139.6, 131.2 (q, J = 40.4 Hz), 127.0, 125.6 (q, J = 3.8 Hz), 125.5 (q, J = 278.7Hz), 40.9, 21.6; HRMS (ESI) calcd. for $C_{11}H_{13}F_3NS$ (MH^+): 248.0721, found 248.0725; IR (film): νmax/cm^{-1} 2965, 1616, 1320, 1163, 1122, 1064, 832.

(*E*)-*S*-isopropyl-*N*-(4-methoxybenzylidene)thiohydroxylamine (6f-*i*Pr)

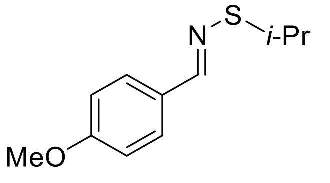

Synthesised from (4-methoxyphenyl)methanamine (0.860 mL, 6.00 mmol) using general procedure **2.1**. Purified by flash column chromatography on silica gel (eluent: hexane : DCM = 5:1); yellow oil (120 mg, 14% yield); ^{1}H NMR (400 MHz, *Chloroform-d*) δ 8.40 (s, 1H), 7.56 (d, *J* = 8.8 Hz, 2H), 6.90 (d, *J* = 8.8 Hz, 2H), 3.83 (s, 3H), 3.54 (hept, *J* = 6.8 Hz, 1H), 1.41 (d, *J* = 6.8 Hz, 6H); ^{13}C NMR (101 MHz, *Chloroform-d*) δ 161.0, 155.8, 130.1, 128.5, 114.1, 55.4, 40.5, 21.7. HRMS (ESI) calcd. for $C_{11}H_{16}ONS$ (MH^+): 210.0953, found 210.0950; IR (film): νmax/cm^{-1} 2960, 1606, 1509, 1246, 1165, 1031, 829.

(*E*)-S-isopropyl-*N*-(3-methoxybenzylidene)thiohydroxylamine (6g-*i*Pr)

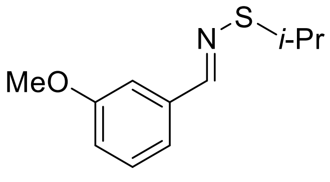

Synthesised from (3-methoxyphenyl)methanamine (0.860 mL, 6.00 mmol) using general procedure **2.1**. Purified by flash column chromatography on silica gel (eluent: hexane : DCM = 5:1); yellow oil (163 mg, 15% yield); ^{1}H NMR (400 MHz, *Chloroform-d*) δ 8.43 (s, 1H), 7.29 (t, *J* = 7.9 Hz, 1H), 7.21 (dd, *J* = 2.7, 1.3 Hz, 1H), 7.15 (dt, *J* = 7.9, 1.3 Hz, 1H), 6.91 (ddd, *J* = 7.9, 2.7, 1.3 Hz, 1H), 3.84 (s, 3H), 3.56 (hept, *J* = 6.8 Hz, 1H), 1.42 (d, *J* = 6.8 Hz, 6H); ^{13}C NMR (101 MHz, *Chloroform-d*) δ 159.8, 155.8, 138.1, 129.5, 120.0, 116.1, 110.9, 55.3, 40.5, 21.6; HRMS (ESI) calcd. for $C_{11}H_{16}ONS$ (MH^+): 210.0953, found 210.0937; IR (film): νmax/cm^{-1} 2960, 1598, 1463, 1262, 1151, 1044, 779, 686.

(*E*)-*S*-isopropyl-*N*-(naphthalen-1-ylmethylene)thiohydroxylamine (6h-*i*Pr)

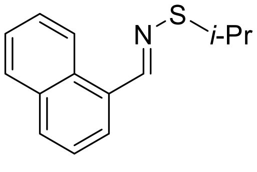

Synthesised from naphthalen-1-ylmethanamine (943 mg, 6.00 mmol) using general procedure **2.1**. Purified by flash column chromatography on silica gel (eluent: hexane : DCM = 5:1); yellow oil (29.0 mg, 3% yield); ^{1}H NMR (400 MHz, *Chloroform-d*) δ 9.14 (s, 1H), 8.82 (d, *J* = 8.2 Hz, 1H), 7.87 (dd, *J*

= 8.2, 4.3 Hz, 2H), 7.79 (d, *J* = 6.5 Hz, 1H), 7.59 – 7.46 (m, 3H), 3.65 (hept, *J* = 6.5 Hz, 1H), 1.49 (d, *J* = 6.5 Hz, 6H); ^{13}C NMR (101 MHz, *Chloroform-d*) δ 156.1, 134.0, 132.2, 130.6, 130.5, 128.7, 127.8, 127.2, 126.1, 125.4, 124.4, 40.8, 21.9; HRMS (ESI) calcd. for $C_{14}H_{16}NS$ (MH$^+$): 230.1003, found 230.1002; IR (film): νmax/cm^{-1} 2958, 1567, 1432, 1128, 854.

(*E*)-*S*-isopropyl-*N*-(1-phenylethylidene)thiohydroxylamine (6i-*i*Pr)

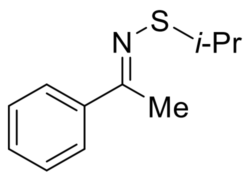

Synthesised from 1-phenylethan-1-amine (0.770 mL, 6.00 mmol) using general procedure **2.1**. Purified by flash column chromatography on silica gel (eluent: hexane : DCM = 5:1); yellow oil (240 mg, 31% yield); ^{1}H NMR (400 MHz, *Chloroform-d*) δ 7.76 (dd, *J* = 8.0, 1.7 Hz, 2H), 7.45 – 7.32 (m, 3H), 3.54 (hept, *J* = 6.8 Hz, 1H), 2.37 (s, 3H), 1.48 (d, *J* = 6.8 Hz, 6H).^{13}C NMR (101 MHz, *Chloroform-d*) δ 159.8, 139.8, 128.9, 128.3, 125.9, 41.0, 21.8, 19.5; HRMS (ESI) calcd. for $C_{11}H_{16}NS$ (MH$^+$): 194.1003, found 194.0998; IR (film): νmax/cm^{-1} 2960, 1444, 1363, 1238, 757, 689.

***S*-isopropyl-*N*-(1-(naphthalen-1-yl)ethylidene)thiohydroxylamine (6j-*i*Pr)**

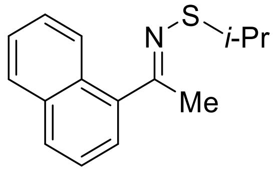

Synthesised from 1-(naphthalen-1-yl)ethan-1-amine (1.02 g, 6.00 mmol) using general procedure **2.1**. Purified by flash column chromatography on silica gel (eluent: hexane : DCM = 5:1); yellow oil (320 mg, 65% yield, *E/Z* 5:1); ^{1}H NMR (400 MHz, *Chloroform-d*) *E* isomer: δ 8.22 – 8.17 (m, 1H), 7.89 – 7.82 (m, 2H), 7.52 – 7.44 (m, 4H), 3.58 (heptd, *J* = 6.8, 0.9 Hz, 1H), 2.50 (d, *J* = 0.9 Hz, 3H), 1.46 (dd, *J* = 6.8, 0.9 Hz, 6H); *Z* isomer: δ 7.93 – 7.89 (m, 1H), 7.70 – 7.64 (m, 1H), 7.56 – 7.52 (m, 4H), 7.28 (dt, *J* = 6.8, 1.0 Hz, 1H), 3.43 (heptd, *J* = 6.8, 1.0 Hz, 1H), 2.48 (d, *J* = 0.8 Hz, 3H), 1.32 – 1.30 (m, 6H); ^{13}C NMR (101 MHz, *Chloroform-d*) *E* isomer: δ 163.3, 140.0, 134.1, 130.5, 128.9, 128.5, 126.4, 126.0, 125.9, 125.2, 125.2, 41.0, 31.7, 24.5, 22.0; *Z* isomer: δ 164.7, 139.1, 133.8, 130.5, 128.8, 128.7, 127.6, 126.7, 125.7, 124.9, 122.9,

40.6, 29.6, 22.8, 21.6; HRMS (ESI) calcd. for $C_{15}H_{18}NS$ (MH^+): 244.1154, found 244.1152; IR (film): νmax/cm^{-1} 2960, 1508, 1237, 794, 769.

(*E*)-*N*-(3,4-dihydronaphthalen-1(2H)-ylidene)-*S*-isopropylthiohydroxylamine (6k-*i*Pr)

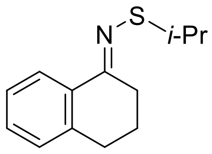

Synthesised from 1,2,3,4,4a,8a-hexahydronaphthalen-1-amine (0.900 mL, 6.00 mmol) using general procedure **2.1**. Purified by flash column chromatography on silica gel (eluent: hexane : DCM = 5:1); red oil (135 mg, 15% yield); ^{1}H NMR (400 MHz, *Chloroform-d*) δ 8.13 – 8.00 (m, 1H), 7.24 – 7.19 (m, 2H), 7.14 – 7.09 (m, 1H), 3.49 (heptd, *J* = 6.8, 1.8 Hz, 1H), 2.78 (t, *J* = 6.8 Hz, 2H), 2.59 (td, *J* = 6.8, 1.8 Hz, 2H), 2.01 – 1.90 (m, 2H), 1.44 (dd, *J* = 6.8, 1.8 Hz, 6H); ^{13}C NMR (101 MHz, *Chloroform-d*) δ 159.6, 138.7, 134.2, 128.8, 128.3, 126.3, 125.0, 40.8, 31.9, 29.7, 22.2, 21.7; HRMS (ESI) calcd. for $C_{13}H_{18}NS$ (MH^+): 220.1154, found 220.1152; IR (film): νmax/cm^{-1} 2928, 1451, 1237, 759.

(*E*)-*S*-isopropyl-*N*-(pyridin-2-ylmethylene)thiohydroxylamine (6l-*i*Pr)

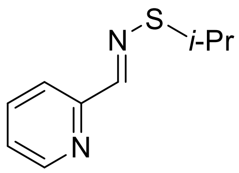

Synthesised from pyridin-2-ylmethanamine (0.620 mL, 6.00 mmol) using general procedure **2.1**. Purified by flash column chromatography on silica gel (eluent: DCM : MeOH = 50:1); brown oil (320 mg, 44% yield); ^{1}H NMR (400 MHz, *Chloroform-d*) δ 8.59 – 8.58 (m, 1H), 8.54 (s, 1H), 7.88 (d, *J* = 7.8 Hz, 1H), 7.70 (d, *J* = 7.8, Hz, 1H), 7.26 – 7.21 (m, 1H), 3.59 (hept, *J* = 6.8 Hz, 1H), 1.43 (d, *J* = 6.8 Hz, 6H); ^{13}C NMR (101 MHz, *Chloroform-d*) δ 156.3, 149.3, 136.3, 123.8, 120.2, 40.6, 21.4; HRMS (ESI) calcd. for $C_9H_{13}N_2S$ (MH^+): 181.0799, found 181.0792; IR (film): νmax/cm^{-1} 2961, 1464, 1432, 768.

(*E*)-*N*-benzylidene-*S*-(*tert*-butyl)thiohydroxylamine (6a-*t*Bu) [14]

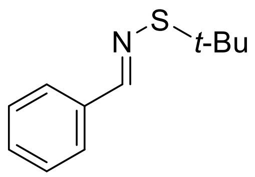

Synthesised from benzylamine (1.30 mL, 10.0 mmol) using general procedure **2.2**. Purified by flash column chromatography on silica gel (eluent: hexane : DCM = 5:1); yellow oil (350 mg, 36% yield over two steps); ^{1}H NMR (400 MHz, *Chloroform-d*) δ 8.47 (s, 1H), 7.68 – 7.58 (m, 2H), 7.44 – 7.32 (m, 3H), 1.47 (s, 9H); ^{13}C NMR (101 MHz, *Chloroform-d*) δ 155.3, 137.0, 129.6, 128.5, 126.8, 47.0, 29.2.

(*E*)-*S*-(*tert*-butyl)-*N*-(4-fluorobenzylidene)thiohydroxylamine (6b-*t*Bu)

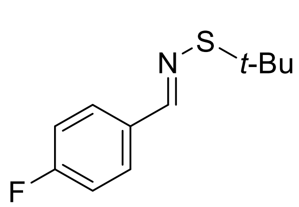

Synthesised from (4-fluorophenyl)methanamine (1.10 mL, 10.0 mmol) using general procedure **2.2**. Purified by flash column chromatography on silica gel (eluent: DCM : MeOH = 50:1); yellow oil (528 mg, 50% yield over two steps); ^{1}H NMR (400 MHz, *Chloroform-d*) δ 8.43 (s, 1H), 7.62 (dd, *J* = 8.7, 5.6 Hz, 2H), 7.06 (t, *J* = 8.7 Hz, 2H), 1.46 (s, 9H); ^{13}C NMR (101 MHz, *Chloroform-d*) δ 163.7 (d, *J* = 242.4 Hz), 154.0, 133.5 (d, *J* = 10.1 Hz), 128.7 (d, *J* = 10.1 Hz), 115.7 (d, *J* = 20.2 Hz), 47.1, 29.3; HRMS (ESI) calcd. for $C_{11}H_{15}FNS$ (MH^{+}): 212.0909, found 212.0904; IR (film): νmax/cm^{-1} 2961, 1599, 1507, 1228, 1151, 832.

(*E*)-*S*-(*tert*-butyl)-*N*-(4-chlorobenzylidene)thiohydroxylamine (6c-*t*Bu)

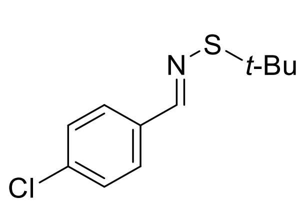

Synthesised from (4-chlorophenyl)methanamine (1.20 mL, 10.0 mmol) using general procedure **2.2**. Purified by flash column chromatography on silica gel (eluent: Hexane : DCM = 5:1); yellow oil (530 mg, 47% yield over two steps); ^{1}H NMR (400 MHz, *Chloroform-d*) δ 8.40 (s, 1H), 7.54 (d, *J* = 8.6 Hz, 2H), 7.32 (d, *J* = 8.6 Hz, 2H), 1.44 (s, 9H); ^{13}C NMR (101 MHz, *Chloroform-d*) δ 153.9, 135.5, 128.9, 128.0, 47.3, 29.3; HRMS (ESI) calcd. for $C_{11}H_{15}{}^{35}ClNS$ (MH^{+}): 228.0614, found 228.0608; IR (film): νmax/cm^{-1} 2960, 1488, 1360, 1165, 1086, 818.

(*E*)-*N*-(4-bromobenzylidene)-*S*-(*tert*-butyl)thiohydroxylamine (6d-*t*Bu)

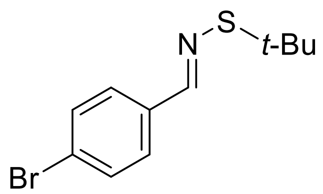

Synthesised from (4-bromophenyl)methanamine (1.80 g, 10.0 mmol) using general procedure **2.2**. Purified by flash column chromatography on silica gel (eluent: Hexane : DCM = 5:1); yellow oil (408 mg, 30% yield over two steps); ^{1}H NMR (400 MHz, *Chloroform-d*) δ 8.40 (s, 1H), 7.52 – 7.47 (m, 4H), 1.46 (s, 9H); ^{13}C NMR (101 MHz, *Chloroform-d*) δ 153.9, 135.9, 131.8, 128.2, 123.9, 47.3, 29.3; HRMS (ESI) calcd. for $C_{11}H_{15}{}^{79}BrNS$ (MH^+): 272.0109 and 274.0088, found 272.0103 and 274.0082; IR (film): νmax/cm^{-1} 2959, 1485, 1360, 1165, 1069, 1008, 814.

(*E*)-*S*-(*tert*-butyl)-*N*-(4-(trifluoromethyl)benzylidene)thiohydroxylamine (6e-*t*Bu)

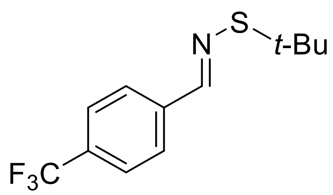

Synthesised from (4-(trifluoromethyl)phenyl)methanamine (1.40 mL, 10.0 mmol) using general procedure **2.2**. Purified by flash column chromatography on silica gel (eluent: hexane : DCM = 5:1); yellow oil (385 mg, 30% yield over two steps); ^{1}H NMR (400 MHz, *Chloroform-d*) δ 8.49 (s, 1H), 7.73 (d, *J* = 8.1 Hz, 2H), 7.63 (d, *J* = 8.1 Hz, 2H), 1.47 (s, 9H); ^{13}C NMR (101 MHz, *Chloroform-d*) δ 153.4, 139.8, 131.1 (q, *J* = 32.5 Hz), 126.9, 125.7 (q, *J* = 273.7 Hz), 125.62 (q, *J* = 3.8 Hz), 47.5, 29.3; HRMS (ESI) calcd. for $C_{12}H_{15}F_3NS$ (MH^+): 262.0877, found 262.0876; IR (film): νmax/cm^{-1} 2963, 1616, 1320, 1163, 1122, 1064, 831.

(*E*)-*S*-(*tert*-butyl)-*N*-(4-methoxybenzylidene)thiohydroxylamine (6f-*t*Bu)

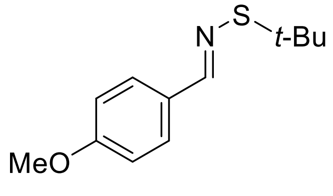

Synthesised from (4-methoxyphenyl)methanamine (1.30 mL, 10.0 mmol) using general procedure **2.2**. Purified by flash column chromatography on silica gel (eluent: hexane : DCM = 5:1); yellow oil (220 mg, 20% yield over two steps); ^{1}H NMR (400 MHz, *Chloroform-d*) δ 8.41 (s, 1H), 7.58 (d, *J* = 8.8 Hz, 2H), 6.90 (d, *J* = 8.8 Hz, 2H), 3.83 (s, 3H), 1.45 (s, 9H); ^{13}C NMR (101

MHz, *Chloroform-d*) δ 160.9, 155.0, 130.3, 128.3, 113.9, 55.3, 46.8, 29.2; HRMS (ESI) calcd. for $C_{12}H_{18}NOS$ (MH^+): 224.1103, found 224.1104; IR (film): νmax/cm^{-1} 2959, 1606, 1509, 1246, 1163, 828.

(*E*)-*S*-(*tert*-butyl)-*N*-(3-methoxybenzylidene)thiohydroxylamine (6g-*t*Bu)

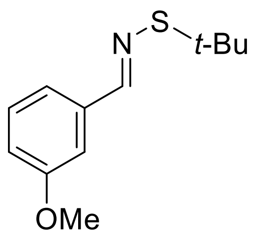

Synthesised from (3-methoxyphenyl)methanamine (1.30 mL, 10.0 mmol) using general procedure **2.2**. Purified by flash column chromatography on silica gel (eluent: hexane : DCM = 5:1); yellow oil (669 mg, 60% yield over two steps); ^{1}H NMR (400 MHz, *Chloroform-d*) δ 8.42 (s, 1H), 7.32 – 7.12 (m, 3H), 6.95 – 6.84 (m, 1H), 3.82 (s, 3H), 1.45 (s, 9H); ^{13}C NMR (101 MHz, *Chloroform-d*) δ 159.9, 155.2, 138.4, 129.6, 120.0, 115.8, 111.2, 55.4, 47.1, 29.3; HRMS (ESI) calcd. for $C_{12}H_{18}NOS$ (MH^+): 224.1103, found 224.1096; IR (film): νmax/cm^{-1} 2959, 1601, 1457, 1262, 1043, 779, 686.

(*E*)-*S*-(*tert*-butyl)-*N*-(naphthalen-1-ylmethylene)thiohydroxylamine (6h-*t*Bu)

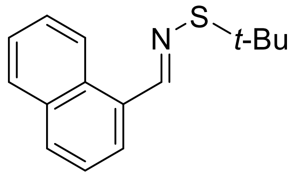

Synthesised from naphthalen-1-ylmethanamine (1.40 mL, 10.0 mmol) using general procedure **2.2**. Purified by flash column chromatography on silica gel (eluent: hexane : DCM = 5:1); yellow oil (480 mg, 40% yield over two steps); ^{1}H NMR (400 MHz, *Chloroform-d*) δ 9.14 (s, 1H), 9.00 – 8.85 (m, 1H), 7.93 – 7.75 (m, 3H), 7.63 – 7.43 (m, 3H), 1.55 (s, 9H); ^{13}C NMR (101 MHz, *Chloroform-d*) δ 155.6, 134.0, 132.3, 130.6, 130.4, 128.7, 127.9, 127.2, 126.1, 125.4, 124.6, 47.3, 29.5; HRMS (ESI) calcd. for $C_{15}H_{18}NS$ (MH^+): 244.1154, found 244.1157; IR (film): νmax/cm^{-1} 2959, 1454, 1360, 1164, 769.

(*E*)-*S*-(*tert*-butyl)-*N*-(1-phenylethylidene)thiohydroxylamine (6i-*t*Bu)

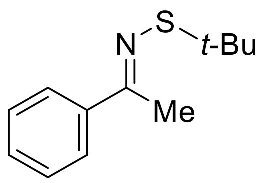

Synthesised from 1-phenylethan-1-amine (1.30 mL, 10.0 mmol) using general procedure **2.2**. Purified by flash column chromatography on silica gel (eluent: hexane : DCM = 5:1); yellow oil (1.00 g, 96% yield over two steps); ^{1}H NMR (400 MHz, *Chloroform-d*) δ 7.86 – 7.68 (m, 2H), 7.47 – 7.30 (m, 3H), 2.34 (s, 3H), 1.49 (s, 9H); ^{13}C NMR (101 MHz, *Chloroform-d*) δ 158.8, 139.9, 128.8, 128.3, 125.8, 46.8, 29.4, 19.5; HRMS (ESI) calcd. for $C_{12}H_{18}NS$ (MH^{+}): 208.1160, found 208.1157; IR (film): νmax/cm^{-1} 2959, 1337, 800, 774.

(*E*)-*S*-(*tert*-butyl)-*N*-(3,4-dihydronaphthalen-1(2H)-ylidene)thiohydroxylamine (6k-*t*Bu)

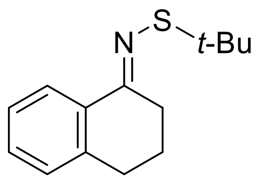

Synthesised from 1,2,3,4-tetrahydronaphthalen-1-amine (1.40 mL, 10.0 mmol) using general procedure **2.2**. Purified by flash column chromatography on silica gel (eluent: hexane : DCM = 5:1); yellow oil (620 mg, 53% yield over two steps); ^{1}H NMR (400 MHz, *Chloroform-d*) δ 8.16 – 7.99 (m, 1H), 7.25 – 7.19 (m, 2H), 7.12 – 7.09 (m, 1H), 2.82 – 2.73 (m, 2H), 2.58 (t, *J* = 6.6 Hz, 2H), 2.00 – 1.88 (m, 2H), 1.48 (s, 9H); ^{13}C NMR (101 MHz, *Chloroform-d*) δ 158.8, 138.8, 134.5, 128.8, 128.4, 126.4, 125.1, 46.7, 32.1, 29.8, 29.4, 22.3, 14.2; HRMS (ESI) calcd. for $C_{14}H_{20}NS$ (MH^{+}): 234.1311, found 234.1316; IR (film): νmax/cm^{-1} 2957, 1453, 1359, 1163, 759, 730.

(*E*)-*S*-(*tert*-butyl)-*N*-(pyridin-2-ylmethylene)thiohydroxylamine (6l-*t*Bu)

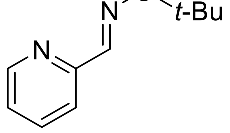

Synthesised from pyridin-2-ylmethanamine (1.10 g, 10.0 mmol) using general procedure **2.2**. Purified by flash column chromatography on silica gel (eluent: DCM : MeOH = 50:1); yellow oil (420 mg, 43% yield over two steps); ^{1}H NMR (400 MHz, *Chloroform-d*) δ 8.58 – 8.53 (m, 2H), 7.94 – 7.91 (m, 1H), 7.71 – 7.67 (m, 1H), 7.25 – 7.20 (m, 1H), 1.46 (s, 9H); ^{13}C NMR (101 MHz, *Chloroform-d*) δ 156.0,

154.8, 149.2, 136.3, 123.8, 120.0, 47.4, 29.2; HRMS (ESI) calcd. for $C_{10}H_{14}N_2NaS$ (MNa^+): 217.0770, found 217.0771; IR (film): νmax/cm^{-1} 2960, 1567, 1461, 1432, 1361, 1164, 767.

***S*-(*tert*-butyl)-*N*-(thiophen-2-ylmethylene)thiohydroxylamine (6m-*t*Bu)**

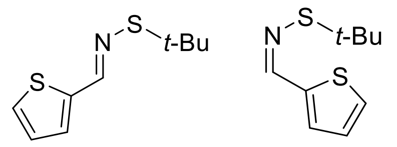

Synthesised from thiophen-2-ylmethanamine (1.00 mL, 10.0 mmol) using general procedure **2.2**. Purified by flash column chromatography on silica gel (eluent: hexane : DCM = 5:1); yellow oil (650 mg, 65% yield over two steps, *E/Z* 10:3); *E* isomer: ^{1}H NMR (400 MHz, *Chloroform-d*) δ 8.54 (s, 1H), 7.33 (dd, *J* = 5.0, 1.2 Hz, 1H), 7.12 (ddd, *J* = 3.6, 1.2, 0.6 Hz, 1H), 7.03 (ddd, *J* = 5.0, 3.6, 0.6 Hz, 1H), 1.44 (s, 9H); *Z* isomer: ^{1}H NMR (400 MHz, *Chloroform-d*) δ 8.49 (s, 1H), 7.62 (ddd, *J* = 5.0, 1.2, 0.5 Hz, 1H), 7.47 (ddd, *J* = 3.8, 1.2, 0.5 Hz, 1H), 7.16 (dd, *J* = 5.0, 3.8 Hz, 1H), 1.48 (s, 9H); *E* isomer: ^{13}C NMR (101 MHz, *Chloroform-d*) δ 149.3, 139.2, 128.1, 127.6, 127.3, 47.3, 29.2; *Z* isomer: ^{13}C NMR (101 MHz, *Chloroform-d*) δ 145.8, 143.9, 131.6, 130.6, 48.3, 29.0; HRMS (ESI) calcd. for $C_9H_{14}NS_2$ (MH^+): 200.0562, found 200.0570; IR (film): νmax/cm^{-1} 2959, 1360, 1163, 701.

***N*-((*E*)-((3r,5r,7r)-adamantan-1-yl)methylene)-*S*-(*tert*-butyl)thiohydroxylamine (6n-*t*Bu)**

Synthesised from ((3r,5r,7r)-adamantan-1-yl)methanamine (1.65 g, 10.0 mmol) using general procedure **2.2**. Purified by flash column chromatography on silica gel (eluent: hexane : DCM = 5:1); yellow waxy solid (1.00 g, 81% yield over two steps); ^{1}H NMR (400 MHz, *Chloroform-d*) δ 7.62 (s, 1H), 2.01 – 1.99 (m, 3H), 1.73 – 1.64 (m, 12H), 1.37 (s, 9H); ^{13}C NMR (101 MHz, *Chloroform-d*) δ 167.9, 46.3, 40.8, 39.5, 36.9, 31.7, 30.7, 29.1, 28.2; HRMS (ESI) calcd. for $C_{15}H_{26}NS$ (MH^+): 252.1780, found 252.1782; IR (film): ν_{max}/cm^{-1} 2902, 2848, 1452, 1360, 1165, 739.

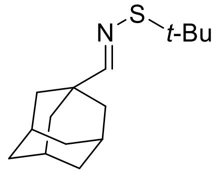

(*R,E*)-*N*-benzylidenepropane-2-sulfinamide ((*R*)-12a) [15]

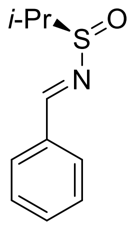

Synthesised from (*E*)-*N*-benzylidene-*S*-isopropylthiohydroxylamine (36.0 mg, 0.200 mmol) using general procedure **2.3**. Purified by flash column chromatography on silica gel (eluent: hexane : EtOAc = 10:1); yellow oil (20.0 mg, 51% yield); $[\alpha]_D^{20}$ = −72.23 (c = 1.0, $CHCl_3$), Lit. $[\alpha]_D^{20}$ = −112 (c = 1, $CHCl_3$); ^{1}H NMR (400 MHz, *Chloroform-d*) δ 8.59 (s, 1H), 7.88 – 7.82 (m, 2H), 7.56 – 7.44 (m, 3H), 2.98 (hept, *J* = 6.9 Hz, 1H), 1.31 (d, *J* = 6.9 Hz, 3H), 1.23 (d, *J* = 6.9 Hz, 3H); ^{13}C NMR (101 MHz, *Chloroform-d*) δ 162.5, 133.9, 132.5, 129.4, 128.9, 53.9, 14.7, 13.5; HRMS (ESI) calcd. for $C_{10}H_{14}NOS$ (MH^+): 196.0796, found 196.0791; IR (film): νmax/cm^{-1} 2933, 1581, 1185, 802; HPLC data: 98% *ee*, determined by HPLC (IBN5 column, flow rate: 1.0 mL/min, hexane/propanol: 95:5): tr(minor) = 9.01 min, tr(major) = 10.24 min. See Section 4 for images depicting the HPLC traces.

(*R,E*)-*N*-(4-fluorobenzylidene)propane-2-sulfinamide ((*R*)-12b)

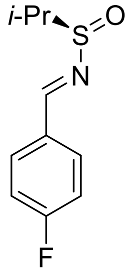

Synthesised from (*E*)-*N*-(4-fluorobenzylidene)-*S*-isopropylthiohydroxylamine (40.0 mg, 0.200 mmol) using general procedure **2.3**. Purified by flash column chromatography on silica gel (eluent: hexane : EtOAc = 10:1); yellow oil (30.0 mg, 70% yield); $[\alpha]_D^{20}$ = −29.92 (c = 1.0, $CHCl_3$); ^{1}H NMR (400 MHz, *Chloroform-d*) δ 8.54 (s, 1H), 7.96 – 7.79 (m, 2H), 7.25 – 7.09 (m, 2H), 2.96 (hept, *J* = 6.9 Hz, 1H), 1.25 (dd, *J* = 35.1, 6.9 Hz, 6H); ^{13}C NMR (101 MHz, *Chloroform-d*) δ 165.4 (d, *J* = 254.3 Hz), 161.2, 131.7 (d, *J* = 9.1 Hz), 130.5 (d, *J* = 3.1 Hz), 116.3 (d, *J* = 22.1 Hz), 54.0, 14.8, 13.6; ^{19}F NMR (376 MHz, *Chloroform-d*) δ −105.69; HRMS (ESI) calcd. for $C_{10}H_{12}FNONaS$ (MNa^+): 236.0521, found 236.0514; IR (film): νmax/cm^{-1} 2930, 1582, 1508, 1231, 1086; HPLC data: 99% *ee*, determined by HPLC (IBN5 column, flow rate: 1.0 mL/min,

hexane/propanol: 95:5): tr(minor)= 7.50 min, tr(major)= 10.54 min. See Section 4 for images depicting the HPLC traces.

(*R*,*E*)-*N*-(4-chlorobenzylidene)propane-2-sulfinamide ((*R*)-12c)

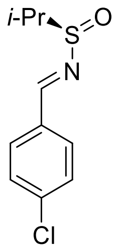

Synthesised from (*E*)-*S*-isopropyl-*N*-(4-chlorobenzylidene)thiohydroxylamine (43.0 mg, 0.200 mmol) using general procedure **2.3**. Purified by flash column chromatography on silica gel (eluent: hexane : EtOAc = 10:1); yellow oil (37.0 mg, 80% yield); $[\alpha]_D^{20} = -56.37$ (c = 1.5, $CHCl_3$); ^1H NMR (400 MHz, *Chloroform-d*) δ 8.54 (s, 1H), 7.79 (d, *J* = 8.5 Hz, 2H), 7.45 (d, *J* = 8.5 Hz, 2H), 2.97 (hept, *J* = 6.9 Hz, 1H), 1.30 (d, *J* = 6.9 Hz, 3H), 1.22 (d, *J* = 6.8 Hz, 3H); ^{13}C NMR (101 MHz, *Chloroform-d*) δ 161.4, 138.8, 132.5, 130.6, 129.4, 54.0, 14.9, 13.7; HRMS (ESI) calcd. for $C_{10}H_{13}{}^{35}ClNOS$ (MH^+): 230.0406, found 230.0401; IR (film): $\nu max/cm^{-1}$ 2924, 1591, 1085, 825; HPLC data: 98% *ee*, determined by HPLC (IBN5 column, flow rate: 1.0 mL/min, hexane/propanol: 95:5): tr(minor) = 7.81 min, tr(major) = 11.51 min. See Section 4 for images depicting the HPLC traces.

(*R*,*E*)-*N*-(4-bromobenzylidene)propane-2-sulfinamide ((*R*)-12d)

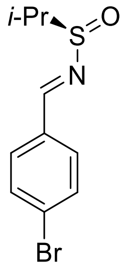

Synthesised from (*E*)-*N*-(4-bromobenzylidene)-*S*-isopropylthiohydroxylamine (26.0 mg, 0.100 mmol) using general procedure **2.3**. Purified by flash column chromatography on silica gel (eluent: hexane : EtOAc = 10:1); yellow oil (17.0 mg, 62% yield); $[\alpha]_D^{20} = -13.57$ (c = 1.0, $CHCl_3$); ^1H NMR (400 MHz, *Chloroform-d*) δ 8.53 (s, 1H), 7.72 (d, *J* = 8.6 Hz, 2H), 7.62 (d, *J* = 8.6 Hz, 2H), 2.98 (hept, *J* = 6.9 Hz, 1H), 1.31 (d, *J* = 6.9 Hz, 3H), 1.22 (d, *J* = 6.9 Hz, 3H); ^{13}C NMR (101 MHz, *Chloroform-d*) δ 161.5, 132.9, 132.4, 130.8, 127.4, 54.0, 14.9, 13.7; HRMS (ESI) calcd. for $C_{10}H_{12}{}^{79}BrNONaS$ (MNa^+): 297.9700 and 295.9721, found 297.9694 and 295.9718; IR (film): $\nu max/cm^{-1}$ 2924, 1608, 1088, 821; HPLC data: 99% *ee*, determined by HPLC (IBN5 column, flow rate: 1.0

mL/min, hexane/propanol: 95:5): tr(minor)= 8.44 min, tr(major)= 12.79 min. See Section 4 for images depicting the HPLC traces.

(*R*,*E*)-*N*-(4-(trifluoromethyl)benzylidene)propane-2-sulfinamide ((*R*)-12e)

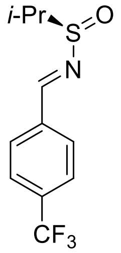

Synthesised from (*E*)-*S*-isopropyl-*N*-(4-trifluoromethylbenzylidene)thiohydroxylamine (50.0 mg, 0.200 mmol) using general procedure **2.3**. Purified by flash column chromatography on silica gel (eluent: hexane : EtOAc = 10:1); yellow oil (31 mg, 60% yield); $[\alpha]_D^{20} = -100.14$ (c = 1.0, $CHCl_3$); ^1H NMR (400 MHz, *Chloroform-d*) δ 8.63 (s, 1H), 7.96 (d, *J* = 8.1 Hz, 2H), 7.73 (d, *J* = 8.1 Hz, 2H), 3.01 (hept, *J* = 6.9 Hz, 1H), 1.32 (d, *J* = 6.9 Hz, 3H), 1.23 (d, *J* = 6.9 Hz, 3H); ^{13}C NMR (101 MHz, *Chloroform-d*) δ 161.4, 136.8, 133.8 (q, *J* = 32.6 Hz), 129.6, 126.1 (q, *J* = 3.8 Hz), 123.2 (q, *J* = 274.2 Hz), 54.1, 14.9, 13.7; ^{19}F NMR (376 MHz, *Chloroform-d*) δ –62.94; HRMS (ESI) calcd. for $C_{11}H_{12}F_3NONaS$ (MNa^+): 286.0489, found 286.0486; IR (film): νmax/cm^{-1} 2933, 1322, 1121, 1065; HPLC data: 98% *ee*, determined by HPLC (IBN5 column, flow rate: 1.0 mL/min, hexane/propanol: 95:5): tr(minor) = 7.42 min, tr(major) = 15.36 min. See Section 4 for images depicting the HPLC traces.

(*R*,*E*)-*N*-(4-methoxybenzylidene)propane-2-sulfinamide ((*R*)-12f) [15]

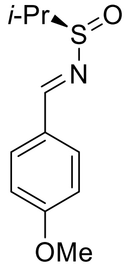

Synthesised from (*E*)-*S*-isopropyl-*N*-(4-methoxybenzylidene)thiohydroxylamine (23.0 mg, 0.100 mmol) using general procedure **2.3**. Purified by flash column chromatography on silica gel (eluent: hexane : EtOAc = 10:1); yellow oil (18.0 mg, 74% yield); $[\alpha]_D^{20} = -34.84$ (c = 1.0, $CHCl_3$), Lit. $[\alpha]_D^{20} = 71$ for the opposite *S* isomer (c = 1, $CHCl_3$); ^1H NMR (400 MHz, *Chloroform-d*) δ 8.51 (s, 1H), 7.80 (d, *J* = 8.7 Hz, 2H), 6.97 (d, *J* = 8.7 Hz, 2H), 3.87 (s, 3H), 2.94 (hept, *J* = 6.9 Hz, 1H), 1.30 (d, *J* = 6.9 Hz, 3H), 1.21 (d, *J* = 6.9 Hz, 3H); ^{13}C NMR (101 MHz, *Chloroform-d*) δ 163.1, 161.6, 131.3, 127.2, 114.4, 55.5, 53.9, 14.8, 13.5; HRMS (ESI) calcd. for $C_{11}H_{16}NO_2S$ (MH^+): 226.0902, found

226.0897; IR (film): vmax/cm^{-1} 2965, 1594, 1568, 1511, 1253, 1028; HPLC data: 98% *ee*, determined by HPLC (IBN5 column, flow rate: 1.0 mL/min, hexane/propanol: 95:5): tr(minor)= 10.05 min, tr(major) = 12.57 min. See Section 4 for images depicting the HPLC traces.

(*R*,*E*)-*N*-(3-methoxybenzylidene)propane-2-sulfinamide ((*R*)-12g)[16]

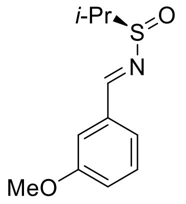

Synthesised from (*E*)-*S*-isopropyl-*N*-(3-methoxybenzylidene)thiohydroxylamine (42.0 mg, 0.200 mmol) using general procedure **2.3**. Purified by flash column chromatography on silica gel (eluent: hexane : EtOAc = 10:1); yellow oil (25.0 mg, 56% yield); $[\alpha]_D^{20}$ = −47.78 (c = 1.0, $CHCl_3$), Lit. $[\alpha]_D^{20}$ = −74.8 (c = 1, $CHCl_3$); ^{1}H NMR (400 MHz, *Chloroform-d*) δ 8.55 (s, 1H), 7.42 – 7.34 (m, 3H), 7.07 (dt, *J* = 6.9, 2.7 Hz, 1H), 3.86 (s, 3H), 2.98 (hept, *J* = 6.9 Hz, 1H), 1.31 (d, *J* = 6.9 Hz, 3H), 1.22 (d, *J* = 6.9 Hz, 3H); ^{13}C NMR (101 MHz, *Chloroform-d*) δ 162.4, 160.0, 135.2, 130.0, 122.6, 119.0, 112.9, 55.4, 53.8, 14.8, 13.5; HRMS (ESI) calcd. for $C_{11}H_{15}NO_2NaS$ (MNa^+): 248.0721, found 248.0717; IR (film): vmax/cm^{-1} 2965, 1607, 1577, 1266, 1085; HPLC data: 94% *ee*, determined by HPLC (IBN5 column, flow rate: 1.0 mL/min, hexane/propanol: 95:5): tr(minor) = 9.14 min, tr(major) = 11.22 min. See Section 4 for images depicting the HPLC traces.

(*R*,*E*)-*N*-(naphthalen-1-ylmethylene)propane-2-sulfinamide ((*R*)-12h)

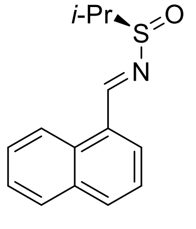

Synthesised from (*E*)-*S*-isopropyl-*N*-(naphthalen-1-ylmethylene)thiohydroxylamine (23.0 mg, 0.100 mmol) using general procedure **2.3**. Purified by flash column chromatography on silica gel (eluent: hexane : EtOAc = 10:1); yellow oil (11.0 mg, 45% yield); $[\alpha]_D^{20}$ = −2.08 (c = 1.0, $CHCl_3$); ^{1}H NMR (400 MHz, *Chloroform-d*) δ 9.17 (s, 1H), 9.02 (dd, *J* = 8.5, 1.1 Hz, 1H),

8.13 – 7.99 (m, 2H), 7.93 (d, J = 8.5 Hz, 1H), 7.71 – 7.56 (m, 3H), 3.06 (hept, J = 6.9 Hz, 1H), 1.37 (d, J = 6.9 Hz, 3H), 1.29 (d, J = 6.9 Hz, 3H); ^{13}C NMR (101 MHz, *Chloroform-d*) δ 162.4, 134.0, 133.4, 132.0, 131.3, 129.4, 128.9, 128.1, 126.6, 125.3, 124.5, 54.1, 14.9, 13.8; HRMS (ESI) calcd. for $C_{14}H_{15}NONaS$ (MNa^+): 268.0772, found 268.0768; IR (film): νmax/cm^{-1} 2927, 1582, 1087, 774; HPLC data: 93% *ee*, determined by HPLC (IC column, flow rate: 1.0 mL/min, hexane/propanol: 95:5): tr(minor) = 24.51 min, tr(major)= 22.23 min. See Section 4 for images depicting the HPLC traces.

(*R*,*E*)-*N*-(1-phenylethylidene)propane-2-sulfinamide ((*R*)-12i)

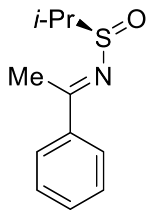

Synthesised from (*E*)-*S*-isopropyl-*N*-(1-phenylethylidene)thiohydroxylamine (40.0 mg, 0.200 mmol) using general procedure **2.3**. Purified by flash column chromatography on silica gel (eluent: hexane : EtOAc = 10:1); yellow oil (31.0 mg, 74% yield); $[\alpha]_D^{20}$ = −6.4 (c = 1.0, $CHCl_3$); ^{1}H NMR (400 MHz, *Chloroform-d*) δ 7.89 (d, J = 7.3 Hz, 2H), 7.52 – 7.39 (m, 3H), 3.01 (hept, J = 6.9 Hz, 1H), 2.76 (s, 3H), 1.35 (d, J = 4.1 Hz, 1H), 1.34 (d, J = 4.1 Hz, 1H); ^{13}C NMR (101 MHz, *Chloroform-d*) δ 176.2, 138.6, 131.7, 128.5, 127.2, 54.7, 19.8, 14.7, 14.4; HRMS (ESI) calcd. for $C_{11}H_{15}NNaOS$ (MNa^+): 232.0772, found 232.0767; IR (film): νmax/cm^{-1} 2985, 1565, 1460, 1224, 785; HPLC data: 97% *ee*, determined by HPLC (IBN5 column, flow rate: 1.0 mL/min, hexane/propanol: 95:5): tr(minor) = 12.07 min, tr(major) = 10.51 min. See Section 4 for images depicting the HPLC traces.

(*R*)-*N*-(1-(naphthalen-1-yl)ethylidene)propane-2-sulfinamide ((*R*)-12j)

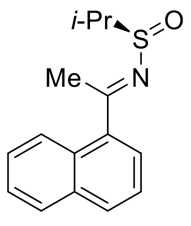

Synthesised from *S*-isopropyl-*N*-(1-(naphthalen-1-yl)ethylidene)thiohydroxylamine (122 mg, 0.500 mmol) using general procedure **2.3**. Purified by flash column chromatography on silica gel (eluent: hexane : EtOAc = 10:1); yellow oil (45.0 mg, 35% yield, *E/Z* 2:1); $[\alpha]_D^{20}$ = −40.3 (c = 1.0,

$CHCl_3$); ^{1}H NMR (400 MHz, *Chloroform-d*) δ 8.23 – 8.15 (m, 1H, *Z* isomer), 7.91– 7.89 (m, 3H, *E*+*Z* isomer), 7.60 – 7.42 (m, 6H, *E*+*Z* isomer), 3.06 (hept, *J* = 6.9 Hz, 1H, *E* isomer), 2.87 (s, 3H, *E* isomer), 2.79 (p, *J* = 7.4 Hz, 3H, *Z* isomer), 2.64 (s, 3H, *Z* isomer), 1.36 (d, *J* = 1.9 Hz, 3H, *E* isomer), 1.34 (d, *J* = 1.9 Hz, 3H, *E* isomer), 1.23 (d, *J* = 7.0 Hz, 3H, *Z* isomer), 1.16 (d, *J* = 7.0 Hz, 3H, *Z* isomer); ^{13}C NMR (101 MHz, *Chloroform-d*) *E* isomer: δ 181.1, 139.0, 133.8, 130.2, 128.6, 126.9, 126.2, 125.1, 125.0, 124.9, 54.4, 24.9, 14.9, 14.1; *Z* isomer: δ 133.2, 129.4, 129.3, 129.0, 126.4, 123.9, 123.6, 122.4, 53.6, 30.7, 13.9 (two peaks missing from this isomer); HRMS (ESI) calcd. for $C_{15}H_{17}NONaS$ (MNa^+): 282.0929, found 282.0926; IR (film): νmax/cm^{-1} 2926, 1738, 1237, 1045, 730; HPLC data: 99% *ee*, determined by HPLC (IBN5 column, flow rate: 1.0 mL/min, hexane/propanol: 95:5): tr(minor) = 15.86 min, tr(major) = 14.54 min. See Section 4 for images depicting the HPLC traces.

(*R*,*E*)-*N*-(3,4-dihydronaphthalen-1(2H)-ylidene)propane-2-sulfinamide ((*R*)-12k)

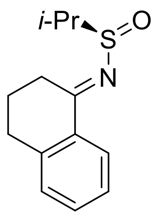

Synthesised from (*E*)-*N*-(3,4-dihydronaphthalen-1(2H)-ylidene)-*S*-isopropylthiohydroxylamine (22.0 mg, 0.100 mmol) using general procedure **2.3**. Purified by flash column chromatography on silica gel (eluent: hexane : EtOAc = 10:1); yellow oil (21.0 mg, 89% yield); $[\alpha]_D^{20}$ = 1.94 (c = 0.5, $CHCl_3$); ^{1}H NMR (400 MHz, *Chloroform-d*) δ 8.18 (dd, *J* = 7.8, 1.4 Hz, 1H), 7.39 (td, *J* = 7.8, 1.4 Hz, 1H), 7.28 – 7.23 (m, 2H), 7.19 (dd, *J* = 7.8, 1.4 Hz, 1H), 3.28 (ddd, *J* = 17.4, 9.0, 4.9 Hz, 1H), 3.07 – 2.96 (m, 2H), 2.90 – 2.85 (m, 2H), 2.09 – 1.92 (m, 2H), 1.36 (d, *J* = 4.8 Hz, 3H), 1.34 (d, *J* = 4.8 Hz, 3H)); ^{13}C NMR (101 MHz, *Chloroform-d*) δ 176.8, 142.3, 132.9, 132.1, 129.0, 127.2, 126.6, 54.7, 32.4, 29.6, 22.7, 14.7, 14.6; HRMS (ESI) calcd. for $C_{13}H_{17}NONaS$ (MNa^+): 258.0929, found 258.0926; IR (film): νmax/cm^{-1} 2927, 1583, 1509, 1231, 1087; HPLC data: 93% *ee*, determined by HPLC (IC column, flow rate: 1.0 mL/min, hexane/propanol: 95:5):

tr(minor)= 14.70 min, tr(major)= 10.75 min. See Section 4 for images depicting the HPLC traces.

(*R*,*E*)-*N*-(pyridin-2-ylmethylene)propane-2-sulfinamide ((*R*)-12l)

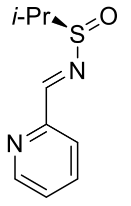

Synthesised from (*E*)-*S*-isopropyl-*N*-(pyridin-2-ylmethylene)thiohydroxylamine (36.0 mg, 0.200 mmol) using general procedure **2.3**. Purified by flash column chromatography on silica gel (eluent: DCM : MeOH = 25:1); brown oil (28.0 mg, 71% yield); $[\alpha]_D^{20} = -67.66$ (c = 1.0, $CHCl_3$); ^{1}H NMR (400 MHz, *Chloroform-d*) δ 8.75 (ddd, *J* = 4.8, 1.2, 0.9 Hz, 1H), 8.69 (s, 1H), 7.98 (dt, *J* = 7.7, 1.2 Hz, 1H), 7.87 – 7.77 (m, 1H), 7.40 (ddd, *J* = 7.7, 4.8, 1.2 Hz, 1H), 3.05 (hept, *J* = 6.9 Hz, 1H), 1.33 (d, *J* = 6.9 Hz, 3H), 1.21 (d, *J* = 6.9 Hz, 3H); ^{13}C NMR (101 MHz, *Chloroform-d*) δ 163.4, 152.3, 150.4, 136.9, 126.1, 123.7, 53.8, 15.2, 13.4; HRMS (ESI) calcd. for $C_9H_{13}N_2OS$ (MH^+): 197.0749, found 197.0743; IR (film): vmax/cm^{-1} 2988, 1587, 1464, 1124, 785; HPLC data: 90% *ee*, determined by HPLC (IBN5 column, flow rate: 1.0 mL/min, hexane/propanol: 95:5): tr(minor) = 15.75 min, tr(major) = 18.34 min. See Section 4 for images depicting the HPLC traces.

(*S*,*E*)-*N*-benzylidene-2-methylpropane-2-sulfinamide ((*S*)-13a) [17]

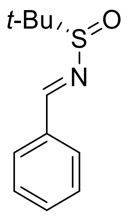

Synthesised from (*E*)-*N*-benzylidene-*S*-(*tert*-butyl)thiohydroxylamine (40.0 mg, 0.200 mmol) using general procedure **2.4**. Purified by flash column chromatography on silica gel (eluent: hexane : EtOAc = 10:1); yellow oil (37.0 mg, 88% yield); $[\alpha]_D^{20} = 75.62$ (c = 1, $CHCl_3$), Lit. $[\alpha]_D^{20} = 122.38$ (c = 1, $CHCl_3$); ^{1}H NMR (400 MHz, *Chloroform-d*) δ 8.59 (s, 1H), 7.85 (dt, *J* = 6.7, 1.6 Hz, 2H), 7.57 – 7.43 (m, 3H), 1.26 (s, 9H); ^{13}C NMR (101 MHz, *Chloroform-d*) δ 162.8, 134.2, 132.5, 129.5, 129.0, 57.9, 22.7; HPLC data: 99% *ee*, determined by HPLC (IC column, flow rate: 1.0 mL/min, hexane/propanol: 95:5): tr(minor) = 14.45 min, tr(major) = 13.60 min. See Section 4 for images depicting the HPLC traces.

(*S,E*)-*N*-(4-fluorobenzylidene)-2-methylpropane-2-sulfinamide ((*S*)-13b) [18]

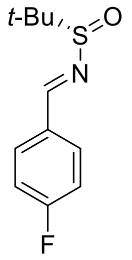

Synthesised from (*E*)-*S*-(*tert*-butyl)-N-(4-fluorobenzylidene)thiohydroxylamine (43.0 mg, 0.200 mmol) using general procedure **2.4**. Purified by flash column chromatography on silica gel (eluent: hexane : EtOAc = 10:1); colourless oil (32.0 mg, 70% yield); $[\alpha]_D^{20}$ = 117.66 (c = 1.0, $CHCl_3$); ^{1}H NMR (400 MHz, *Chloroform-d*) δ 8.54 (s, 1H), 7.86 (dd, *J* = 8.7, 5.4 Hz, 2H), 7.15 (t, *J* = 8.7 Hz, 2H), 1.25 (s, 9H); ^{13}C NMR (101 MHz, *Chloroform-d*) δ 165.3 (d, *J* = 254.0 Hz), 161.4, 131.6 (d, *J* = 9.1 Hz), 130.6 (d, *J* = 2.9 Hz), 116.3 (d, *J* = 22.2 Hz), 57.9, 22.7; ^{19}F NMR (376 MHz, Chloroform-d) δ –105.8; HPLC data: 94% *ee*, determined by HPLC (IC column, flow rate: 1.0 mL/min, hexane/propanol: 95:5): tr(minor) = 14.49 min, tr(major) = 13.39 min. See Section 4 for images depicting the HPLC traces.

(*S,E*)-*N*-(4-chlorobenzylidene)-2-methylpropane-2-sulfinamide ((*S*)-13c) [17]

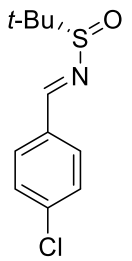

Synthesised from (*E*)-*S*-(*tert*-butyl)-N-(4-chlorobenzylidene)thiohydroxylamine (45.0 mg, 0.200 mmol) using general procedure **2.4**. Purified by flash column chromatography on silica gel (eluent: hexane : EtOAc = 10:1); colourless oil (39.0 mg, 80% yield); $[\alpha]_D^{20}$ = 67.17 (c = 1.0, $CHCl_3$), Lit. $[\alpha]_D^{20}$ = 77.1 (c = 1, $CHCl_3$); ^{1}H NMR (400 MHz, *Chloroform-d*) δ 8.54 (s, 1H), 7.78 (d, *J* = 8.6 Hz, 2H), 7.44 (d, *J* = 8.6 Hz, 2H), 1.25 (s, 9H); ^{13}C NMR (101 MHz, *Chloroform-d*) δ 161.4, 138.6, 132.5, 130.5, 129.3, 129.3, 57.9, 22.6; HPLC data: 98% *ee*, determined by HPLC (IC column, flow rate: 1.0 mL/min, hexane/propanol: 95:5): tr(minor) = 15.02 min, tr(major) = 13.78 min. See Section 4 for images depicting the HPLC traces.

(*S,E*)-*N*-(4-bromobenzylidene)-2-methylpropane-2-sulfinamide ((*S*)-13d) [19]

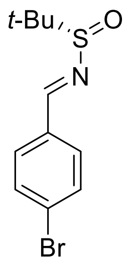

Synthesised from (*E*)-*S*-(*tert*-butyl)-*N*-(4-bromobenzylidene)thiohydroxylamine (55.0 mg, 0.200 mmol) using general procedure **2.4**. Purified by flash column chromatography on silica gel (eluent: hexane : EtOAc = 10:1); colourless oil (38.0 mg, 66% yield); $[\alpha]_D^{20}$ = 74.58 (c = 1.0, $CHCl_3$); ^{1}H NMR (400 MHz, *Chloroform-d*) δ 8.53 (s, 1H), 7.71 (d, *J* = 8.5 Hz, 2H), 7.61 (d, *J* = 8.5 Hz, 2H), 1.26 (s, 9H); ^{13}C NMR (101 MHz, *Chloroform-d*) δ 161.7, 133.0, 132.4, 130.8, 127.3, 58.0, 22.7; HPLC data: 99% *ee*, determined by HPLC (IC column, flow rate: 1.0 mL/min, hexane/propanol: 95:5): tr(minor)= 13.46 min, tr(major)= 14.60 min. See Section 4 for images depicting the HPLC traces.

(*S,E*)-2-methyl-*N*-(4-(trifluoromethyl)benzylidene)propane-2-sulfinamide ((*S*)-13e) [20]

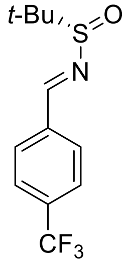

Synthesised from (*E*)-*S*-(*tert*-butyl)-*N*-(4-trifluoromethylbenzylidene)thiohydroxylamine (53.0 mg, 0.200 mmol) using general procedure **2.4**. Purified by flash column chromatography on silica gel (eluent: hexane : EtOAc = 10:1); colourless oil (30.0 mg, 54% yield); $[\alpha]_D^{20}$ = 77.42 (c = 1.0, $CHCl_3$), Lit. $[\alpha]_D^{20}$ = 74.9 (c = 1, $CHCl_3$); ^{1}H NMR (400 MHz, *Chloroform-d*) δ 8.63 (s, 1H), 7.97 (d, *J* = 8.0 Hz, 2H), 7.74 (d, *J* = 8.0 Hz, 2H), 1.28 (s, 9H); ^{13}C NMR (101 MHz, *Chloroform-d*) δ 161.6, 136.9, 133.7 (q, *J* = 32.8 Hz), 129.6, 126.0 (q, *J* = 3.8 Hz), 125.8 (q, *J* = 273.7 Hz), 58.3, 22.7; ^{19}F NMR (376 MHz, Chloroform-d) δ −62.9; HPLC data: 98% *ee*, determined by HPLC (IC column, flow rate: 1.0 mL/min, hexane/propanol: 95:5): tr(minor) = 11.01 min, tr(major) = 9.44 min. See Section 4 for images depicting the HPLC traces.

(*S,E*)-*N*-(4-methoxybenzylidene)-2-methylpropane-2-sulfinamide ((*S*)-13f) [21]

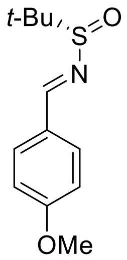

Synthesised from (*E*)-*S*-(*tert*-butyl)-*N*-(4-methoxybenzylidene)thiohydroxylamine (45.0 mg, 0.200 mmol) using general procedure **2.4**. Purified by flash column chromatography on silica gel (eluent: hexane : EtOAc = 10:1); yellow oil (40.0 mg, 83% yield); $[\alpha]_D^{20}$ = 92.11 (c = 1, $CHCl_3$), Lit. $[\alpha]_D^{20}$ = 45.4 (c = 1, CH_2Cl_2); ^{1}H NMR (400 MHz, *Chloroform-d*) δ 8.50 (s, 1H), 7.80 (d, *J* = 9.0 Hz, 2H), 6.96 (d, *J* = 9.0 Hz, 2H), 3.86 (s, 3H), 1.24 (s, 9H); ^{13}C NMR (101 MHz, *Chloroform-d*) δ 163.0, 161.7, 131.2, 127.3, 114.3, 57.5, 55.5, 22.5; HPLC data: 96% *ee*, determined by HPLC (AD-H column, flow rate: 1.0 mL/min, hexane/propanol: 95:5): tr(minor) = 14.92 min, tr(major) = 15.61 min. See Section 4 for images depicting the HPLC traces.

(*S,E*)-*N*-(3-methoxybenzylidene)-2-methylpropane-2-sulfinamide ((*S*)-13g) [17]

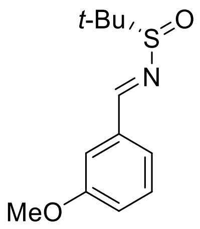

Synthesised from (*E*)-*S*-(*tert*-butyl)-*N*-(3-methoxybenzylidene)thiohydroxylamine (45.0 mg, 0.200 mmol) using general procedure **2.4**. Purified by flash column chromatography on silica gel (eluent: hexane : EtOAc = 10:1); yellow oil (35.0 mg, 72% yield); $[\alpha]_D^{20}$ = 86.70 (c = 1, $CHCl_3$), Lit. $[\alpha]_D^{20}$ = 76.7 (c = 1, $CHCl_3$); ^{1}H NMR (400 MHz, *Chloroform-d*) δ 8.55 (s, 1H), 7.43 – 7.35 (m, 3H), 7.12 – 7.03 (m, 1H), 3.86 (s, 3H), 1.26 (s, 9H); ^{13}C NMR (101 MHz, *Chloroform-d*) δ 162.6, 160.0, 135.4, 130.0, 122.5, 118.8, 113.1, 57.8, 55.4, 22.6; HPLC data: 96% *ee*, determined by HPLC (AD-H column, flow rate: 1.0 mL/min, hexane/propanol: 95:5): tr(minor) = 10.40 min, tr(major) = 9.59 min. See Section 4 for images depicting the HPLC traces.

(*S,E*)-2-methyl-*N*-(naphthalen-1-ylmethylene)propane-2-sulfinamide ((*S*)-13h) [22]

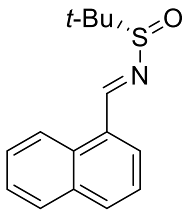

Synthesised from (*E*)-*S*-(*tert*-butyl)-*N*-(naphthalen-1-ylmethylene)thiohydroxylamine (50.0 mg, 0.200 mmol) using general procedure **2.4**. Purified by flash column chromatography on silica gel (eluent: hexane : EtOAc = 10:1); colourless oil (28.0 mg, 55% yield); $[\alpha]_D^{20}$ = 6.02 (c = 1.0, $CHCl_3$), Lit. $[\alpha]_D^{20}$ = 1.45 (c = 1, $CHCl_3$); ^{1}H NMR (400 MHz, *Chloroform-d*) δ 9.16 (s, 1H), 9.04 (d, *J* = 8.5 Hz, 1H), 8.05 (d, *J* = 7.4 Hz, 1H), 8.02 (d, *J* = 8.5 Hz, 1H); 7.93 (d, *J* = 7.7 Hz, 1H), 7.67 – 7.62 (m, 1H), 7.61 – 7.56 (m, 2H), 1.33 (s, 9H); ^{13}C NMR (101 MHz, *Chloroform-d*) δ 162.6, 134.0, 133.4, 132.1, 131.3, 129.5, 128.9, 128.1, 126.6, 125.3, 124.5, 57.8, 22.7; HPLC data: 95% *ee*, determined by HPLC (IBN5 column, flow rate: 1.0 mL/min, hexane/propanol: 95:5): tr(minor) = 9.74 min, tr(major) = 7.79 min. See Section 4 for images depicting the HPLC traces.

(*S,E*)-2-methyl-*N*-(1-phenylethylidene)propane-2-sulfinamide ((*S*)-13i) [23]

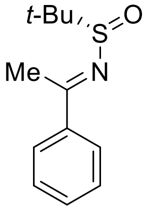

Synthesised from (*E*)-*S*-(*tert*-butyl)-*N*-(1-phenylethylidene)thiohydroxylamine (42.0 mg, 0.200 mmol) using general procedure **2.4**. Purified by flash column chromatography on silica gel (eluent: hexane : EtOAc = 10:1); colourless oil (27.0 mg, 61% yield); $[\alpha]_D^{20}$ = 5.19 (c = 1.0, $CHCl_3$), Lit. $[\alpha]_D^{20}$ = 13 (c = 1.03, CH_2Cl_2); ^{1}H NMR (400 MHz, *Chloroform-d*) δ 7.89 (d, *J* = 7.3 Hz, 2H), 7.54 – 7.39 (m, 3H), 2.77 (s, 3H), 1.32 (s, 9H); ^{13}C NMR (101 MHz, *Chloroform-d*) δ 176.5, 138.9, 131.8, 128.6, 127.3, 57.5, 22.6, 19.9; HPLC data: 98% *ee*, determined by HPLC (IC column, flow rate: 1.0 mL/min, hexane/propanol: 95:5): tr(minor)= 34.77 min, tr(major)= 41.62 min. See Section 4 for images depicting the HPLC traces.

(*S,E*)-*N*-(3,4-dihydronaphthalen-1(2H)-ylidene)-2-methylpropane-2-sulfinamide ((*S*)-13k)[23]

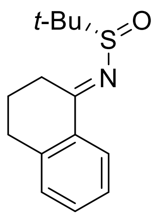

Synthesised from (*E*)-*S*-(*tert*-butyl)-N-(3,4-dihydronaphthalen-1(2H)-ylidene)thiohydroxylamine (47.0 mg, 0.200 mmol) using general procedure **2.4**. Purified by flash column chromatography on silica gel (eluent: hexane : EtOAc = 10:1); colourless oil (21.0 mg, 42% yield); $[\alpha]_D^{20}$ = 20.08 (c = 1.00, $CHCl_3$), Lit. $[\alpha]^{20}_D$ = 27 (c = 0.84, CH_2Cl_2); ^{1}H NMR (400 MHz, *Chloroform-d*) δ 8.17 (dd, *J* = 7.9, 1.4 Hz, 1H), 7.38 (td, *J* = 7.9, 1.4 Hz, 1H), 7.27 – 7.22 (m, 1H), 7.21 – 7.13 (m, 1H), 3.32 – 3.22 (m, 1H), 3.09 – 3.02 (m, 1H), 2.89 – 2.86 (m, 2H), 2.07 – 1.91 (m, 2H), 1.32 (s, 9H); ^{13}C NMR (101 MHz, *Chloroform-d*) δ 177.1, 142.3, 133.2, 132.1, 129.0, 127.1, 126.6, 57.3, 32.5, 29.6, 22.8, 22.6; HPLC data: 97% *ee*, determined by HPLC (IC column, flow rate: 1.0 mL/min, hexane/propanol: 95:5): tr(minor) = 35.41 min, tr(major) = 38.59 min. See Section 4 for images depicting the HPLC traces.

(*S,E*)-2-methyl-*N*-(pyridin-2-ylmethylene)propane-2-sulfinamide ((*S*)-13l) [24]

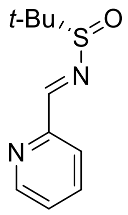

Synthesised from (*E*)-*S*-(*tert*-butyl)-*N*-(pyridin-2-ylmethylene)thiohydroxylamine (39.0 mg, 0.200 mmol) using general procedure **2.4**. Purified by flash column chromatography on silica gel (eluent: DCM : MeOH = 50:1); black oil (34.0 mg, 81% yield); $[\alpha]_D^{20}$ = 142.59 (c = 1.0, $CHCl_3$); ^{1}H NMR (400 MHz, *Chloroform-d*) δ 8.74 (ddd, *J* = 4.8, 1.5, 0.8 Hz, 1H), 8.69 (s, 1H), 8.01 (dt, *J* = 7.9, 1.5 Hz, 1H), 7.81 (tdd, *J* = 7.9, 1.5, 0.8 Hz, 1H), 7.40 (ddd, *J* = 7.9, 4.8, 1.5 Hz, 1H), 1.28 (s, 9H); ^{13}C NMR (101 MHz, *Chloroform-d*) δ 163.8, 152.5, 150.3, 136.9, 126.0, 123.2, 58.2, 22.8; HPLC data: 92% *ee*, determined by HPLC (IC column, flow rate: 1.0 mL/min, hexane/propanol: 95:5): tr(minor) = 14.93 min, tr(major) = 11.54 min. See Section 4 for images depicting the HPLC traces.

(*S,E*)-2-methyl-*N*-(thiophen-2-ylmethylene)propane-2-sulfinamide (*S*)-13m [24]

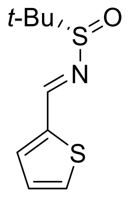

Synthesised from *S*-(*tert*-butyl)-*N*-(thiophen-2-ylmethylene)thiohydroxylamine (40.0 mg, 0.200 mmol) using general procedure **2.4**. Purified by flash column chromatography on silica gel (eluent: hexane : EtOAc = 10:1); black solid (26.0 mg, 60% yield); $[\alpha]_D^{20}$ = 5.06 (c = 1.0, $CHCl_3$); ^{1}H NMR (400 MHz, *Chloroform-d*) δ 8.67 (d, *J* = 1.1 Hz, 1H), 7.58 (dt, *J* = 5.0, 1.1 Hz, 1H), 7.53 (dd, *J* = 3.7, 1.1 Hz, 1H), 7.14 (dd, *J* = 5.0, 3.7 Hz, 1H), 1.24 (s, 9H); ^{13}C NMR (101 MHz, *Chloroform-d*) δ 155.5, 140.6, 133.9, 132.4, 128.2, 58.0, 22.6; HPLC data: 90% *ee*, determined by HPLC (IC column, flow rate: 1.0 mL/min, hexane/propanol: 95:5): tr(minor) = 17.83 min, tr(major) = 16.53 min. See Section 4 for images depicting the HPLC traces.

(*S*)-*N*-((*E*)-((1s,3R)-adamantan-1-yl)methylene)-2-methylpropane-2-sulfinamide ((*S*)-13n) [25]

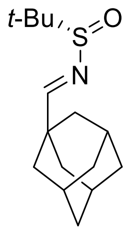

Synthesised from *N*-((*E*)-((3r,5r,7r)-adamantan-1-yl)methylene)-*S*-(*tert*-butyl)thiohydroxylamine (50.0 mg, 0.200 mmol) using general procedure **2.4**. Purified by flash column chromatography on silica gel (eluent: hexane : EtOAc = 10:1); white solid (5.00 mg, 10% yield); $[\alpha]_D^{20}$ = 112.74 (c = 0.25 $CHCl_3$), Lit. $[\alpha]^{20}_D$ = −163.2 (c = 0.5, $CHCl_3$) for *R* isomer; ^{1}H NMR (400 MHz, *Chloroform-d*) δ 7.78 (s, 1H), 2.06 (s, 3H), 1.79 – 1.68 (m, 12H), 1.18 (s, 9H); ^{13}C NMR (101 MHz, *Chloroform-d*) δ 175.2, 56.5, 40.2, 39.2, 36.7, 27.9, 22.4; HPLC data: 80% *ee*, determined by HPLC (IC column, flow rate: 1.0 mL/min, hexane/propanol: 95:5): tr(minor) = 11.22 min, tr(major) = 10.53 min. See Section 4 for images depicting the HPLC traces.

Cinacalcet synthesis [26 – 29]

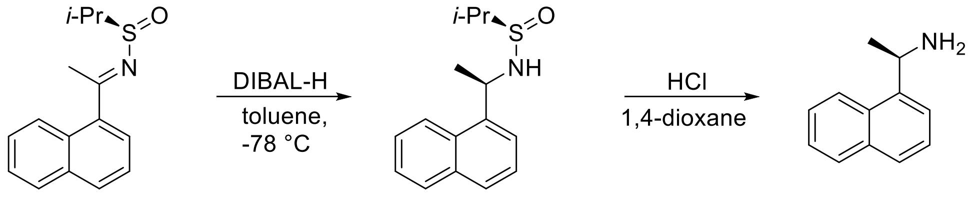

50% yield

14, 98% yield, 99% ee

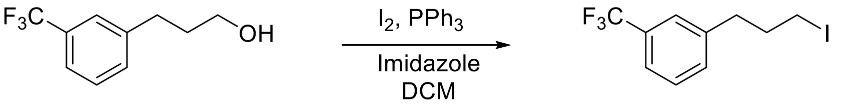

15, 65% yield

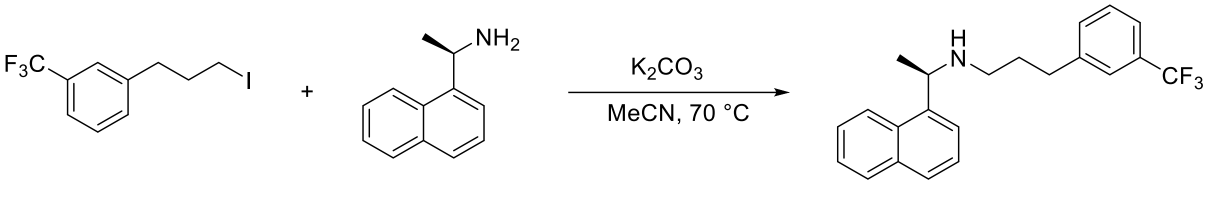

16, 40% yield, **Cinacalcet**

To a flame dried round bottom flask containing a magnetic stirring bar was added ((*R,E*)-*N*-(1-(naphthalen-1-yl)ethylidene)propane-2-sulfinamide (25.0 mg, 0.100 mmol, 1.0 equiv.) in dry toluene (1 mL). The solution was then cool down to –78 °C, which was followed by the addition of 1.0 M DIBAL-H in toluene (0.200 mL, 0.200 mmol, 2.0 equiv.) dropwise and the remaining mixture was stirred at the same temperature for 1 h. Upon completion, the reaction mixture was quenched by adding sat. aq. NH_4Cl (5 mL) and then extracted with ethyl acetate (3 × 5 mL). The combined organic phases were then washed with brine (10 mL), dried over anhydrous $MgSO_4$ and concentrated *in vacuo* to give the crude product mixture, which was purified by flash column chromatography on silica gel (eluent: hexane : EtOAc 10:4) to provide the (*R*)-*N*-((*R*)-1-(naphthalen-1-yl)ethyl)propane-2-sulfinamide (13.0 mg, 50% yield).

To a flame dried round bottom flask containing a magnetic stirring bar was added (*R*)-*N*-((*R*)-1-(naphthalen-1-yl)ethyl)propane-2-sulfinamide (13.0 mg, 0.050 mmol, 1.0 equiv.) in MeOH (50.0 μL). which was followed by the addition of 4.0 M HCl in 1,4-dioxane (25.0 μL, 0.100

mmol, 2.0 equiv.) dropwise and the remain mixture was stirred at the room temperature for 30 min. Upon completion, the reaction was concentrated in *vacuo* and the remain residue was dissolved in water (10 mL) and the solution was washed with Et_2O (10 mL). The aqueous phase was then adjusted to pH 11 by using sat aq. NaOH solution which was then extracted with ethyl acetate (3 × 5 mL) The combined organic phases were then washed with brine (10 mL), dried over anhydrous $MgSO_4$ and concentrated *in vacuo* to give the (*R*)-1-(naphthalen-1-yl)ethan-1-amine **14** (8.4 mg, 98% yield) which was pure enough for next step.

To a flame dried round bottom flask containing a magnetic stirring bar was added 3-(3-(trifluoromethyl)phenyl)propan-1-ol (204 mg, 1.00 mmol, 1.0 equiv.) in DCM (5.00 mL), which was followed by the addition of imidazole (8.20 mg, 0.100 mmol, 0.1 equiv.) and triphenylphosphine (289 mg, 1.10 mmol, 1.1 equiv.). at room temperature. The I_2 (254 mg, 1.00 mmol, 1.0 equiv.) was added portionwise and the mixture was then stirred at room temperature for 1 h. Upon finished, the reaction was quenched by sat $Na_2S_2O_3$ (10 mL) and extracted with DCM (3 × 10 mL). The combined organic phases were then washed with brine (10 mL), dried over anhydrous $MgSO_4$ and concentrated *in vacuo* to give the crude product mixture, which was purified by flash column chromatography on silica gel (eluent: hexane : EtOAc 20: 1) to provide 1-(3-iodopropyl)-3-(trifluoromethyl)benzene **15** (204 mg, 65% yield).

To a small vial containing a magnetic stirring bar was added (*R*)-1-(naphthalen-1-yl)ethan-1-amine (8.40 mg, 0.050 mmol, 1.0 equiv.), K_2CO_3 (8.30 mg, 0.060 mmol, 1.2 equiv.) and 1-(3-iodopropyl)-3-(trifluoromethyl)benzene (20.0 mg, 0.060, 1.2 equiv.) in MeCN (2 mL). The vial was then sealed and the mixture was stirred at 70 °C overnight. Upon completion, the reaction was cool down to room temperature and concentrated *in vacuo* to give the crude product mixture, which was purified by flash column chromatography on silica gel (eluent: hexane : EtOAc 1:1) to provide Cinacalcet **16** as white solid (7.00 mg, 40% yield, 20% yield over three steps); $[\alpha]_D^{20}$ = 13.85 (c = 0.7, $CHCl_3$), Lit. $[\alpha]_D^{20}$ = 20 (c = 1.0, $CHCl_3$)[29]; ^{1}H NMR (400

MHz, *Chloroform-d*) δ 8.23 – 8.11 (m, 1H), 7.88 (dd, *J* = 8.0, 1.6 Hz, 1H), 7.76 (dt, *J* = 8.0, 1.6 Hz, 1H), 7.66 (d, *J* = 7.1 Hz, 1H), 7.55 – 7.29 (m, 7H), 4.64 (q, *J* = 6.6 Hz, 1H), 2.77 – 2.54 (m, 4H), 1.85 (p, *J* = 7.4 Hz, 2H), 1.51 (d, *J* = 6.6 Hz, 3H). The ^{1}H NMR data matched those reported in the literature[29]; ^{13}C NMR (101 MHz, *Chloroform-d*) δ 143.1, 134.1, 131.8, 131.4, 130.8, 130.5, 129.1, 128.7, 127.3, 125.9, 125.8, 125.4, 125.1 (q, *J* = 3.8), 123.0, 122.7 (q, *J* = 3.8), 53.8, 47.3, 33.5, 31.9, 23.7. The **C**F_3 and **C**CF_3 signals were not observed, presumably due to the relatively small sample size and splitting of the signals into quartets; ^{19}F NMR (376 MHz, chloroform-d) δ –62.4.

Large scale synthesis and further transformation [26,27, 30]

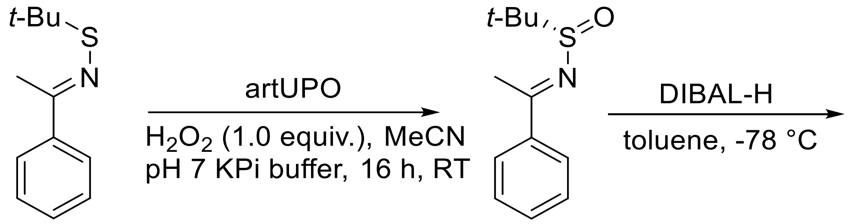

6j-*t*Bu, 3 mmol, 622 mg **(*S*)-13i**, 60% yield, 98% ee

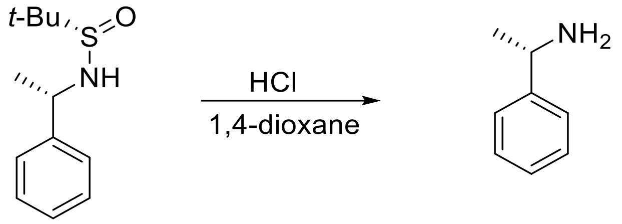

17, 99% yield, dr > 20:1 **18**, 99% yield, precursor for **Mavacamten**

To a round bottom flask containing a magnetic stirring bar was added artUPO 15 mL (final concentration 0.25 g/mL) and KPi buffer (100 mM, pH = 7, 180 mL). The solution was diluted by the addition of deionised water (57 mL), followed by addition of the (*E*)-*S*-(*tert*-butyl)-*N*-(1-phenylethylidene)thiohydroxylamine (622 mg, 3.0 mmol, 1.0 equiv., final concentration 10 mM) in MeCN (72 mL). Next, 36 mL of an 83 mM H_2O_2 solution (prepared from 400 µL 30% H_2O_2 in 36 mL deionised water) was added over a 10 h period, using a syringe pump. After the H_2O_2 addition was complete, the reaction was then stirred at room temperature for a further 6

h. The reaction mixture was then extracted with ethyl acetate (3 × 50 mL). The combined organic phases were then washed with brine (50 mL), dried over anhydrous $MgSO_4$ and concentrated *in vacuo* to give the crude product mixture, which was purified by flash column chromatography on silica gel (eluent: hexane : EtOAc 10:1) to provide the (*S,E*)-2-methyl-*N*-(1-phenylethylidene)propane-2-sulfinamide **(*S*)-13j** (400 mg, 60% yield).

To a flame dried round bottom flask containing a magnetic stirring bar was added (*S,E*)-2-methyl-*N*-(1-phenylethylidene)propane-2-sulfinamide (270 mg, 1 mmol, 1.0 equiv.) in dry toluene (10 mL). The solution was then cool down to −78 °C, which was followed by the addition of 1.0 M DIBAL-H in toluene (2 mL, 2 mmol, 2.0 equiv.) dropwise and the remain mixture was stirred at the same temperature for 1 h. Upon completion, the reaction mixture was quenched by adding sat NH_4Cl (20 mL) and then extracted with ethyl acetate (3 × 20 mL). The combined organic phases were then washed with brine (10 mL), dried over anhydrous $MgSO_4$ and concentrated *in vacuo* to give the crude product mixture, which was purified by flash column chromatography on silica gel (eluent: hexane : EtOAc 10: 4) to provide the (*S*)-2-methyl-*N*-((*S*)-1-phenylethyl)propane-2-sulfinamide **17** (225 mg, 99% yield).

To a flame dried round bottom flask containing a magnetic stirring bar was added (*S*)-2-methyl-*N*-((*S*)-1-phenylethyl)propane-2-sulfinamide (225 mg, 1.0 mmol, 1.0 equiv.) in MeOH (10 mL). which was followed by the addition of 4.0 M HCl in 1,4-dioxane (0.5 mL, 2 mmol, 2.0 equiv.) dropwise and the remain mixture was stirred at the room temperature for 30 min. Upon completion, the reaction was concentrated in *vacuo* and the remain residue was dissolved in water (20 mL) and the solution was washed with EtO_2 (20 mL). The aqueous phase was then adjusted to pH 11 by using sat aq. NaOH solution which was then extracted with ethyl acetate (3 × 20 mL) The combined organic phases were then washed with brine (10 mL), dried over anhydrous $MgSO_4$ and concentrated *in vacuo* to give (*S*)-1-phenylethan-1-amine **18** (107 mg, 99% yield) without any purification as light yellow oil (107 mg, 59% yield over three steps);

$[\alpha]_D^{20}$ = −26.53 (c = 1.0, $CHCl_3$), Lit. $[\alpha]_D^{20}$ = −30.6 (c = 1.0, $CHCl_3$); ^1H NMR (400 MHz, *Chloroform-d*) δ 7.35 – 7.28 (m, 4H), 7.25 – 7.19 (m, 1H), 4.09 (q, *J* = 7.0 Hz, 1H), 1.48 (s, 2H), 1.37 (d, *J* = 6.6 Hz, 3H); ^{13}C NMR (101 MHz, *Chloroform-d*) δ 147.9, 128.6, 126.9, 125.8, 51.4, 25.8.

HPLC Data: >99 % *ee* determined by HPLC (IBN5, flow rate: 1.0 mL/ min, hexane/ isopropanol = 95:5) tr (minor) = 12.51 min, tr (major) = 10.99 min. See Section 4.0 for images depicting the HPLC trace.

4) Enzyme production

Production scale fermentations of artUPO and r*Aae*UPO-PaDa-I-H were carried out in a 7 L non-jacketed glass vessel cultivated on an ADI1010 Bio-controller and monitored using BioXpert V.2 (Applikon). A 7 L bioreactor was charged with 3 L of a basal salts medium containing 26.7 mL^{-1} H_3PO_4, 85 % (w/v); 1.17 g L^{-1} $CaSO_4.2H_2O$; 18.2 g L^{-1} K_2SO_4; 14.9 g L^{-1} $MgSO_4.7H_2O$; 4.13 g L^{-1} KOH; 40.0 g L^{-1} glycerol) and sterilised at 121 ºC for 30 min. Subsequently, 4.35 mL L^{-1} of filter sterilised PTM_1 trace salts (containing 6.0 g L^{-1} $CuSO_4.5H_2O$; 0.08 g L^{-1} NaI; 3.0 g L^{-1} $MnSO_4.H_2O$; 0.2 g L^{-1} $Na_2MoO_4.2H_2O$; 0.02 g L^{-1} H_3BO_3; 0.5 g L^{-1} $CoCl_2$; 20.0 g L^{-1} $ZnCl_2$; 65 g L^{-1} $FeSO_4.7H_2O$; 0.2 g L^{-1} biotin; 5.0 mL L^{-1} H_2SO_4) was added. The following parameters were set for the fermentation: temperature at 30 ºC, pH 5.0, airflow at 2 L min^{-1}, dissolved oxygen (DO) at 30% relative to air saturation and maintained by stirrer limits set to 350 - 1000 rpm plus O_2 supplementation at 0.6 L min^{-1}. The pH was adjusted to 5.0 using 28 % ammonium hydroxide solution.

Recombinant *Pichia pastoris* X-33 strains expressing artUPO and r*Aae*UPO-PaDa-I-H were generated as described previously.[31,32] Gene sequences for the UPOs are shown in Table S1. A glycerol stock of the artUPO/ r*Aae*UPO-PaDa-I-H recombinant *P. pastoris* strain was streaked onto a Yeast extract Peptone Dextrose (YPD) plate with 100 µg mL^{-1} zeocin and incubated at 30 °C for 3 d. A single colony was selected and grown in 5 mL Buffered Glycerol-complex Medium (BMGY) containing 1% yeast extract; 2% peptone; 100 mM potassium phosphate pH 6.0; 1.34 % yeast nitrogen base; 4 x 10^{-5} % biotin and 1 % glycerol for 24 h at 30 °C with shaking at 220 rpm. The 5 mL starter culture was used to further inoculate 300 mL of BMGY at a dilution of 1:150 and cultivated in baffled flasks at 30 °C, 200 rpm for a further 20 h. The 300 mL starter culture was directly inoculated into the conditioned fermentation vessel, and the culture grown until all the glycerol had been consumed (determined by DO spike). The cell biomass was further increased by a fed-batch phase on 50% (w/v) glycerol containing 12 mL L^{-1} of filter sterilised PTM_1 trace salts, at a feed rate of 18 mL h^{-1} L^{-1} of initial fermentation volume for 4 h. To induce expression of UPO, a 100 % methanol feed with PTM_1 salts (12 mL L^{-1}) was then added at 2.0 mL h^{-1} L^{-1} of initial fermentation volume for 18 h (artUPO) or 3 h (r*Aae*UPO-PaDa-I-H). When the culture had adapted to the methanol feed rate, had a steady DO and a fast DO spike after stopping the methanol addition, the methanol feed rate was increased to 3.6 mL h^{-1} L^{-1} of initial fermentation volume for 48 h (artUPO) or 18 h (r*Aae*UPO-PaDa-I-H). For artUPO this was further increased to 4.8 mL h^{-1} L^{-1} for 24 h.

The remaining cultivation was then performed at a final feed rate of 6.0 mL h^{-1} L^{-1} of initial fermentation volume for both UPOs. The culture was harvested at 140 h (artUPO) or 76 h (r*Aae*UPO-PaDa-I-H) from the start of methanol addition and centrifuged at 5,000 × *g* for 20 min to remove the cells, with the UPO located in the secretate.

The secretate was clarified by centrifugation at 10,000 × *g* for 20 min and concentrated approximately 10-fold by Tangential Flow Filtration (TFF) using a KrosFlo Research IIi TFF system with a Repligen MiniKros hollow fibre filter (PN: S06-E010-05-N; mPES membrane, 10 kDa MWCO, 2600 cm^2 surface area) at 4 ºC. The feed flow rate was maintained at 1 L min^{-1}, and the transmembrane pressure set to 15 psi. The concentrated liquid UPO secretate was stored at −70 ºC and used crude in all reactions described.

Table S1: artUPO and r*Aae*UPO-PaDa-I-H gene sequences.

artUPO	TCCCAGGATATTGTGGACTTCTCCCAGCATCCGTGGAAGGCACCTGGAC<br>CCAACGATCTCCGCTCCCCCTGTCCCGGACTCAACACGCTCGCGAACCA<br>CGGCTTCCTCCCTCGCAACGGTCGAAACATCACGATCCCGATGATCGTC<br>CAGGCAGGCTTCGACGGCTACAACGTCCAGCCGGATATCCTCATTTTGG<br>CAGCCAAGGTGGGATTGCTCACCTCGCCTGAACCCGATACCTTCACCCT<br>CGACGATTTGAAGTTGCATGGAACCATCGAGCATGATGCATCGTTGTC<br>GAGGGAGGACTTCGCGCTCGGCGATAACCTCCACTTCAACGAGGCGAT<br>CTTCAACACCCTCGCAAACTCGAACCCTGGTTCCGATGTCTACAACATC<br>ACCTCGGCAGGACAGGTGCTCAAGGACCGCCTCGCCGACTCGCTCGCC<br>AGGAACCCGAACGTCACGAACACCGGCAAAGAGTTCACTATCAGGACT<br>TTGGAGTCCGCCTTCTATCTCTCCGTCATGGGCAACGCAACAACAGGTG<br>AAGCACCCAAAAACTTCGTGCAGATTTTCTTCCGAGAGGAGCGGCTCC<br>CCATCGAGGAGGGCTGGAAGAGGTCCACAACCCCTATCACGTCGGACA<br>CGTTGAACCCCATTGCAGGCCAGATTTCGGAGGCGTCGAACTGGAAGC<br>CCAACCCCGACCAGTGTCCCTGGATCGTCCTCTCGCCCAACTTG
r*Aae*UPO-PaDa-I-H	CCAGGATTACCTCCTGGTCCTCTCGAGAATAGCTCTGCAAAGTTGGTGA<br>ACGACGAGGCTCACCCATGGAAGCCGCTTCGACCTGGCGATATTCGTG<br>GACCTTGCCCTGGTCTCAATACTCTGGCATCTCACGGGTACCTCCCGAG<br>AAATGGCGTTGCAACCCCGGCGCAAATAATAAACGCGGTTCAGGAAGG<br>ATTCAATTTCGACAATCAAGCCGCAATCTTCGCCACATATGCGGCCCAC

	CTTGTGGACGGCAATCTCATTACGGACTTGCTGAGCATCGGACGCAAG ACGCGGCTCACTGGGCCTGATCCACCACCCCCGCTTCCGTTGGTGGAC TCAATGAGCATGGCACCTTCGAAGGCGACGCCAGTATGACCCGAGGTG ACGCATTCTTTGGCAACAACCACGATTTCAATGAGACGCTCTTCGAACA GTTGGTTGACTACAGCAACCGATTTGGAGGAGGAAAATACAATCTTAC CGTCGCGGGGGAGCTCCGTTTCAAGCGCATTCAAGACTCCATTGCGAC CAACCCCAATTTCTCCTTTGTTGACTTTAGGTTCTTTACTGCTTACGGCG AGACCACCTTCCCCGCGAATCTTTTTGTGGATGGGCGCAGGGACGACG GCCAGCTAGATATGGATGCTGCACGGAGTTTTTTCCAATTCAGCCGTAT GCCTGACGATTTCTTCCGCGCACCCAGCCCGAGAAGTGGCACAGGAGT CGAGGTAGTTGTACAGGCTCATCCTATGCAGCCCGGAAGAAATGTCGG CAAGATCAACAGCTACACCGTCGACCCAACATCCTCTGACTTTTCCACC CCCTGCTTGATGTACGAGAAATTCGTCAACATAACGGTCAAGTCACTCT ACCCGAATCCGACGGTGCAGCTTCGCAAAGCCCTTAATACGAATCTCG ATTTCTTATTCCAGGGAGTCGCCGCTGGATGTACCCAGGTCTTCCCATA CGGGCGAGAT

Production of *Cma*UPO and *Dca*UPO

Cloning & generation of recombinant strains

Genes encoding *Cma*UPO and *Dca*UPO were codon optimised for expression in *Pichia pastoris* and synthesised by Twist Bioscience. The genes were amplified from their carrier plasmid by PCR and inserted by In Fusion cloning into the pPICZα B vector (Invitrogen), adapted to include an N-terminal His_6-tag downstream of the alpha mating factor (MFα) secretion signal sequence. The genes were designed not to include the native *Cma*UPO/*Dca*UPO signal peptides, in order for the expressed UPOs to be secreted under the influence of the MFα secretion signal. Gene sequences are shown in **Table S2**.

Table S2: *Cma*UPO and *Dca*UPO gene sequences.

*Cma*UPO	ATTCCCCAATACGCTACATTAGCCGGATTAACCGAGAGACAGTTGGCA GAAGTTGTGCCCCAAATGAACGCCAAGATCCCACCTGCTCCTCCTGGTC CATTGCGATTTGGTGGTTTAAAATTGGTAGACGACAGAGATCACCCTTG GAGACCCTTGCGAGATGGTGACATCAGGGGTCCTTGCCCTGGTTTGAA

	CACCCTGGCAAGTCATGGTTATCTGCCGAGGGATGGTGTCGCTACTCCT ACACAGATTATAAACGCTGCCCAGGAAGGACTAAACTTAATTAATCAG GGTGCTAAGTTGGCTACATATGCTGCCCTCTTGTTAGAAGGCAATGTTG TTACTAACTTACTTTCAATAGGTGGTAAGACACATCGTACTGGTCCTGA TCCACCATCACCCGCAAGTGTTGGCGGTTTGTCGCACCATGGAACGTTC GAGGGTGATGCTTCTACCACAAGGGGTGATGCATTTTTTGGTGACAATC ACTCTTTCAACCAAACATTATTTGACCAGTTTGTTGATTTTTCTAATCGT TTTGGCAATGGATATTATAACTATACGGTTGGAGGTGAGTTAAGGTTTC ACAGAATTCAAGATTCAATTGCTACCAATCCAGAGTTTGACCTAAGGG GCTTCAGACACTCTACCGCTTTTGGAGAGTCTGCCTTTATTGCAAACAA TTTTGTGGACGGCCGTAAAACTGGCGCTGAGGCACATCAACTAGATAT GGATTCTGCCTTATCTTTCTTCCGAGACATGCGATTCCCCAGAGGTTTTC ACAGAGCTGCACAGCCTGGTGGAGGAGAAGGAGTTGACGTTATCTTTA ATGCTCATCCCCTGCAACCAGGACATAACGTAGGCGGAGTTAATAATT ACGTTGTTGATACTTCCAGGGGTTCACTCTTTGATCAGTGTGGGATTTA TACCTACATGGTGAATACTACTATTAGGGATCTGTATCCAAATCCAACC GGTGTTCTAAGACGTAACTTGAACATTAACCTTGATTTCCTCTACGAAG CTTTCGGTTTCACTTCTGCAGATTGCCCACAAGTTAGGCCGTTCGGTAG GAAC
*Dca*UPO	GCGCCATGGAAAGCGCCCGGTCCAGACGACGTTCGAGGACCCTGTCCT ATGCTTAATACCTTGGCTAATCACGGGTTTCTTCCGCATGATGGGAAGA ACATCGATGTTAACACAACCGTCAATGCGCTCTCAAGTGCCTTAAACTT AGATGACGAGCTATCGAGAGATCTCCACACATTTGCCGTAACTACAAA CCCCCAACCGAATGCCACATGGTTCTCGCTGAATCACCTCTCTAGACAT AATGTGTTAGAACATGACGCCTCGTTGTCTCGGCAAGACGCCTATTTCG GCCCGCCGGATGTCTTTAACGCCGCAGTCTTCAACGAGACGAAAGCAT ACTGGACTGGCGACATAATTAATTTTCAGATGGCGGCTAACGCTCTCAC GGCACGTCTTATGACAAGCAATCTAACCAACCCCGAATTCTCAATGTCC CAATTAGGACGTGGATTCGGGCTGGGCGAGACTGTAGCTTACGTAACT ATCCTGGGTAGCAAGGAGACGCGGACCGTGCCAAAAGCGTTTGTCGAA TATTTGTTCGAGAACGAGAGGTTGCCTTATGAATTGGGTTTCAAGAAAA TGAAATCTGCGCTAACGGAAGATGAATTAACTACGATGATGGGAGAAA TTTACTCACTTCAGCATTTACCGGAATCCTTTACGAAGCCTTTTGCGAA

	ACGCAGTGAAGCCCCTTTTGAAAAGCGAGCAGAGAAGAGGTGCCCATT TCAC

The recombinant *Cma*UPO/*Dca*UPO plasmids were used to transform Stellar™ Competent *E. coli* cells (Takara Bio) and plated overnight at 37 °C on low-salt LB agar plates containing 25 µg mL^{-1} zeocin. Transformant colonies were used to inoculate 10 mL starter cultures of low-salt LB with 25 µg mL^{-1} zeocin and grown overnight at 37 °C with shaking at 180 rpm. Recombinant plasmid DNA was extracted and purified using the QIAGEN plasmid *Plus* Midi Kit, and *Cma*UPO/*Dca*UPO sequences were confirmed by Sanger sequencing (Source BioScience).

Purified plasmid DNA was linearised with *PmeI* restriction enzyme and used to transform *P. pastoris* strain X-33 (Invitrogen) *via* electroporation. The transformed cells were plated on a Yeast extract Peptone Dextrose plate with 100 µg mL^{-1} zeocin (YPDZ) and incubated at 30 °C for 3 d. Colonies were streaked onto fresh YPDZ plates and incubated for a further 3 d at 30 °C. Clean single colonies were then used to inoculate 5 mL Buffered Glycerol-complex Medium (BMGY) containing 1% yeast extract; 2% peptone; 100 mM potassium phosphate pH 6.0; 1.34% (w/v) yeast nitrogen base; 4×10^{-5} % biotin and 1% glycerol, and grown for 24 h at 30 °C with shaking at 220 rpm. Glycerol stocks for the transformed *Cma*UPO/*Dca*UPO *P. pastoris* strains were prepared using a 1:1 mixture of BMGY culture with 50 % (v/v) sterile glycerol and stored at −70 °C.

Fermentation & UPO production

A 0.5 L MiniBio fermenter (Applikon) was charged with 200 mL of a basal salts medium containing 26.7 mL L^{-1} H_3PO_4, 85 % (w/v); 1.17 g L^{-1} $CaSO_4\cdot2H_2O$; 18.2 g L^{-1} K_2SO_4; 14.9 g L^{-1} $MgSO_4\cdot7H_2O$; 4.13 g L^{-1} KOH; 40.0 g L^{-1} glycerol, and 4.35 mL L^{-1} of PTM_1 trace salts containing 6.0 g L^{-1} $CuSO_4\cdot5H_2O$; 0.08 g L^{-1} NaI; 3.0 g L^{-1} $MnSO_4\cdot H_2O$; 0.2 g L^{-1} $Na_2MoO_4\cdot2H_2O$; 0.02 g L^{-1} H_3BO_3; 0.5 g L^{-1} $CoCl_2$; 20.0 g L^{-1} $ZnCl_2$; 65 g L^{-1} $FeSO_4\cdot7H_2O$; 0.2 g L^{-1} biotin; 5.0 mL L^{-1} H_2SO_4). The following parameters were set for the fermentation: temperature at 30 °C, condenser at 70 %, pH at 5.0, base pump output at 25 %, airflow at 200 mL min^{-1}, dissolved oxygen (DO) at 35 % relative to air saturation and maintained by stirrer limits set to 500–1250 rpm plus O_2 supplementation. The pH was adjusted to 5.0 using 28% ammonium hydroxide solution.

A glycerol stock of the *Cma*UPO/*Dca*UPO *P. pastoris* strain was streaked onto a Yeast extract Peptone Dextrose (YPD) plate with 100 µg mL^{-1} zeocin and incubated at 30 °C for 3 d. Two colonies were selected and grown in 2×10 mL Buffered Glycerol-complex Medium (BMGY) containing 1 % yeast extract; 2 % peptone; 100 mM potassium phosphate pH 6.0; 1.34% (w/v) yeast nitrogen base; 4×10^{-5} % biotin and 1 % glycerol for 24 h at 30 °C with shaking at 220 rpm. The starter cultures were pooled and added to the conditioned fermentation vessel, and the culture grown until all the glycerol had been consumed (determined by DO spike; approximately 20 h). The cell biomass was further increased by a fed-batch phase on 50 % (w/v) glycerol containing 12 mL L^{-1} of filter sterilised PTM_1 trace salts, at a feed rate of 18 mL h^{-1} L^{-1} of initial fermentation volume for 4 h. To induce expression of UPO, a 100% methanol feed with PTM_1 salts (12 mL L^{-1}) was then added at 3.6 mL h^{-1} L^{-1} of initial fermentation volume for 3 h, before increasing to 4.2 mL h^{-1} L^{-1} for 24 h. When the culture had adapted to the methanol feed rate, had a steady DO % and a fast DO spike after stopping methanol addition, the feed rate was increased to 7.2 mL h^{-1} L^{-1} of initial fermentation volume for 24 h, and further increased to 7.8 mL h^{-1} L^{-1} for the remainder of the cultivation. The cultures were harvested at 120 h from the start of methanol addition and centrifuged at 5,000 × g for 20 min to remove the cells, with the UPO located in the secretate. The secretate was further clarified at 10,000 × g for 20 min and concentrated approximately 8-fold by spin concentration at 4000 × g (Pierce Protein Concentrator PES, 10K MWCO). The concentrated liquid UPO secretate was stored at −70 ºC and used crude in all reactions described.

Enzyme activity

Peroxidase and peroxygenase activities of the artUPO and r*Aae*UPO-PaDa-I-H concentrated liquid secretates were determined by UV assays with the substrates 2,2'-azino-bis(3-ethylbenzothiazoline-6-sulfonic acid) (ABTS) and peroxygenase 1,2-(methylenedioxy-4-nitrobenzene) (NBD), respectively, as described in detail previously.[32]

5) HPLC Data

LC DATA FILE

6 Mar 2025

File Name	\\storage.york.ac.uk\chemistry\services\Chromatography\CDCSHPLCAG1100\20250217_BM959_PHME_65_35_ASH 2025-02-17 19-19-33\SI_REC.D\dad1C.ch
Title	LC DATA FILE
Sampling Rate	150

Sample Name SI_REC

Detector UV (210 nm)

dad1C.ch

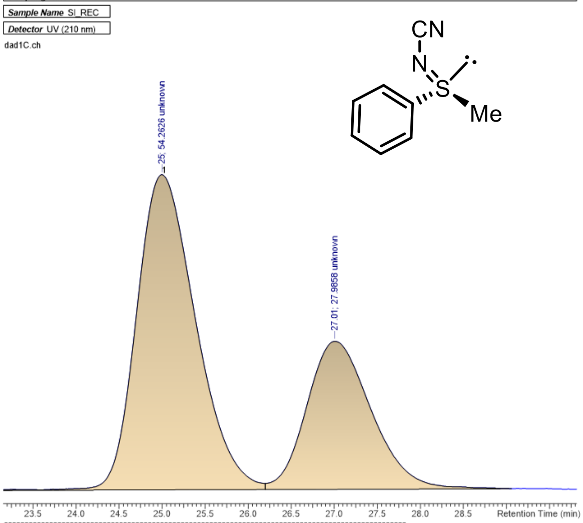

No.	Name	tR	Peak Area (Y units*ms)	Area (%)	Width
1	unknown	25.00	3255753.250	65.974	1.250
2	unknown	27.01	1679147.530	34.026	1.364

LC DATA FILE

6 Mar 2025

File Name	\\storage.york.ac.uk\chemistry\services\Chromatography\CDCSHPLCAG1100\20250217_BM959_PHME_65_35_ASH 2025-02-17 19-19-33\SI_STD.D\dad1C.ch
Title	LC DATA FILE
Sampling Rate	150

Sample Name SI_STD

Detector UV (210 nm)

dad1C.ch

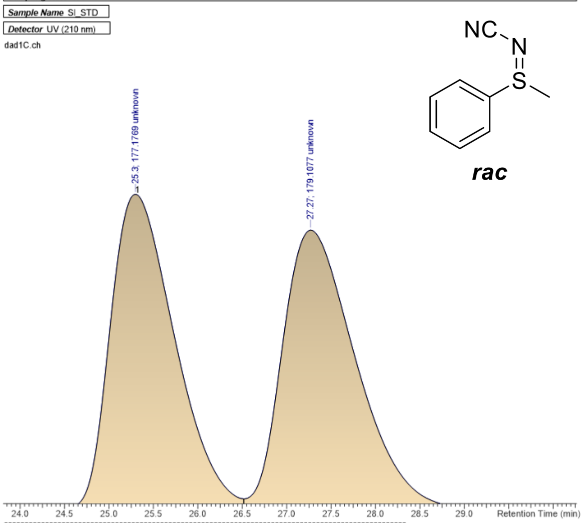

No.	Name	tR	Peak Area (Y units*ms)	Area (%)	Width
1	unknown	25.30	10630615.540	49.729	1.343
2	unknown	27.27	10746461.792	50.271	1.516

Figure S4. HPLC trace for ***(S)*-4a**

LC DATA FILE

7 Mar 2025

File Name	\\storage.york.ac.uk\chemistry\services\Chromatography\CDCSHPLCAG1100\20250217_BM959_PHME_65_35_ASH 2025-02-17 19-19-33\SI_REC(CONC).D\dad1C.ch
Title	LC DATA FILE
Sampling Rate	150

Sample Name SI_REC(conc)

Detector UV (210 nm)

dad1C.ch

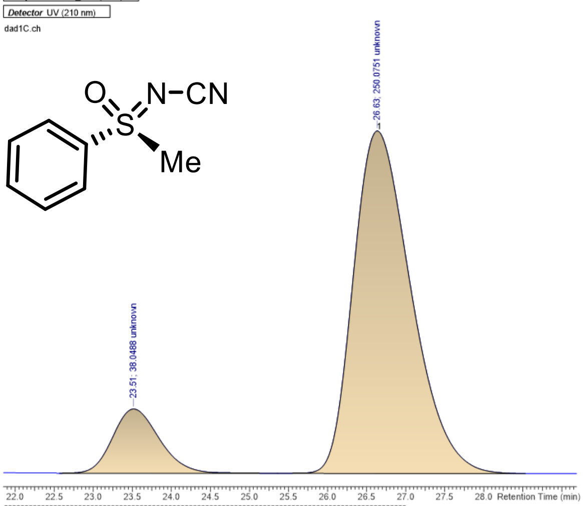

No.	Name	tR	Peak Area (Y units*ms)	Area (%)	Width
1	unknown	23.51	2282929.687	13.206	1.089
2	unknown	26.63	15004507.141	86.794	1.356

LC DATA FILE

7 Mar 2025

File Name	\\storage.york.ac.uk\chemistry\services\Chromatography\CDCSHPLCAG1100\20250217_BM959_PHME_65_35_ASH 2025-02-17 19-19-33\SO_STD.D\dad1C.ch
Title	LC DATA FILE
Sampling Rate	150

Sample Name SO_STD

Detector UV (210 nm)

dad1C.ch

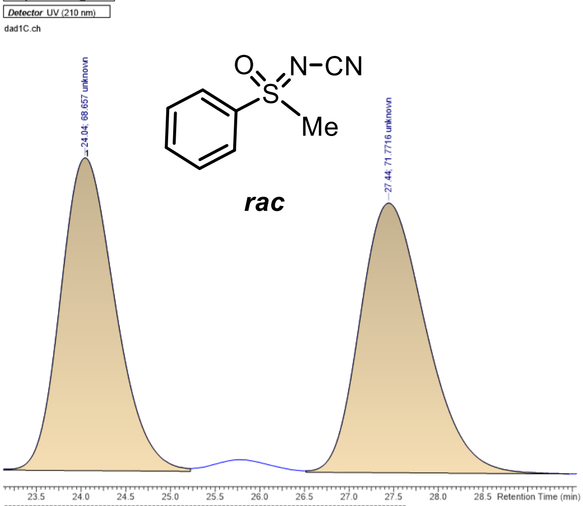

No.	Name	tR	Peak Area (Y units*ms)	Area (%)	Width
1	unknown	24.04	4119418.488	48.891	1.114
2	unknown	27.44	4306293.640	51.109	1.353

Figure S5. HPLC trace for ***(S)*-5a**

LC DATA FILE

21 Mar 2025

File Name \\storage.york.ac.uk\chemistry\services\Chromatography\CDCSPAC\Data\250321_BM959_4MEO(1)_ADH8317 2025-03-21 18-30-15\RED.D\dad1A.ch
Title LC DATA FILE
Sample Name red
Detector UV (270 nm)
UV Trace Bandwidth 4 nm

dad1A.ch

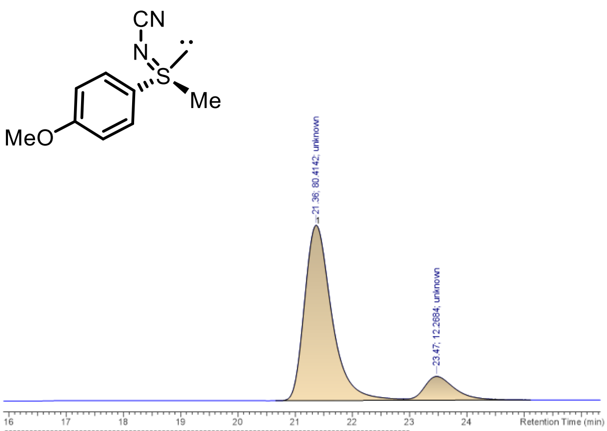

No.	Name	tR	Peak Area (Y units*ms)	Area (%)	Width
1	unknown	21.36	4824854.736	86.763	0.829
2	unknown	23.47	736102.295	13.237	0.926

LC DATA FILE

21 Mar 2025

File Name \\storage.york.ac.uk\chemistry\services\Chromatography\CDCSPAC\Data\250321_BM959_4MEO_ADH8317 2025-03-21 17-28-13\4MEO STD.D\dad1A.ch
Title LC DATA FILE
Sampling Rate 150
Sample Name 4MeO STD
Detector UV (270 nm)

dad1A.ch

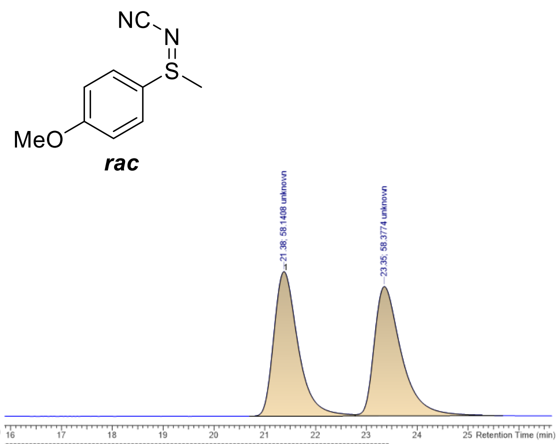

No.	Name	tR	Peak Area (Y units*ms)	Area (%)	Width
1	unknown	21.38	3488445.282	49.898	0.833
2	unknown	23.35	3502645.798	50.102	0.919

Figure S6. HPLC Data for **(*S*)-4b**

LC DATA FILE

21 Mar 2025

File Name \\storage.york.ac.uk\chemistry\services\Chromatography\CDCSHPLCAG1100\20250225_BM959_ADH_80_20_4MEOSCALEUPSO_ 2025-02-25 22-06-05\SULFOXIMINE_RECOV.D\dad1A.ch
Title LC DATA FILE
Sample Name Sulfoximine_recov
Detector UV (254 nm)
UV Trace Bandwidth 4 nm

dad1A.ch

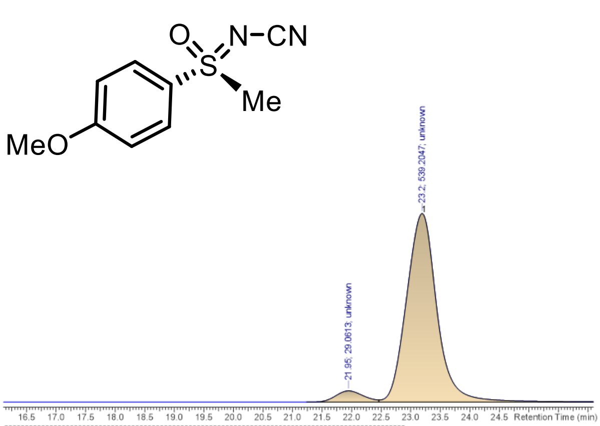

No.	Name	tR	Peak Area (Y units*ms)	Area (%)	Width
1	unknown	21.95	1743679.250	5.114	0.873
2	unknown	23.20	32352282.000	94.886	0.903

LC DATA FILE

21 Mar 2025

File Name \\storage.york.ac.uk\chemistry\services\Chromatography\CDCSHPLCAG1100\20250307_BSM_4MEOSTD_ADH_8020_1FR 2025-03-08 19-21-42\4MEOSTD(2).D\dad1A.ch
Title LC DATA FILE
Sample Name 4MEOSTD
Detector UV (254 nm)
UV Trace Bandwidth 4 nm

dad1A.ch

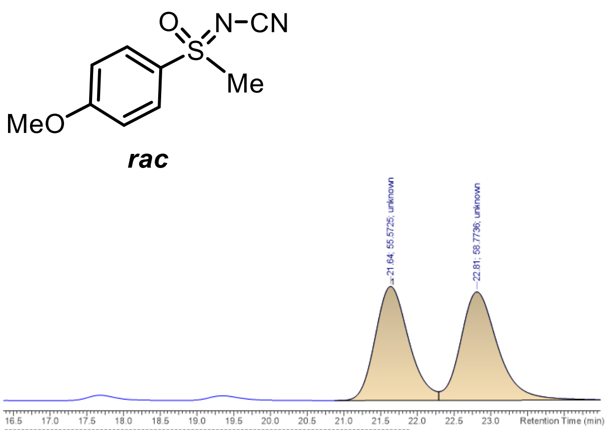

No.	Name	tR	Peak Area (Y units*ms)	Area (%)	Width
1	unknown	21.64	3334347.153	48.600	0.826
2	unknown	22.81	3526417.236	51.400	0.884

Figure S7. HPLC Data for **(*S*)-5b**

LC DATA FILE

9 Mar 2025

File Name	\\storage.york.ac.uk\chemistry\services\Chromatography\CDCSHPLCAG1100\20250307_BSM_4MEOSTD_ADH_8020_1FR 2025-03-09 15-55-48\3MEOREC.D\dad1A.ch
Title	LC DATA FILE
Sampling Rate	150

Sample Name 3MEOREC

Detector UV (254 nm)

dad1A.ch

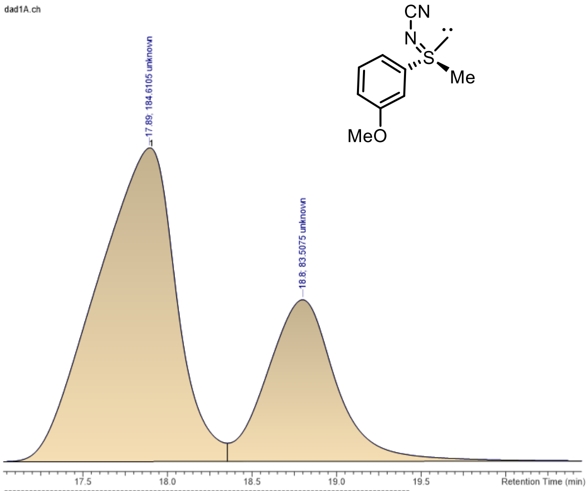

No.	Name	tR	Peak Area (Y units*ms)	Area (%)	Width
1	unknown	17.89	11076628.418	68.854	0.878
2	unknown	18.80	5010449.982	31.146	0.712

LC DATA FILE

9 Mar 2025

File Name	\\storage.york.ac.uk\chemistry\services\Chromatography\CDCSHPLCAG1100\20250307_BSM_4MEOSTD_ADH_8020_1FR 2025-03-09 15-55-48\3MEOSTD.D\dad1A.ch
Title	LC DATA FILE
Sampling Rate	150

Sample Name 3MEOSTD

Detector UV (254 nm)

dad1A.ch

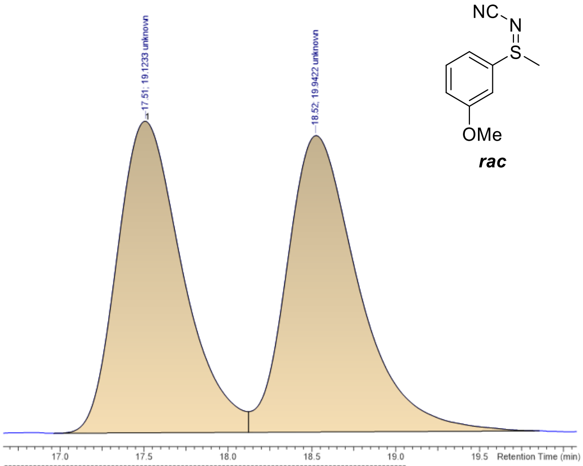

No.	Name	tR	Peak Area (Y units*ms)	Area (%)	Width
1	unknown	17.51	1147395.401	48.952	0.703
2	unknown	18.52	1196533.813	51.048	0.745

Figure S8. HPLC Data for **(*S*)-4c**

LC DATA FILE

9 Mar 2025

File Name	\\storage.york.ac.uk\chemistry\services\Chromatography\CDCSHPLCAG1100\20250217_BM959_ADH_8020_3MEO_RERUN STD 2025-02-20 19-56-48\SO_REC.D\dad1C.ch
Title	LC DATA FILE
Sampling Rate	150

Sample Name SO_REC

Detector UV (210 nm)

dad1C.ch

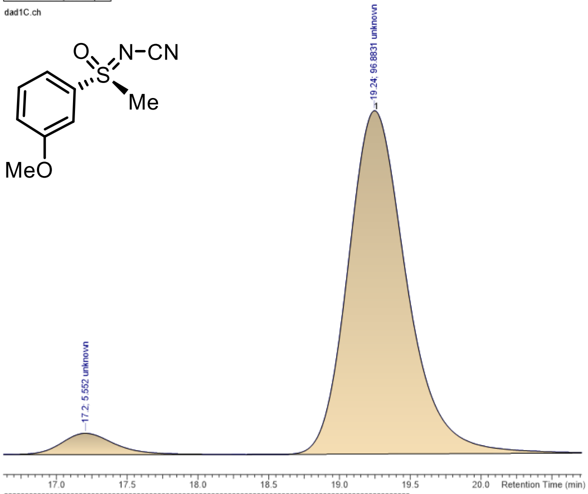

No.	Name	tR	Peak Area (Y units*ms)	Area (%)	Width
1	unknown	17.20	333121.281	5.420	0.661
2	unknown	19.24	5812983.000	94.580	0.729

LC DATA FILE

10 Mar 2025

File Name	\\storage.york.ac.uk\chemistry\services\Chromatography\CDCSHPLCAG1100\20250307_BSM_3MEOSTDSO_ADH_8020_1FR 2025-03-09 20-45-43\3MEOSTD.D\dad1C.ch
Title	LC DATA FILE
Sampling Rate	150

Sample Name 3MEOSTD

Detector UV (210 nm)

dad1C.ch

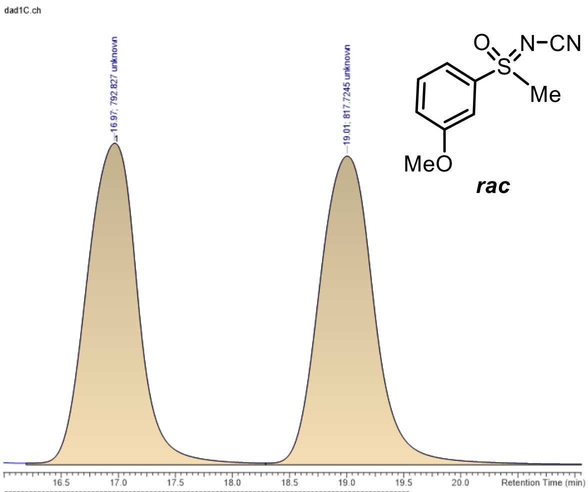

No.	Name	tR	Peak Area (Y units*ms)	Area (%)	Width
1	unknown	16.97	47569617.920	49.227	0.807
2	unknown	19.01	49063469.238	50.773	0.860

Figure S9. HPLC Data for **(*S*)-5c**

LC DATA FILE

10 Mar 2025

File Name	\\storage.york.ac.uk\chemistry\services\Chromatography\CDCSPAC\Data\20240701-BM959_HEX90_ADH_4CLSORAC 2024-07-01 17-50-39\4_CL_SI_ART.D\dad1A.ch
Title	LC DATA FILE
Sampling Rate	150

Sample Name 4_Cl_SI_art

Detector UV (254 nm)

dad1A.ch

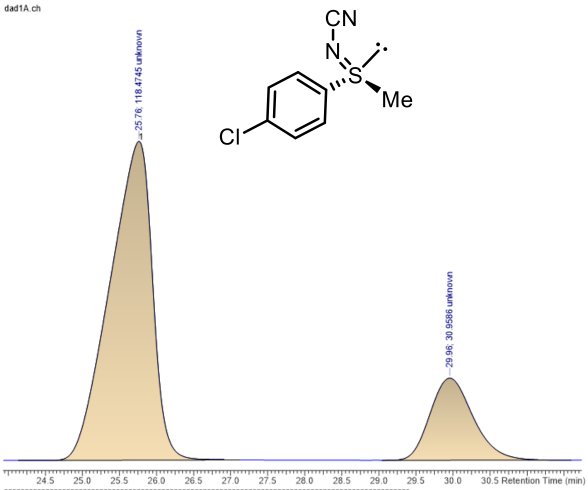

No.	Name	tR	Peak Area (Y units*ms)	Area (%)	Width
1	unknown	25.76	7108470.154	79.283	1.074
2	unknown	29.96	1857514.229	20.717	1.056

LC DATA FILE

10 Mar 2025

File Name	\\storage.york.ac.uk\chemistry\services\Chromatography\CDCSPAC\Data\20240701-BM959_HEX90_ADH_4CLSORAC 2024-07-01 17-50-39\4_CL_SI_STD.D\dad1A.ch
Title	LC DATA FILE
Sampling Rate	150

Sample Name 4_Cl_SI_std

Detector UV (254 nm)

dad1A.ch

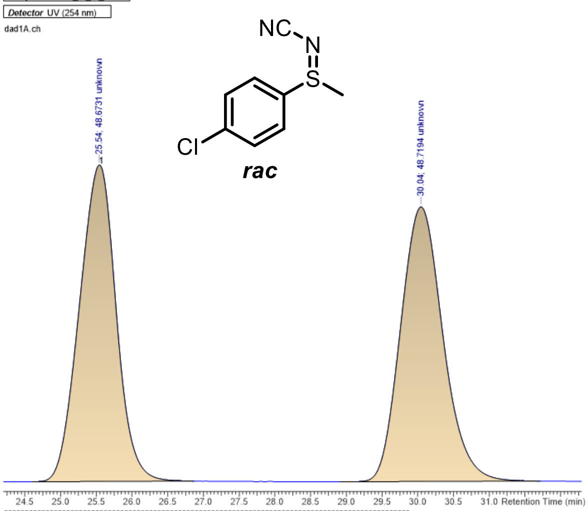

No.	Name	tR	Peak Area (Y units*ms)	Area (%)	Width
1	unknown	25.54	2920383.911	49.976	0.942
2	unknown	30.04	2923161.850	50.024	1.079

Figure S10. HPLC Data for **(*S*)-4d**

LC DATA FILE

10 Mar 2025

File Name	\\storage.york.ac.uk\chemistry\services\Chromatography\CDCSPAC\Data\20240701-BM959_HEX90_ADH_4CLSOART 2024-07-01 12-56-06\4_CL_SO_ART.D\dad1A.ch
Title	LC DATA FILE
Sampling Rate	150

Sample Name 4_Cl_SO_art

Detector UV (254 nm)

dad1A.ch

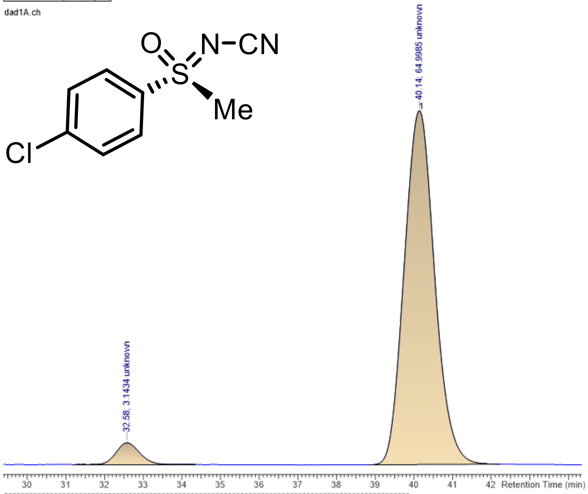

No.	Name	tR	Peak Area (Y units*ms)	Area (%)	Width
1	unknown	32.58	188601.109	4.613	1.117
2	unknown	40.14	3899912.250	95.387	1.436

LC DATA FILE

10 Mar 2025

File Name	\\storage.york.ac.uk\chemistry\services\Chromatography\CDCSPAC\Data\20240701-BM959_HEX90_ADH_4CLSORAC 2024-07-01 17-50-39\4_CL_SO_RAC_1.D\dad1A.ch
Title	LC DATA FILE
Sampling Rate	150

Sample Name 4_Cl_SO_rac

Detector UV (254 nm)

dad1A.ch

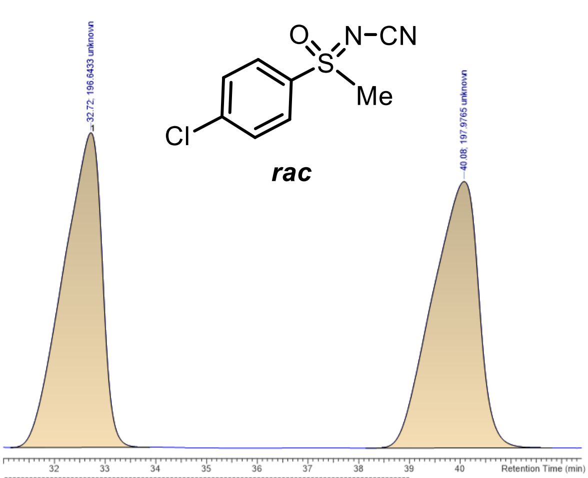

No.	Name	tR	Peak Area (Y units*ms)	Area (%)	Width
1	unknown	32.72	11798600.464	49.831	1.457
2	unknown	40.08	11878592.834	50.169	1.741

Figure S11. HPLC Data for **(*S*)-5d**

LC DATA FILE

19 Mar 2025

File Name	\\storage.york.ac.uk\chemistry\services\Chromatography\CDCSPAC\Data\250314_BM959_4CF3SM_STD_PAP_ADH8218 2025-03-17 11-40-43\SM_SCALEUP_1.D\dad1A.ch
Title	LC DATA FILE
Sampling Rate	150

Sample Name SM_SCALEUP_1

Detector UV (254 nm)

dad1A.ch

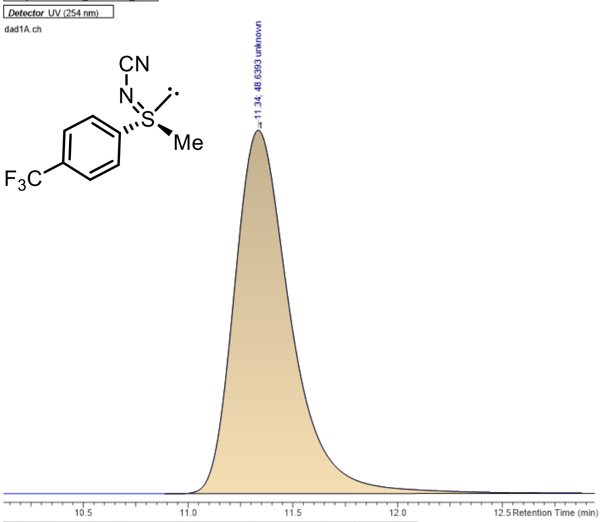

No.	Name	tR	Peak Area (Y units*ms)	Area (%)	Width
1	unknown	11.34	2918359.222	100.000	0.466

LC DATA FILE

19 Mar 2025

File Name	\\storage.york.ac.uk\chemistry\services\Chromatography\CDCSPAC\Data\250314_BM959_4CF3SM_STD_PAP_ADH8218 2025-03-17 11-40-43\SM_STD.D\dad1A.ch
Title	LC DATA FILE
Sampling Rate	150

Sample Name SM_STD

Detector UV (254 nm)

dad1A.ch

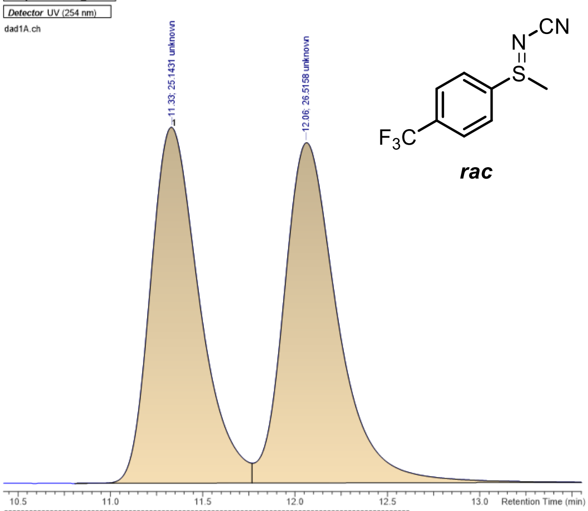

No.	Name	tR	Peak Area (Y units*ms)	Area (%)	Width
1	unknown	11.33	1508585.243	48.671	0.473
2	unknown	12.06	1590949.516	51.329	0.500

Figure S12. HPLC Data for **(*S*)-4e**

LC DATA FILE

10 Mar 2025

File Name	\\storage.york.ac.uk\chemistry\services\Chromatography\CDCSHPLCAG1100\20250217_BM959_ADH_8020_4CF3_2NDSCALKE 2025-02-21 10-13-23\SO_REC.D\dad1A.ch
Title	LC DATA FILE
Sampling Rate	150

Sample Name SO_REC

Detector UV (254 nm)

dad1A.ch

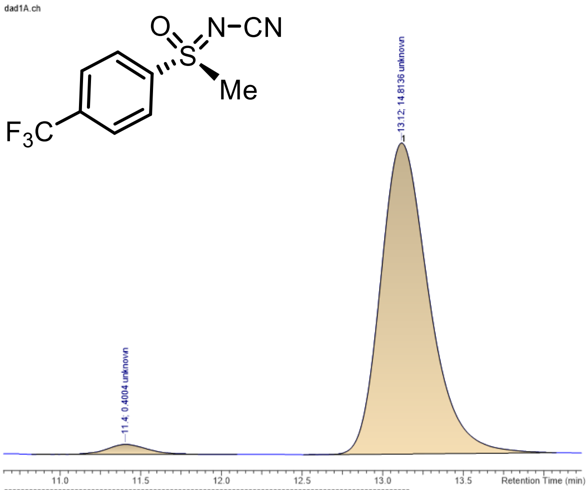

No.	Name	tR	Peak Area (Y units*ms)	Area (%)	Width
1	unknown	11.40	24021.160	2.631	0.447
2	unknown	13.12	888815.125	97.369	0.532

LC DATA FILE

10 Mar 2025

File Name	\\storage.york.ac.uk\chemistry\services\Chromatography\CDCSHPLCAG1100\20250304_BM959_4CF3_STD_ADH_8020 2025-03-04 17-52-56\SO_RACEMATE_STD.D\dad1A.ch
Title	LC DATA FILE
Sampling Rate	150

Sample Name SO_Racemate_STD

Detector UV (254 nm)

dad1A.ch

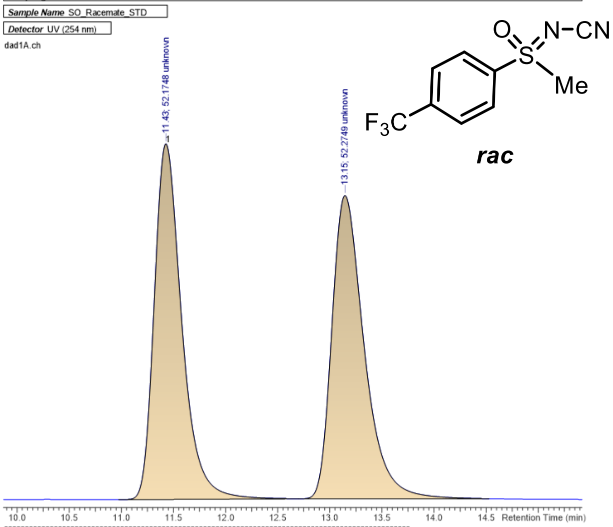

No.	Name	tR	Peak Area (Y units*ms)	Area (%)	Width
1	unknown	11.43	3130487.137	49.952	0.463
2	unknown	13.15	3136493.683	50.048	0.546

Figure S13. HPLC Data for **(*S*)-5e**

LC DATA FILE

10 Mar 2025

File Name	\\storage.york.ac.uk\chemistry\services\Chromatography\CDCSPAC\Data\20240709_BM959_ASH7030_4CN_EZS 2024-07-09 12-57-08\BSM_4CN_SULFILIMINEREC.D\dad1A.ch
Title	LC DATA FILE
Sampling Rate	150

Sample Name BSM_4CN_sulfilimineréco

Detector UV (254 nm)

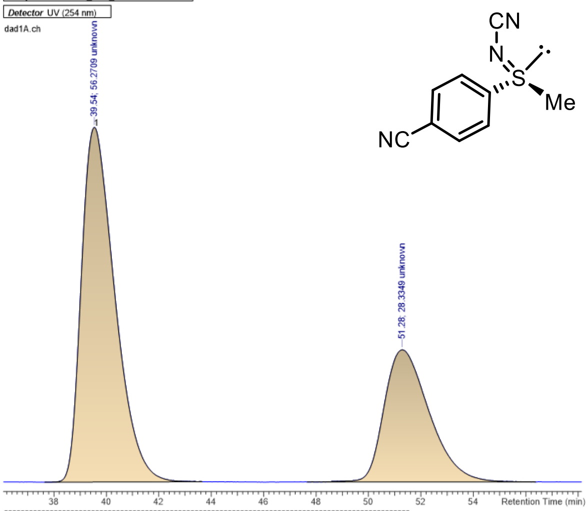

No.	Name	tR	Peak Area (Y units*ms)	Area (%)	Width
1	unknown	39.54	3376254.044	66.509	2.324
2	unknown	51.28	1700095.024	33.491	3.126

LC DATA FILE

10 Mar 2025

File Name	\\storage.york.ac.uk\chemistry\services\Chromatography\CDCSPAC\Data\20240709_BM959_ASH7030_4CN_SM 2024-07-09 11-53-42\BSM_IPR_SULFOXIMINESM.D\dad1A.ch
Title	LC DATA FILE
Sampling Rate	150

Sample Name BSM_4CN_sulfilimineSM

Detector UV (254 nm)

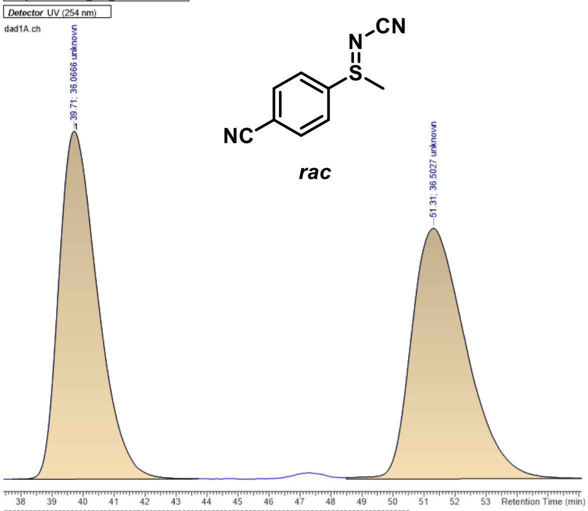

No.	Name	tR	Peak Area (Y units*ms)	Area (%)	Width
1	unknown	39.71	2163994.904	49.700	2.277
2	unknown	51.31	2190161.362	50.300	3.203

Figure S14. HPLC Data for **(*S*)-4f**

LC DATA FILE

10 Mar 2025

File Name	\\storage.york.ac.uk\chemistry\services\Chromatography\CDCSPAC\Data\20240709_BM959_ASH7030_4CN_EZS 2024-07-09 12-57-08\BSM_4CN_SULFOXIMINE_ENZ.D\dad1A.ch
Title	LC DATA FILE
Sampling Rate	150

Sample Name BSM_4CN_sulfoximine_enz

Detector UV (254 nm)

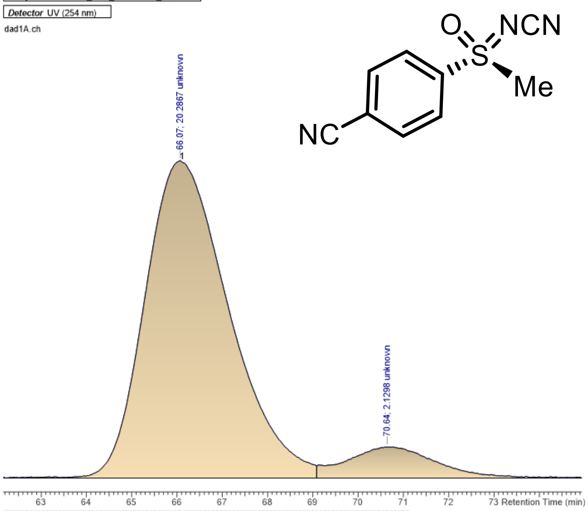

No.	Name	tR	Peak Area (Y units*ms)	Area (%)	Width
1	unknown	66.07	1217200.250	90.499	3.373
2	unknown	70.64	127786.586	9.501	3.605

LC DATA FILE

10 Mar 2025

File Name	\\storage.york.ac.uk\chemistry\services\Chromatography\CDCSPAC\Data\20240709_BM959_ASH7030_4CN_SORACE 2024-07-09 17-42-28\BSM_4CN_SULFOXIMINE_RACEMATE.D\dad1A.ch
Title	LC DATA FILE
Sampling Rate	150

Sample Name BSM_4CN_sulfoximine_racemate

Detector UV (254 nm)

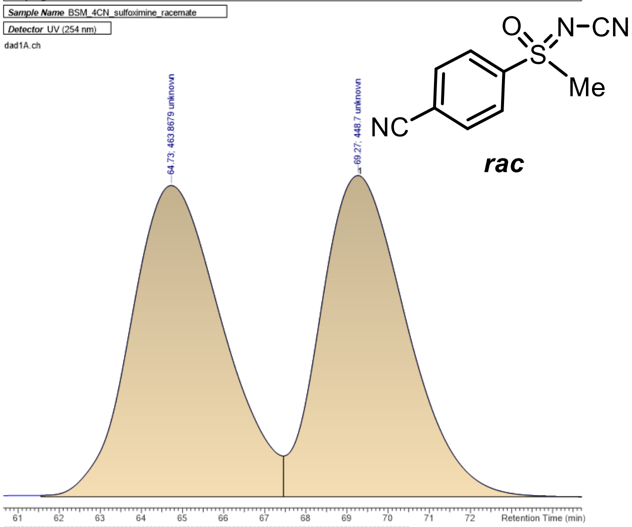

No.	Name	tR	Peak Area (Y units*ms)	Area (%)	Width
1	unknown	64.73	27832071.533	50.831	4.111
2	unknown	69.27	26921998.901	49.169	3.843

Figure S15. HPLC Data for **(*S*)-5f**

LC DATA FILE

10 Mar 2025

File Name	\\storage.york.ac.uk\chemistry\services\Chromatography\CDCSPAC\Data\241127_BM959_ADH8515_4MESMREC 2024-11-27 12-52-42\BM959_4ME_SM_REC.D\dad1A.ch
Title	LC DATA FILE
Sampling Rate	150

Sample Name BM959_4Me_SM_rec

Detector UV (254 nm)

dad1A.ch

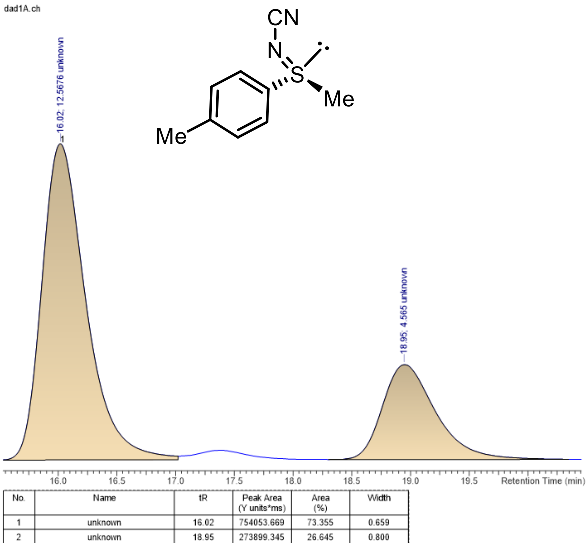

No.	Name	tR	Peak Area (Y units*ms)	Area (%)	Width
1	unknown	16.02	754053.669	73.355	0.659
2	unknown	18.95	273899.345	26.645	0.800

LC DATA FILE

10 Mar 2025

File Name	\\storage.york.ac.uk\chemistry\services\Chromatography\CDCSPAC\Data\241127_BM959_ADH8515_4MESMSTD 2024-11-27 12-06-41\BM959_4ME_SM_STD.D\dad1A.ch
Title	LC DATA FILE
Sampling Rate	150

Sample Name BM959_4Me_SM_STD

Detector UV (254 nm)

dad1A.ch

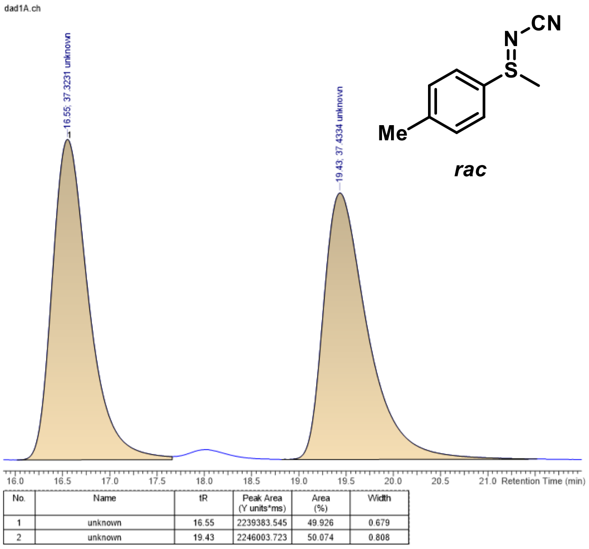

No.	Name	tR	Peak Area (Y units*ms)	Area (%)	Width
1	unknown	16.55	2239383.545	49.926	0.679
2	unknown	19.43	2246003.723	50.074	0.808

Figure S16. HPLC Data for **(*S*)-4g**

LC DATA FILE

16 Mar 2025

File Name	\\storage.york.ac.uk\chemistry\services\Chromatography\CDCSPAC\Data\250314_BM959_4MESO_ADH8515 2025-03-14 18-32-38\PRODUCT.D\dad1A.ch
Title	LC DATA FILE

Detector UV (254 nm)

UV Trace Bandwidth 4 nm

dad1A.ch

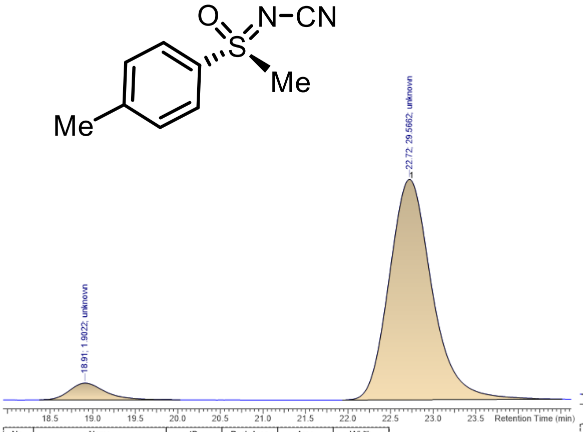

No.	Name	tR	Peak Area (Y units*ms)	Area (%)	Width
1	unknown	18.91	114129.102	6.045	0.745
2	unknown	22.72	1773973.618	93.955	0.889

LC DATA FILE

16 Mar 2025

File Name	\\storage.york.ac.uk\chemistry\services\Chromatography\CDCSPAC\Data\250314_BM959_4MESTD_ADH8515 2025-03-14 18-02-18\STD.D\dad1A.ch
Title	LC DATA FILE

Detector UV (254 nm)

UV Trace Bandwidth 4 nm

dad1A.ch

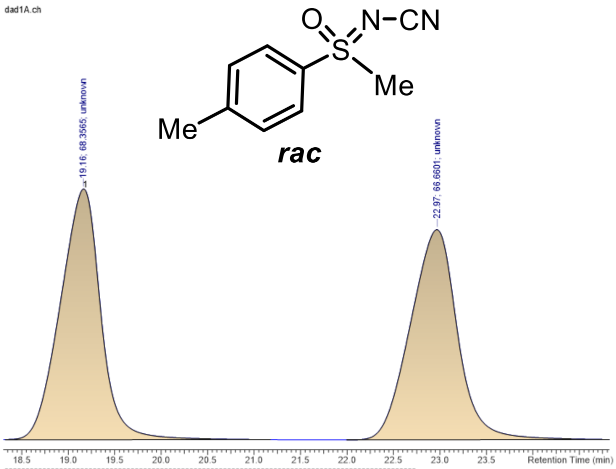

No.	Name	tR	Peak Area (Y units*ms)	Area (%)	Width
1	unknown	19.16	4101388.550	50.628	0.777
2	unknown	22.97	3999605.713	49.372	0.909

Figure S17. HPLC Data for **(*S*)-5g**

LC DATA FILE

16 Feb 2025

File Name	\\storage.york.ac.uk\chemistry\services\Chromatography\CDCSPAC\Data\241208_BM959ASH6040_3NO2 2024-12-08 15-18-51\3-NO2_SM-REC.D\dad1E.ch
Title	LC DATA FILE
Sampling Rate	150

Sample Name 3-NO2_SM-REC

Detector UV (280 nm)

dad1E.ch

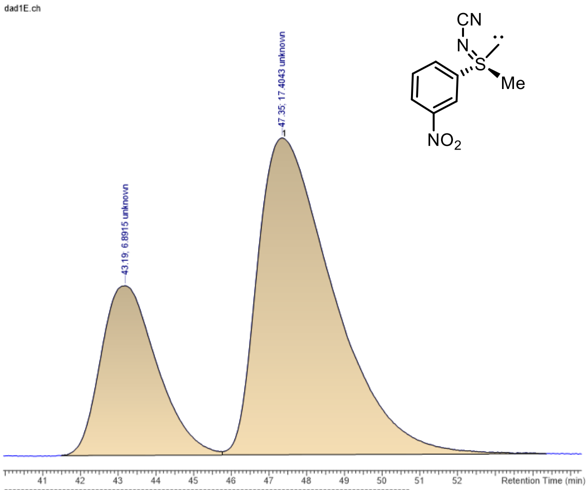

No.	Name	tR	Peak Area (Y units*ms)	Area (%)	Width
1	unknown	43.19	413488.226	28.365	2.728
2	unknown	47.35	1044260.330	71.635	3.617

LC DATA FILE

16 Feb 2025

File Name	\\storage.york.ac.uk\chemistry\services\Chromatography\CDCSPAC\Data\241208_BM959ASH6040_3NO2 2024-12-08 15-18-51\3-NO2_STD.D\dad1E.ch
Title	LC DATA FILE
Sampling Rate	150

Sample Name 3-NO2_STD

Detector UV (280 nm)

dad1E.ch

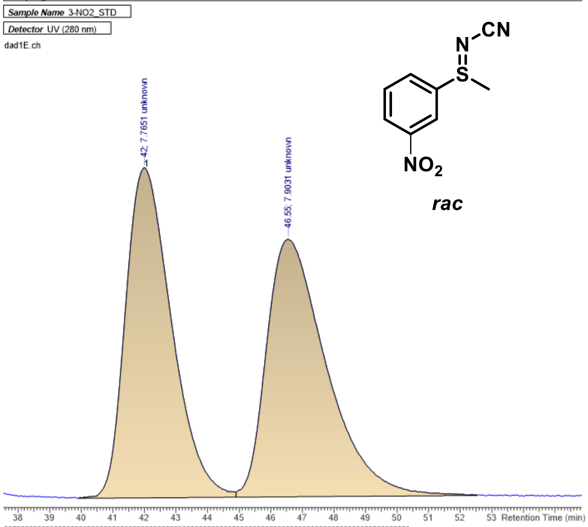

No.	Name	tR	Peak Area (Y units*ms)	Area (%)	Width
1	unknown	42.00	465907.030	49.560	2.659
2	unknown	46.55	474187.860	50.440	3.366

Figure S18. HPLC Data for **(*S*)-4h**

LC DATA FILE

7 Apr 2025

File Name	\\storage.york.ac.uk\chemistry\services\Chromatography\CDCSPAC\Data\20250403_BM959_ASH6040_3NO2STD 2025-04-03 16-36-07\ENZ.D\dad1A.ch
Title	LC DATA FILE
Sampling Rate	150

Sample Name ENZ

Detector UV (254 nm)

dad1A.ch

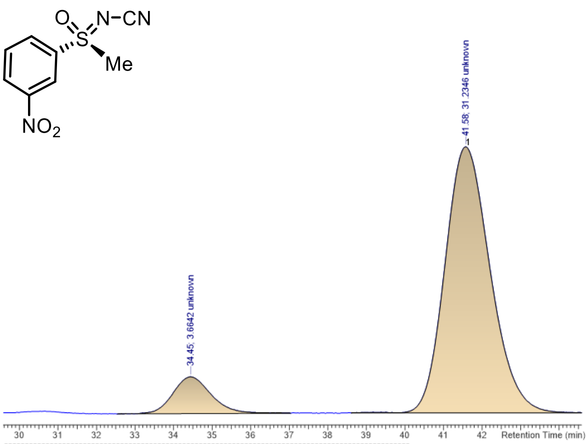

No.	Name	tR	Peak Area (Y units*ms)	Area (%)	Width
1	unknown	34.45	219851.589	10.499	1.768
2	unknown	41.58	1874073.601	89.501	2.136

LC DATA FILE

7 Apr 2025

File Name	\\storage.york.ac.uk\chemistry\services\Chromatography\CDCSPAC\Data\20250403_BM959_ASH6040_3NO2STD 2025-04-03 16-36-07\STD.D\dad1A.ch
Title	LC DATA FILE
Sampling Rate	150

Sample Name STD

Detector UV (254 nm)

dad1A.ch

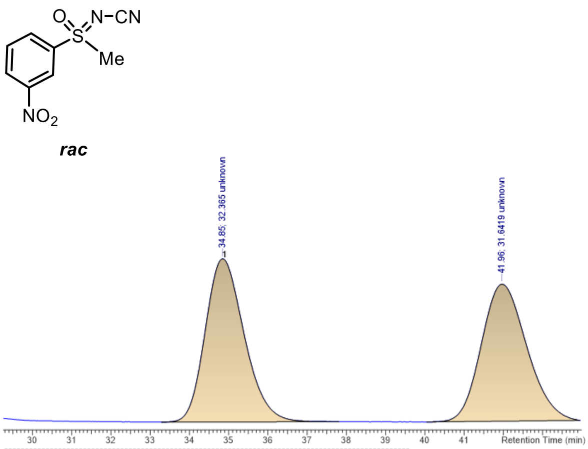

No.	Name	tR	Peak Area (Y units*ms)	Area (%)	Width
1	unknown	34.85	1941900.101	50.565	1.805
2	unknown	41.96	1898514.748	49.435	2.127

Figure S19. HPLC Data for **(*S*)-5h**

LC DATA FILE

15 Feb 2025

File Name	\\storage.york.ac.uk\chemistry\services\Chromatography\CDCSPAC\Data\241210_BM959_1023_3CL_ASH6535 2024-12-10 13-29-56\3CL SM REC.D\dad1E.ch
Title	LC DATA FILE
Sampling Rate	150

Sample Name 3CL SM rec

Detector UV (280 nm)

dad1E.ch

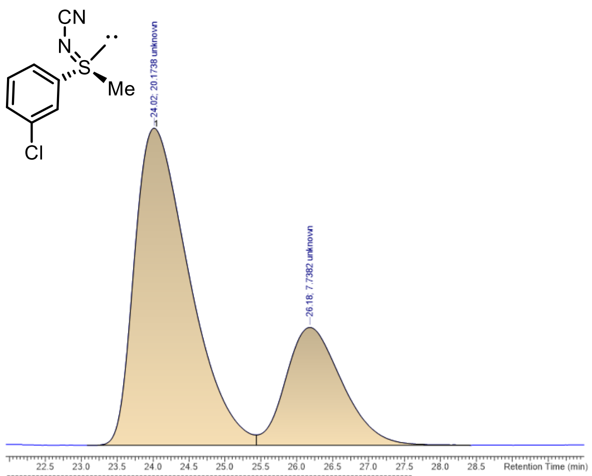

No.	Name	tR	Peak Area (Y units*ms)	Area (%)	Width
1	unknown	24.02	1210428.543	72.276	1.399
2	unknown	26.18	464294.128	27.724	1.431

LC DATA FILE

15 Feb 2025

File Name	\\storage.york.ac.uk\chemistry\services\Chromatography\CDCSPAC\Data\241210_BM959_1023_3CL_ASH6535 2024-12-10 13-29-56\3CL SM STD.D\dad1E.ch
Title	LC DATA FILE
Sampling Rate	150

Sample Name 3CL SM STD

Detector UV (280 nm)

dad1E.ch

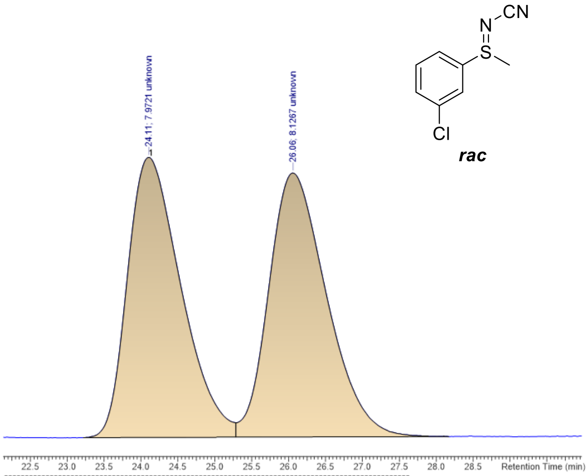

No.	Name	tR	Peak Area (Y units*ms)	Area (%)	Width
1	unknown	24.11	478327.074	49.520	1.334
2	unknown	26.06	487599.449	50.480	1.428

Figure S20. HPLC Data for **(*S*)-4i**

LC DATA FILE

16 Mar 2025

File Name	\\storage.york.ac.uk\chemistry\services\Chromatography\CDCSPAC\Data\250314_BM959_4MESO_ADH8515 2025-03-14 19-31-14\PRODUCT.D\dad1A.ch
Type	HPLC
Points Count	4651
Date Stamp	14-Mar-25, 20:03:34
Date	14 Mar 2025 20:34:38
Title	LC DATA FILE
Comment	pRODUCT
Sampling Rate	150
Detector Lag	0
Short File Name	dad1A.ch

Sample Name pRODUCT

Detector	UV (254 nm)
UV Trace Bandwidth	4 nm

dad1A.ch

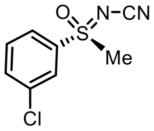

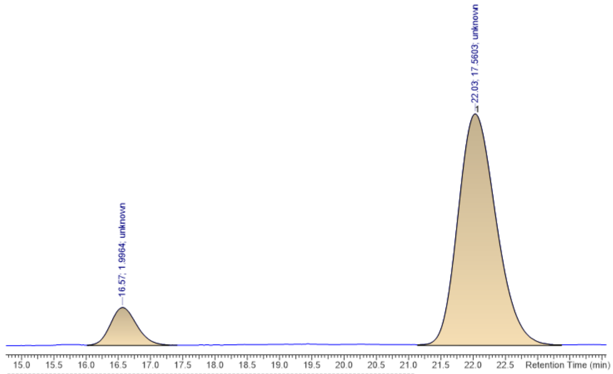

No.	Name	tR	Peak Area (Y units*ms)	Area (%)	Width
1	unknown	16.57	119785.383	10.208	0.746
2	unknown	22.03	1053616.500	89.792	1.083

LC DATA FILE

16 Mar 2025

File Name	\\storage.york.ac.uk\chemistry\services\Chromatography\CDCSPAC\Data\250314_BM959_4MESO_ADH8515 2025-03-14 19-31-14\STDF.D\dad1A.ch
Type	HPLC
Points Count	4112
Date Stamp	14-Mar-25, 20:35:47
Date	14 Mar 2025 21:03:14
Title	LC DATA FILE
Comment	STD.C
Sampling Rate	150
Detector Lag	0
Short File Name	dad1A.ch

Sample Name STD.C

Detector	UV (254 nm)
UV Trace Bandwidth	4 nm

dad1A.ch

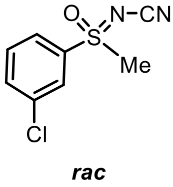

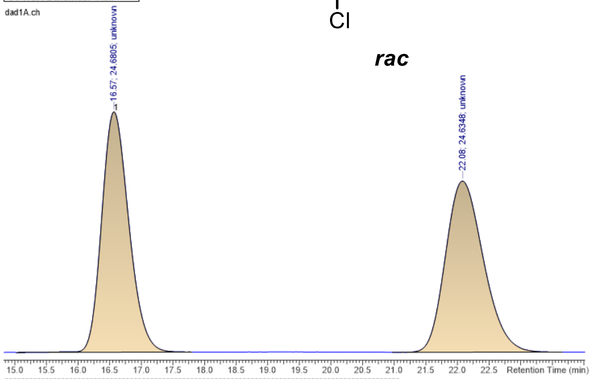

No.	Name	tR	Peak Area (Y units*ms)	Area (%)	Width
1	unknown	16.57	1480831.490	50.046	0.781
2	unknown	22.08	1478087.540	49.954	1.102

Figure S21. Data for **(*S*)-5i**

LC DATA FILE

11 Mar 2025

File Name	\\storage.york.ac.uk\chemistry\services\Chromatography\CDCSHPLCAG1100\20250217_BM959_ASH_7030_4CF33CL_SCALE 2025-02-24 12-13-32\SI_REC.D\dad1A.ch
Title	LC DATA FILE
Sampling Rate	150

Sample Name SI_REC

Detector UV (254 nm)

dad1A.ch

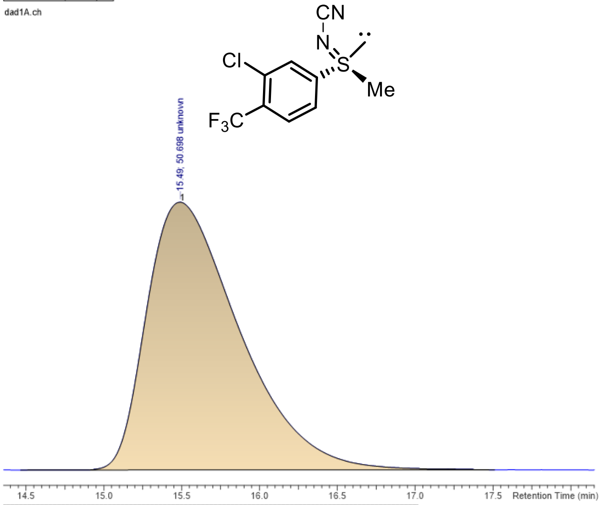

No.	Name	tR	Peak Area (Y units*ms)	Area (%)	Width
1	unknown	15.49	3041877.823	100.000	1.085

LC DATA FILE

11 Mar 2025

File Name	\\storage.york.ac.uk\chemistry\services\Chromatography\CDCSHPLCAG1100\20250217_BM959_ASH_7030_4CF33CL_SCALE 2025-02-24 12-13-32\SI_STD.D\dad1A.ch
Title	LC DATA FILE
Sampling Rate	150

Sample Name SI_STD

Detector UV (254 nm)

dad1A.ch

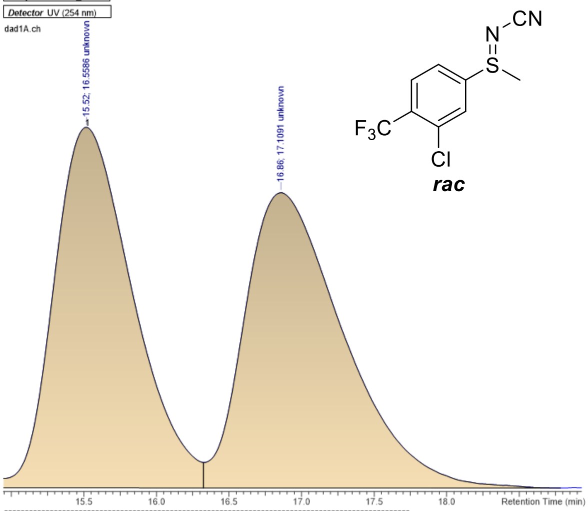

No.	Name	tR	Peak Area (Y units*ms)	Area (%)	Width
1	unknown	15.52	993516.541	49.182	0.993
2	unknown	16.86	1026548.882	50.818	1.233

Figure S22. HPLC Data for **(*S*)-4j**

LC DATA FILE

11 Mar 2025

File Name	\\storage.york.ac.uk\chemistry\services\Chromatography\CDCSHPLCAG1100\20250217_BM959_ASH_7030_4CF33CL_SCALE 2025-02-24 12-13-32\SO_REC.D\dad1A.ch
Title	LC DATA FILE
Sampling Rate	150

Sample Name SO_REC

Detector UV (254 nm)

dad1A.ch

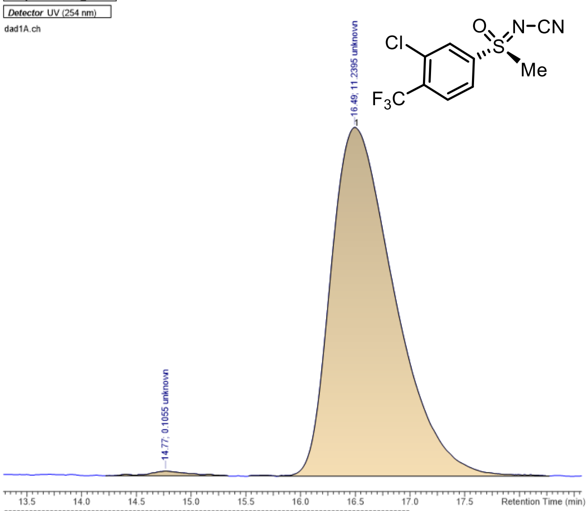

No.	Name	tR	Peak Area (Y units*ms)	Area (%)	Width
1	unknown	14.77	6331.787	0.930	0.581
2	unknown	16.49	674369.375	99.070	1.055

LC DATA FILE

11 Mar 2025

File Name	\\storage.york.ac.uk\chemistry\services\Chromatography\CDCSHPLCAG1100\20250217_BM959_ASH_7030_4CF33CL_SCALE 2025-02-24 12-13-32\SO_STD.D\dad1A.ch
Title	LC DATA FILE
Sampling Rate	150

Sample Name SO_STD

Detector UV (254 nm)

dad1A.ch

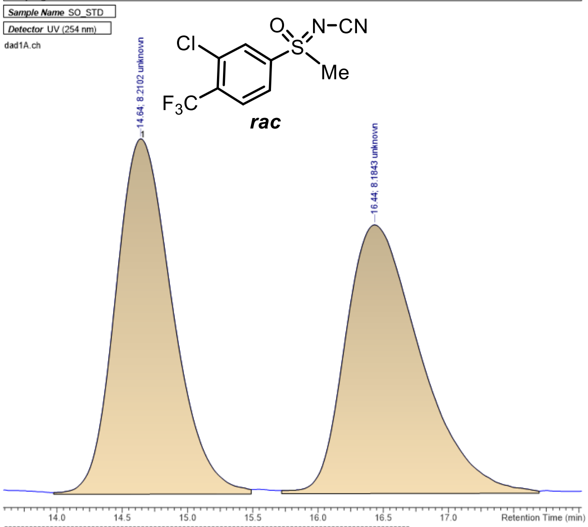

No.	Name	tR	Peak Area (Y units*ms)	Area (%)	Width
1	unknown	14.64	492611.625	50.079	0.789
2	unknown	16.44	491060.406	49.921	1.036

Figure S23. HPLC Data for **(*S*)-5j**

LC DATA FILE

11 Mar 2025

File Name	\\storage.york.ac.uk\chemistry\services\Chromatography\CDCSHPLCAG1100\20250212_BM959_2PYRD_PURIFI_SO_80_20_ADH 2025-02-17 15-36-39\SO_REC.D\dad1A.ch
Title	LC DATA FILE
Sampling Rate	150

Sample Name SO_REC

Detector UV (254 nm)

dad1A.ch

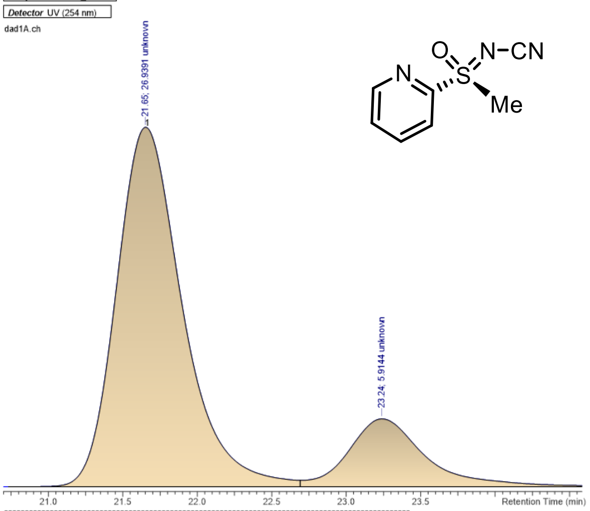

No.	Name	tR	Peak Area (Y units*ms)	Area (%)	Width
1	unknown	21.65	1616344.528	81.998	0.780
2	unknown	23.24	354863.720	18.002	0.851

LC DATA FILE

11 Mar 2025

File Name	\\storage.york.ac.uk\chemistry\services\Chromatography\CDCSHPLCAG1100\20250212_BM959_2PYRD_PURIFIED_80_20_ADH 2025-02-17 13-55-28\SM_MCPBA.D\dad1A.ch
Title	LC DATA FILE
Sampling Rate	150

Sample Name SM_mcpba

Detector UV (254 nm)

dad1A.ch

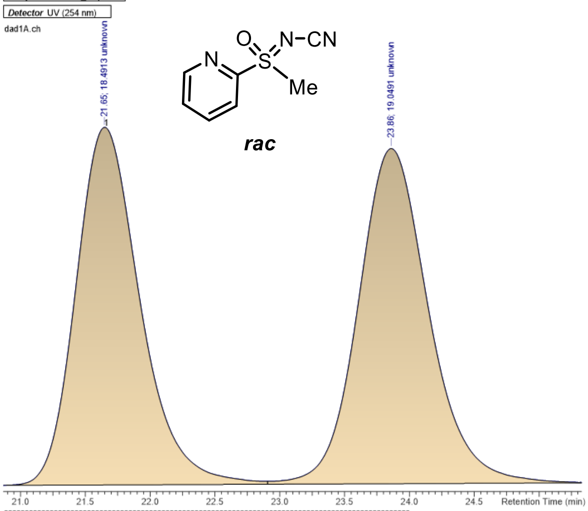

No.	Name	tR	Peak Area (Y units*ms)	Area (%)	Width
1	unknown	21.65	1109476.625	49.257	0.881
2	unknown	23.86	1142948.000	50.743	0.961

Figure S24. HPLC Data for **(*S*)-5k**

LC DATA FILE

16 Mar 2025

File Name	\\storage.york.ac.uk\chemistry\services\Chromatography\CDCSPAC\Data\250314_BM959_4CF3STD_ADH8515 2025-03-14 16-39-09\SM.D\dad1E.ch
Title	LC DATA FILE
Detector	UV (280 nm)
UV Trace Bandwidth	16 nm

dad1E.ch

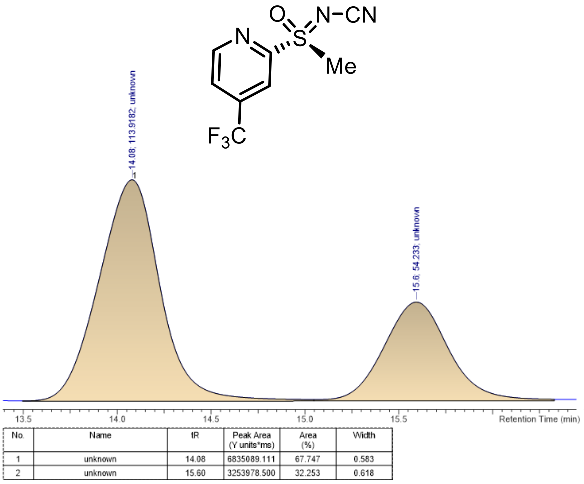

No.	Name	tR	Peak Area (Y units*ms)	Area (%)	Width
1	unknown	14.08	6835089.111	67.747	0.583
2	unknown	15.60	3253978.500	32.253	0.618

LC DATA FILE

16 Mar 2025

File Name	\\storage.york.ac.uk\chemistry\services\Chromatography\CDCSPAC\Data\250314_BM959_4CF3STD_ADH8515 2025-03-14 16-39-09\STDCONVC.D\dad1E.ch
Title	LC DATA FILE
Detector	UV (280 nm)
UV Trace Bandwidth	16 nm

dad1E.ch

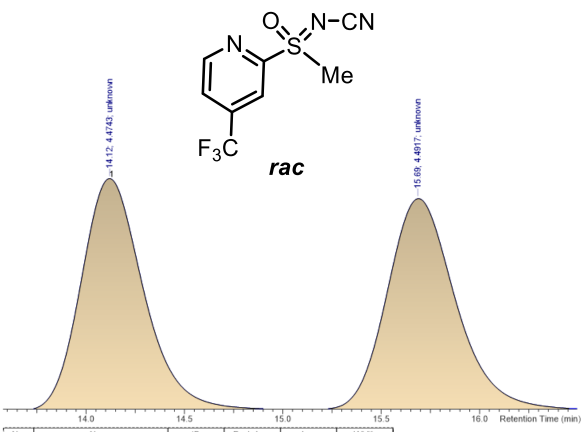

No.	Name	tR	Peak Area (Y units*ms)	Area (%)	Width
1	unknown	14.12	268457.022	49.903	0.571
2	unknown	15.69	269499.578	50.097	0.627

Figure S25. HPLC Data for **(*S*)-4l**

LC DATA FILE

16 Mar 2025

File Name	\\storage.york.ac.uk\chemistry\services\Chromatography\CDCSPAC\Data\250314_BM959_4CF3STD_ADH8515 2025-03-14 16-39-09\PRODUCT.D\dad1E.ch
Title	LC DATA FILE
Detector	UV (280 nm)
UV Trace Bandwidth	16 nm

dad1E.ch

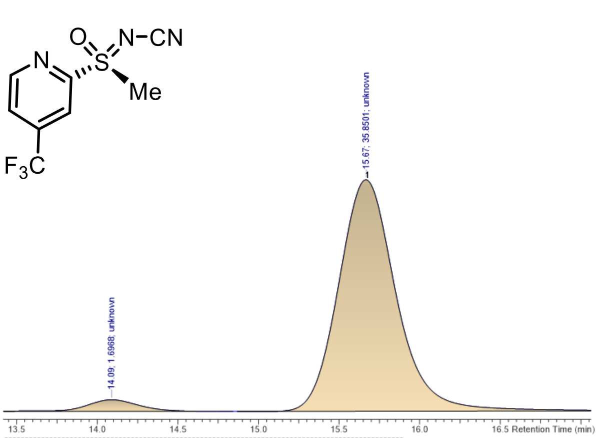

No.	Name	tR	Peak Area (Y units*ms)	Area (%)	Width
1	unknown	14.09	101810.889	4.519	0.573
2	unknown	15.67	2151007.919	95.481	0.629

LC DATA FILE

16 Mar 2025

File Name	\\storage.york.ac.uk\chemistry\services\Chromatography\CDCSPAC\Data\250314_BM959_4CF3STD_ADH8515 2025-03-14 16-39-09\STDCONVC.D\dad1E.ch
Title	LC DATA FILE
Detector	UV (280 nm)
UV Trace Bandwidth	16 nm

dad1E.ch

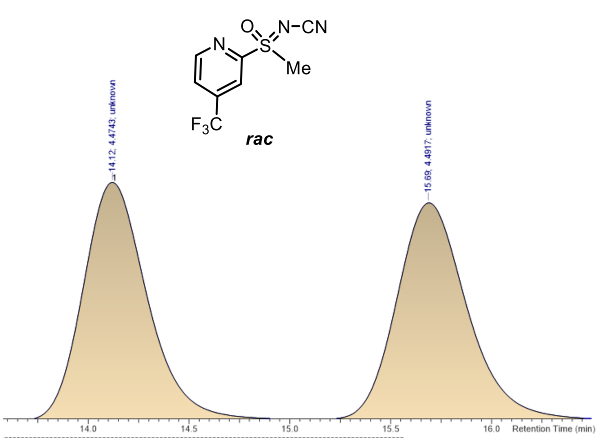

No.	Name	tR	Peak Area (Y units*ms)	Area (%)	Width
1	unknown	14.12	268457.022	49.903	0.571
2	unknown	15.69	269499.578	50.097	0.627

Figure S26. HPLC Data for **(*S*)-5l**

LC DATA FILE

25 Feb 2025

File Name	\\storage.york.ac.uk\chemistry\services\Chromatography\CDCSHPLCAG1100\20250214_BM959_ADH_8515_511_STD_PURE 2025-02-14 16-39-45\SM_REC_MCPBA.D\dad1A.ch
Title	LC DATA FILE
Sampling Rate	150

Sample Name SM_rec_mcpba

Detector UV (254 nm)

dad1A.ch

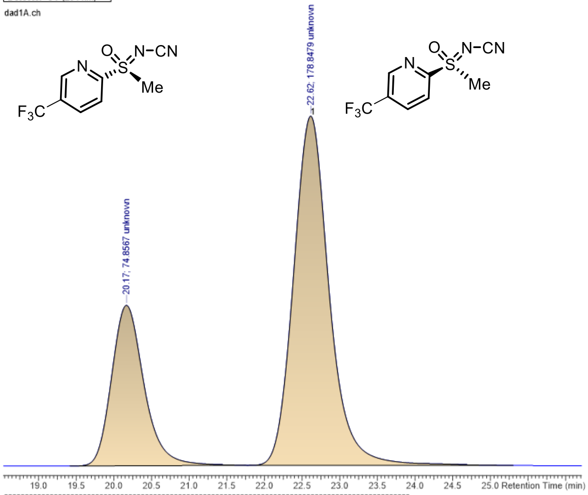

No.	Name	tR	Peak Area (Y units*ms)	Area (%)	Width
1	unknown	20.17	4491403.198	29.505	0.763
2	unknown	22.62	10730874.023	70.495	0.828

LC DATA FILE

25 Feb 2025

File Name	\\storage.york.ac.uk\chemistry\services\Chromatography\CDCSHPLCAG1100\20250214_BM959_ADH_8515_511_STD_PURE 2025-02-14 16-39-45\STD_SO.D\dad1A.ch
Title	LC DATA FILE
Sampling Rate	150

Sample Name STD_SO

Detector UV (254 nm)

dad1A.ch

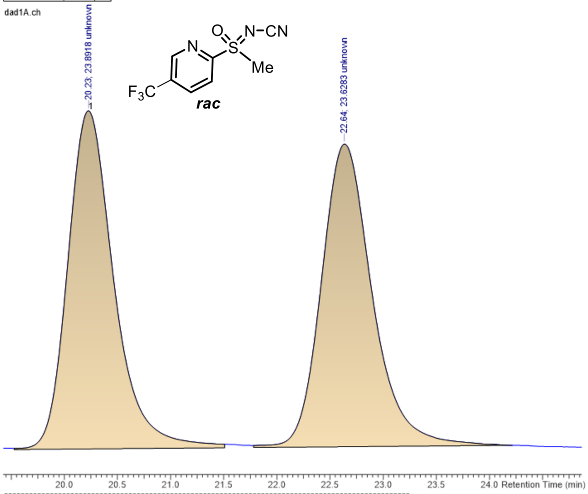

No.	Name	tR	Peak Area (Y units*ms)	Area (%)	Width
1	unknown	20.23	1433509.483	50.277	0.757
2	unknown	22.64	1417698.784	49.723	0.839

Figure S27. HPLC Data for **(*S*)-4m** following m-CPBA Oxidation

LC DATA FILE

11 Mar 2025

File Name	\\storage.york.ac.uk\chemistry\services\Chromatography\CDCSHPLCAG1100\20250212_BM959_511_PURIFIED_85_15_ADH 2025-02-14 19-13-05\SULFOXIMINE_RECOVERED.D\dad1A.ch
Title	LC DATA FILE
Sampling Rate	150

Sample Name Sulfoximine_recovered

Detector UV (254 nm)

dad1A.ch

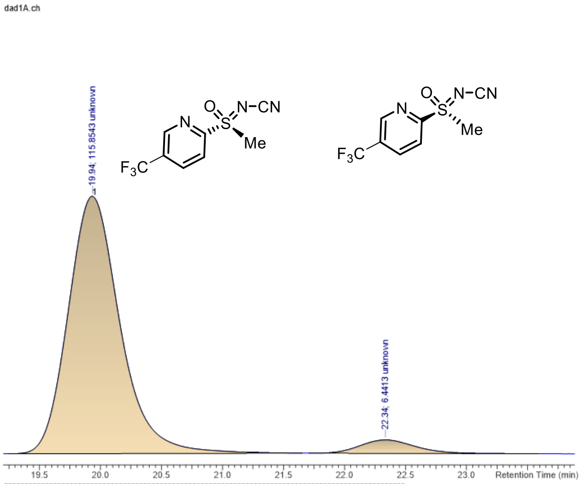

No.	Name	tR	Peak Area (Y units*ms)	Area (%)	Width
1	unknown	19.94	6951255.341	94.733	0.750
2	unknown	22.34	386475.706	5.267	0.821

LC DATA FILE

25 Feb 2025

File Name	\\storage.york.ac.uk\chemistry\services\Chromatography\CDCSHPLCAG1100\20250214_BM959_ADH_8515_511_STD_PURE 2025-02-14 16-39-45\STD_SO.D\dad1A.ch
Title	LC DATA FILE
Sampling Rate	150

Sample Name STD_SO

Detector UV (254 nm)

dad1A.ch

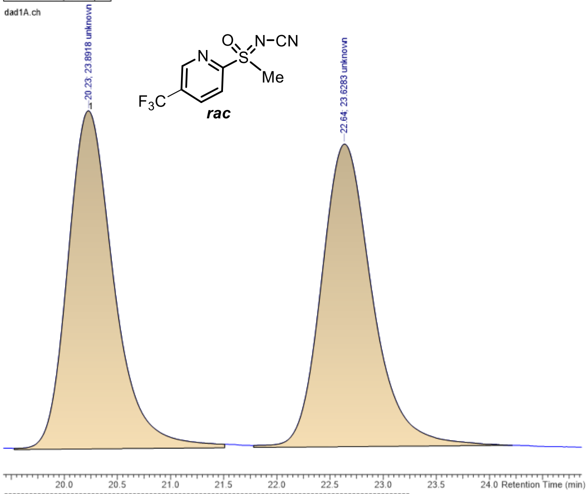

No.	Name	tR	Peak Area (Y units*ms)	Area (%)	Width
1	unknown	20.23	1433509.483	50.277	0.757
2	unknown	22.64	1417698.784	49.723	0.839

Figure S28. HPLC Data for **(*S*)-5m**

LC DATA FILE

11 Mar 2025

File Name	\\storage.york.ac.uk\chemistry\services\Chromatography\CDCSHPLCAG1100\20250217_BM959_ADH_8020_ETHYL_REC 2025-02-19 14-44-56\SM_REC.D\dad1A.ch
Title	LC DATA FILE
Sampling Rate	150
Sample Name	SM_REC
Detector	UV (254 nm)

dad1A.ch

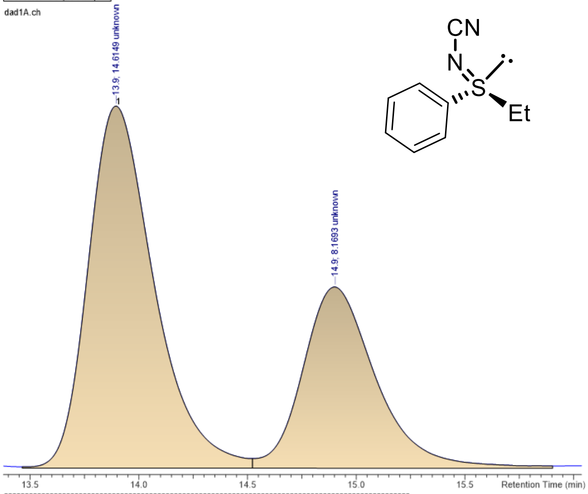

No.	Name	tR	Peak Area (Y units*ms)	Area (%)	Width
1	unknown	13.90	876894.500	64.145	0.539
2	unknown	14.90	490158.531	35.855	0.586

LC DATA FILE

11 Mar 2025

File Name	\\storage.york.ac.uk\chemistry\services\Chromatography\CDCSHPLCAG1100\20250217_BM959_ADH_8020_ETHYL_STD 2025-02-19 09-58-19\SM_STD.D\dad1A.ch
Title	LC DATA FILE
Sampling Rate	150
Sample Name	SM_STD
Detector	UV (254 nm)

dad1A.ch

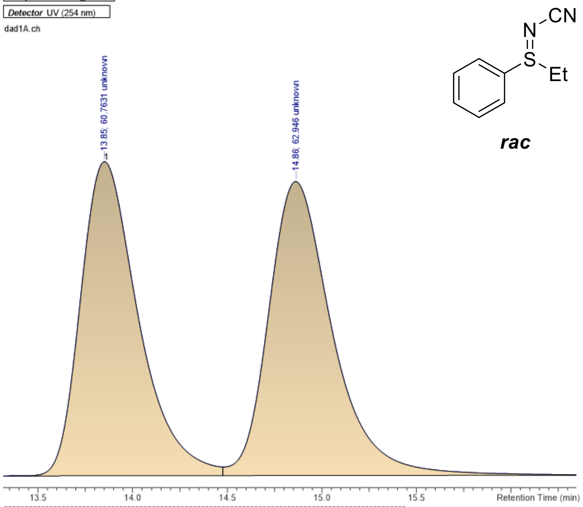

No.	Name	tR	Peak Area (Y units*ms)	Area (%)	Width
1	unknown	13.85	3645786.896	49.118	0.541
2	unknown	14.86	3776762.924	50.882	0.585

Figure S29. HPLC Data for **(*S*)-4n**

LC DATA FILE

11 Mar 2025

File Name	\\storage.york.ac.uk\chemistry\services\Chromatography\CDCSHPLCAG1100\20250217_BM959_ADH_8020_ETHYL_REC 2025-02-19 14-44-56\SO_REC.D\dad1A.ch
Title	LC DATA FILE
Sampling Rate	150
Sample Name	SO_REC
Detector	UV (254 nm)

dad1A.ch

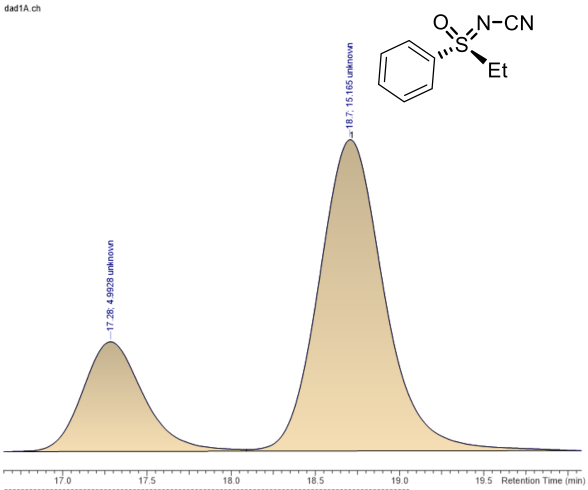

No.	Name	tR	Peak Area (Y units*ms)	Area (%)	Width
1	unknown	17.28	299566.412	24.768	0.643
2	unknown	18.70	909900.169	75.232	0.684

LC DATA FILE

11 Mar 2025

File Name	\\storage.york.ac.uk\chemistry\services\Chromatography\CDCSHPLCAG1100\20250217_BM959_ADH_8020_ETHYL_STD 2025-02-19 09-58-19\SO_STD.D\dad1A.ch
Title	LC DATA FILE
Sampling Rate	150
Sample Name	SO_STD
Detector	UV (254 nm)

dad1A.ch

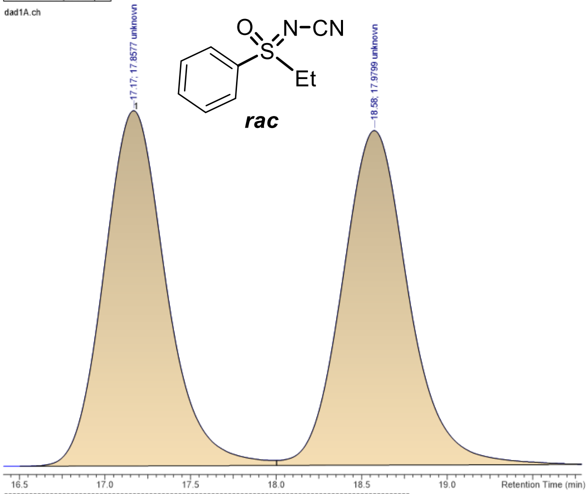

No.	Name	tR	Peak Area (Y units*ms)	Area (%)	Width
1	unknown	17.17	1071461.334	49.829	0.646
2	unknown	18.58	1078795.509	50.171	0.687

Figure S30. HPLC Data for **(*S*)-5n**

LC DATA FILE

11 Mar 2025

File Name	\\storage.york.ac.uk\chemistry\services\Chromatography\CDCSPAC\Data\240102_BM959_ADH-_NPR_ENZ 2024-12-02 18-43-29\N-PR-RECSM.D\dad1A.ch
Title	LC DATA FILE
Sampling Rate	150

Sample Name n-pr-recSM

Detector UV (254 nm)

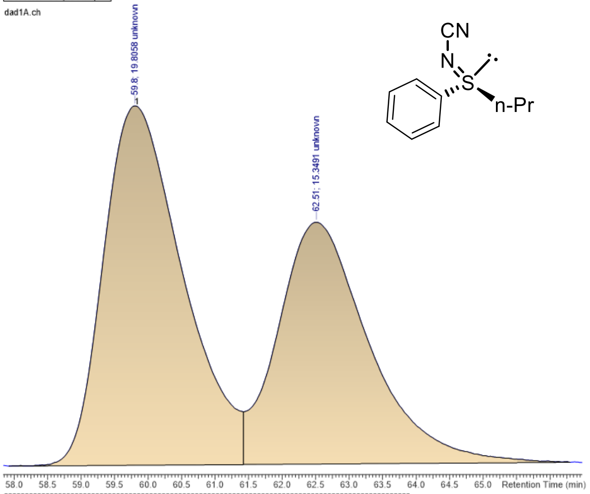

No.	Name	tR	Peak Area (Y units*ms)	Area (%)	Width
1	unknown	59.80	1188348.312	56.339	2.169
2	unknown	62.51	920944.462	43.661	2.386

LC DATA FILE

11 Mar 2025

File Name	\\storage.york.ac.uk\chemistry\services\Chromatography\CDCSPAC\Data\240102_BM959_ADH-_NPR_ENZ 2024-12-02 18-43-29\N-PR-SM-STD.D\dad1A.ch
Title	LC DATA FILE
Sampling Rate	150

Sample Name n-pr-SM-std

Detector UV (254 nm)

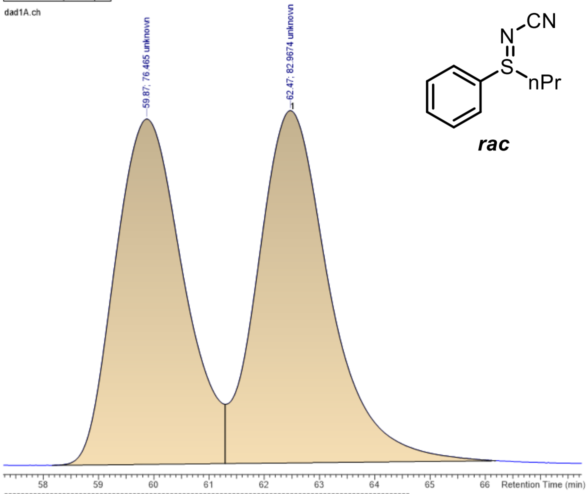

No.	Name	tR	Peak Area (Y units*ms)	Area (%)	Width
1	unknown	59.87	4587899.780	47.961	2.300
2	unknown	62.47	4978043.518	52.039	2.271

Figure S31. HPLC Data for ***(S*)-4o**

LC DATA FILE

12 Mar 2025

File Name	\\storage.york.ac.uk\chemistry\services\Chromatography\CDCSHPLCAG1100\20250226_BM959_ADH_90_10_NPR_SO_STD 2025-02-26 17-31-19\SULFOXIMINE_RECO.D\dad1A.ch
Title	LC DATA FILE
Sampling Rate	150

Sample Name Sulfoximine_reco

Detector UV (254 nm)

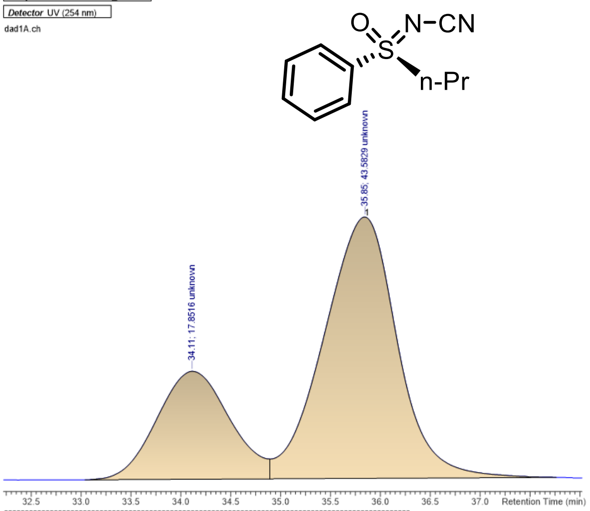

No.	Name	tR	Peak Area (Y units*ms)	Area (%)	Width
1	unknown	34.11	1071098.557	29.058	1.361
2	unknown	35.85	2614973.145	70.942	1.319

LC DATA FILE

12 Mar 2025

File Name	\\storage.york.ac.uk\chemistry\services\Chromatography\CDCSHPLCAG1100\20250226_BM959_ADH_90_10_NPR_SO_STD 2025-02-26 17-31-19\SULFOXIMINE_STD.D\dad1A.ch
Title	LC DATA FILE
Sampling Rate	150

Sample Name Sulfoximine_STD

Detector UV (254 nm)

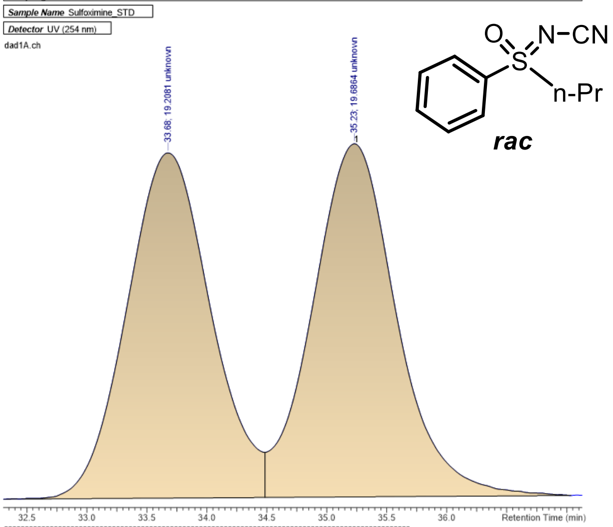

No.	Name	tR	Peak Area (Y units*ms)	Area (%)	Width
1	unknown	33.68	1152487.564	49.385	1.280
2	unknown	35.23	1181184.769	50.615	1.240

Figure S32. HPLC Data for ***(S*)-5o**

LC DATA FILE

12 Mar 2025

File Name	\\storage.york.ac.uk\chemistry\services\Chromatography\CDCSPAC\Data\241128_BM959_ASH7030_IPRSM_STD 2024-11-28 10-41-47\IPR_SM_ENZ.D\dad1A.ch
Title	LC DATA FILE
Sampling Rate	150

Sample Name iPr_SM_Enz

Detector UV (254 nm)

dad1A.ch

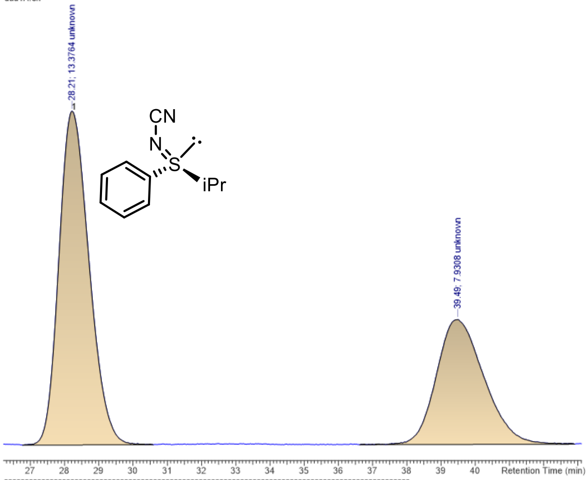

No.	Name	tR	Peak Area (Y units*ms)	Area (%)	Width
1	unknown	28.21	802583.942	62.779	1.621
2	unknown	39.49	475846.081	37.221	2.539

LC DATA FILE

12 Mar 2025

File Name	\\storage.york.ac.uk\chemistry\services\Chromatography\CDCSPAC\Data\241128_BM959_ASH7030_IPRSM_STD 2024-11-28 10-41-47\IPR_SM_STD.D\dad1A.ch
Title	LC DATA FILE
Sampling Rate	150

Sample Name iPr_SM_Std

Detector UV (254 nm)

dad1A.ch

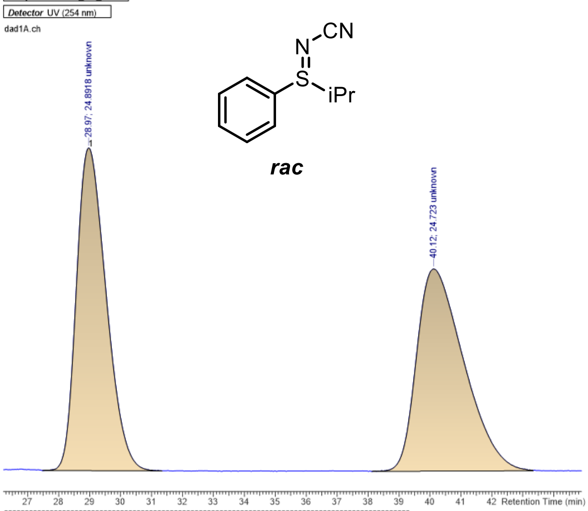

No.	Name	tR	Peak Area (Y units*ms)	Area (%)	Width
1	unknown	28.97	1493505.375	50.170	1.772
2	unknown	40.12	1483381.250	49.830	2.812

Figure S33. HPLC Data for **(*S*)-4p**

LC DATA FILE

22 Mar 2025

File Name	\\storage.york.ac.uk\chemistry\services\Chromatography\CDCSPAC\Data\20250322_BM959_ISOPROPYLSTD_7030ADS 2025-03-22 19-49-01\ISOPROPYLREC.D\dad1A.ch
Title	LC DATA FILE
Sampling Rate	150

Sample Name isopropylrec

Detector UV (254 nm)

dad1A.ch

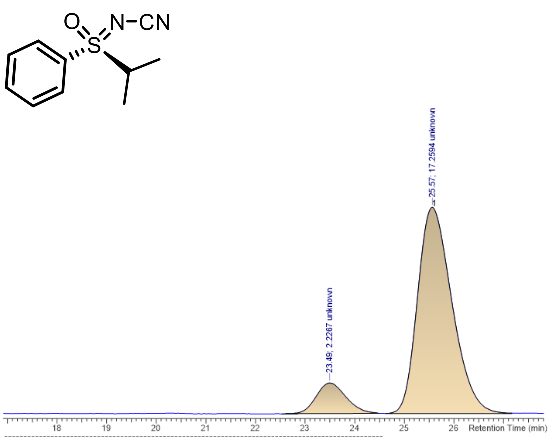

No.	Name	tR	Peak Area (Y units*ms)	Area (%)	Width
1	unknown	23.49	133602.719	11.427	1.106
2	unknown	25.57	1035566.812	88.573	1.270

LC DATA FILE

22 Mar 2025

File Name	\\storage.york.ac.uk\chemistry\services\Chromatography\CDCSPAC\Data\20250322_BM959_ISOPROPYLSTD_7030ADS 2025-03-22 19-49-01\STD.D\dad1A.ch
Title	LC DATA FILE
Sampling Rate	150

Sample Name std

Detector UV (254 nm)

dad1A.ch

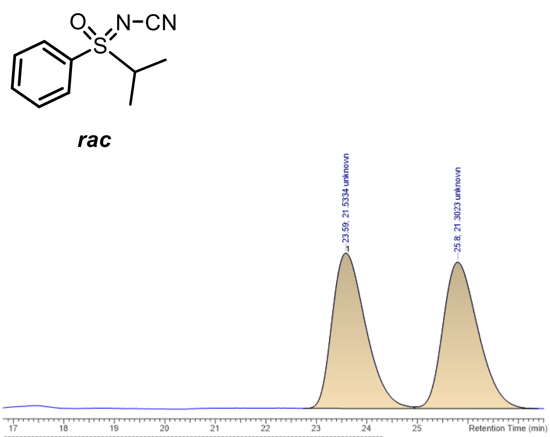

No.	Name	tR	Peak Area (Y units*ms)	Area (%)	Width
1	unknown	23.59	1292003.500	50.270	1.213
2	unknown	25.80	1278138.750	49.730	1.273

Figure S34. HPLC Data for **(*S*)-5p**

LC DATA FILE

12 Mar 2025

File Name	\\storage.york.ac.uk\chemistry\services\Chromatography\CDCSPAC\Data\241125_BM959_522ASMMCPBA_2_ADH8515_493-1 2024-11-25 17-22-09\522A_SM_ENZ_MCPBA_2.D\dad1A.ch
Title	LC DATA FILE
Sampling Rate	150

Sample Name 522A_SM_ENZ_mcpba_2

Detector UV (254 nm)

dad1A.ch

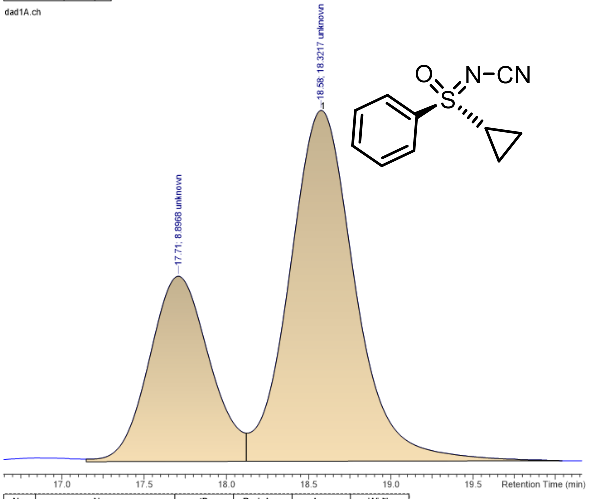

No.	Name	tR	Peak Area (Y units*ms)	Area (%)	Width
1	unknown	17.71	533809.834	32.687	0.671
2	unknown	18.58	1099304.581	67.313	0.696

LC DATA FILE

9 May 2025

File Name	\\storage.york.ac.uk\chemistry\services\Chromatography\CDCSPAC\Data\20250322_BM959_CYCLOPROPYLSTD_8515ADH 2025-03-22 17-25-27\CYCLOPROPYLSTD.D\dad1A.ch
Title	LC DATA FILE
Sampling Rate	150

Sample Name Cyclopropylstd

Detector UV (254 nm)

dad1A.ch

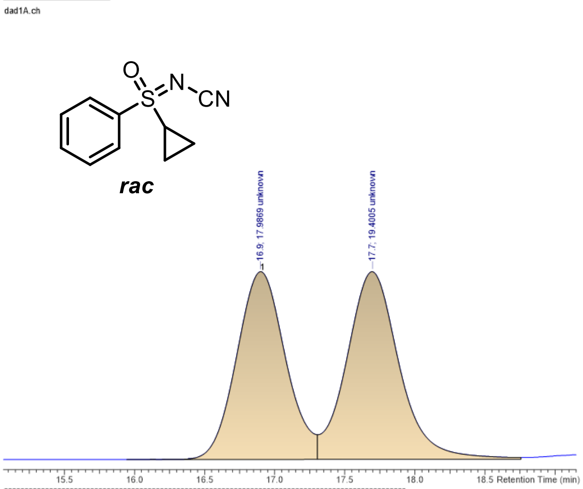

No.	Name	tR	Peak Area (Y units*ms)	Area (%)	Width
1	unknown	16.90	1079215.736	48.110	0.635
2	unknown	17.70	1164031.677	51.890	0.654

Figure S35. HPLC Data for **(*S*)-4q**

LC DATA FILE

22 Mar 2025

File Name	\\storage.york.ac.uk\chemistry\services\Chromatography\CDCSPAC\Data\250318_BM959_CYCLOWHSTD_STD_ADH8713 2025-03-18 15-15-49\REC_SO.D\dad1A.ch
Title	LC DATA FILE
Sampling Rate	150

Sample Name Rec_SO

Detector UV (254 nm)

dad1A.ch

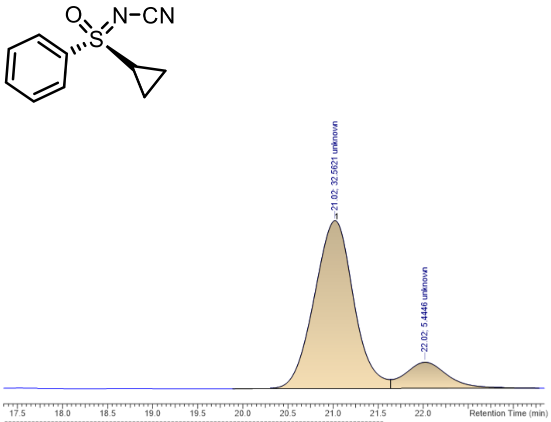

No.	Name	tR	Peak Area (Y units*ms)	Area (%)	Width
1	unknown	21.02	1953724.365	85.675	0.786
2	unknown	22.02	326675.663	14.325	0.849

LC DATA FILE

22 Mar 2025

File Name	\\storage.york.ac.uk\chemistry\services\Chromatography\CDCSPAC\Data\20250322_BM959_CYCLOPROPYLSTD_8713ADH 2025-03-22 16-22-47\CYCLOPROPYLSTD.D\dad1A.ch
Title	LC DATA FILE
Sampling Rate	150

Sample Name Cyclopropylstd

Detector UV (254 nm)

dad1A.ch

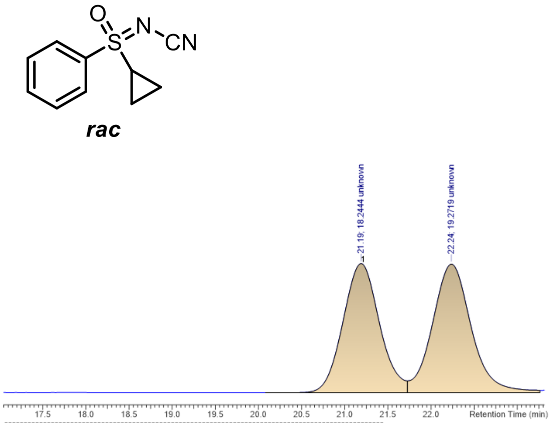

No.	Name	tR	Peak Area (Y units*ms)	Area (%)	Width
1	unknown	21.19	1094662.857	48.631	0.768
2	unknown	22.24	1156315.498	51.369	0.789

Figure S36. HPLC Data for **(*S*)-5q**

LC DATA FILE

12 Mar 2025

File Name \\storage.york.ac.uk\chemistry\services\Chromatography\CDCSHPLCAG1100\20250217_BM959_ASH_65_35_CH2PH_RECSM 2025-02-18 16-58-24\SM_REC.D\dad1A.ch

Title LC DATA FILE

Sampling Rate 150

Sample Name SM_REC

Detector UV (254 nm)

dad1A.ch

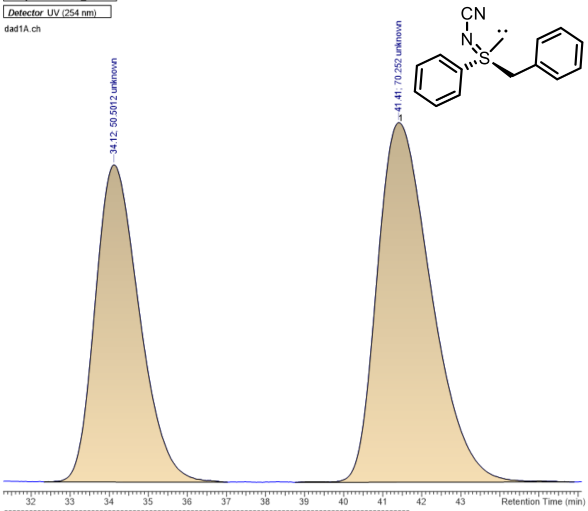

No.	Name	tR	Peak Area (Y units*ms)	Area (%)	Width
1	unknown	34.12	3030073.700	41.822	2.064
2	unknown	41.41	4215122.681	58.178	2.532

LC DATA FILE

12 Mar 2025

File Name \\storage.york.ac.uk\chemistry\services\Chromatography\CDCSHPLCAG1100\20250217_BM959_ASH_65_35_CH2PH 2025-02-18 09-58-58\SM_STD.D\dad1A.ch

Title LC DATA FILE

Sampling Rate 150

Sample Name SM_STD

Detector UV (254 nm)

dad1A.ch

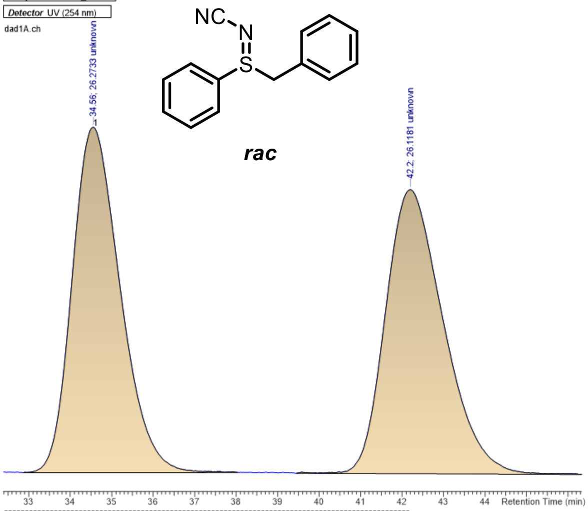

No.	Name	tR	Peak Area (Y units*ms)	Area (%)	Width
1	unknown	34.56	1576399.625	50.148	2.108
2	unknown	42.20	1567083.500	49.852	2.555

Figure S37. HPLC Data for **(*S*)-4r**

LC DATA FILE

12 Mar 2025

File Name \\storage.york.ac.uk\chemistry\services\Chromatography\CDCSHPLCAG1100\20250217_BM959_ASH_65_35_CH2PH_REC 2025-02-18 15-11-50\SO_REC.D\dad1D.ch

Title LC DATA FILE

Sampling Rate 150

Sample Name SO_REC

Detector UV (230 nm)

dad1D.ch

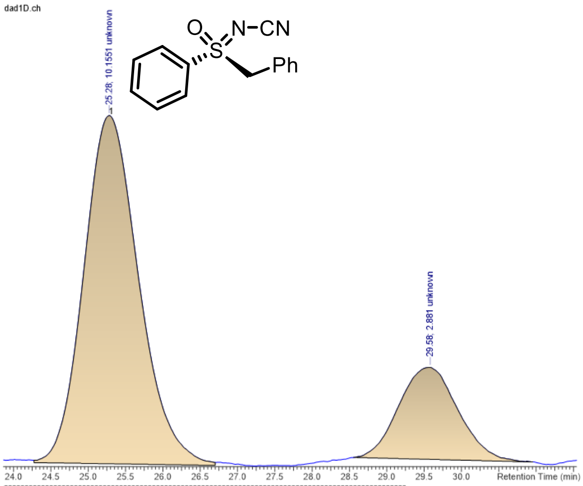

No.	Name	tR	Peak Area (Y units*ms)	Area (%)	Width
1	unknown	25.28	609308.438	77.900	1.331
2	unknown	29.58	172859.953	22.100	1.470

LC DATA FILE

12 Mar 2025

File Name \\storage.york.ac.uk\chemistry\services\Chromatography\CDCSHPLCAG1100\20250217_BM959_ASH_65_35_CH2PH_SO 2025-02-18 12-22-58\SO_STD.D\dad1D.ch

Title LC DATA FILE

Sampling Rate 150

Sample Name SO_STD

Detector UV (230 nm)

dad1D.ch

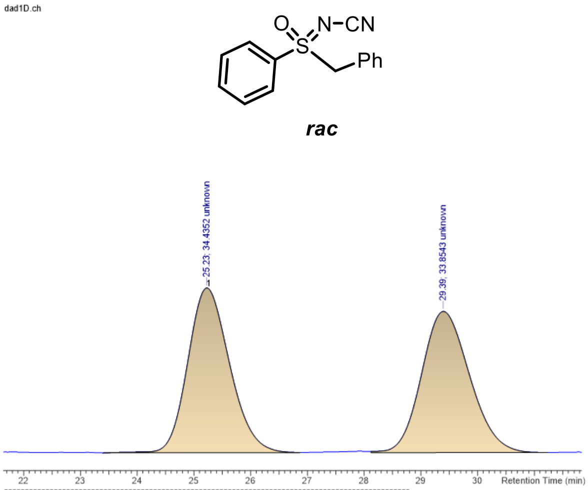

No.	Name	tR	Peak Area (Y units*ms)	Area (%)	Width
1	unknown	25.23	2066113.875	50.425	1.341
2	unknown	29.39	2031259.750	49.575	1.551

Figure S38. HPLC Data for **(*S*)-5r**

LC DATA FILE

12 Mar 2025

File Name	\\storage.york.ac.uk\chemistry\services\Chromatography\CDCSPAC\Data\20240723_BM959_523_RACE_ENZ_ASH_70_30 2024-07-23 16-30-09\523 PRODUCT.D\dad1A.ch
Title	LC DATA FILE
Sampling Rate	150

Sample Name 523 product

Detector UV (254 nm)

dad1A.ch

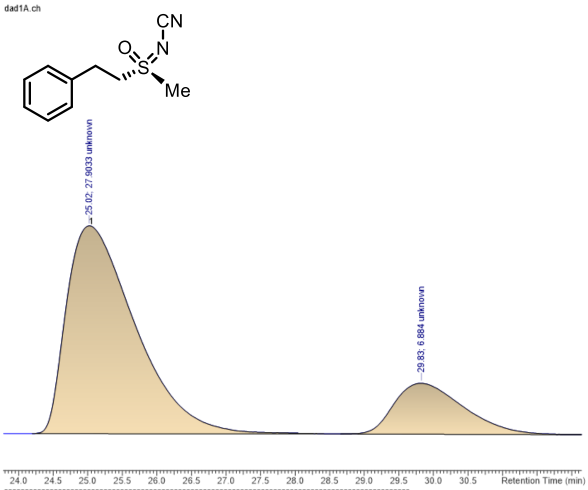

No.	Name	tR	Peak Area (Y units*ms)	Area (%)	Width
1	unknown	25.02	1674195.375	80.211	1.819
2	unknown	29.83	413037.687	19.789	1.858

LC DATA FILE

12 Mar 2025

File Name	\\storage.york.ac.uk\chemistry\services\Chromatography\CDCSPAC\Data\20240723_BM959_523_RACE_ENZ_ASH_70_30 2024-07-23 16-30-09\523 RACEMIC.D\dad1A.ch
Title	LC DATA FILE
Sampling Rate	150

Sample Name 523 racemic

Detector UV (254 nm)

dad1A.ch

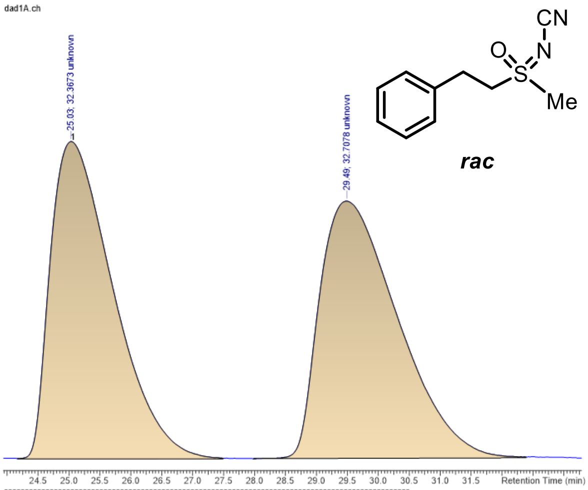

No.	Name	tR	Peak Area (Y units*ms)	Area (%)	Width
1	unknown	25.03	1942035.500	49.738	1.887
2	unknown	29.49	1962466.500	50.262	2.378

Figure S39. HPLC Data for ***(S)*-5s**

LC DATA FILE

12 Mar 2025

File Name	\\storage.york.ac.uk\chemistry\services\Chromatography\CDCSHPLCAG1100\20250304_BM959_4NO2IPR_REC_ADH_8020 2025-03-04 16-48-57\SULFILIMINE_REC.D\dad1A.ch
Title	LC DATA FILE
Sampling Rate	150

Sample Name SUlfilimine_Rec

Detector UV (254 nm)

dad1A.ch

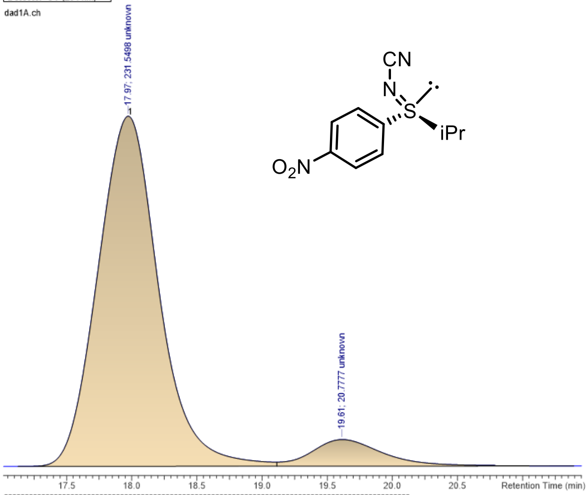

No.	Name	tR	Peak Area (Y units*ms)	Area (%)	Width
1	unknown	17.97	13892990.112	91.766	0.834
2	unknown	19.61	1246664.200	8.234	0.951

LC DATA FILE

12 Mar 2025

File Name	\\storage.york.ac.uk\chemistry\services\Chromatography\CDCSHPLCAG1100\20250304_BM959_4NO2IPR_ADH_8020 2025-03-04 14-36-06\SULFILIMINE_STD.D\dad1A.ch
Title	LC DATA FILE
Sampling Rate	150

Sample Name Sulfilimine_STD

Detector UV (254 nm)

dad1A.ch

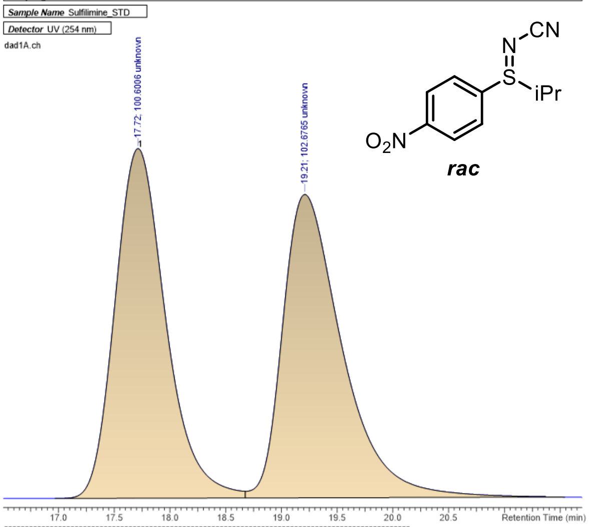

No.	Name	tR	Peak Area (Y units*ms)	Area (%)	Width
1	unknown	17.72	6036034.241	49.489	0.813
2	unknown	19.21	6160592.194	50.511	0.933

Figure S40. HPLC trace for **(*S*)-4t**

LC DATA FILE

12 Mar 2025

File Name	\\storage.york.ac.uk\chemistry\services\Chromatography\CDCSHPLCAG1100\20250304_BM959_4NO2IPR_REC_ADH_8020 2025-03-04 16-48-57\SULFOXIMINE_REC.D\dad1A.ch
Title	LC DATA FILE
Sampling Rate	150

Sample Name SUlfoximine_Rec

Detector UV (254 nm)

dad1A.ch

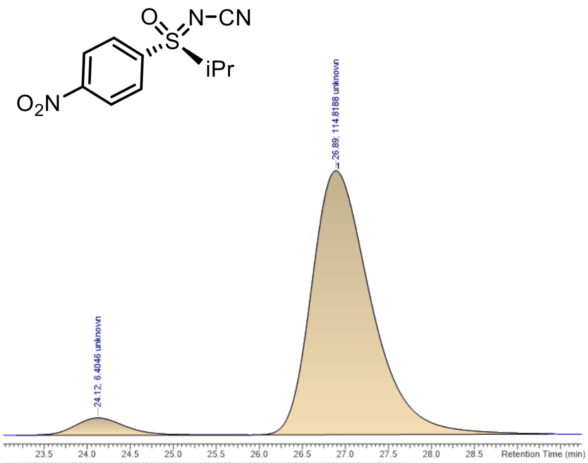

No.	Name	tR	Peak Area (Y units*ms)	Area (%)	Width
1	unknown	24.12	384276.352	5.283	1.048
2	unknown	26.89	6889129.486	94.717	1.221

LC DATA FILE

12 Mar 2025

File Name	\\storage.york.ac.uk\chemistry\services\Chromatography\CDCSHPLCAG1100\20250304_BM959_4NO2IPR_ADH_8020 2025-03-04 14-36-06\SULFOXIMINE_STD.D\dad1A.ch
Title	LC DATA FILE
Sampling Rate	150

Sample Name SUlfoximine_STD

Detector UV (254 nm)

dad1A.ch

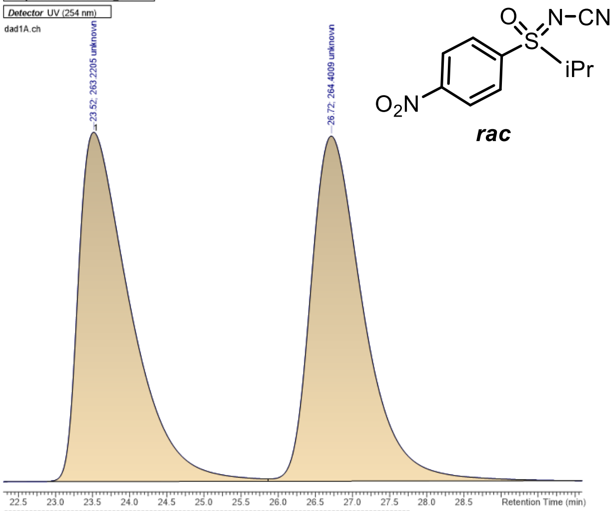

No.	Name	tR	Peak Area (Y units*ms)	Area (%)	Width
1	unknown	23.52	15793229.370	49.888	1.221
2	unknown	26.72	15864052.734	50.112	1.234

Figure S41. HPLC trace for **(*S*)-5t**

LC DATA FILE

16 Mar 2025

**File Name**	\\storage.york.ac.uk\chemistry\services\Chromatography\CDCSPAC\Data\250313_BM959_CF3ET_ASH7030 2025-03-13 16-36-42\ENZ_SM.D\dad1A.ch
**Title**	LC DATA FILE
**Detector**	UV (254 nm)
**UV Trace Bandwidth**	4 nm

dad1A.ch

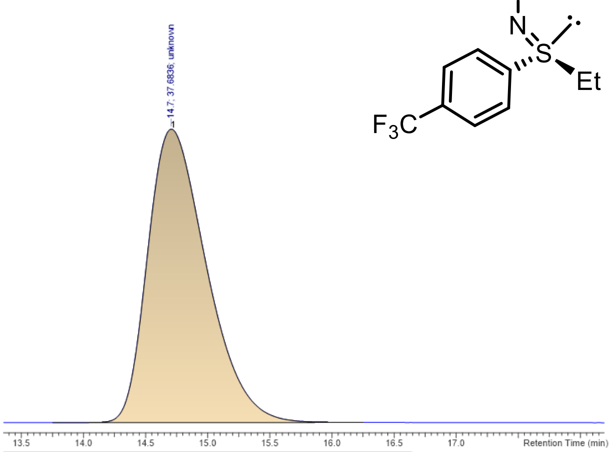

No.	Name	tR	Peak Area (Y units*ms)	Area (%)	Width
1	unknown	14.70	2261018.600	100.000	0.866

LC DATA FILE

17 Mar 2025

**File Name**	\\storage.york.ac.uk\chemistry\services\Chromatography\CDCSPAC\Data\250314_BM959_4CF3ETHYLSMPAPER(1)_ASH7030 2025-03-17 10-04-40\STARTING.D\dad1A.ch
**Title**	LC DATA FILE
**Sampling Rate**	150
**Sample Name**	Starting
**Detector**	UV (254 nm)

dad1A.ch

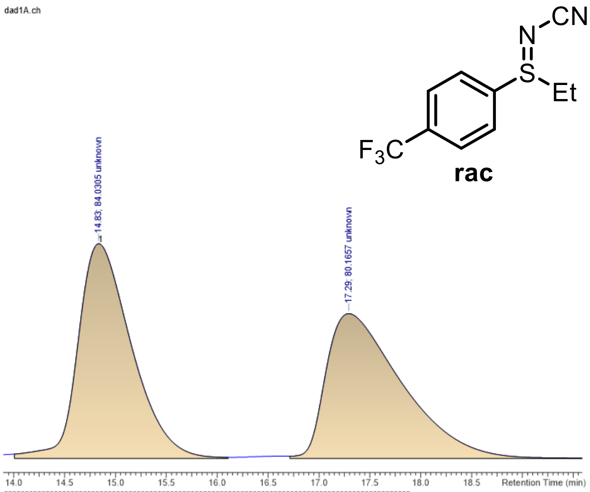

No.	Name	tR	Peak Area (Y units*ms)	Area (%)	Width
1	unknown	14.83	5041831.055	51.177	0.934
2	unknown	17.29	4809944.916	48.823	1.346

Figure S42. HPLC Data for **(*S*)-4u**

LC DATA FILE

16 Mar 2025

**File Name**	\\storage.york.ac.uk\chemistry\services\Chromatography\CDCSPAC\Data\250313_BM959_CF3ET_ASH7030 2025-03-13 16-36-42\ENZ_SO.D\dad1E.ch
**Title**	LC DATA FILE
**Detector**	UV (280 nm)
**UV Trace Bandwidth**	16 nm

dad1E.ch

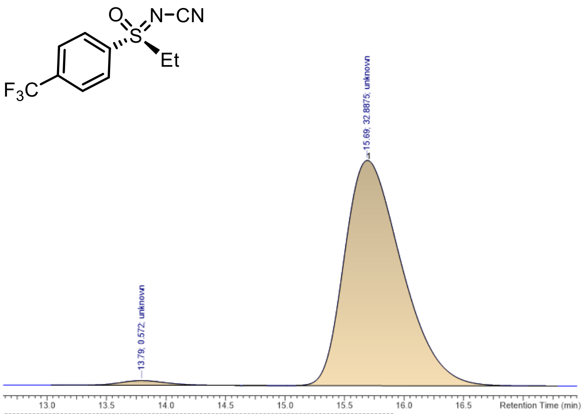

No.	Name	tR	Peak Area (Y units*ms)	Area (%)	Width
1	unknown	13.79	34319.057	1.709	0.705
2	unknown	15.69	1973251.190	98.291	0.881

LC DATA FILE

16 Mar 2025

**File Name**	\\storage.york.ac.uk\chemistry\services\Chromatography\CDCSPAC\Data\250313_BM959_CF3ET_ASH7030 2025-03-13 16-36-42\SO-STD.D\dad1E.ch
**Title**	LC DATA FILE
**Detector**	UV (280 nm)
**UV Trace Bandwidth**	16 nm

dad1E.ch

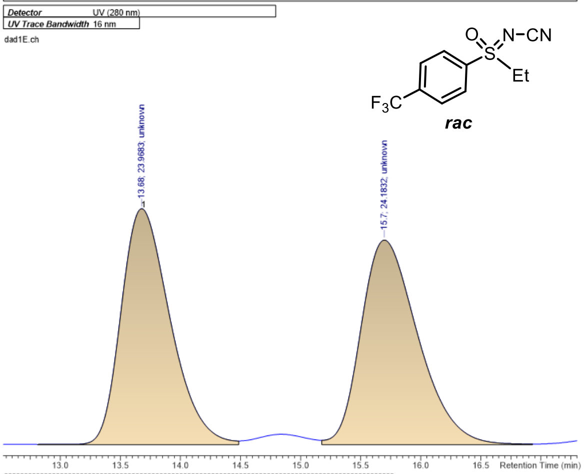

No.	Name	tR	Peak Area (Y units*ms)	Area (%)	Width
1	unknown	13.68	1438095.703	49.777	0.744
2	unknown	15.70	1450989.304	50.223	0.864

Figure S43. HPLC Data for **(*S*)-5u**

LC DATA FILE

22 Aug

File Name	\\storage.york.ac.uk\chemistry\services\Chromatography\CDCSPAC\Data\11042024JDG558_ 230R 2024-04-11 09-15-33\001-0101.D\dad1A.ch
Type	HPLC
Points Count	3854
Date Stamp	11-Apr-24, 09:16:34
Date	11 Apr 2024 09:42:20
Title	LC DATA FILE
Comment	230-r
Sampling Rate	150
Detector Lag	0
Short File Name	dad1A.ch
Sample Data	230-r
Instrument	UV detector (254 nm)Column

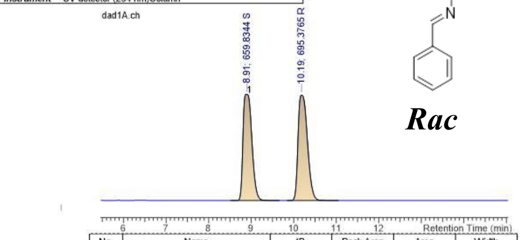

No.	Name	tR	Peak Area (Y units*ms)	Area (%)	Width
1	S	8.91	39590064.697	48.689	0.383
2	R	10.19	41722587.891	51.311	0.406

LC DATA FILE

22 Aug 2024

File Name	C:\PROGRAM FILES\ACD64\004-0101_1
Type	HPLC
Points Count	3345
Date Stamp	11-Apr-24, 09:45:44
Date	11 Apr 2024 10:08:06
Title	LC DATA FILE
Comment	230-2
Sampling Rate	150
Internal Name	004-0101_1
Detector Lag	0
Short File Name	004-0101_1
Sample Data	230-2
Instrument	UV detector (254 nm)Column

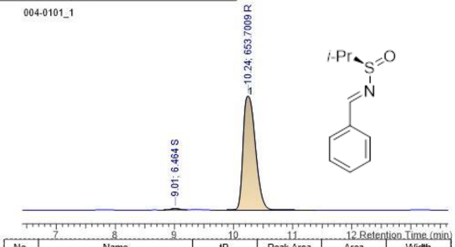

No.	Name	tR	Peak Area (Y units*ms)	Area (%)	Width
1	S	9.01	387839.969	0.979	0.279
2	R	10.24	39222056.000	99.021	0.387

Figure S44. HPLC Data for ***(R)*-12a**

LC DATA FILE

23 Aug 2024

File Name	\\storage.york.ac.uk\chemistry\services\Chromatography\CDCSPAC\Data\20240614-JDG558_HEX95_IPA5_IBN5 280-1 2024-06-14 15-33-06\001-0101.D\dad1A.ch
Type	HPLC
Points Count	2557
Date Stamp	14-Jun-24, 15:34:11
Date	14 Jun 2024 15:51:16
Title	LC DATA FILE
Comment	280-1
Sampling Rate	150
Detector Lag	0
Short File Name	dad1A.ch
Sample Data	280-1
Instrument	UV detector (254 nm)Column

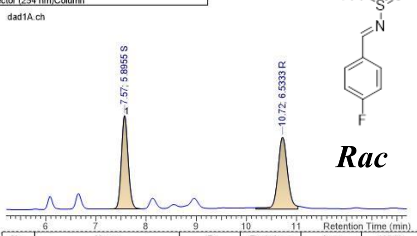

No.	Name	tR	Peak Area (Y units*ms)	Area (%)	Width
1	S	7.57	353731.213	47.434	0.242
2	R	10.72	391997.023	52.566	0.336

LC DATA FILE

23 Aug 2024

Name	C:\PROGRAM FILES\ACD64\003-0301_1
	HPLC
ts Count	3752
Stamp	14-Jun-24, 14:38:54
	14 Jun 2024 15:03:58
	LC DATA FILE
ment	278-1
pling Rate	150
nal Name	003-0301_1
ctor Lag	0
t File Name	003-0301_1
ple Data	278-1
ument	UV detector (254 nm)Column

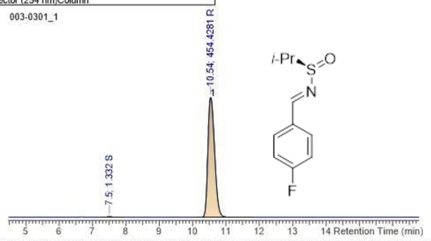

No.	Name	tR	Peak Area (Y units*ms)	Area (%)	Width
1	S	7.50	79921.461	0.292	0.238
2	R	10.54	27265688.000	99.708	0.368

Figure S45. HPLC Data for ***(R)*-12b**

LC DATA FILE

22 Aug 2024

File Name	\\storage.york.ac.uk\chemistry\services\Chromatography\CDCSPAC\Data\20240509_JDG558_256 RACEMIC 2024-05-10 08-19-07\001-0101.D\dad1D.ch
Type	HPLC
Points Count	4501
Date Stamp	10-May-24, 08:20:17
Date	10 May 2024 08:50:24
Title	LC DATA FILE
Comment	256-1
Sampling Rate	150
Detector Lag	0
Short File Name	dad1D.ch
Sample Data	256-1
Instrument	UV detector (230 nm)Column

dad1D.ch

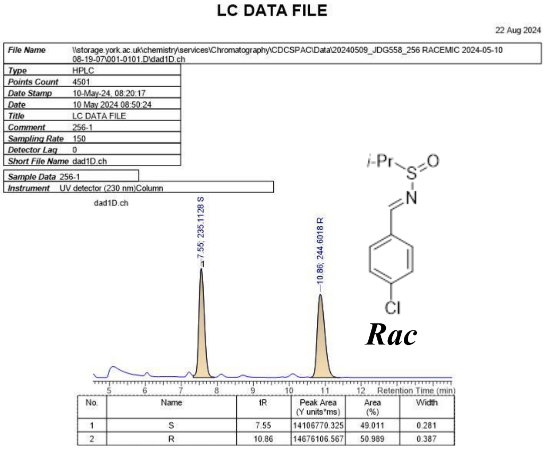

No.	Name	tR	Peak Area (Y units*ms)	Area (%)	Width
1	S	7.55	14106770.325	49.011	0.281
2	R	10.86	14676106.567	50.989	0.387

LC DATA FILE

22 Aug 2024

File Name	\\storage.york.ac.uk\chemistry\services\Chromatography\CDCSPAC\Data\20240509_JDG558_255 CHIRAL 2024-05-10 09-28-16\003-0101.D\dad1D.ch
Type	HPLC
Points Count	4501
Date Stamp	10-May-24, 09:29:17
Date	10 May 2024 09:59:24
Title	LC DATA FILE
Comment	255-1
Sampling Rate	150
Detector Lag	0
Short File Name	dad1D.ch
Sample Data	255-1
Instrument	UV detector (230 nm)Column

dad1D.ch

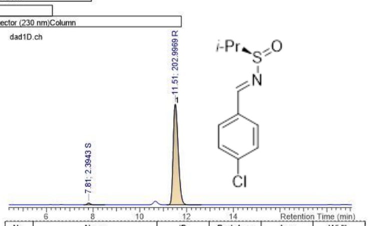

No.	Name	tR	Peak Area (Y units*ms)	Area (%)	Width
1	S	7.81	143660.344	1.166	0.249
2	R	11.51	12179812.000	98.834	0.392

Figure S46. HPLC Data for **(*R*)-12c**

LC DATA FILE

23 Aug 2024

File Name	\\storage.york.ac.uk\chemistry\services\Chromatography\CDCSPAC\Data\20240614-JDG558_HEX95_IPA5_IBN5 278-280 2024-06-14 13-45-23\002-0201.D\dad1A.ch
Type	HPLC
Points Count	3752
Date Stamp	14-Jun-24, 14:12:38
Date	14 Jun 2024 14:37:42
Title	LC DATA FILE
Comment	280-2
Sampling Rate	150
Detector Lag	0
Short File Name	dad1A.ch
Sample Data	280-2
Instrument	UV detector (254 nm)Column

dad1A.ch

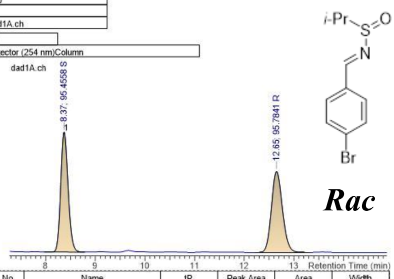

No.	Name	tR	Peak Area (Y units*ms)	Area (%)	Width
1	S	8.37	5727347.717	49.914	0.270
2	R	12.65	5747046.661	50.086	0.402

LC DATA FILE

23 Aug 2024

File Name	\\storage.york.ac.uk\chemistry\services\Chromatography\CDCSPAC\Data\20240614-JDG558_HEX95_IPA5_IBN5 278-280 2024-06-14 13-45-23\004-0401.D\dad1A.ch
Type	HPLC
Points Count	3751
Date Stamp	14-Jun-24, 15:05:09
Date	14 Jun 2024 15:30:12
Title	LC DATA FILE
Comment	278-2
Sampling Rate	150
Detector Lag	0
Short File Name	dad1A.ch
Sample Data	278-2
Instrument	UV detector (254 nm)Column

dad1A.ch

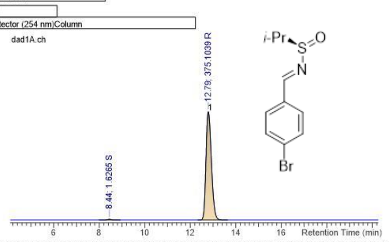

No.	Name	tR	Peak Area (Y units*ms)	Area (%)	Width
1	S	8.44	97592.311	0.432	0.273
2	R	12.79	22506236.572	99.568	0.433

Figure S47. HPLC Data for **(*R*)-12d**

LC DATA FILE

23 Aug 2024

File Name	C:\PROGRAM FILES\ACD64\002-0201_1
Type	HPLC
Points Count	3751
Date Stamp	12-Jun-24, 13:38:59
Date	12 Jun 2024 14:04:04
Title	LC DATA FILE
Comment	276-2-ibn5-95/5
Sampling Rate	150
Internal Name	002-0201_1
Detector Lag	0
Short File Name	002-0201_1
Sample Data	276-2-ibn5-95/5
Instrument	UV detector (254 nm)Column

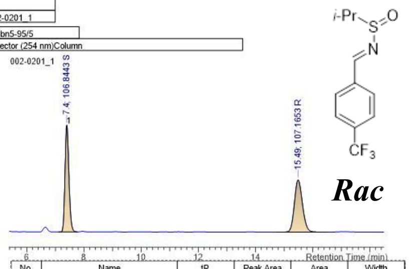

No.	Name	tR	Peak Area (Y units*ms)	Area (%)	Width
1	S	7.40	6410659.332	49.925	0.247
2	R	15.49	6429920.197	50.075	0.511

LC DATA FILE

23 Aug 2024

File Name	\\storage.york.ac.uk\chemistry\services\Chromatography\CDCSPAC\Data\10042024JDG558_ 274-276-IBNN5-95-5 2024-06-12 13-11-15\004-0401.D\dad1A.ch
Type	HPLC
Points Count	3751
Date Stamp	12-Jun-24, 14:32:25
Date	12 Jun 2024 14:57:28
Title	LC DATA FILE
Comment	275-2ibn5-95/5
Sampling Rate	150
Detector Lag	0
Short File Name	dad1A.ch
Sample Data	275-2ibn5-95/5
Instrument	UV detector (254 nm)Column

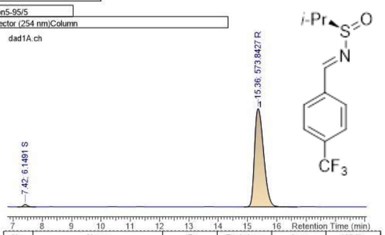

No.	Name	tR	Peak Area (Y units*ms)	Area (%)	Width
1	S	7.42	368944.188	1.060	0.241
2	R	15.36	34430560.000	98.940	0.571

Figure S48. HPLC Data for **(*R*)-12e**

LC DATA FILE

22 Aug 2024

File Name	\\storage.york.ac.uk\chemistry\services\Chromatography\CDCSPAC\Data\20240503_JDG253 AND 244-IBN5 2024-05-03 14-04-57\021-0101.D\dad1A.ch
Type	HPLC
Points Count	3035
Date Stamp	03-May-24, 14:06:01
Date	03 May 2024 14:26:16
Title	LC DATA FILE
Comment	JDG-253-1
Sampling Rate	150
Detector Lag	0
Short File Name	dad1A.ch
Sample Data	JDG-253-1
Instrument	UV detector (254 nm)Column

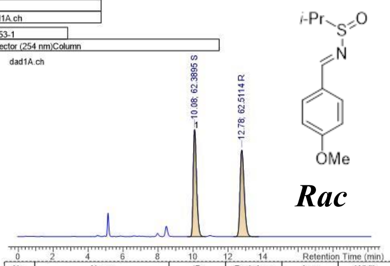

No.	Name	tR	Peak Area (Y units*ms)	Area (%)	Width
1	S	10.08	3743367.691	49.951	0.345
2	R	12.78	3750686.417	50.049	0.428

LC DATA FILE

22 Aug 2024

File Name	\\storage.york.ac.uk\chemistry\services\Chromatography\CDCSPAC\Data\20240503_JDG253 AND 244-1IBN5 2024-05-03 14-30-34\022-0101.D\dad1B.ch
Type	HPLC
Points Count	3751
Date Stamp	03-May-24, 14:31:37
Date	03 May 2024 14:56:40
Title	LC DATA FILE
Comment	JDG-253-2
Sampling Rate	150
Detector Lag	0
Short File Name	dad1B.ch
Sample Data	JDG-253-2
Instrument	UV detector (254 nm)Column

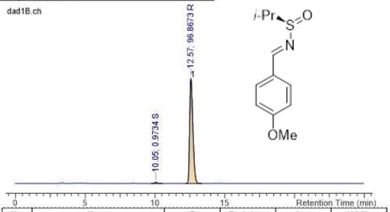

No.	Name	tR	Peak Area (Y units*ms)	Area (%)	Width
1	S	10.05	58404.350	0.995	0.321
2	R	12.57	5812037.201	99.005	0.419

Figure S49. HPLC Data for **(*R*)-12f**

LC DATA FILE

23 Aug 2024

File Name	\\storage.york.ac.uk\chemistry\services\Chromatography\CDCSPAC\Data\10042024JDG558_ 274-276-IBNN5-95-5 2024-06-12 13-11-15\001-0101.D\dad1A.ch
Type	HPLC
Points Count	3751
Date Stamp	12-Jun-24, 13:12:17
Date	12 Jun 2024 13:37:22
Title	LC DATA FILE
Comment	276-1-ibn5-95/5
Sampling Rate	150
Detector Lag	0
Short File Name	dad1A.ch
Sample Data	276-1-ibn5-95/5
Instrument	UV detector (254 nm)Column

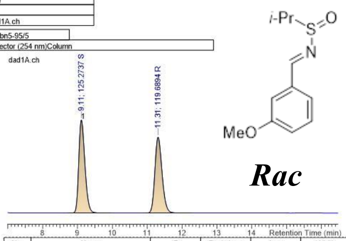

No.	Name	tR	Peak Area (Y units*ms)	Area (%)	Width
1	S	9.11	7516419.525	51.140	0.317
2	R	11.31	7181361.694	48.860	0.371

LC DATA FILE

23 Aug 2024

File Name	\\storage.york.ac.uk\chemistry\services\Chromatography\CDCSPAC\Data\10042024JDG558_ 274-276-IBNN5-95-5 2024-06-12 13-11-15\003-0301.D\dad1A.ch
Type	HPLC
Points Count	3751
Date Stamp	12-Jun-24, 14:05:42
Date	12 Jun 2024 14:30:46
Title	LC DATA FILE
Comment	274-2ibn5-95/5
Sampling Rate	150
Detector Lag	0
Short File Name	dad1A.ch
Sample Data	274-2ibn5-95/5
Instrument	UV detector (254 nm)Column

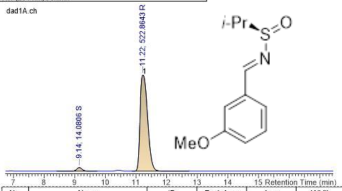

No.	Name	tR	Peak Area (Y units*ms)	Area (%)	Width
1	S	9.14	844836.330	2.622	0.311
2	R	11.22	31371859.131	97.378	0.451

Figure S50. HPLC Data for **(*R*)-12g**

LC DATA FILE

23 Aug 2024

File Name	C:\PROGRAM FILES\ACD64\001-0101_1
Type	HPLC
Points Count	4501
Date Stamp	18-Jun-24, 15:58:45
Date	18 Jun 2024 16:28:50
Title	LC DATA FILE
Comment	281-RAC
Sampling Rate	150
Internal Name	001-0101_1
Detector Lag	0
Short File Name	001-0101_1
Sample Data	281-RAC
Instrument	UV detector (254 nm)Column

001-0101_1

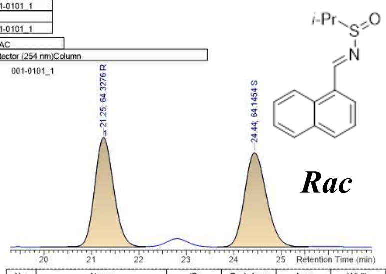

No.	Name	tR	Peak Area (Y units*ms)	Area (%)	Width
1	R	21.25	3859653.750	50.071	0.726
2	S	24.44	3848723.500	49.929	0.844

LC DATA FILE

23 Aug 2024

File Name	C:\PROGRAM FILES\ACD64\002-0101_1
Type	HPLC
Points Count	4501
Date Stamp	18-Jun-24, 16:30:59
Date	18 Jun 2024 17:01:04
Title	LC DATA FILE
Comment	281-2
Sampling Rate	150
Internal Name	002-0101_1
Detector Lag	0
Short File Name	002-0101_1
Sample Data	281-2
Instrument	UV detector (254 nm)Column

002-0101_1

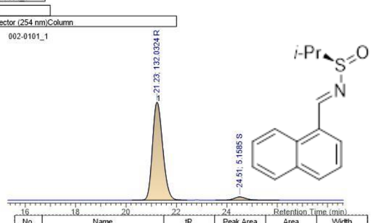

No.	Name	tR	Peak Area (Y units*ms)	Area (%)	Width
1	R	21.23	7921943.000	96.240	0.743
2	S	24.51	309512.094	3.760	0.859

Figure S51. HPLC Data for **(*R*)-12h**

LC DATA FILE

23 Aug 2024

File Name	C:\PROGRAM FILES\ACD64\20240514_SAH_THIOLRACEMIC 80_20_1
Type	HPLC
Points Count	4501
Date Stamp	17-May-24, 13:47:41
Date	17 May 2024 14:17:44
Title	LC DATA FILE
Comment	20240517JDG265-1
Sampling Rate	150
Internal Name	20240514_SAH_THIOLRACEMIC 80_20_1
Detector Lag	0
Short File Name	20240514_SAH_THIOLRACEMIC 80_20_1
Sample Data	20240517JDG265-1
Instrument	UV detector (254 nm)Column

20240514_SAH_THIOLRACEMIC 80_20_1

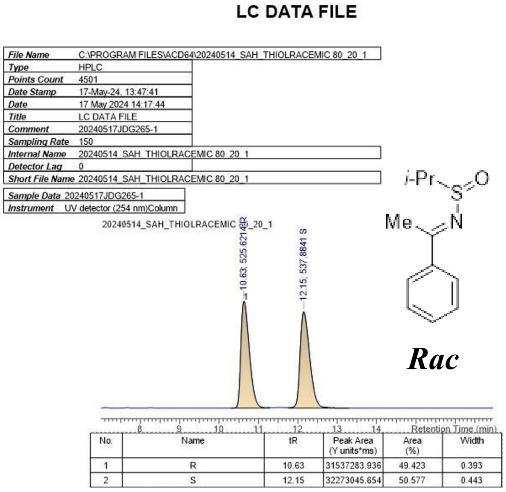

No.	Name	tR	Peak Area (Y units*ms)	Area (%)	Width
1	R	10.63	31537283.936	49.423	0.393
2	S	12.15	32273045.654	50.577	0.443

LC DATA FILE

23 Aug 2024

File Name	\\storage.york.ac.uk\chemistry\services\Chromatography\CDCSPAC\Data\20240517_JDG_260-2 AND 264-2 2024-05-17 15-27-14\20240514_SAH_THIOLRACEMIC 80_20.D\dad1A.ch
Type	HPLC
Points Count	2251
Date Stamp	17-May-24, 15:28:18
Date	17 May 2024 15:43:22
Title	LC DATA FILE
Comment	20240517JDG260-2
Sampling Rate	150
Detector Lag	0
Short File Name	dad1A.ch
Sample Data	20240517JDG260-2
Instrument	UV detector (254 nm)Column

dad1A.ch

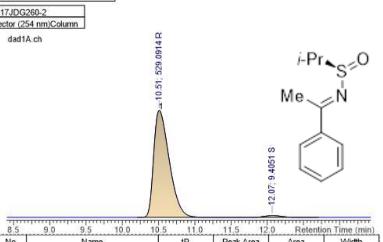

No.	Name	tR	Peak Area (Y units*ms)	Area (%)	Width
1	S	12.07	564308.681	1.747	0.392
2	R	10.51	31745482.178	98.253	0.392

Figure S52. HPLC Data for **(*R*)-12i**

LC DATA FILE

23 Aug 2024

File Name	\\storage.york.ac.uk\chemistry\services\Chromatography\CDCSPAC\Data\20240808_JDG558_H95_IPA5_IBN5_340R 2024-08-08 12-26-11\20240807_SAH_KS-2-074.D\dad1A.ch
Type	HPLC
Points Count	4309
Date Stamp	08-Aug-24, 12:27:14
Date	08 Aug 2024 12:56:02
Title	LC DATA FILE
Comment	20240807_JDG_340R_IBN5955
Sampling Rate	150
Detector Lag	0
Short File Name	dad1A.ch
Sample Data	20240807_JDG_340R_IBN5955
Instrument	UV detector (254 nm)Column

dad1A.ch

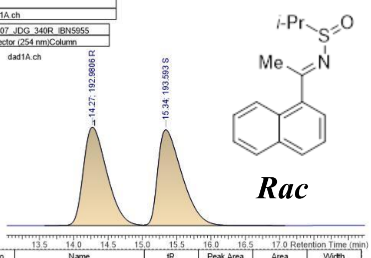

No.	Name	tR	Peak Area (Y units*ms)	Area (%)	Width
1	R	14.27	11578836.365	49.921	0.628
2	S	15.34	11615579.224	50.079	0.643

LC DATA FILE

23 Aug 2024

File Name	C:\PROGRAM FILES\ACD64\20240807_SAH_KS-2-074_1
Type	HPLC
Points Count	3723
Date Stamp	08-Aug-24, 12:58:10
Date	08 Aug 2024 13:23:04
Title	LC DATA FILE
Comment	20240807_JDG_340E_IBN5955
Sampling Rate	150
Internal Name	20240807_SAH_KS-2-074_1
Detector Lag	0
Short File Name	20240807_SAH_KS-2-074_1
Sample Data	20240807_JDG_340E_IBN5955
Instrument	UV detector (254 nm)Column

20240807_SAH_KS-2-074_1

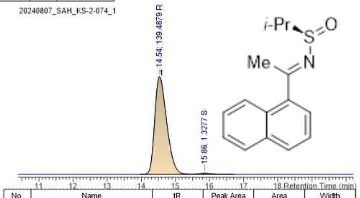

No.	Name	tR	Peak Area (Y units*ms)	Area (%)	Width
1	S	15.86	79664.819	0.943	0.590
2	R	14.54	8369276.733	99.057	0.641

Figure S53. HPLC Data for **(*R*)-12j**

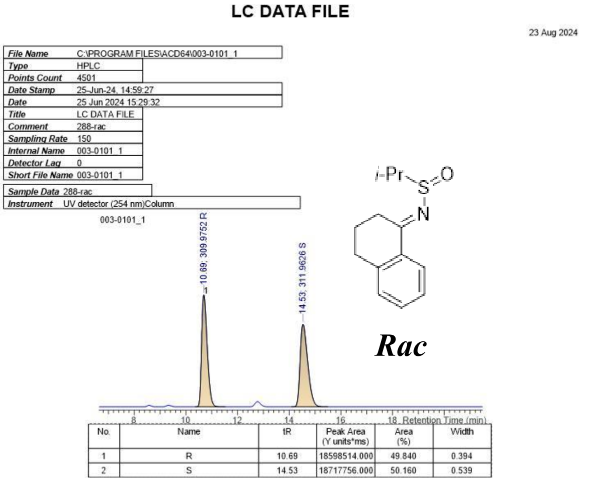

No.	Name	tR	Peak Area (Y units*ms)	Area (%)	Width
1	R	10.69	18598514.000	49.840	0.394
2	S	14.53	18717756.000	50.160	0.539

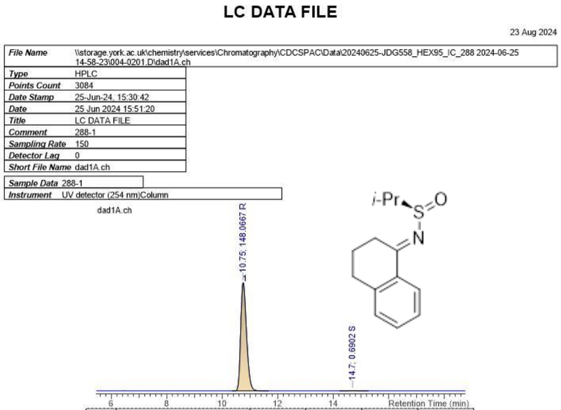

No.	Name	tR	Peak Area (Y units*ms)	Area (%)	Width
1	R	10.75	8884003.000	99.536	0.378
2	S	14.70	41411.766	0.464	0.502

Figure S54. HPLC Data for **(*R*)-12k**

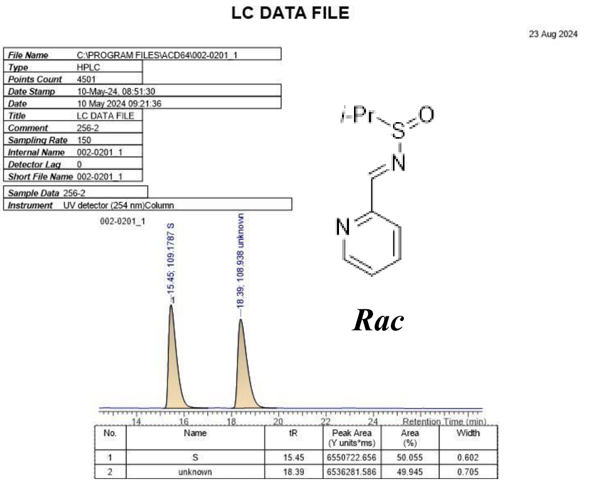

No.	Name	tR	Peak Area (Y units*ms)	Area (%)	Width
1	S	15.45	6550722.656	50.055	0.602
2	unknown	18.39	6536281.586	49.945	0.705

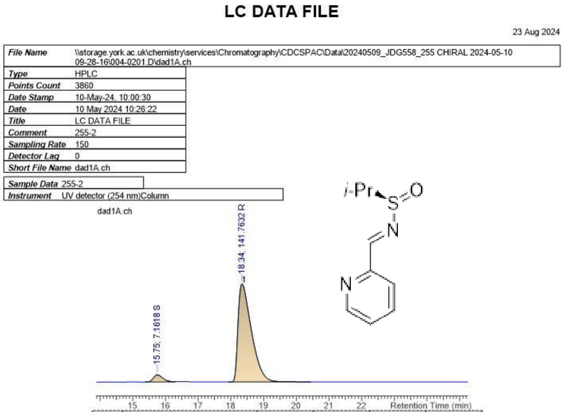

No.	Name	tR	Peak Area (Y units*ms)	Area (%)	Width
1	S	15.75	429709.597	4.809	0.520
2	R	18.34	8505791.931	95.191	0.741

Figure S55. HPLC Data for **(*R*)-12l**

LC DATA FILE

26 Aug 2024

File Name	\\storage.york.ac.uk\chemistry\services\Chromatography\CDCSPAC\Data\20240501_JDG246-2 2024-05-01 15-04-00\011-0101.D\dad1A.ch
Type	HPLC
Points Count	3751
Date Stamp	01-May-24, 15:05:04
Date	01 May 2024 15:30:10
Title	LC DATA FILE
Comment	jdg-246-r
Sampling Rate	150
Detector Lag	0
Short File Name	dad1A.ch
Sample Data	jdg-246-r
Instrument	UV detector (254 nm)Column

dad1A.ch

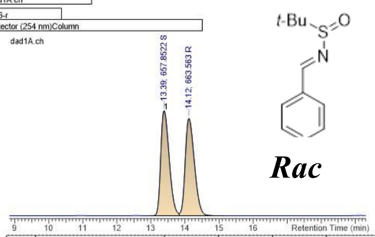

No.	Name	tR	Peak Area (Y units*ms)	Area (%)	Width
1	S	13.39	39471130.371	49.784	0.456
2	R	14.12	39813782.959	50.216	0.494

LC DATA FILE

26 Aug 2024

File Name	C:\windows\system32\021-0101_1
Type	HPLC
Points Count	3751
Date Stamp	01-May-24, 15:54:33
Date	01 May 2024 16:19:40
Title	LC DATA FILE
Comment	jdg-246-2
Sampling Rate	150
Internal Name	021-0101_1
Detector Lag	0
Short File Name	021-0101_1
Sample Data	jdg-246-2
Instrument	UV detector (254 nm)Column

021-0101_1

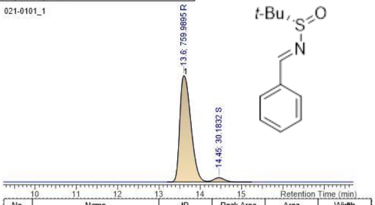

No.	Name	tR	Peak Area (Y units*ms)	Area (%)	Width
1	S	14.45	1810994.568	3.820	0.476
2	R	13.60	45599370.117	96.180	0.493

Figure S56. HPLC Data for **(*S*)-13a**

LC DATA FILE

26 Aug 2024

File Name	\\storage.york.ac.uk\chemistry\services\Chromatography\CDCSPAC\Data\20240717_JDG558_300-303 RAC_IC 2024-07-17 12-29-00\001-0101.D\dad1A.ch
Type	HPLC
Points Count	4501
Date Stamp	17-Jul-24, 12:30:01
Date	17 Jul 2024 13:00:06
Title	LC DATA FILE
Comment	LJC-300-RAC
Sampling Rate	150
Detector Lag	0
Short File Name	dad1A.ch
Sample Data	LJC-300-RAC
Instrument	UV detector (254 nm)Column

dad1A.ch

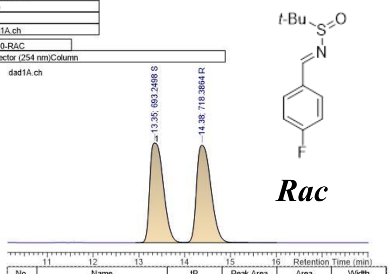

No.	Name	tR	Peak Area (Y units*ms)	Area (%)	Width
1	S	13.35	41594985.352	49.110	0.552
2	R	14.38	43103181.152	50.890	0.581

LC DATA FILE

26 Aug 2024

File Name	C:\windows\system32\ENZYMATIC SAMPLE_1
Type	HPLC
Points Count	3064
Date Stamp	17-Jul-24, 13:58:02
Date	17 Jul 2024 14:18:30
Title	LC DATA FILE
Comment	LJC-300-ENY
Sampling Rate	150
Internal Name	ENZYMATIC SAMPLE_1
Detector Lag	0
Short File Name	ENZYMATIC SAMPLE_1
Sample Data	LJC-300-ENY
Instrument	UV detector (254 nm)Column

ENZYMATIC SAMPLE_1

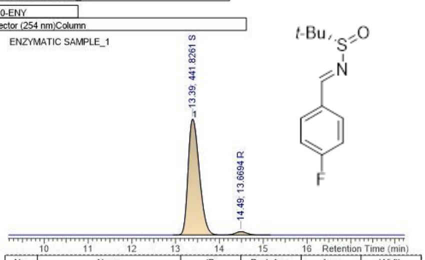

No.	Name	tR	Peak Area (Y units*ms)	Area (%)	Width
1	S	13.39	26509568.481	96.999	0.470
2	R	14.49	820163.097	3.001	0.483

Figure S57. HPLC Data for **(*S*)-13b**

LC DATA FILE

26 Aug 2024

File Name	C:\windows\system32\001-0101_1
Type	HPLC
Points Count	4018
Date Stamp	04-Jul-24, 13:57:35
Date	04 Jul 2024 14:24:28
Title	LC DATA FILE
Comment	297-RAC
Sampling Rate	150
Internal Name	001-0101_1
Detector Lag	0
Short File Name	001-0101_1
Sample Data	297-RAC
Instrument	UV detector (254 nm)Column

001-0101_1

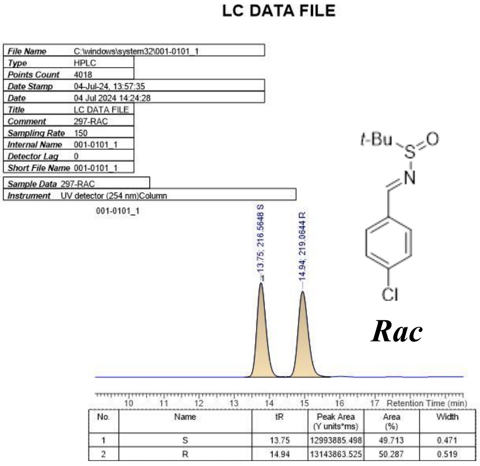

No.	Name	tR	Peak Area (Y units*ms)	Area (%)	Width
1	S	13.75	12993885.498	49.713	0.471
2	R	14.94	13143863.525	50.287	0.519

LC DATA FILE

26 Aug 2024

File Name	\\storage.york.ac.uk\chemistry\services\Chromatography\CDCSPAC\Data\20240704-JDG558_HEX95_IC_297-RAC 2024-07-04 13-30-05\4-CN-SO_ENZY.D\dad1A.ch
Type	HPLC
Points Count	3498
Date Stamp	04-Jul-24, 13:31:05
Date	04 Jul 2024 13:54:30
Title	LC DATA FILE
Comment	297-rac
Sampling Rate	150
Detector Lag	0
Short File Name	dad1A.ch
Sample Data	297-rac
Instrument	UV detector (254 nm)Column

dad1A.ch

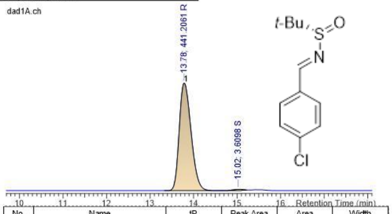

No.	Name	tR	Peak Area (Y units*ms)	Area (%)	Width
1	S	15.02	216590.469	0.812	0.486
2	R	13.78	26472368.000	99.188	0.499

Figure S58. HPLC Data for **(*S*)-13c**

LC DATA FILE

26 Aug 2024

File Name	C:\windows\system32\003-0101_1
Type	HPLC
Points Count	4502
Date Stamp	29-Jul-24, 10:56:21
Date	29 Jul 2024 11:26:26
Title	LC DATA FILE
Comment	316-1
Sampling Rate	150
Internal Name	003-0101_1
Detector Lag	0
Short File Name	003-0101_1
Sample Data	316-1
Instrument	UV detector (254 nm)Column

003-0101_1

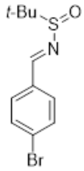

Rac

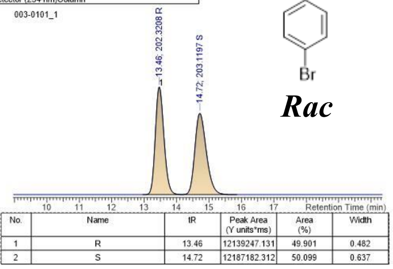

No.	Name	tR	Peak Area (Y units*ms)	Area (%)	Width
1	R	13.46	12139247.131	49.901	0.482
2	S	14.72	12187182.312	50.099	0.637

LC DATA FILE

26 Aug 2024

File Name	\\storage.york.ac.uk\chemistry\services\Chromatography\CDCSPAC\Data\20240729_JDG558_HEX95_IPA5_IC-315 2024-07-29 12-29-46\006-0101.D\dad1A.ch
Type	HPLC
Points Count	3001
Date Stamp	29-Jul-24, 12:30:49
Date	29 Jul 2024 12:50:54
Title	LC DATA FILE
Comment	315-1
Sampling Rate	150
Detector Lag	0
Short File Name	dad1A.ch
Sample Data	315-1
Instrument	UV detector (254 nm)Column

dad1A.ch

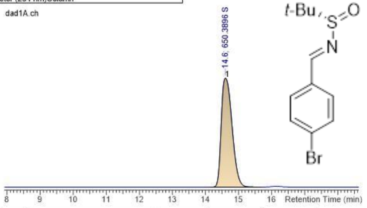

No.	Name	tR	Peak Area (Y units*ms)	Area (%)	Width
1	S	14.60	39023378.906	100.000	0.606

Figure S59. HPLC Data for **(*S*)-13d**

LC DATA FILE

26 Aug 2024

File Name	\\storage.york.ac.uk\chemistry\services\Chromatography\CDCSPAC\Data\20240729_JDG558_HEX95_IPA5_IC 2024-07-29 10-55-19\004-0201.D\dad1A.ch
Type	HPLC
Points Count	4501
Date Stamp	29-Jul-24, 11:27:37
Date	29 Jul 2024 11:57:42
Title	LC DATA FILE
Comment	316-2
Sampling Rate	150
Detector Lag	0
Short File Name	dad1A.ch
Sample Data	316-2
Instrument	UV detector (254 nm)Column

dad1A.ch

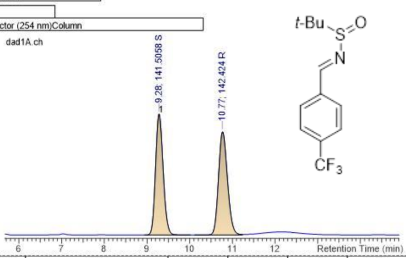

No.	Name	tR	Peak Area (Y units*ms)	Area (%)	Width
1	S	9.28	8490349.731	49.838	0.323
2	R	10.77	8545437.927	50.162	0.382

LC DATA FILE

26 Aug 2024

File Name	C:\windows\system32\007-0201_1
Type	HPLC
Points Count	2162
Date Stamp	29-Jul-24, 12:52:05
Date	29 Jul 2024 13:06:34
Title	LC DATA FILE
Comment	315-2
Sampling Rate	150
Internal Name	007-0201_1
Detector Lag	0
Short File Name	007-0201_1
Sample Data	315-2
Instrument	UV detector (254 nm)Column

007-0201_1

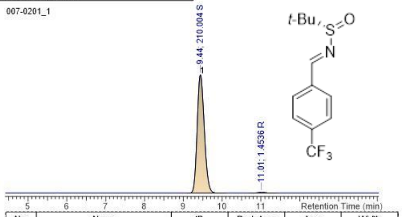

No.	Name	tR	Peak Area (Y units*ms)	Area (%)	Width
1	S	9.44	12600242.000	99.313	0.334
2	R	11.01	87217.688	0.687	0.380

Figure S60. HPLC Data for **(*S*)-13e**

LC DATA FILE

26 Aug 2024

File Name	\\storage.york.ac.uk\chemistry\services\Chromatography\CDCSPAC\Data\20240625-JDG558_HEX95_IC_291-RAC 2024-07-01 11-26-52\001-0101.D\dad1A.ch
Type	HPLC
Points Count	4501
Date Stamp	01-Jul-24, 11:27:55
Date	01 Jul 2024 11:57:58
Title	LC DATA FILE
Comment	291-rac-ADH
Sampling Rate	150
Detector Lag	0
Short File Name	dad1A.ch
Sample Data	291-rac-ADH
Instrument	UV detector (254 nm)Column

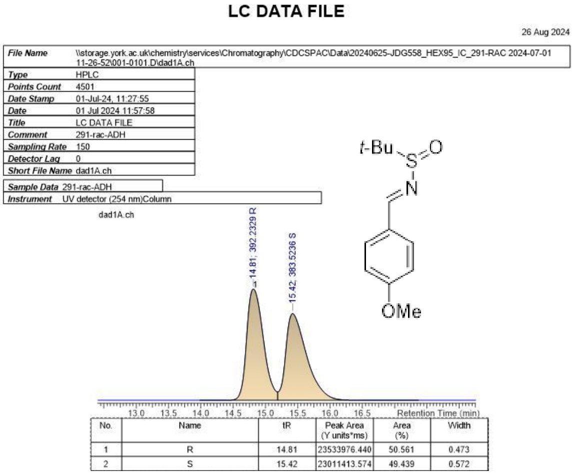

No.	Name	tR	Peak Area (Y units*ms)	Area (%)	Width
1	R	14.81	23533976.440	50.561	0.473
2	S	15.42	23011413.574	49.439	0.572

LC DATA FILE

26 Aug 2024

File Name	\\storage.york.ac.uk\chemistry\services\Chromatography\CDCSPAC\Data\20240701-JDG558_HEX95_ADH_291-1 2024-07-01 12-01-47\002-0101.D\dad1A.ch
Type	HPLC
Points Count	4501
Date Stamp	01-Jul-24, 12:02:50
Date	01 Jul 2024 12:32:54
Title	LC DATA FILE
Comment	291-1
Sampling Rate	150
Detector Lag	0
Short File Name	dad1A.ch
Sample Data	291-1
Instrument	UV detector (254 nm)Column

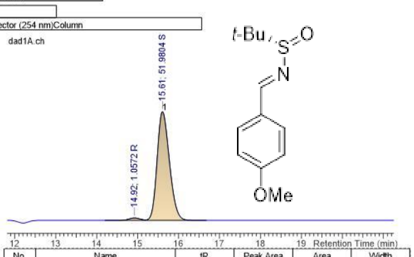

No.	Name	tR	Peak Area (Y units*ms)	Area (%)	Width
1	R	14.92	63431.361	1.993	0.510
2	S	15.61	3118824.234	98.007	0.548

Figure S61. HPLC Data for **(*S*)-13f**

LC DATA FILE

26 Aug 2024

File Name	C:\windows\system32\4-CN-SO_ENZY_1
Type	HPLC
Points Count	2886
Date Stamp	03-Jul-24, 15:56:15
Date	03 Jul 2024 16:15:34
Title	LC DATA FILE
Comment	294-rac
Sampling Rate	150
Internal Name	4-CN-SO_ENZY_1
Detector Lag	0
Short File Name	4-CN-SO_ENZY_1
Sample Data	294-rac
Instrument	UV detector (254 nm)Column

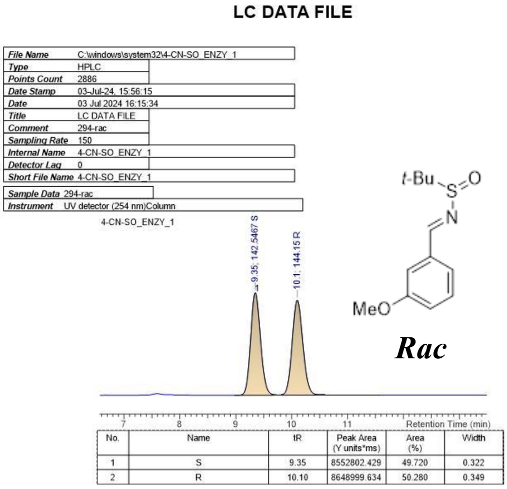

No.	Name	tR	Peak Area (Y units*ms)	Area (%)	Width
1	S	9.35	8552802.429	49.720	0.322
2	R	10.10	8648999.634	50.280	0.349

LC DATA FILE

26 Aug 2024

File Name	\\storage.york.ac.uk\chemistry\services\Chromatography\CDCSPAC\Data\20240703-JDG558_HEX95_ADH_294-1 2024-07-03 16-17-53\4-CN-SO_ENZY.D\dad1A.ch
Type	HPLC
Points Count	4501
Date Stamp	03-Jul-24, 16:18:56
Date	03 Jul 2024 16:49:00
Title	LC DATA FILE
Comment	294-1
Sampling Rate	150
Detector Lag	0
Short File Name	dad1A.ch
Sample Data	294-1
Instrument	UV detector (254 nm)Column

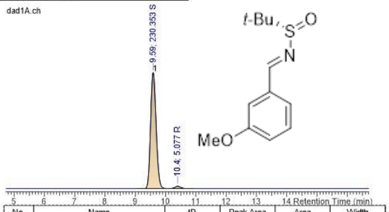

No.	Name	tR	Peak Area (Y units*ms)	Area (%)	Width
1	S	9.59	13821177.063	97.844	0.332
2	R	10.40	304617.119	2.156	0.357

Figure S62. HPLC Data for**(*S*)-13g**

LC DATA FILE

26 Aug 2024

File Name	\\storage.york.ac.uk\chemistry\services\Chromatography\CDCSPAC\Data\20240729_JDG558_HEX95_IPA5_IBN5-318-2 2024-07-29 14-44-11\005-0101.D\dad1A.ch
Type	HPLC
Points Count	1853
Date Stamp	29-Jul-24, 14:45:15
Date	29 Jul 2024 14:57:40
Title	LC DATA FILE
Comment	318-2
Sampling Rate	150
Detector Lag	0
Short File Name	dad1A.ch
Sample Data	318-2
Instrument	UV detector (254 nm)Column

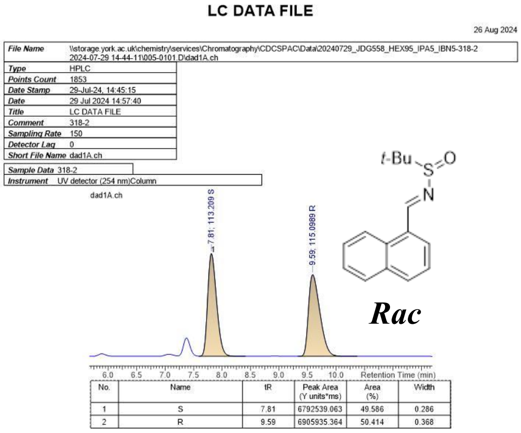

No.	Name	tR	Peak Area (Y units*ms)	Area (%)	Width
1	S	7.81	6792539.063	49.586	0.286
2	R	9.59	6905935.364	50.414	0.368

LC DATA FILE

26 Aug 2024

File Name	\\storage.york.ac.uk\chemistry\services\Chromatography\CDCSPAC\Data\20240729_JDG558_HEX95_IPA5_IBN5-318-1 2024-07-29 15-02-36\008-0101.D\dad1A.ch
Type	HPLC
Points Count	1980
Date Stamp	29-Jul-24, 15:03:41
Date	29 Jul 2024 15:16:56
Title	LC DATA FILE
Comment	318-1
Sampling Rate	150
Detector Lag	0
Short File Name	dad1A.ch
Sample Data	318-1
Instrument	UV detector (254 nm)Column

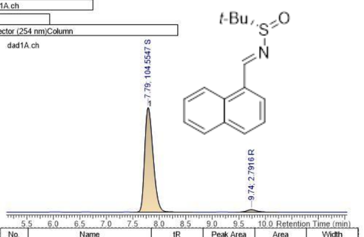

No.	Name	tR	Peak Area (Y units*ms)	Area (%)	Width
1	S	7.79	6273284.454	97.399	0.292
2	R	9.74	167494.941	2.601	0.325

Figure S63. HPLC Data for **(*S*)-13h**

LC DATA FILE

26 Aug 2024

File Name	C:\windows\system32\002-0201_1
Type	HPLC
Points Count	7911
Date Stamp	31-Jul-24, 12:50:15
Date	31 Jul 2024 13:43:02
Title	LC DATA FILE
Comment	321-2R
Sampling Rate	150
Internal Name	002-0201_1
Detector Lag	0
Short File Name	002-0201_1
Sample Data	321-2R
Instrument	UV detector (254 nm)Column

002-0201_1

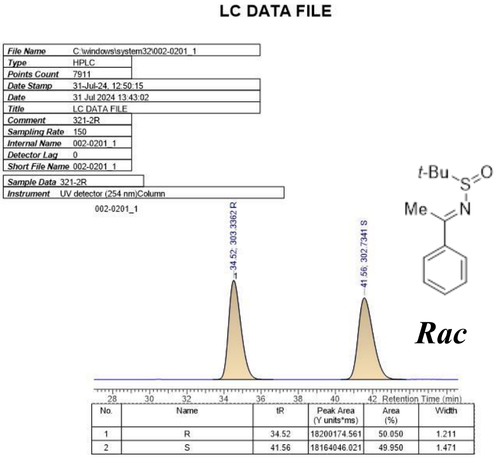

No.	Name	tR	Peak Area (Y units*ms)	Area (%)	Width
1	R	34.52	18200174.561	50.050	1.211
2	S	41.56	18164046.021	49.950	1.471

LC DATA FILE

26 Aug 2024

File Name	C:\windows\system32\004-0201_1
Type	HPLC
Points Count	6935
Date Stamp	31-Jul-24, 14:36:12
Date	31 Jul 2024 15:22:28
Title	LC DATA FILE
Comment	321-2
Sampling Rate	150
Internal Name	004-0201_1
Detector Lag	0
Short File Name	004-0201_1
Sample Data	321-2
Instrument	UV detector (254 nm)Column

004-0201_1

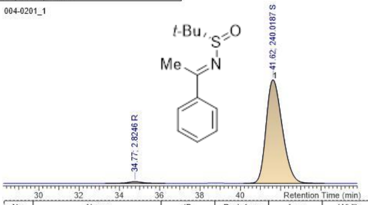

No.	Name	tR	Peak Area (Y units*ms)	Area (%)	Width
1	R	34.77	169475.684	1.163	1.174
2	S	41.62	14401123.352	98.837	1.467

Figure S64. HPLC Data for **(*S*)-13i**

LC DATA FILE

26 Aug 2024

File Name	C:\windows\system32\001-0101_1
Type	HPLC
Points Count	9001
Date Stamp	31-Jul-24, 11:49:02
Date	31 Jul 2024 12:49:04
Title	LC DATA FILE
Comment	321-1R
Sampling Rate	150
Internal Name	001-0101_1
Detector Lag	0
Short File Name	001-0101_1
Sample Data	321-1R
Instrument	UV detector (254 nm)Column

001-0101_1

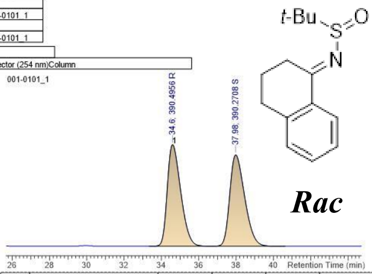

No.	Name	tR	Peak Area (Y units*ms)	Area (%)	Width
1	R	34.60	23429736.328	50.014	1.314
2	S	37.98	23416245.117	49.986	1.461

LC DATA FILE

26 Aug 2024

File Name	C:\windows\system32\003-0101_1
Type	HPLC
Points Count	7501
Date Stamp	31-Jul-24, 13:44:55
Date	31 Jul 2024 14:34:58
Title	LC DATA FILE
Comment	321-1
Sampling Rate	150
Internal Name	003-0101_1
Detector Lag	0
Short File Name	003-0101_1
Sample Data	321-1
Instrument	UV detector (254 nm)Column

003-0101_1

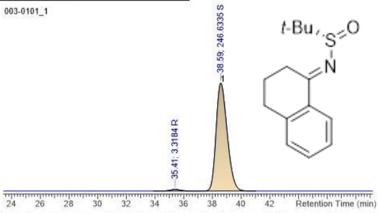

No.	Name	tR	Peak Area (Y units*ms)	Area (%)	Width
1	R	35.41	199104.500	1.328	1.244
2	S	38.59	14798008.000	98.672	1.413

Figure S65. HPLC Data for **(*S*)-13k**

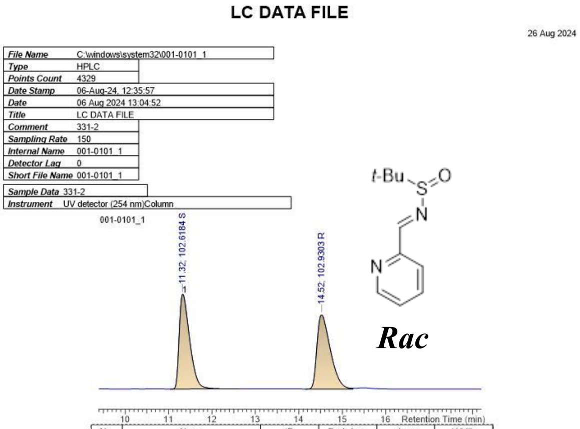

No.	Name	tR	Peak Area (Y units*ms)	Area (%)	Width
1	S	11.32	6157102.203	49.924	0.433
2	R	14.52	6175815.582	50.076	0.561

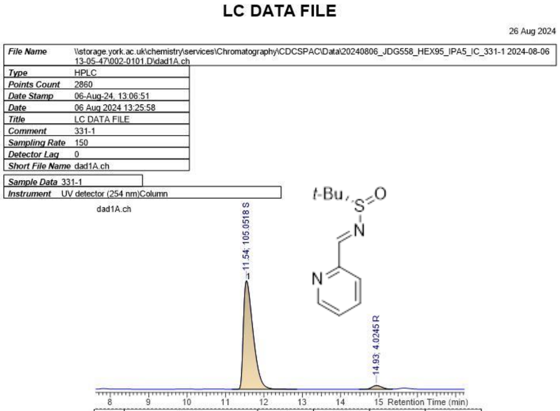

No.	Name	tR	Peak Area (Y units*ms)	Area (%)	Width
1	S	11.54	6303107.500	96.310	0.456
2	R	14.93	241470.297	3.690	0.492

Figure S66. HPLC Data for **(*S*)-13l**

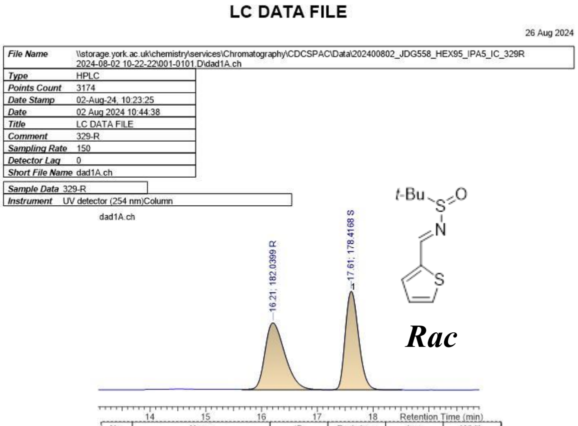

No.	Name	tR	Peak Area (Y units*ms)	Area (%)	Width
1	S	17.61	10705008.545	49.497	0.440
2	R	16.21	10922393.188	50.503	0.649

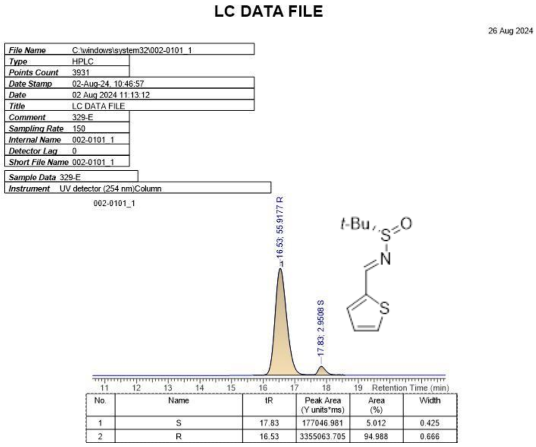

No.	Name	tR	Peak Area (Y units*ms)	Area (%)	Width
1	S	17.83	177046.981	5.012	0.425
2	R	16.53	3355063.705	94.988	0.666

Figure S67. HPLC Data for **(*S*)-13m**

LC DATA FILE

26 Aug 2024

File Name	\\storage.york.ac.uk\chemistry\services\Chromatography\CDCSPAC\Data\20240717_JDG558_300-303 RAC_IC 2024-07-17 12-29-00\ENZYMATIC SAMPLE.D\dad1A.ch
Type	HPLC
Points Count	3193
Date Stamp	17-Jul-24, 13:01:44
Date	17 Jul 2024 13:23:06
Title	LC DATA FILE
Comment	LJC-303-RAC
Sampling Rate	150
Detector Lag	0
Short File Name	dad1A.ch

Sample Data	LJC-303-RAC
Instrument	UV detector (254 nm)Column

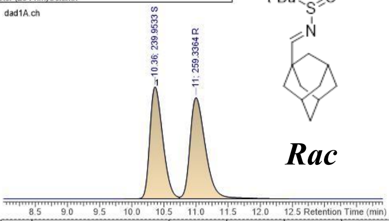

No.	Name	tR	Peak Area (Y units*ms)	Area (%)	Width
1	S	10.36	14397197.571	48.059	0.368
2	R	11.00	15560181.885	51.941	0.424

LC DATA FILE

26 Aug 2024

File Name	\\storage.york.ac.uk\chemistry\services\Chromatography\CDCSPAC\Data\20240717_JDG558_300-303 ENY_IC 2024-07-17 13-25-12\004-0101.D\dad1A.ch
Type	HPLC
Points Count	4501
Date Stamp	17-Jul-24, 13:26:17
Date	17 Jul 2024 13:56:20
Title	LC DATA FILE
Comment	LJC-303-ENY
Sampling Rate	150
Detector Lag	0
Short File Name	dad1A.ch

Sample Data	LJC-303-ENY
Instrument	UV detector (254 nm)Column

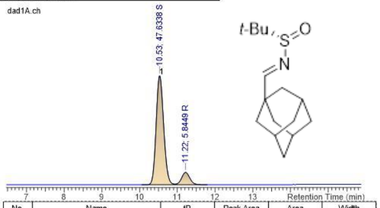

No.	Name	tR	Peak Area (Y units*ms)	Area (%)	Width
1	S	10.53	2858028.259	89.071	0.361
2	R	11.22	350693.293	10.929	0.391

Figure S68. HPLC Data for ***(S)*-13n**

LC DATA FILE

26 Aug 2024

File Name	C:\windows\system32\20240807_SAH_KS-2-074_1
Type	HPLC
Points Count	2671
Date Stamp	12-Aug-24, 13:00:37
Date	12 Aug 2024 13:18:28
Title	LC DATA FILE
Comment	20240812_JDG_345R_IC55955
Sampling Rate	150
Internal Name	20240807_SAH_KS-2-074_1
Detector Lag	0
Short File Name	20240807_SAH_KS-2-074_1

Sample Data	20240812_JDG_345R_IC55955
Instrument	UV detector (254 nm)Column

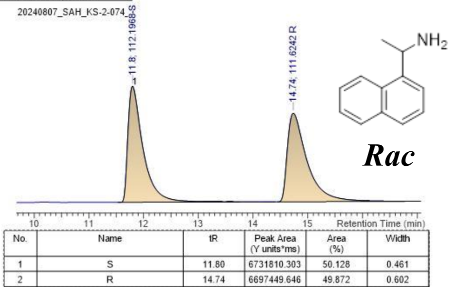

No.	Name	tR	Peak Area (Y units*ms)	Area (%)	Width
1	S	11.80	6731810.303	50.128	0.461
2	R	14.74	6697449.646	49.872	0.602

LC DATA FILE

26 Aug 2024

File Name	\\storage.york.ac.uk\chemistry\services\Chromatography\CDCSPAC\Data\20240812_JDG558_H95_IPA5_IC_345E 2024-08-12 13-19-10\20240807_SAH_KS-2-074.D\dad1A.ch
Type	HPLC
Points Count	3898
Date Stamp	12-Aug-24, 13:20:13
Date	12 Aug 2024 13:46:16
Title	LC DATA FILE
Comment	20240812_JDG_345E_IC55955
Sampling Rate	150
Detector Lag	0
Short File Name	dad1A.ch

Sample Data	20240812_JDG_345E_IC55955
Instrument	UV detector (254 nm)Column

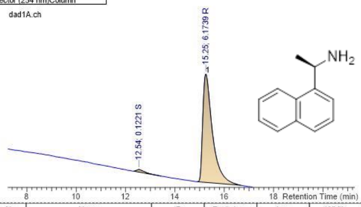

No.	Name	tR	Peak Area (Y units*ms)	Area (%)	Width
1	S	12.54	7327.459	1.940	0.598
2	R	15.25	370434.969	98.060	0.659

Figure S69. HPLC Data for **14**

LC DATA FILE

18 Mar 2025

File Name	\\storage.york.ac.uk\chemistry\services\Chromatography\CDCSPAC\Data\20240814_JDG558_H95_IPA5_IBN5346R 2024-08-14 10-22-06\20240807_SAH_KS-2-074.D\dad1E.ch
Type	HPLC
Points Count	3002
Date Stamp	14-Aug-24, 10:23:10
Date	14 Aug 2024 09:43:14
Title	LC DATA FILE
Comment	20240814_JDG_346R_IBN555955
Sampling Rate	150
Detector Lag	0
Short File Name	dad1E.ch

Sample Data	20240814_JDG_346R_IBN555955
Instrument	UV detector (280 nm)Column

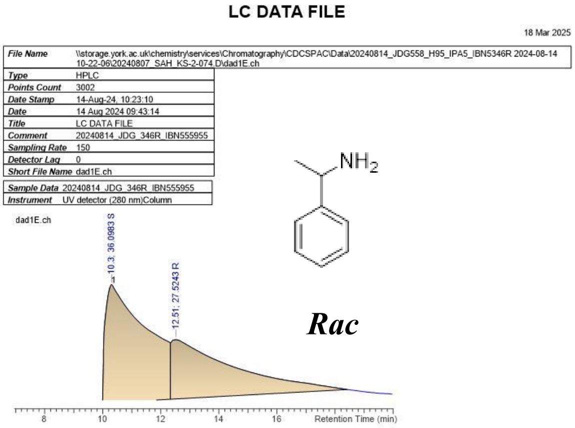

No.	Name	tR	Peak Area (Y units*ms)	Area (%)	Width
1	S	10.30	2165899.200	56.738	3.656
2	R	12.51	1651456.490	43.262	6.552

LC DATA FILE

18 Mar 2025

File Name	\\storage.york.ac.uk\chemistry\services\Chromatography\CDCSPAC\Data\20240814_JDG558_H95_IPA5_IBN5346E 2024-08-14 10-45-09\20240807_SAH_KS-2-074.D\dad1E.ch
Type	HPLC
Points Count	3584
Date Stamp	14-Aug-24, 10:46:13
Date	14 Aug 2024 10:10:10
Title	LC DATA FILE
Comment	20240814_JDG_346E_IBN555955
Sampling Rate	150
Detector Lag	0
Short File Name	dad1E.ch

Sample Data	20240814_JDG_346E_IBN555955
Instrument	UV detector (280 nm)Column

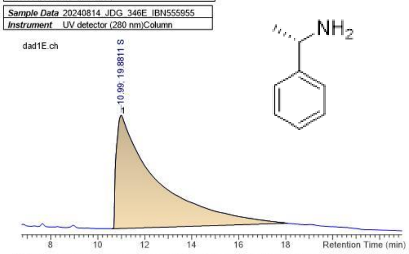

No.	Name	tR	Peak Area (Y units*ms)	Area (%)	Width
1	S	10.99	1192867.000	100.000	2.411

Figure S70. HPLC Data for **18**

6) Molecular Docking

Automated docking was performed using AUTODOCK VINA 1.1.2.[33] Coordinates for the ligands (*R*)-**4e** and **6** were prepared using ELBOW[34] in PHENIX.[35] The appropriate pdbqt files for the models of artUPO and r*Aae*UPO-PaDa-I-H and the ligands were prepared in AUTODOCK Tools from structures with PDB codes 7ZNM[32] and 6EL0[36] respectively and into each of which had been modelled heme with Compound I from P450 structure 1DZ9.[37] The active site of artUPO was contained in a grid size of 34 Å × 34 Å × 34 Å (corresponding to x, y, z) with 1 Å spacing, centred around the catalytic centre at positions -2.55 Å × 5.28 Å × -27.21 Å (corresponding to x, y, z). The active site of r*Aae*UPO-PaDa-I was contained in grid sizes of 34 Å × 34 Å × 34 Å (corresponding to x, y, z) with 1 Å spacing, centred around the catalytic centre at positions -2.26 Å × 4.29 Å × -25.20 Å (corresponding to x, y, z). These values were generated using AutoGrid in the AUTODOCK Tools interface. The dockings were performed by VINA, therefore the posed dockings were below 2 Å r.m.s.d. The results generated by VINA were visualised in AUTODOCK Tools 1.5.6 where the ligand conformations were assessed based upon lowest VINA energy. Figure SXA shows the active site of artUPO with substrate (*R*)-**4e**; Figure SXB shows a superimposition of the active sites of artUPO and r*Aae*UPO-PaDa-I-H with substrate **6**.

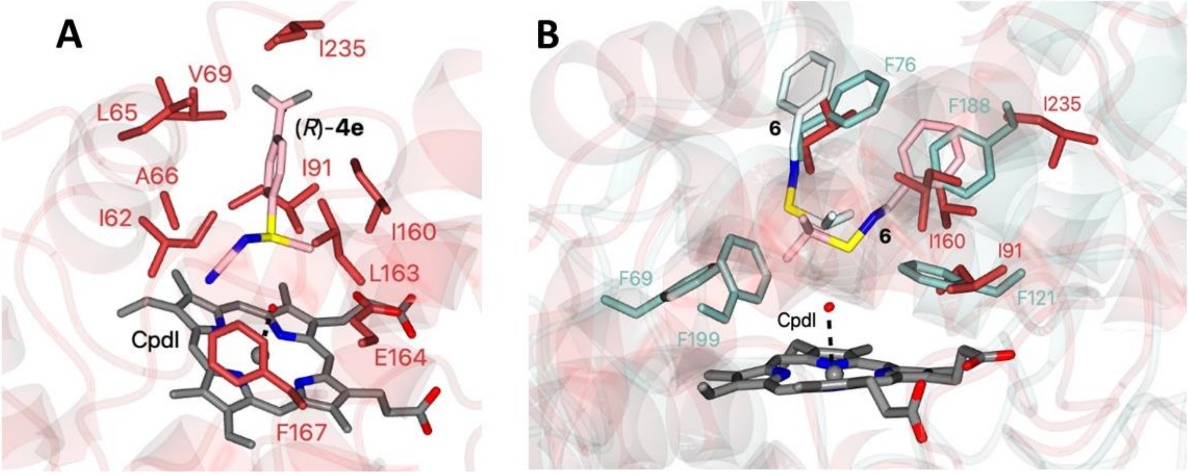

Figure S71. A. Active site of artUPO (from 7ZNM; carbon atoms in red) with (*R*)-**4e** modelled using Autodock VINA.[33] **B**. Superimposition of active sites of artUPO (from 7ZNM; carbon atoms in red) and *Aae*UPO-PaDa-I (from 6EL0; blue) with **6** (pink for artUPO and light blue for *Aae*UPO-PaDa-I) modelled using Autodock VINA.[33]

7) ^{1}H, ^{13}C and ^{19}F NMR spectra

Figure S72: NMR data for **4a**

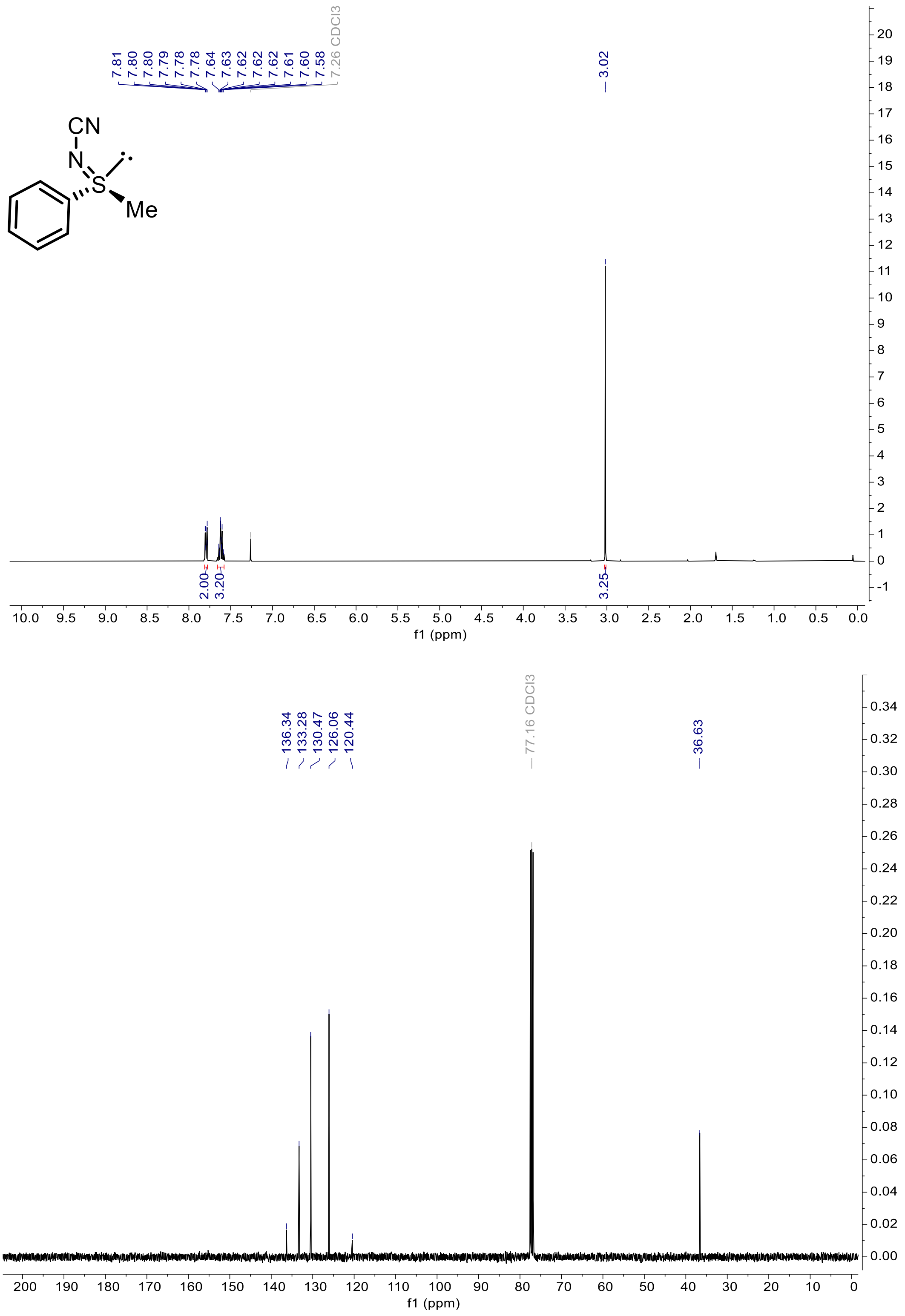

Figure S73: NMR data for **4b**

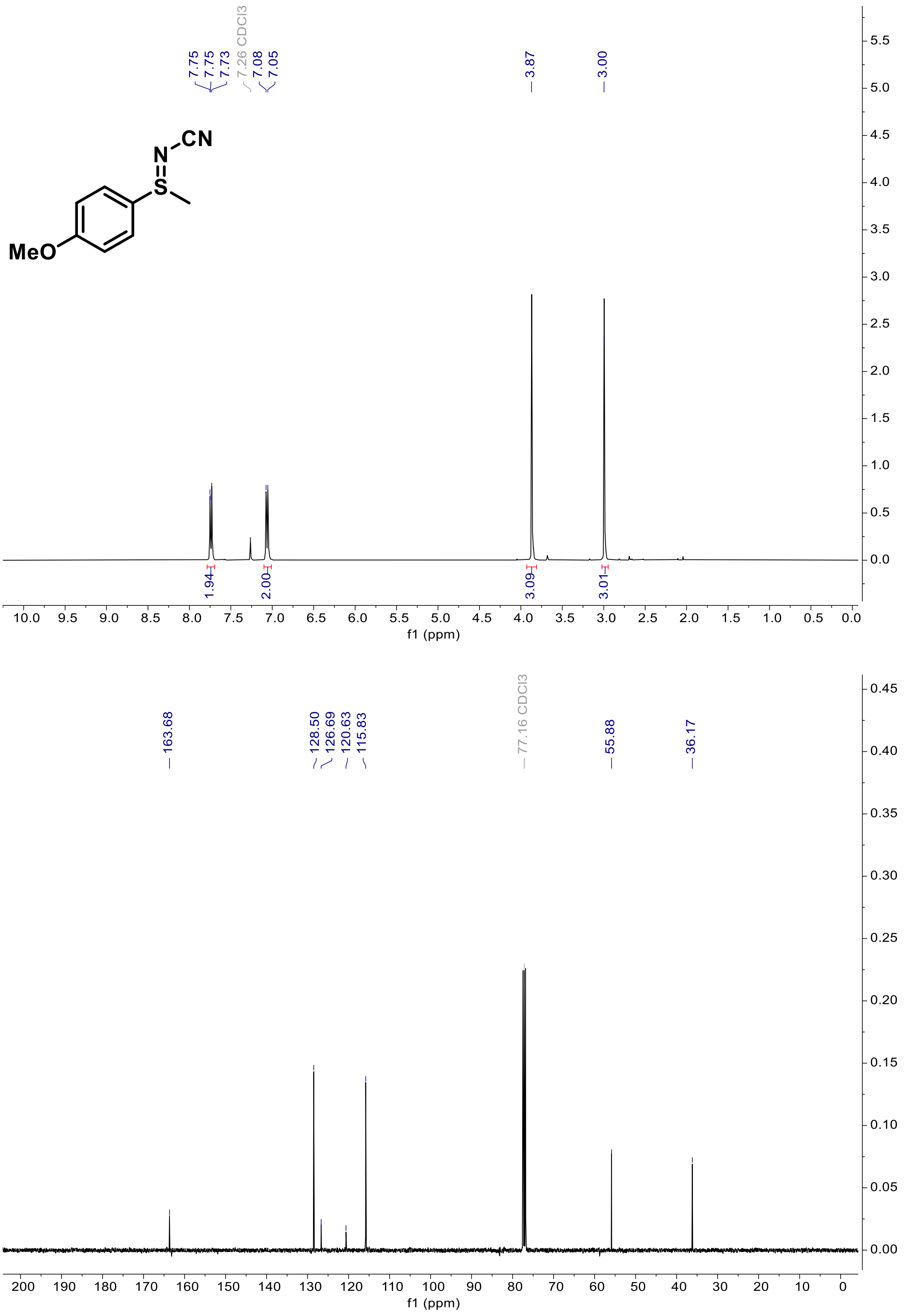

Figure S74: NMR data for **4c**:

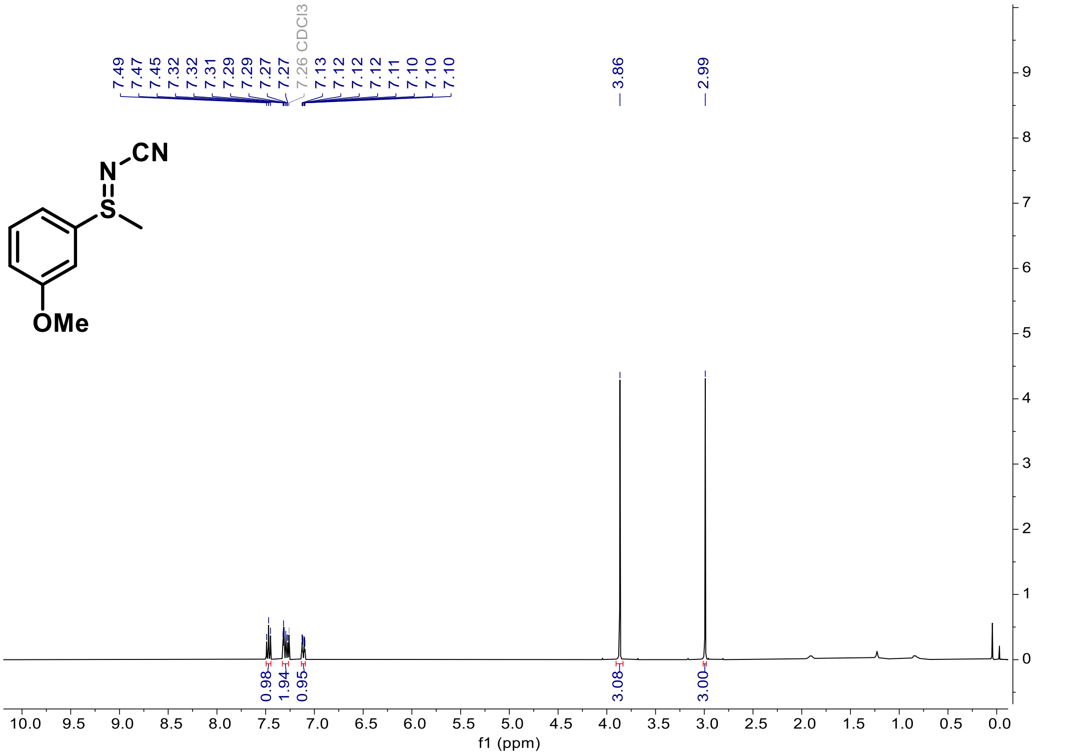

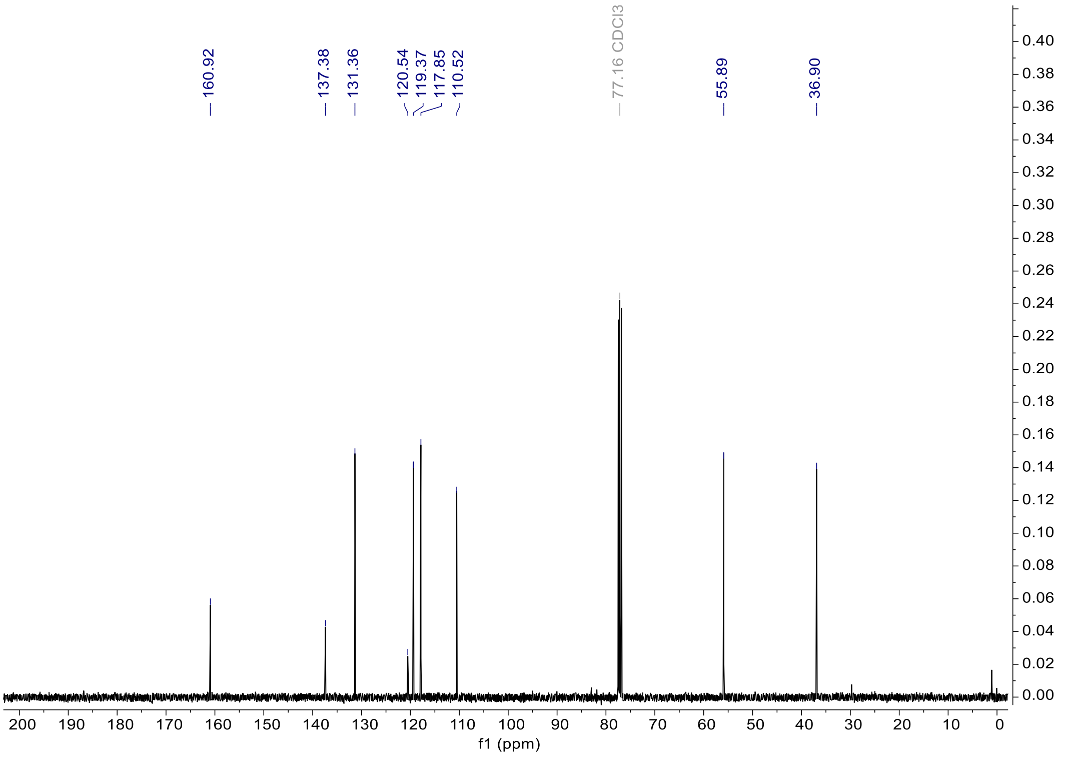

Figure S75: NMR data for **4d:**

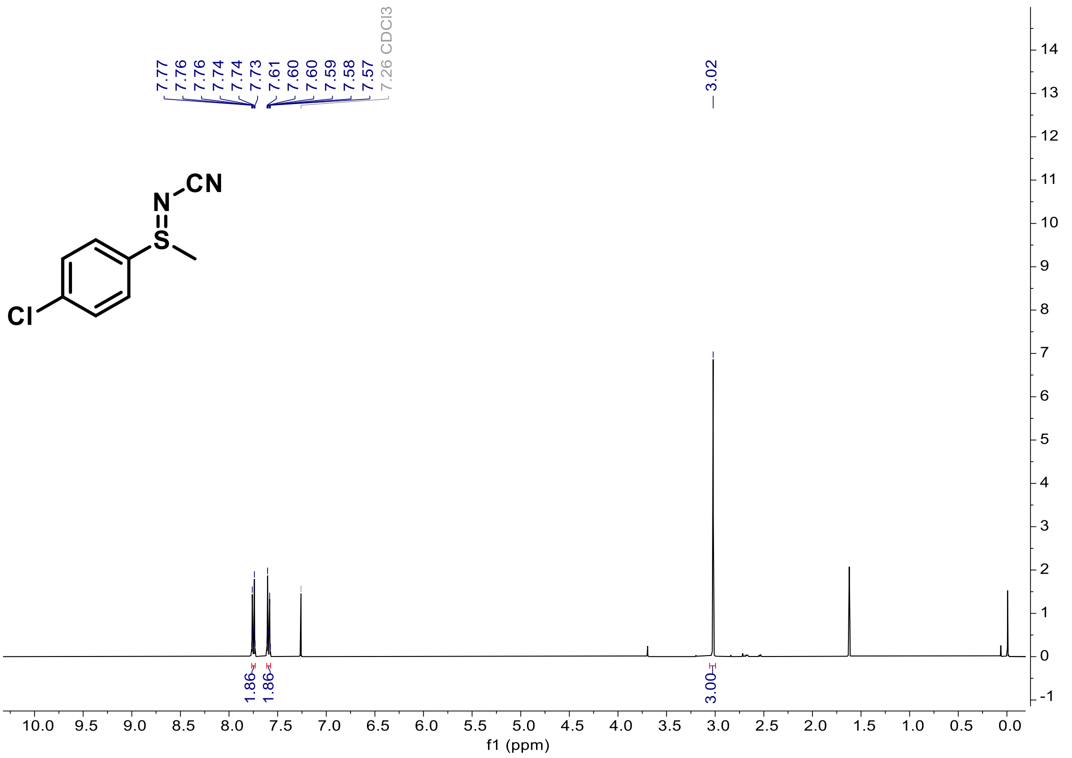

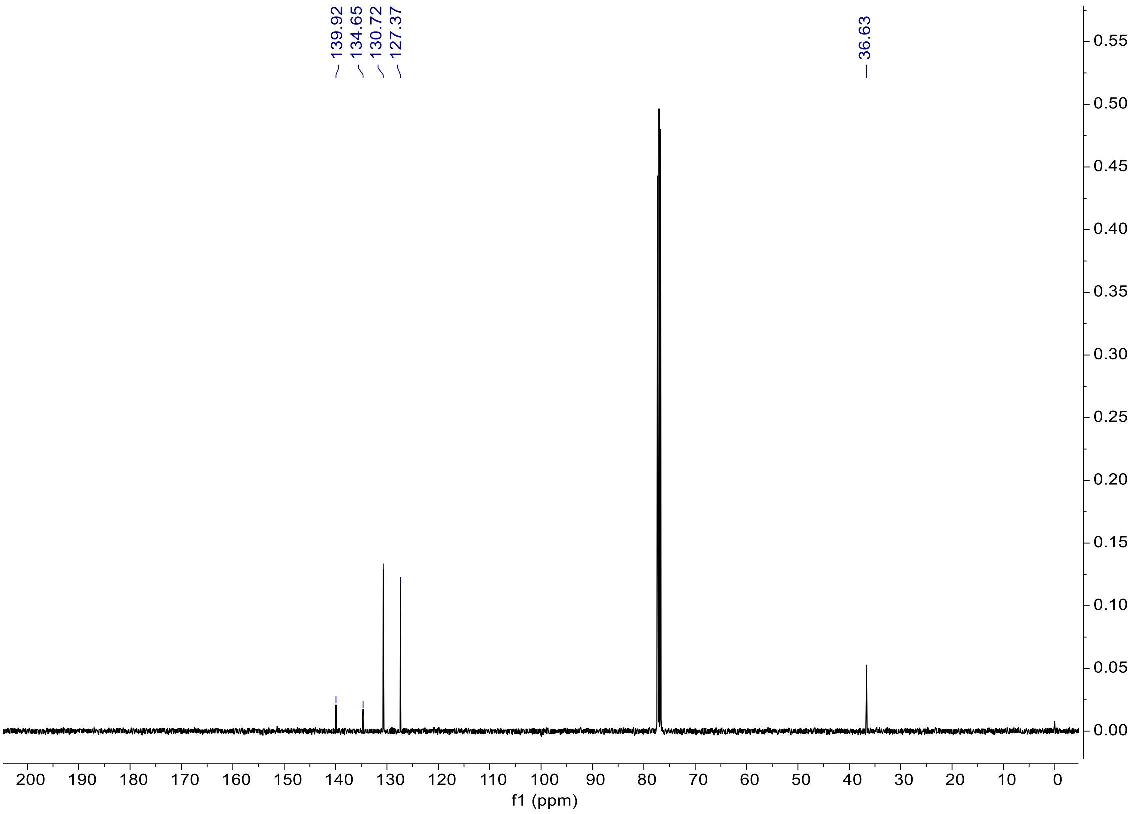

Figure S76: NMR data for **4e**

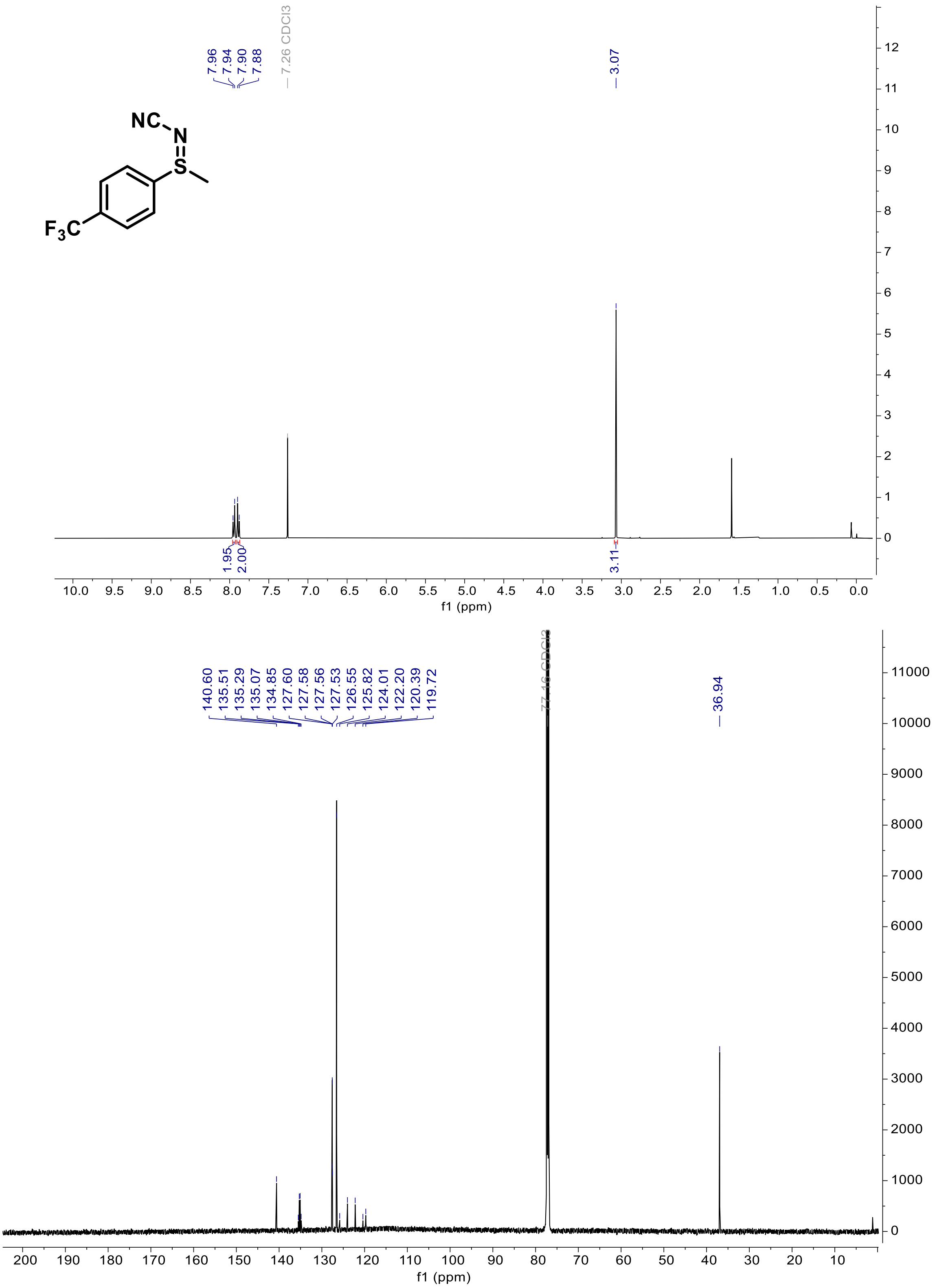

Figure S77: NMR data for **4f:**

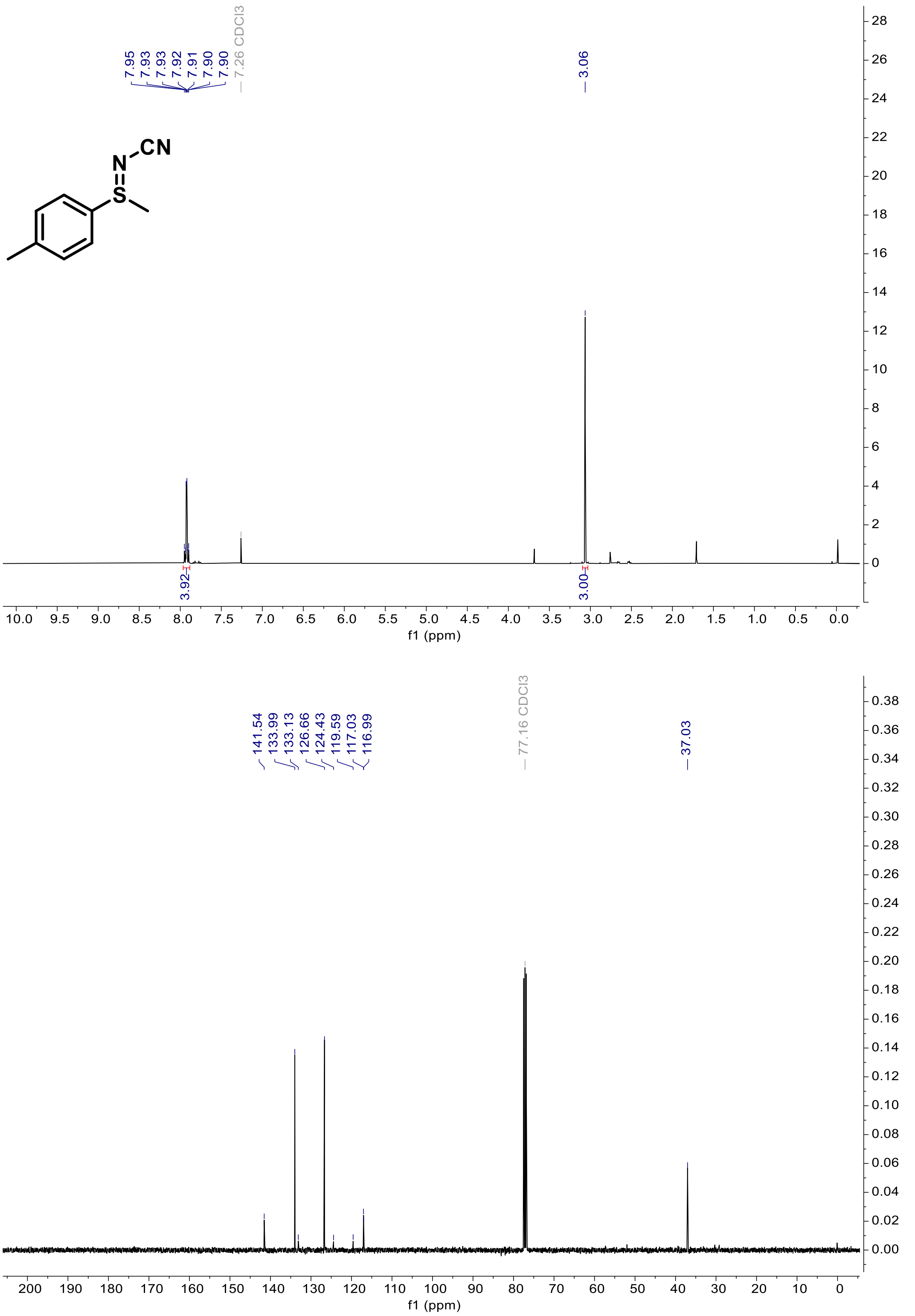

Figure S78: NMR data for **4g**

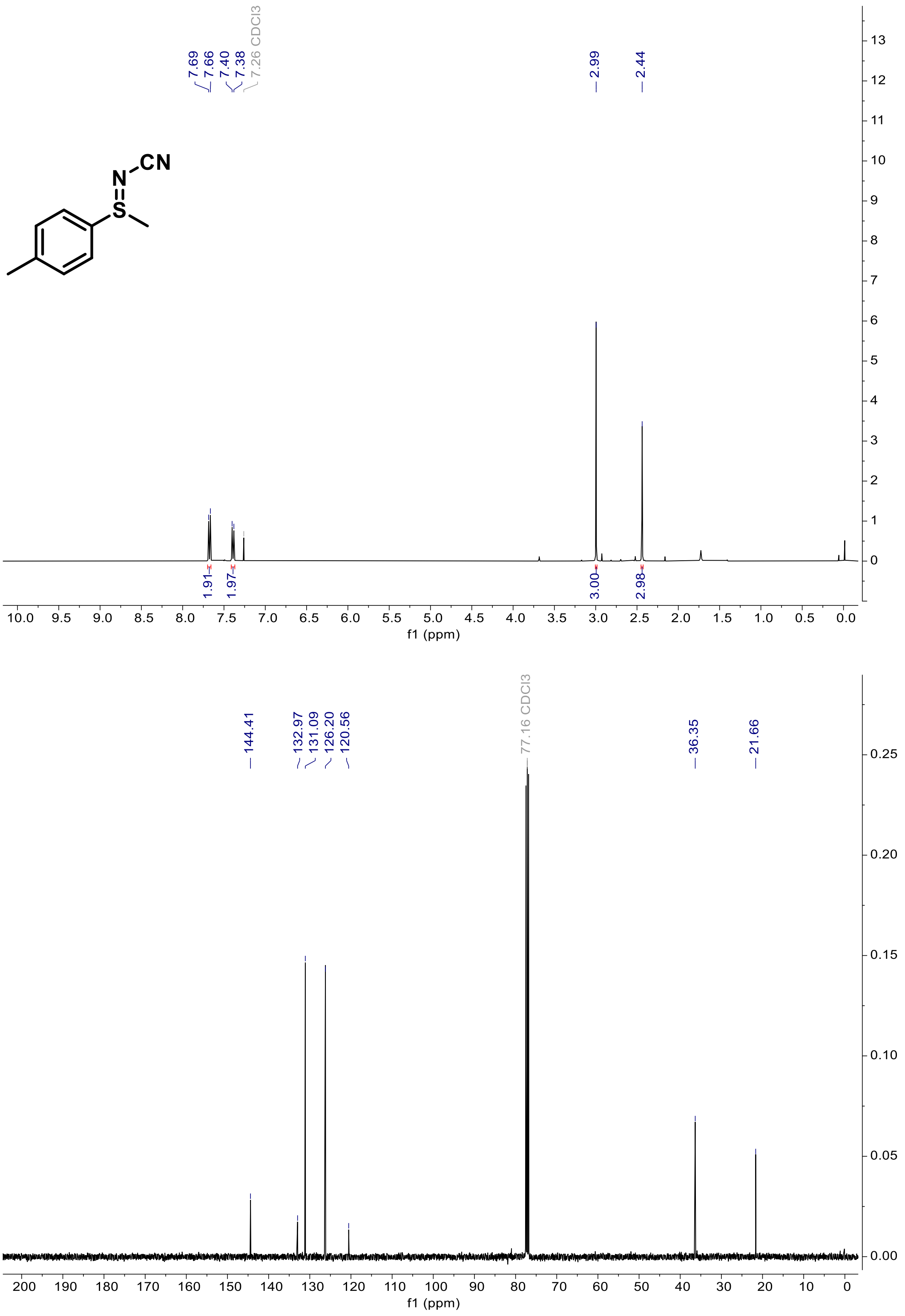

Figure S79: NMR data for **4h**

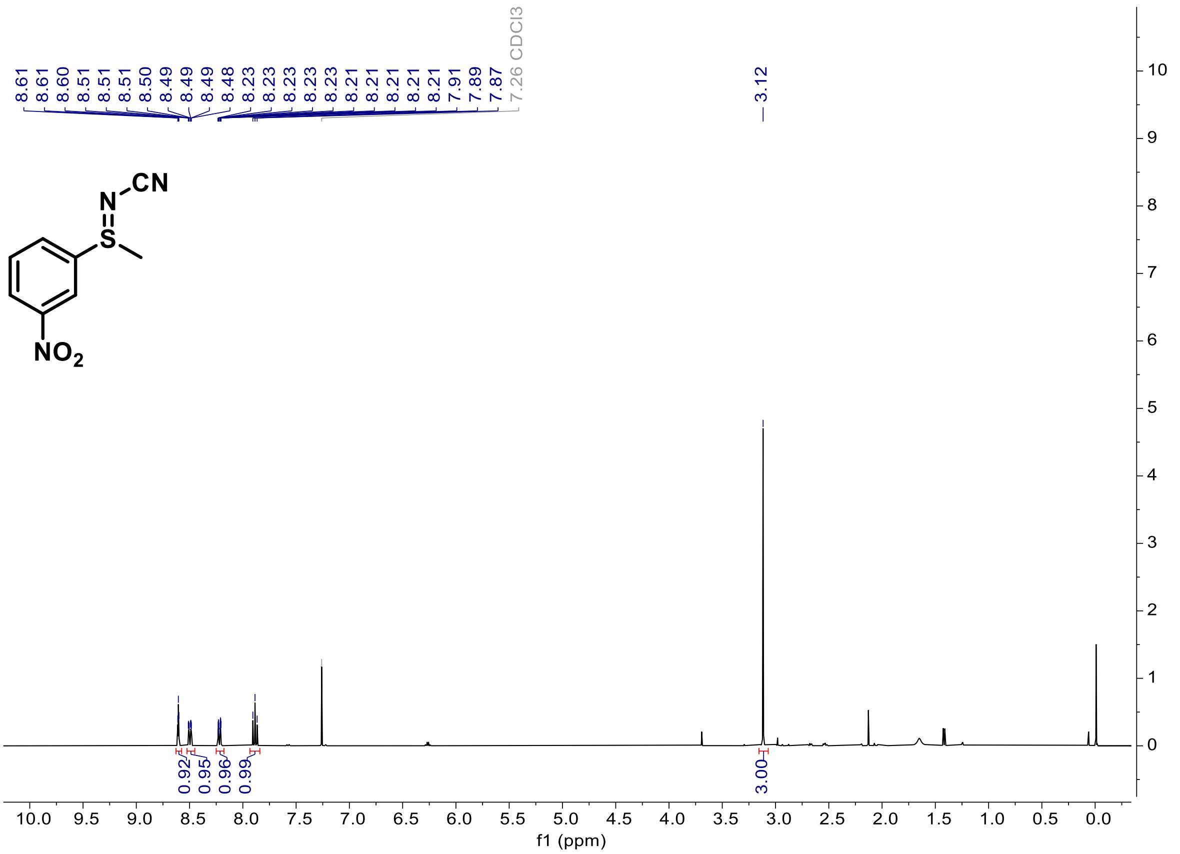

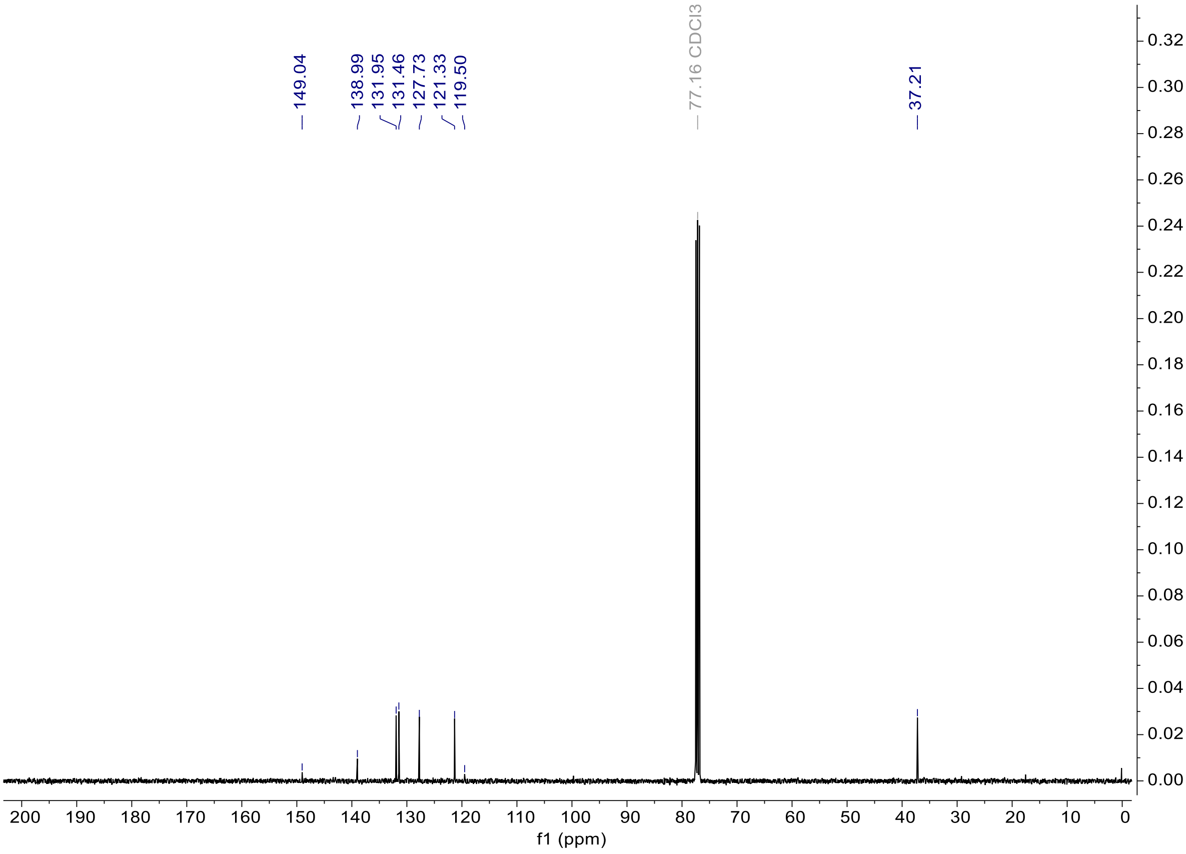

Figure S80: NMR data for **4i**

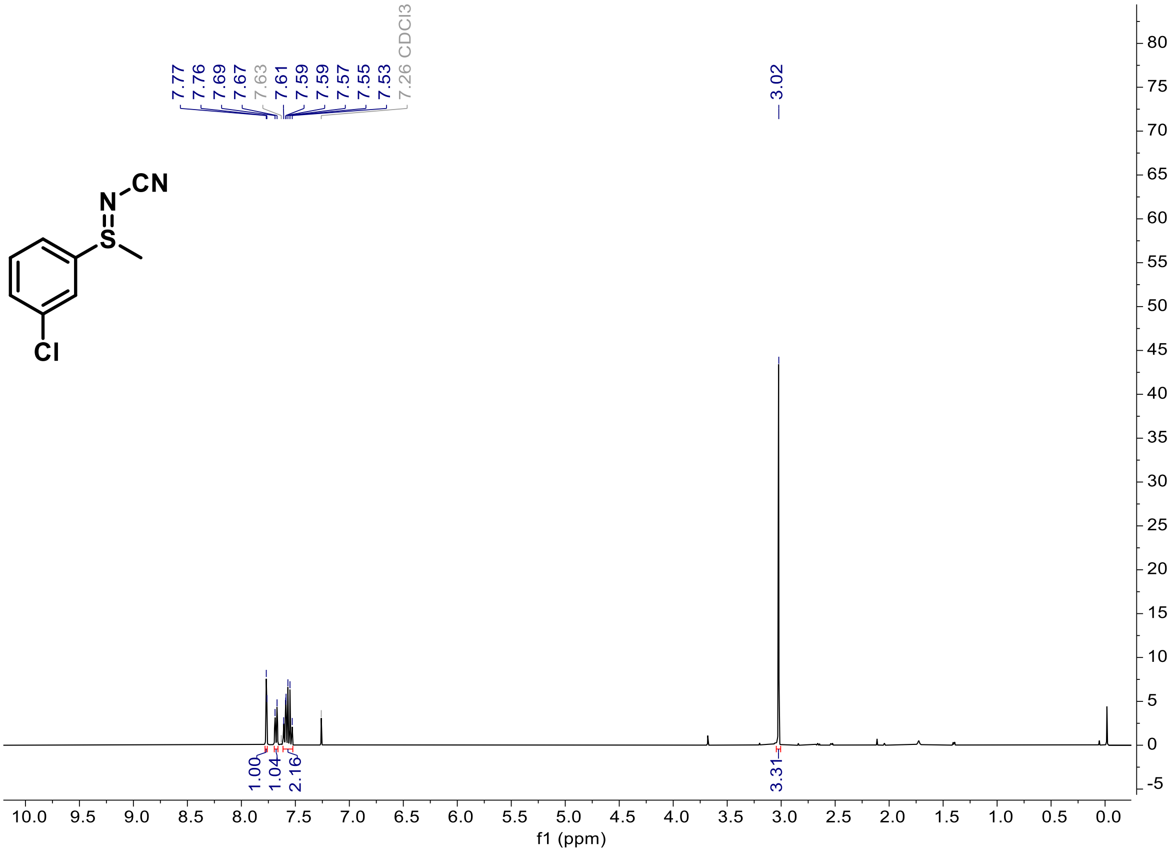

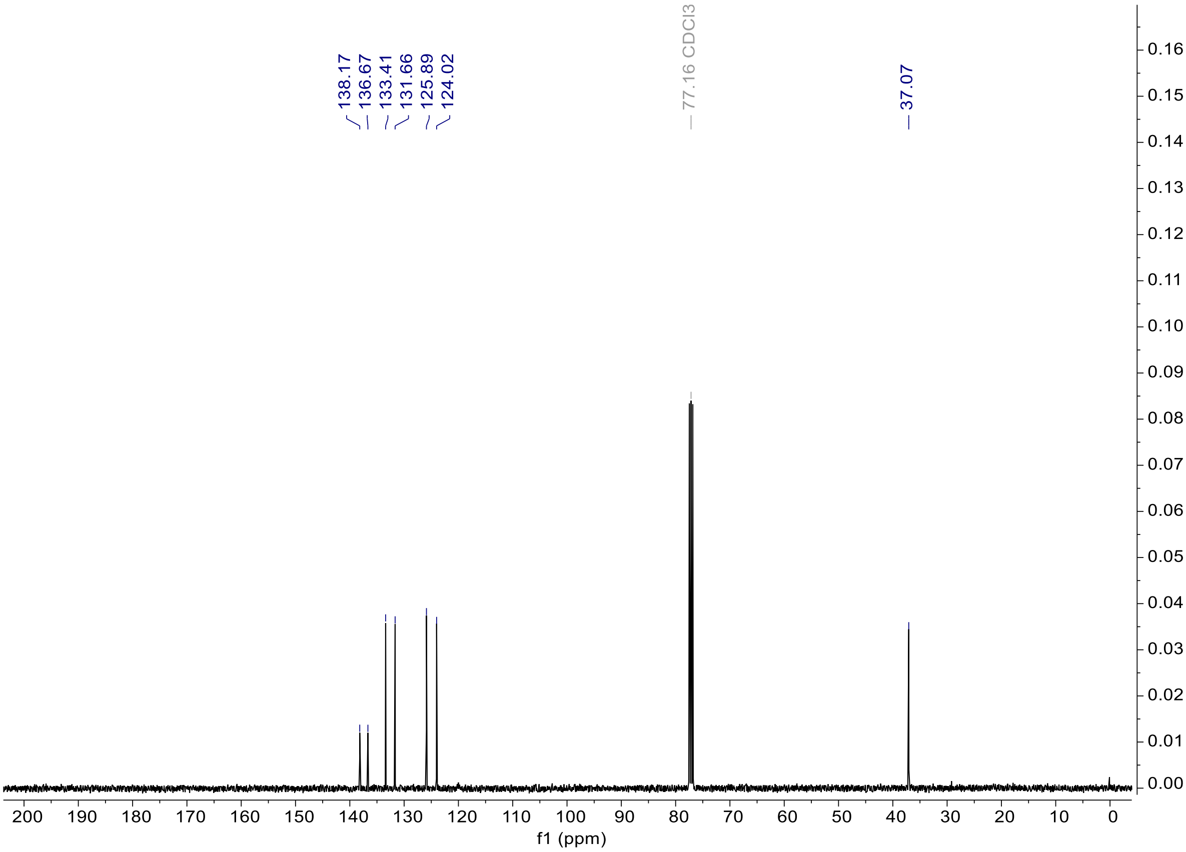

Figure S81: NMR data for **4j:**

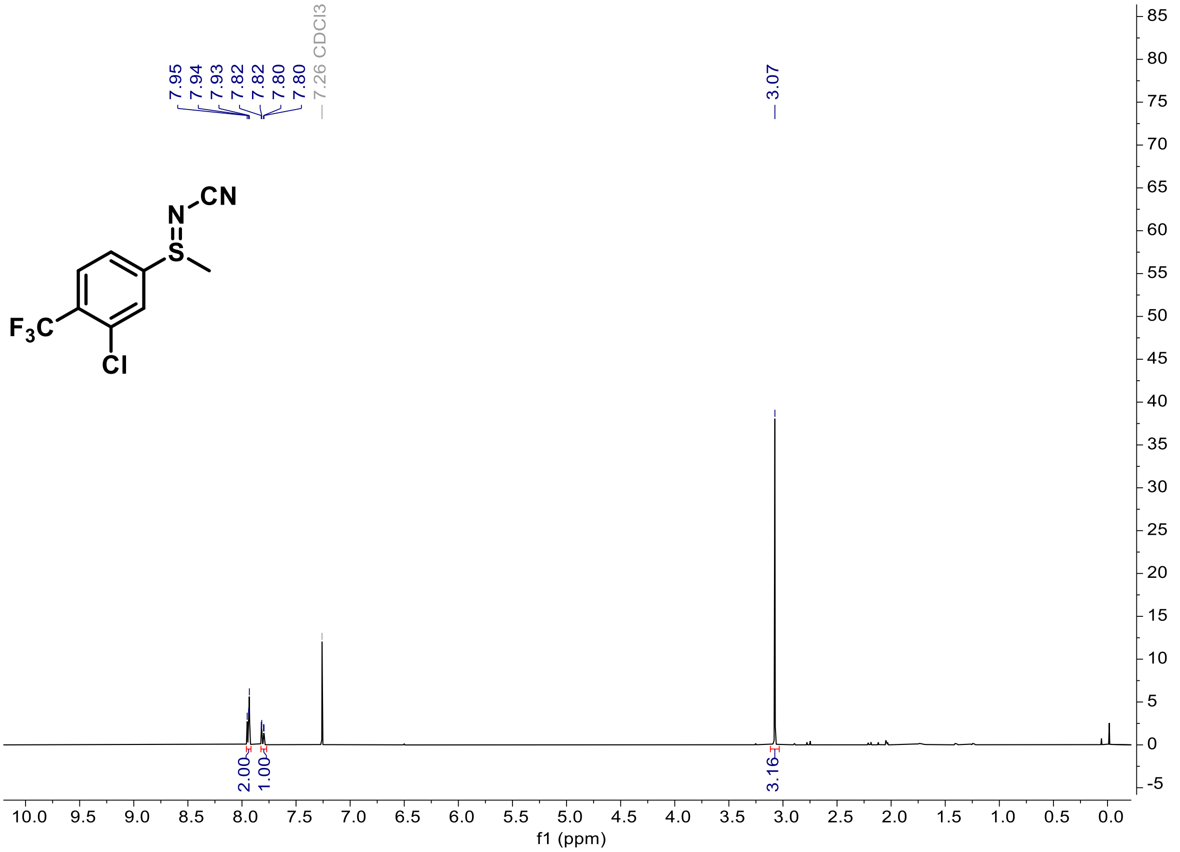

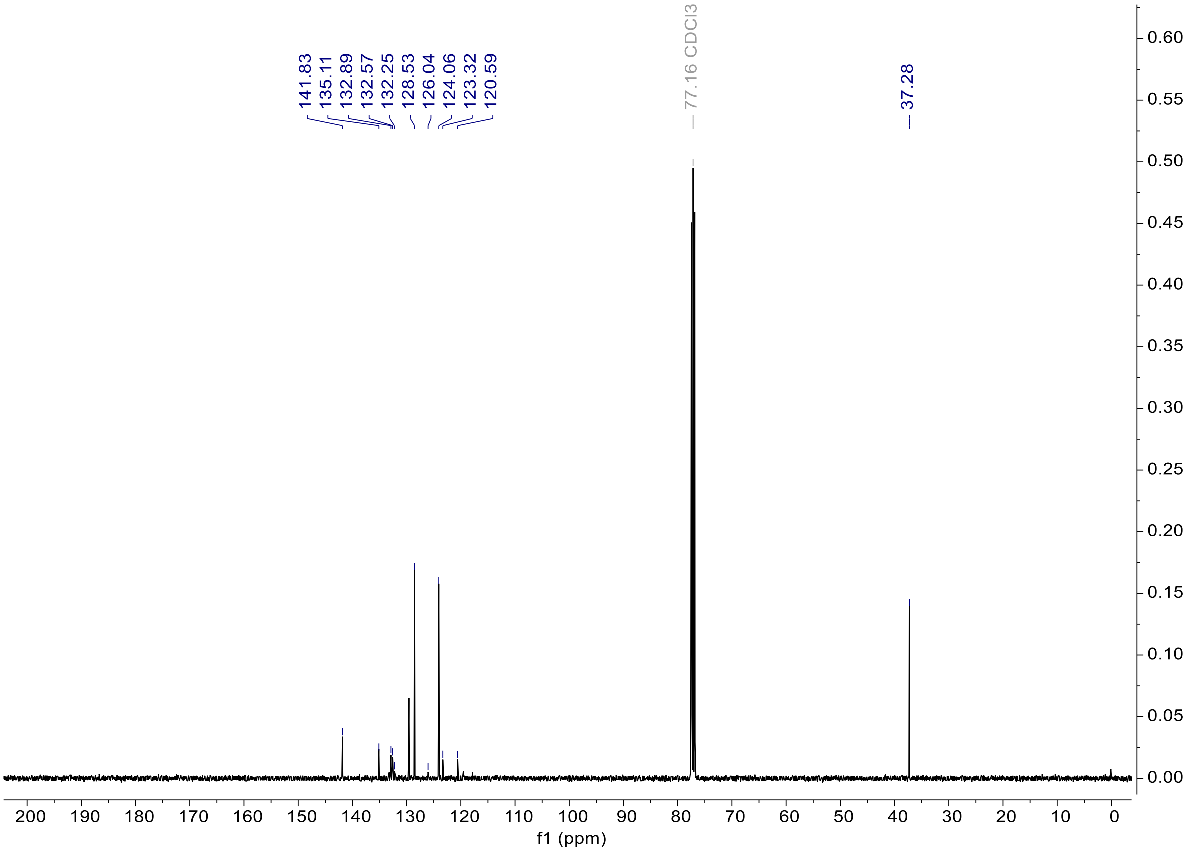

Figure S82: NMR data for **4k**

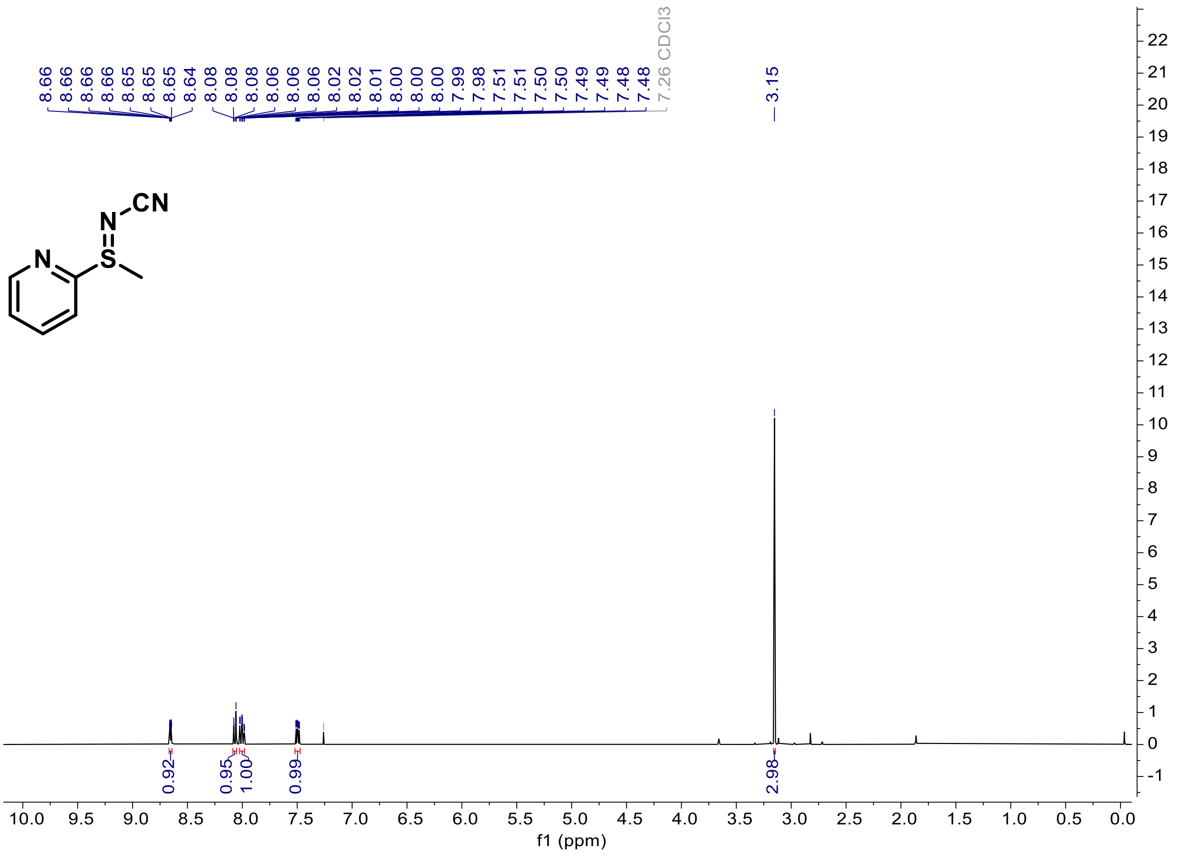

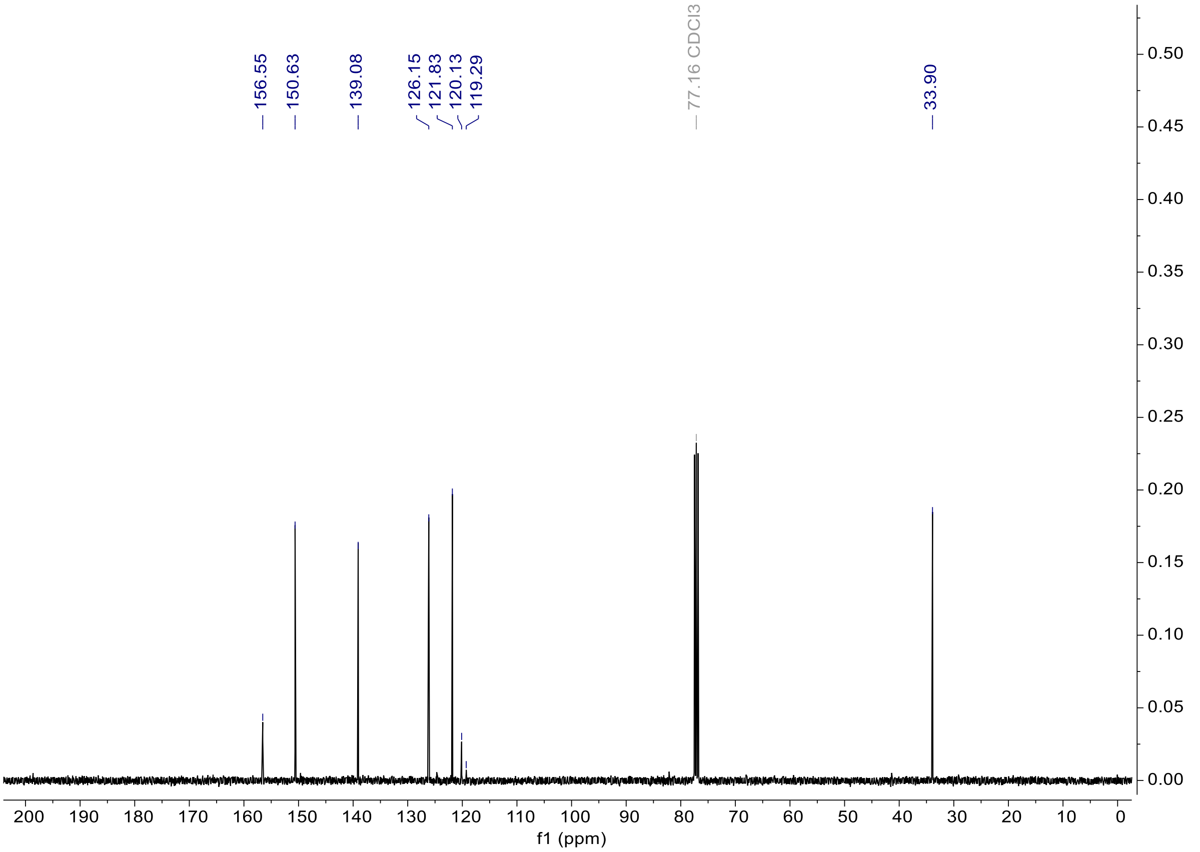

Figure S83: NMR data for **4l**

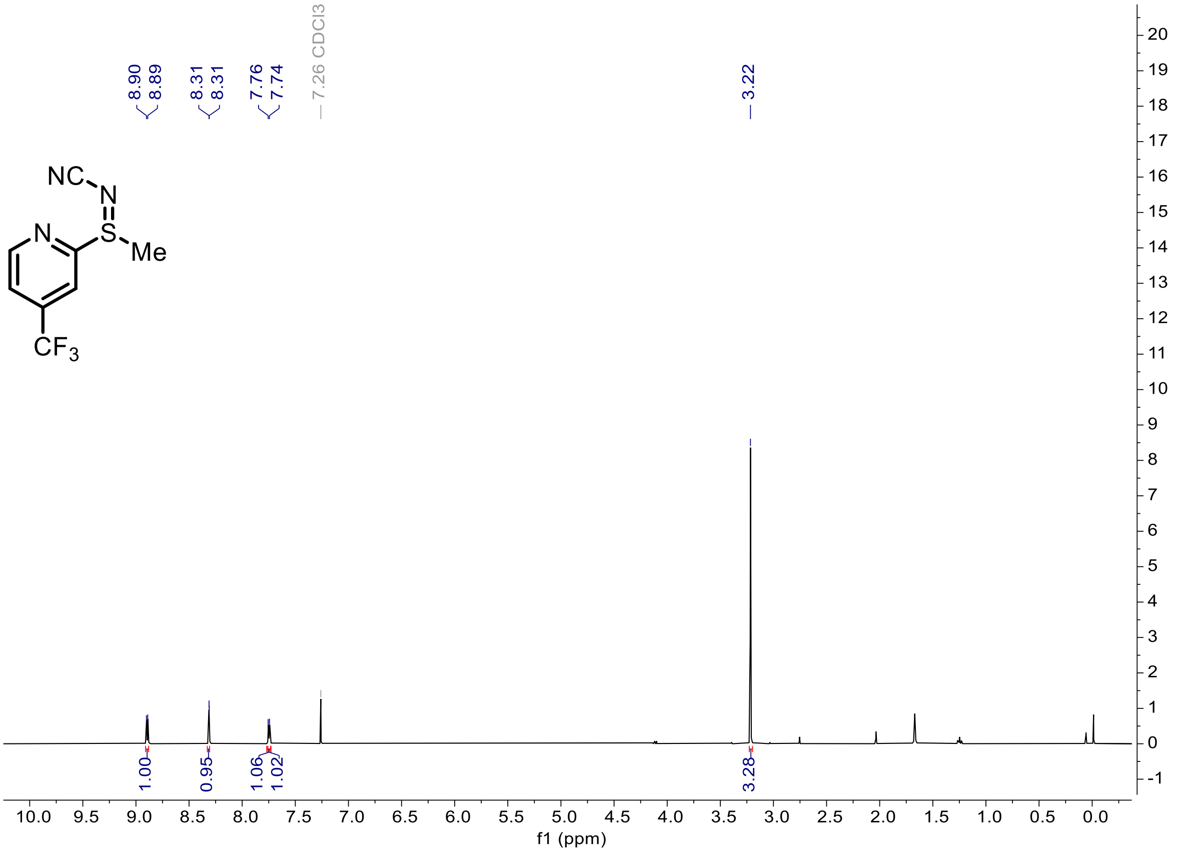

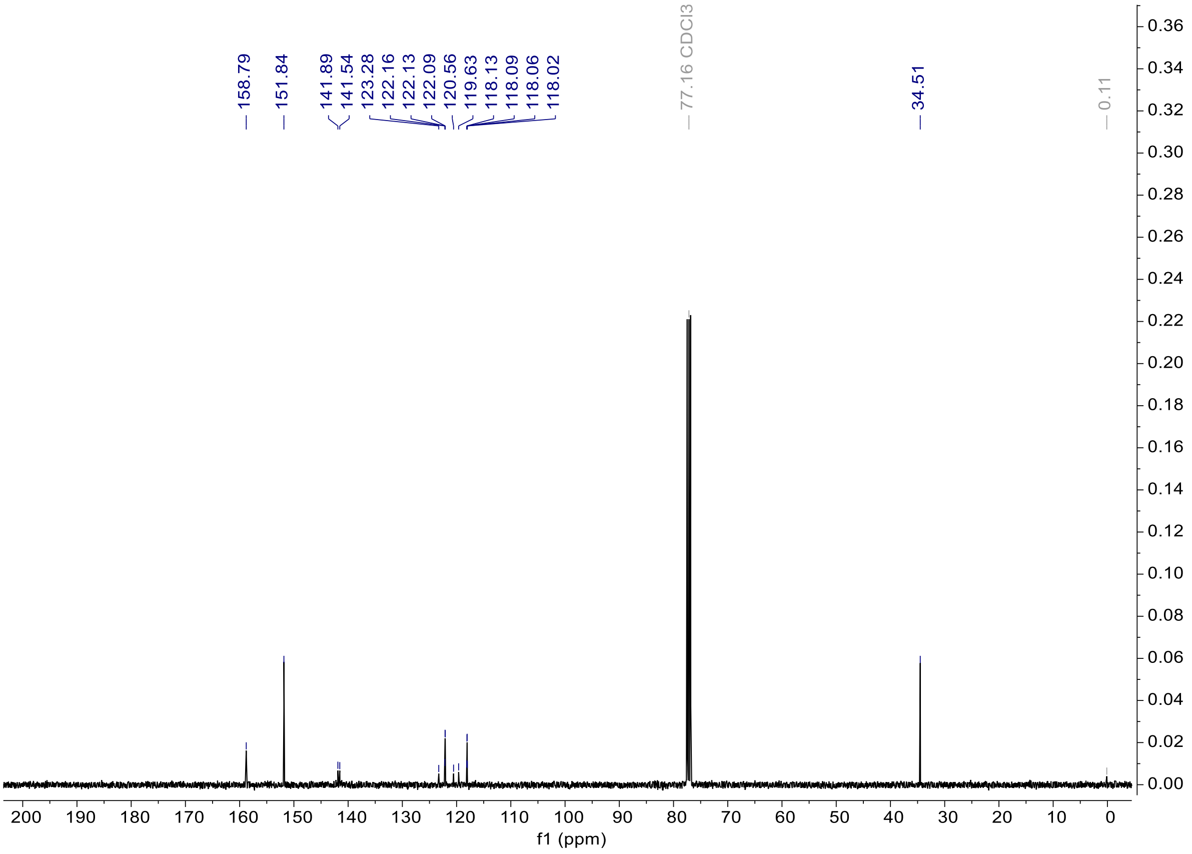

Figure S84: NMR data for **4m**

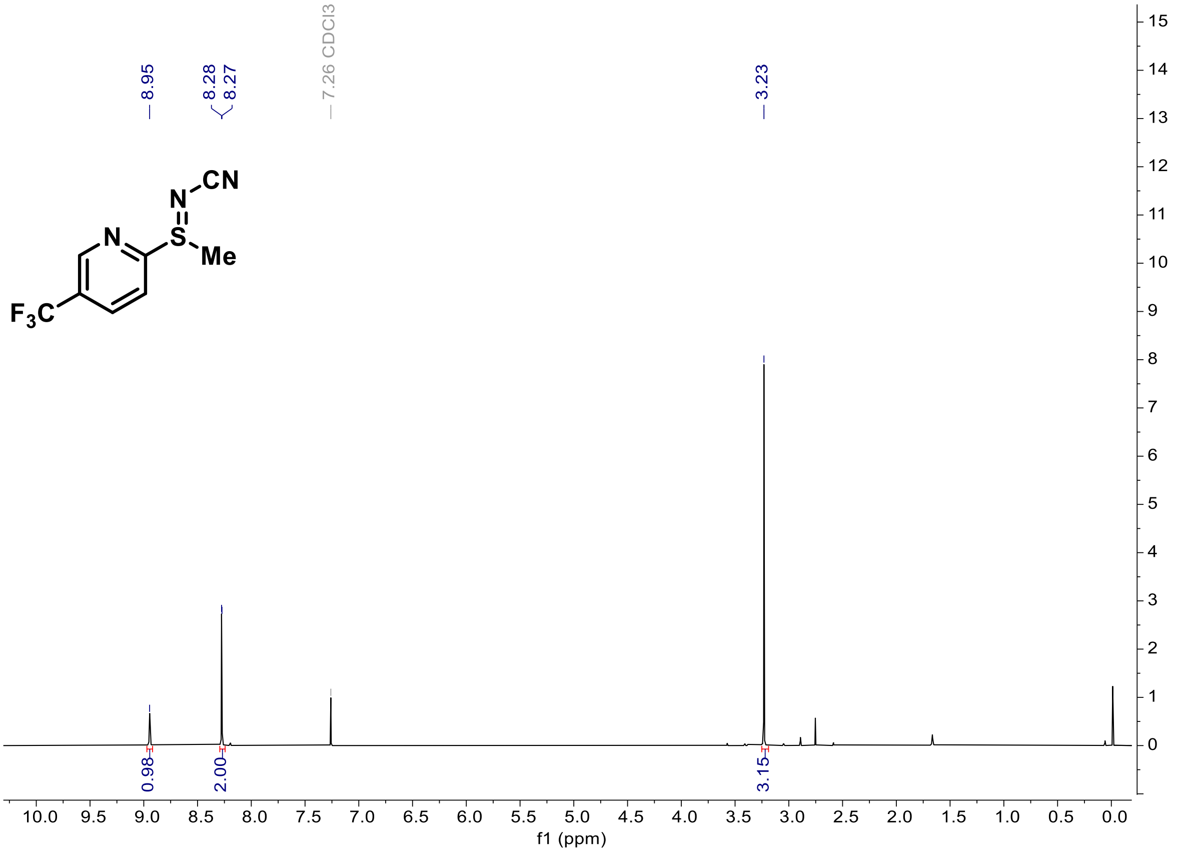

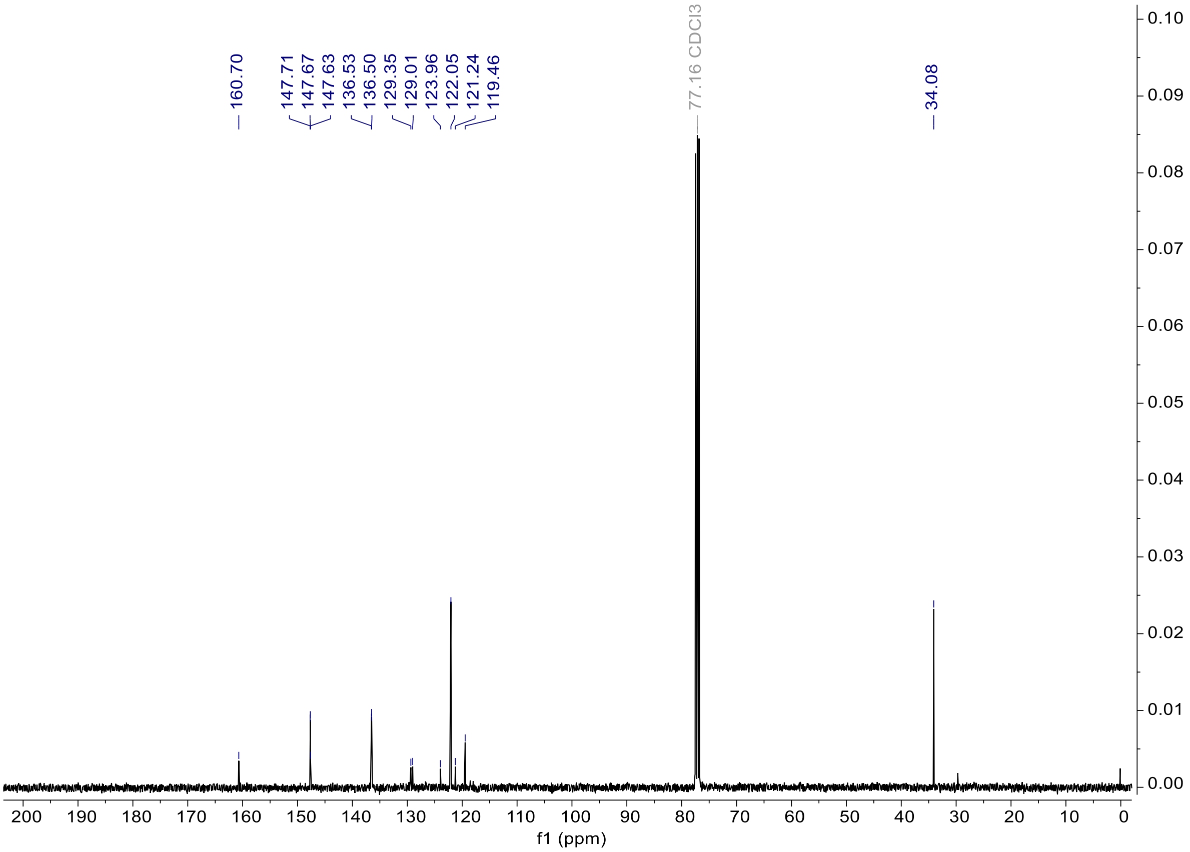

Figure S85: NMR data for **4n**

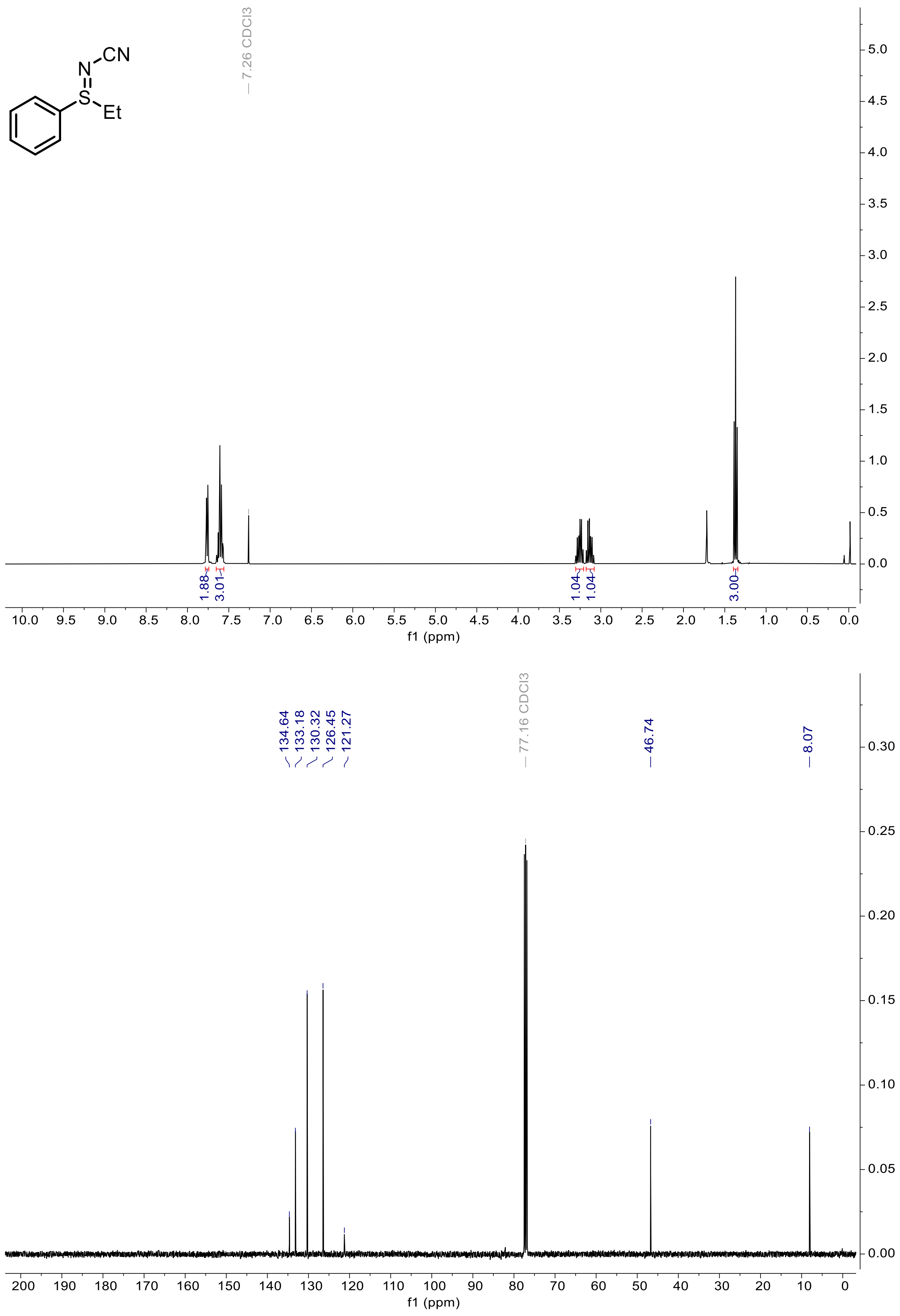

Figure S86: NMR data for **4o**

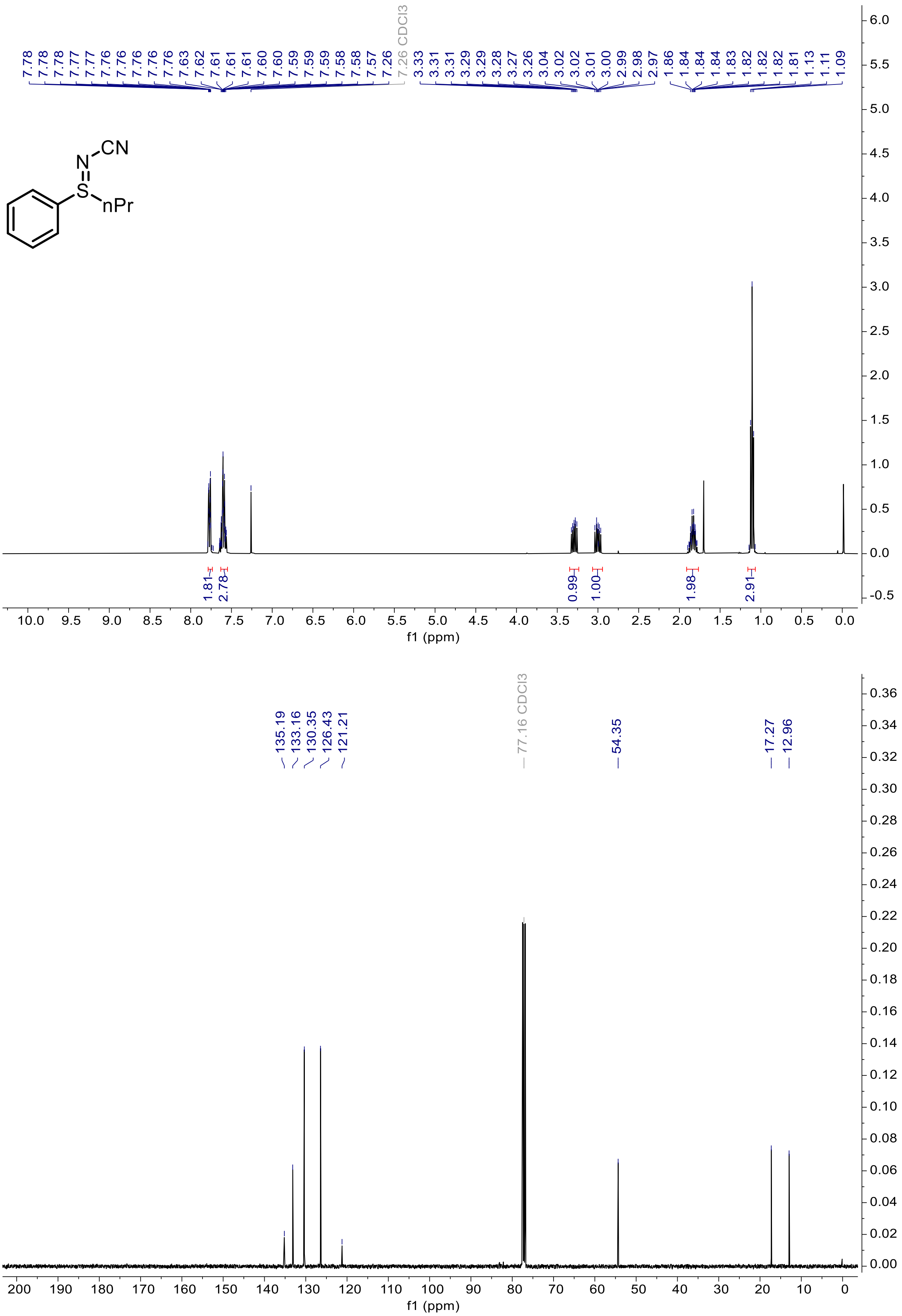

Figure S87: NMR data for **4p**

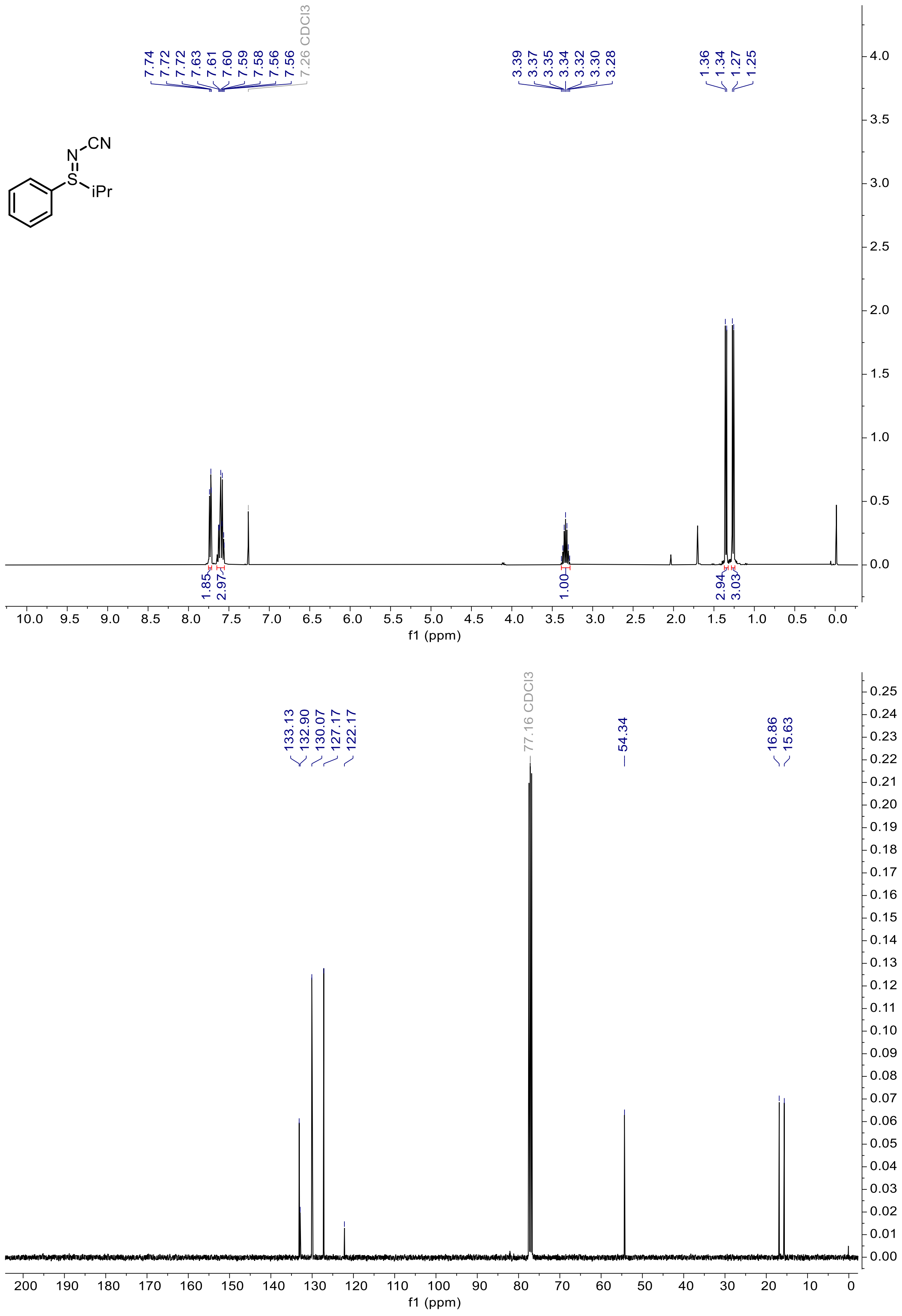

Figure S88: NMR data for **4q**

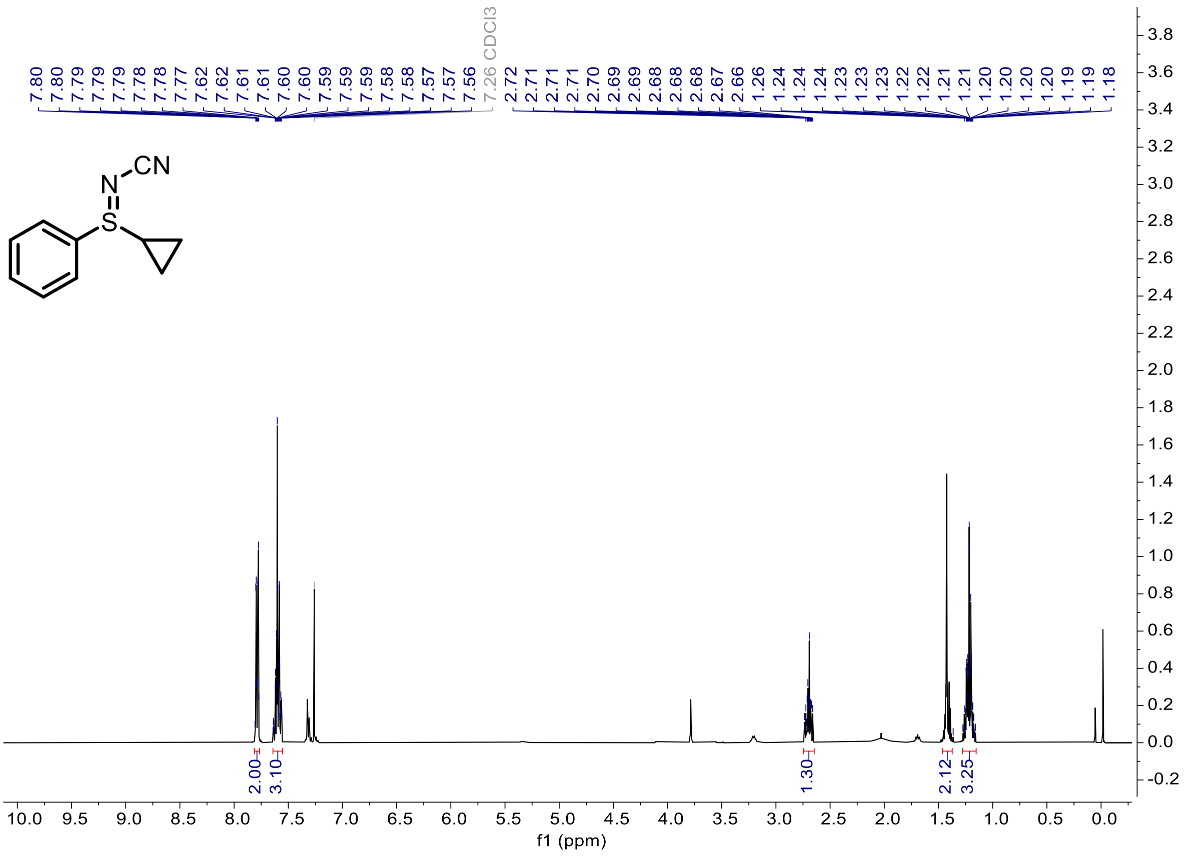

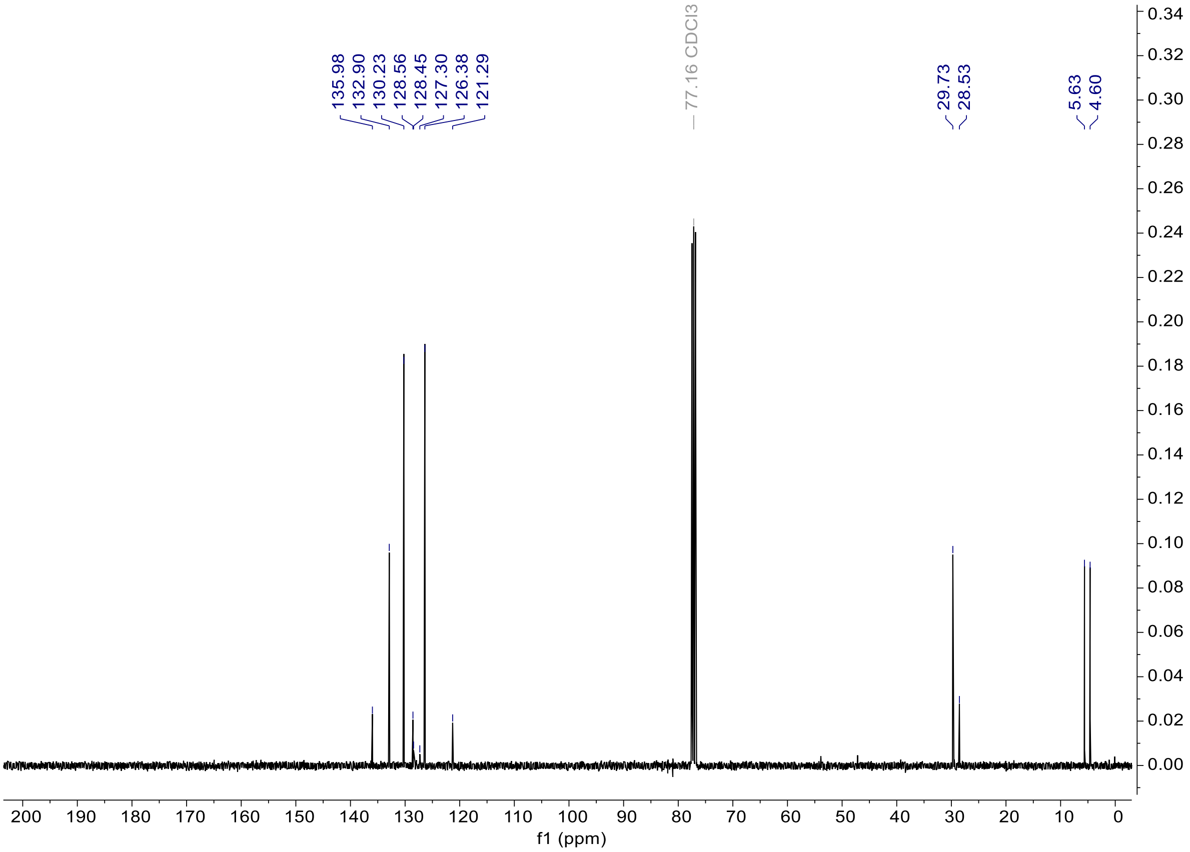

Figure S89: NMR data for **4r**

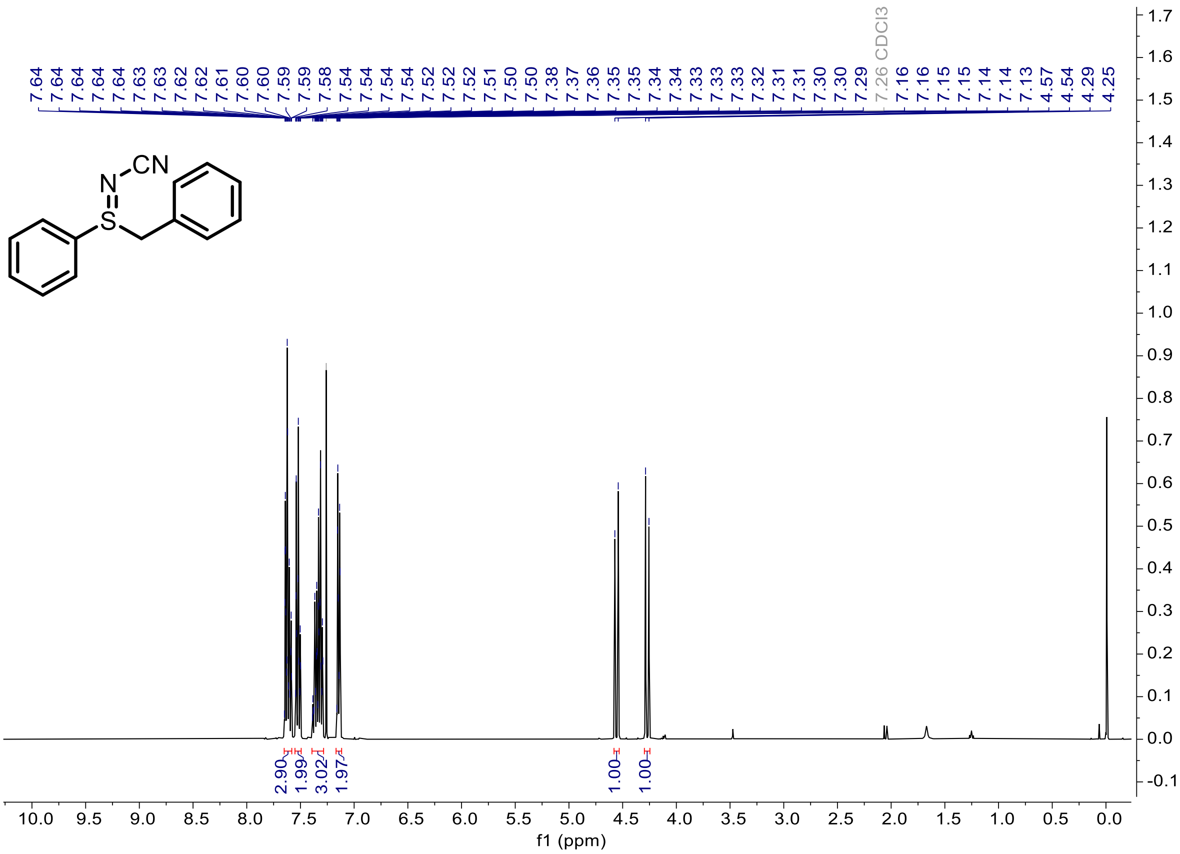

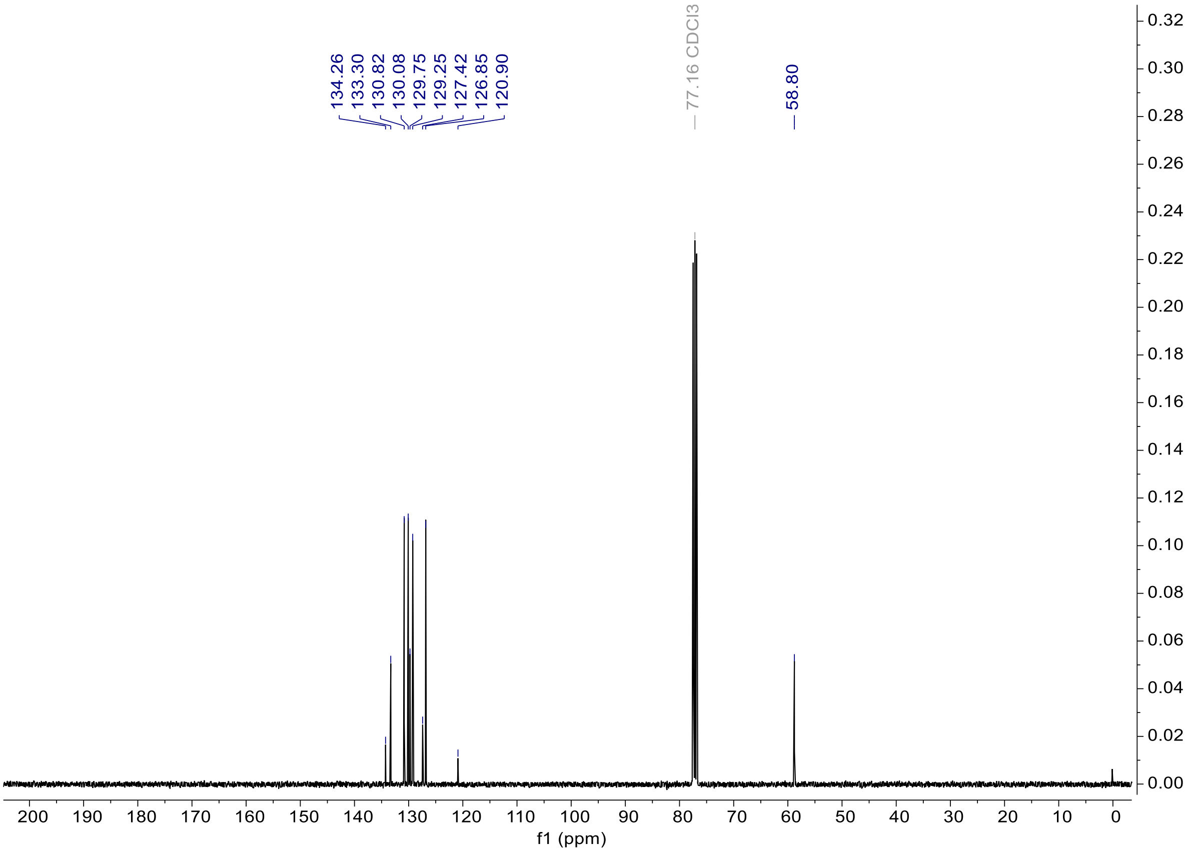

Figure S90: NMR data for **4s**

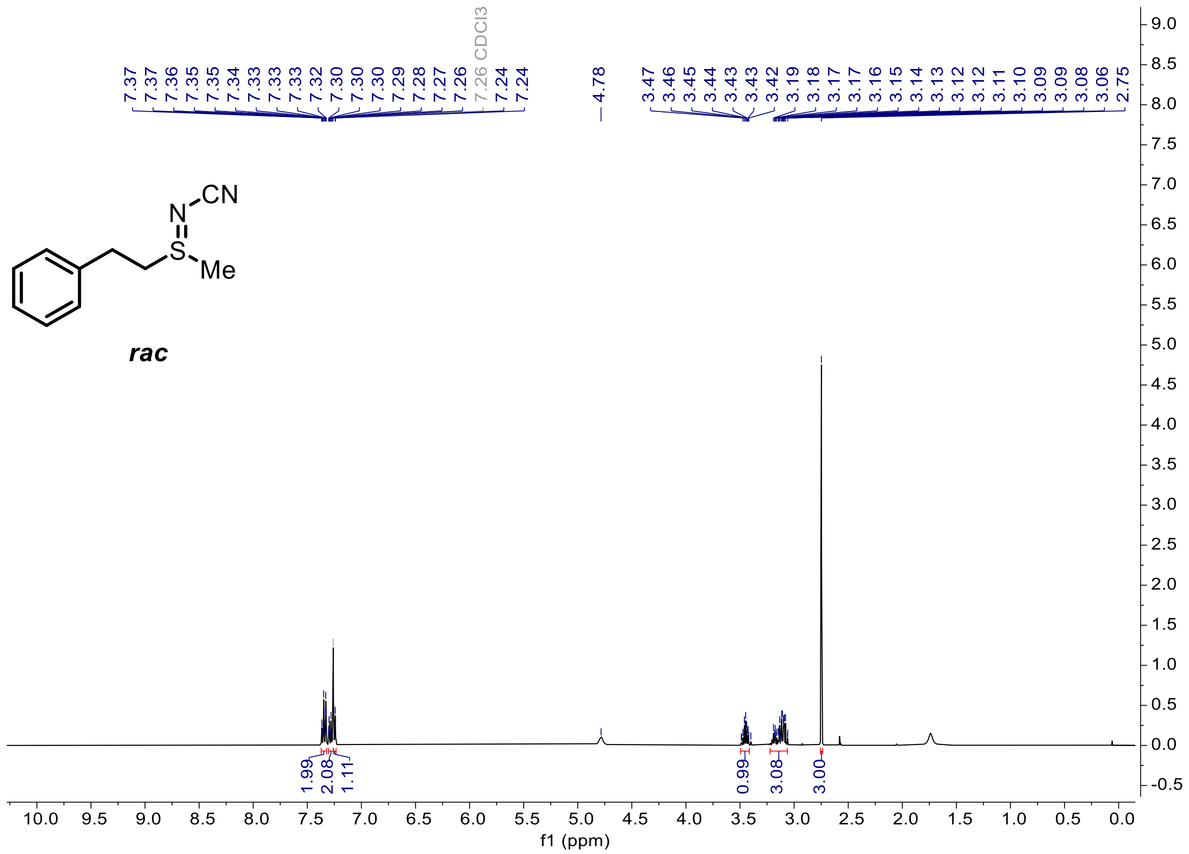

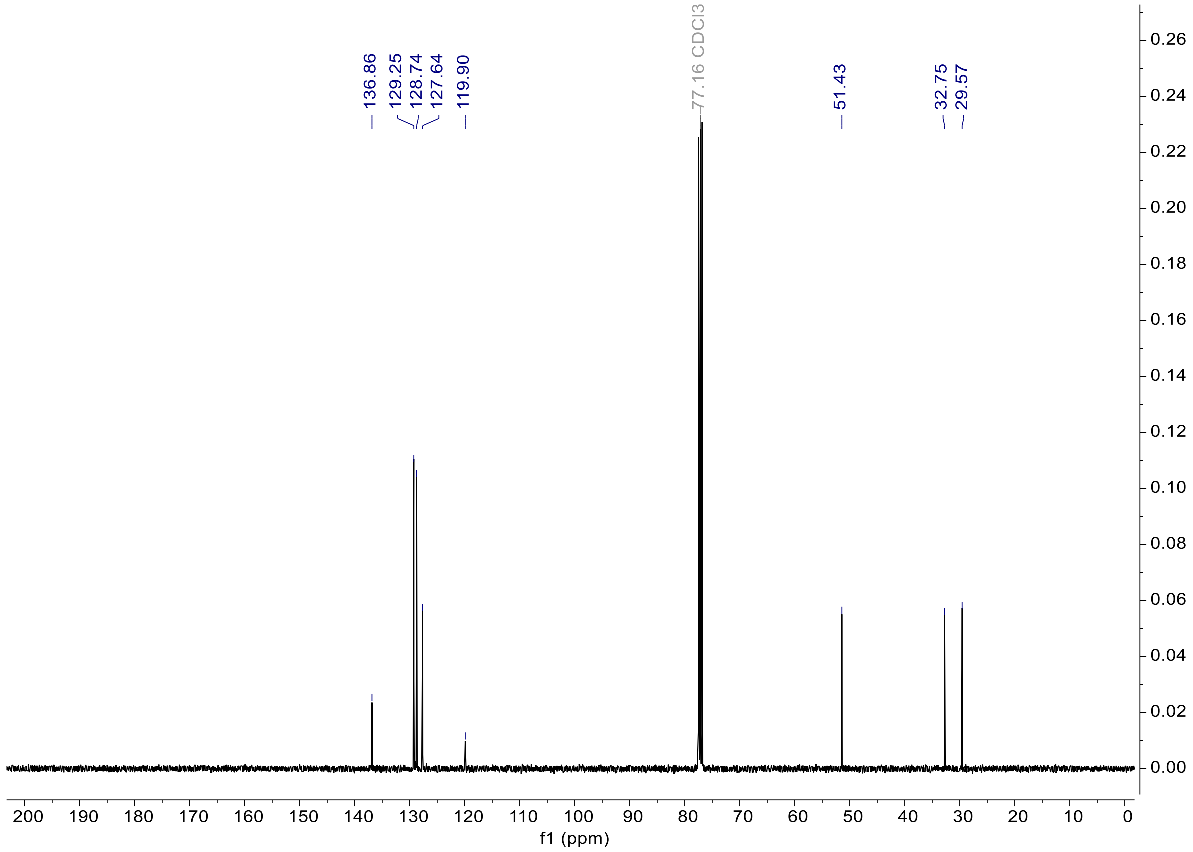

Figure S91: NMR data for **4t**

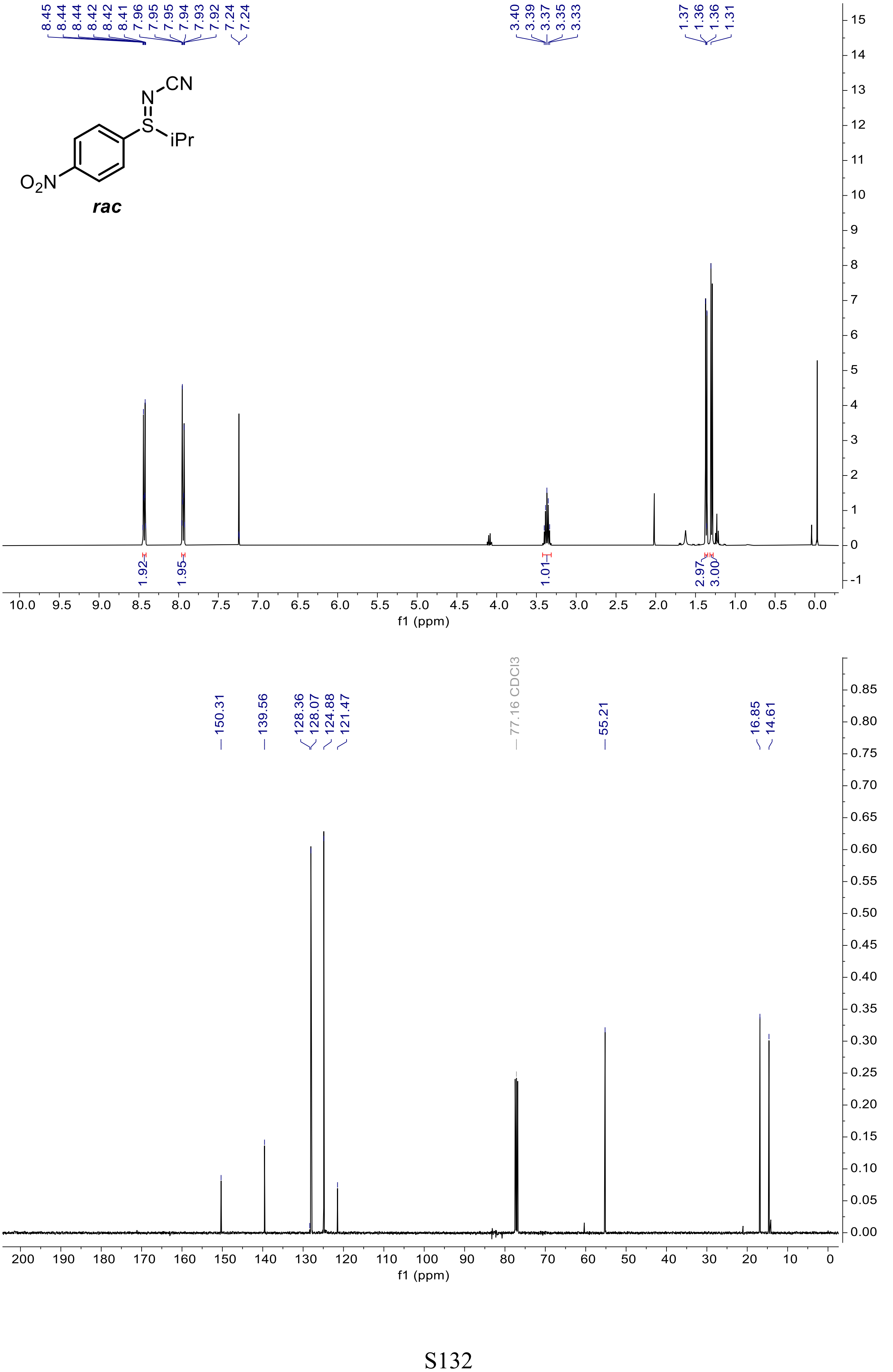

Figure S92: NMR data for **4u**

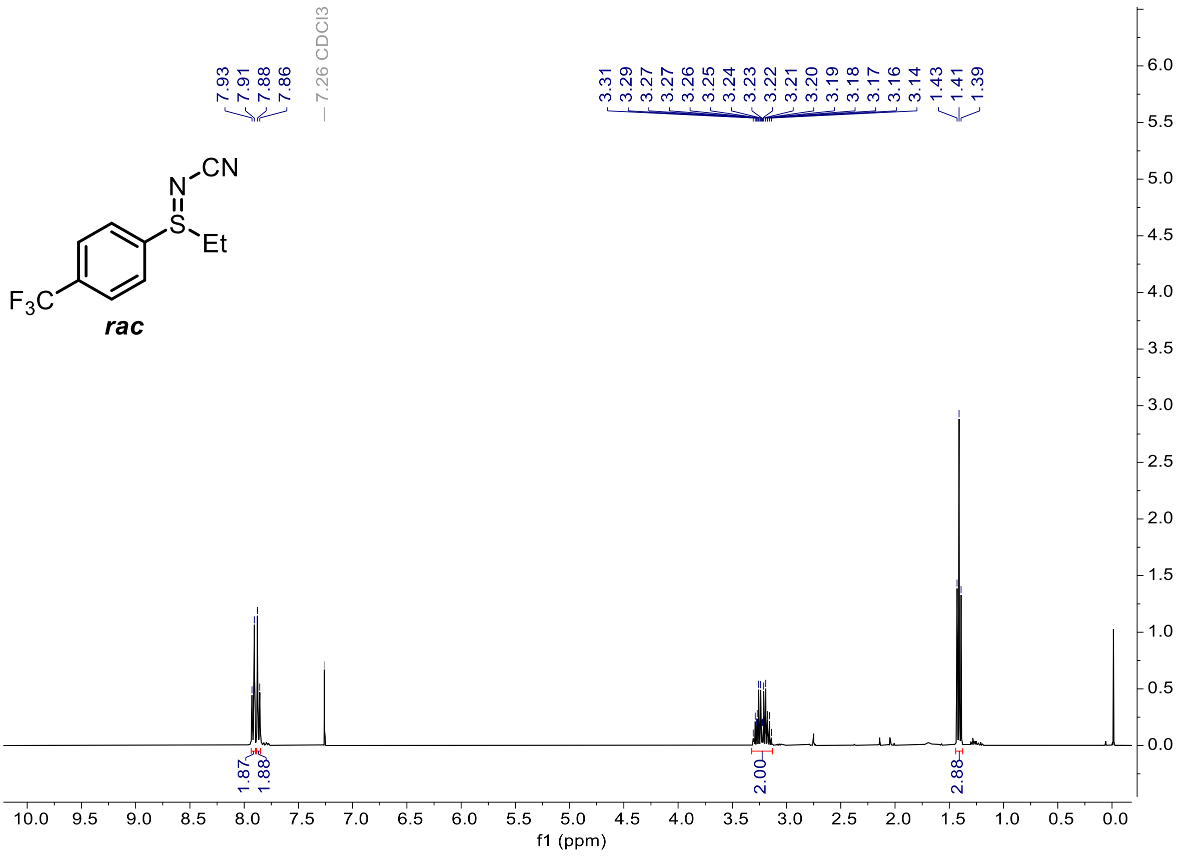

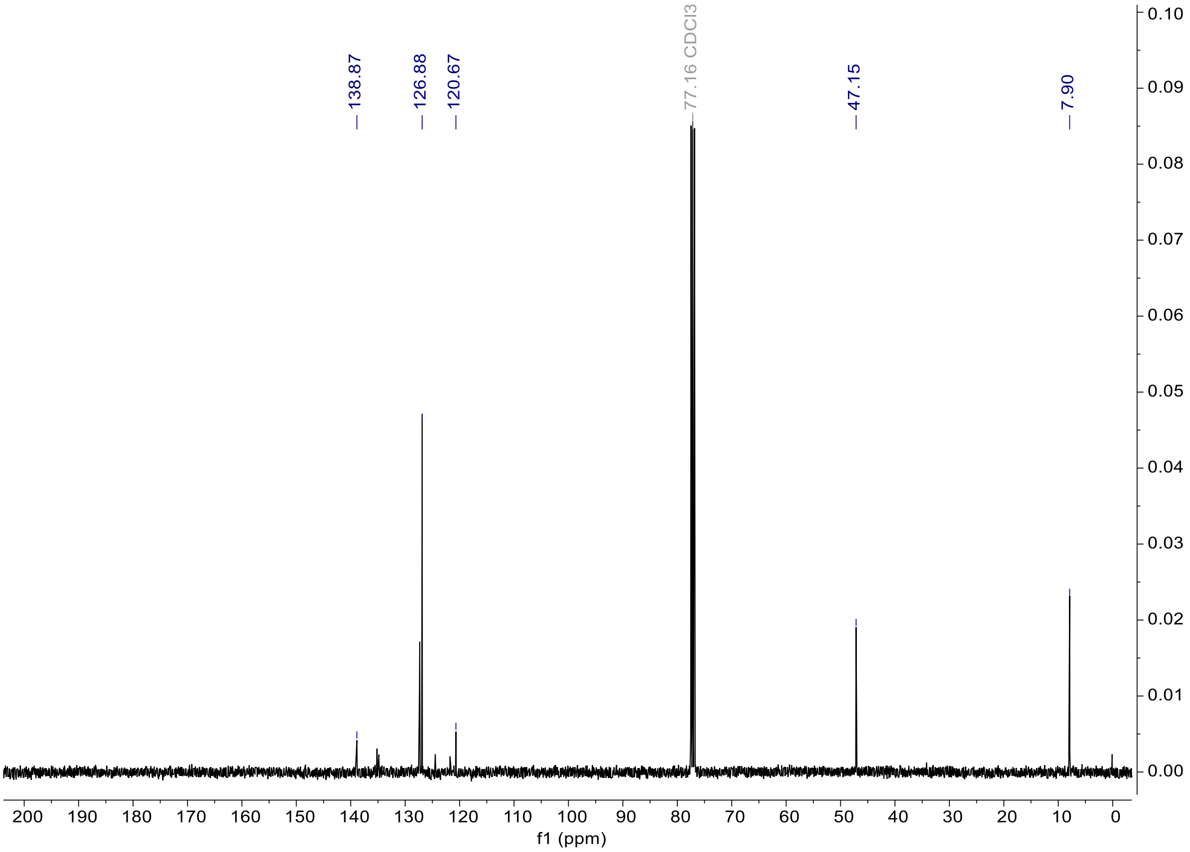

Figure S93: NMR data for **5a**

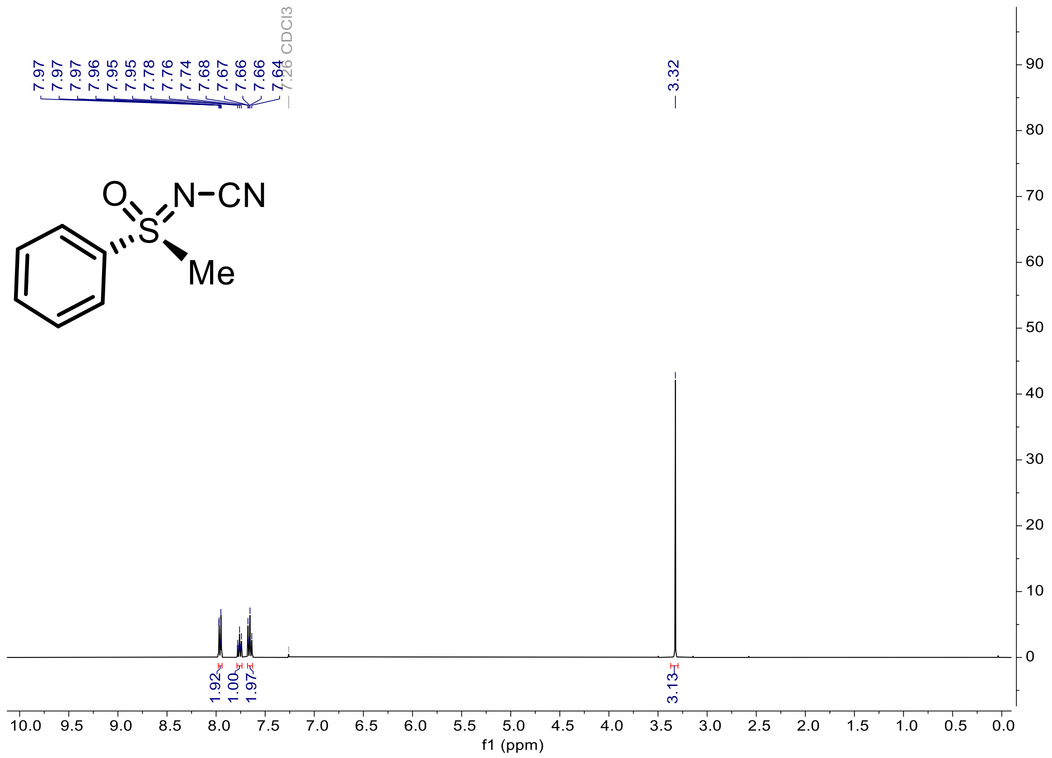

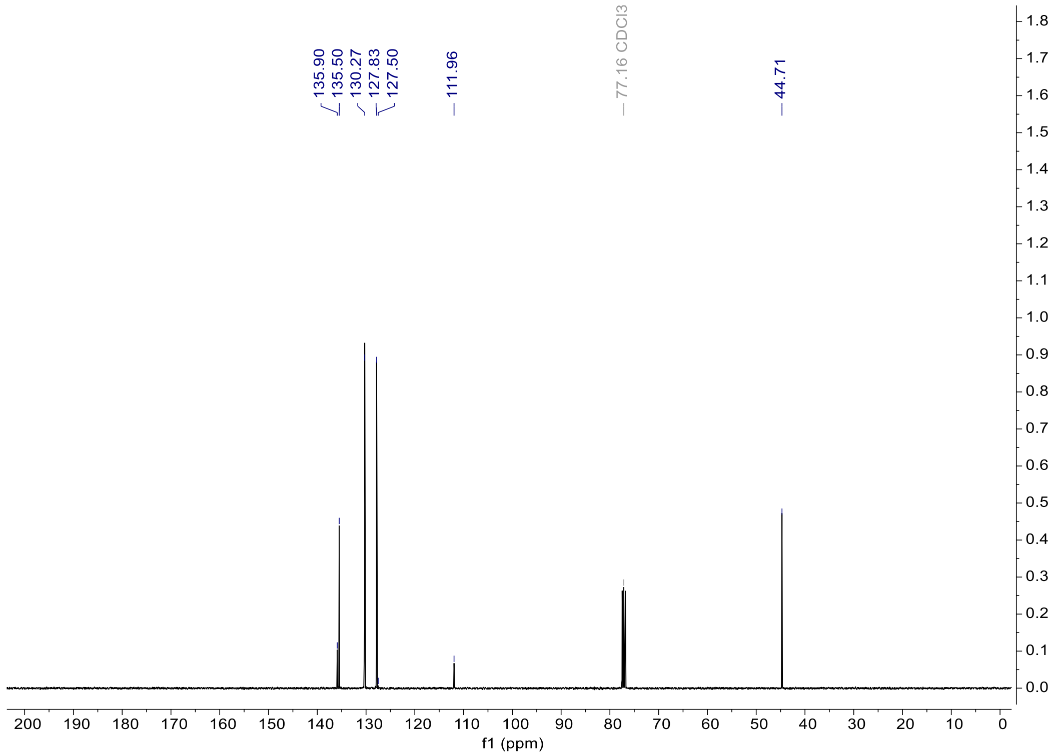

Figure S94: NMR data for **5b**

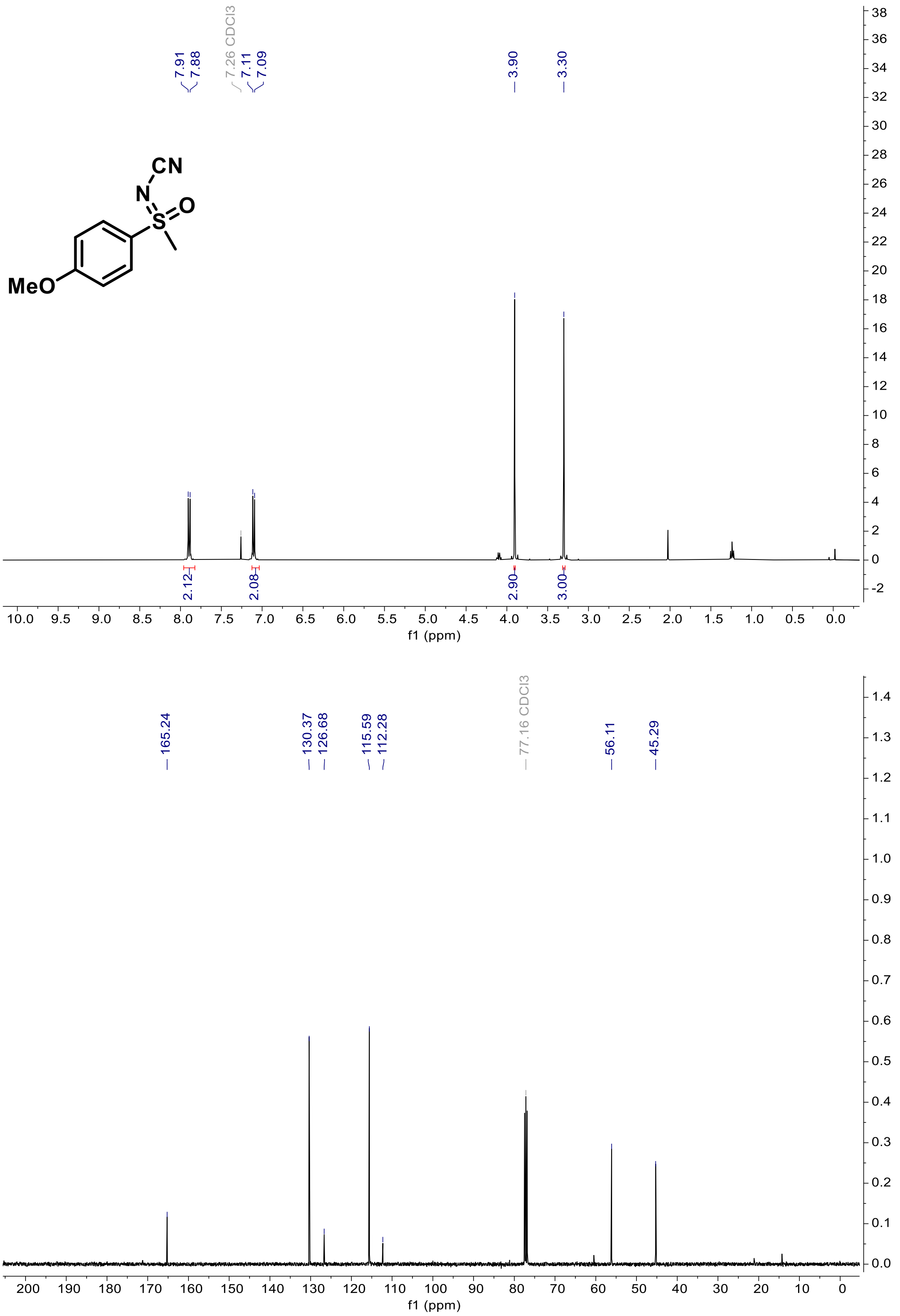

Figure S95: NMR data for **5c**

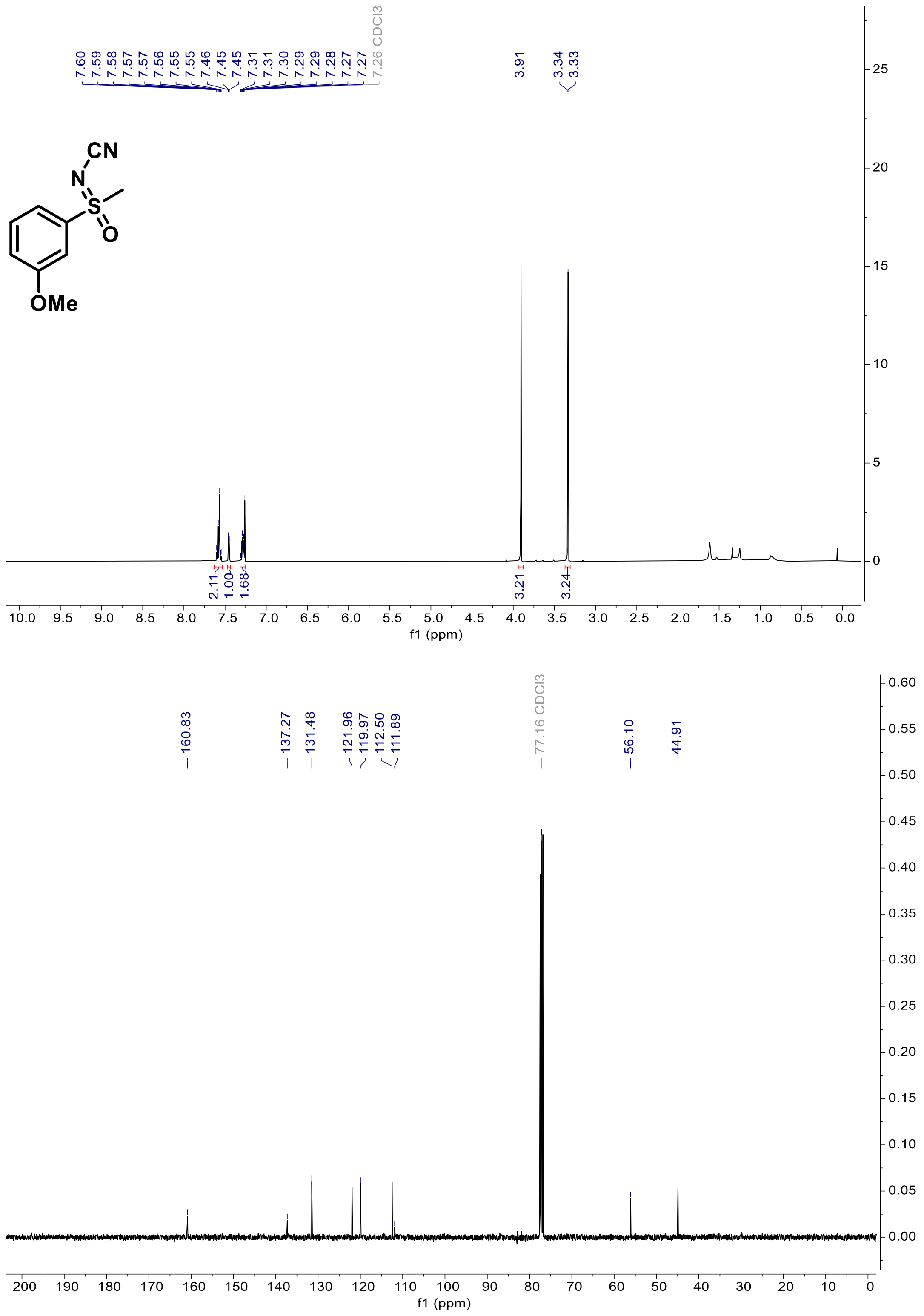

Figure S96: NMR data for **5d**

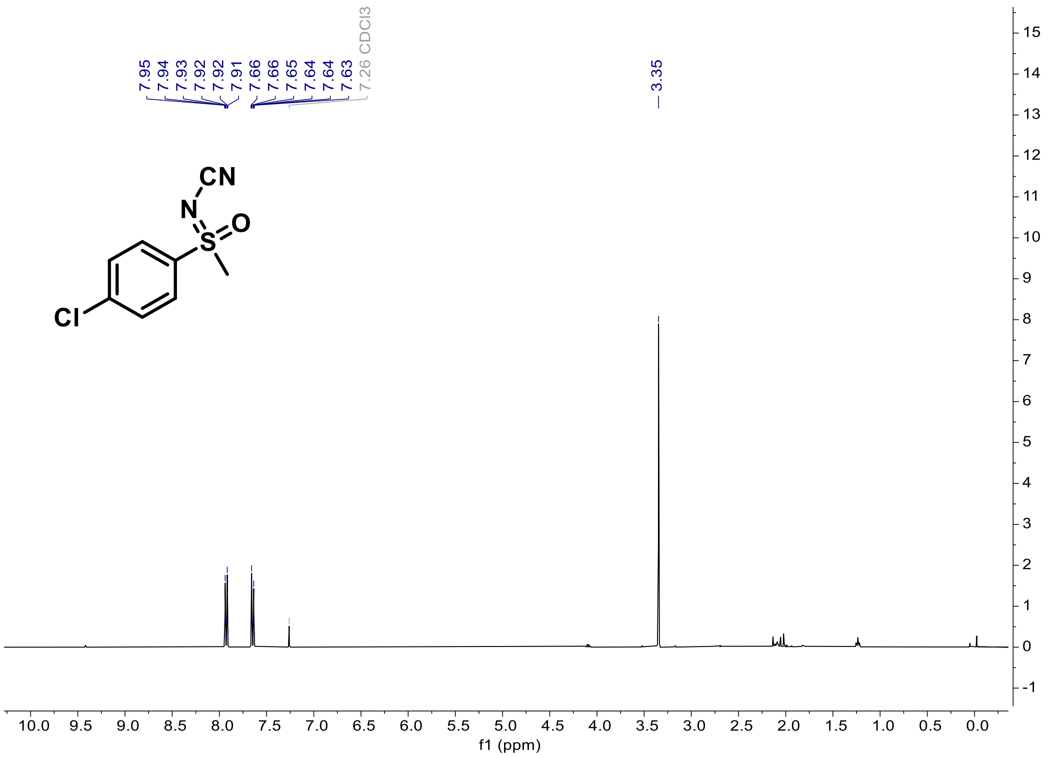

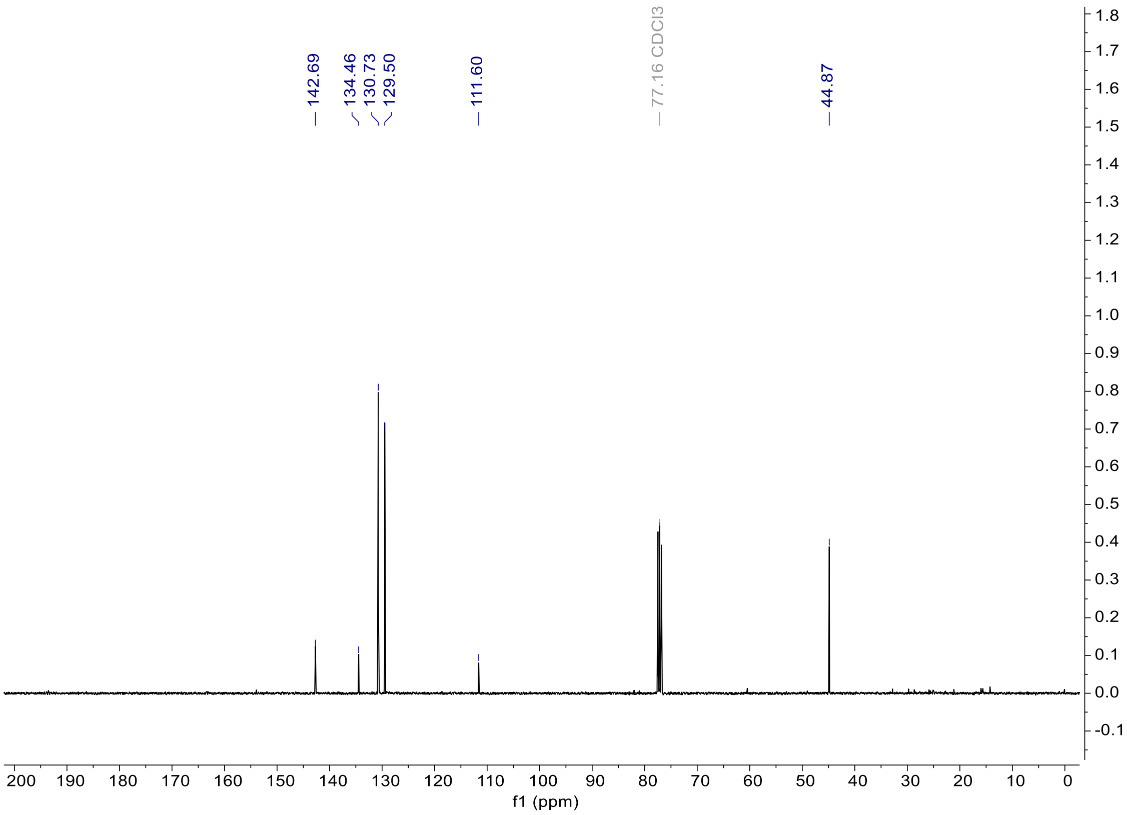

Figure S97: NMR data for **5e**

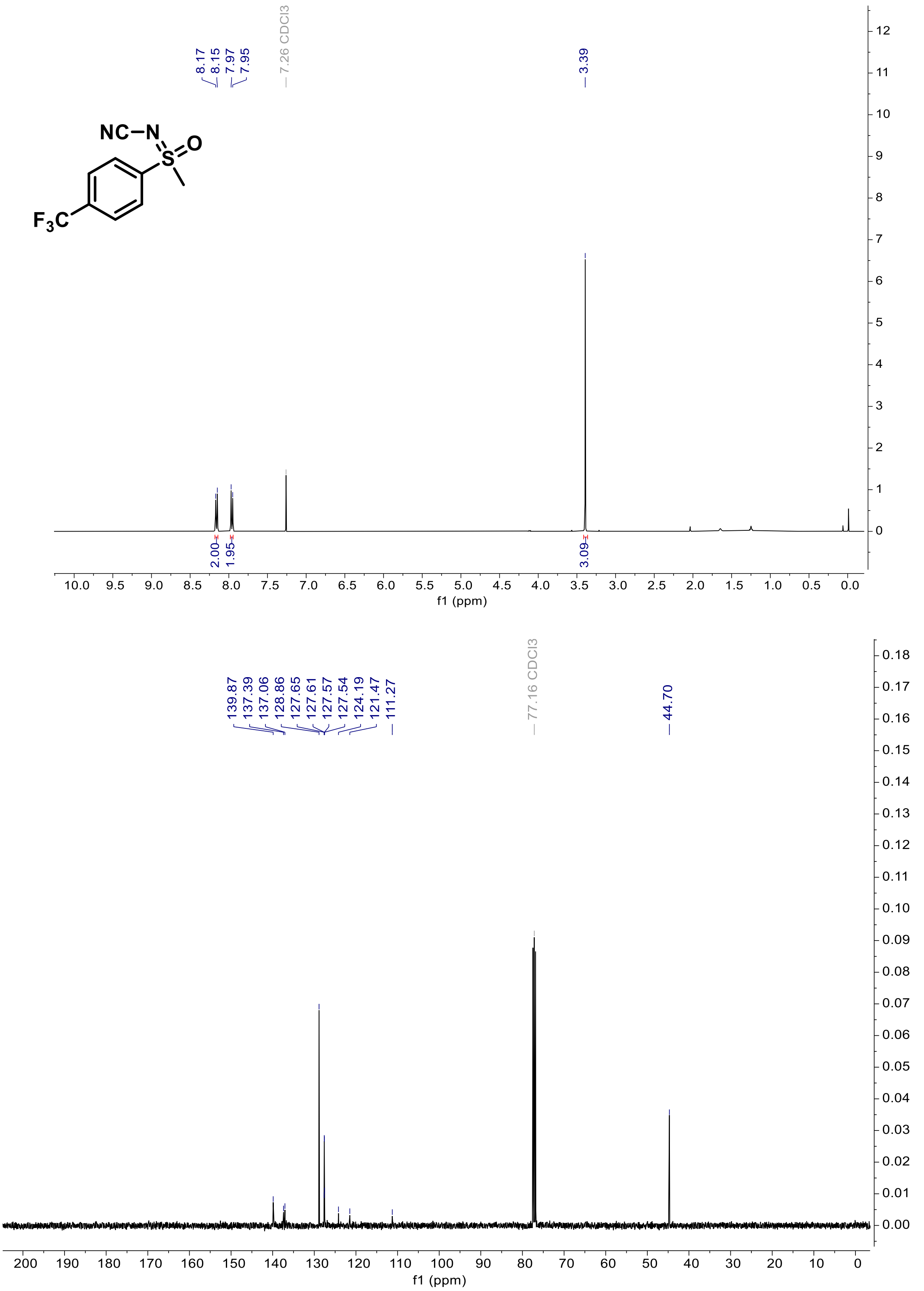

Figure S98: NMR data for **5f**

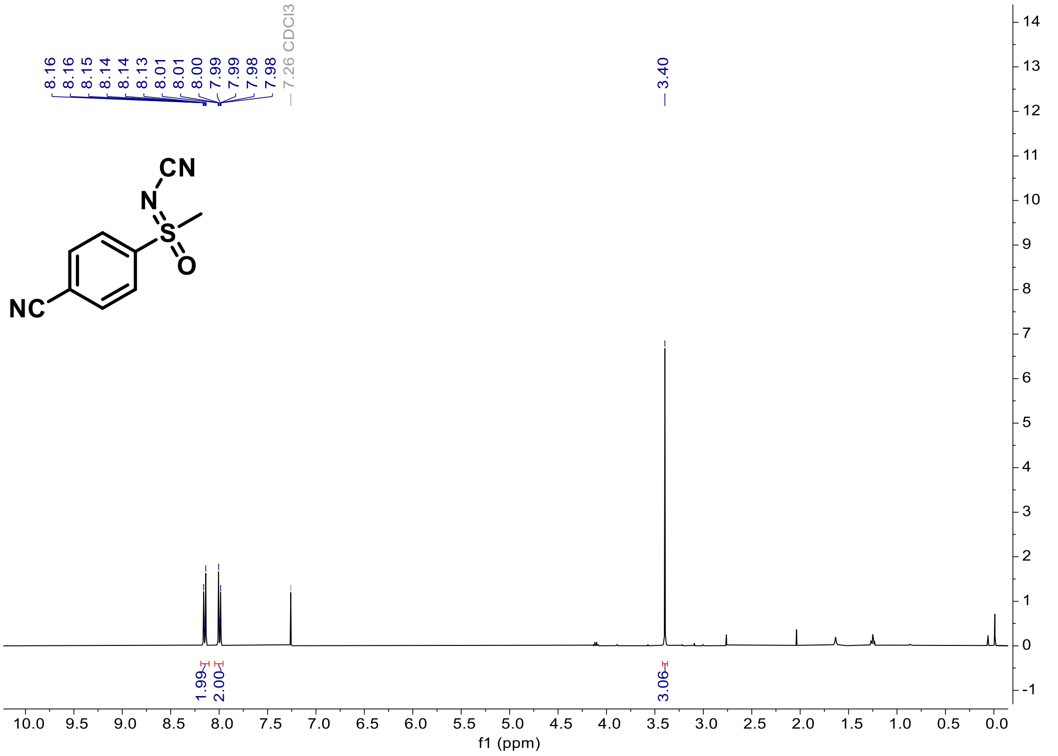

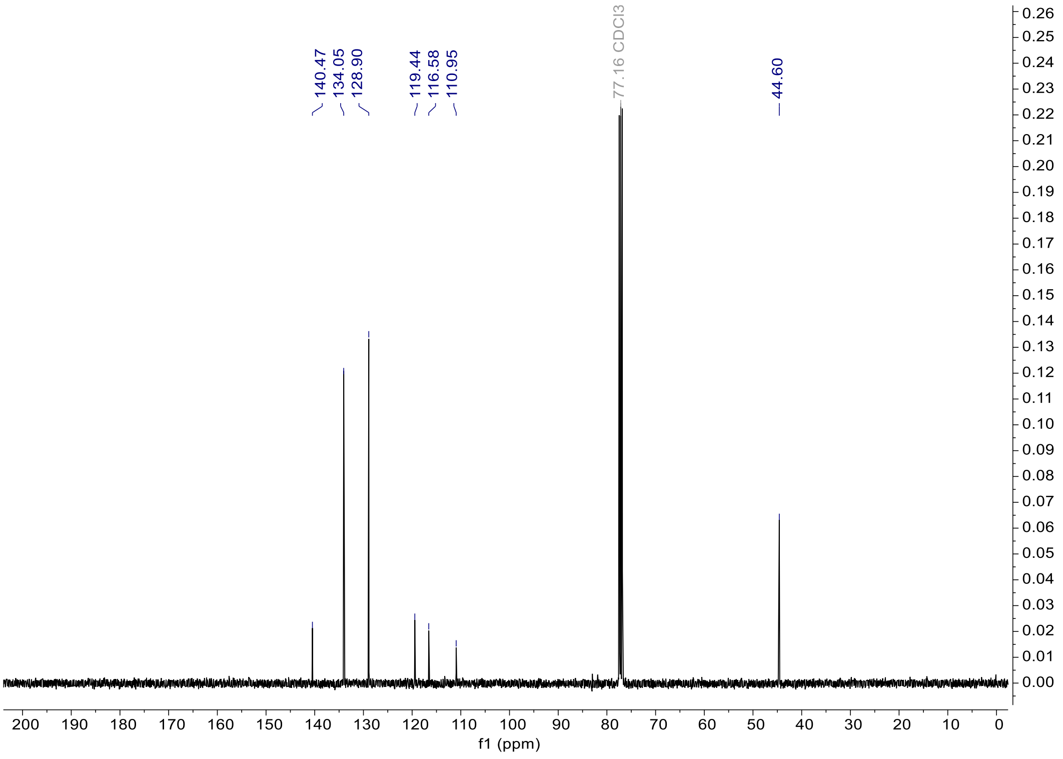

Figure S99: NMR data for **5g**

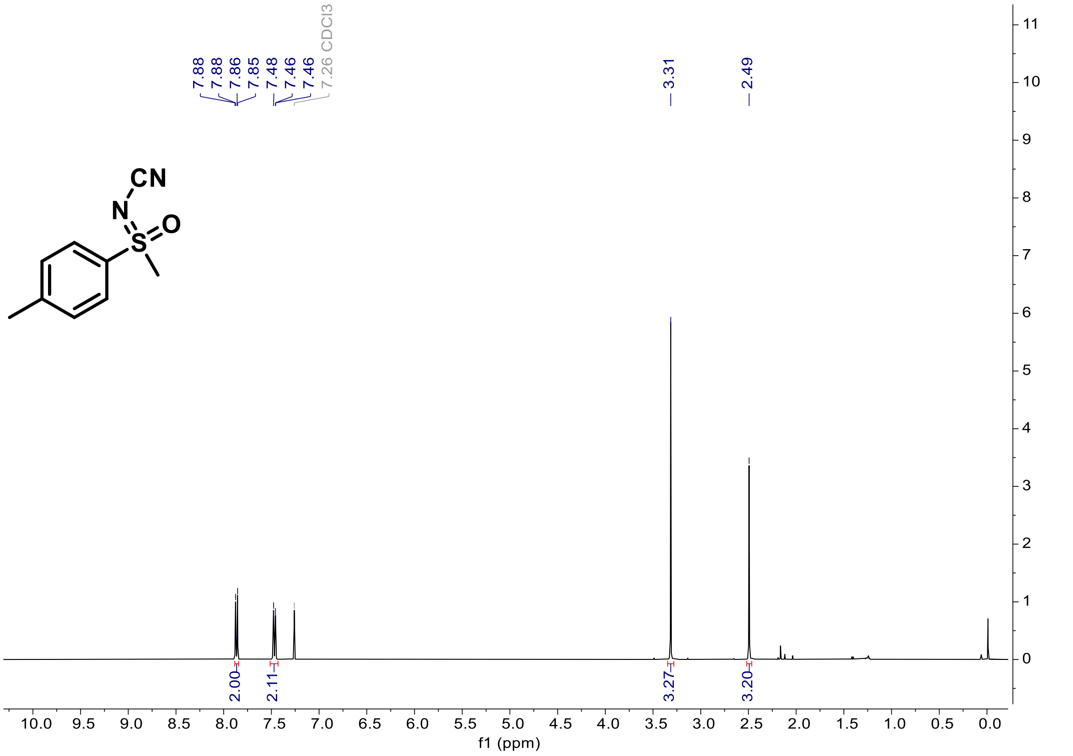

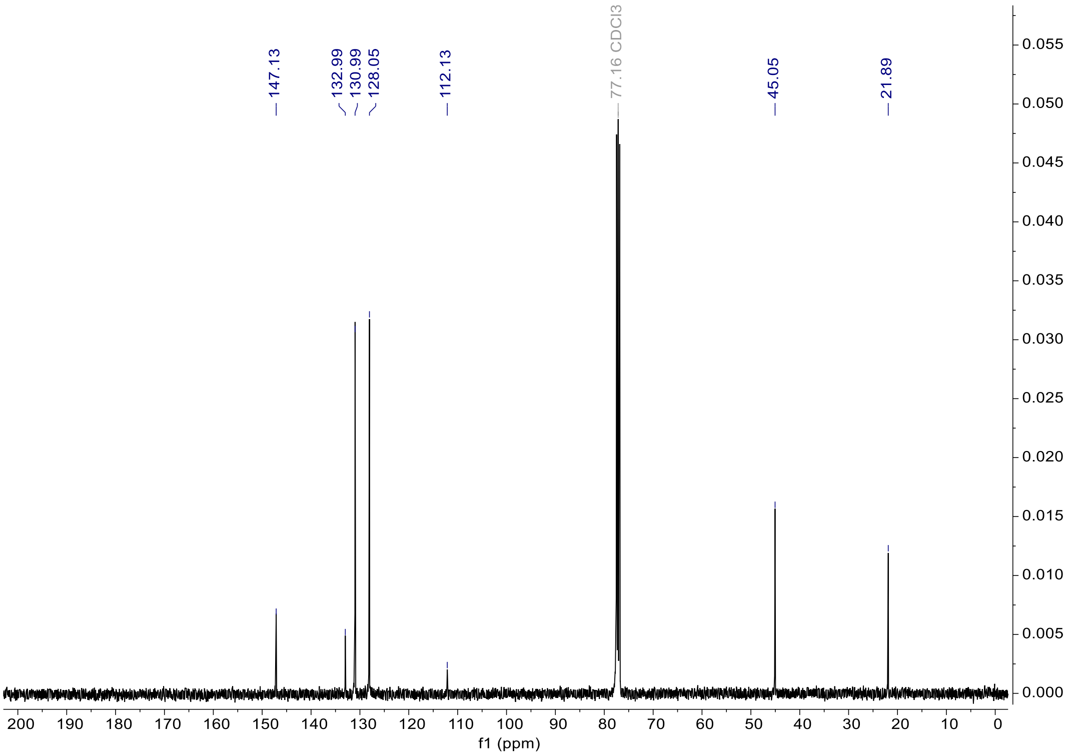

Figure S100: NMR data for **5h**:

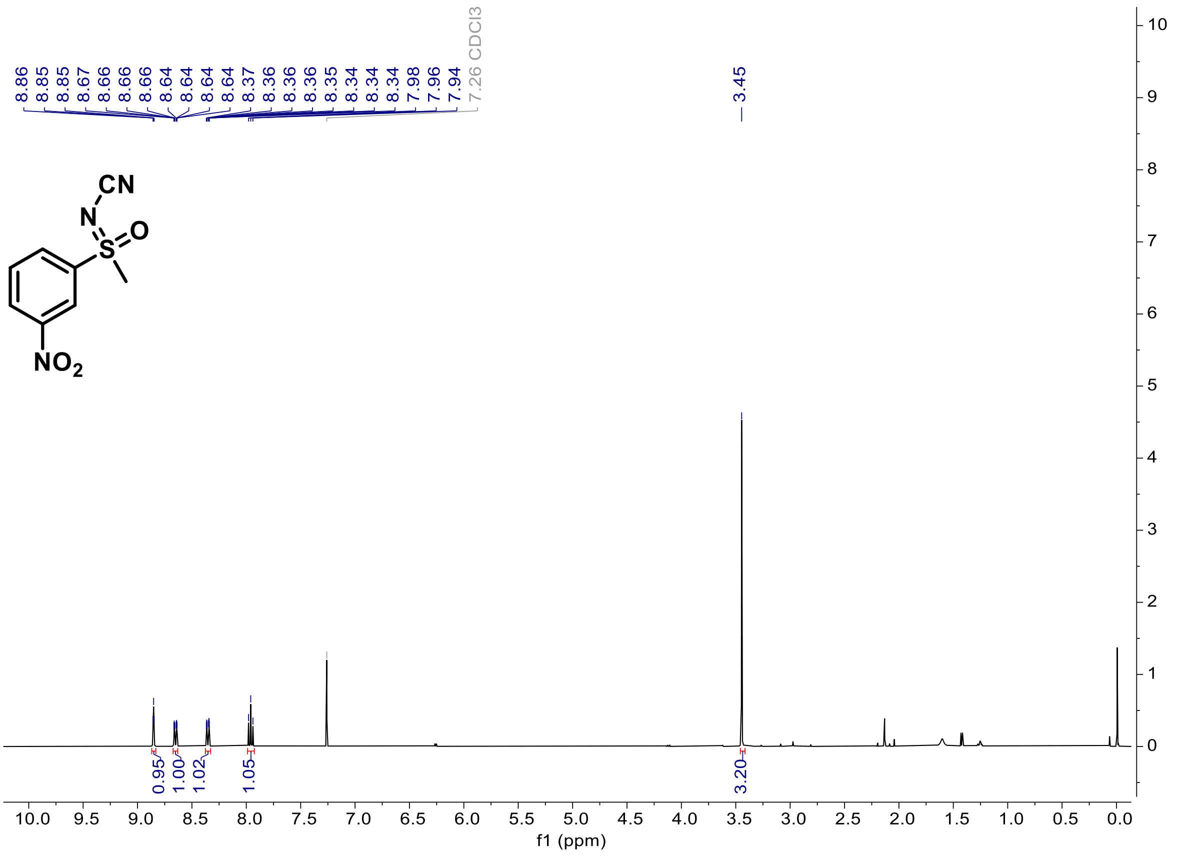

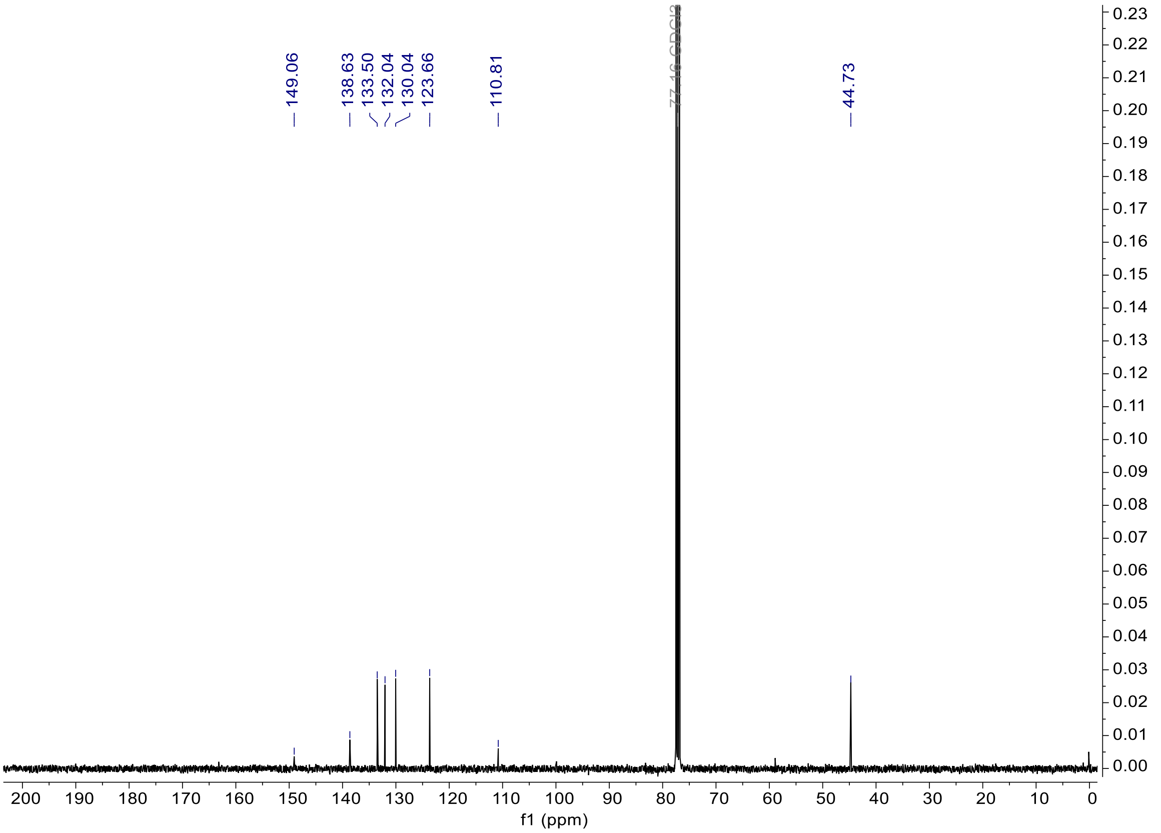

Figure S101: NMR data for **5i**

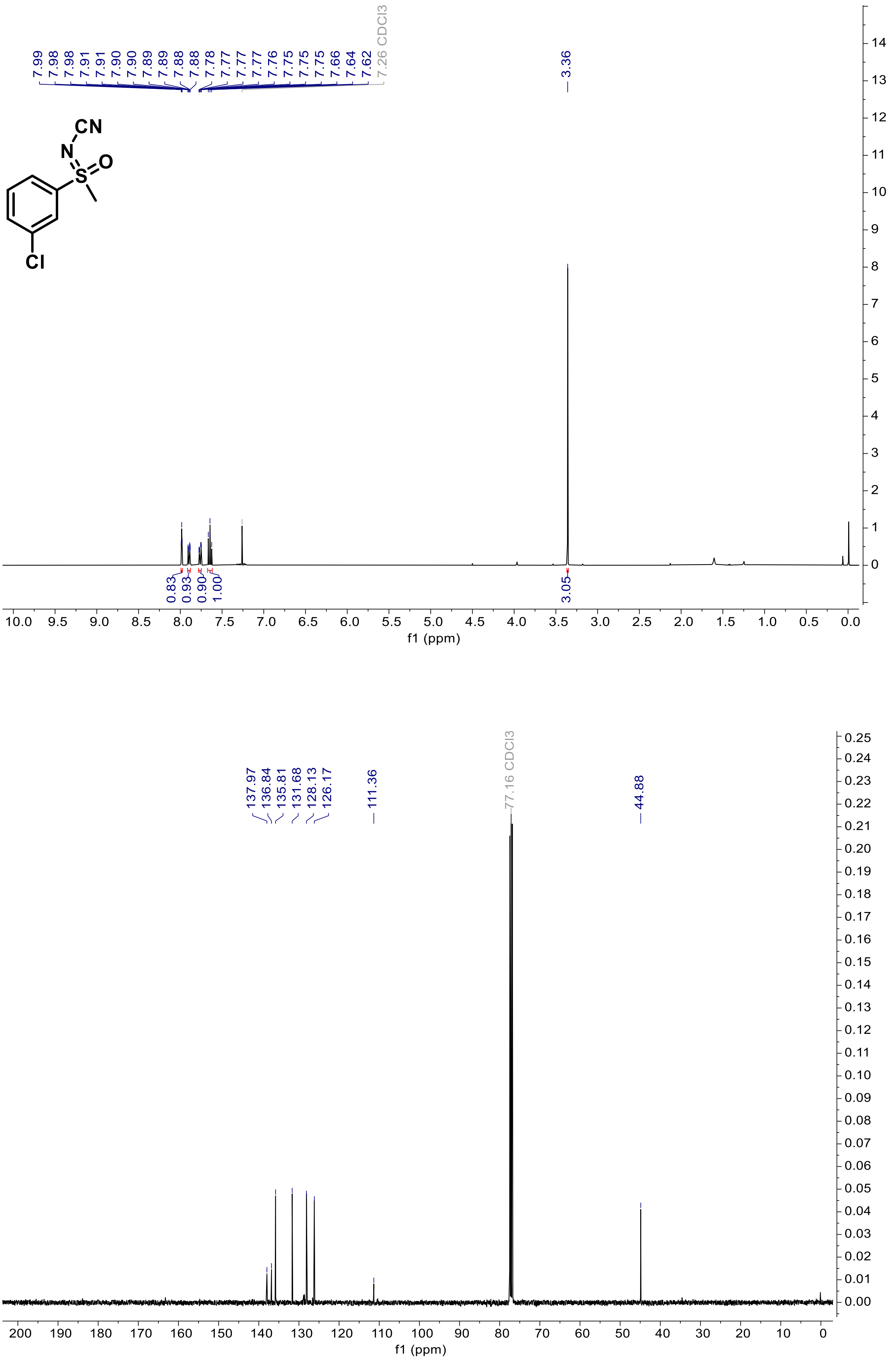

Figure S102: NMR data for **5j**

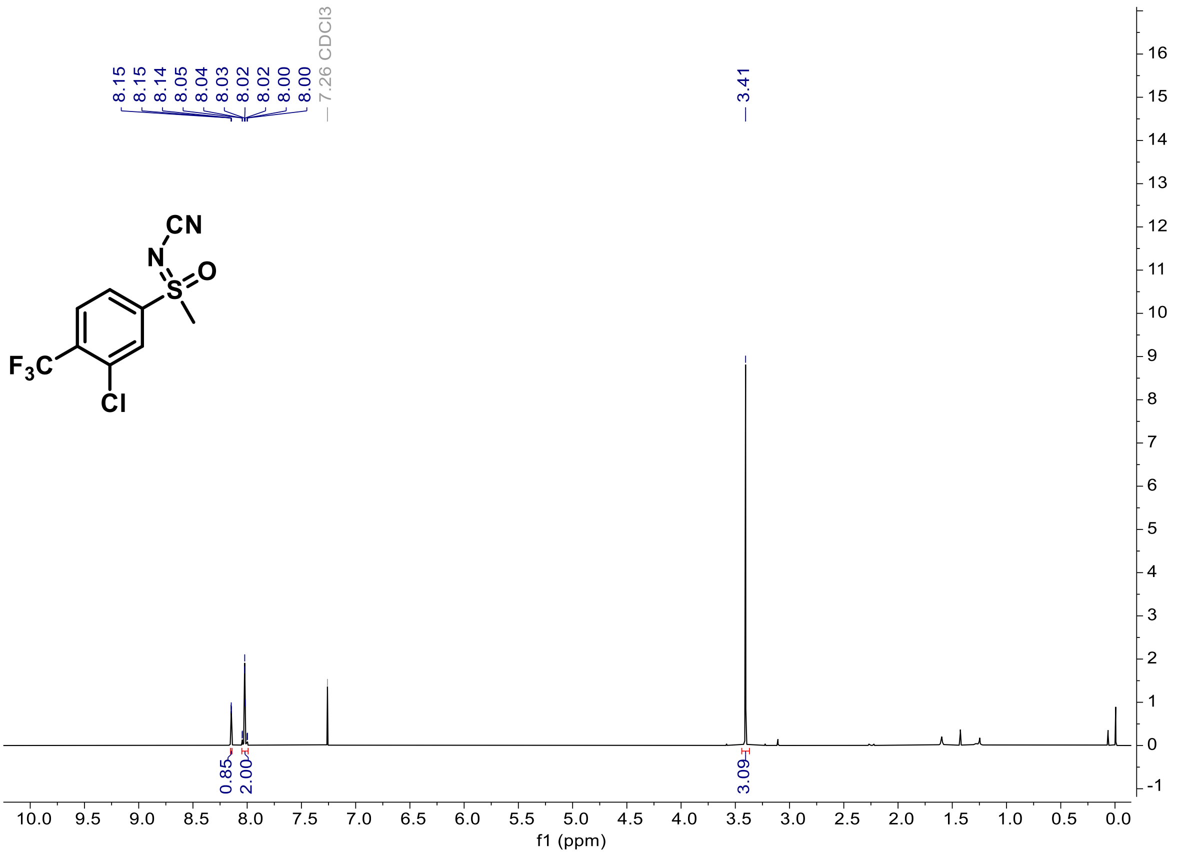

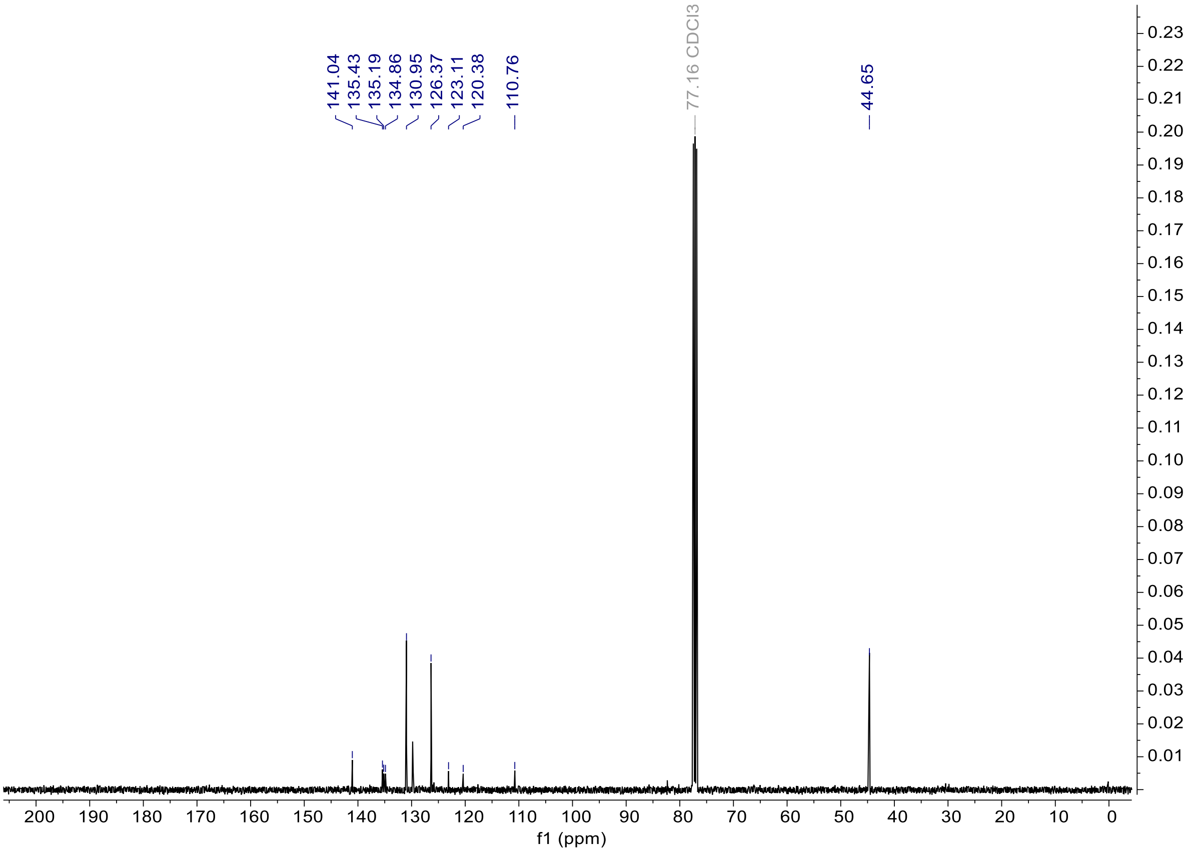

Figure S103: NMR data for **5k**

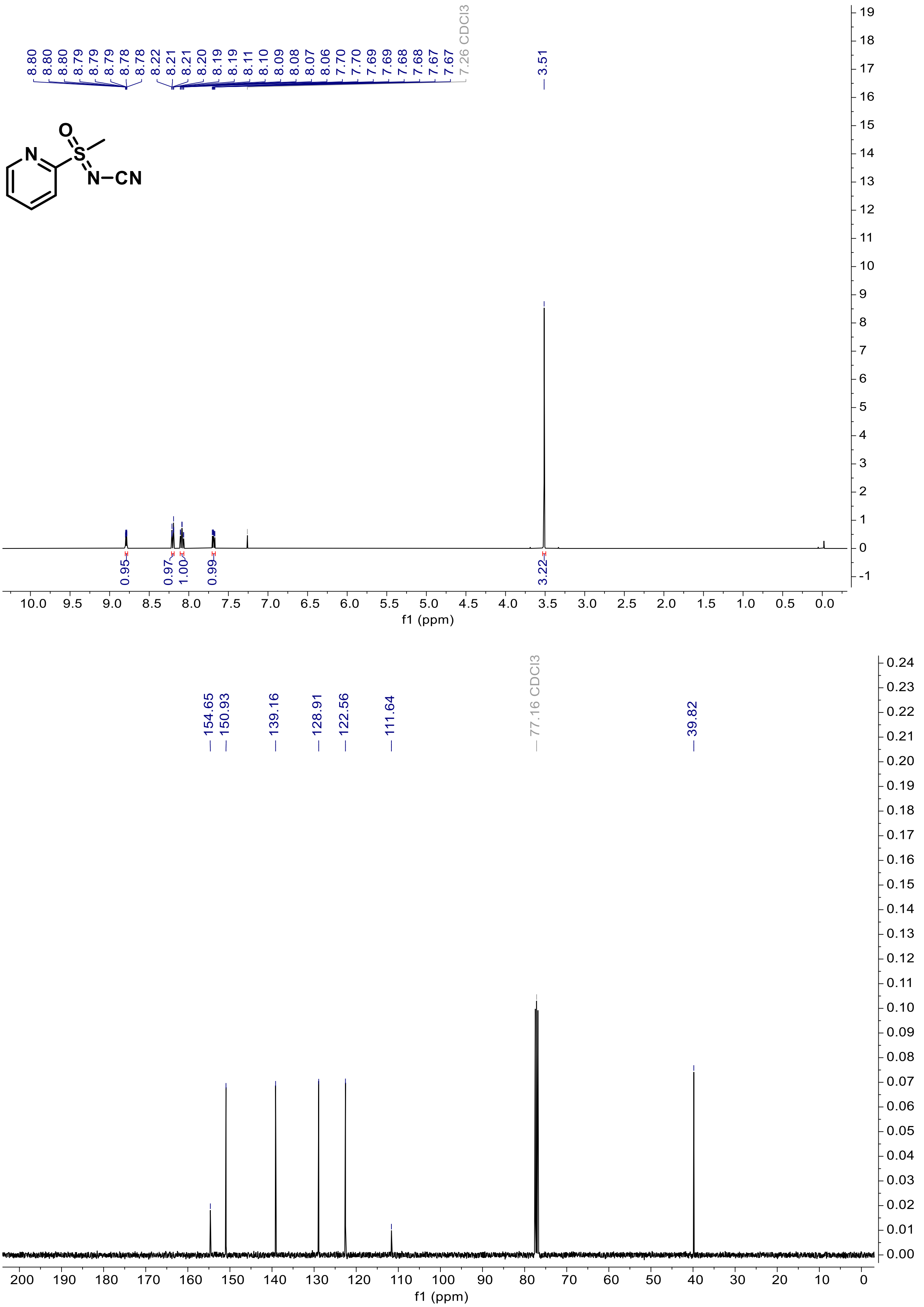

Figure S104: NMR data for **5l**

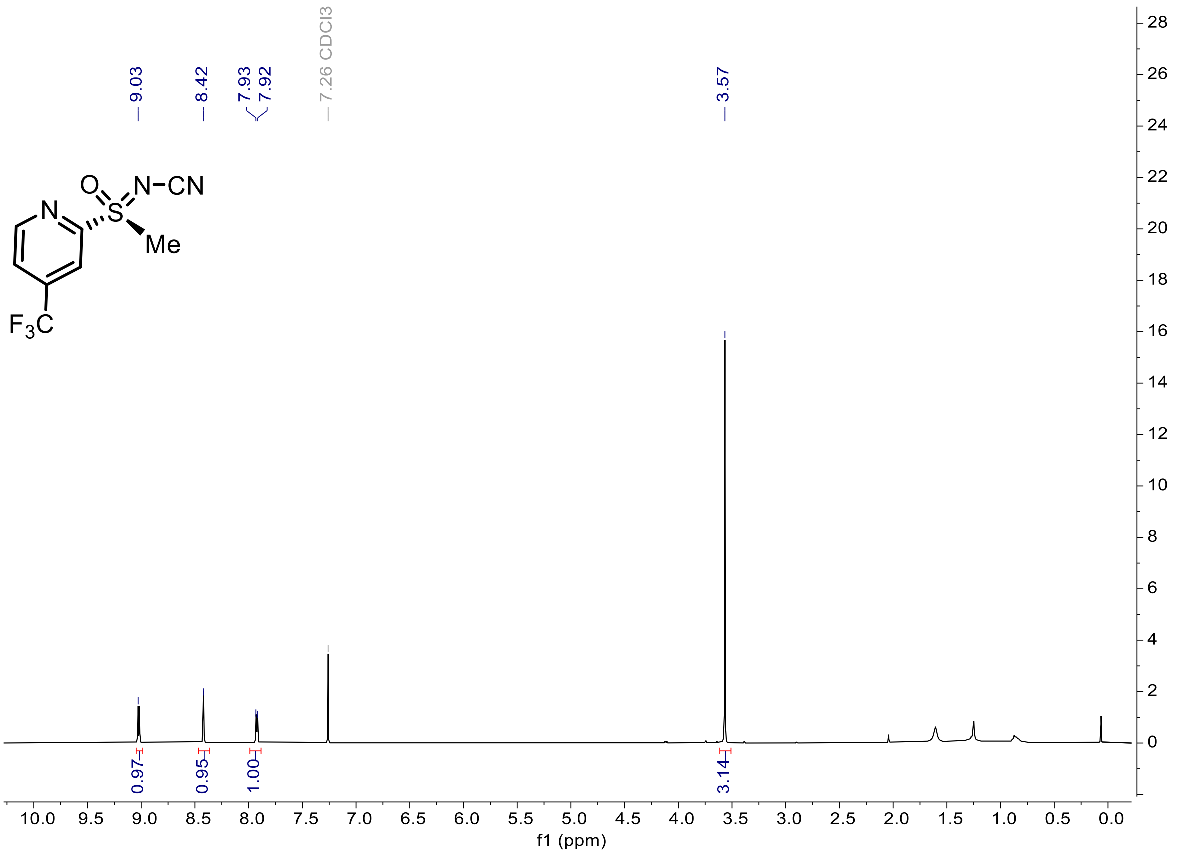

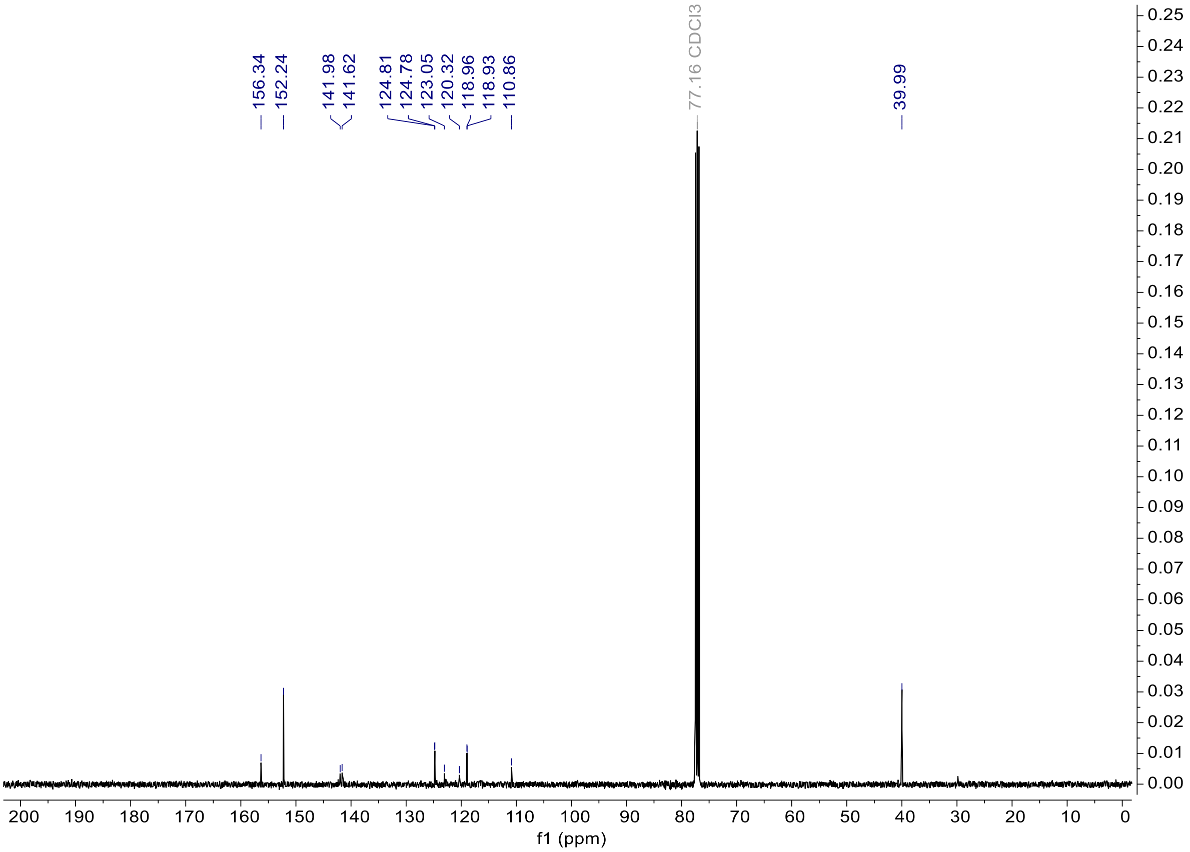

Figure S105: NMR data for **5m**:

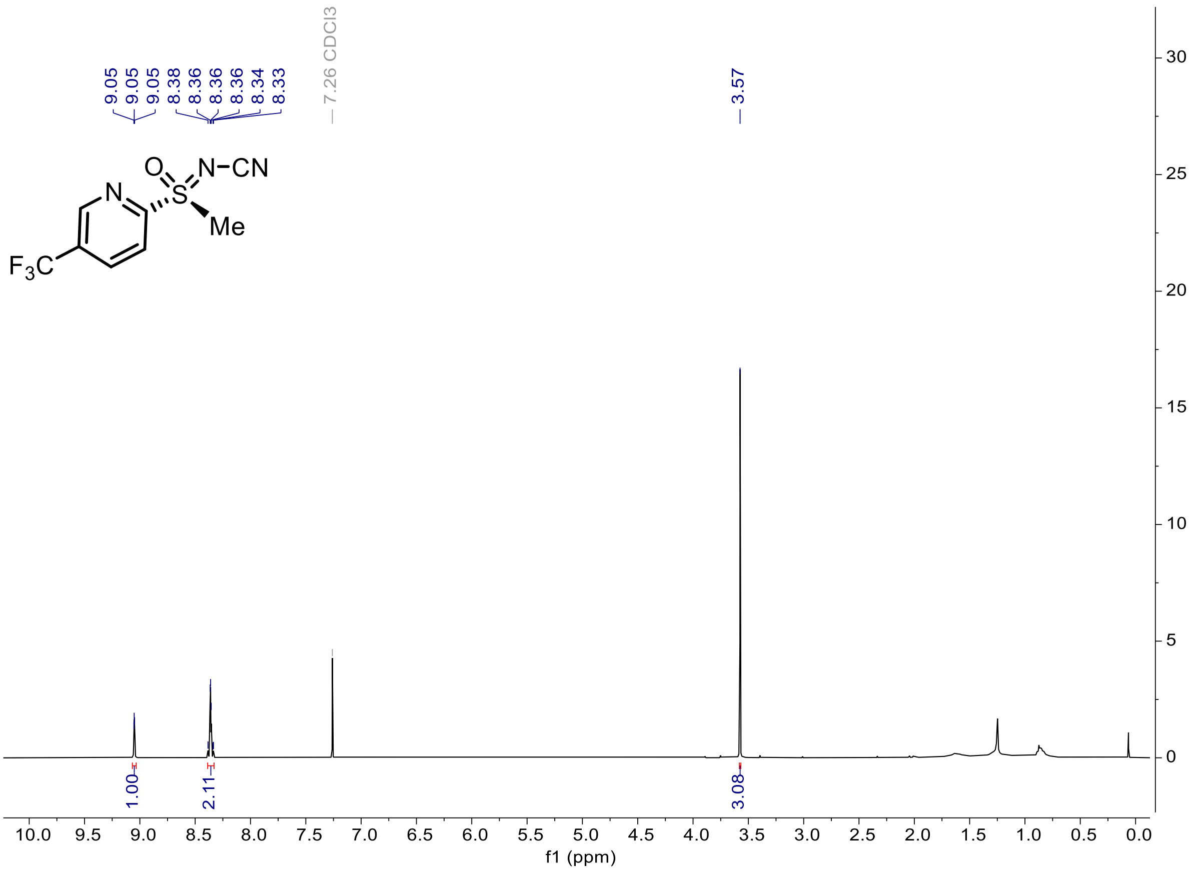

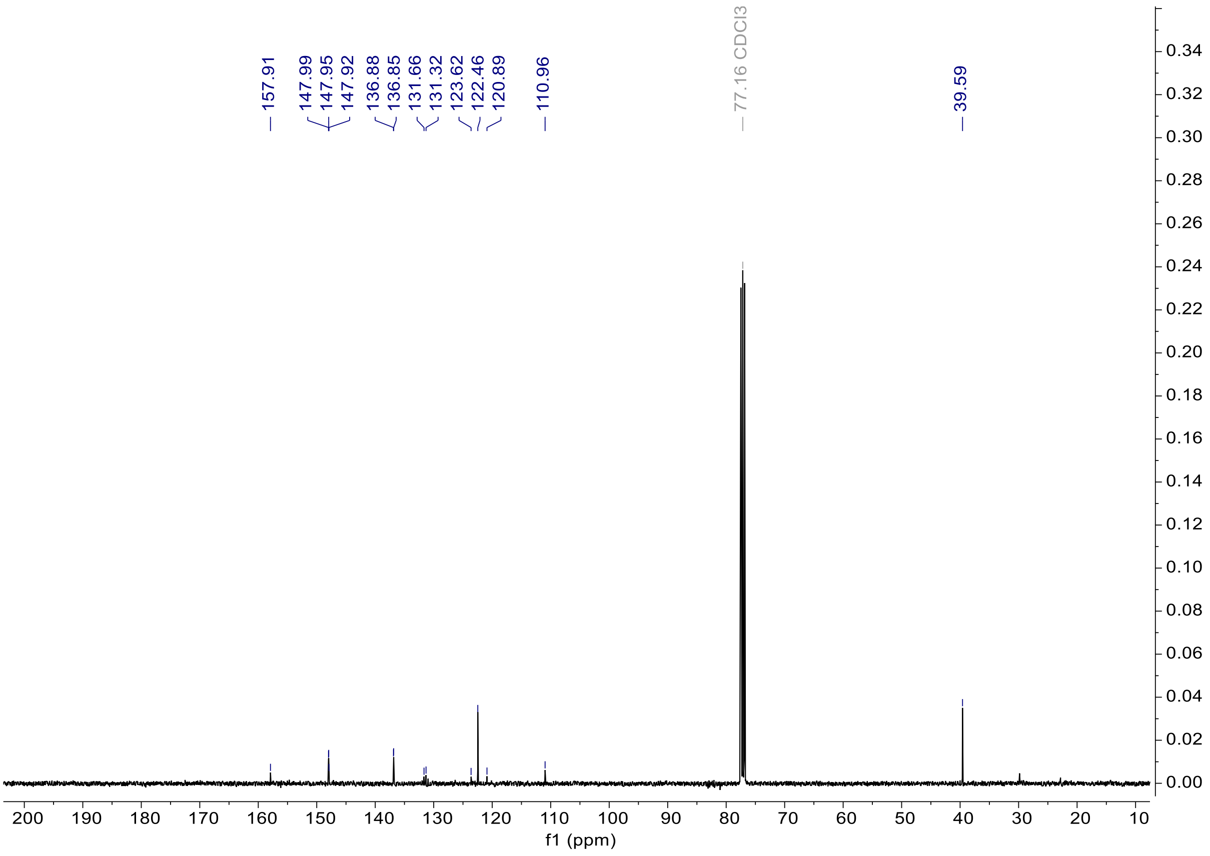

Figure S106: NMR data for **5n**

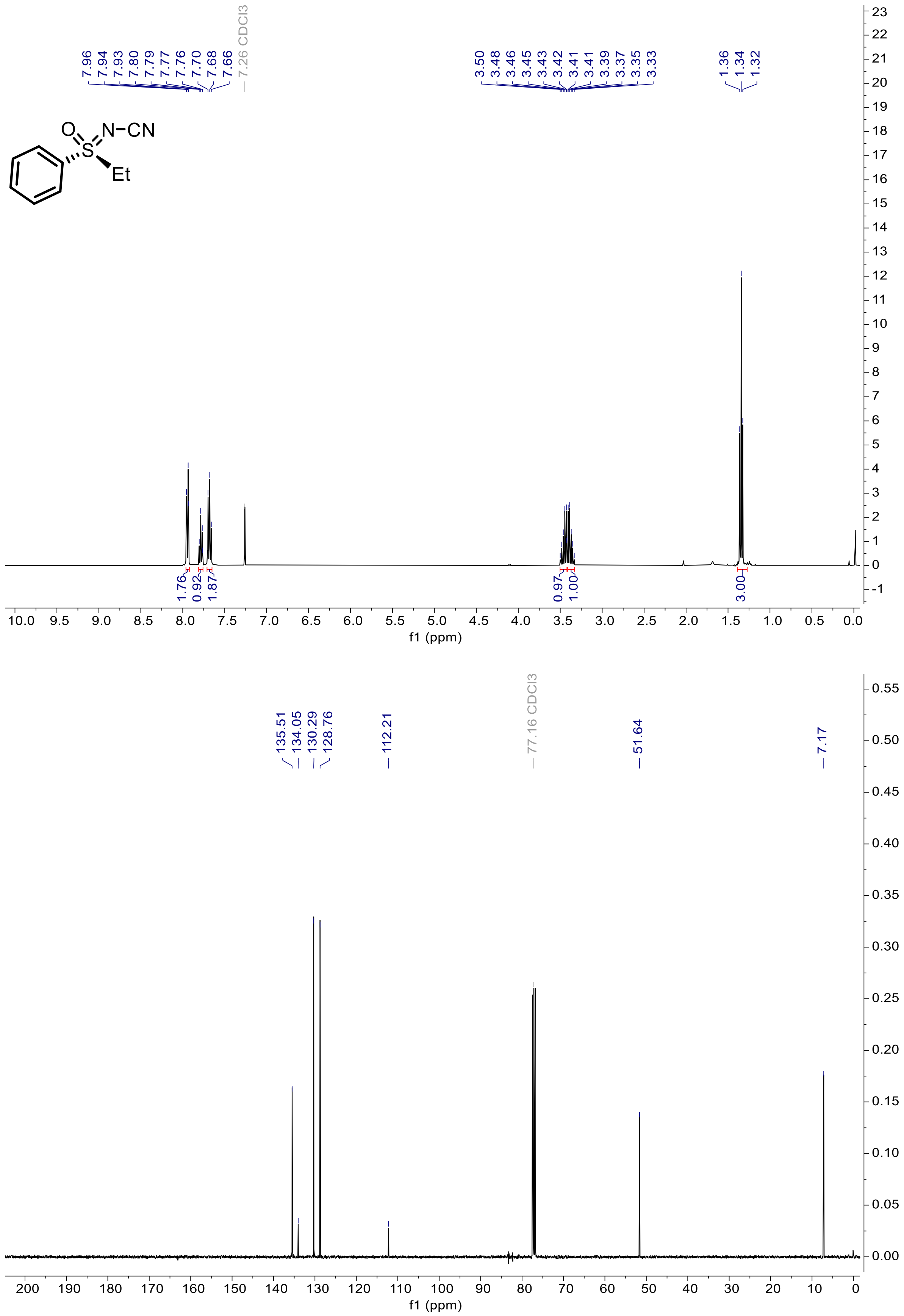

Figure S107: NMR data for **5o**

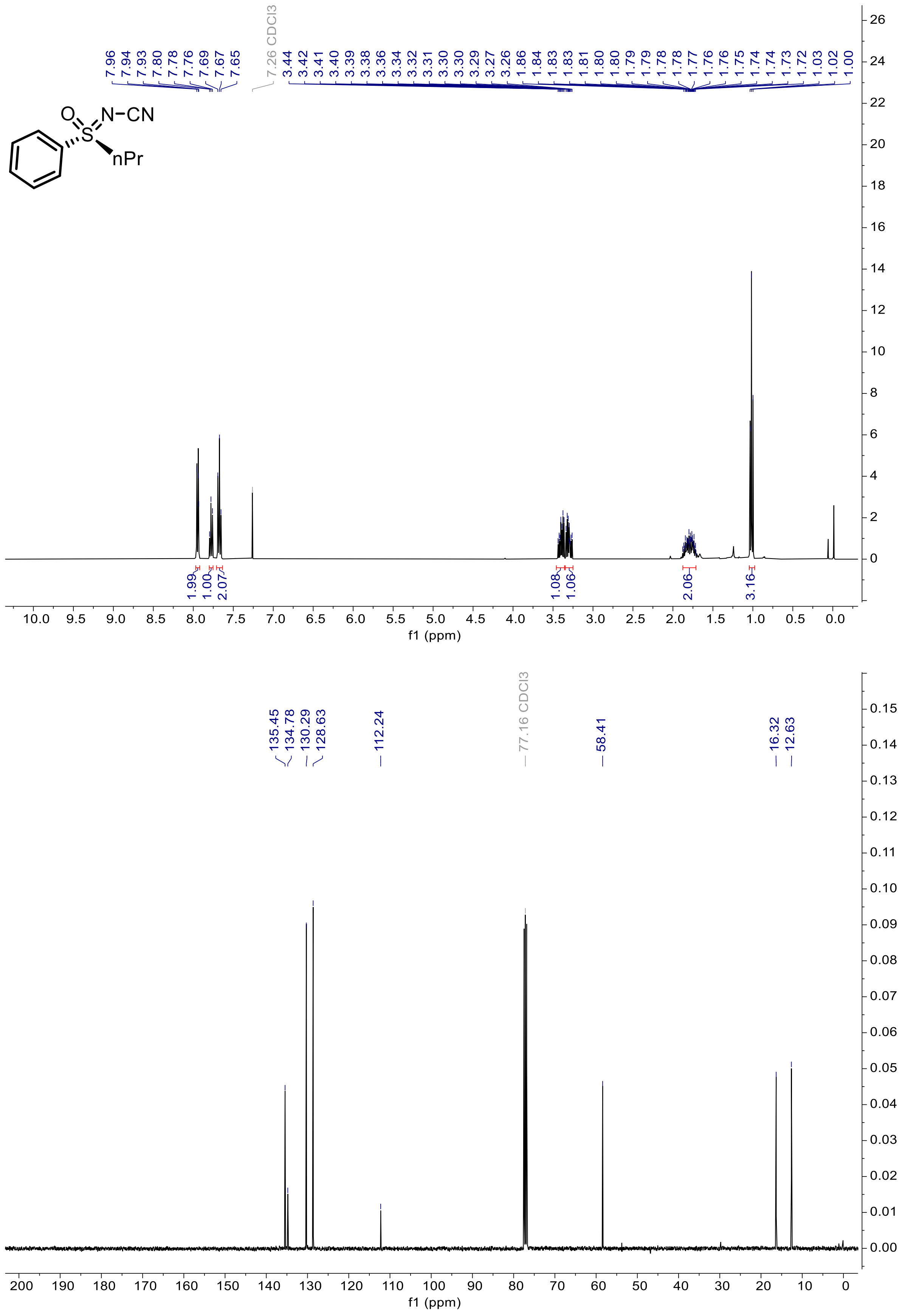

Figure S108: NMR data for **5p**

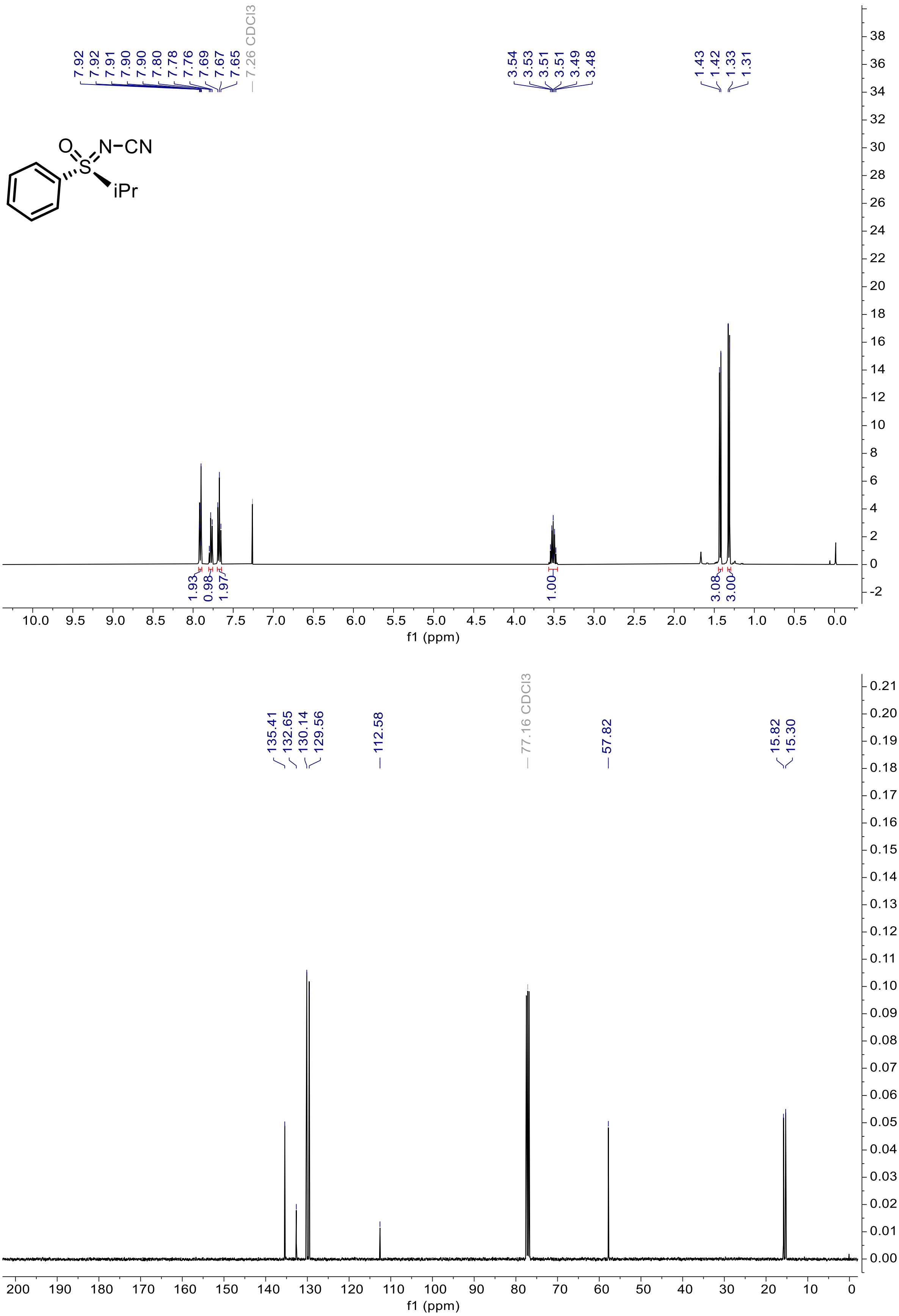

Figure S109: NMR data for **5q**

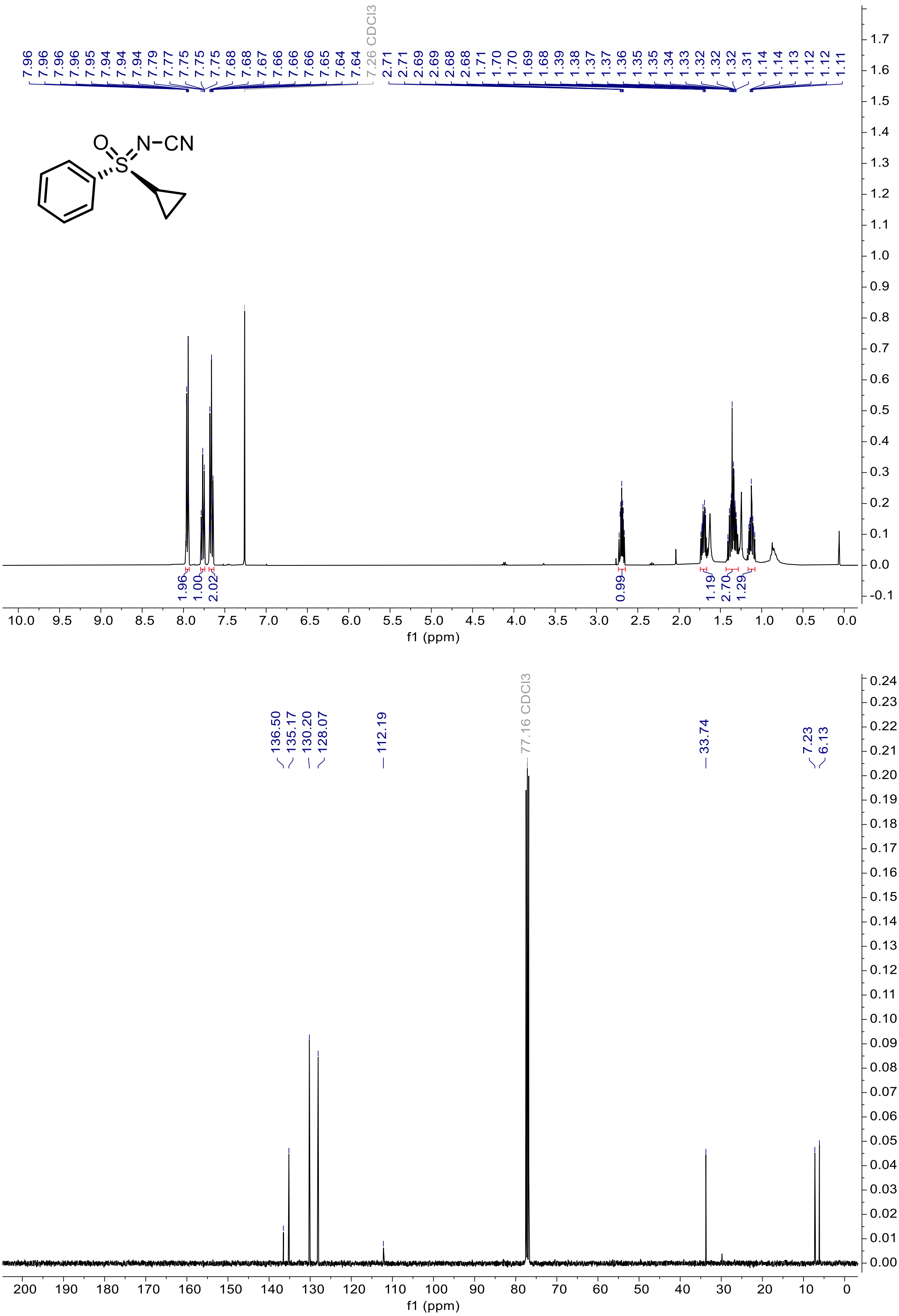

Figure S110: NMR data for **5r**

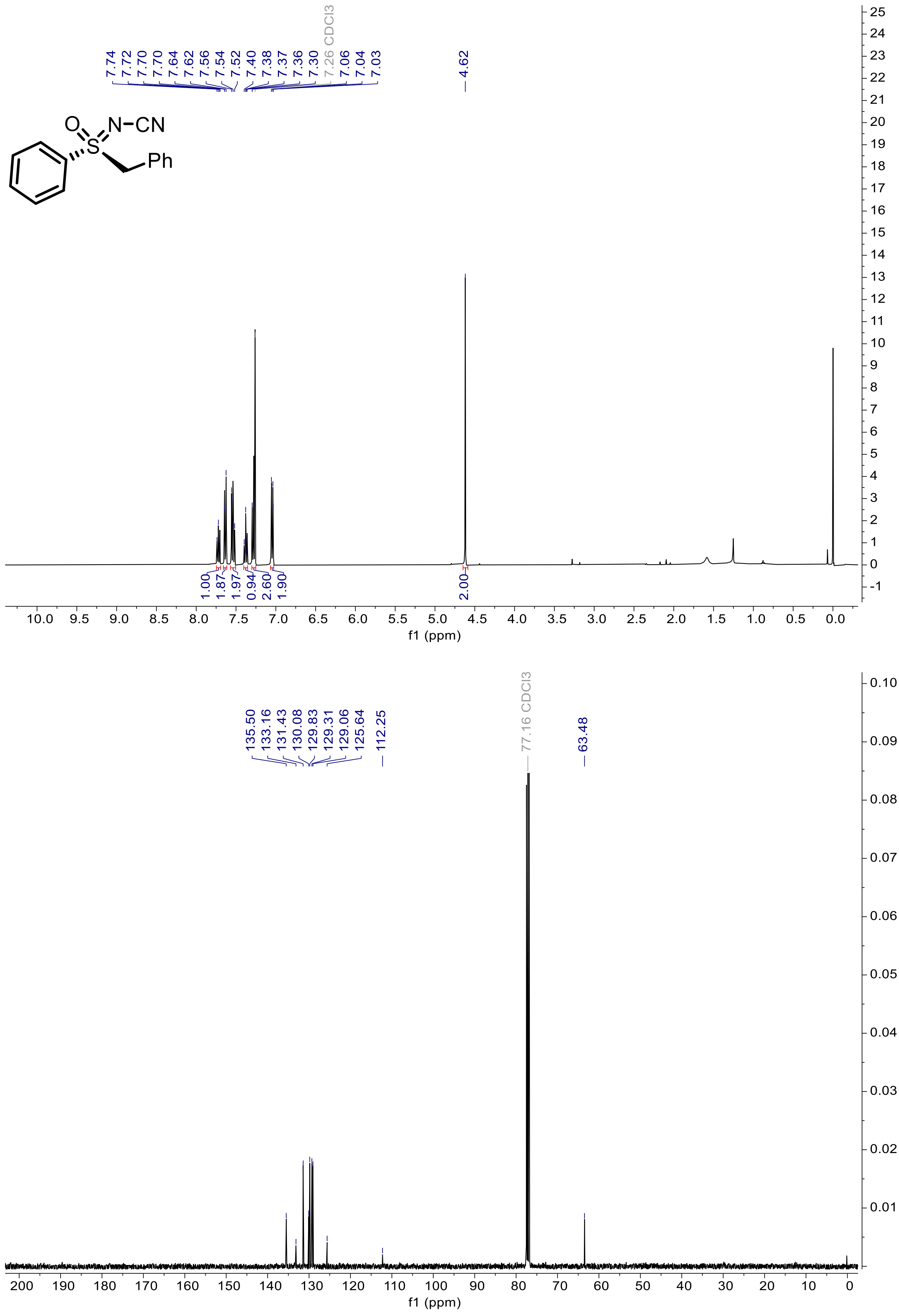

Figure S111: NMR data for **5s**

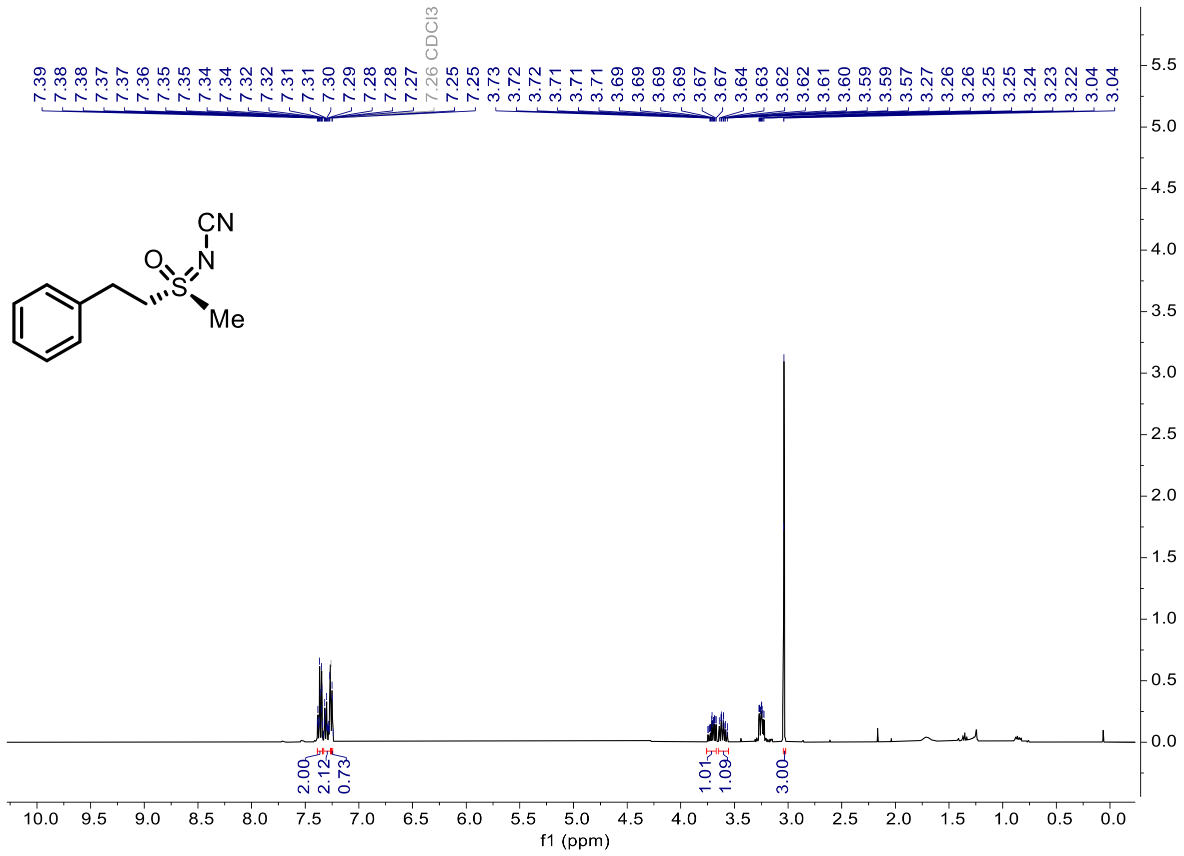

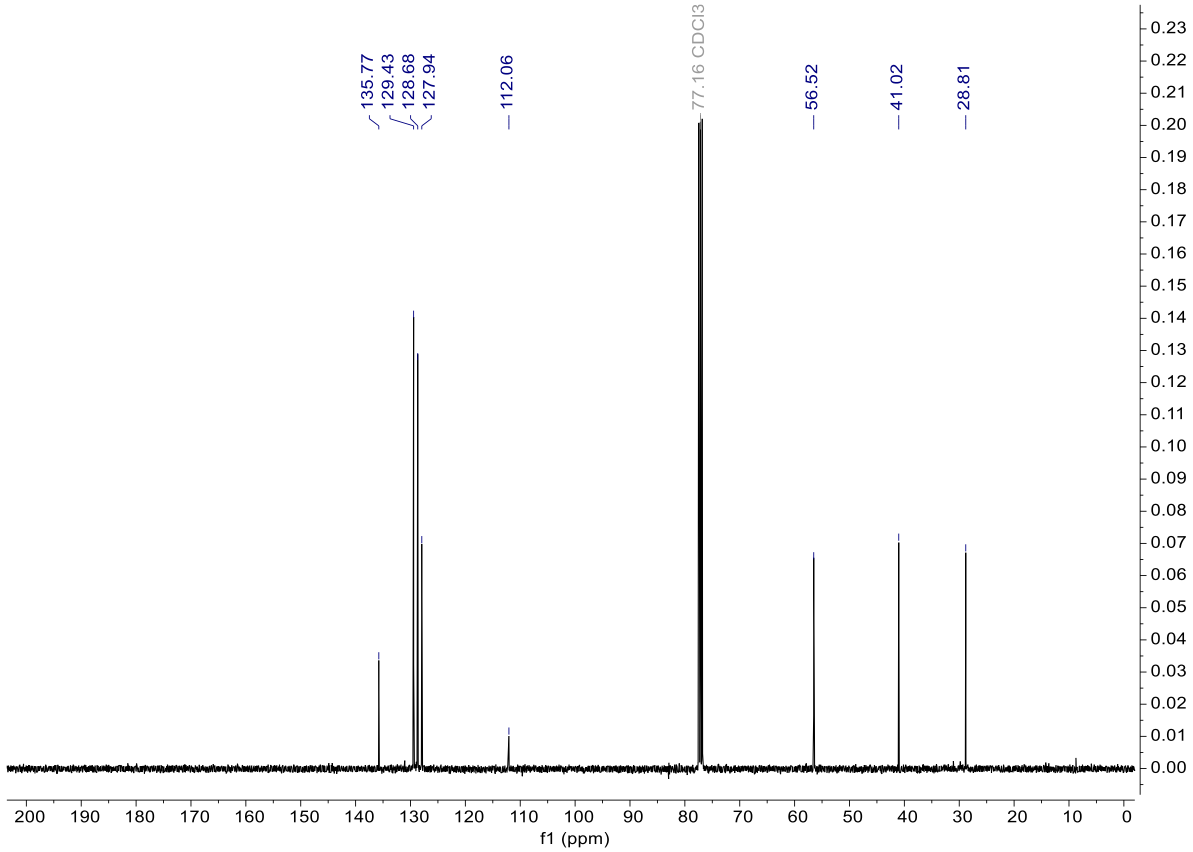

Figure S112: NMR data for **5t**:

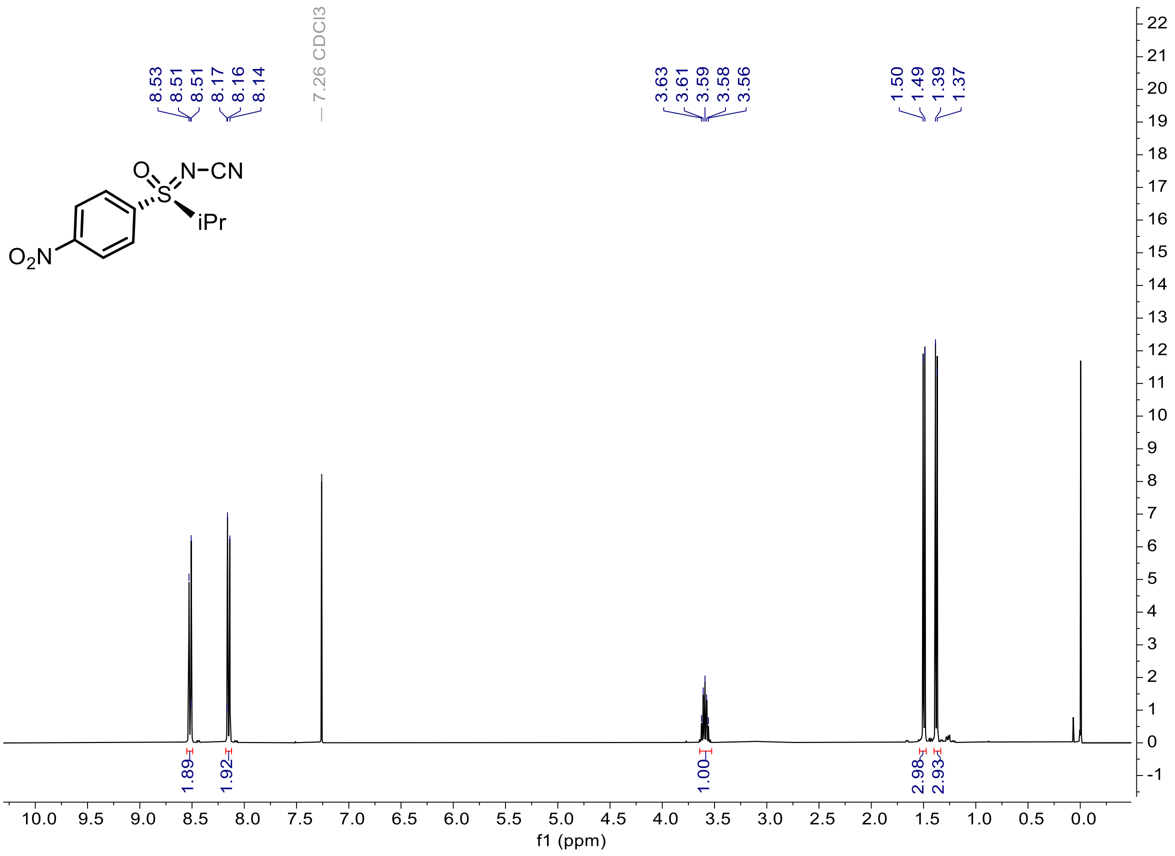

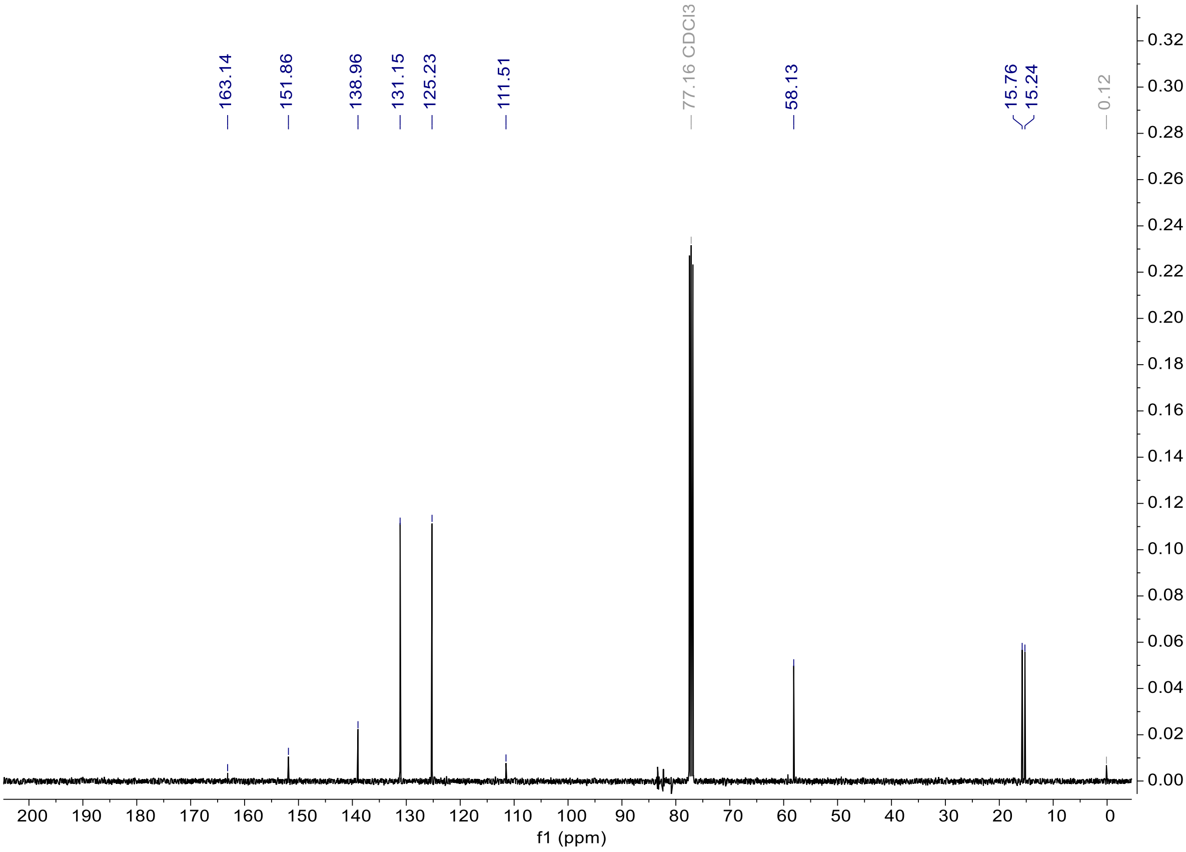

Figure S113: NMR data for **5u**:

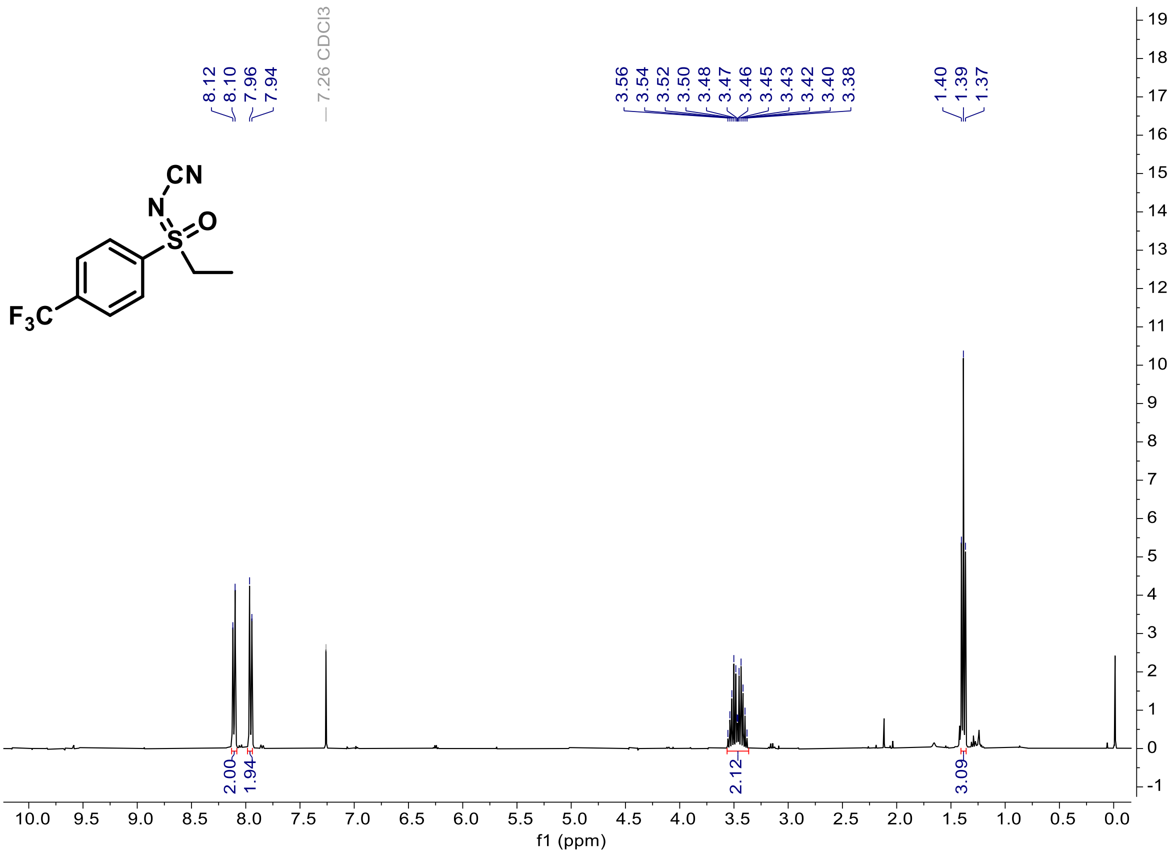

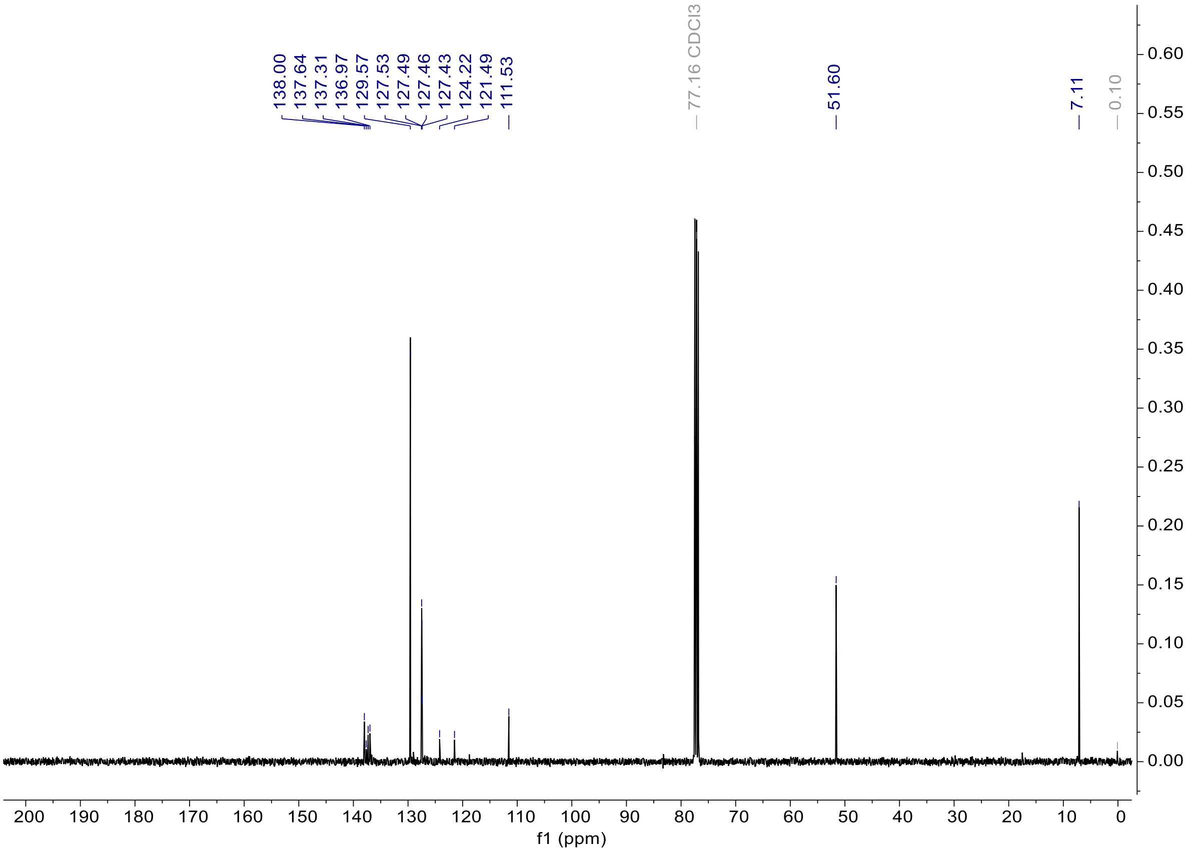

Figure S114: NMR data for **9e**

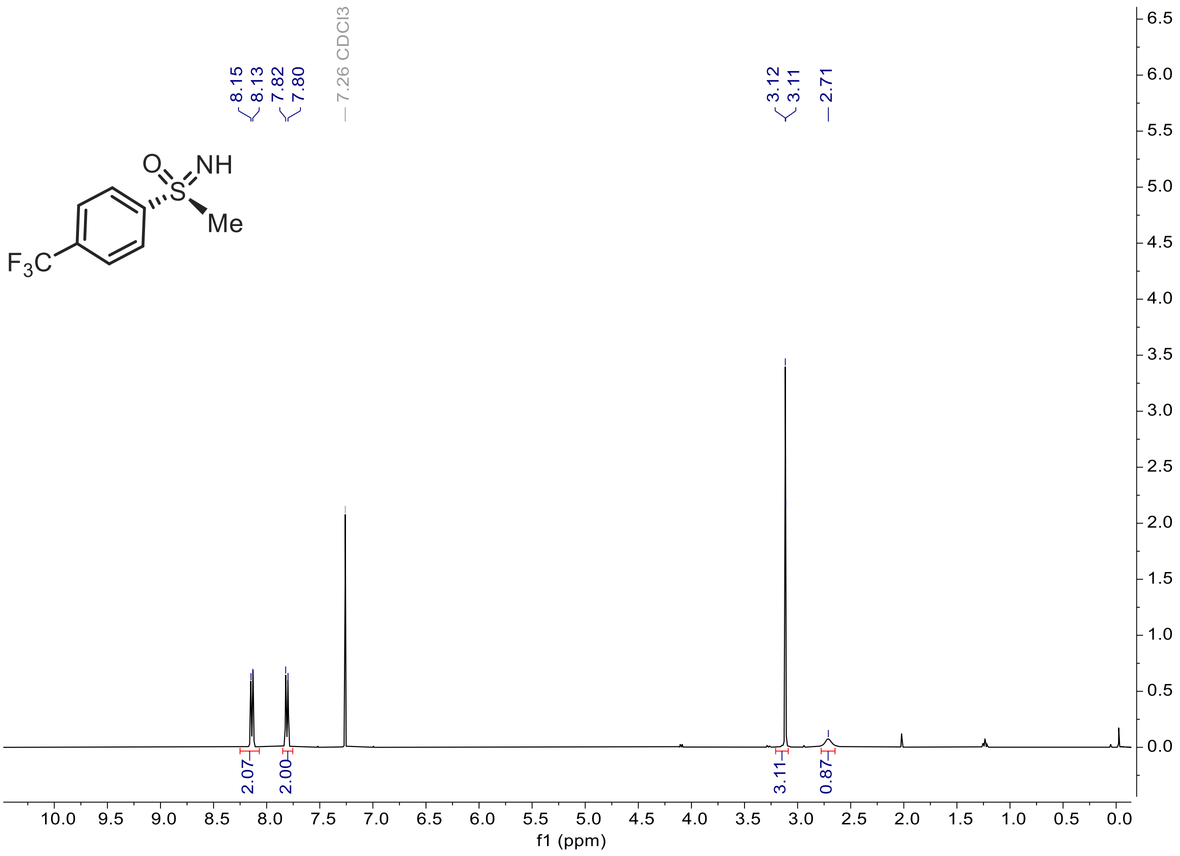

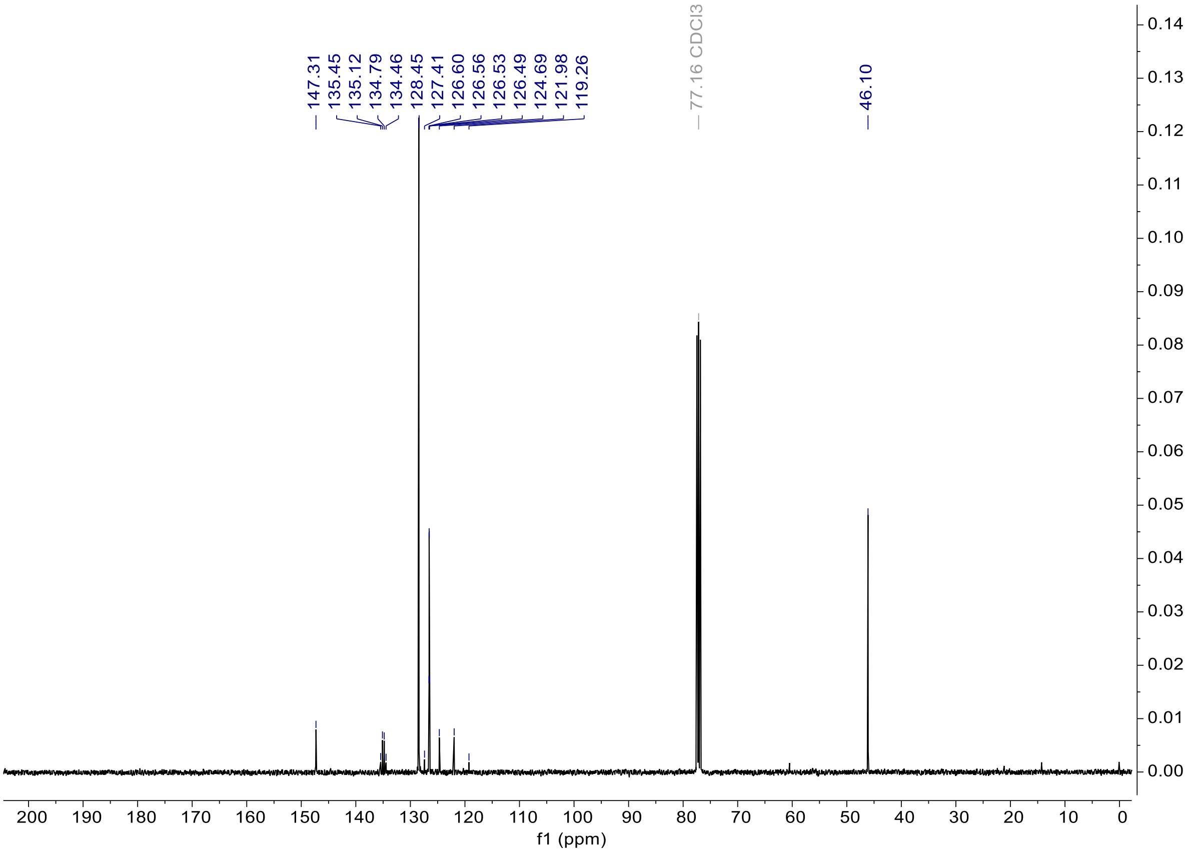

Figure S115: NMR data for **6a-*i*Pr**

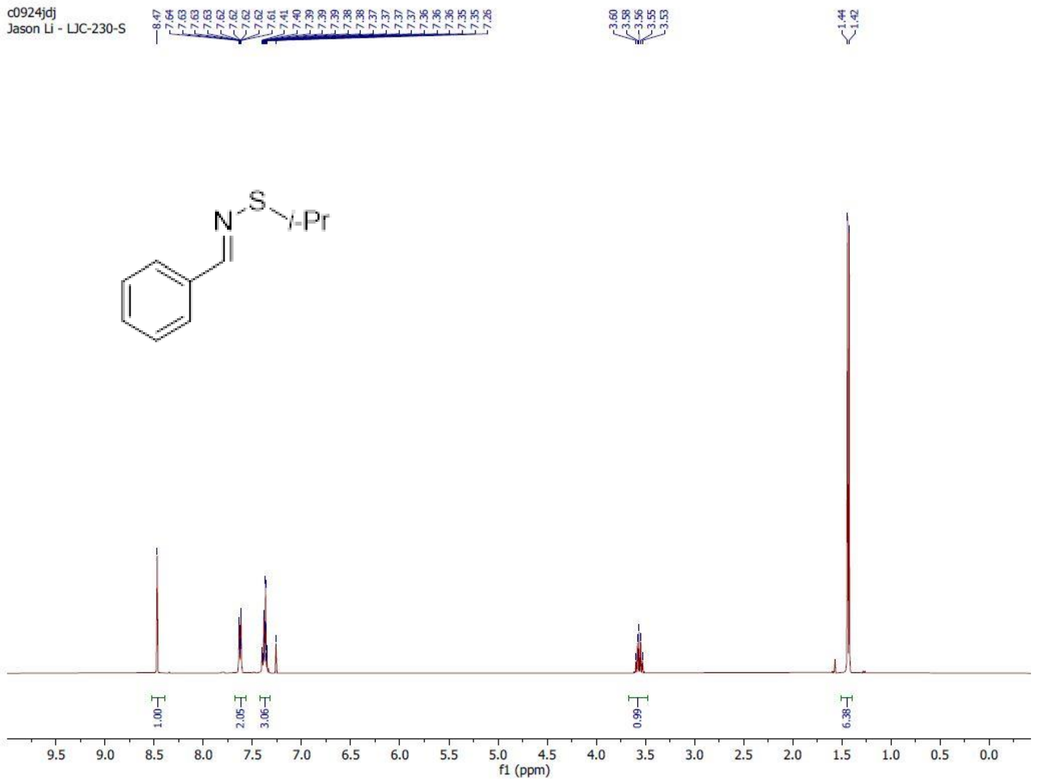

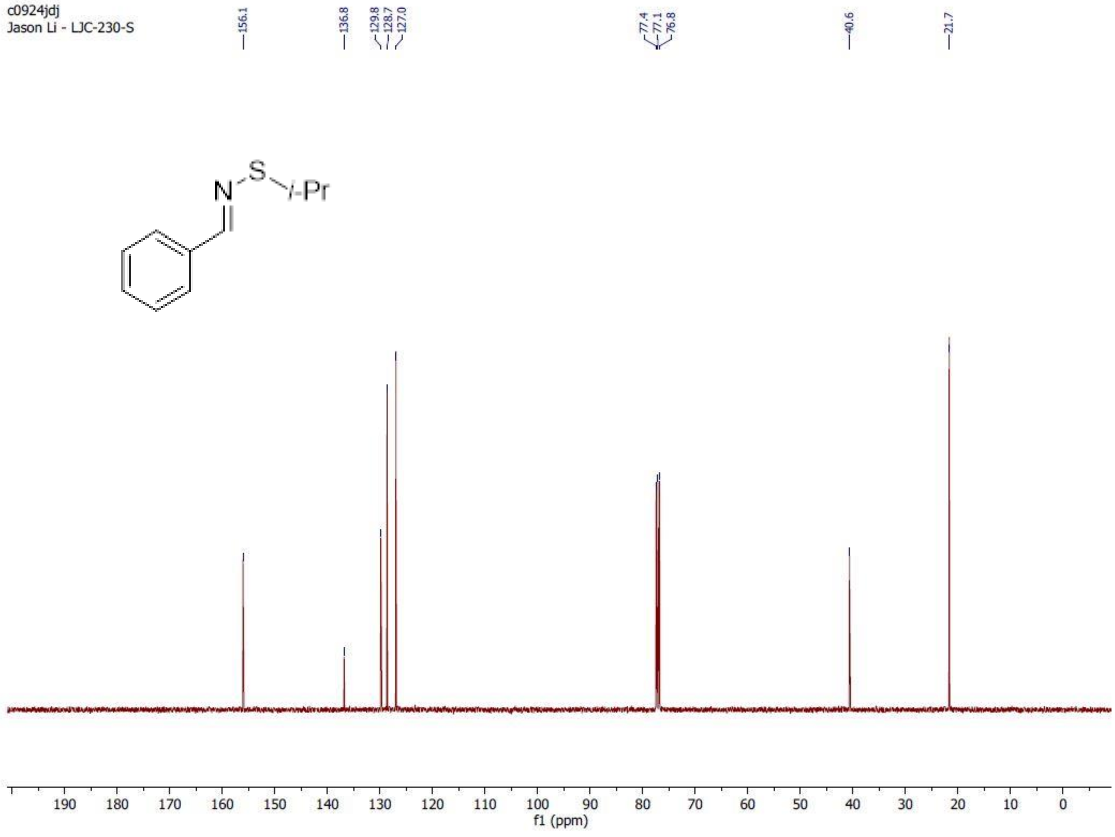

Figure S116: NMR data for **6b-*i*Pr**

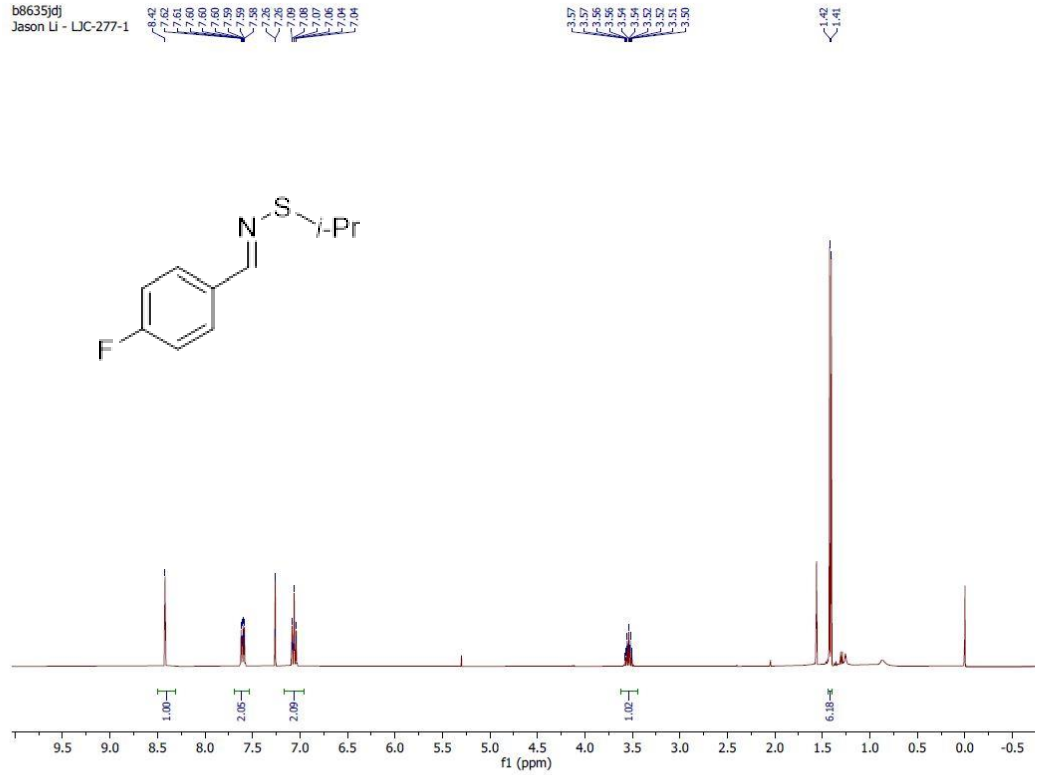

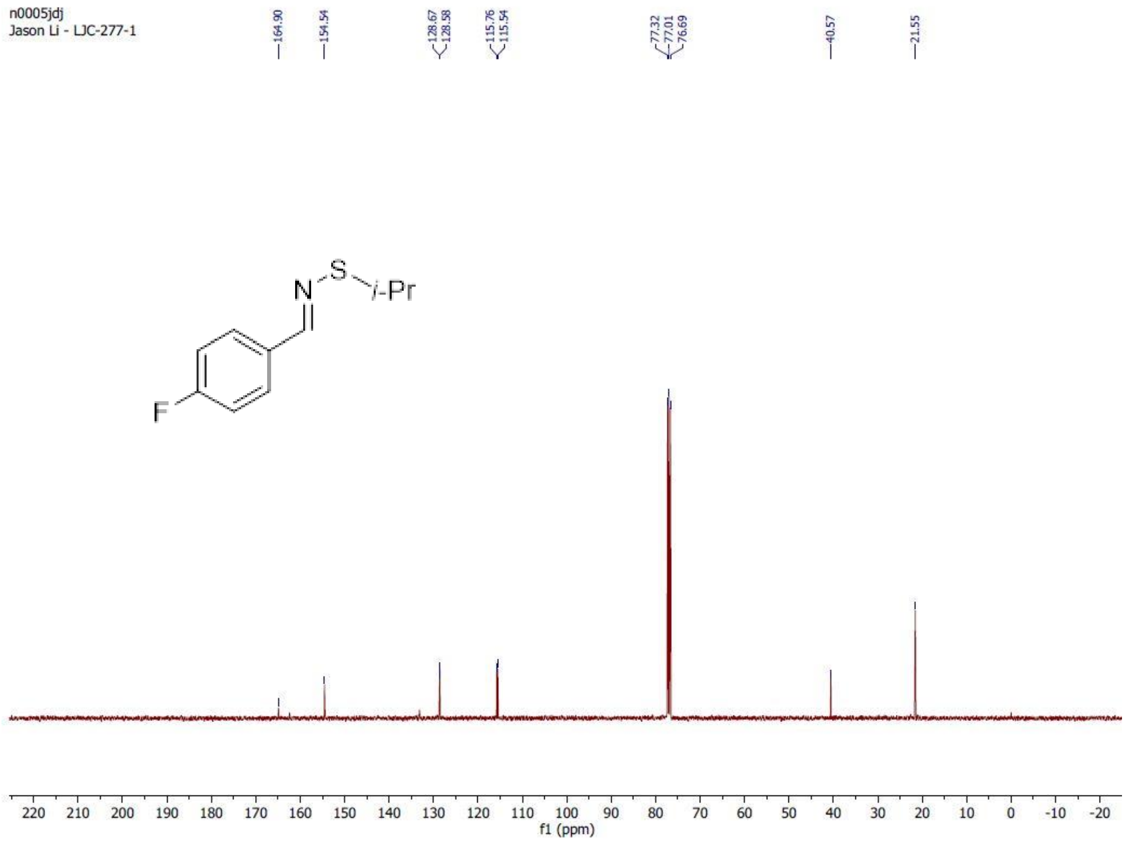

Figure S117: NMR data for **6c-*i*Pr**

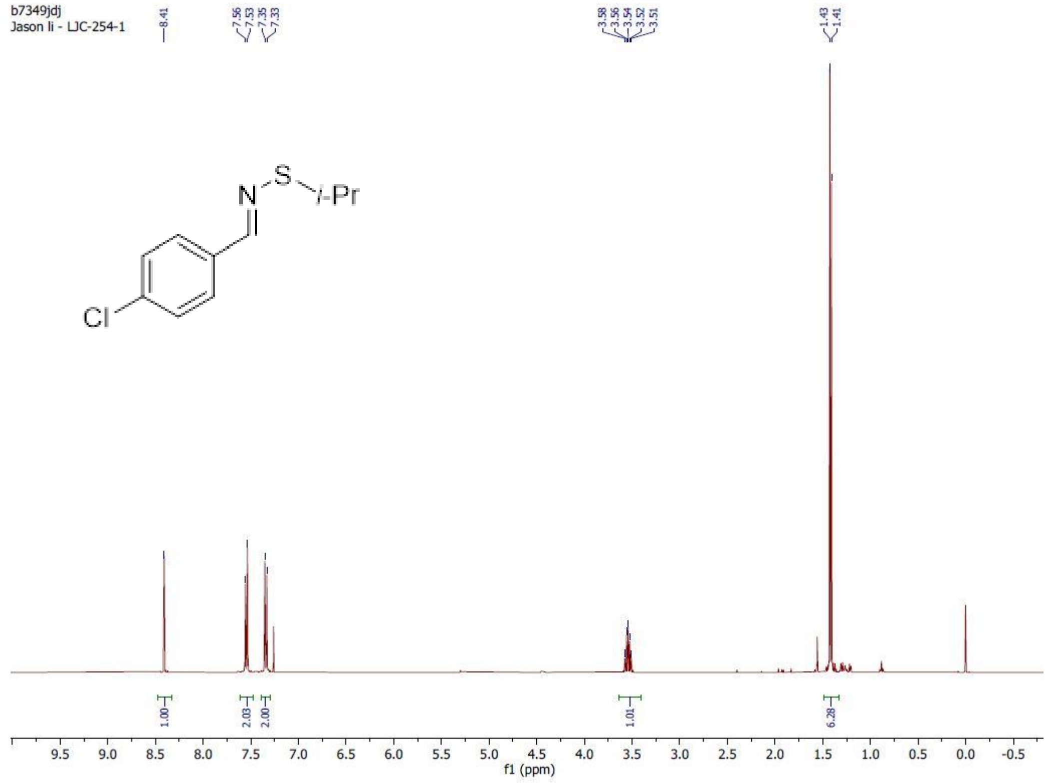

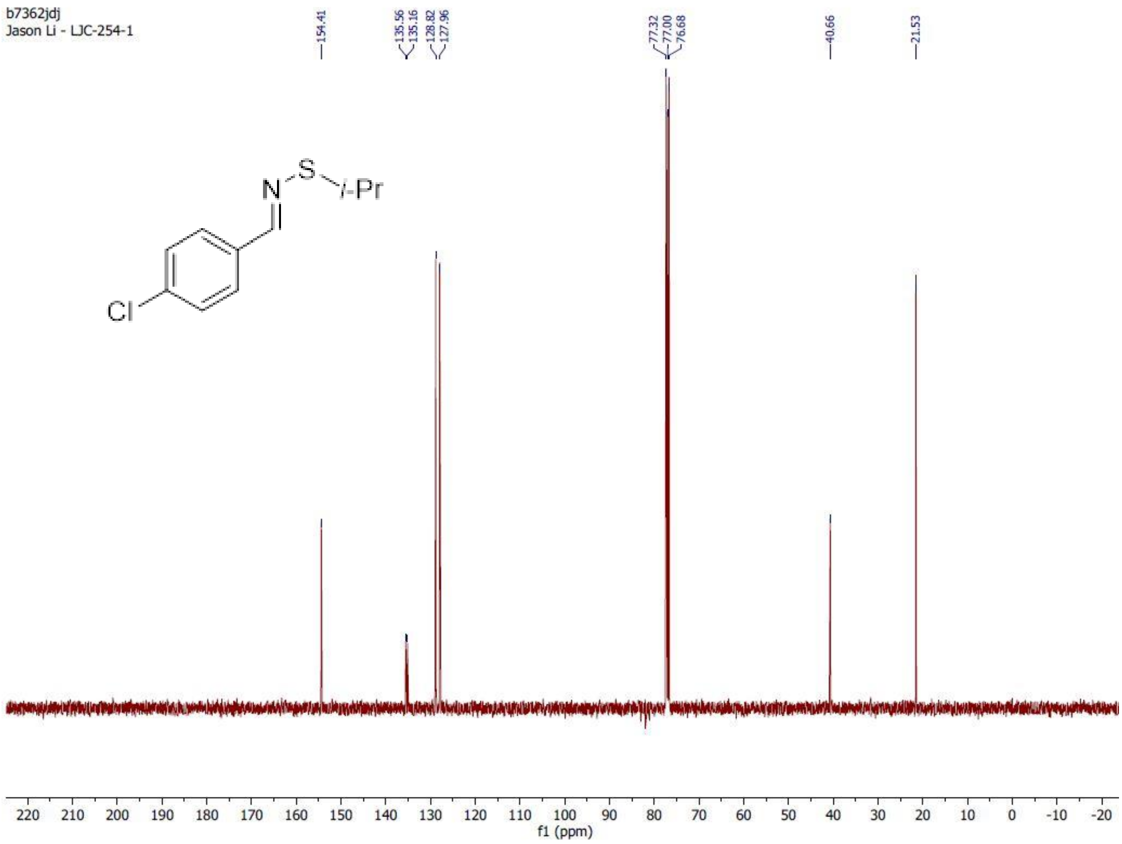

Figure S118: NMR data for **6d-*i*Pr**

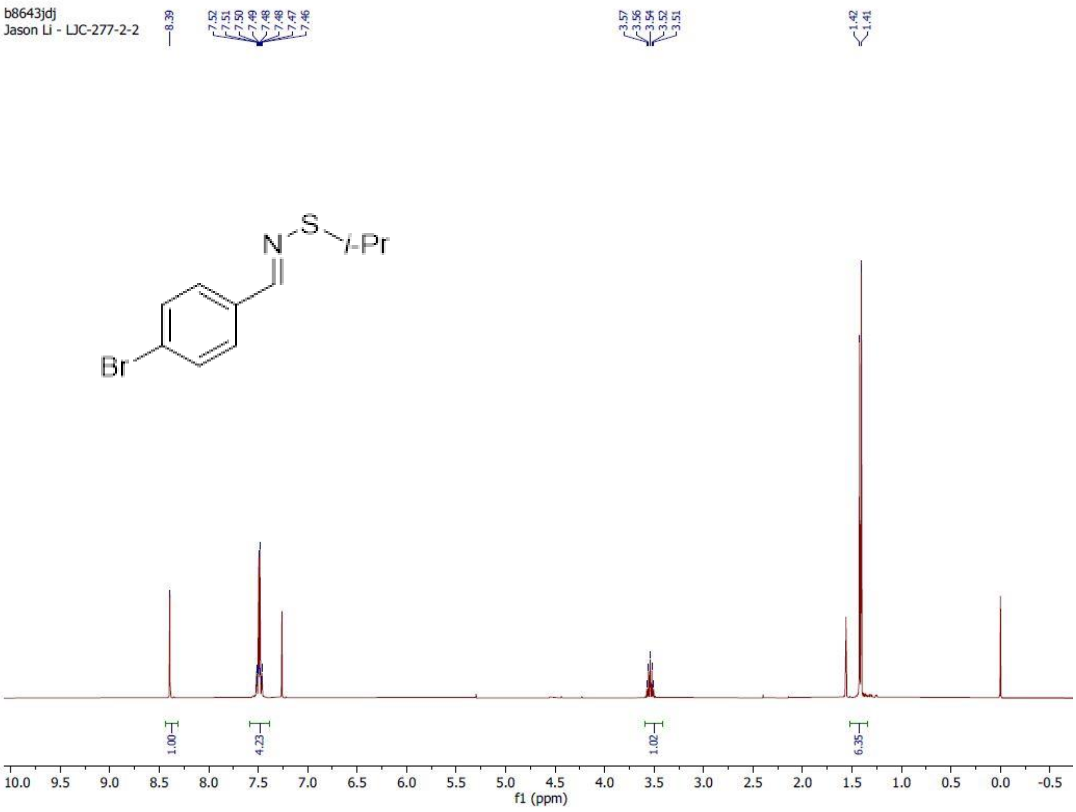

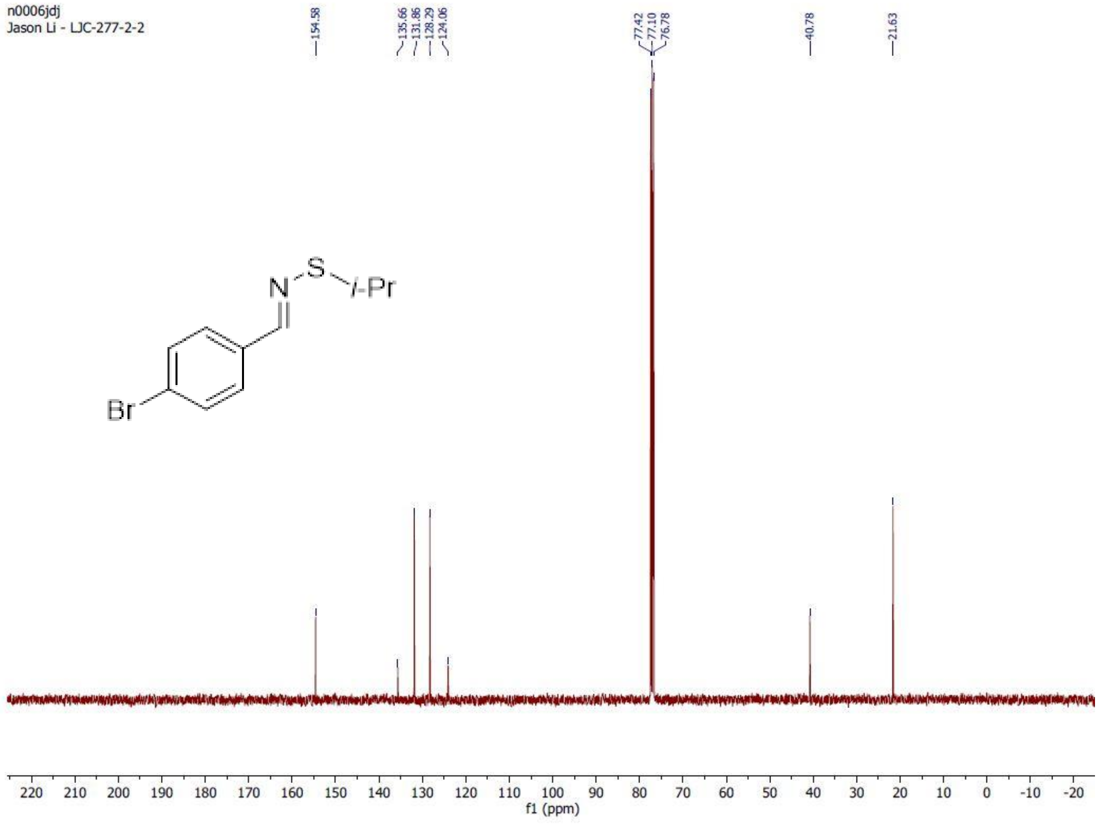

Figure S119: NMR data for **6e-*i*Pr**

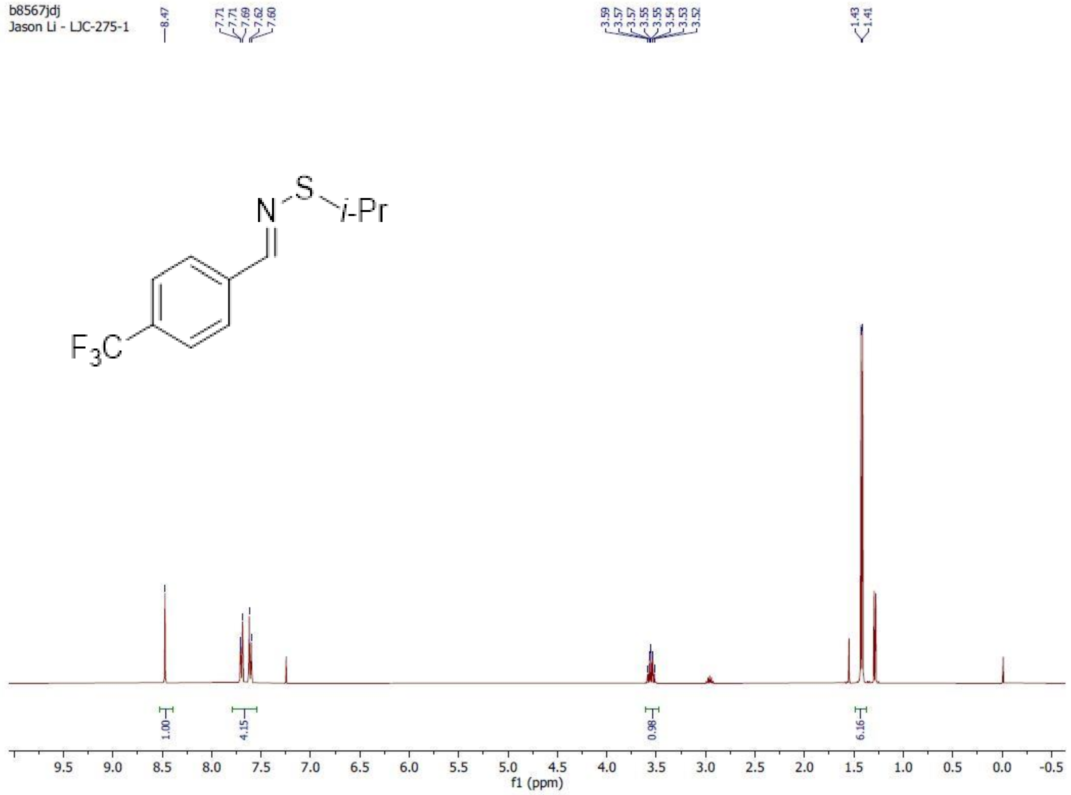

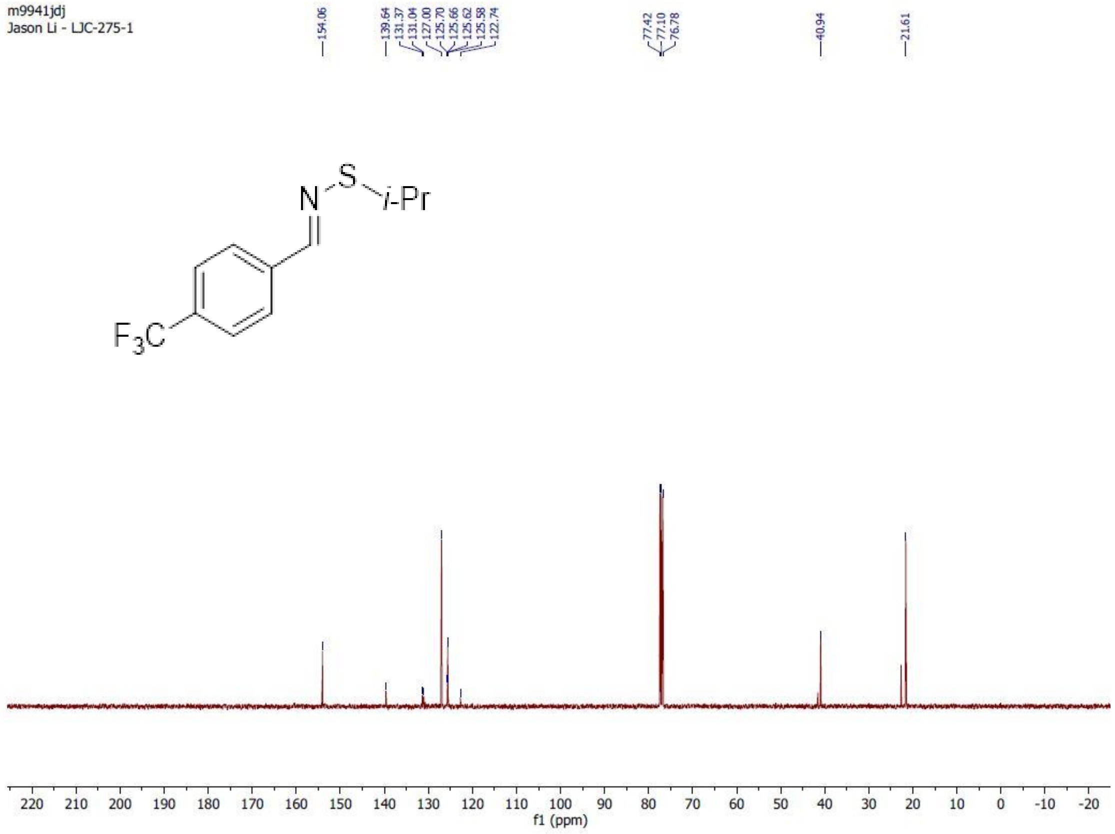

Figure S120: NMR data for **6f-*i*Pr**

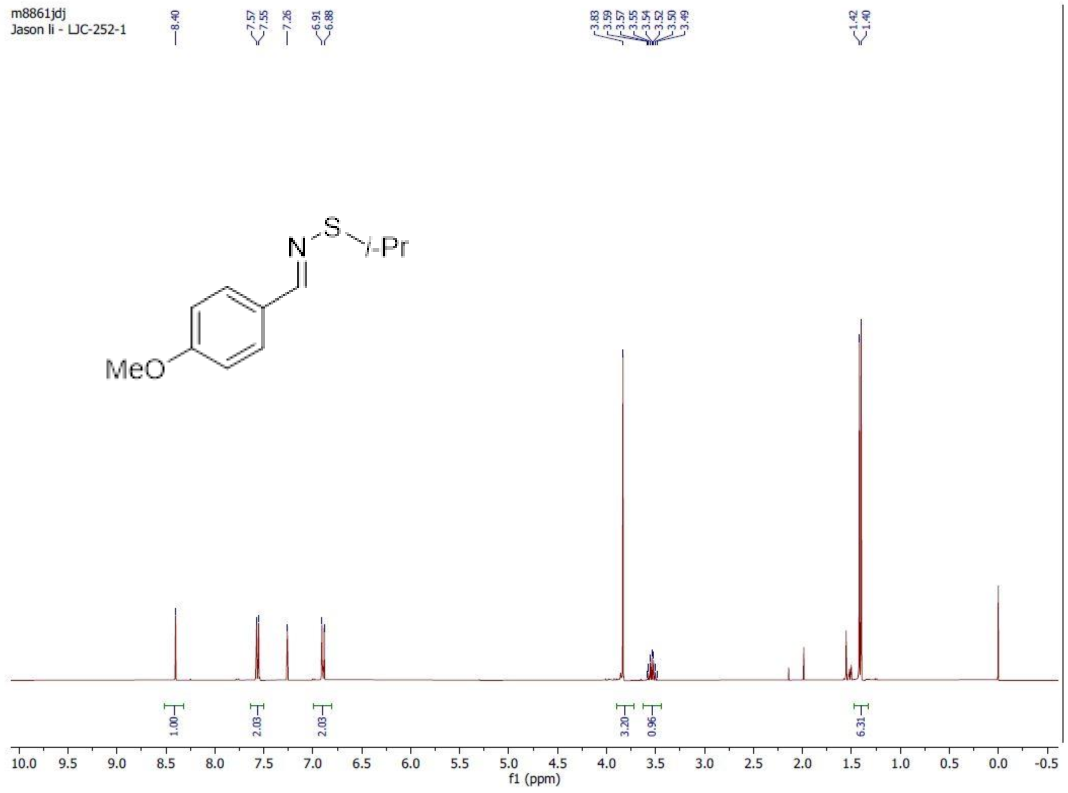

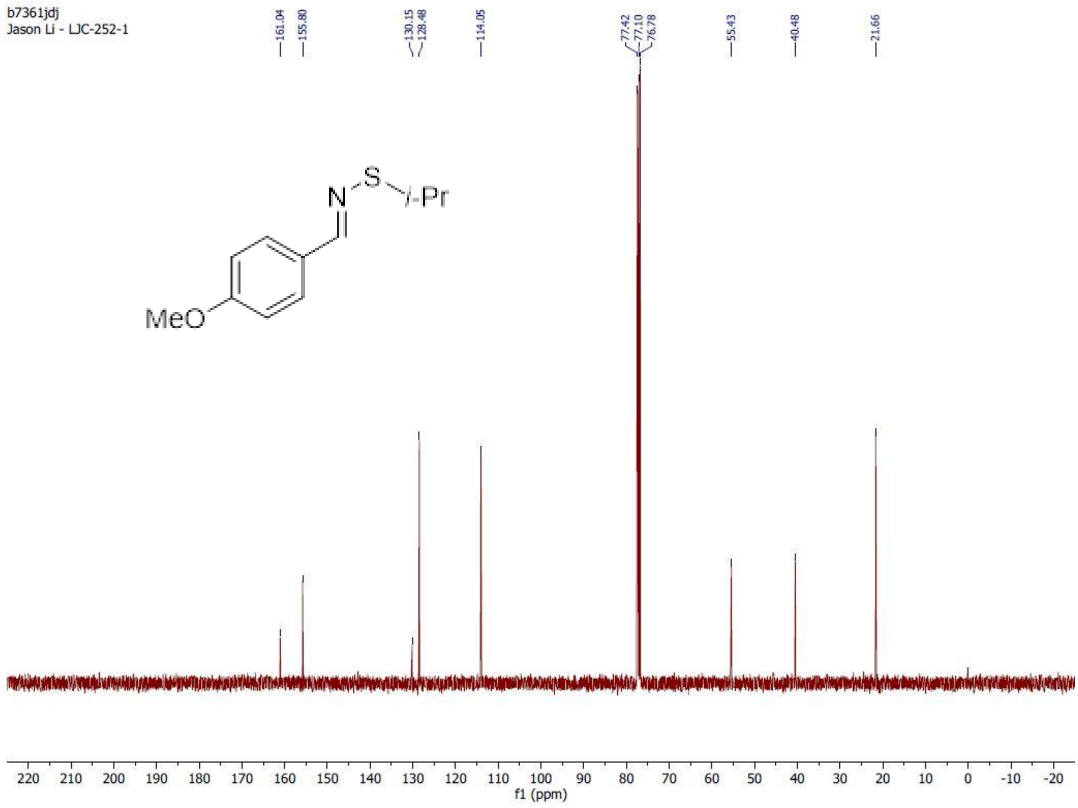

Figure S121: NMR data for **6g-*i*Pr**

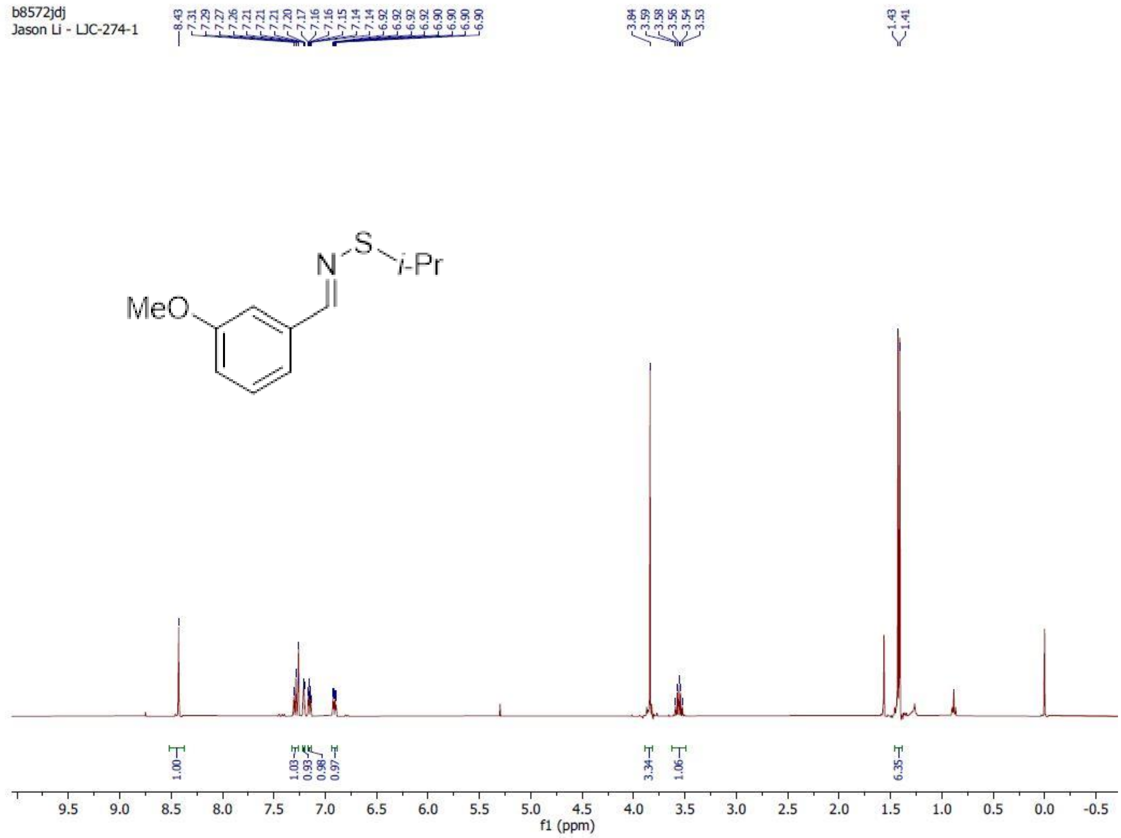

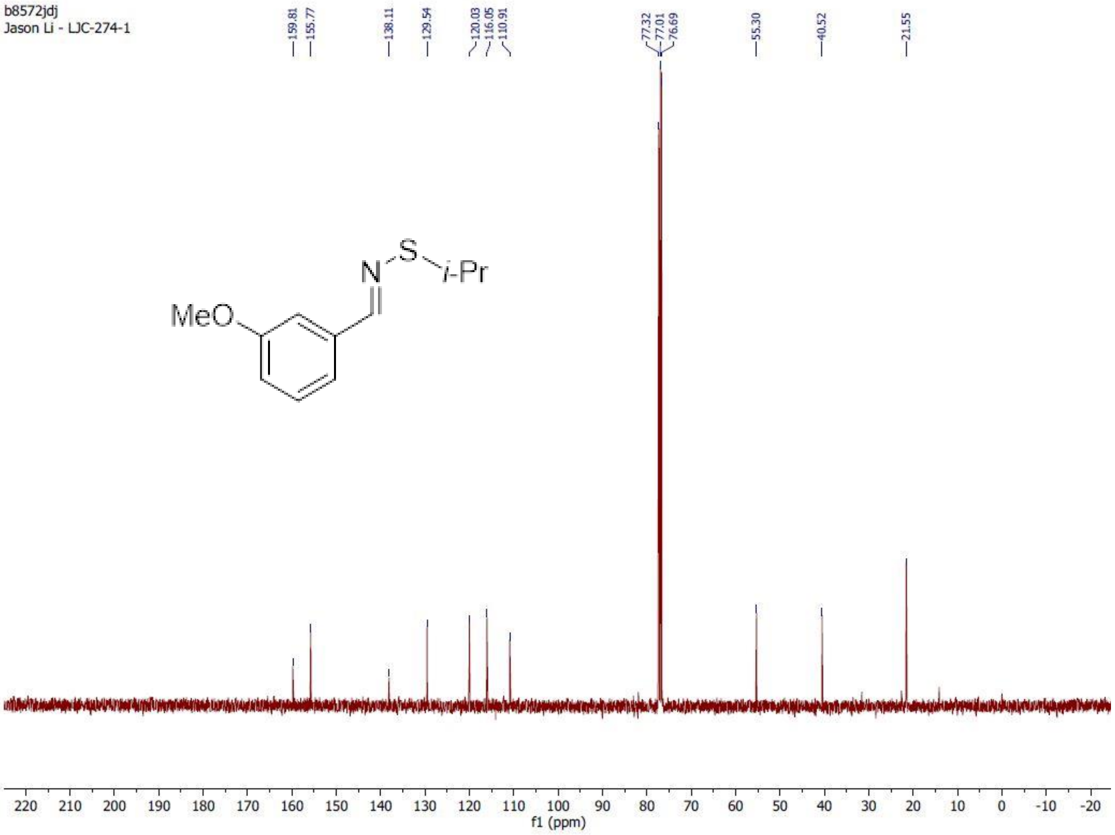

Figure S122: NMR data for **6h-*i*Pr**

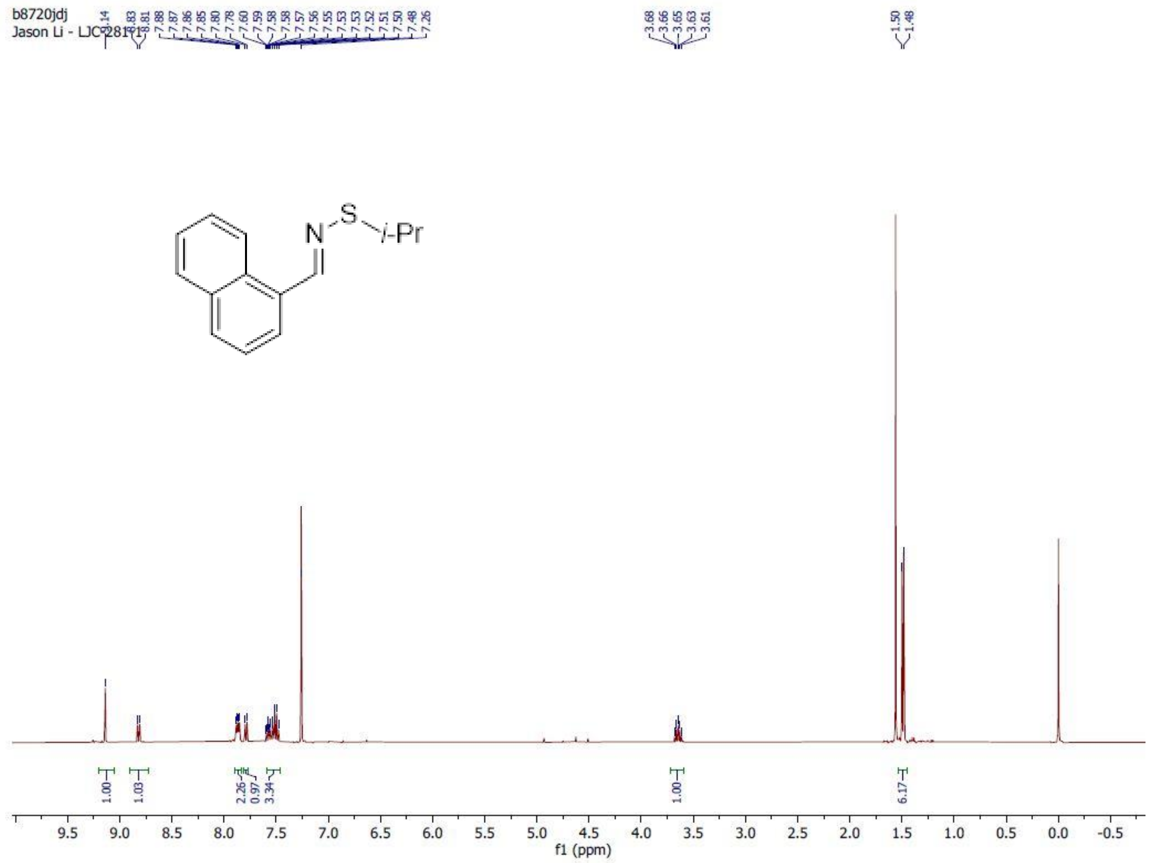

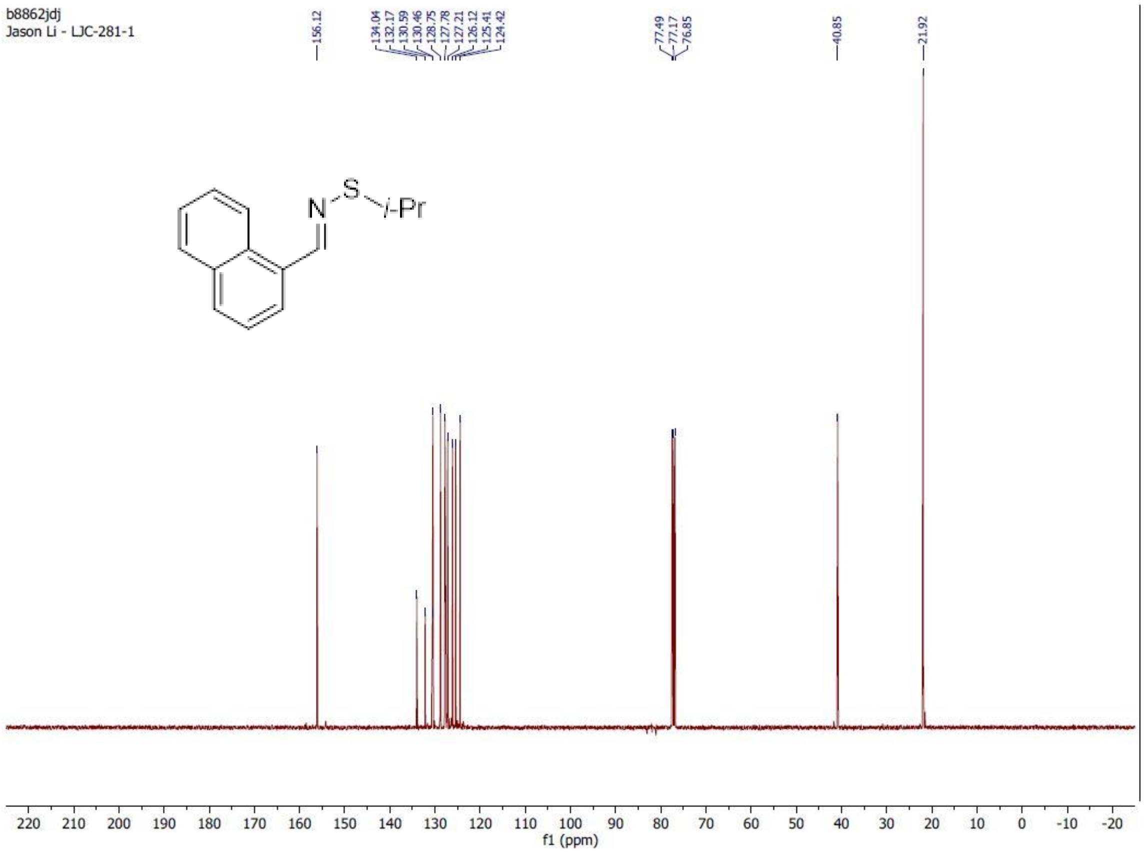

Figure S123: NMR data for **6i-*i*Pr**

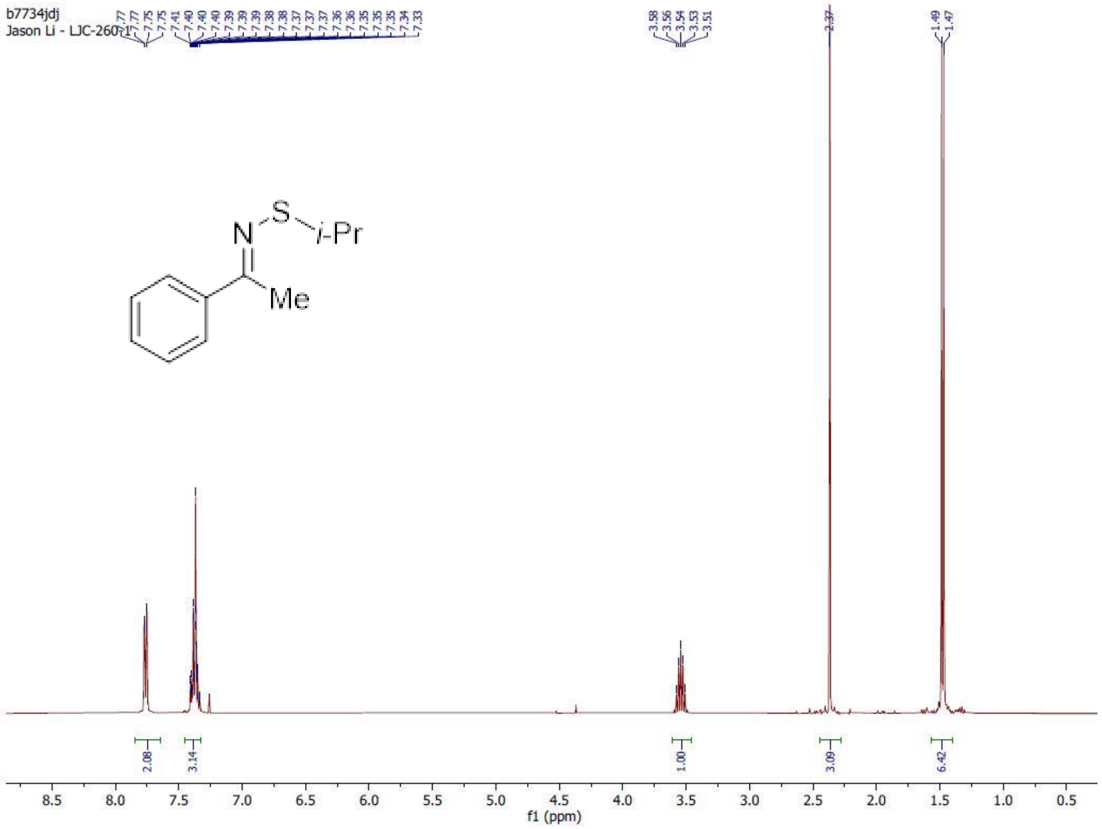

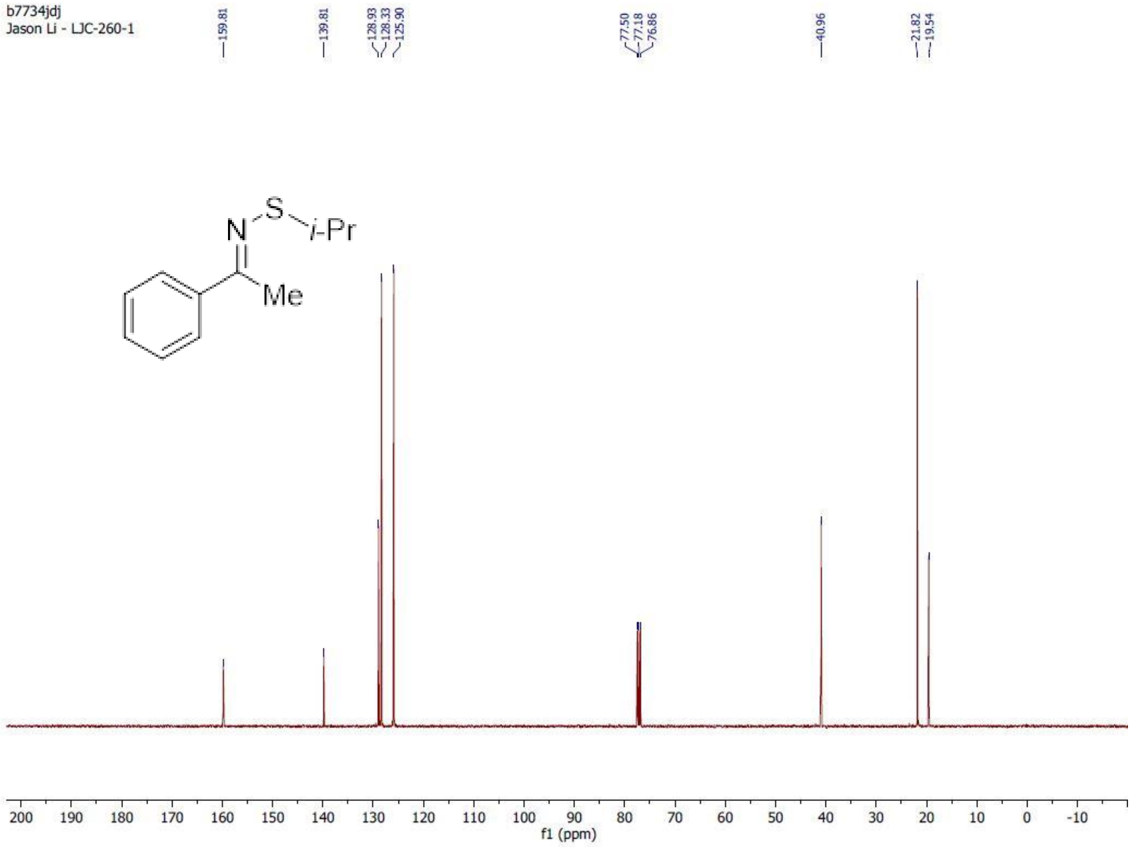

Figure S124: NMR data for **6j-*i*Pr**

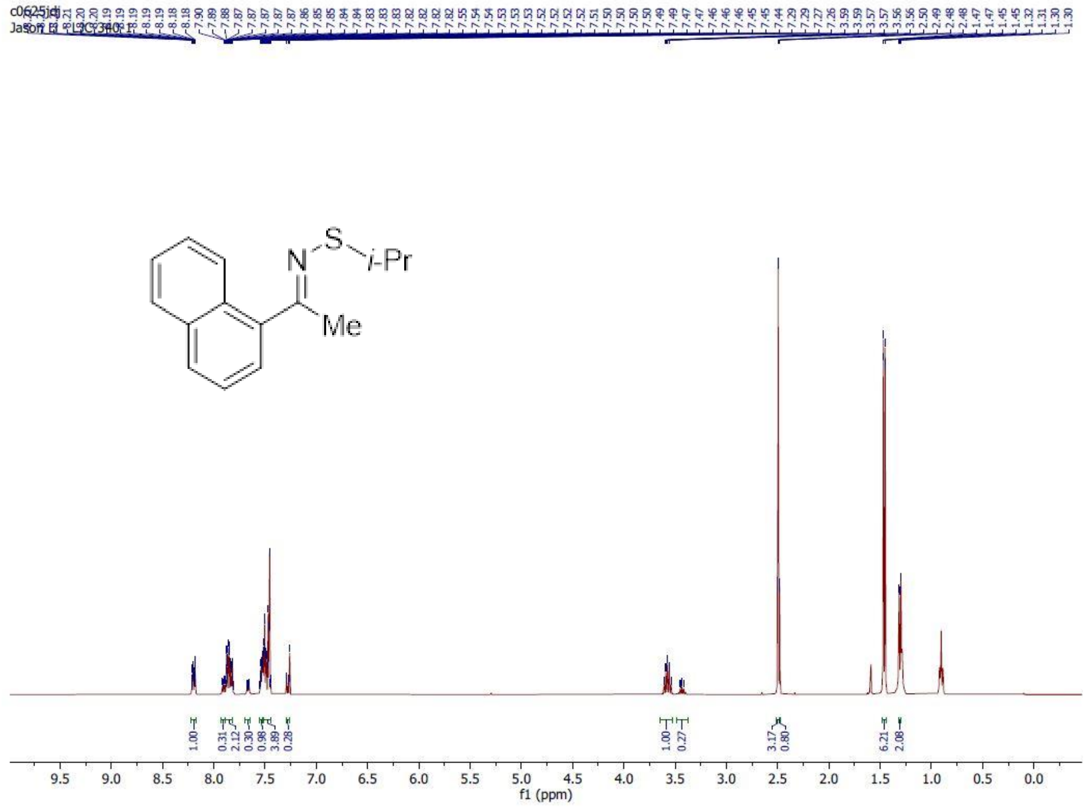

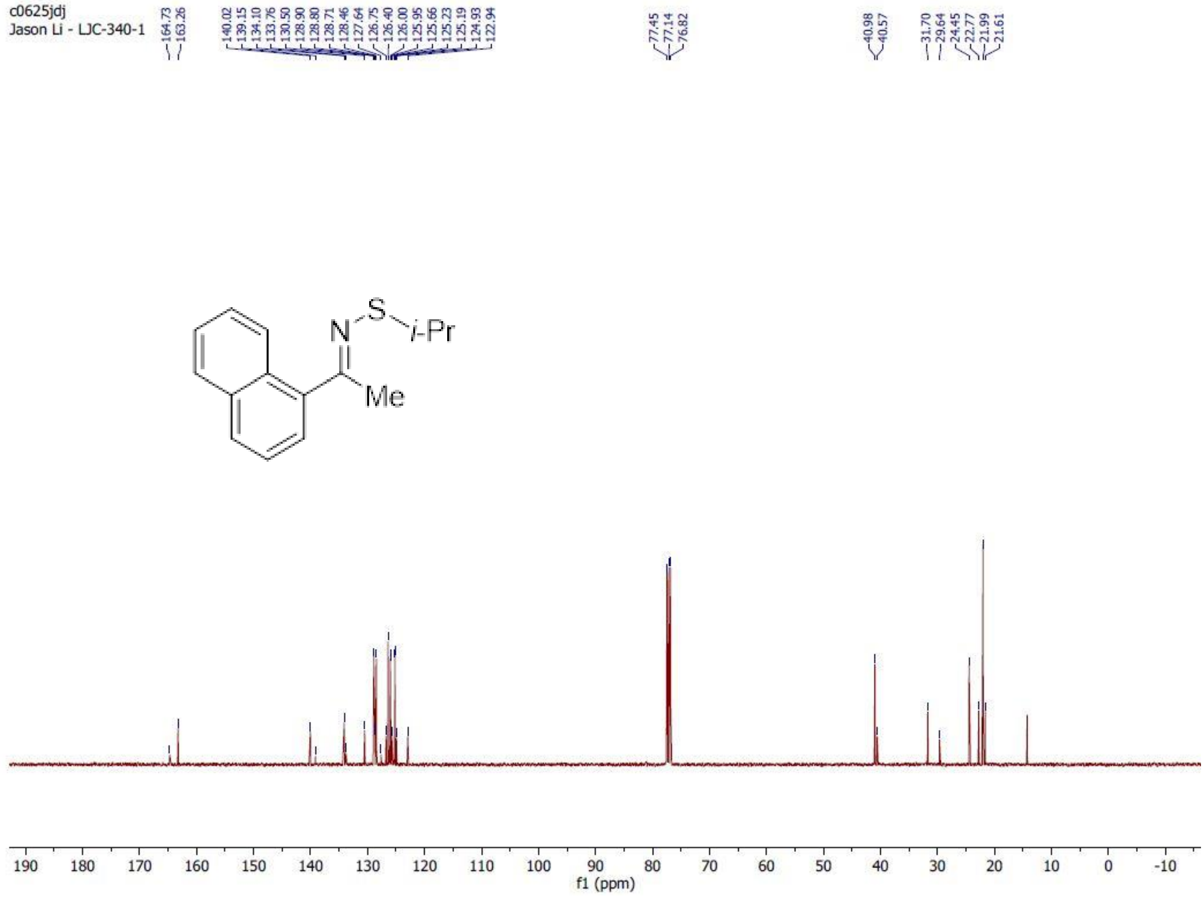

Figure S125: NMR data for **6k-*i*Pr**

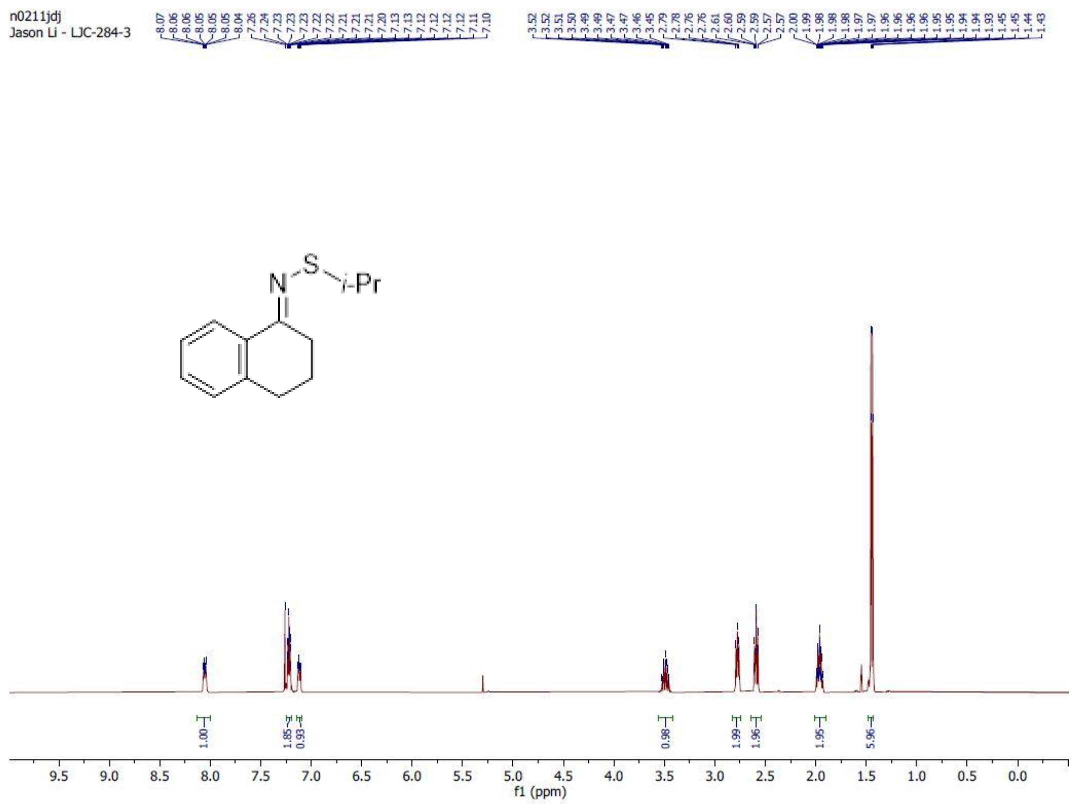

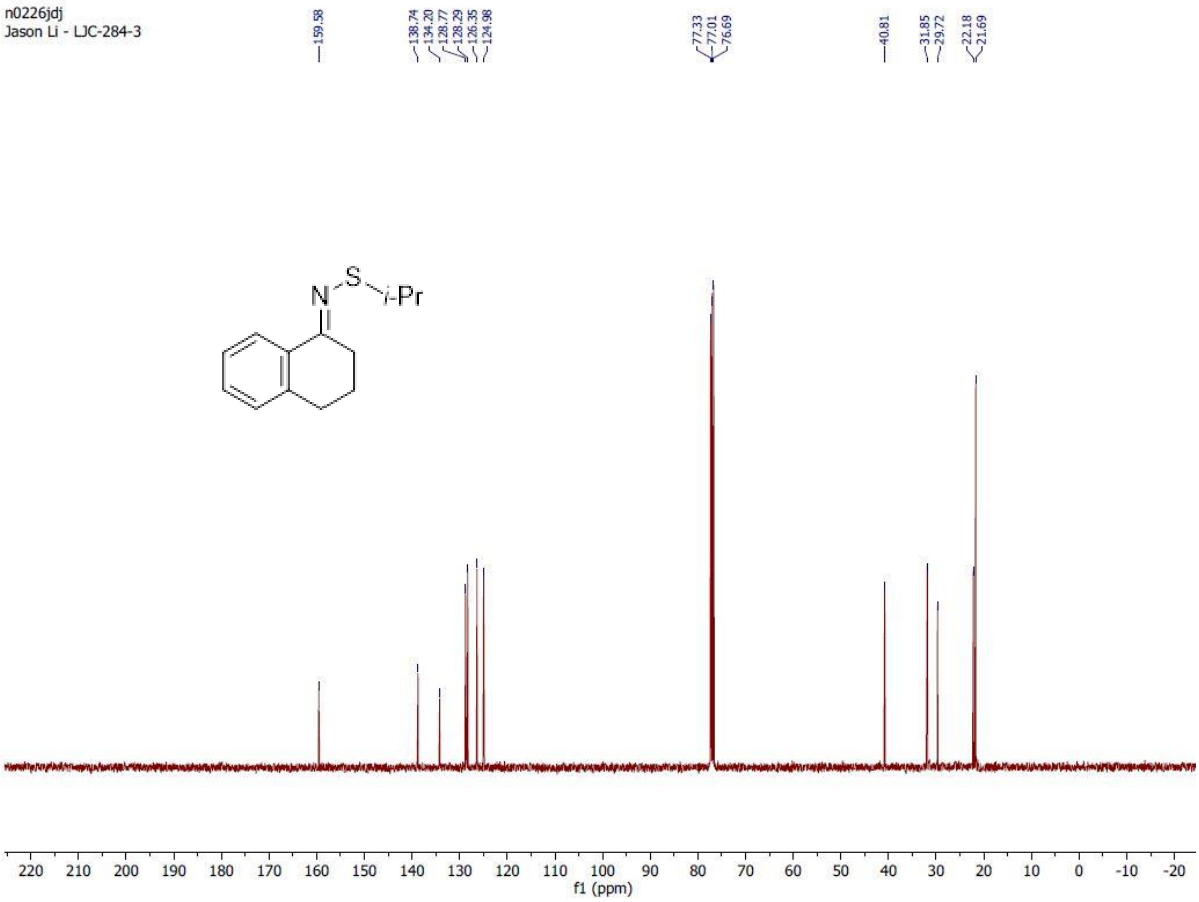

Figure S126: NMR data for **6l-*i*Pr**

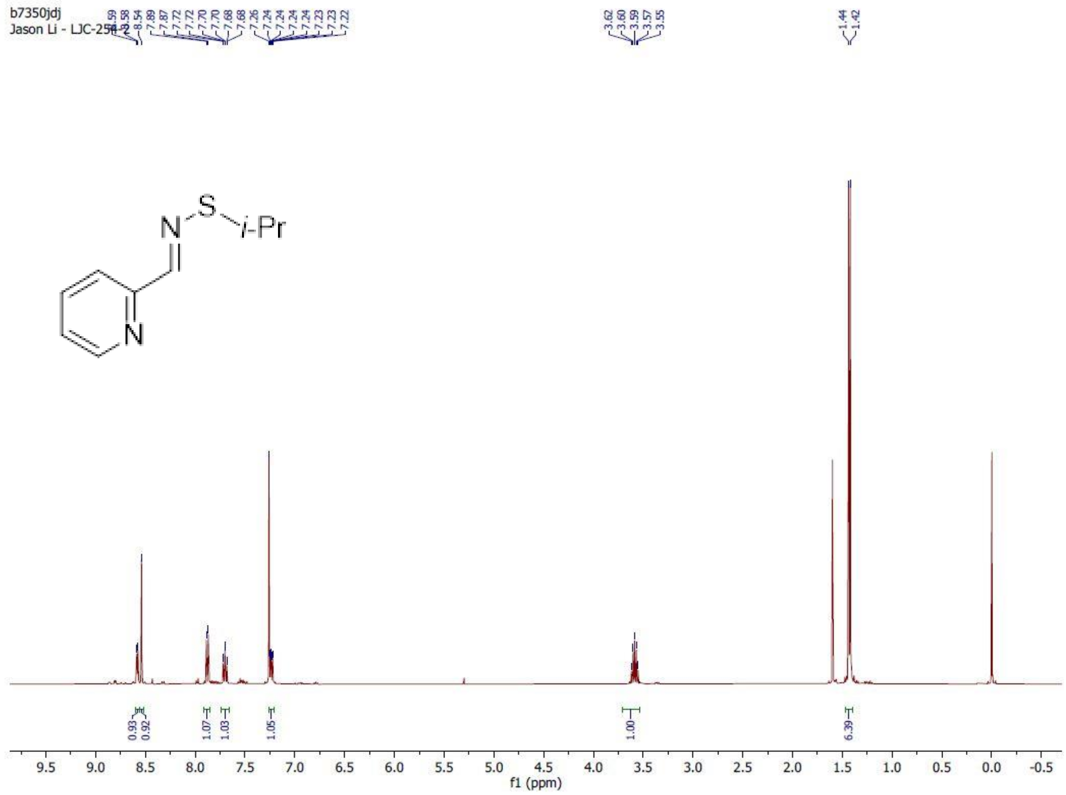

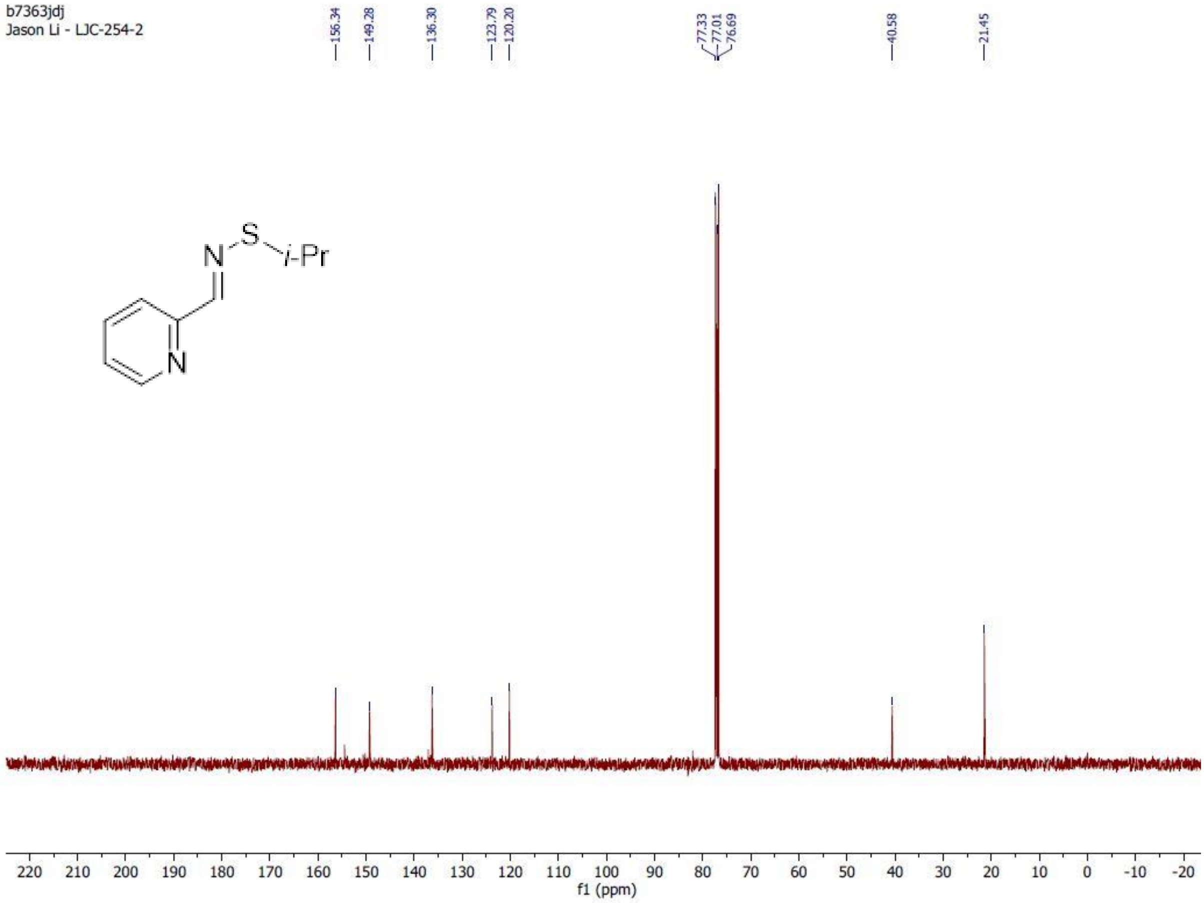

Figure S127: NMR data for **6a-*t*Bu**

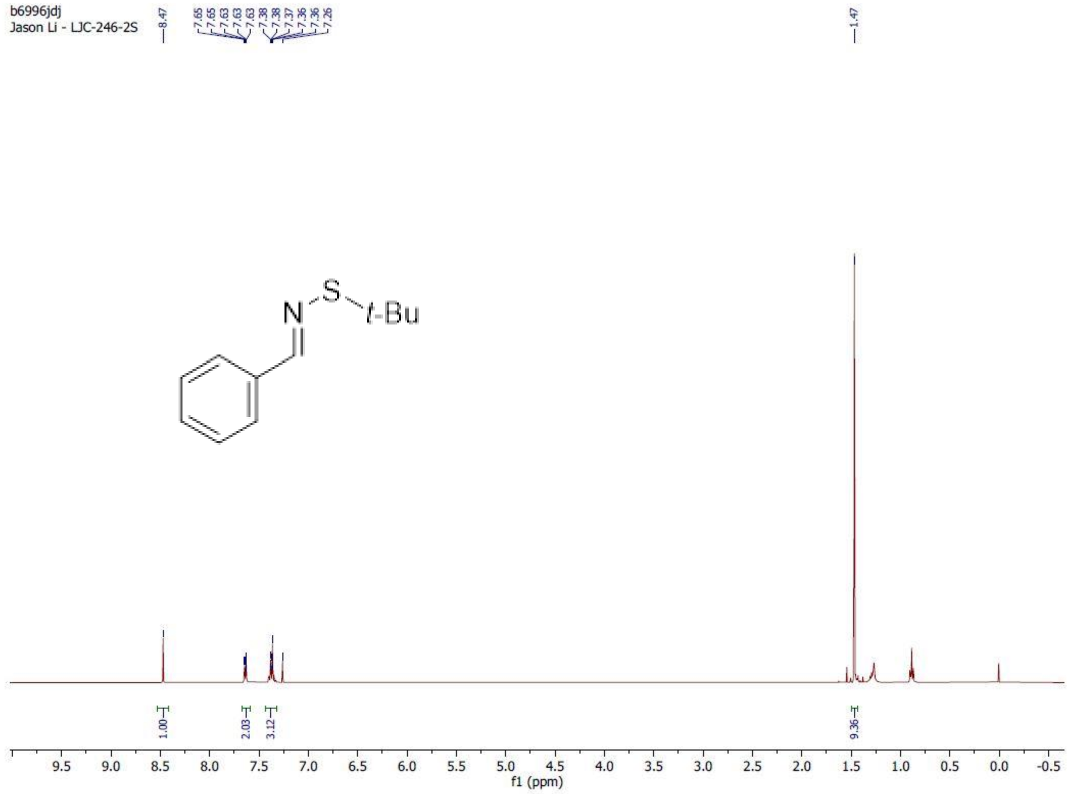

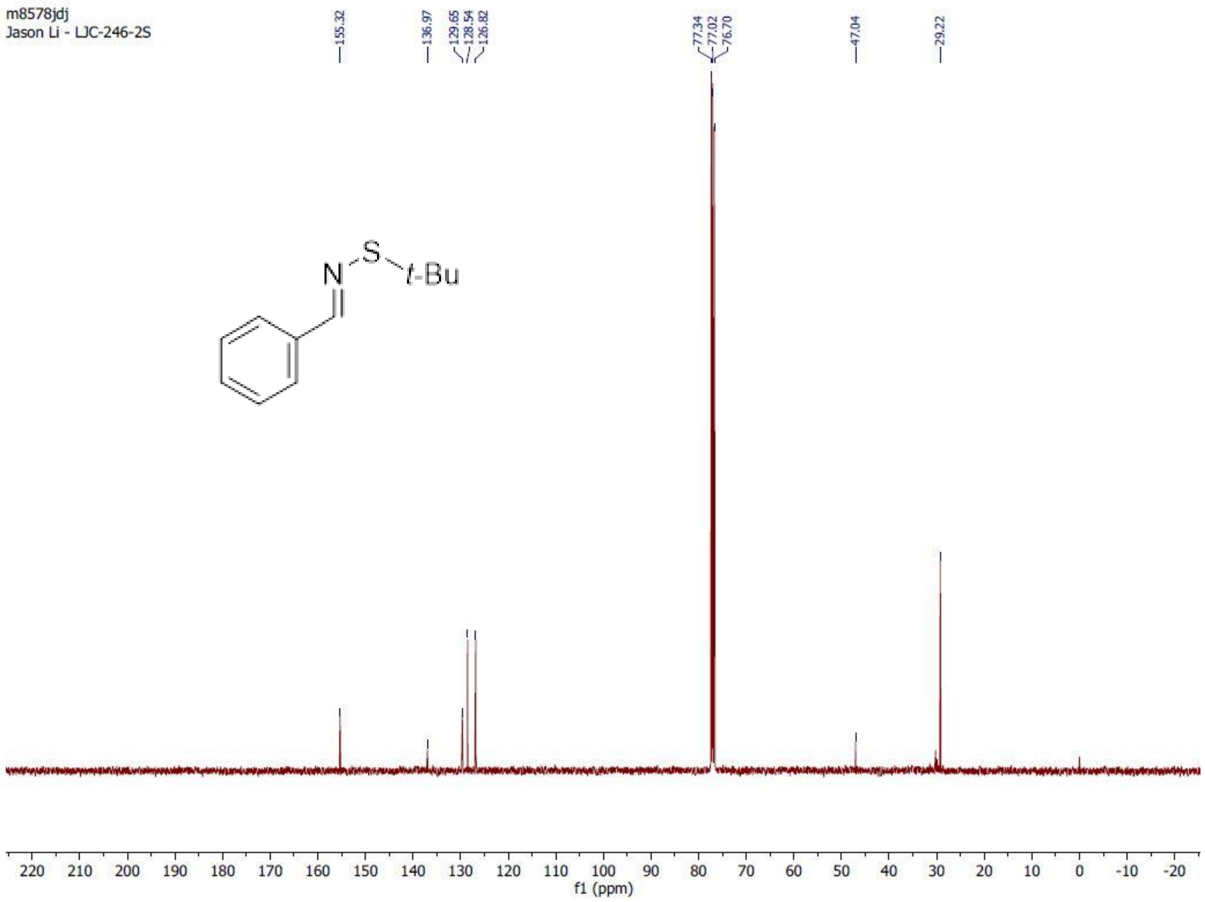

Figure S128: NMR data for **6b-*t*Bu**

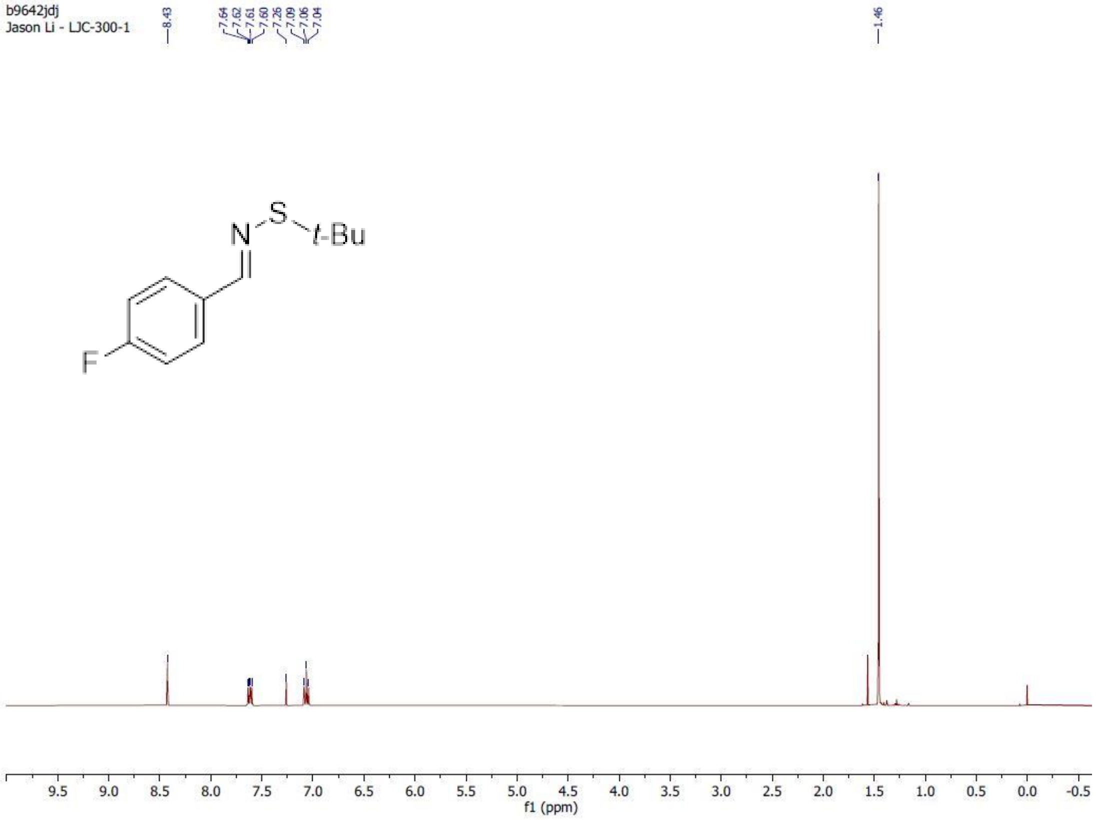

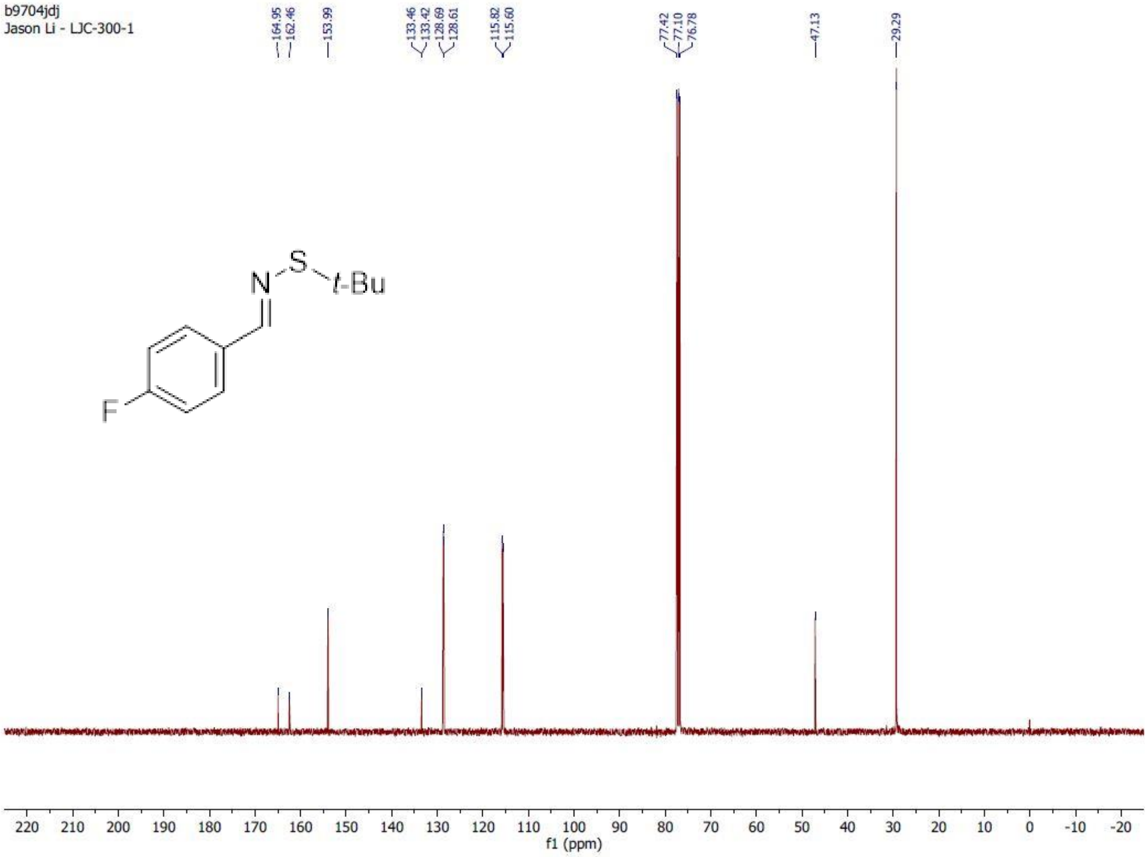

Figure S129: NMR data for **6c-*t*Bu**

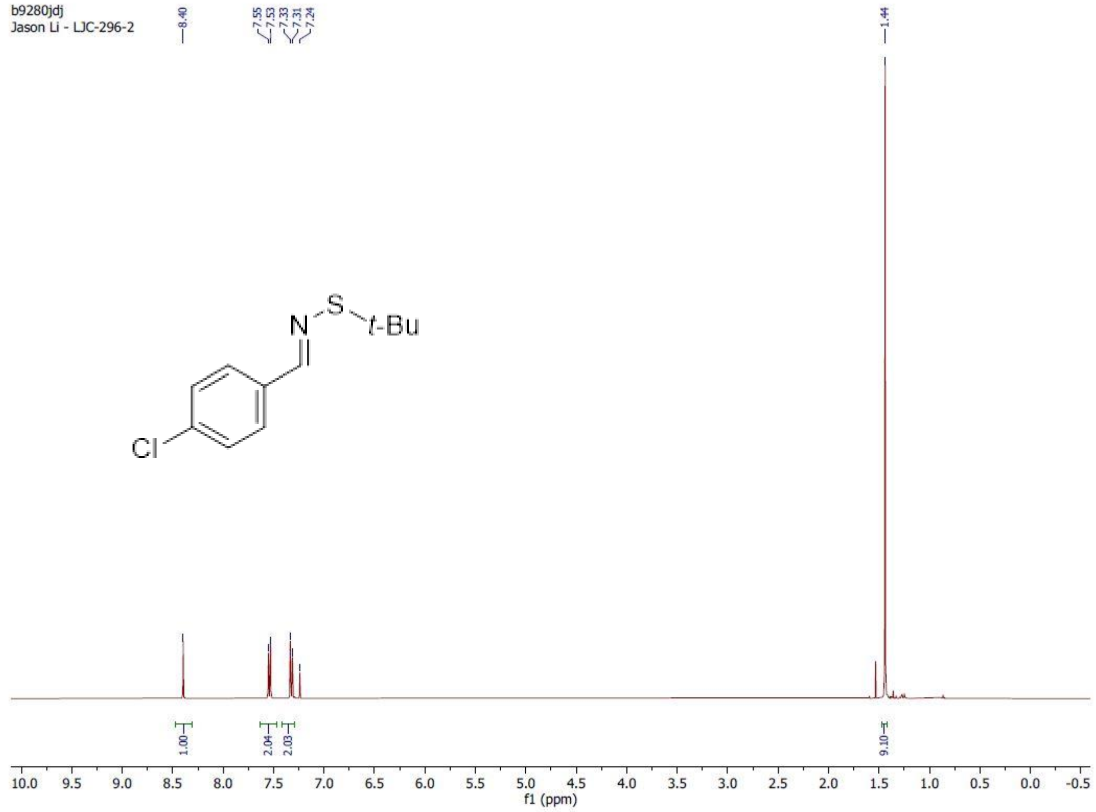

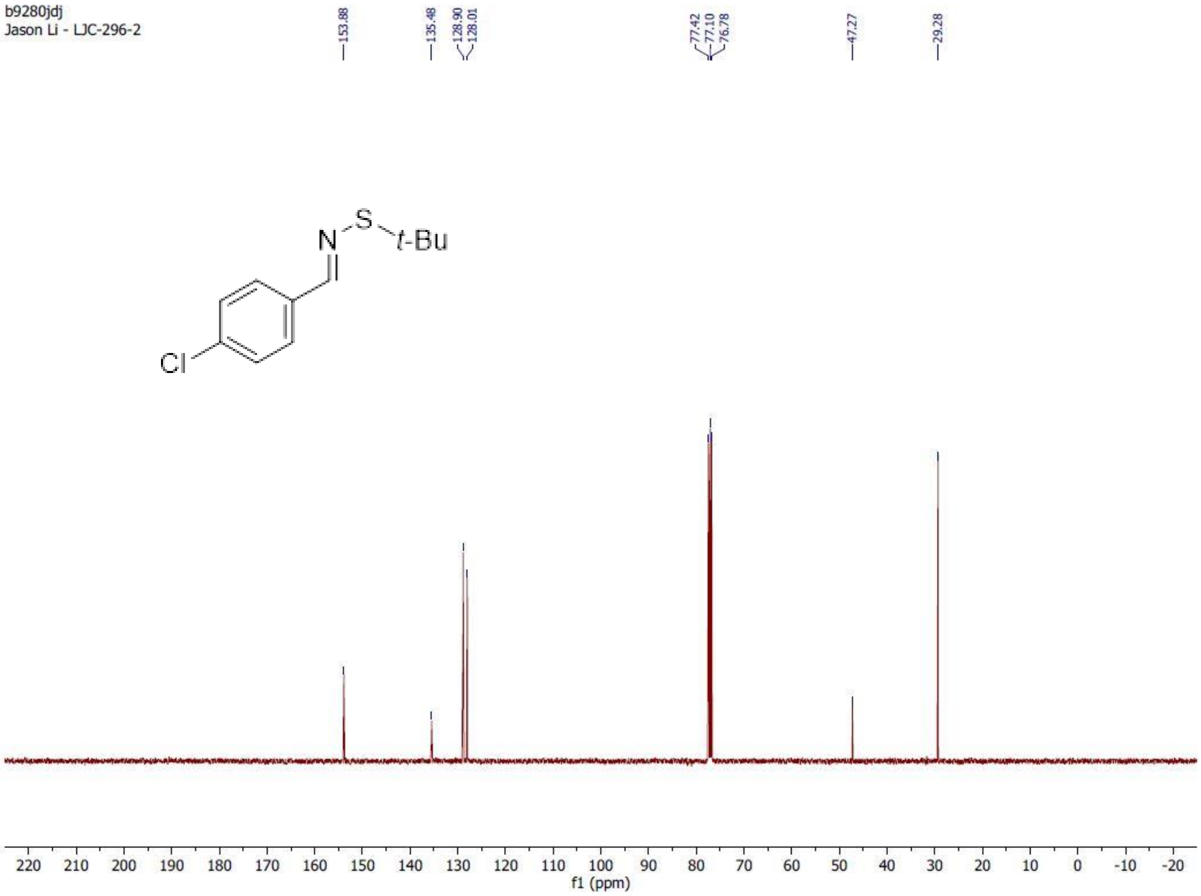

Figure S130: NMR data for **6d-*t*Bu**

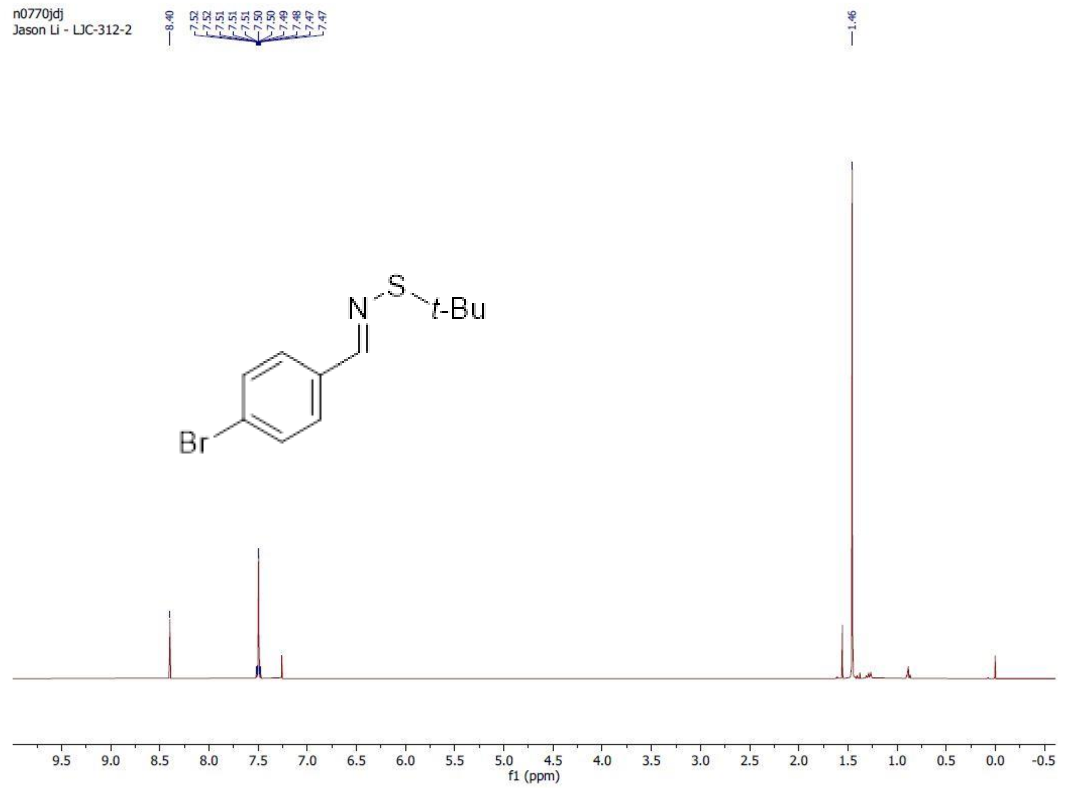

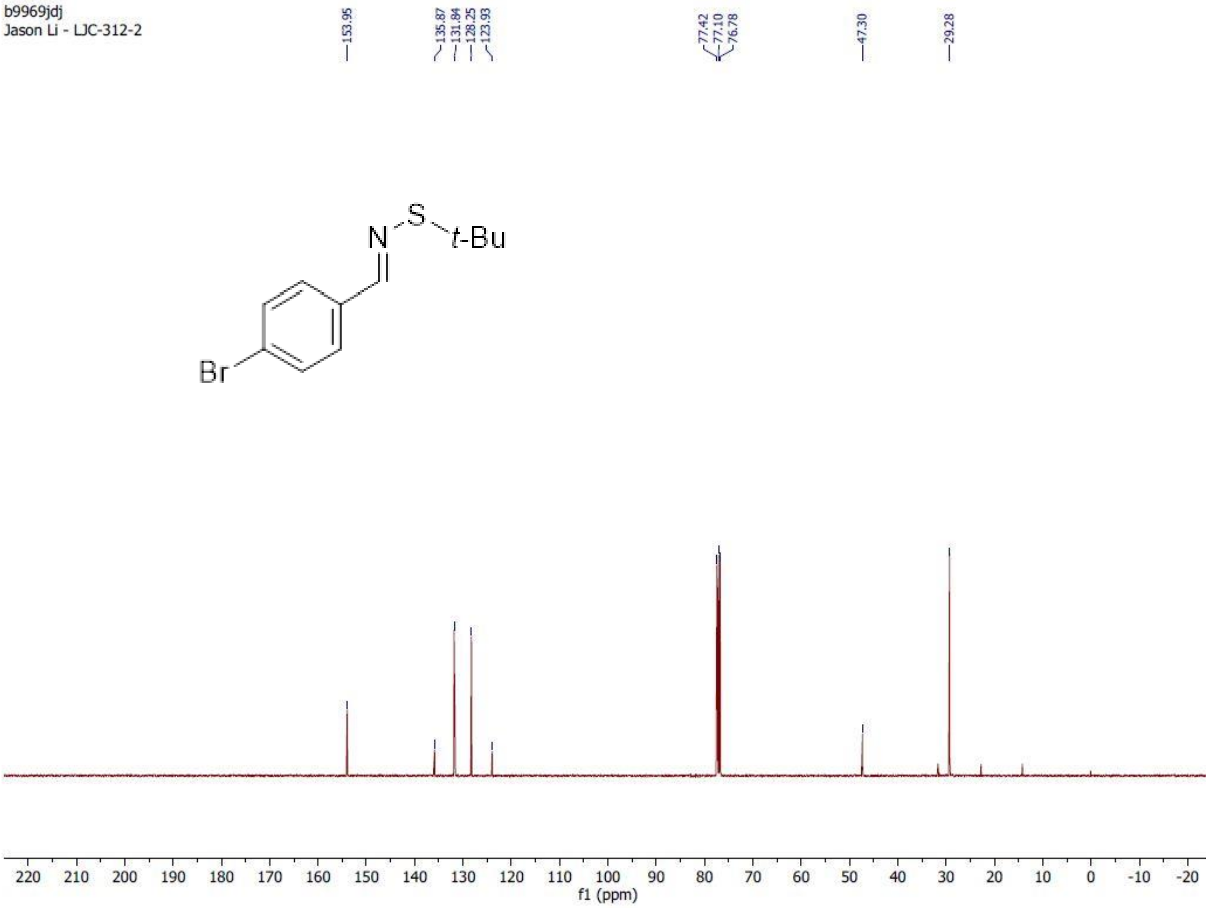

Figure S131: NMR data for **6e-*t*Bu**

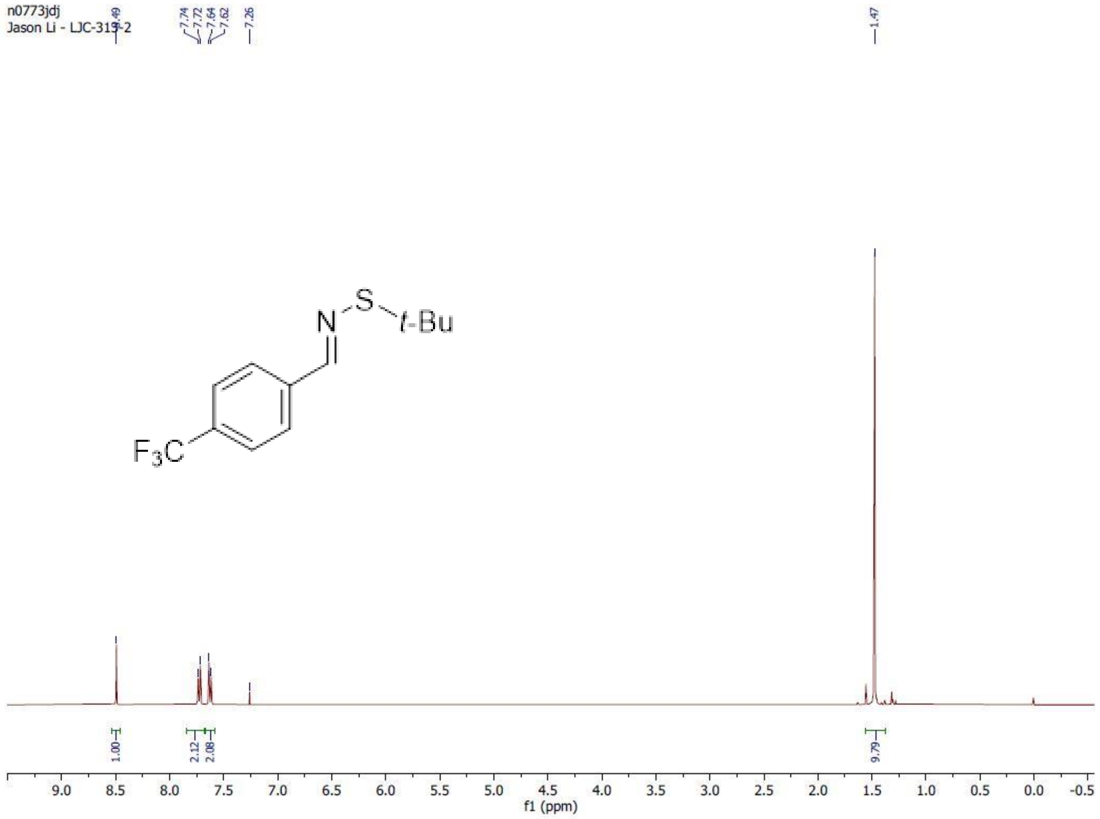

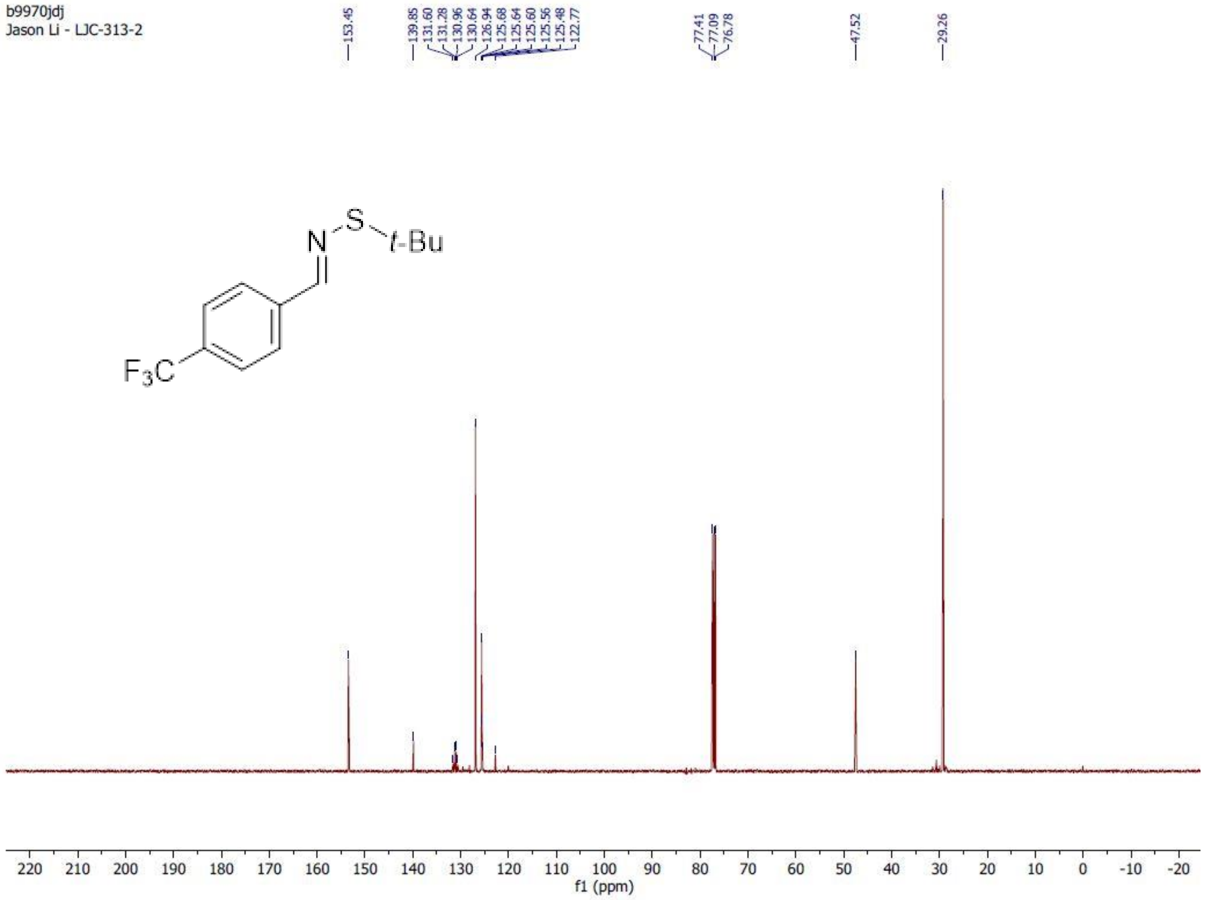

Figure S132: NMR data for **6f-*t*Bu**

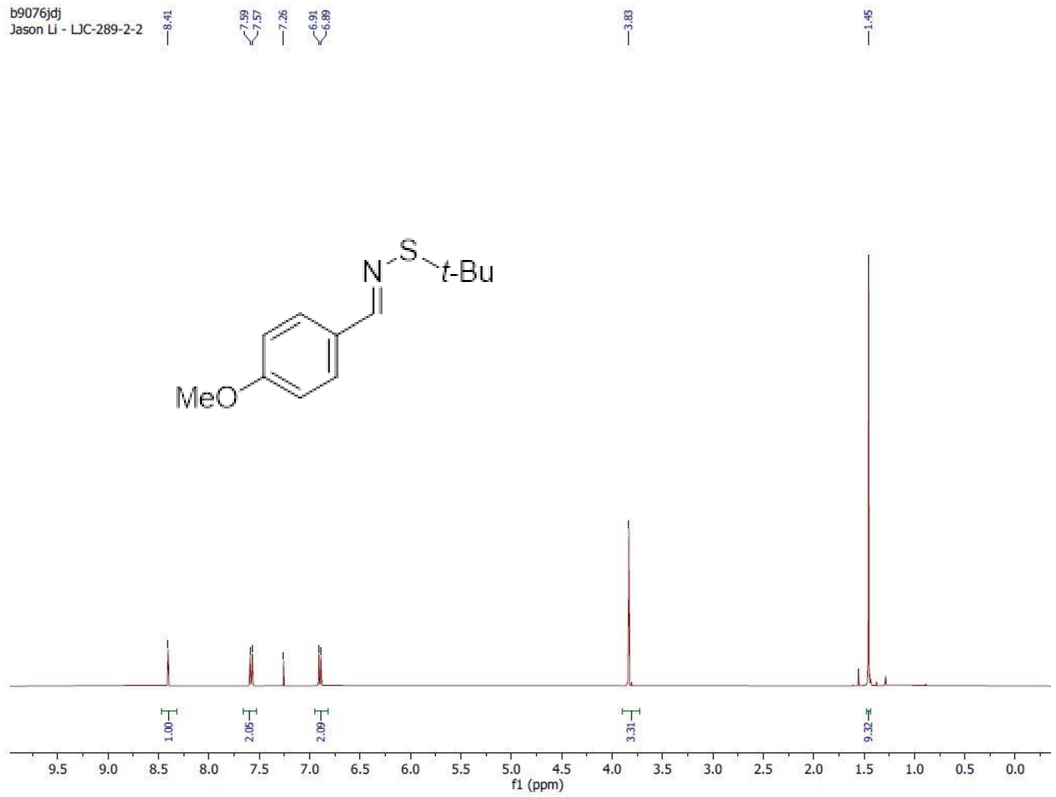

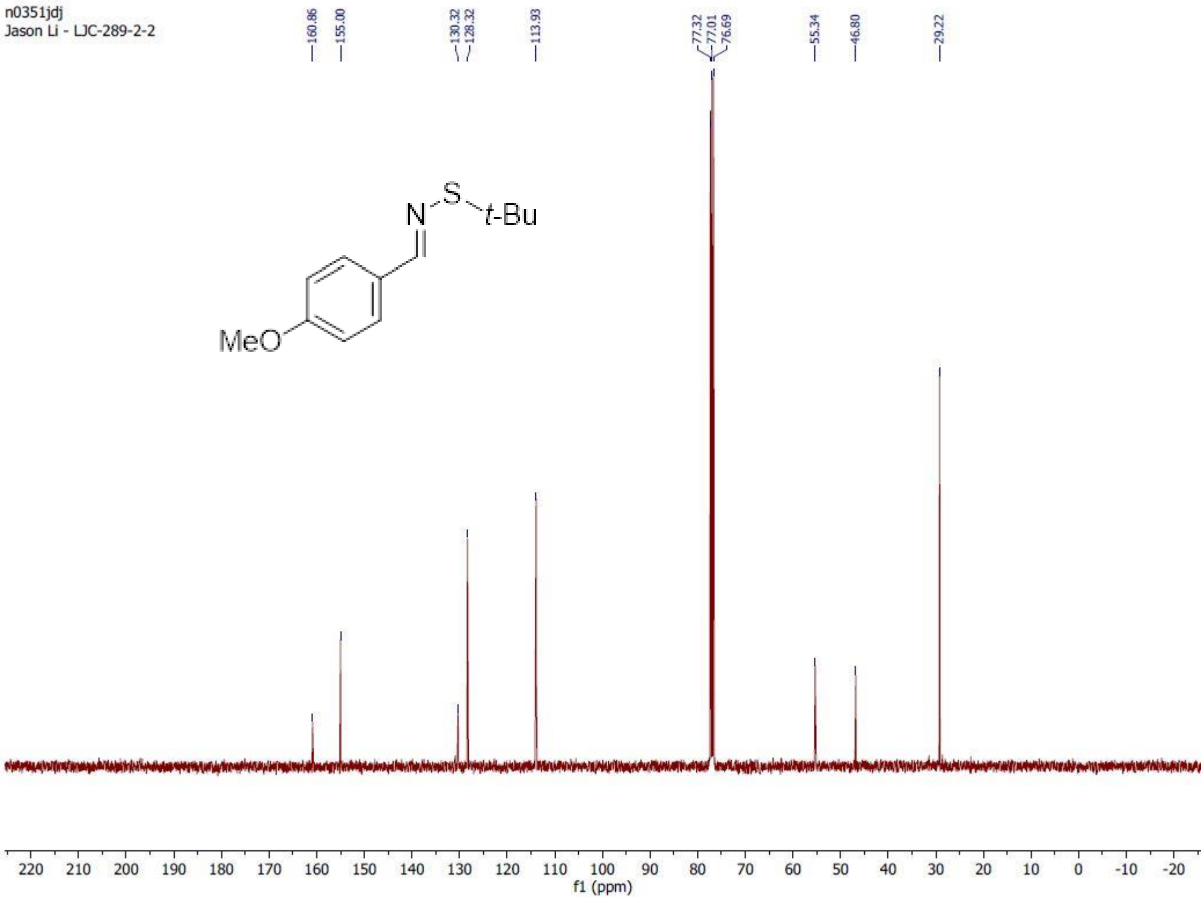

Figure S133: NMR data for **6g-*t*Bu**

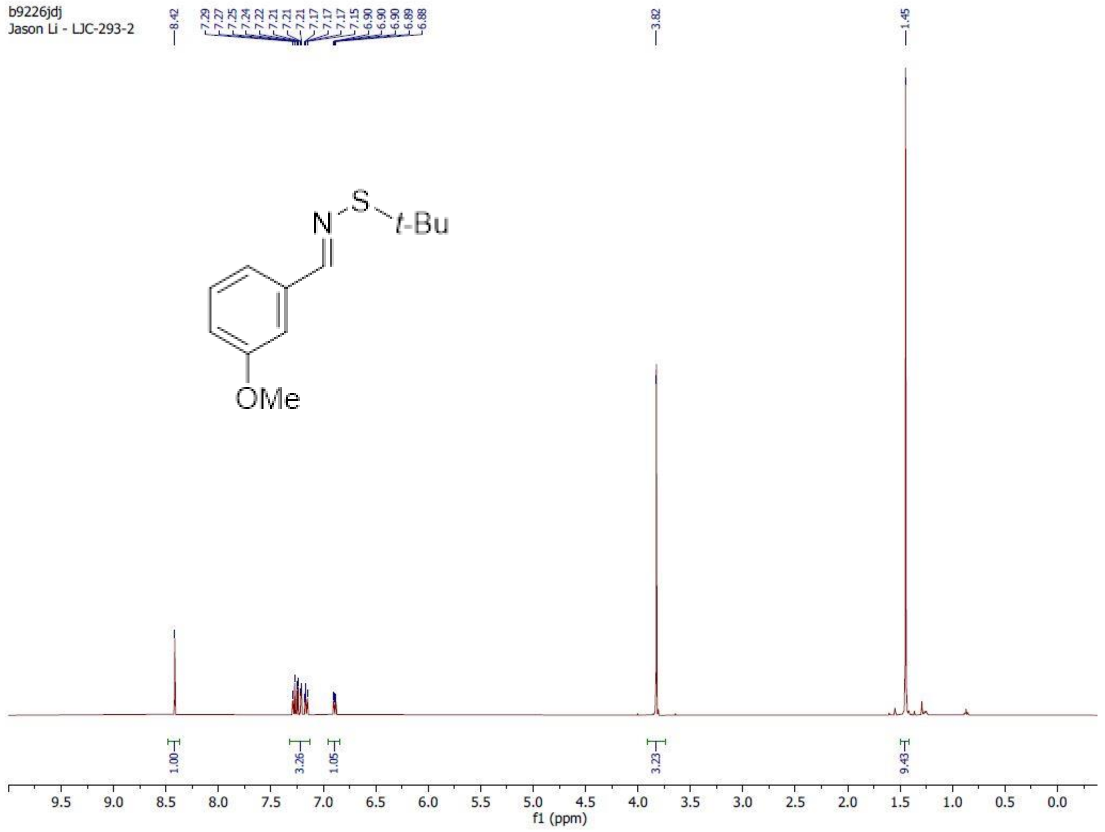

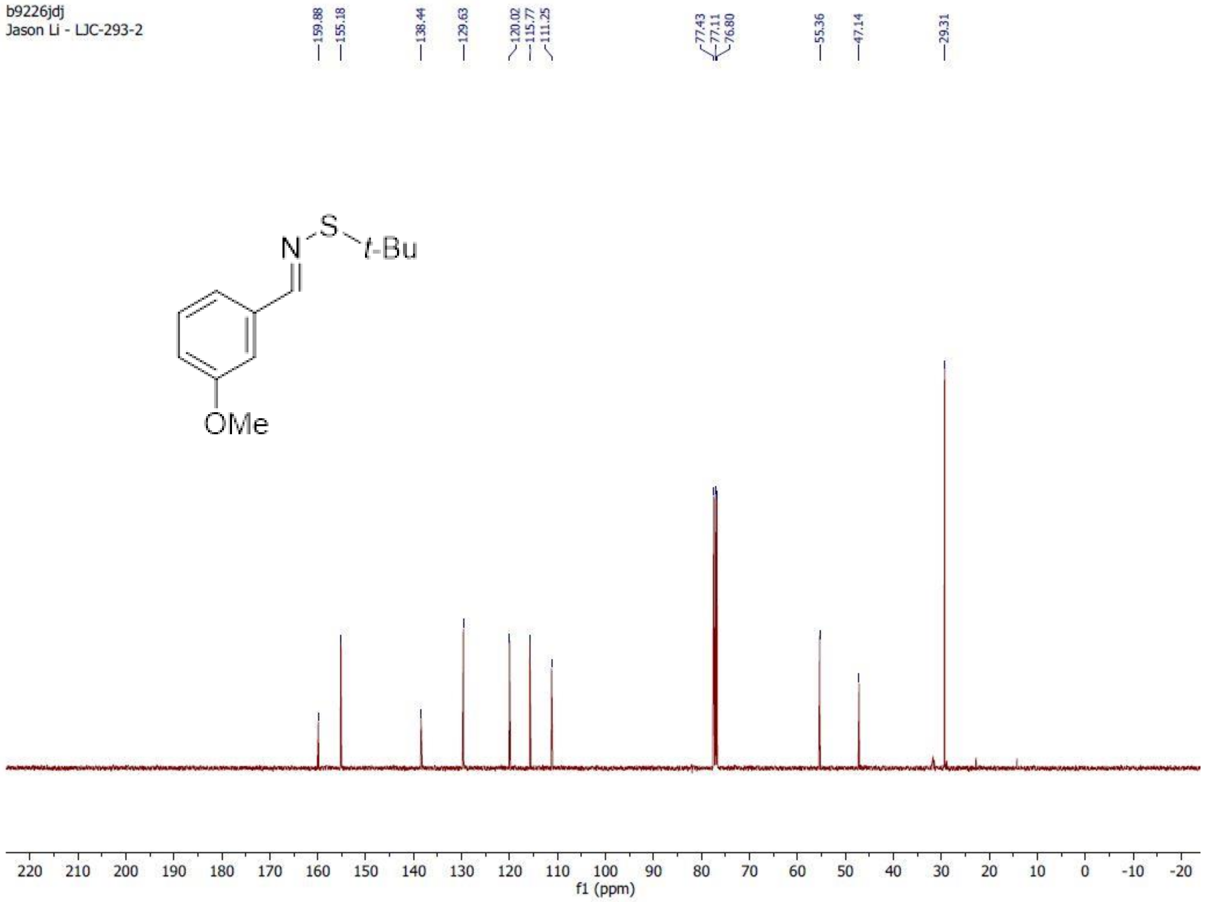

Figure S134: NMR data for **6h-*t*Bu**

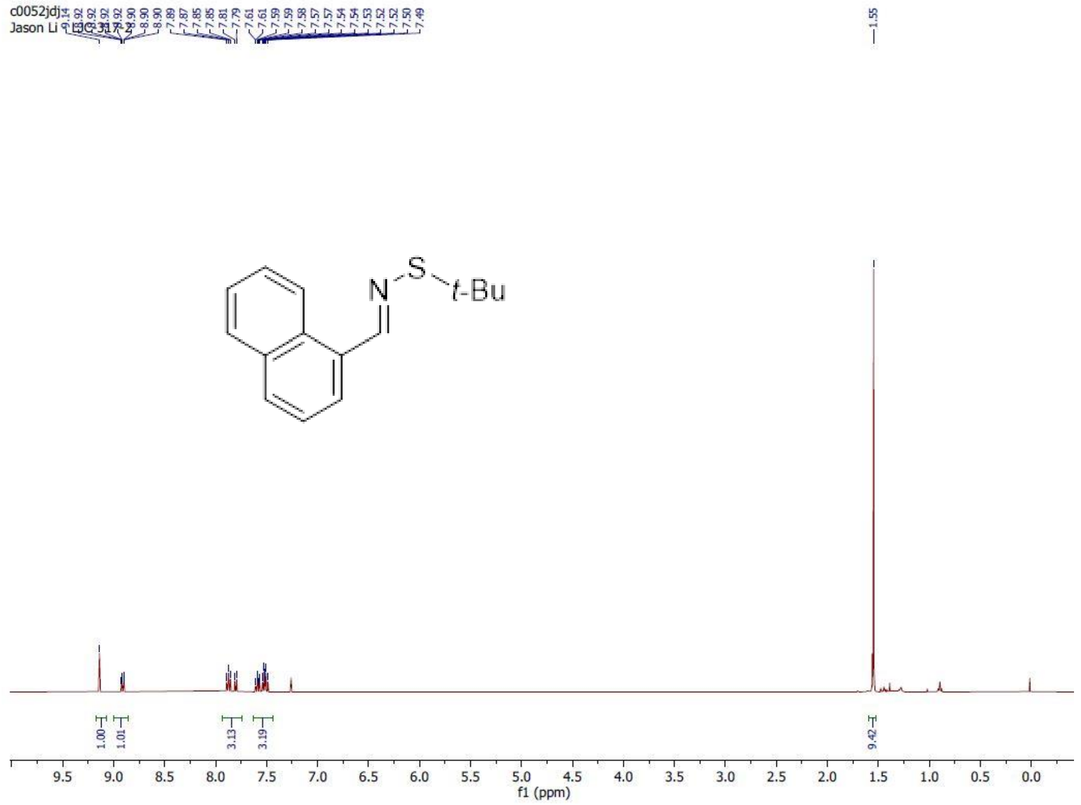

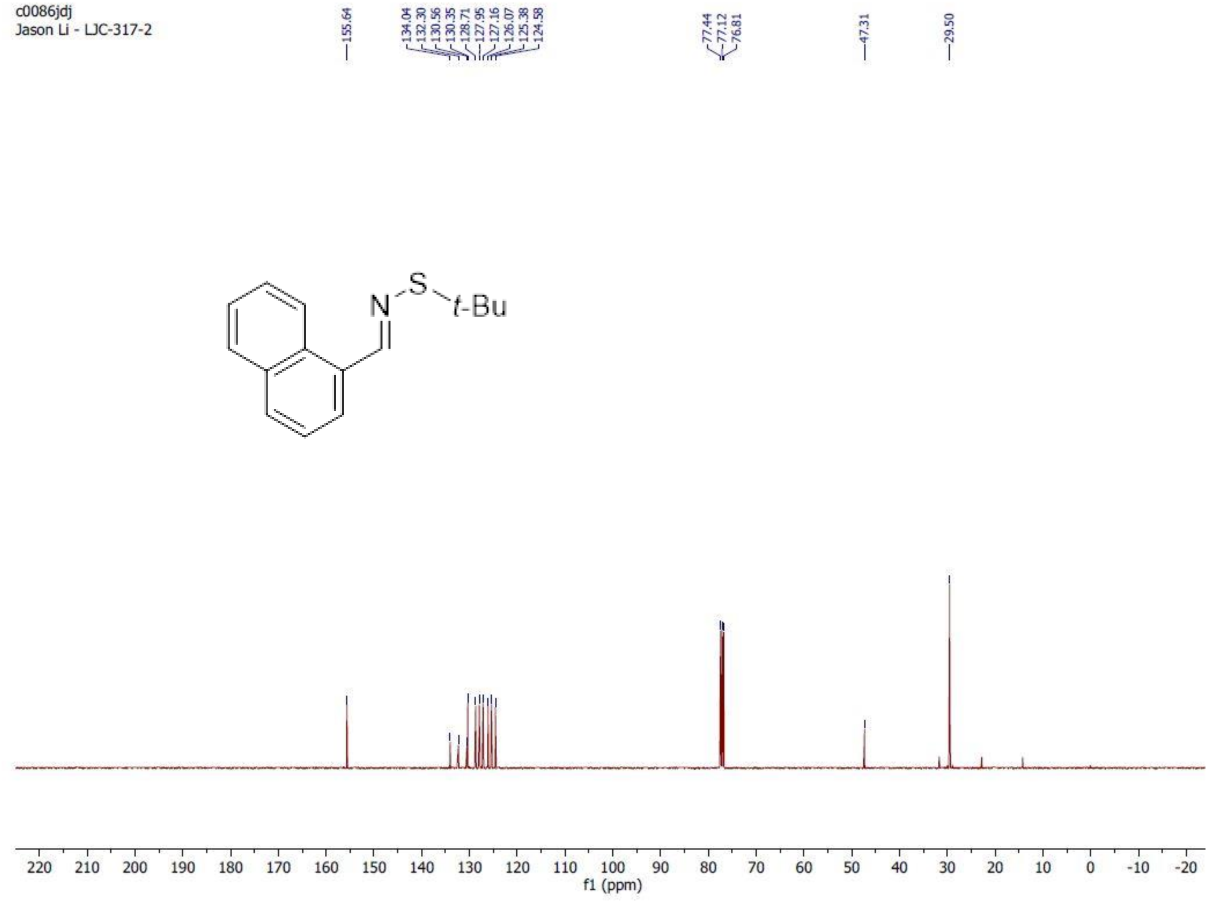

Figure S135: NMR data for **6i-*t*Bu**

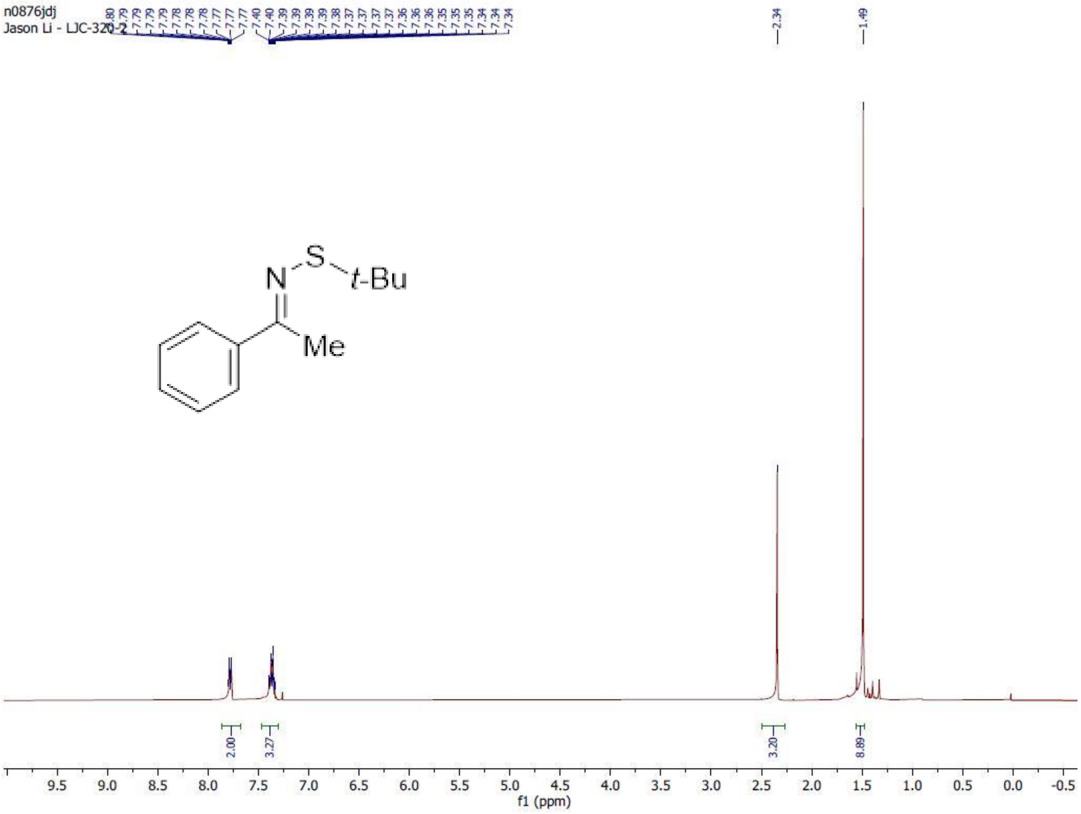

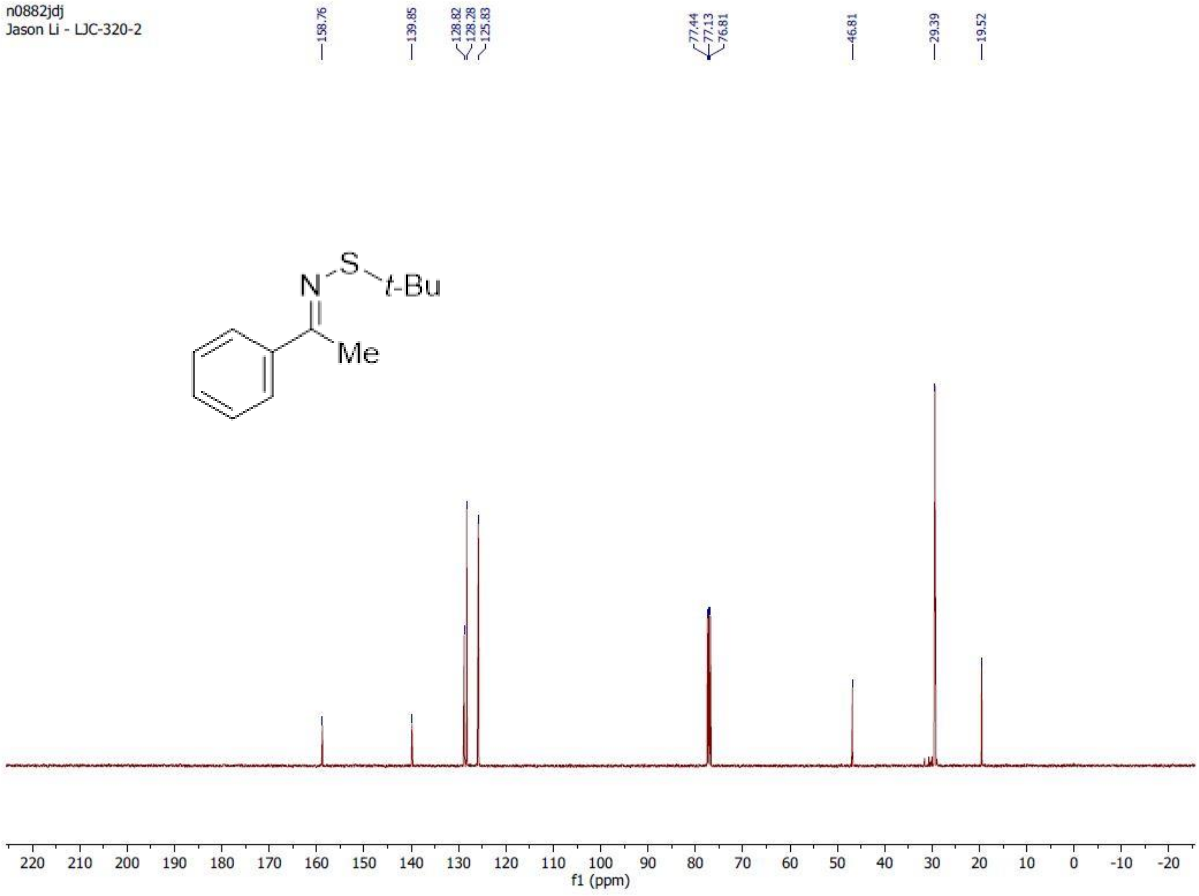

Figure S136: NMR data for **6k-*t*Bu**

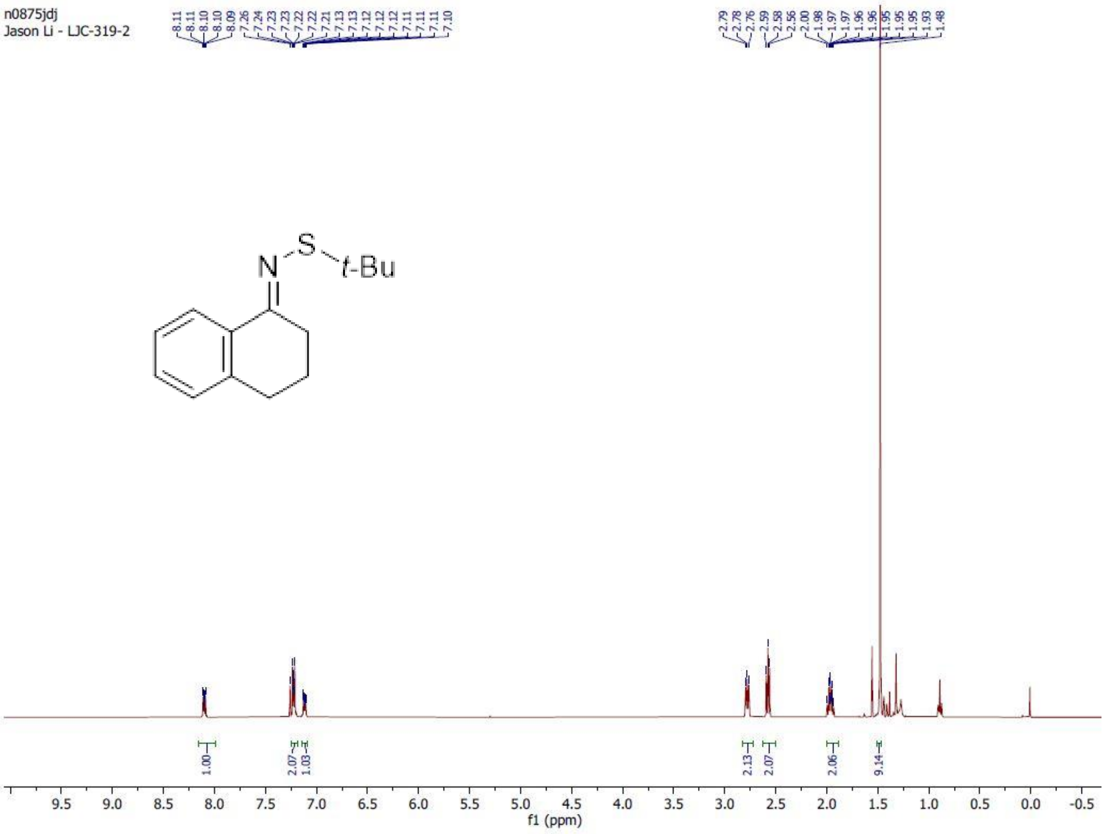

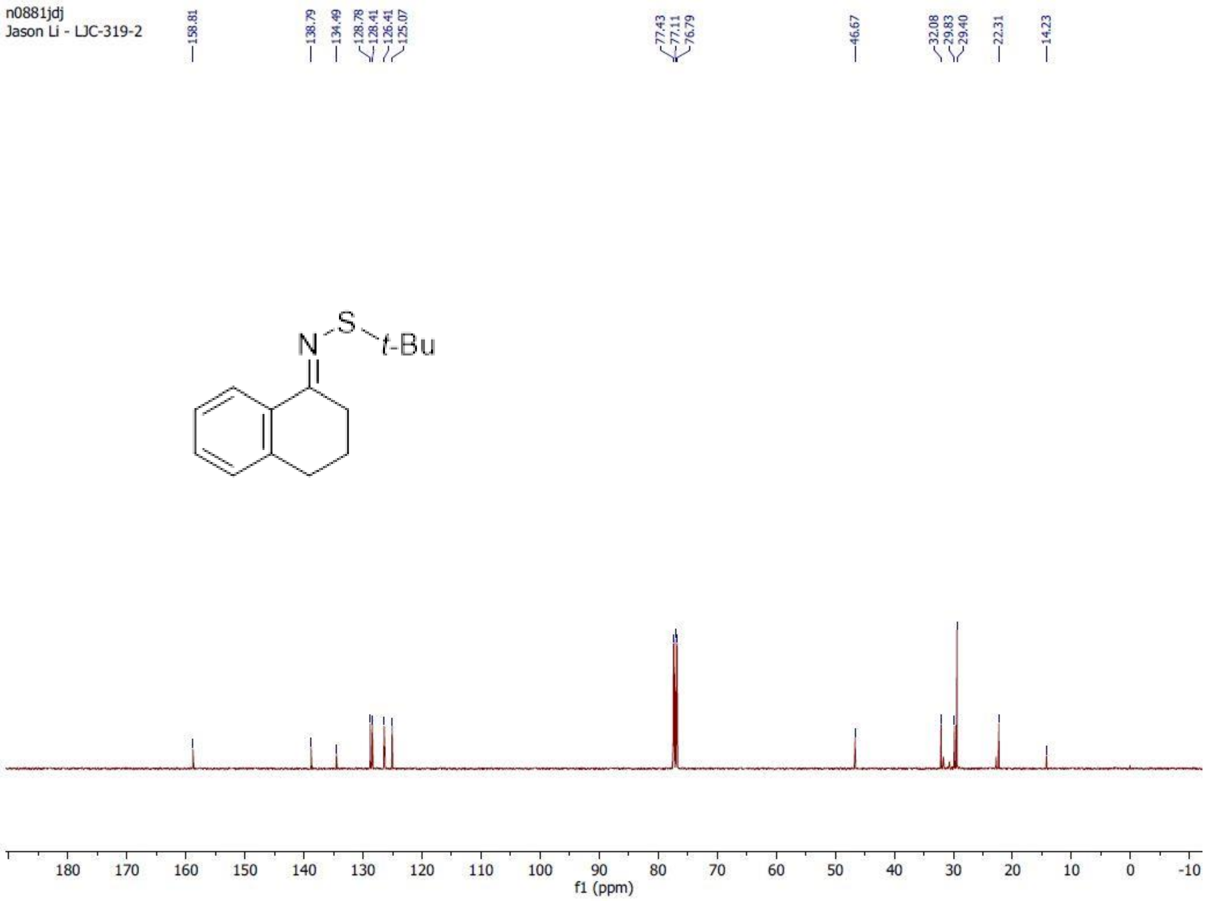

Figure S137: NMR data for **6l-*t*Bu**

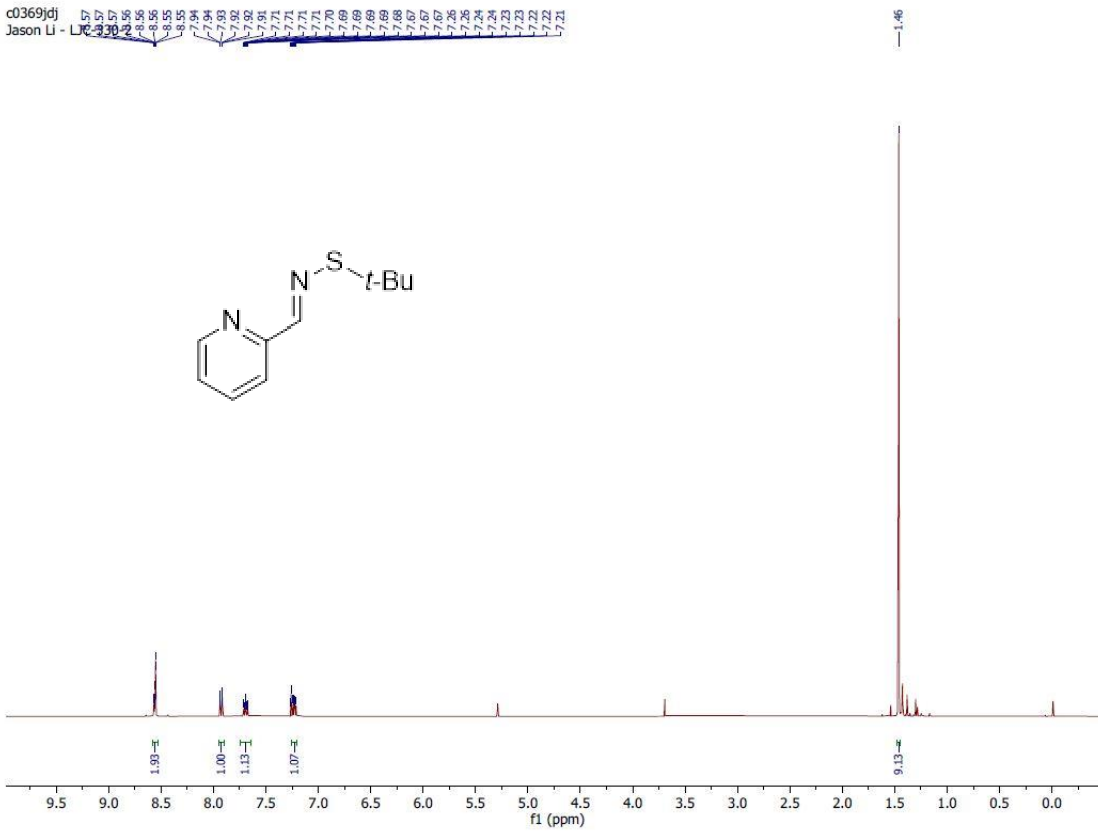

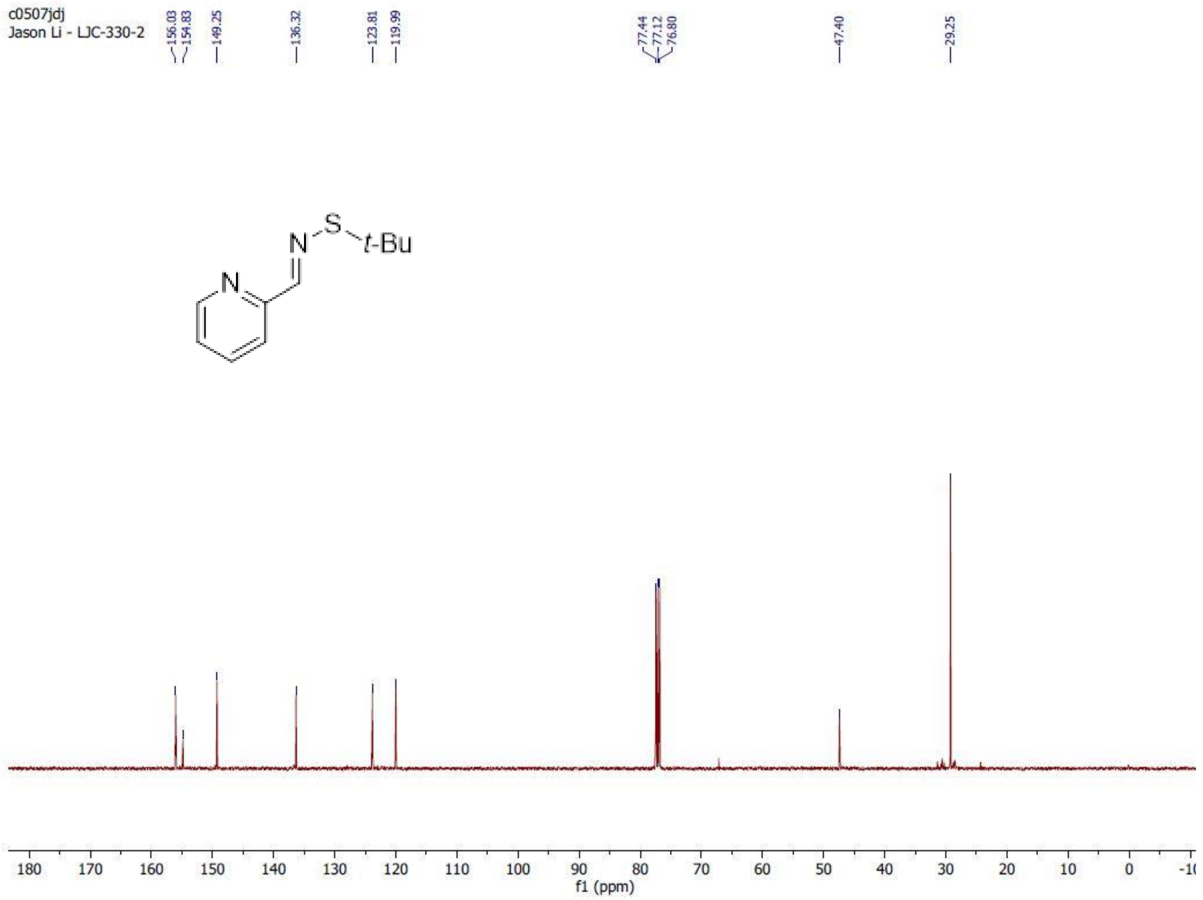

Figure S138: NMR data for **6m-*t*Bu**

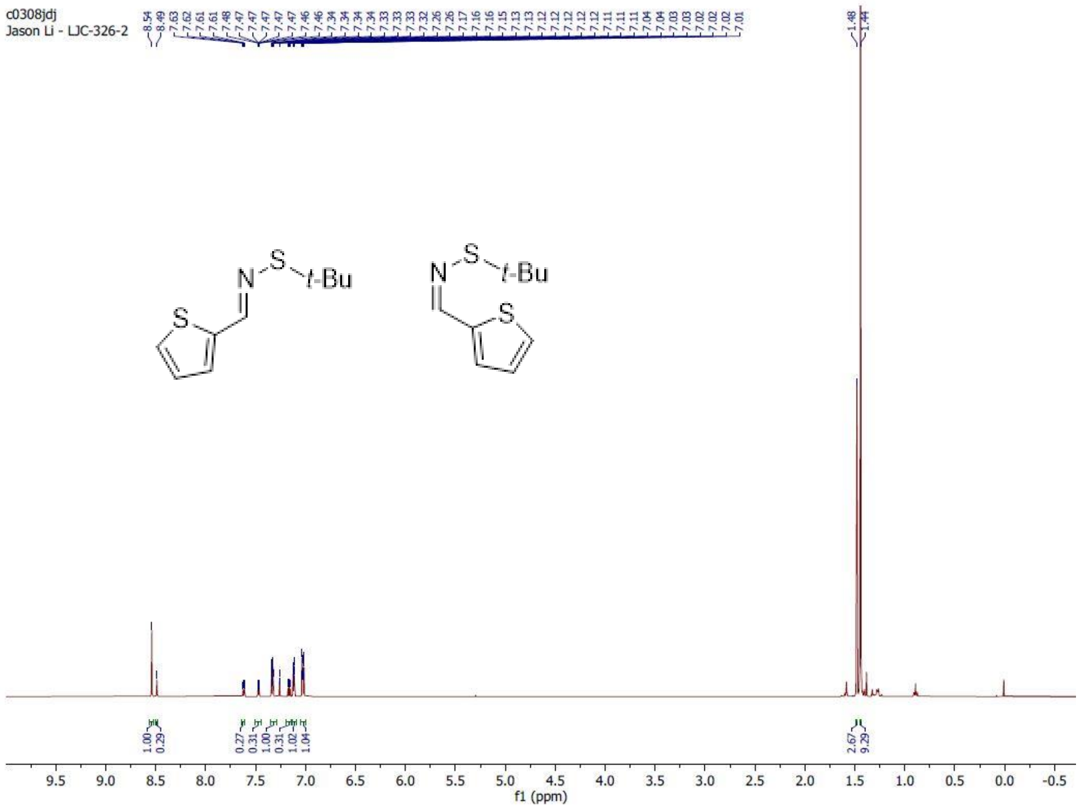

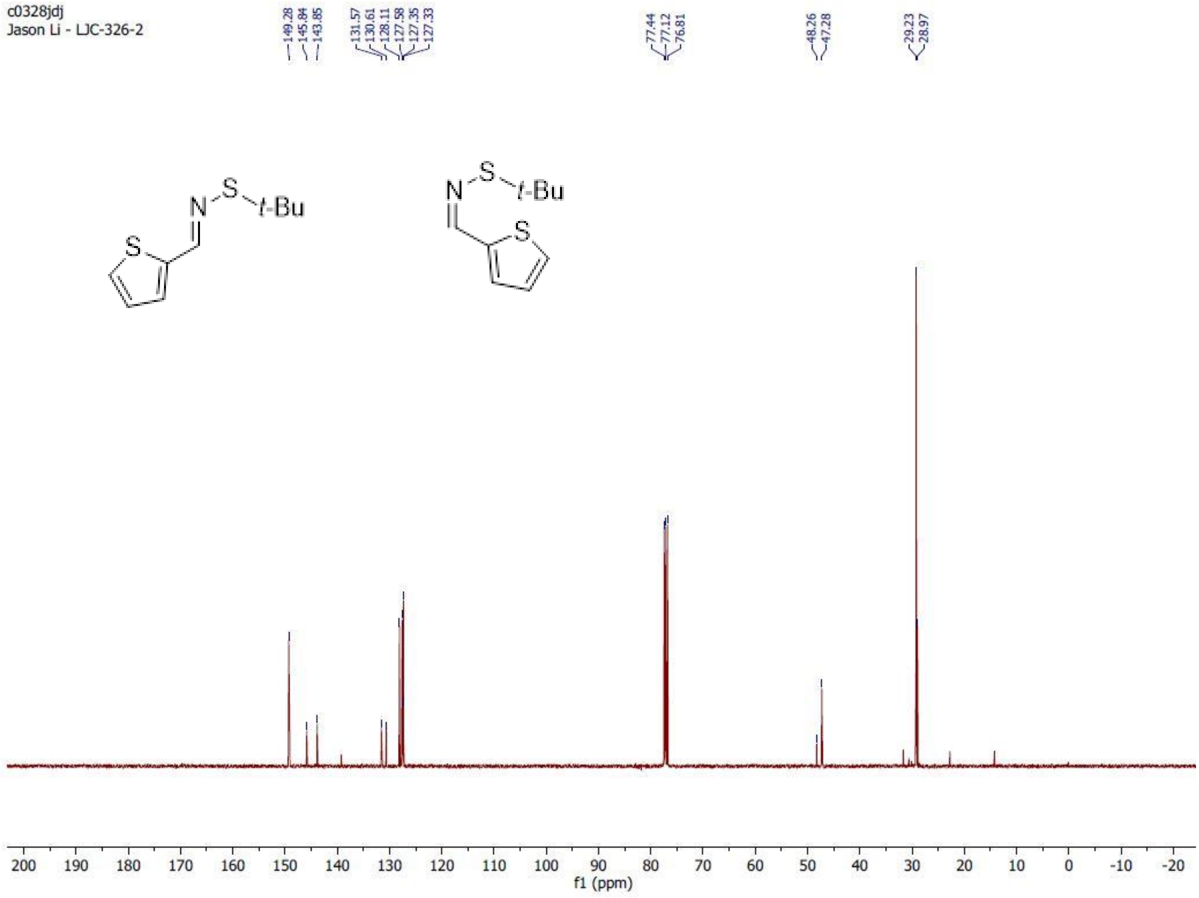

Figure S139: NMR data for **6n-*t*Bu**

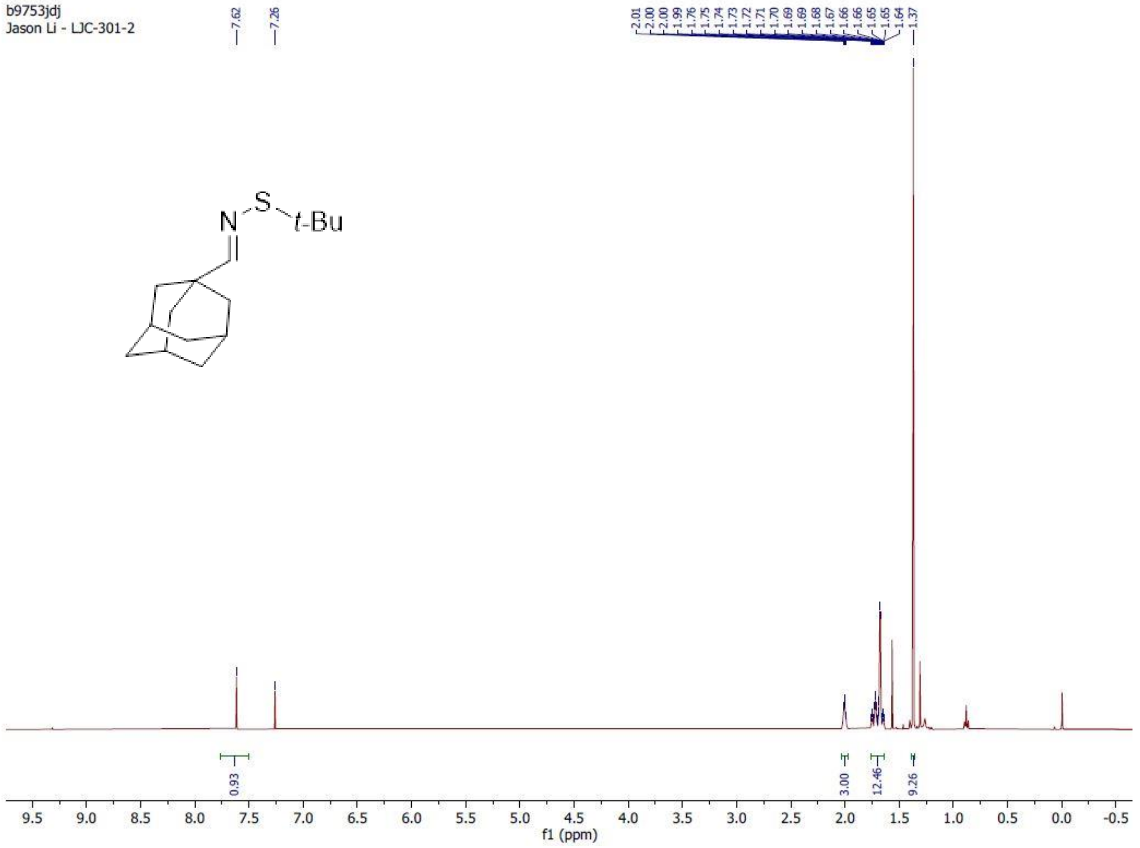

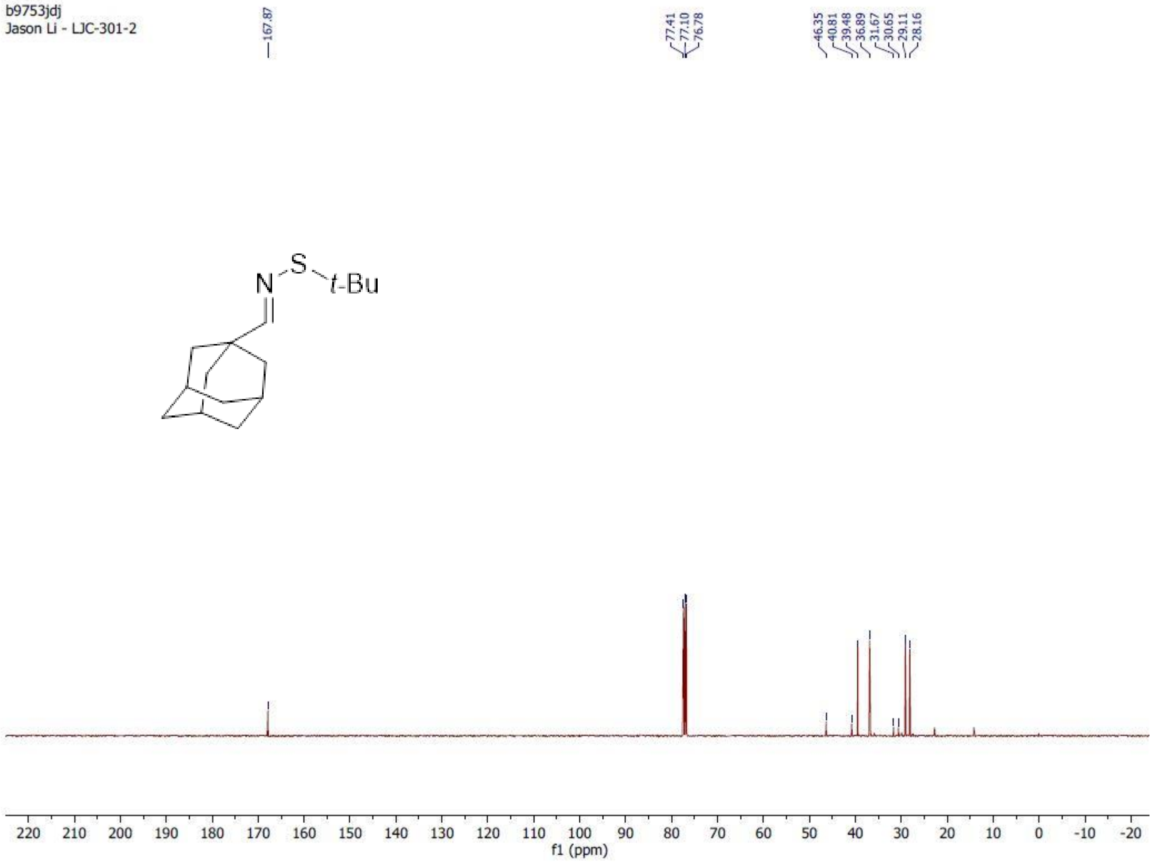

Figure S140: NMR data for **(*R*)-12a**

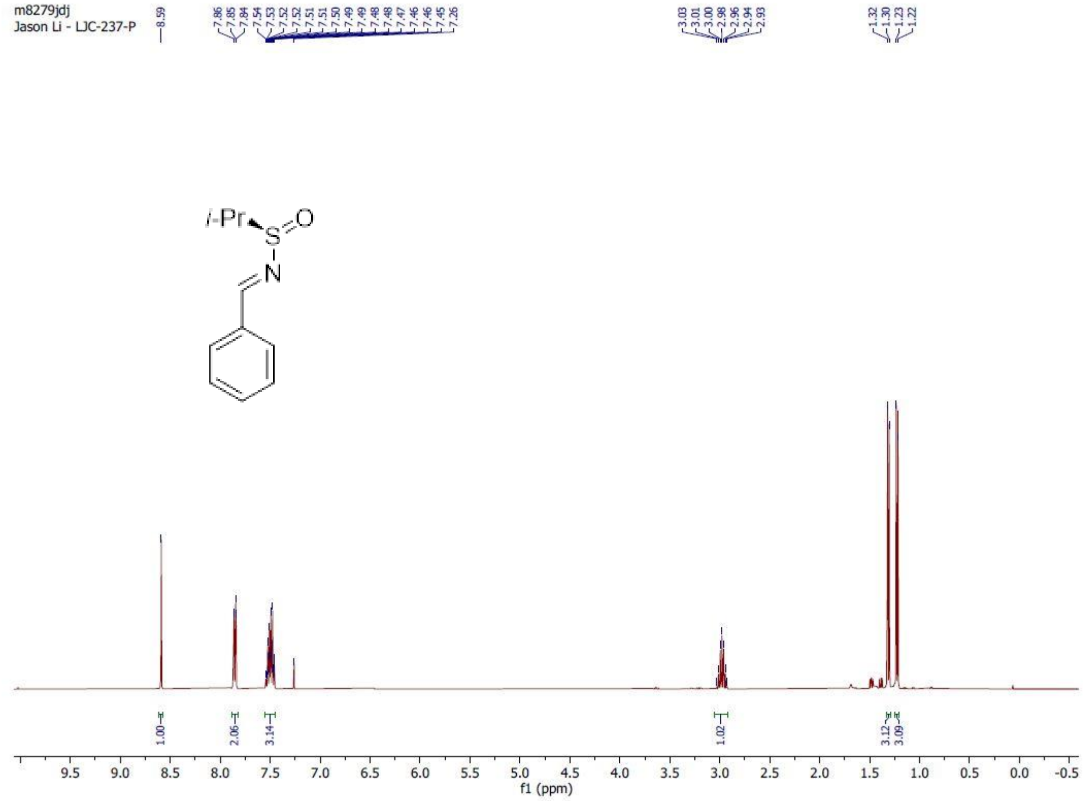

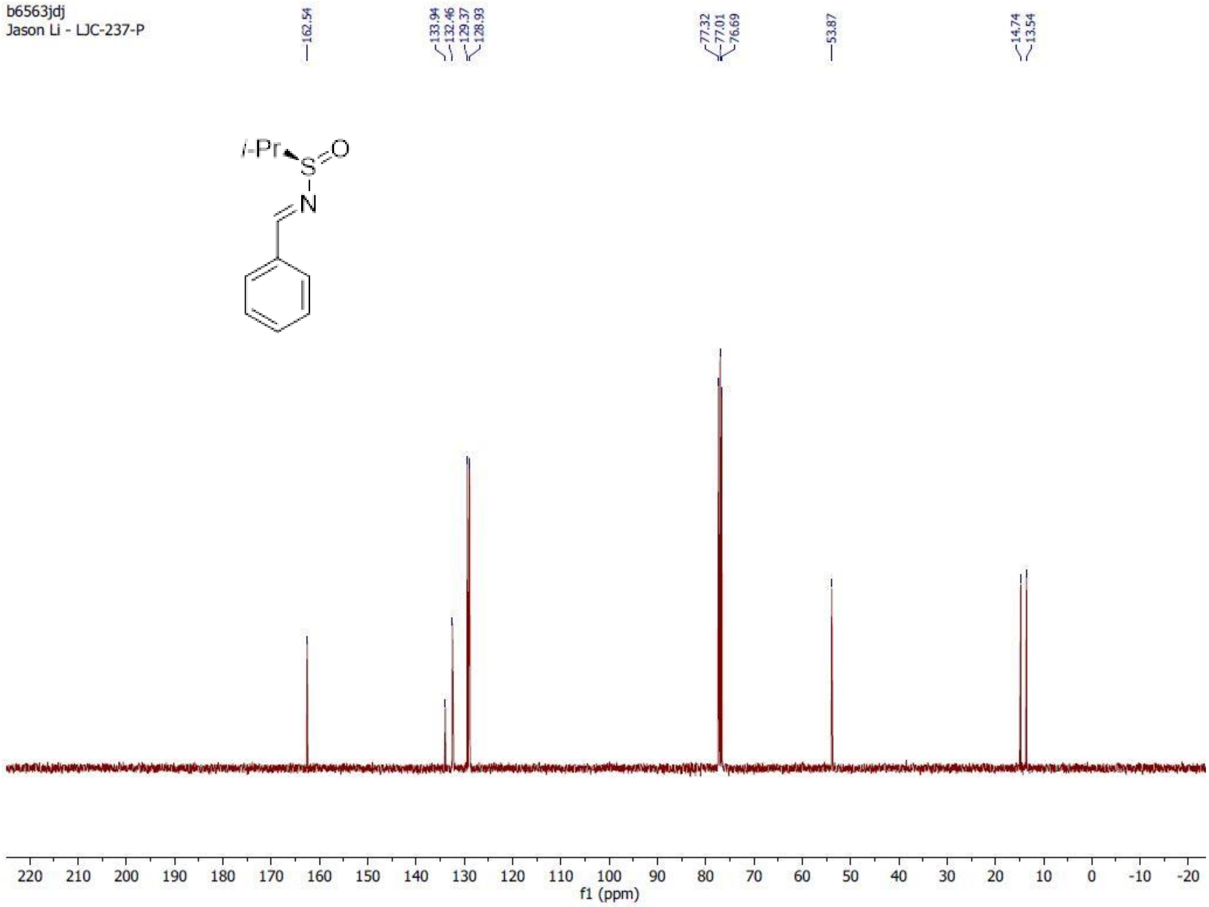

Figure S141: NMR data for **(*R*)-12b**

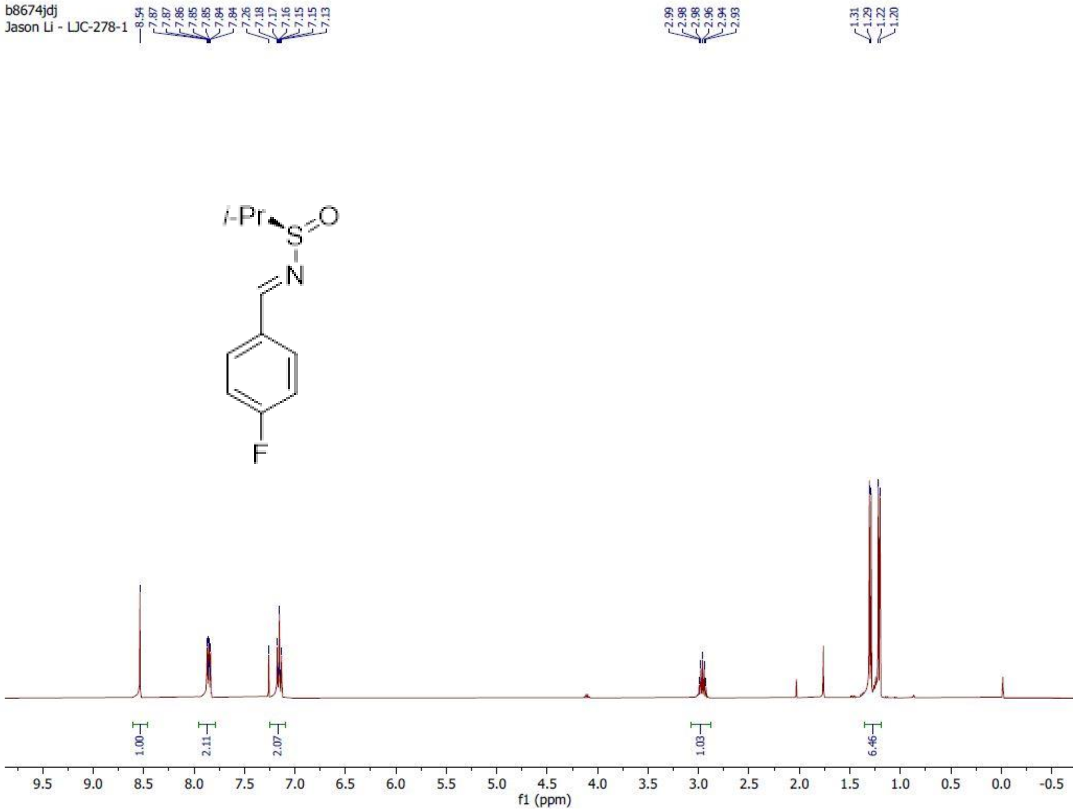

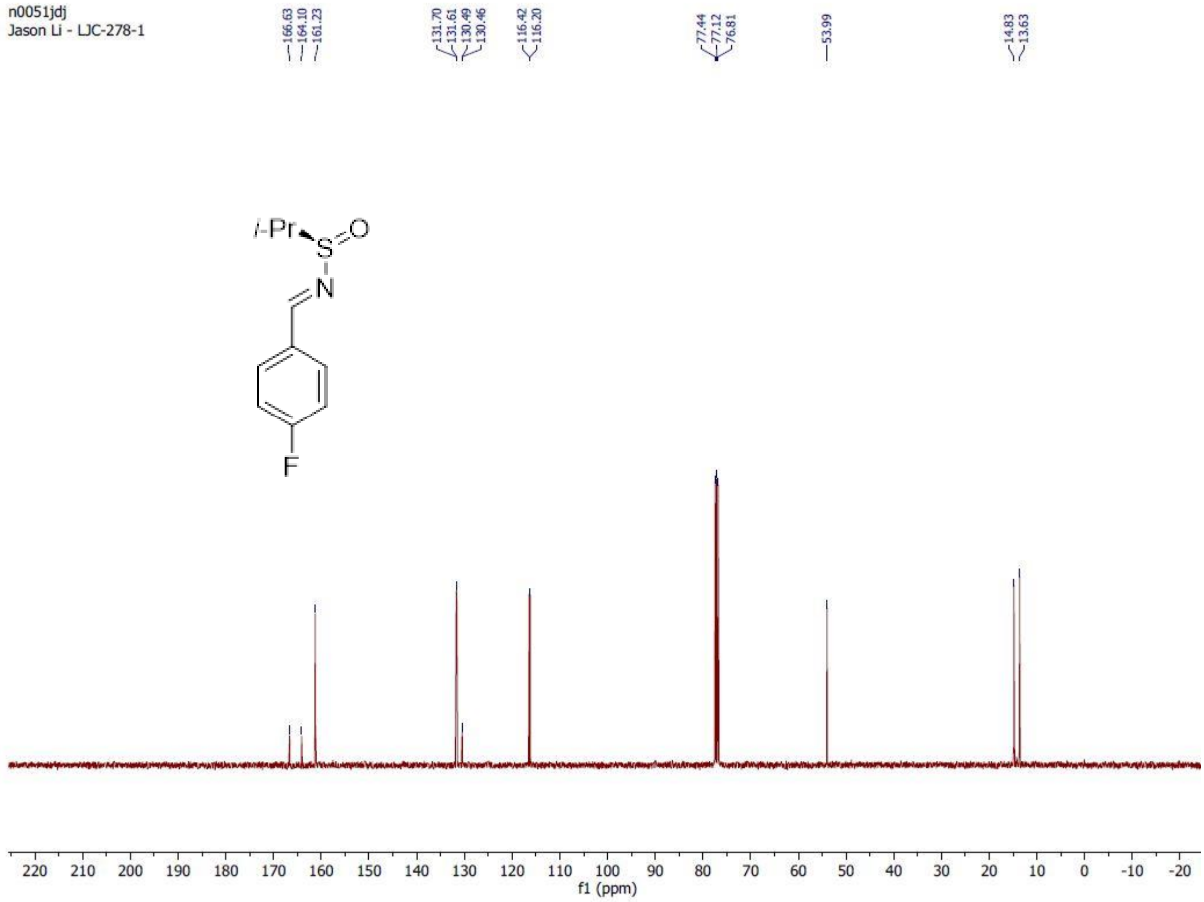

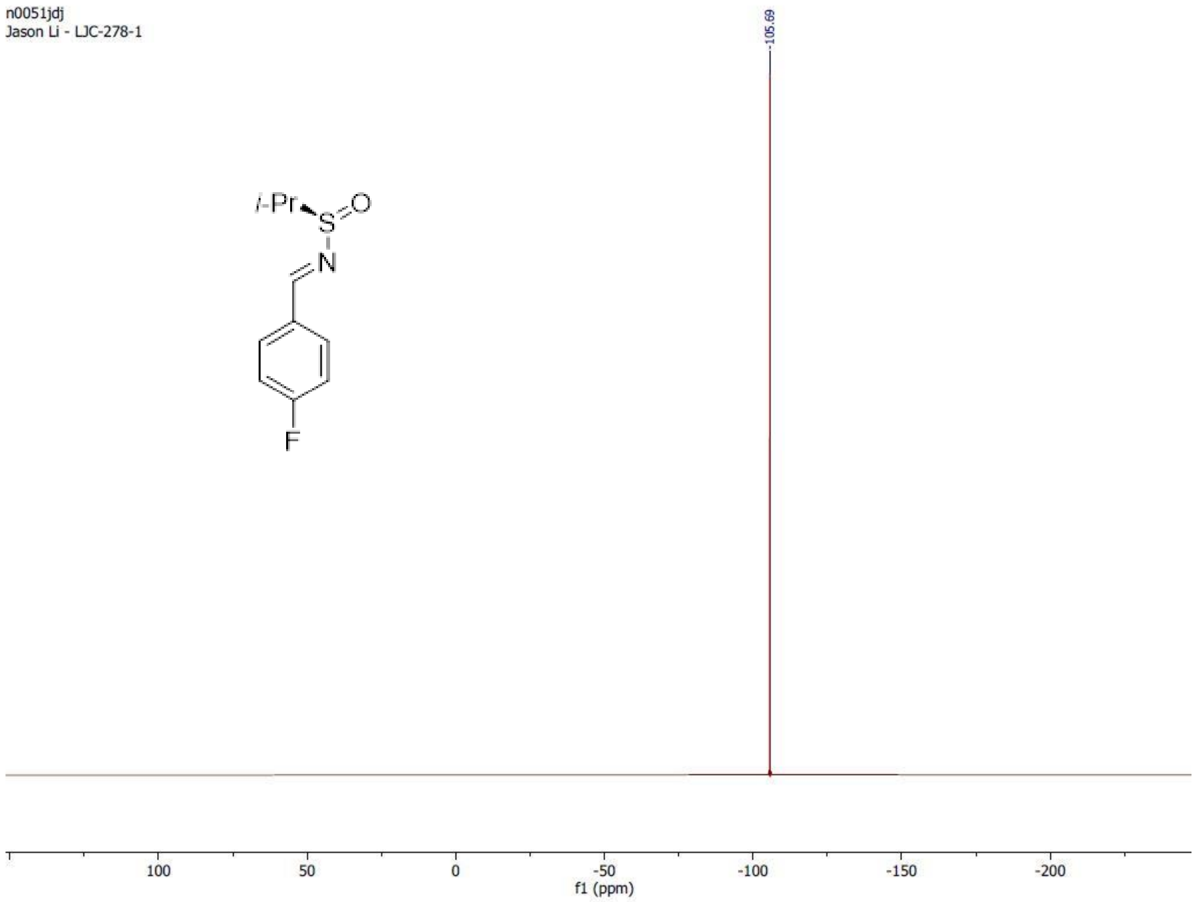
n0051jdj
Jason Li - LJC-278-1
-105.69
i-Pr
S
O
N
F
100
50
0
-50
-100
-150
-200
f1 (ppm)

Figure S142: NMR data for ***(R)*-12c**

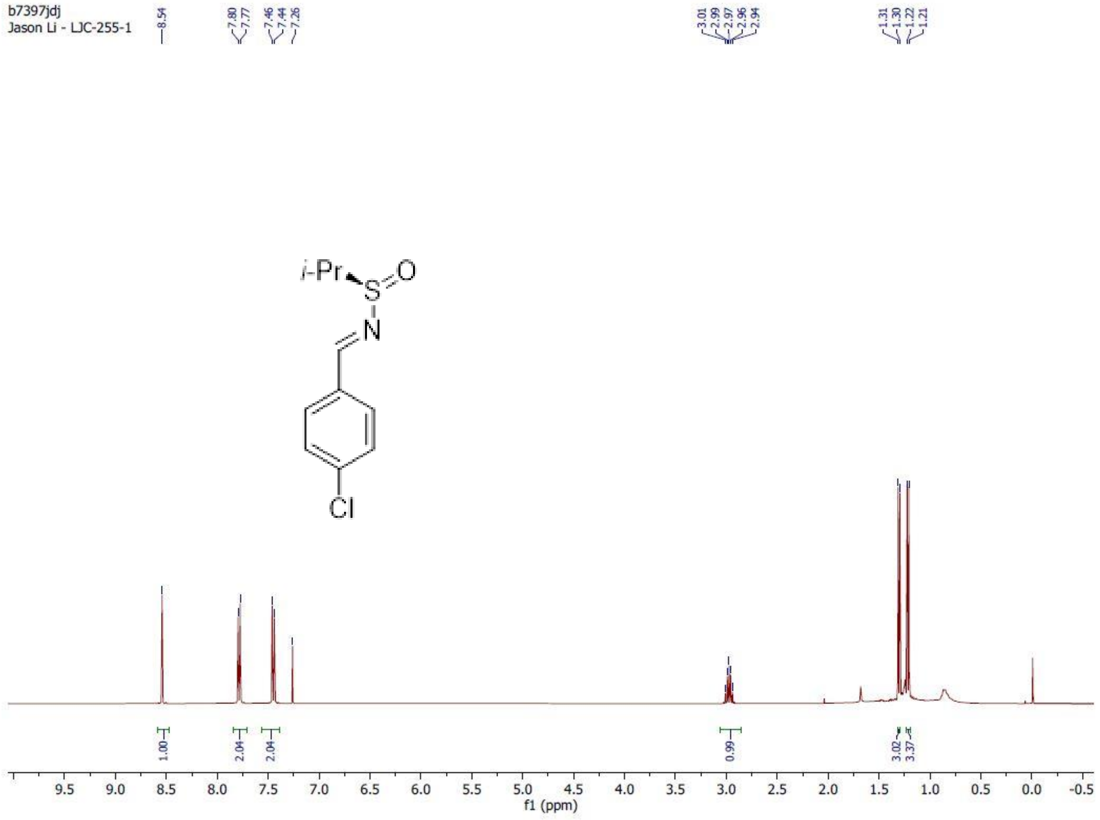

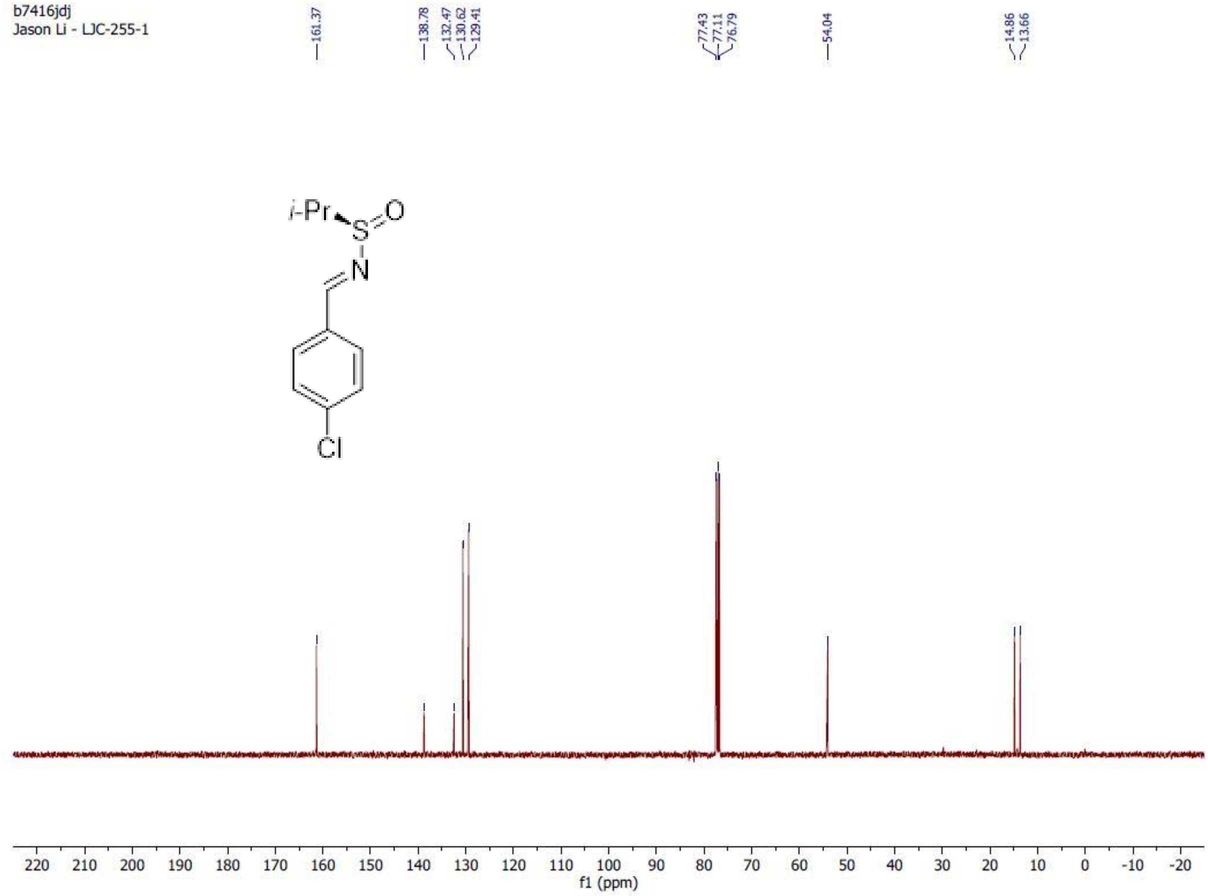

Figure S143: NMR data for **(*R*)-12d**

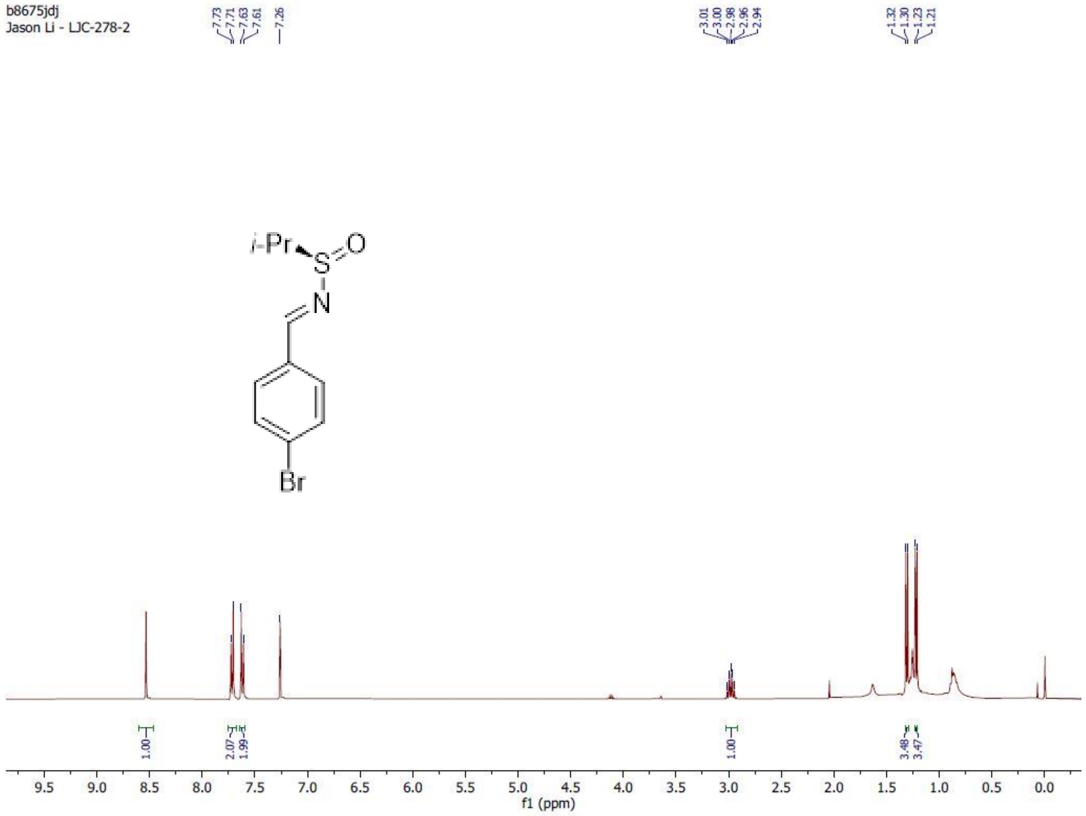

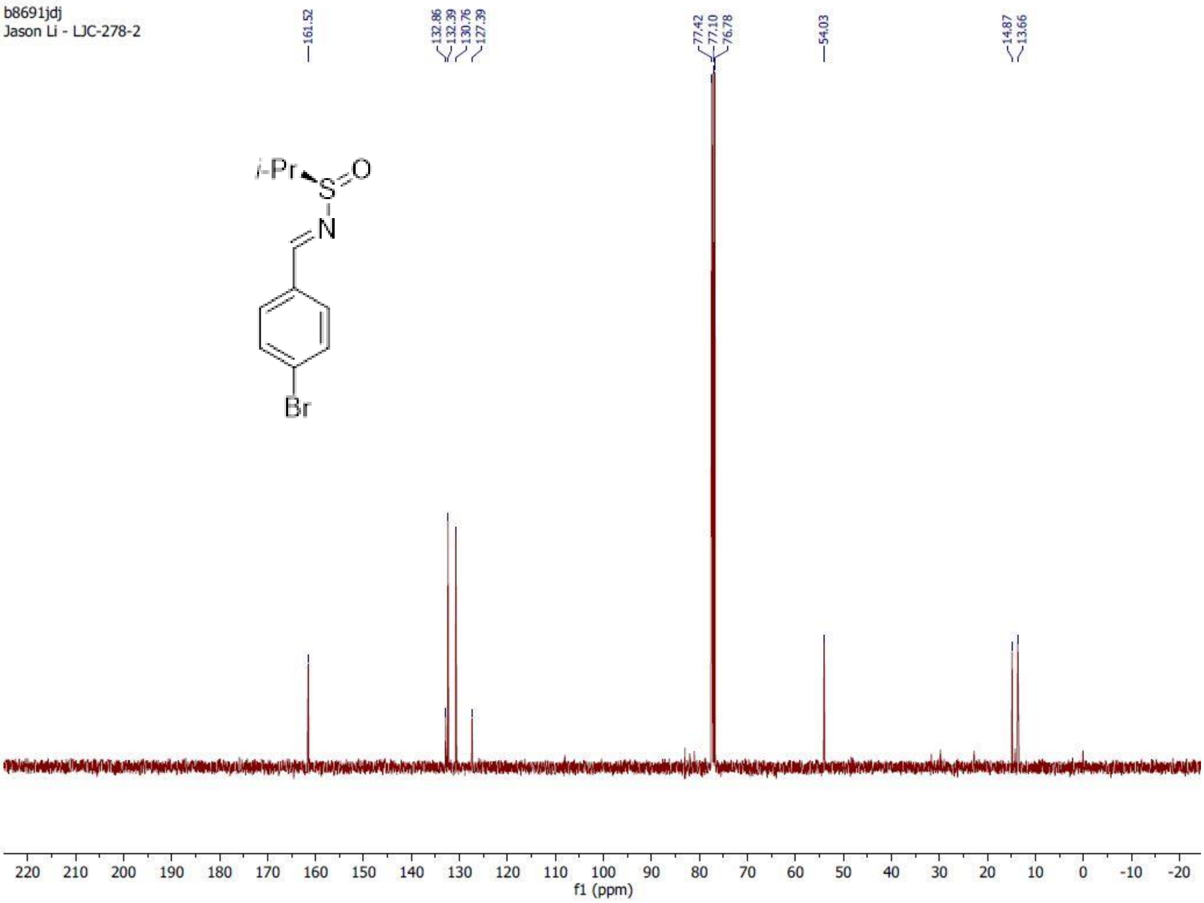

Figure S144: NMR data for **(*R*)-12e**

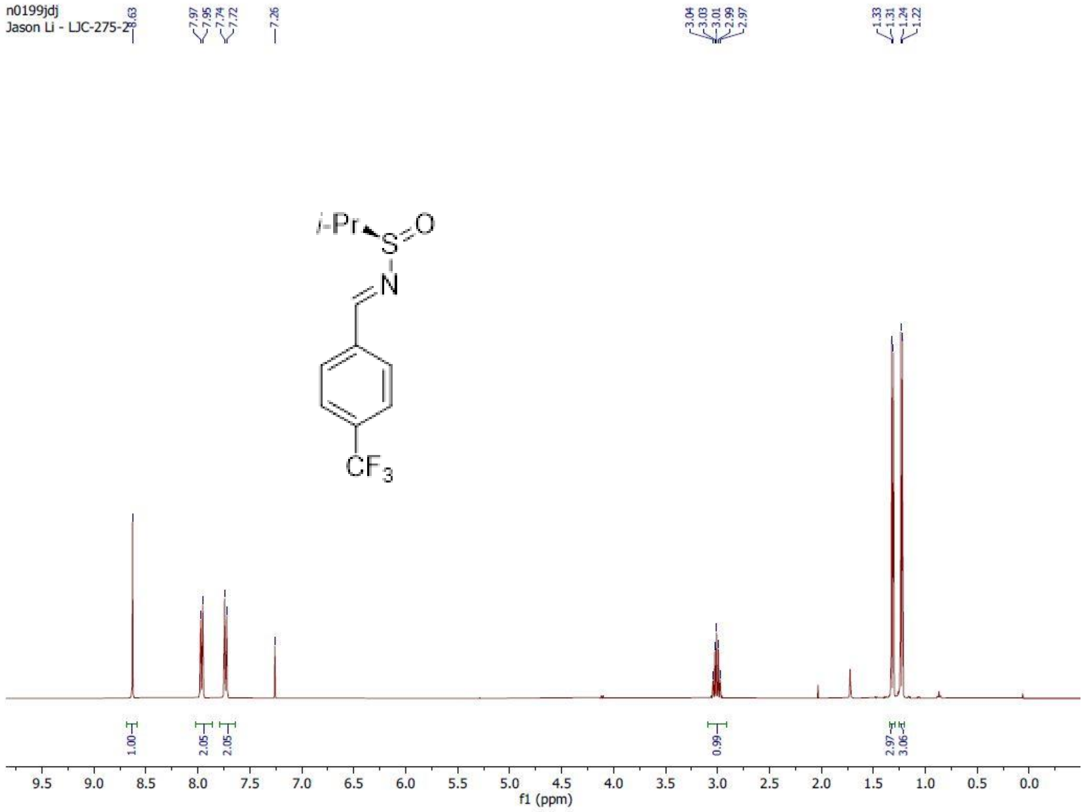

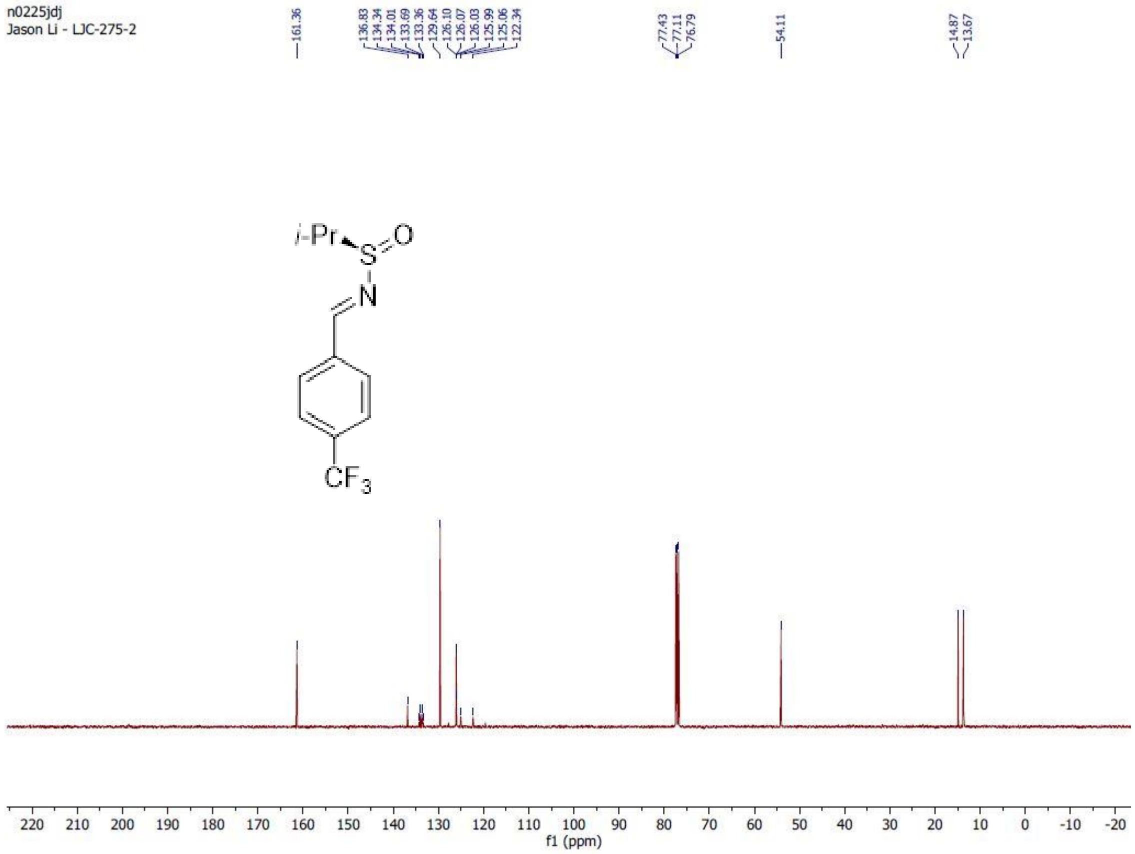

m9930jdj
Jason Li - LJC-275-2

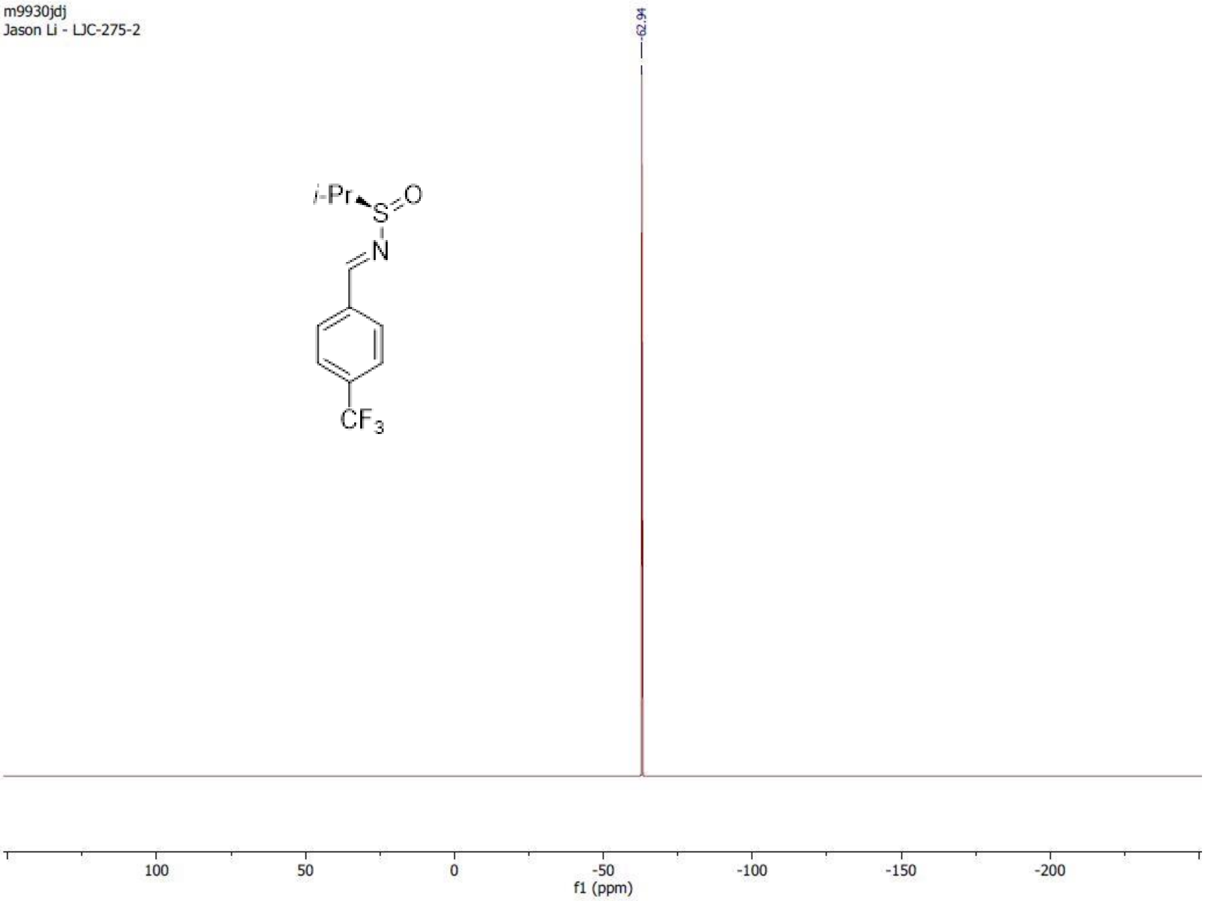

Figure S145: NMR data for **(*R*)-12f**

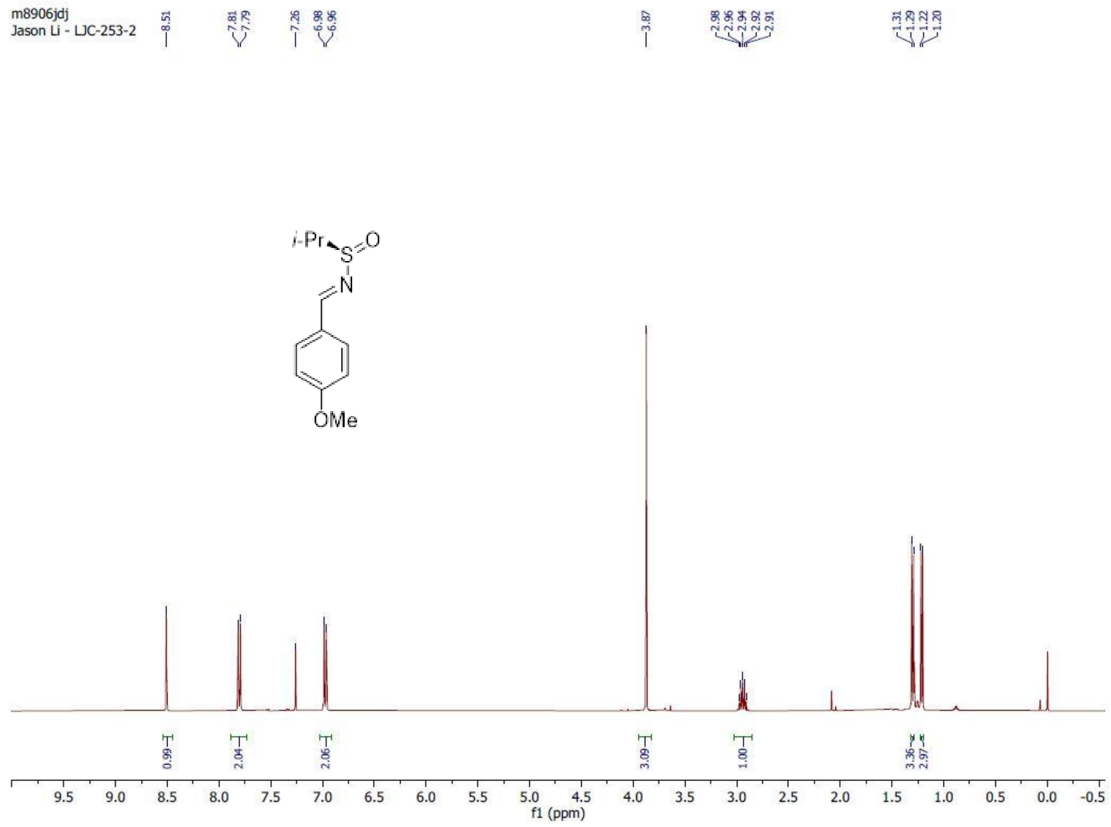

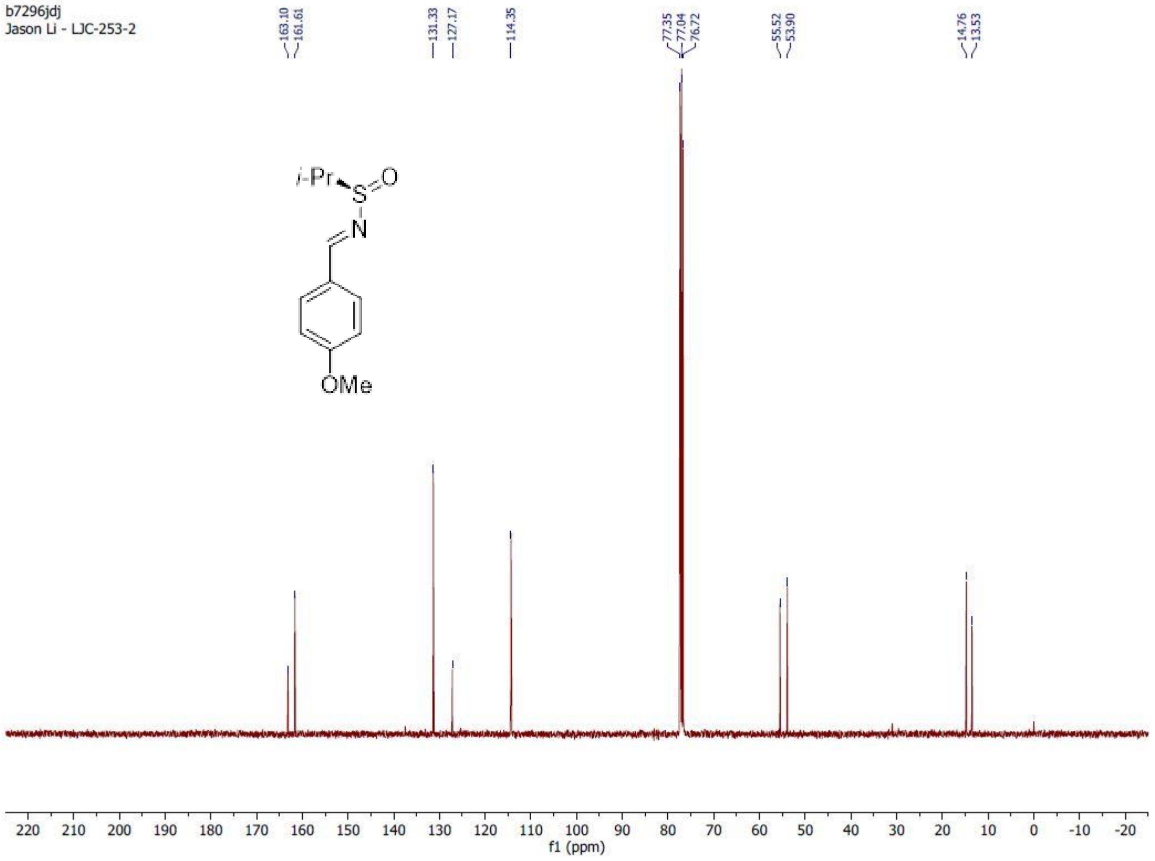

Figure S146: NMR data for **(*R*)-12g**

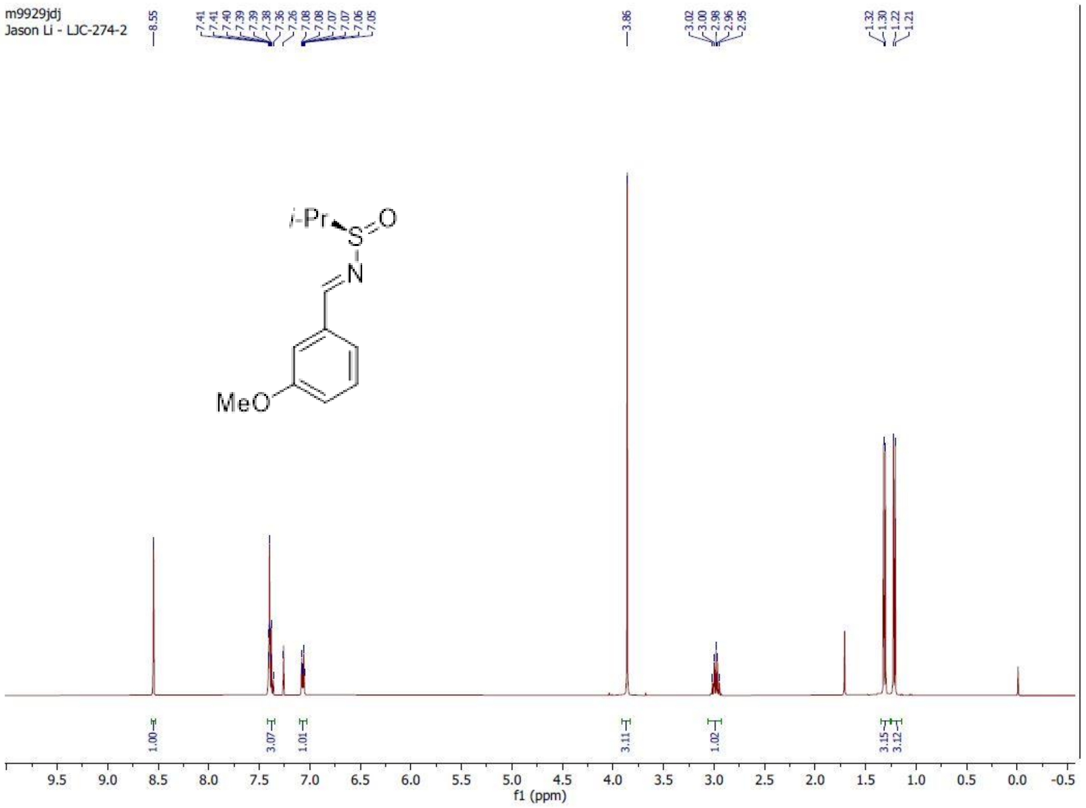

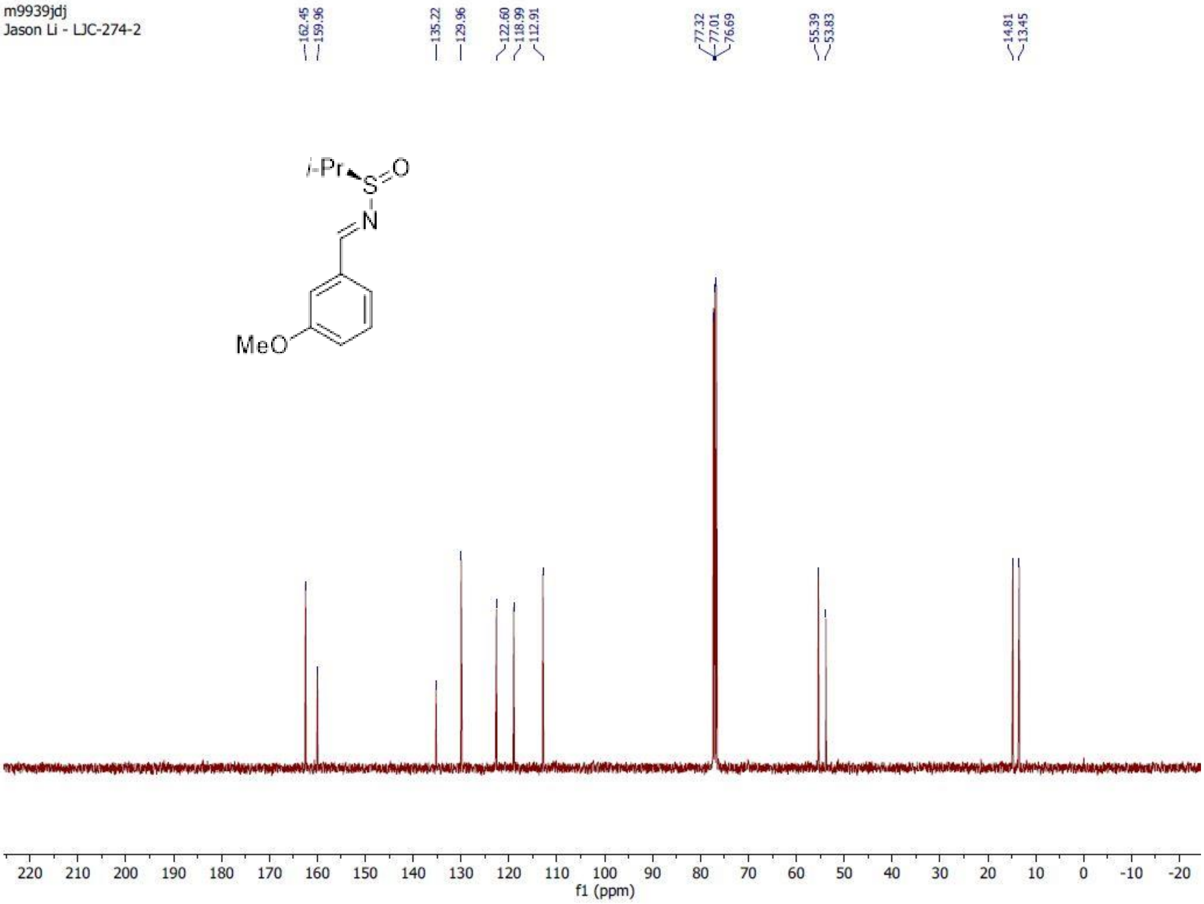

Figure S147: NMR data for **(*R*)-12h**

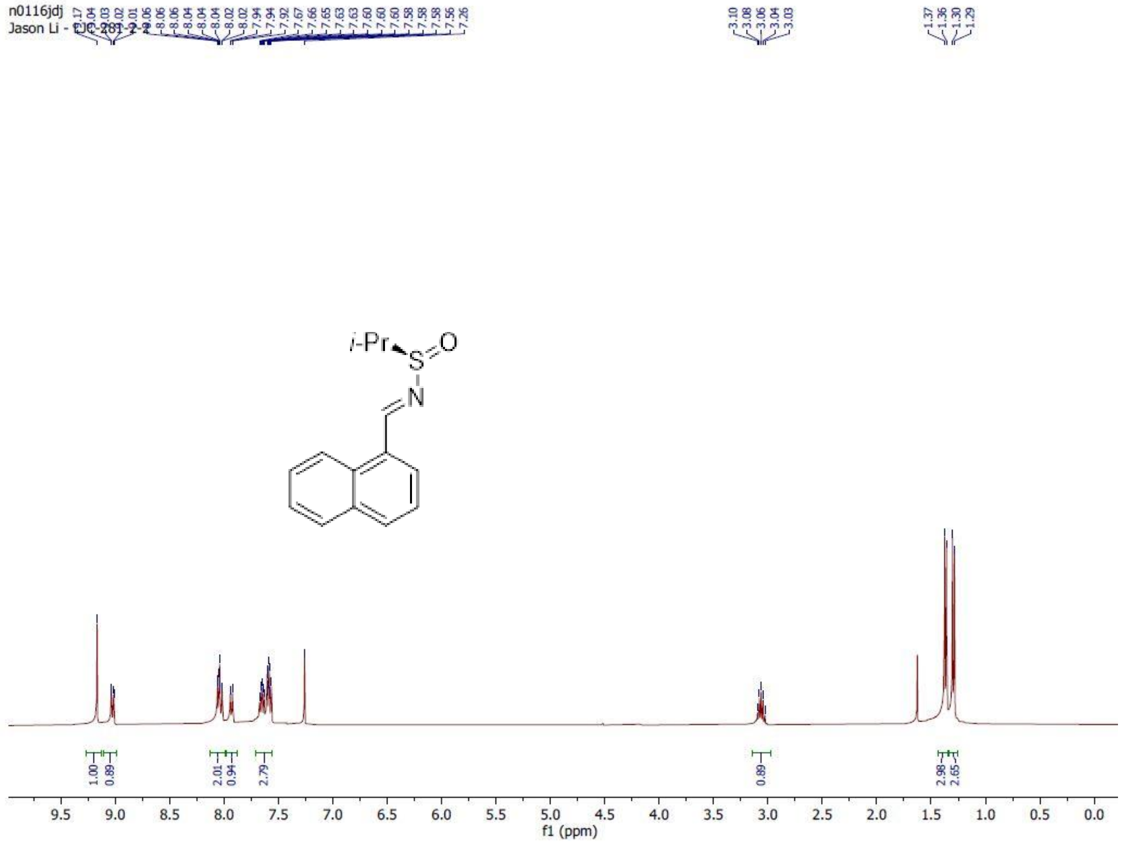

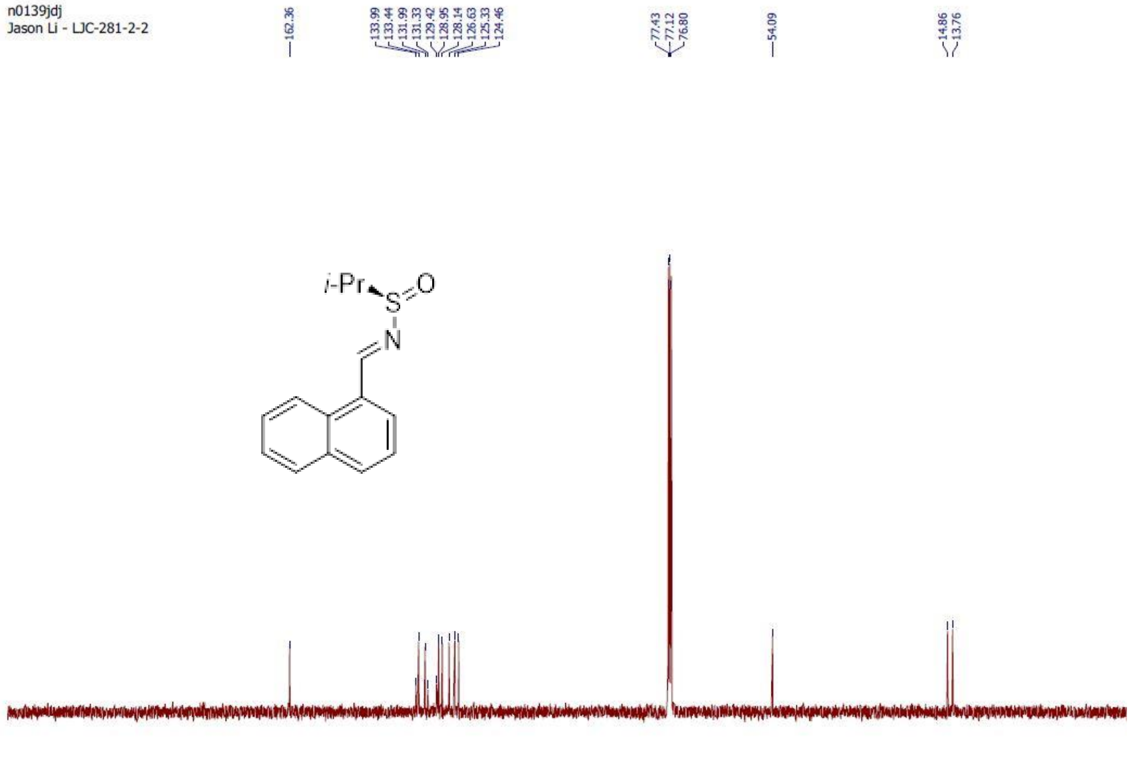

Figure S148: NMR data for ***(R)*-12i**

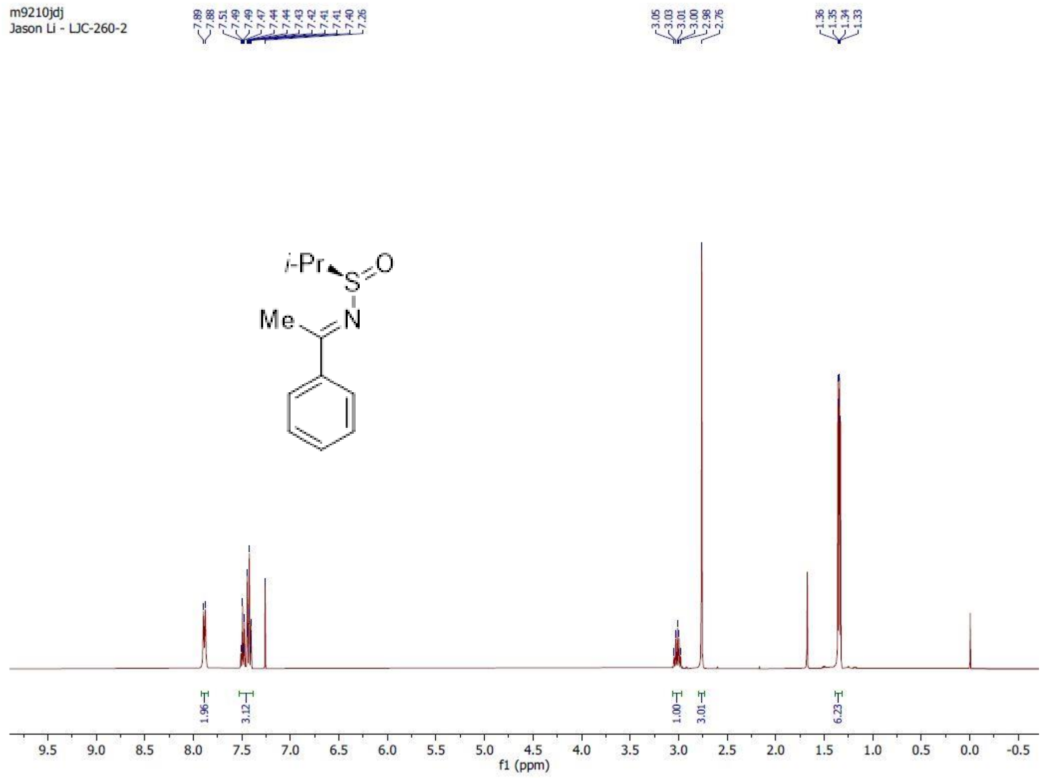

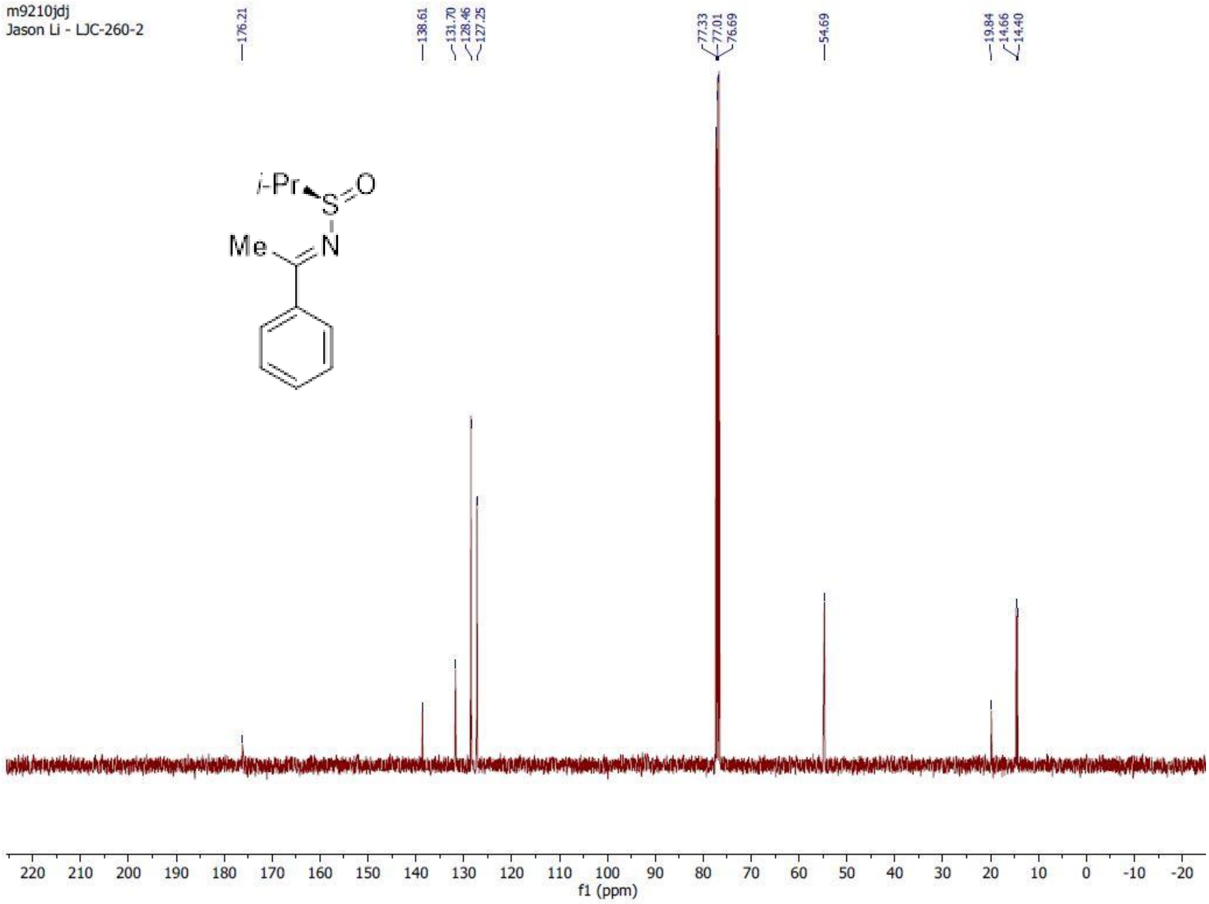

Figure S149: NMR data for ***(R)*-12j**

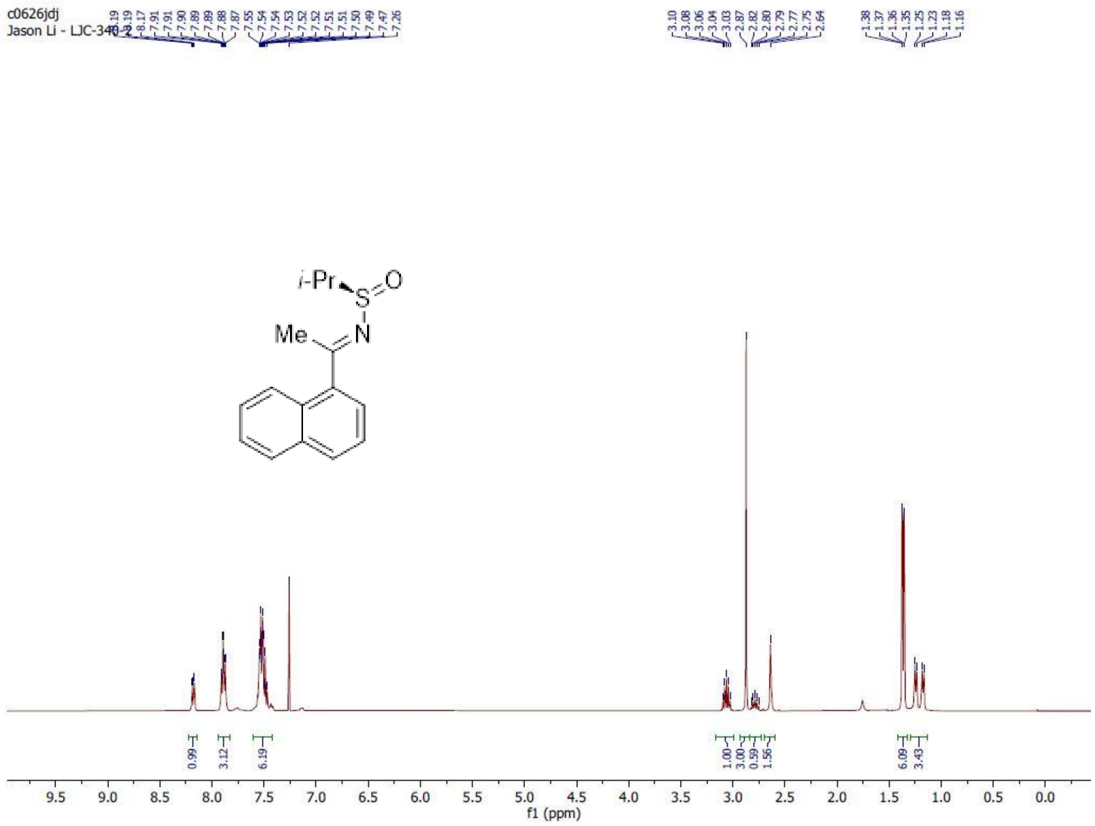

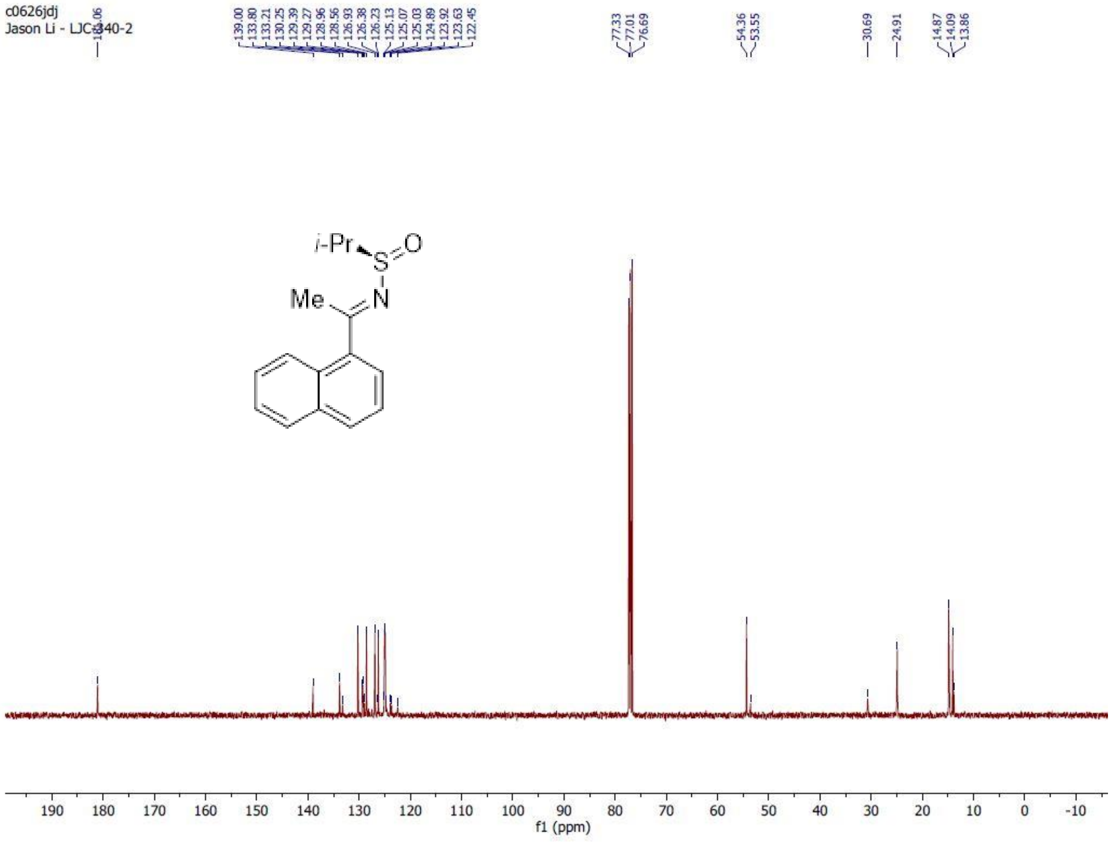

Figure S150: NMR data for **(*R*)-12k**

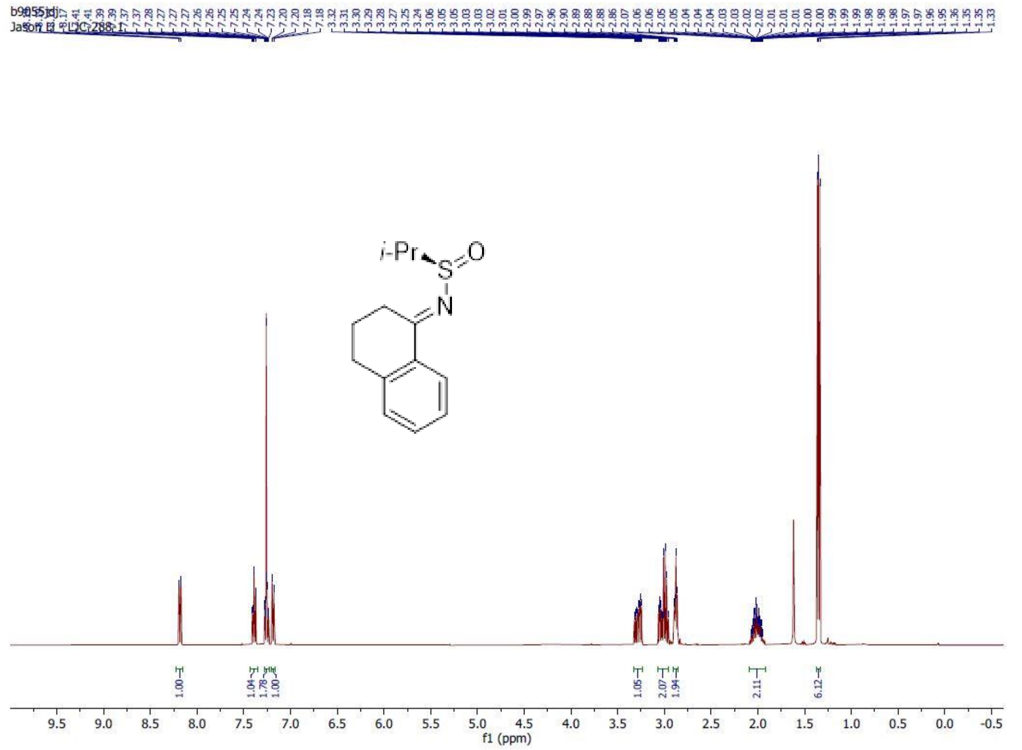

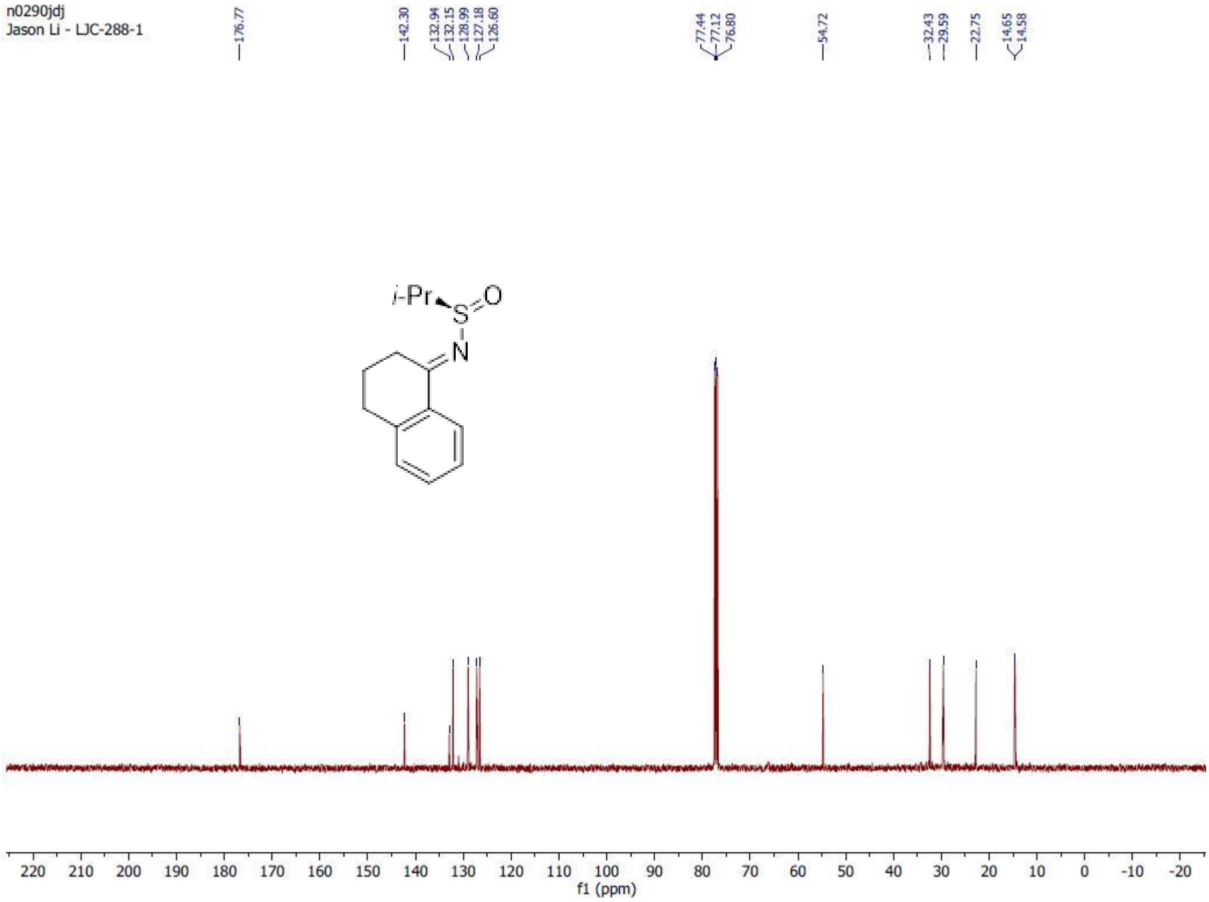

Figure S151: NMR data for **(*R*)-12l**

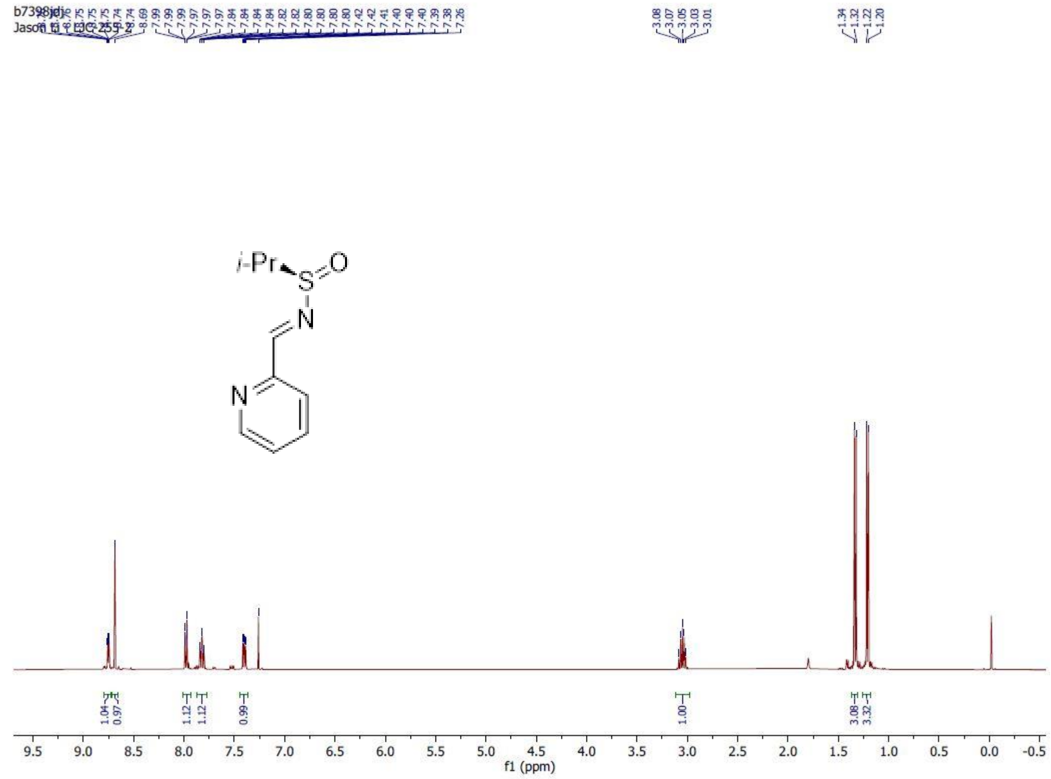

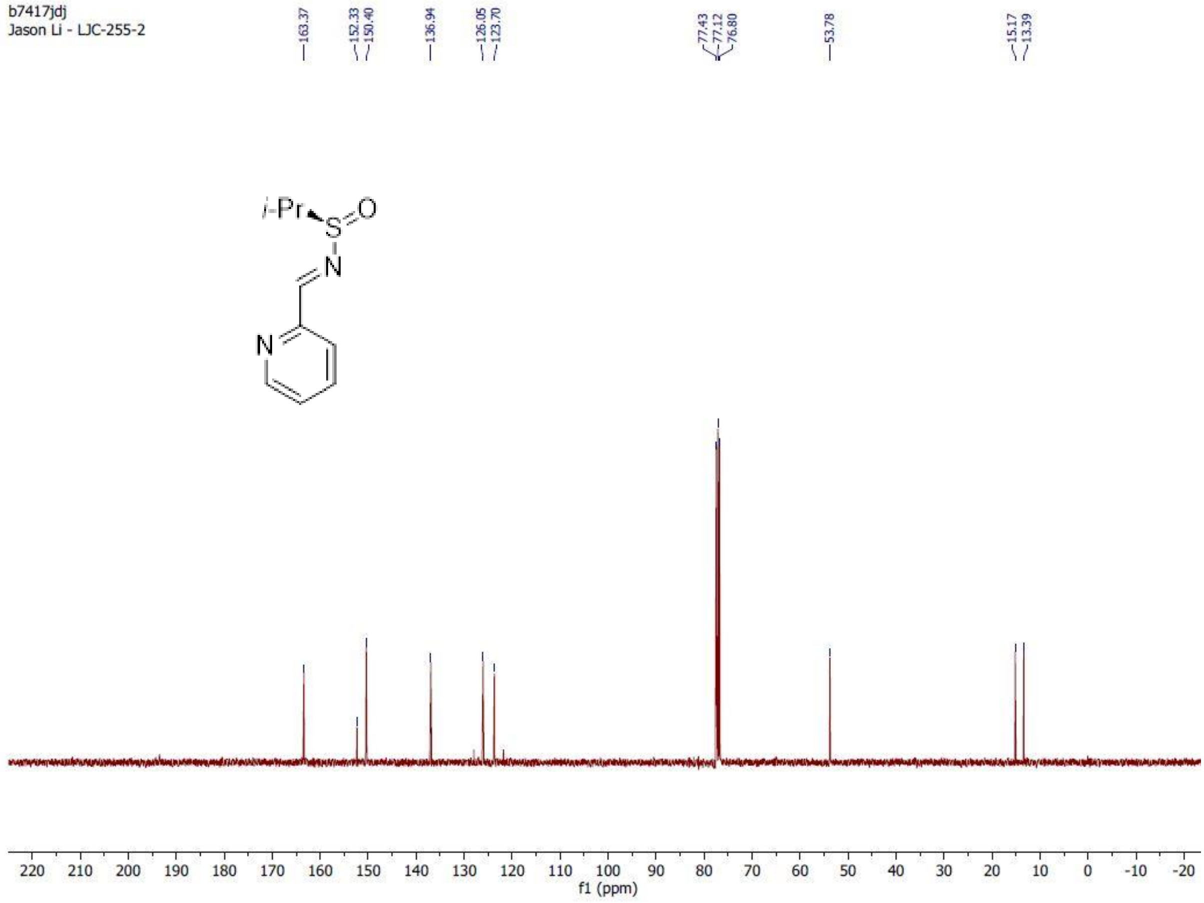

Figure S152: NMR data for **(*S*)-13a**

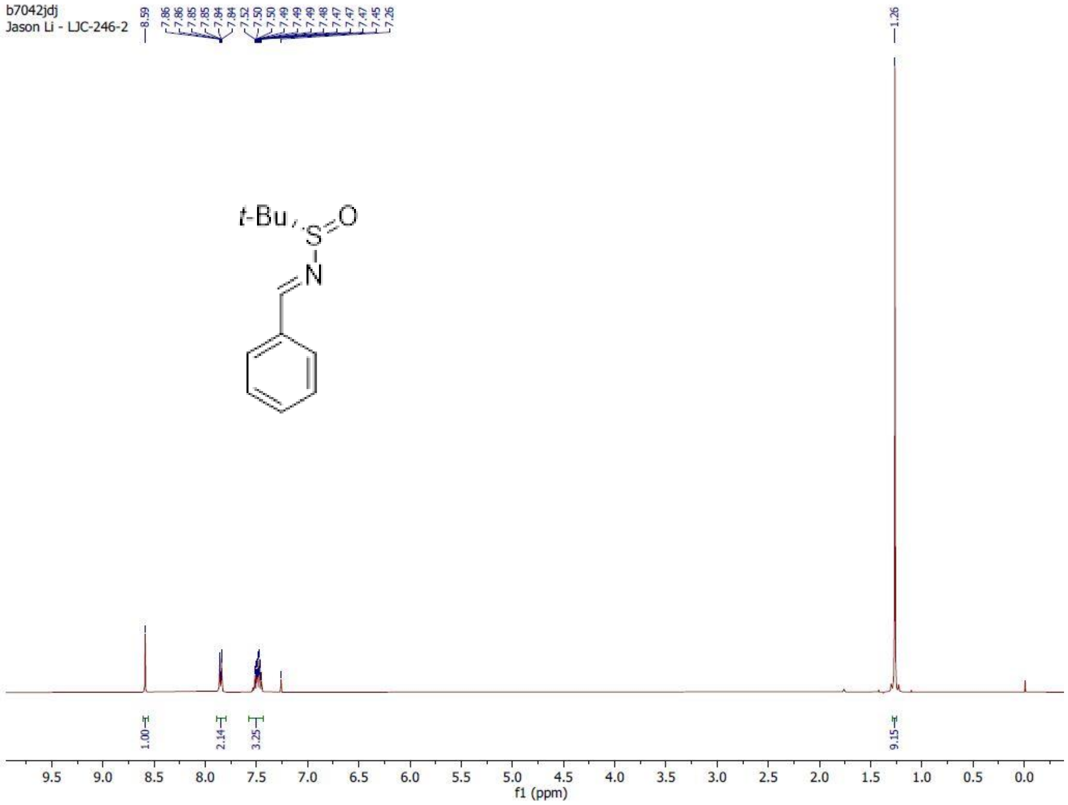

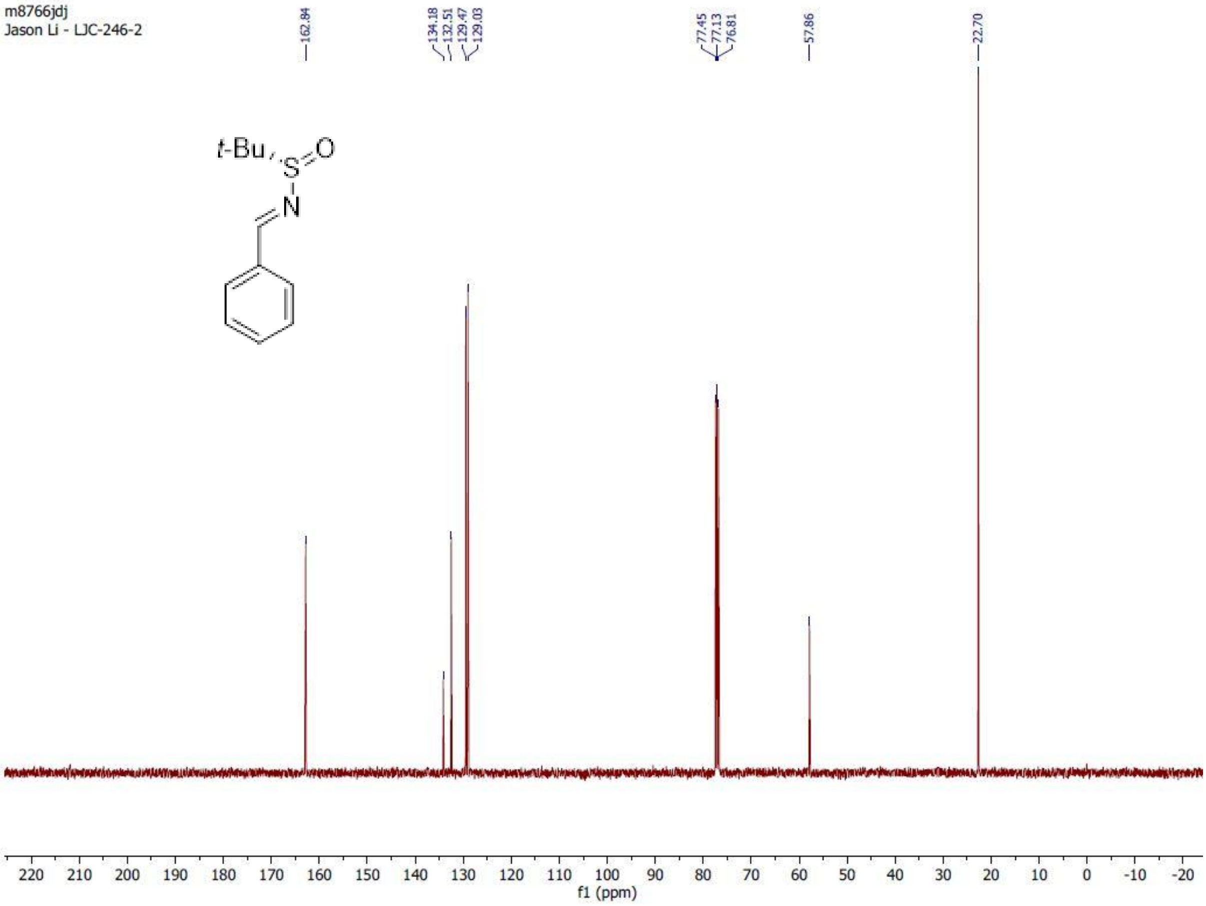

Figure S153: NMR data for **(*S*)-13b**

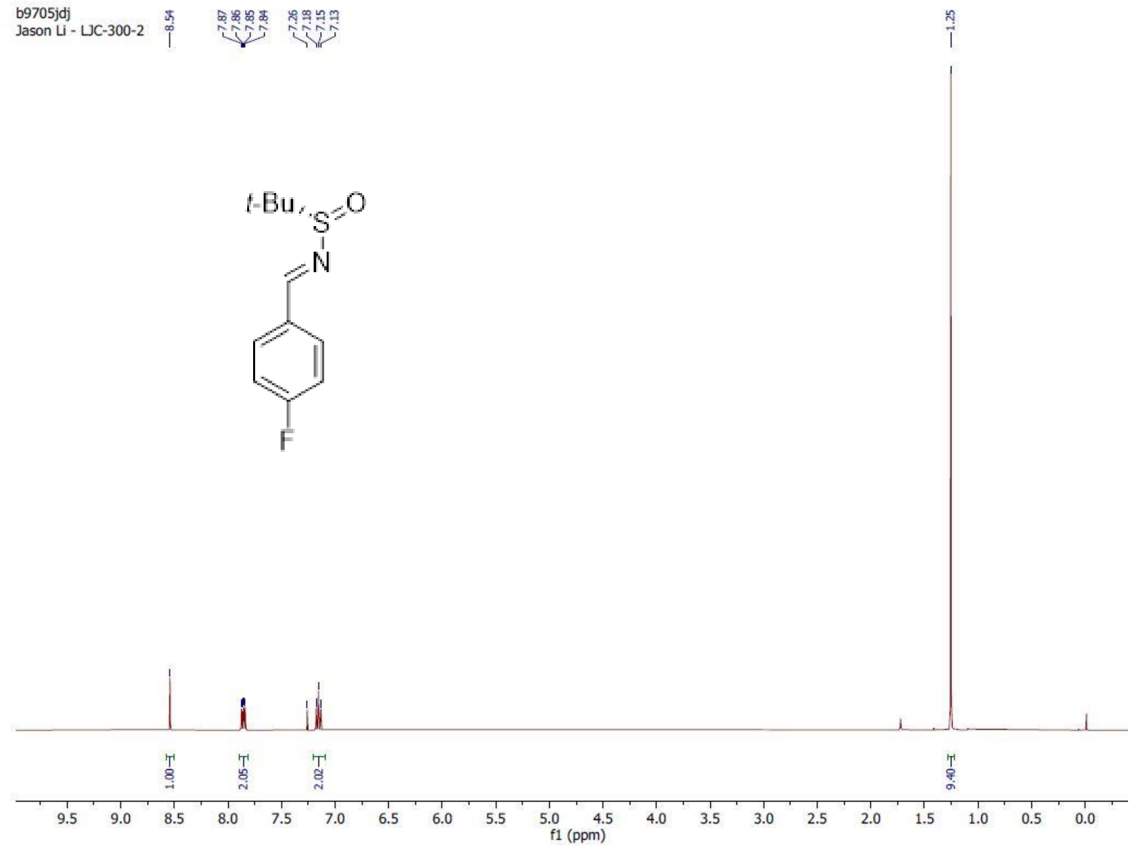

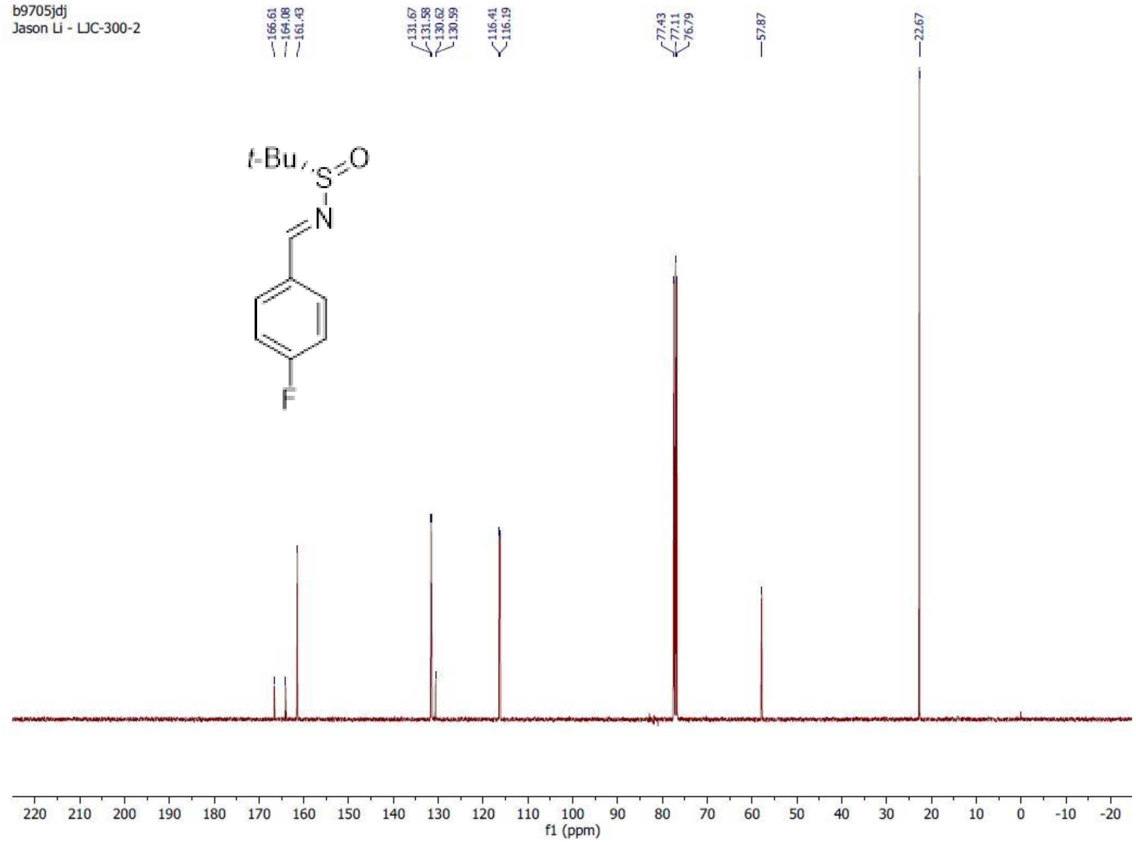

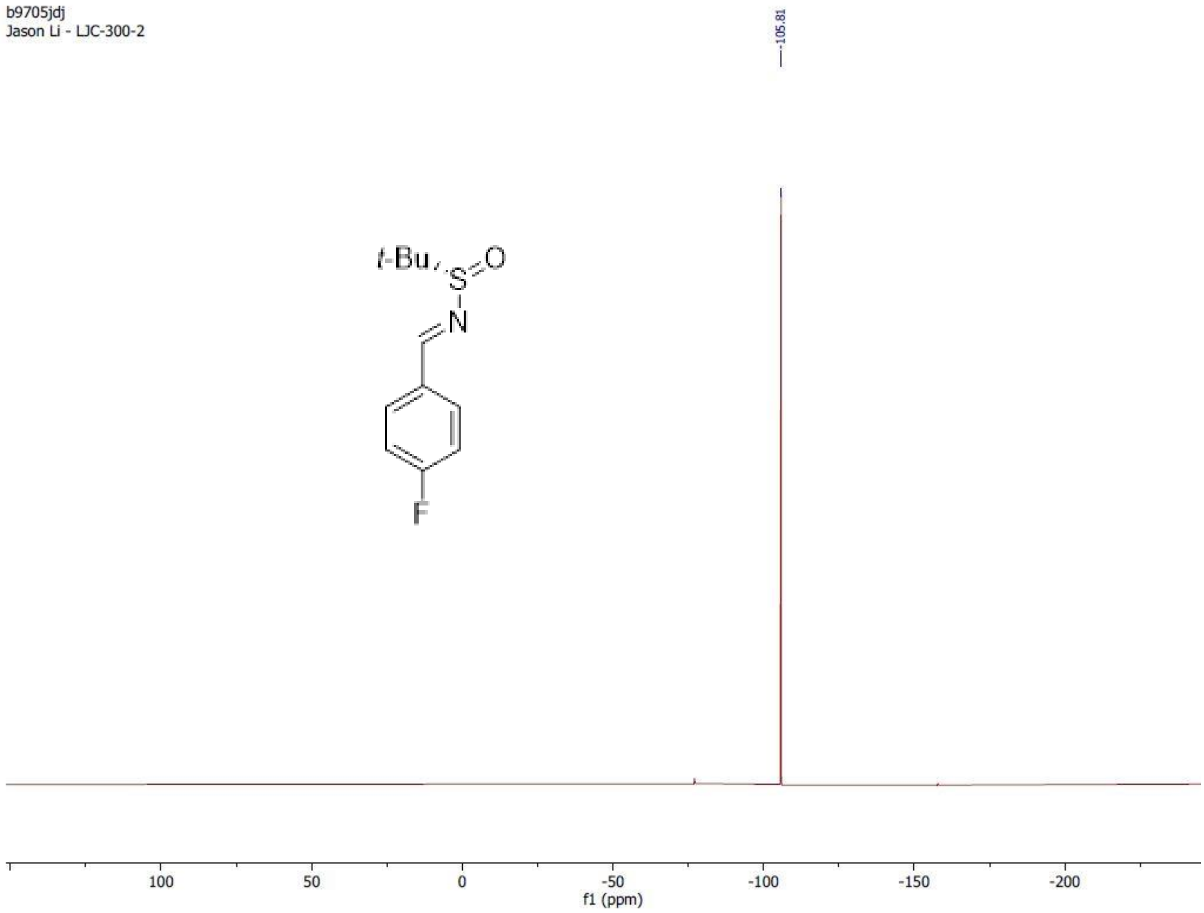
b9705jdj
Jason Li - LJC-300-2
-105.81
t-Bu
S
O
N
F
100
50
0
-50
-100
-150
-200
f1 (ppm)

Figure S154: NMR data for **(*S*)-13c**

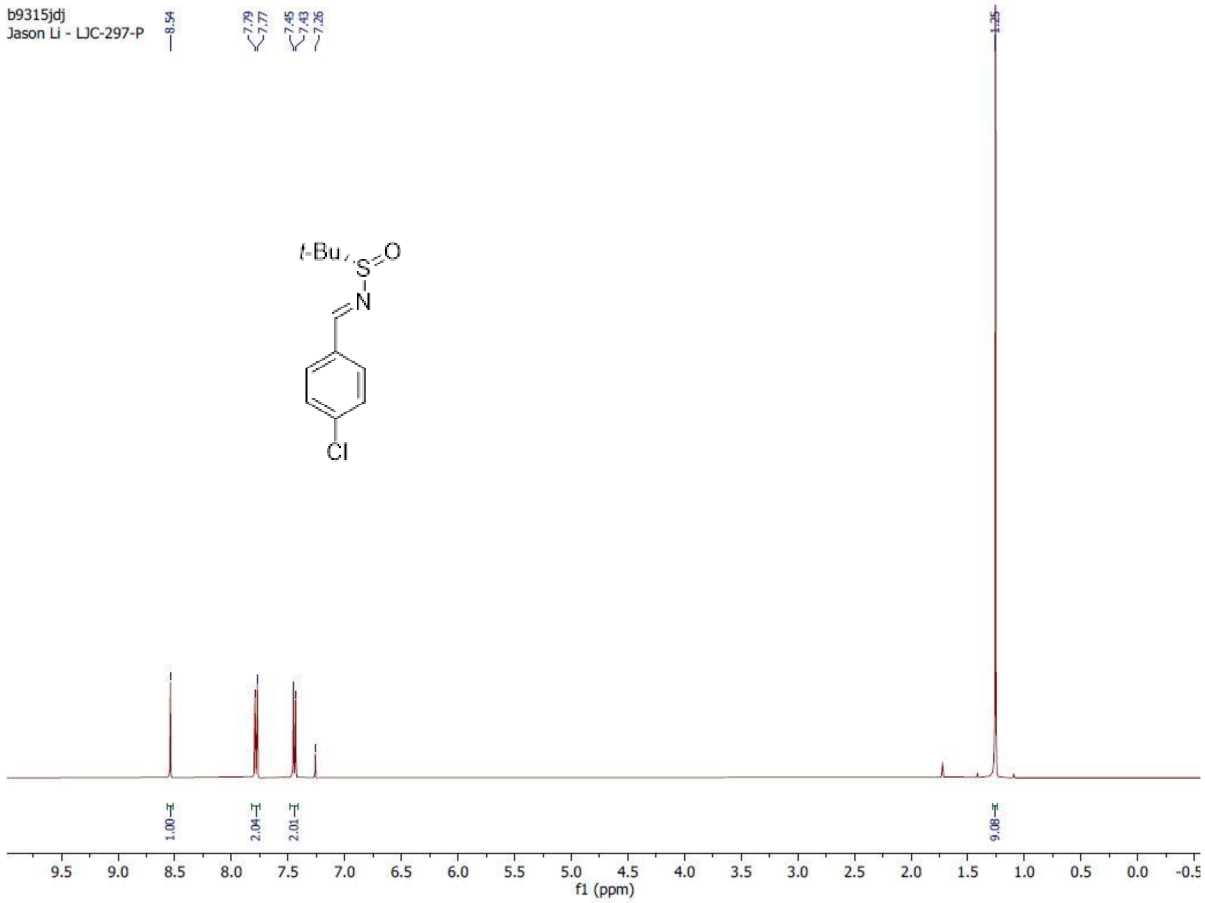

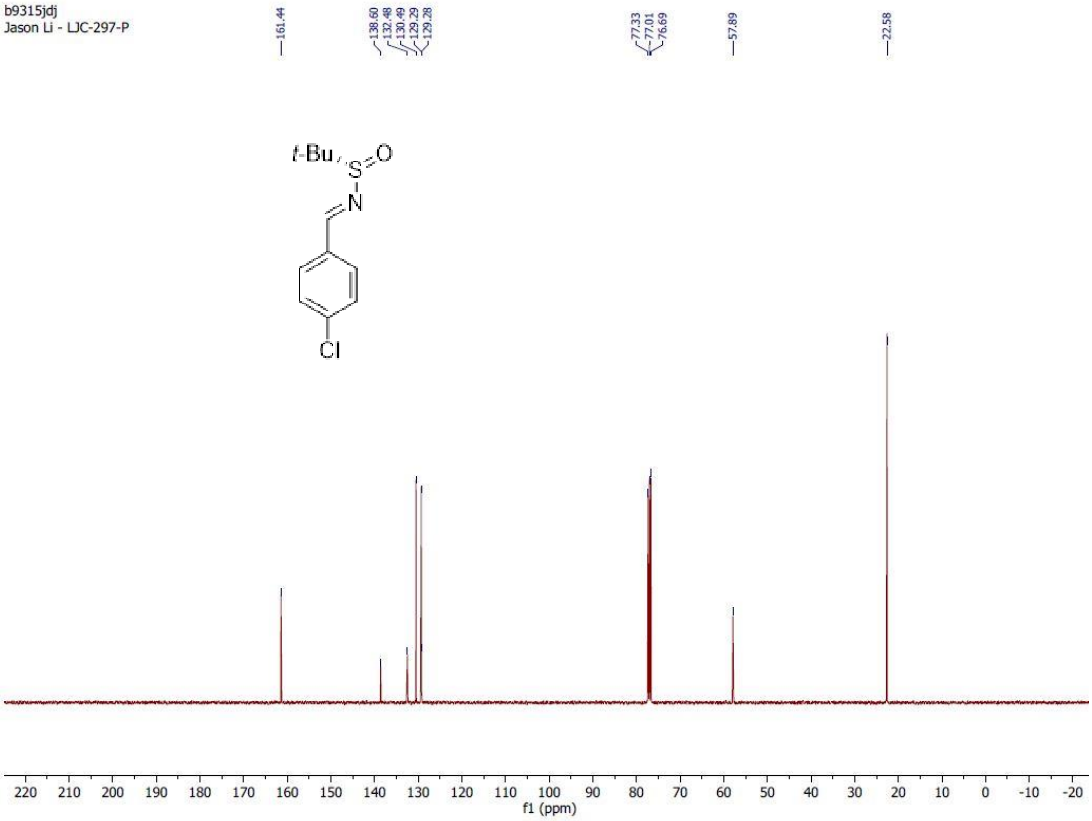

Figure S155: NMR data for **(*S*)-13d**

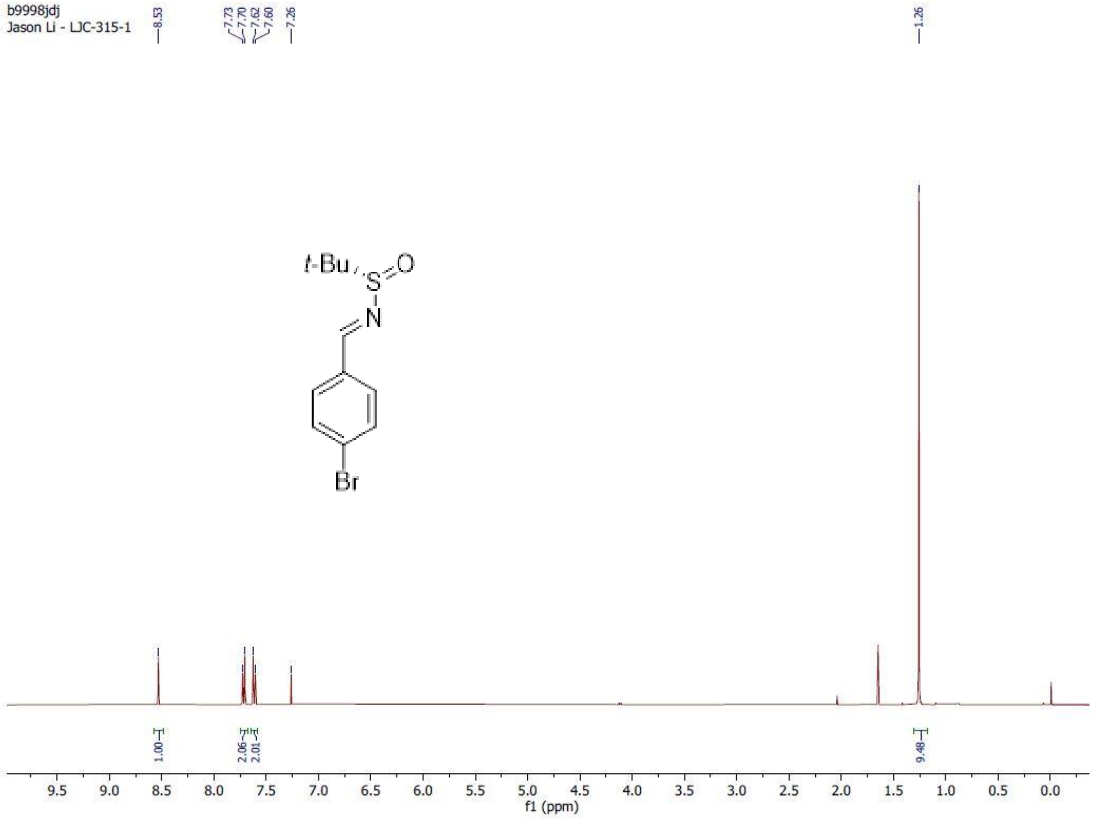

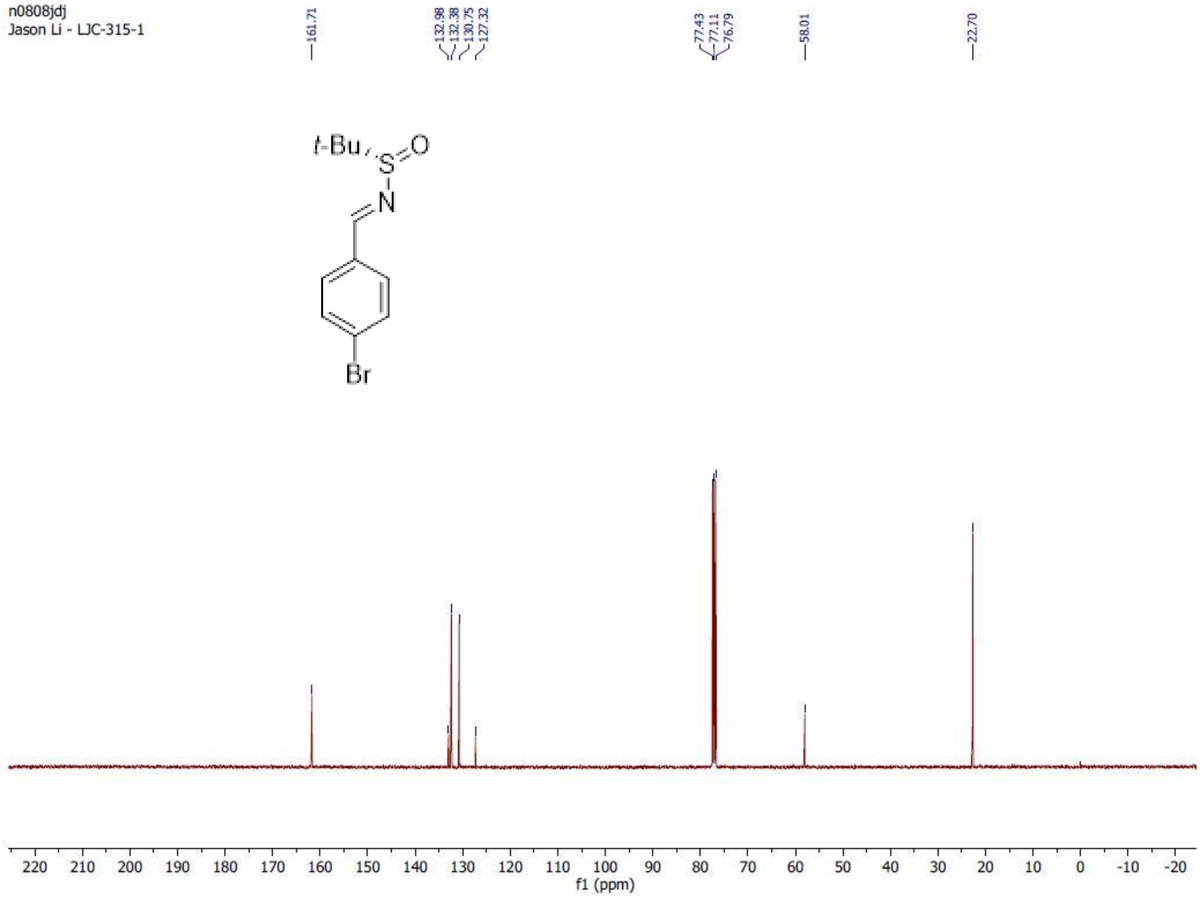

Figure S156: NMR data for **(*S*)-13e**

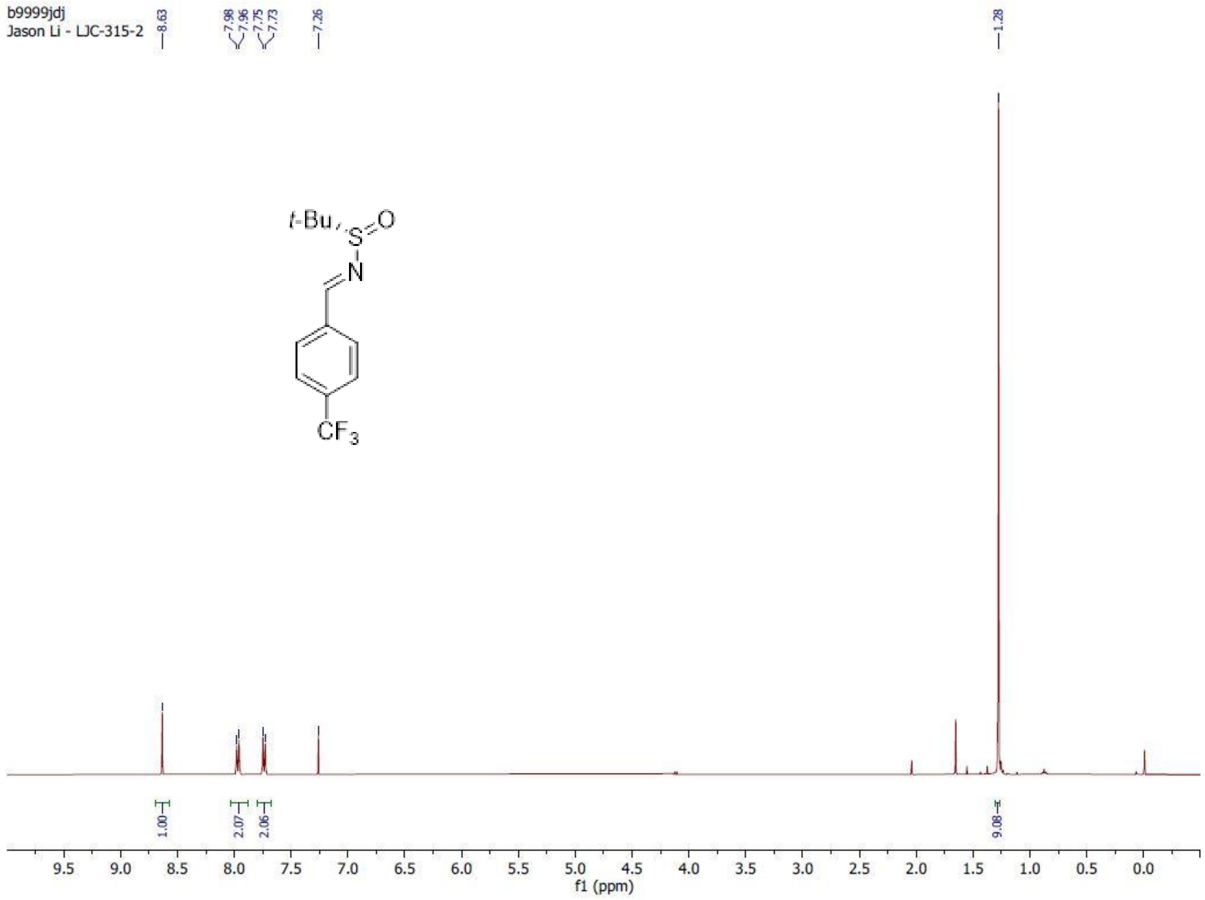

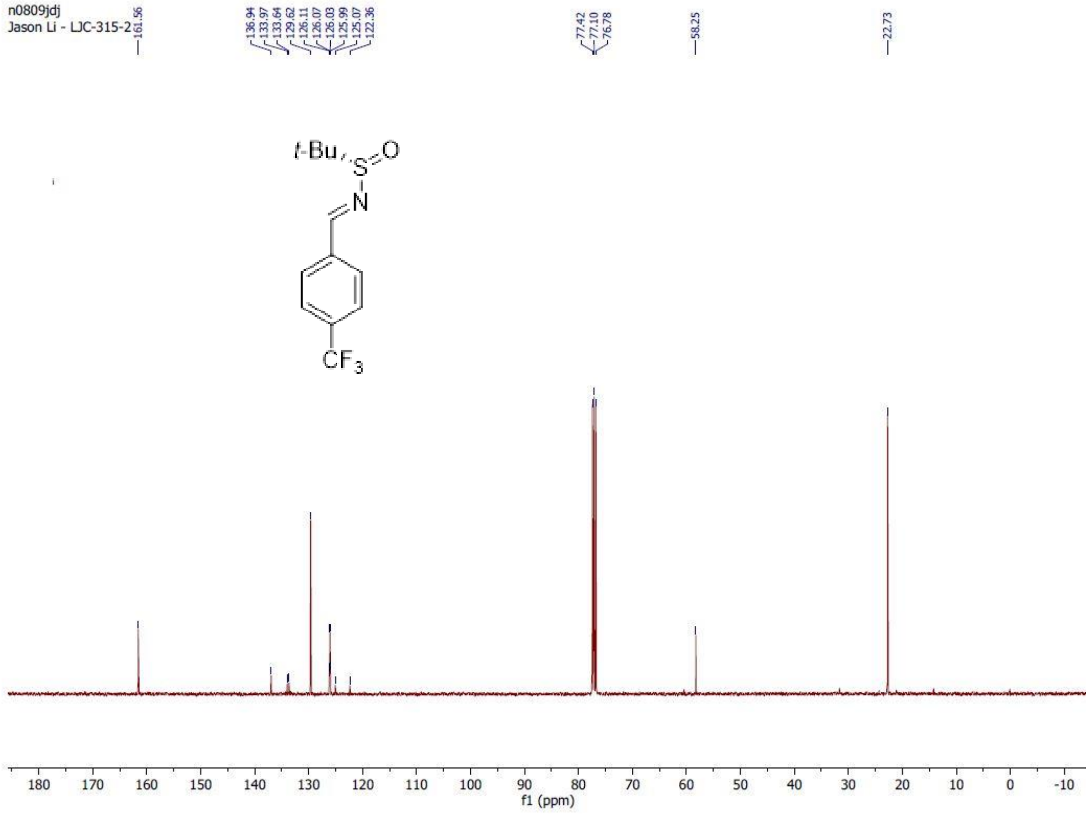

n0809jdj
Jason Li - LJC-315-2

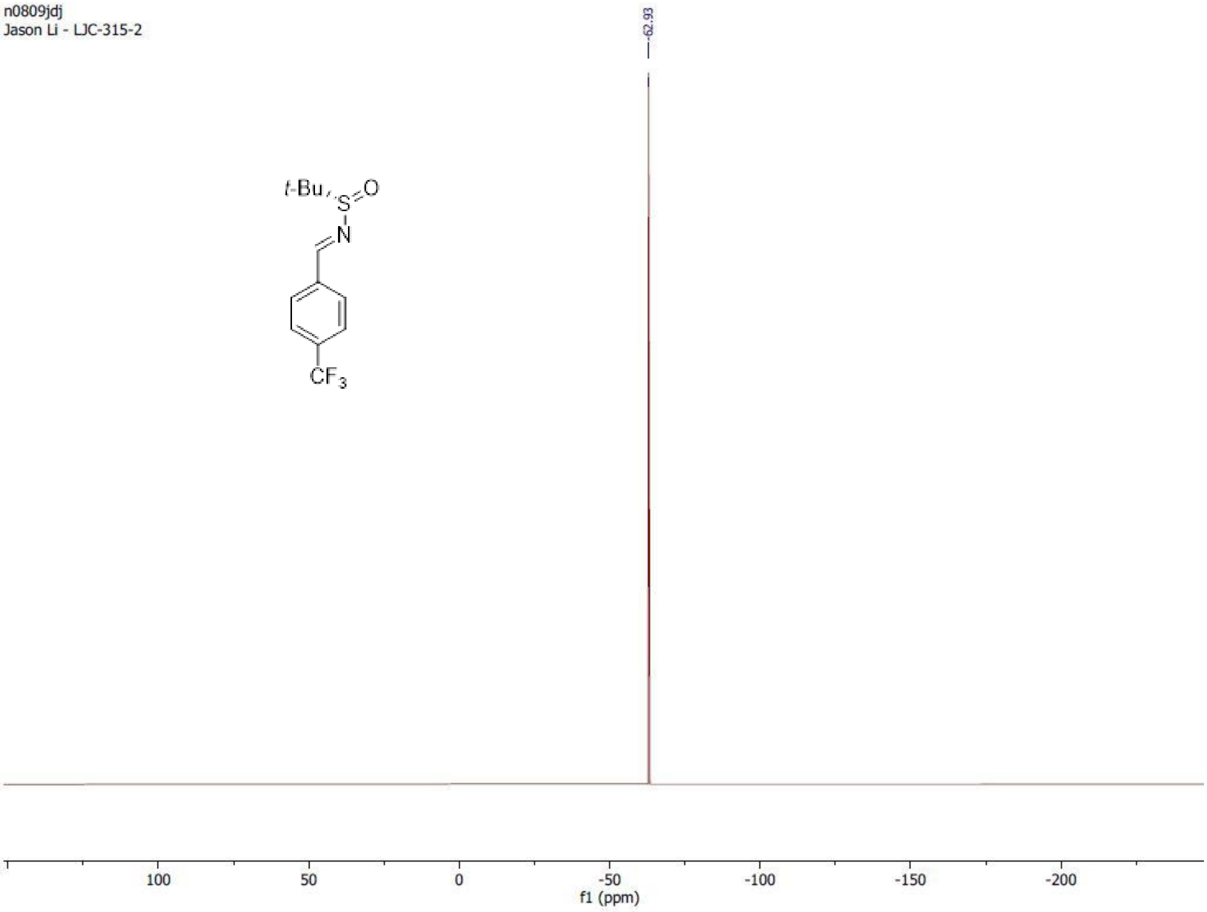

Figure S157: NMR data for **(*S*)-13f**

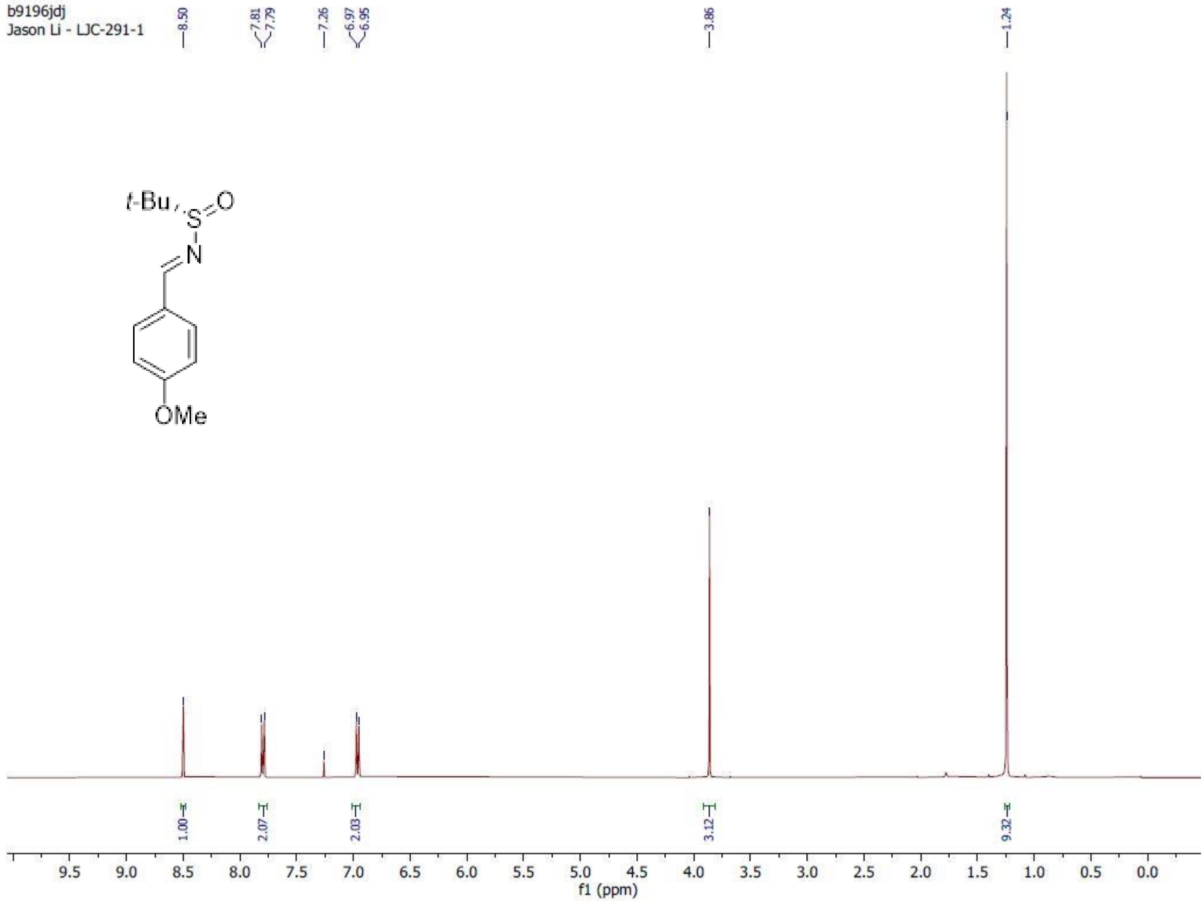

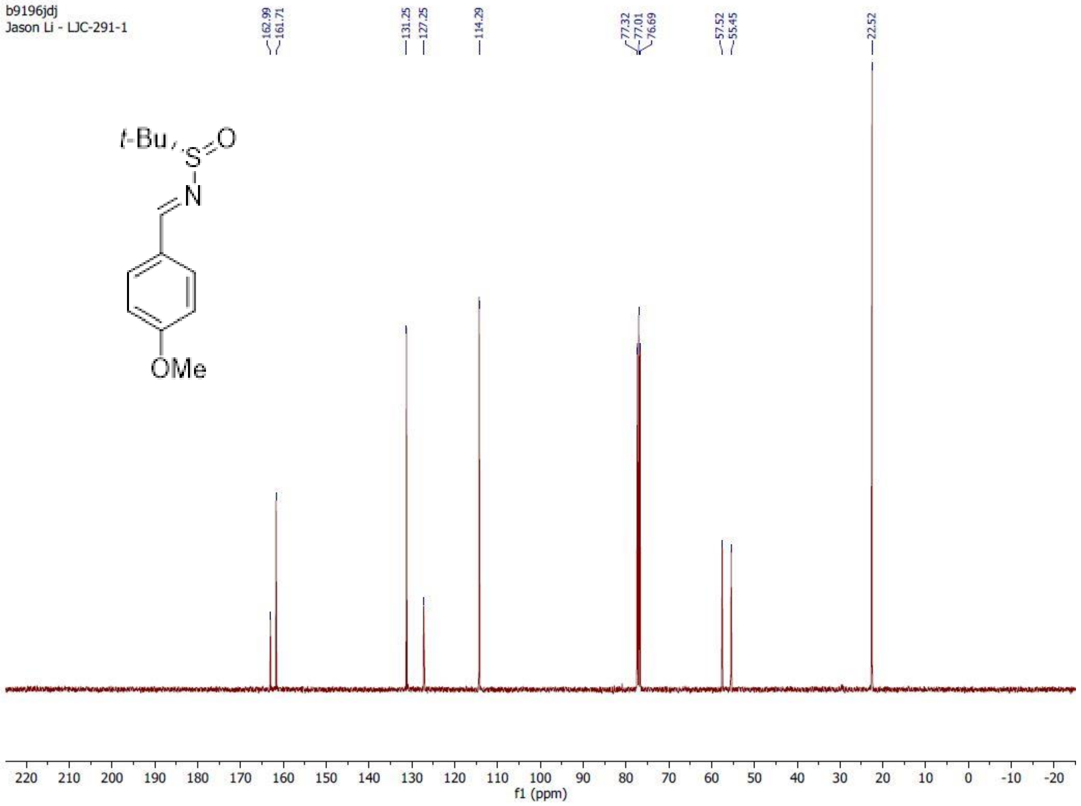

Figure S158: NMR data for **(*S*)-13g**

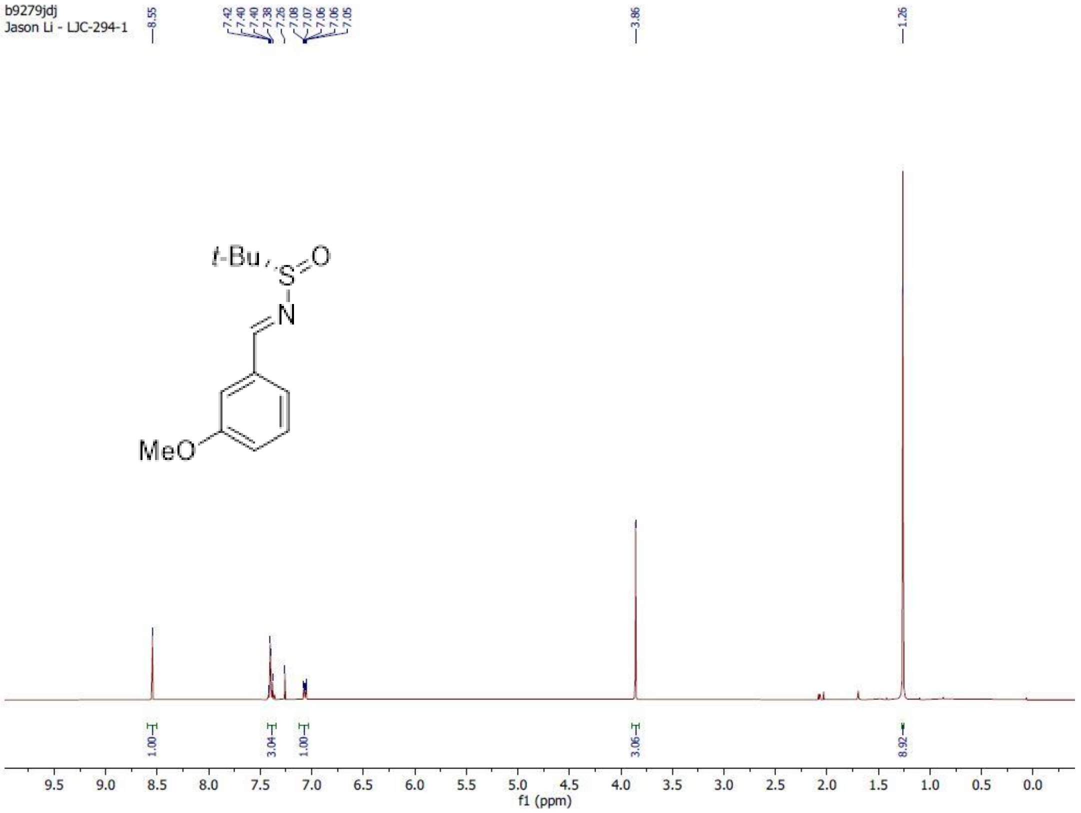

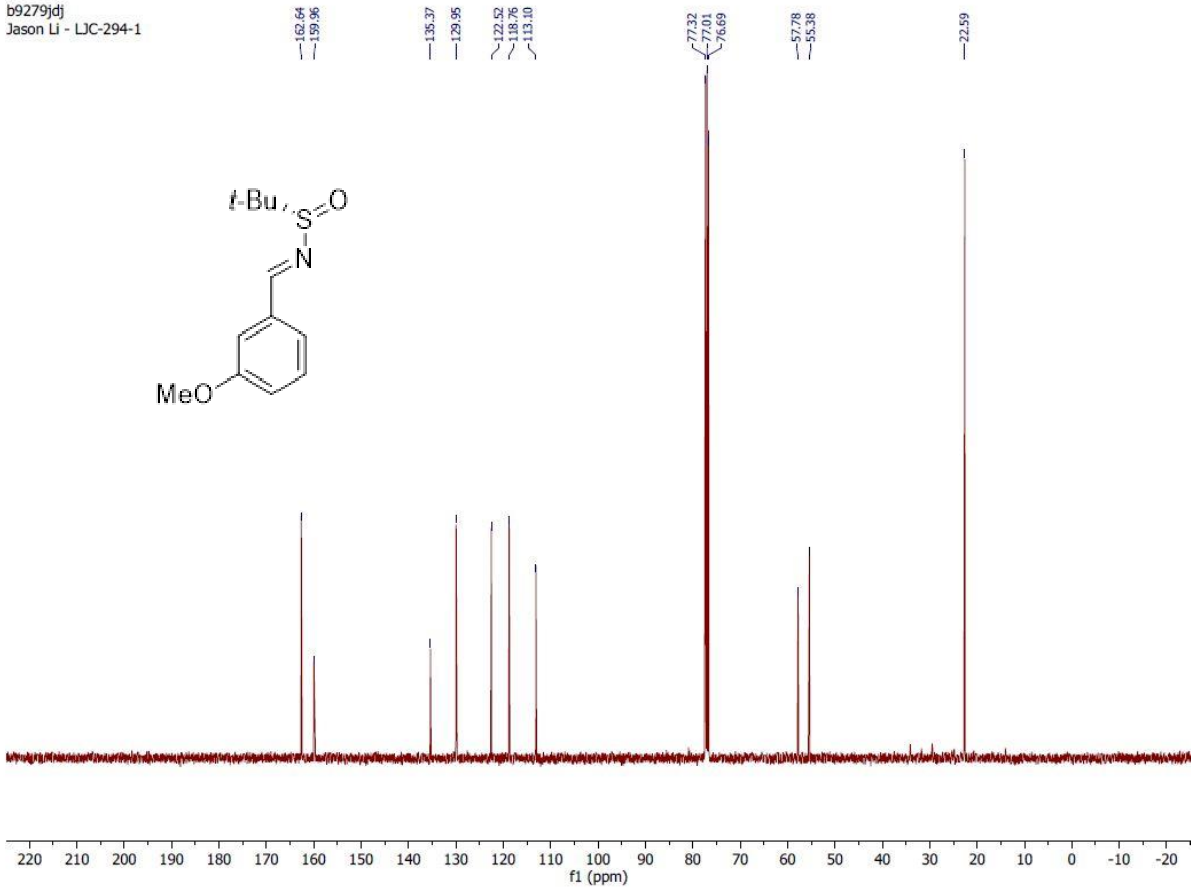

Figure S159: NMR data for **(*S*)-13h**

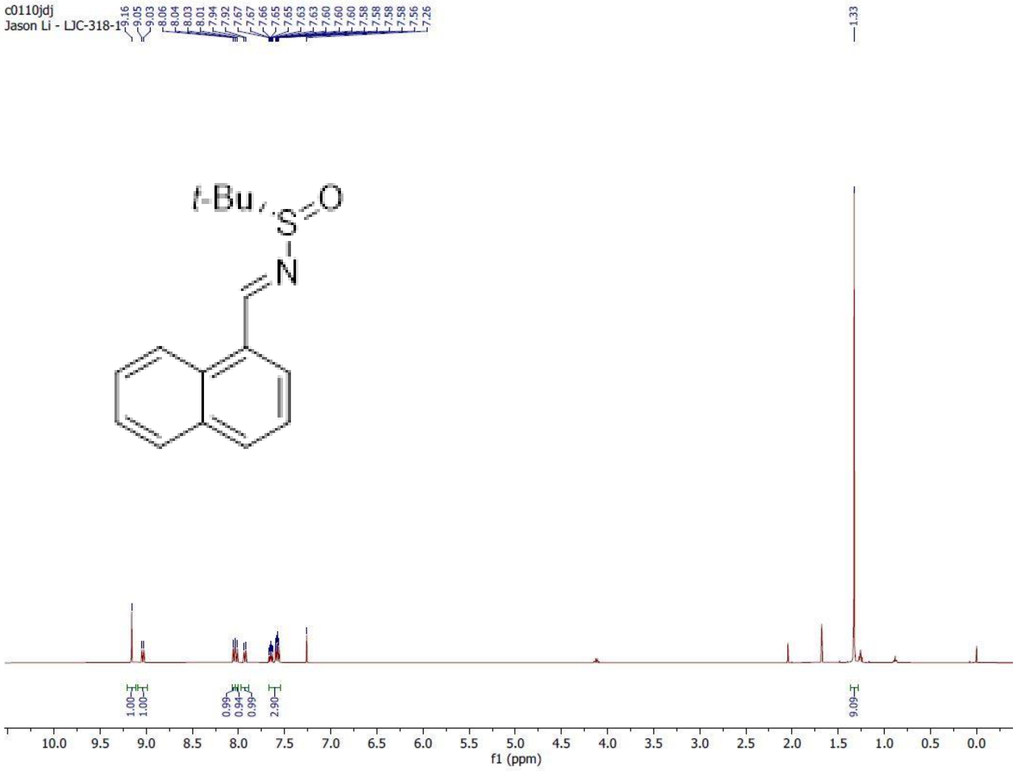

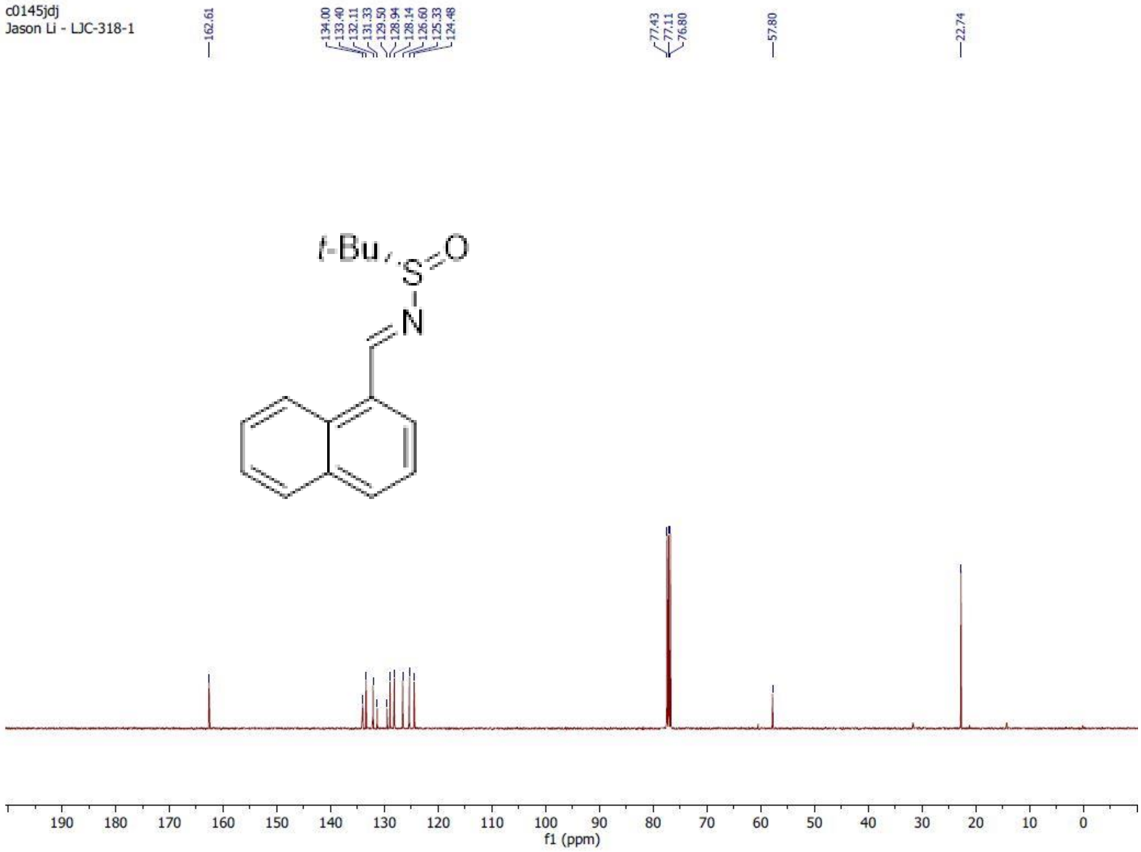

Figure S160: NMR data for **(*S*)-13i**

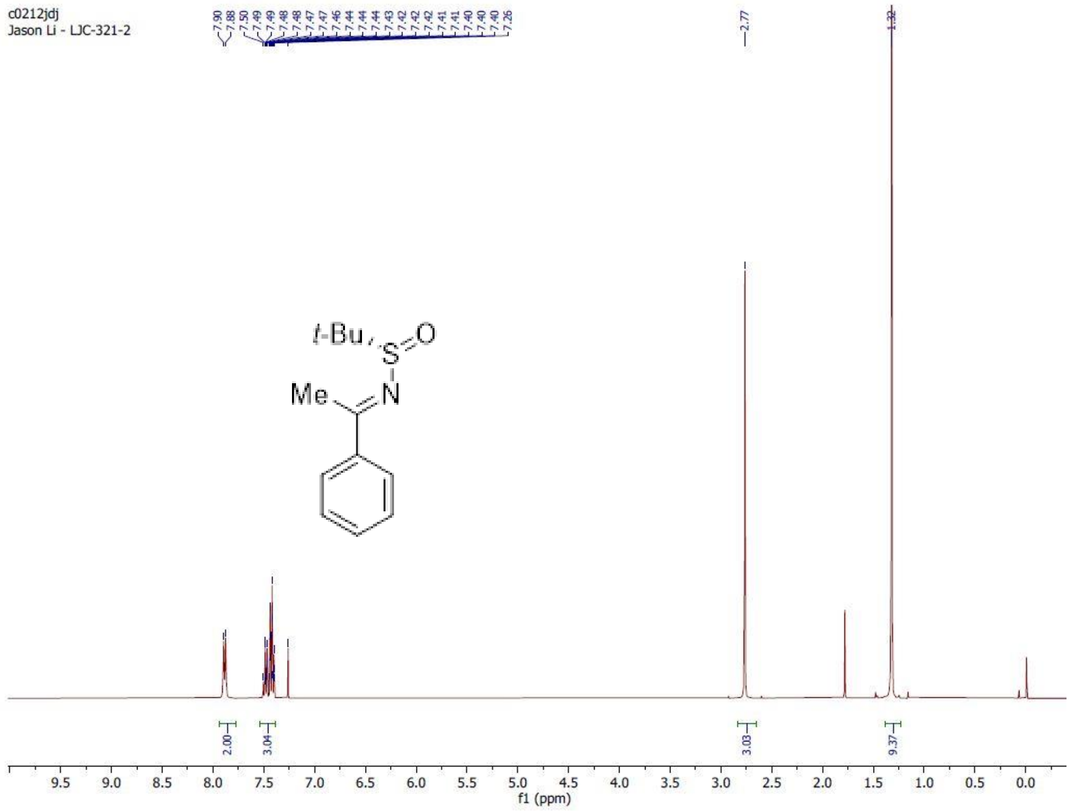

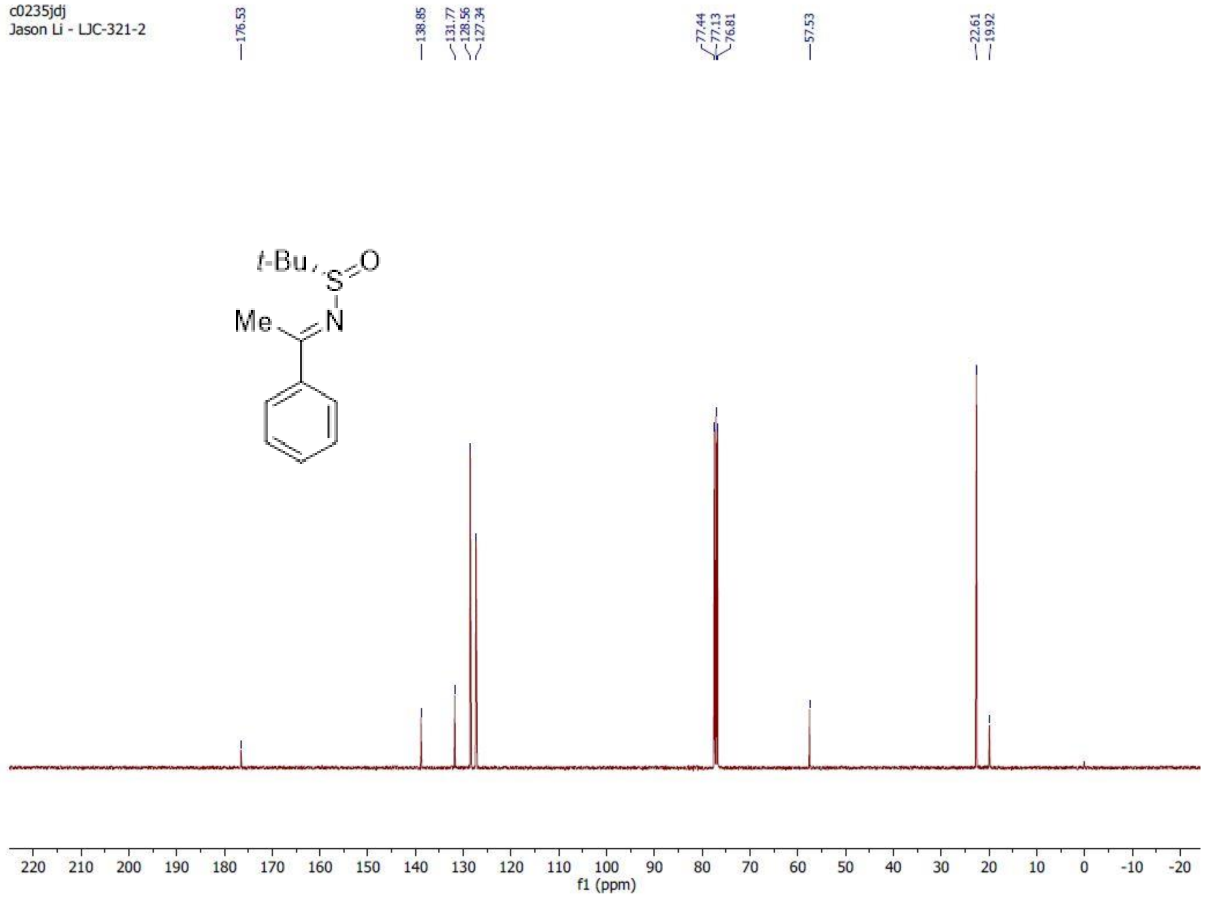

Figure S161: NMR data for **(*S*)-13k**

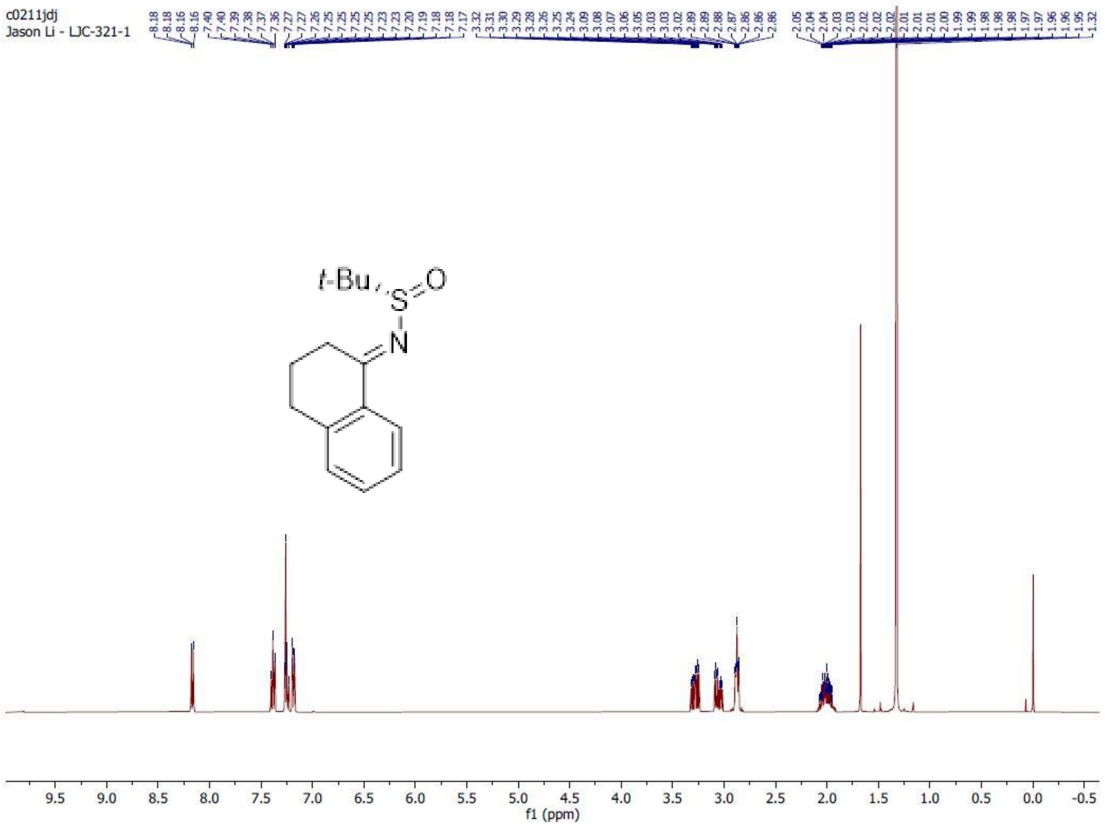

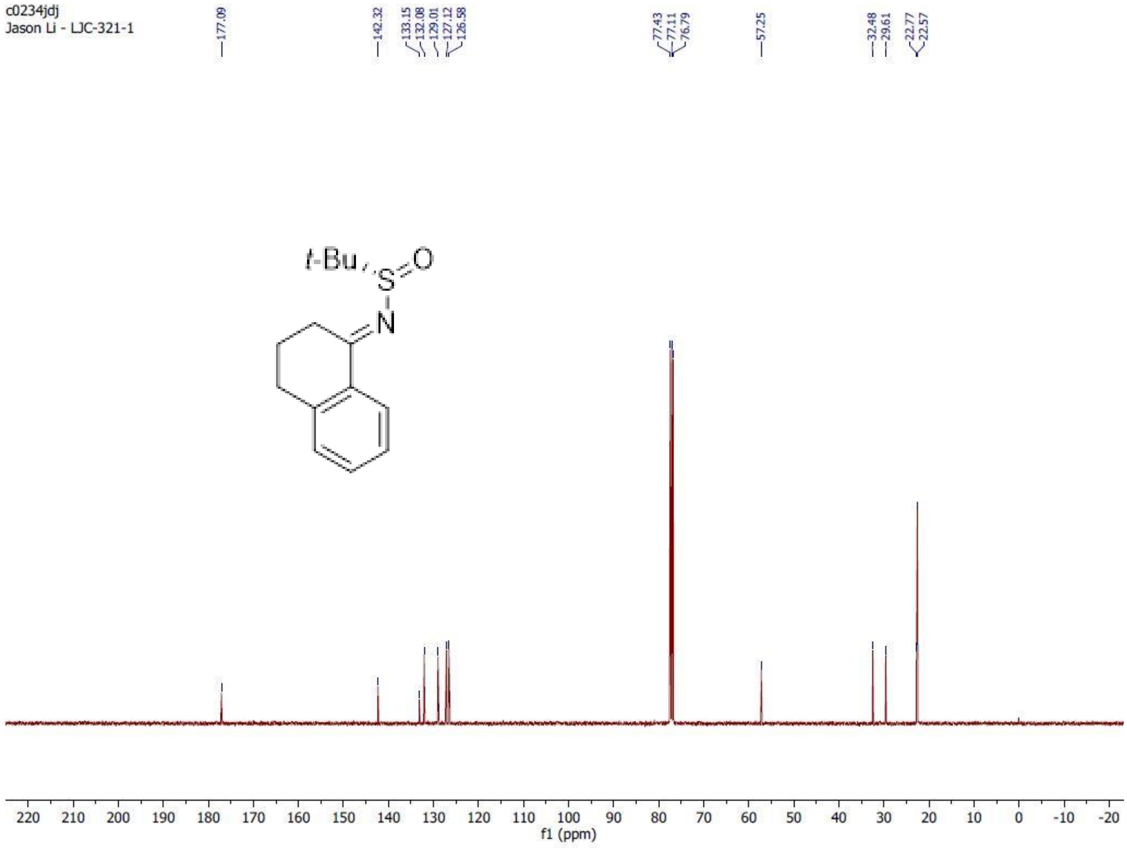

Figure S162: NMR data for **(*S*)-13l**

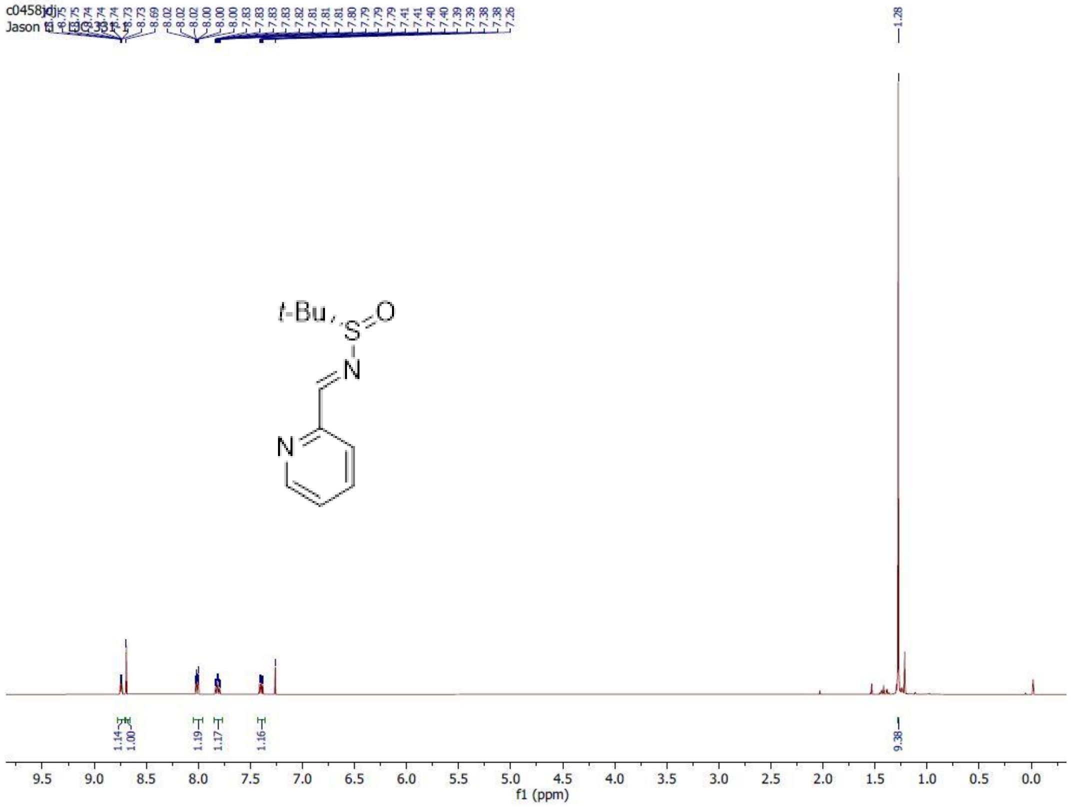

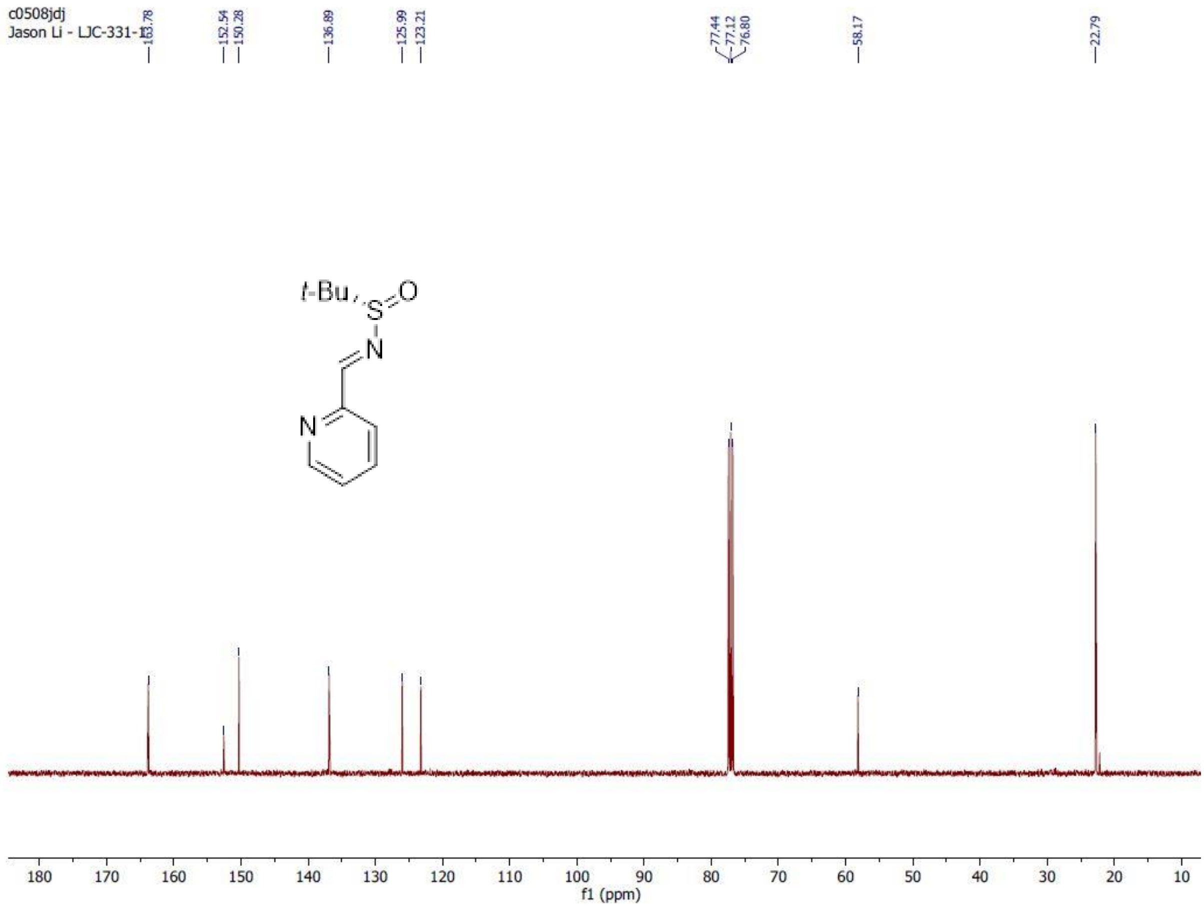

Figure S163: NMR data for **(*S*)-13m**

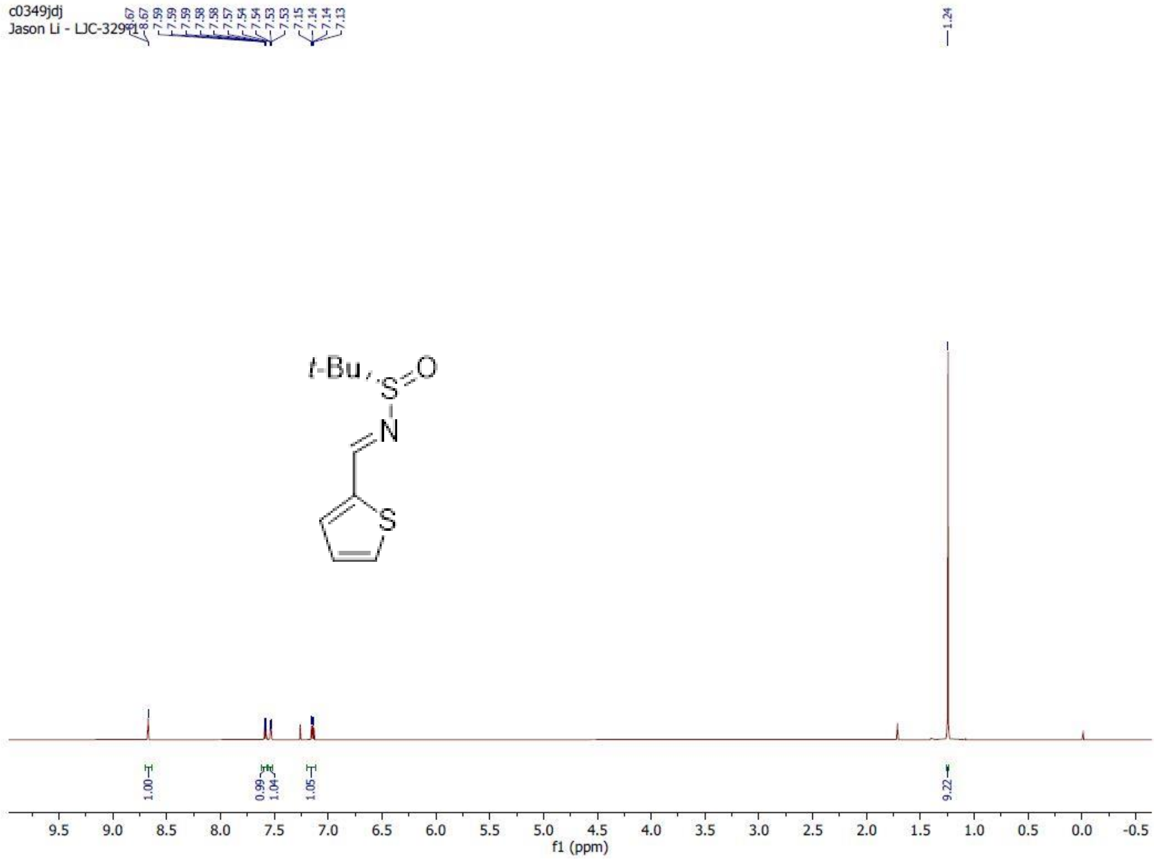

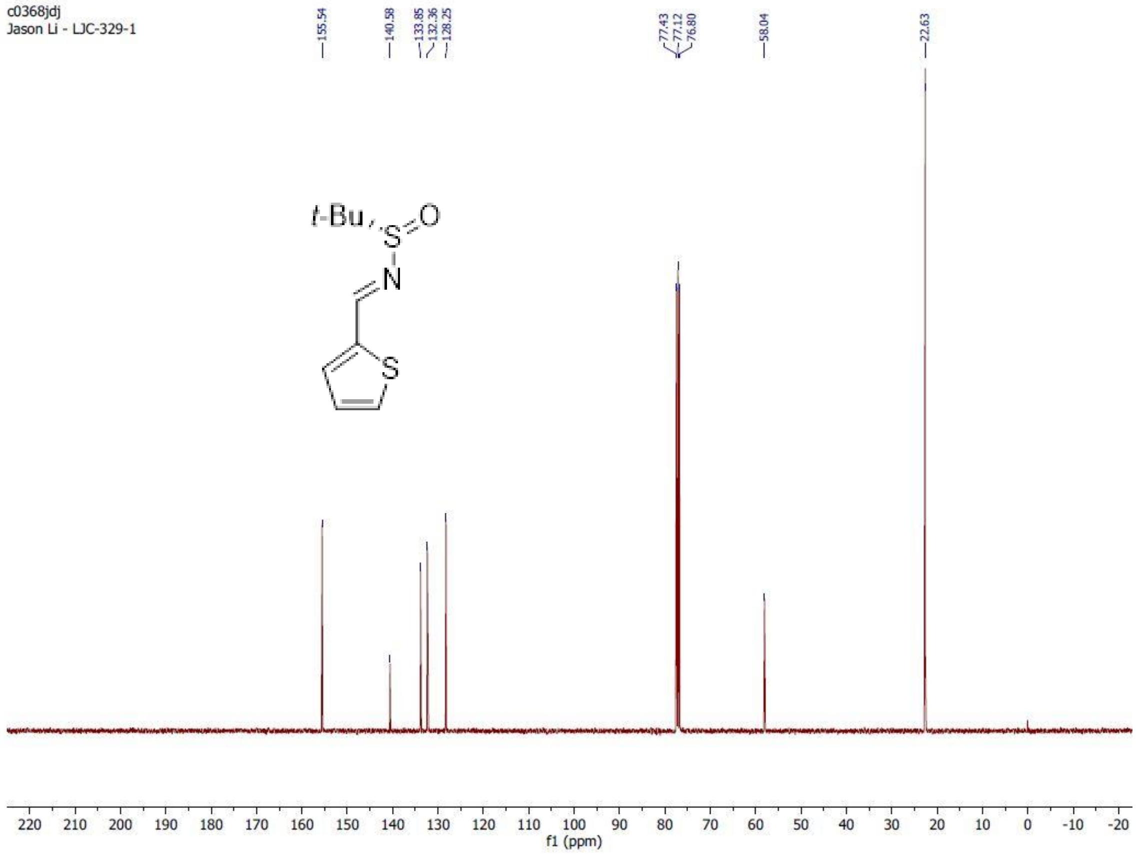

Figure S164: NMR data for **(*S*)-13n**

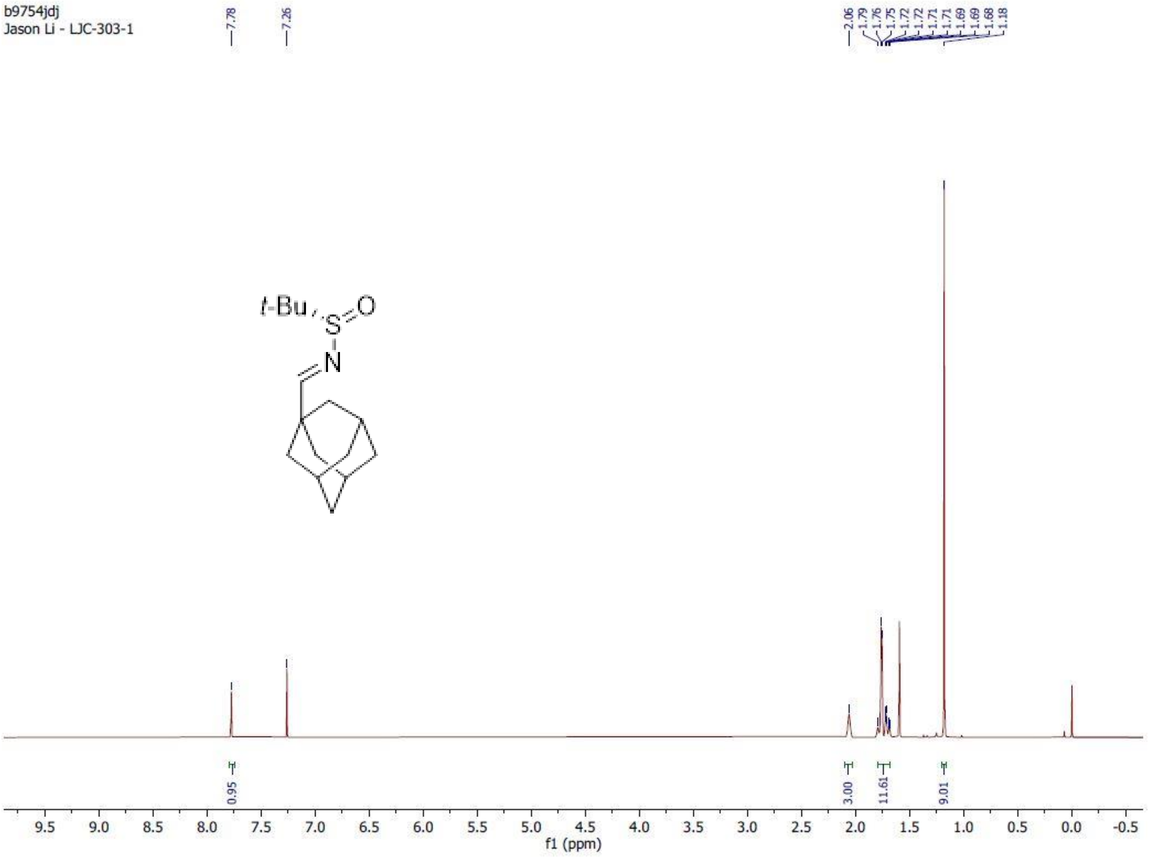

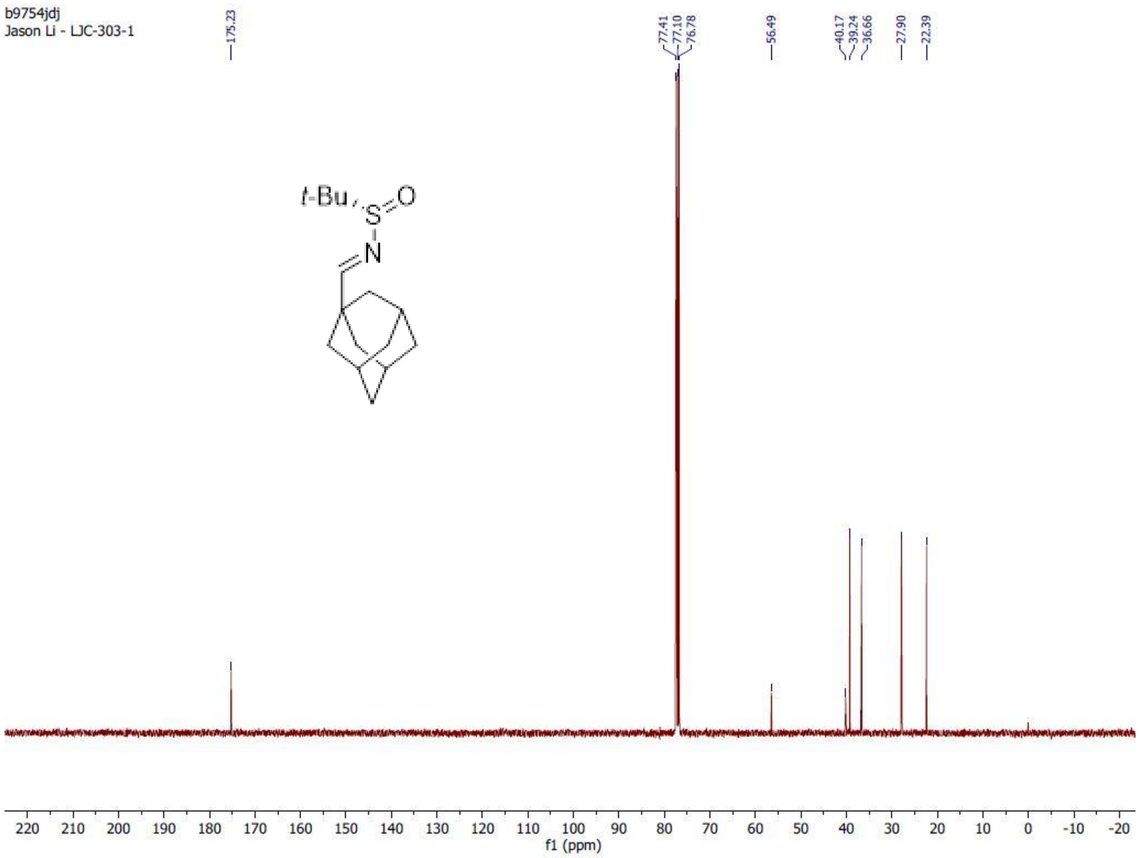

Figure S165: NMR data for **16**

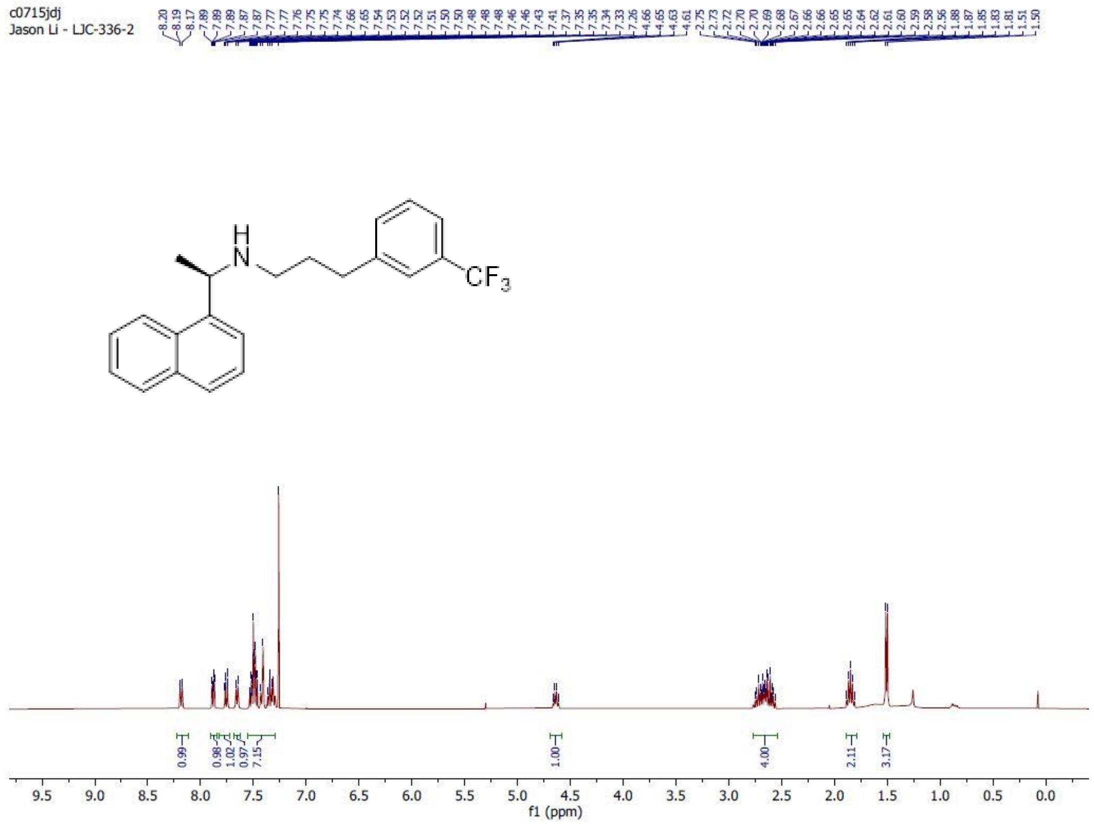

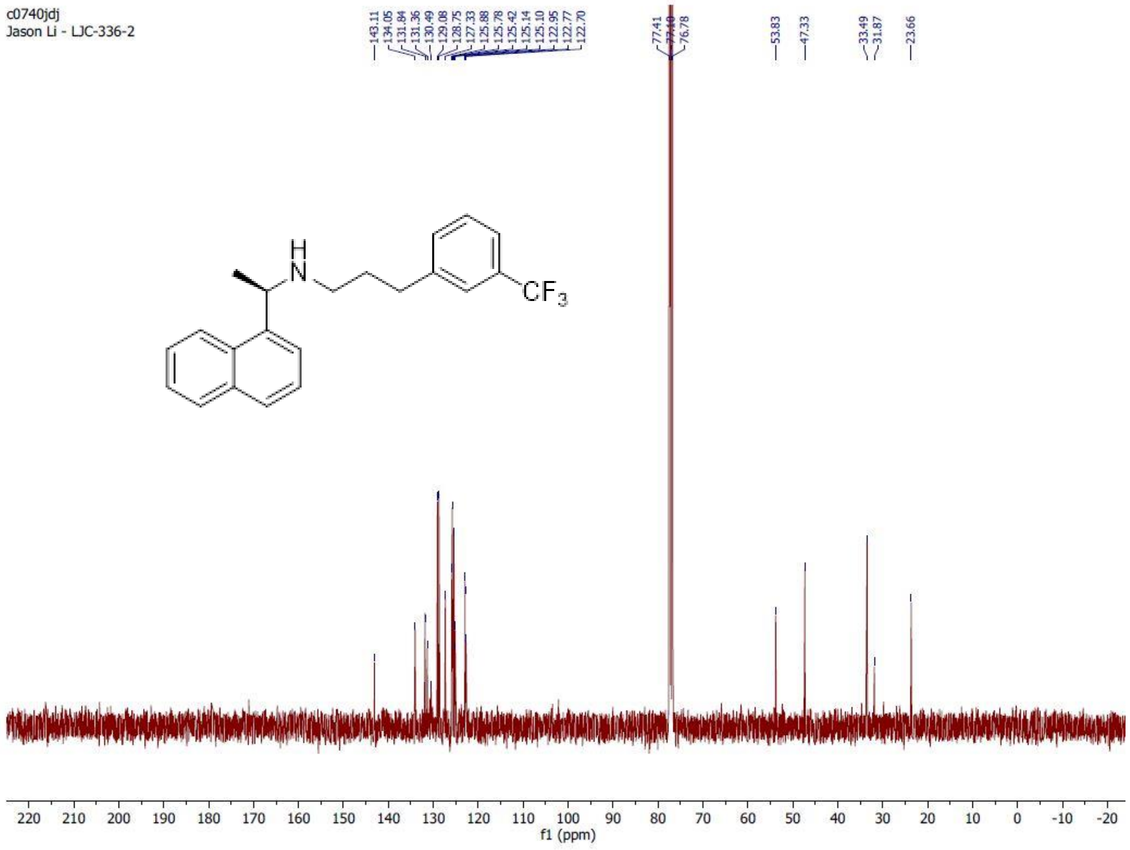

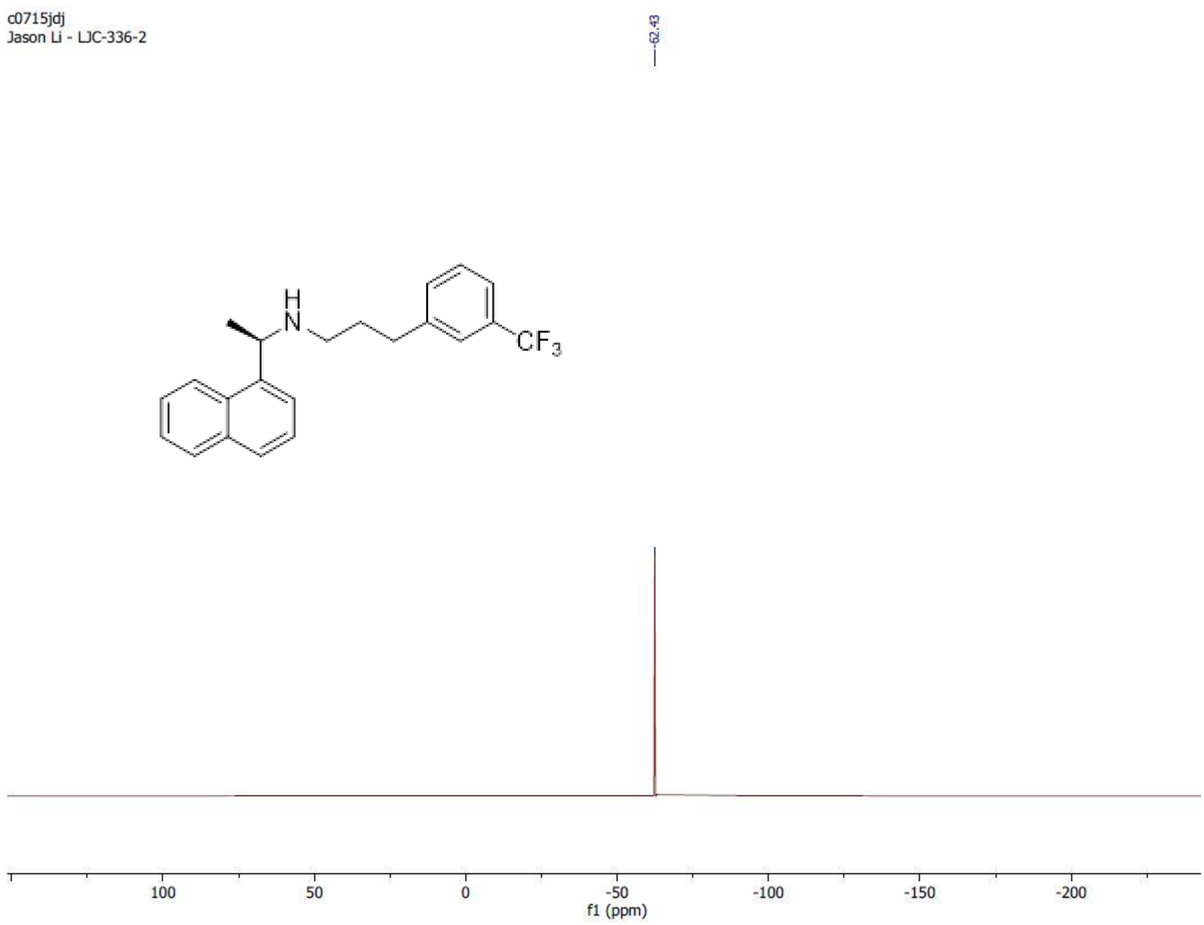
c0715jdj
Jason Li - LJC-336-2
-62.43
H
N
CF3
100
50
0
-50
-100
-150
-200
f1 (ppm)

Figure S166: NMR data for **18**

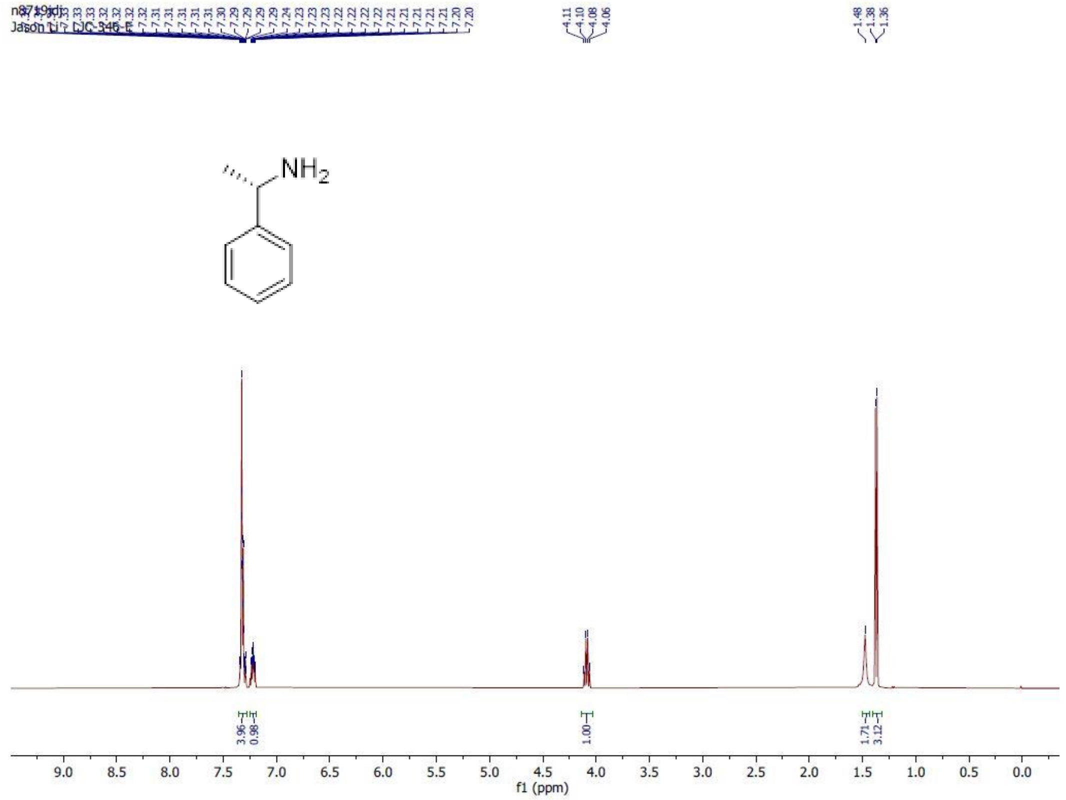

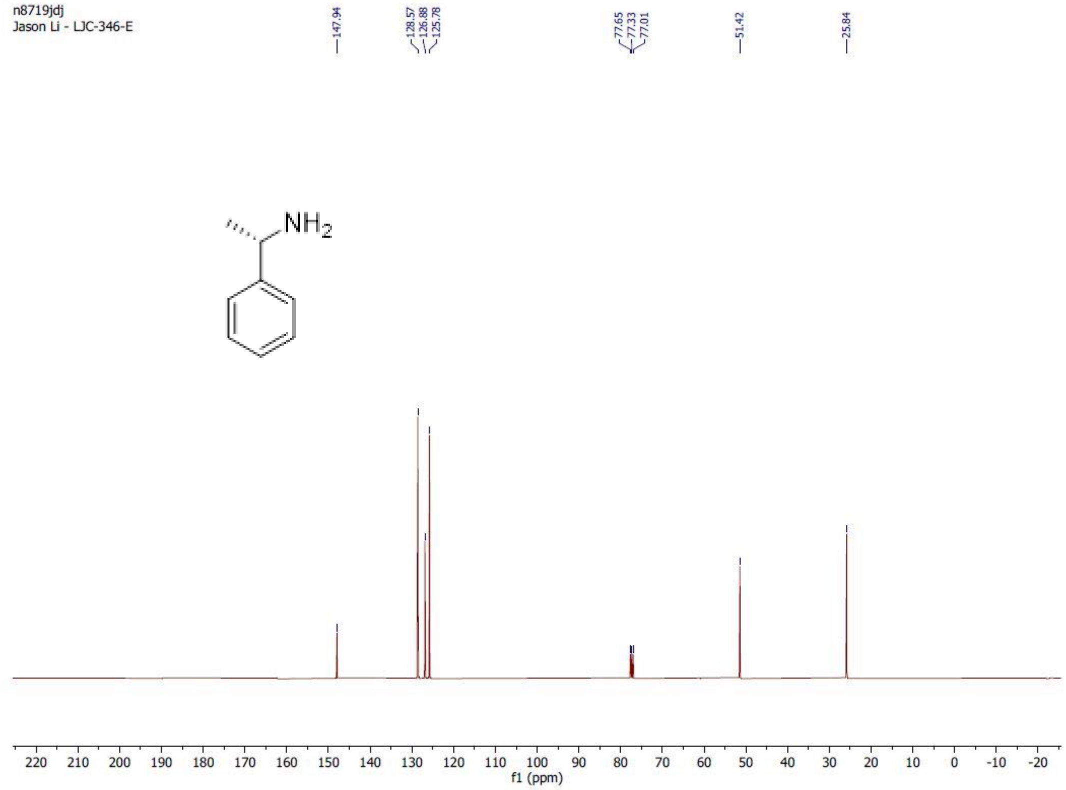

8) Supplementary References

[1] Mancheño, O. G., Bolm, C. Synthesis of *N*-(1*H*)-Tetrazole Sulfoximines. *Org. Lett.* **9**, 2951–2954 (2007).

[2] Kawęcki, R. Synthesis of *N*-Sulfenylimines from Disulfides and Primary Methanamines. *J. Org. Chem.* **87**, 7514–7520 (2022).

[3] Liu. T.-F., Yao, Y., Lu, C.-D. Enantioselective Formation of α-Amino Acid Derivatives *via* [2,3]-Sigmatropic Rearrangement of *N*-Acyl Iminosulfinamides. *Org. Lett.* **25**, 4156–4161 (2023).

[4] Davis, F. A., Friedman, A. J, Nadir, U. K. Chemistry of the sulfur-nitrogen bond. 14. Arenesulfenic acids from *N*-alkylidenearenesulfinamides (sulfinimines). *J. Am. Chem. Soc.* **100**, 2844–2852 (1978).

[5] Hendriks, C. M. M., Lamers, P., Engel, J., Bolm, C. Sulfoxide-to-Sulfilimine Conversions: Use of Modified Burgess-Type Reagents. *Adv. Synth. Catal.* **355**, 3363–3368 (2013).

[6] KIein, M., Waldvogel, S. R. Anodic Dehydrogenative Cyanamidation of Thioethers: Simple and Sustainable Synthesis of *N*-Cyanosulfilimines. *Angew. Chem. Int. Ed.* **60**, 23197–23201 (2021).

[7] Goldberg, F. W., Kettle, J. G., Xiong, J., Lin, D. General synthetic strategies towards *N*-alkyl sulfoximine building blocks for medicinal chemistry and the use of dimethylsulfoximine as a versatile precursor. *Tetrahedron*. **70**, 6613–6622 (2014).

[8a] Barry, N., Brondel, N., Lawrence, S, E., Maguire, A, R. Synthesis of aryl benzyl *N*H Sulfoximines. *Tetrahedron.* **65**, 10660–10670 (2009).

[8b] Brandt, J.; Gais, H.-J. An efficient resolution of (_)-S-methyl-S-phenylsulfoximine with (+)-10-camphorsulfonic acid by the method of half-quantities. *Tetrahedron.* **8**, 902–912 (1997).

[9] Kim, S., Kim, J. E., Lee, J., Lee, P,H. *N*-Imidazolylation of Sulfoximines from *N*-Cyano Sulfoximines, 1-Alkynes, and N-Sulfonyl Azides. *Adv. Synth. Catal.* **357**, 3707–3717 (2015).

[10] Dannenberg, C. A., Fritze, L., Krauskopf, F., Bolm, C. Access to *N*-cyanosulfoximines by transition metal-free iminations of sulfoxides. *Org. Biomol. Chem.* **15**, 1086–1090 (2017).

[11] Reddy, C. M. L., Khan, F. R. N., Saravanan, V. Facile. Synthesis of *N*-1,2,4-oxadiazole Substituted Sulfoximines from *N*-cyano Sulfoximines. *Org. Biomol. Chem.* **17**, 9187 (2019).

[12] Amri, N., Wirth, T. Flow electrosynthesis of Sulfoxides, Sulfones, and Sulfoximines without Supporting Electrolytes. *J. Org. Chem.* **86**, 15961–15972 (2021).

[13] Dong, S., Frings, M., Cheng, H., Wen, J., Zhang, D., Raabe, G., Bolm, C. Organocatalytic Kinetic Resolution of Sulfoximines *J. Am. Chem. Soc.* **138**, 2166−2169 (2016).

[14]. Li, R., Zhou F., Huang X., Zhao J., Zhang H. Pummerer-like Rearrangement Induced Cascade Reactions: Synthesis of Highly Functionalized Imidazoles. *J. Org. Chem*. **88**, 739−744 (2023).

[15] Roe, C., Hobbs, H., Stockman, R. A. Multicomponent Synthesis of Chiral Sulfinimines. *Chem. Eur. J.* **17**, 2704−2708 (2011).

[16] Fernández, I., Valdivia, V., Alcudia, A., Chelouan, A., Khiar, N. Enantiodivergent Approach to Trifluoromethylated Amines: A Concise Route to Both Enantiomeric Analogues of Calcimimetic NPS R-568. *Eur. J. Org. Chem.* **2010**, 1502−1509 (2010).

[17] Petrone, D. A., Yoon H., Weinstabl H., Lautens M. Additive Effects in the Palladium-Catalyzed Carboiodination of Chiral *N*-Allyl Carboxamides. *Angew. Chem. Int. Ed.* **53,** 7908−7912 (2014).

[18] Ramaiah, M. M., Shubha, P. B., Prabhala, P. K., Shivananju, N. S. 1,8-Diazabicyclo[5.4.0]undec-7-ene-mediated formation of *N*-sulfinyl imines. *J. Chem. Res.* **44,** 72−79 (2020).

[19] Reeves, J. T., Visco, M. D., Marsini, M. A., Grinberg, N., Busacca, C. A., Mattson, A. E., Senanayake, C. H. A General Method for Imine Formation Using $B(OCH_2CF_3)_3$. *Org. Lett.* **17**, 2442−2445 (2015).

[20] Kells, K. W, Chong, J. M. Stille Coupling of Stereochemically Defined *α*-Sulfonamidoorganostannanes. *J. Am. Chem. Soc.* **126**, 15666−15667 (2004).

[21] Sanaboina, C., Jana, S., Eppakayala, L. Efficient Microwave-Assisted Synthesis of *N*-(tert-Butylsulfinyl)imines Catalyzed by Amberlist-15. *Synlett.* **25,** 1006−1008 (2014).

[22] Vazquez-Chavez, J., Luna-Morales, S., Cruz-Aguilar, D. A., Díaz-Salazar, H., Vallejo Narváez W. E., Silva-Gutiérrez, R. S. *et al.* The effect of chiral *N*-substituents with methyl or trifluoromethyl groups on the catalytic performance of mono- and bifunctional thioureas. *Org. Biomol. Chem*. **17**, 10045−10051 (2019).

[23] Sirvent, J. A., Foubelo, F., Yus, M. Diastereoselective indium-mediated allylation of *N*-tert-butanesulfinyl ketimines: easy access to asymmetric quaternary stereocenters bearing nitrogen atoms. *Chem. Commun.* **48,** 2543−2545 (2012).

[24] Alexeev, M. S., Strelkova, T. V., Ilyin, M. M., Nelyubina, Y. V., Bespalov, I. A., Medvedev, M. G. *et al.* Amine adducts of triallylborane as highly reactive allylborating agents for Cu(i)-catalyzed allylation of chiral sulfinylimines. *Org. Biomol. Chem*. **22**, 4680−4696 (2024).

[25] Wang, L., Chen, M., Zhang, P., Li, W., Zhang, J. Palladium/PC-Phos-Catalyzed Enantioselective Arylation of General Sulfenate Anions: Scope and Synthetic Applications. *J. Am. Chem. Soc.* **140**, 3467−3473 (2018).

[26] WO2017006282A1·2017-01-12.

[27] US2008261926A1·2008-10-23.

[28] Arava, V. R., Gorentla, L., Dubey, P. K. A novel asymmetric synthesis of cinacalcet hydrochloride. *Beilstein J. Org. Chem.* **8,** 1366−1373 (2012).

[29] Bottari, G., Afanasenko, A., Castillo-Garcia, A. A., Feringa, B. L., Barta, K. Synthesis of Enantioenriched Amines by Iron-Catalysed Amination of Alcohols Employing at Least One Achiral Substrate. *Adv. Synth. Catal*. **363**, 5436−5442 (2021).

[30] Adamkiewicz, A., Mlynarski, J. Diastereoselective Hydrosilylation of *N-(*tert-Butylsulfinyl)imines Catalyzed by Zinc Acetate. *Eur. J. Org. Chem*. **2016,** 1060−1065 (2016).

[31] Bonfield, H. E., Mercer, K., Diaz-Rodriguez, A., Cook, G. C., McKay, B. S. J., Slade, P., Taylor, G. M., Ooi, W. X., Williams, J. D., Roberts, J. P. M., et al. (2020). The Right Light: De Novo Design of a Robust Modular Photochemical Reactor for Optimum Batch and Flow Chemistry. ChemPhotoChem 4, 45-51. 10.1002/cptc.201900203.

[32] Robinson, W. X. Q., Mielke, T., Melling, B., Cuetos, A., Parkin, A., Unsworth, W. P.*, et al.* Comparing the Catalytic and Structural Characteristics of a 'Short' Unspecific Peroxygenase (UPO) Expressed in Pichia pastoris and Escherichia coli. *ChemBioChem.* **24**, e202200558 (2023).

[33] Trott, O., Olson, A. J. AutoDock Vina: improving the speed and accuracy of docking with a new scoring function, efficient optimization, and multithreading. *J Comput Chem.* **31**, 455−461 (2010).

[34] Moriarty, N. W., Grosse-Kunstleve, R. W., Adams, P. D. electronic Ligand Builder and Optimization Workbench (eLBOW): a tool for ligand coordinate and restraint generation. *Acta Crystallogr D Biol Crystallogr.* **65**, 1074−1080 (2009).

[35] Liebschner D, Afonine PV, Baker ML, Bunkóczi G, Chen VB, Croll TI*, et al.* Macromolecular structure determination using X-rays, neutrons and electrons: recent developments in Phenix. *Acta Crystallogr D Struct Biol.* **75**, 861−877 (2019).

[36] Ramirez-Escudero, M., Molina-Espeja, P., Gomez de Santos, P., Hofrichter, M., Sanz-Aparicio, J., Alcalde, M. Structural Insights into the Substrate Promiscuity of a Laboratory-Evolved Peroxygenase. *ACS Chem Biol.* **13**, 3259−3268 (2018).

[37] Schlichting, I., Berendzen. J., Chu. K., Stock, A. M., Maves, S. A., Benson, D. E., *et al.* The Catalytic Pathway of Cytochrome P450cam at Atomic Resolution. *Science.* **287**, 1615−1622 (2000).
